# Breast Cancer Clinical Trial of Chemotherapy and Trastuzumab: Potential Tool to Identify Cardiac Modifying Variants of Dilated Cardiomyopathy

**DOI:** 10.3390/jcdd4020006

**Published:** 2017-05-04

**Authors:** Daniel J. Serie, Julia E. Crook, Brian M. Necela, Bianca C. Axenfeld, Travis J. Dockter, Gerardo Colon-Otero, Edith A. Perez, E. Aubrey Thompson, Nadine Norton

**Affiliations:** 1 Health Sciences Research, Mayo Clinic, Jacksonville, FL 32224, USA; serie.daniel@mayo.edu (D.J.S.); crook.julia@mayo.edu (J.E.C.); 2 Department of Cancer Biology, Mayo Clinic, Jacksonville, FL 32224, USA; necela.brian@mayo.edu (B.M.N.); axenfeld.bianca@mayo.edu (B.C.A.); thompson.aubrey@mayo.edu (E.A.T.); 3 Health Sciences Research, Mayo Clinic, Rochester, MN 55905, USA; dockter.travis@mayo.edu; 4 Hematology/Oncology, Mayo Clinic, Jacksonville, FL 32224, USA; gcolonotero@mayo.edu (G.C.-O.); perez.edith@mayo.edu (E.A.P.)

**Keywords:** dilated cardiomyopathy, modifying variants, GWAS, LVEF, OBSCN, breast cancer, trastuzumab, doxorubicin

## Abstract

Doxorubicin and the ERBB2 targeted therapy, trastuzumab, are routinely used in the treatment of HER2+ breast cancer. In mouse models, doxorubicin is known to cause cardiomyopathy and conditional cardiac knock out of Erbb2 results in dilated cardiomyopathy and increased sensitivity to doxorubicin-induced cell death. In humans, these drugs also result in cardiac phenotypes, but severity and reversibility is highly variable. We examined the association of decline in left ventricular ejection fraction (LVEF) at 15,204 single nucleotide polymorphisms (SNPs) spanning 72 cardiomyopathy genes, in 800 breast cancer patients who received doxorubicin and trastuzumab. For 7033 common SNPs (minor allele frequency (MAF) > 0.01) we performed single marker linear regression. For all SNPs, we performed gene-based testing with SNP-set (Sequence) Kernel Association Tests: SKAT, SKAT-O and SKAT-common/rare under rare variant non-burden; rare variant optimized burden and non-burden tests; and a combination of rare and common variants respectively. Single marker analyses identified seven missense variants in *OBSCN* (*p* = 0.0045–0.0009, MAF = 0.18–0.50) and two in *TTN* (both *p* = 0.04, MAF = 0.22). Gene-based rare variant analyses, SKAT and SKAT-O, performed very similarly (*ILK*, *TCAP*, *DSC2*, *VCL*, *FXN*, *DSP* and *KCNQ1*, *p* = 0.042–0.006). Gene-based tests of rare/common variants were significant at the nominal 5% level for *OBSCN* as well as *TCAP*, *DSC2*, *VCL*, *NEXN*, *KCNJ2* and *DMD* (*p* = 0.044–0.008). Our results suggest that rare and common variants in *OBSCN*, as well as in other genes, could have modifying effects in cardiomyopathy.

## 1. Introduction

Dilated cardiomyopathy (DCM) is the underlying cause of >50% of heart transplants. The high morbidity and mortality associated with this disease underscore the need for a better understanding of the underlying molecular defects. Efforts to identify these defects have made great progress and acknowledge the complexity of the genetic architecture of DCM. Historically, however, they have relied on a somewhat circular argument: DCM genetic cause is described as predominantly autosomal dominant transmission with reduced penetrance, a high degree of locus (>40 genes) and allelic (>197 variants) heterogeneity [1], with most mutations being rare or “private” to each affected family. However, proof of variant pathogenicity within a family, at least for publication in the scientific literature, relies largely on fulfilment of these criteria. In total, ~40% of individuals test positive at known DCM loci [2,3] and even within families who fulfil these criteria, age at onset, response to treatment and disease progression is variable [4]. Despite this fact, the search for the ‘missing’ genetic cause and definition of pathogenicity relies predominantly on a familial-based strategy and criteria, making it particularly difficult to demonstrate pathogenicity in sporadic cases, even when all possible causes other than genetic, (coronary artery disease, chemotherapy-induced cardiotoxicity, valvular disease or repair) are ruled out.

Clearly, the traditional Mendelian paradigm as the causative genetic contribution to DCM genomic architecture is incomplete, and there is a need for alternative and perhaps novel genomic strategies. Genetic association studies of sporadic cases provide a potential alternative. In the first reported genome-wide association study of DCM cases (1179 cases and 1108 controls), common variants at two loci (*BAG3* and *HSP7B*) were associated at genome-wide significance [5]. These data were encouraging because one of the top loci, *BAG3*, was also identified as a DCM gene in multiply affected families [6]. These overlapping but independent reports of common genetic variants (Allele frequency = 87.5% in sporadic, “idiopathic” DCM cases and 79.2% in controls, OR = 1.89) associated with increased disease risk at a DCM locus that was originally determined by Mendelian rare variant criteria, are not hugely surprising, lending weight and confidence for further investigation of known DCM genes and common risk variants. The caveat of such an approach is that DCM is a common disease (prevalence now estimated at 1:250) and often late onset [4]. Many members of the general population (potentially used as controls) could be asymptomatic, but due to expense, echocardiographic screening of large control populations is unlikely. In this study, we present an alternative approach to identify potential common risk variants, using a large clinical trial of breast cancer patients.

We postulate that phenotypic variability between individuals with the same DCM mutation is the result of cardiac modifying variants (i.e., we hypothesize that the severity of known DCM mutations could be influenced by individual genetic background). Differences in genetic background have been observed in animal models of DCM. For example, conditional knock-out of cardiac *Erbb2* in two different lines of mice resulted in a DCM phenotype in both lines, but one showed much later disease onset [7]. However, the homogeneity within such models can be a disadvantage when extrapolating to the human population, and strategies to tease out the human genetic architecture of DCM are required. For example, *ERBB2* itself is not a known DCM gene in the Mendelian sense, but it is the target of the monoclonal antibody and breast cancer drug, trastuzumab (Herceptin), the current standard of care for HER2+ breast cancer patients [8]. In Vitro assays of trastuzumab and human iPSC-derived cardiomyocytes demonstrate complete loss of ERBB2 within 48 h [9], a close parallel between the mouse conditional knock-out model and use of trastuzumab in human patients. Indeed, in the first clinical trial of trastuzumab in the metastatic setting [10], the major clinical side-effect was congestive heart failure in up to 27% of patients, although notably, this figure related to patients who received trastuzumab following anthracyclines, already a well-known cause of dose-dependent, irreversible heart failure, often ending in a phenotype of cardiomyopathy [11]. Nonetheless, the incidence of cardiac events was considerably higher in patients who received both anthracycline and trastuzumab than in patients who received anthracycline alone, hence subsequent trials of trastuzumab employed serial echocardiographic monitoring of patients. These patients may represent an important population to identify cardiac modifying variants because: (1) Phase III clinical trials are typically large (N = 1000’s); (2) Patients receive echocardiography as a standard of care, with left ventricular ejection fraction (LVEF) monitoring at baseline, throughout treatment and on completion of treatment; (3) Patients must have baseline LVEF >50% to be eligible for trastuzumab, so unlikely to be asymptomatic prior to treatment; (4) The average age of breast cancer patients entered into phase III trials of Herceptin was >60 years, hence more age representative of DCM patients in the general population; (5) Family history of dilated cardiomyopathy is a risk factor for anthracycline-induced cardiomyopathy [12,13], suggesting an overlap between disease development following chemotherapy and genetic variants at DCM loci.

In this study, we analyzed the association of genetic variants across 72 known cardiomyopathy genes with decline in LVEF in 800 patients from the N9831 clinical trial [8]. All patients in this group were treated with doxorubicin and trastuzumab. We report results of single variant associations of common genetic variants (minor allele frequency (MAF) > 0.01) as well as those of gene-based association testing. These analyses highlight genetic variants at *OBSCN*, *ILK*, *TCAP*, *DSC2*, *VCL*, *FXN*, *DSP* and *KCNQ1* as potential cardiac modifying variants that may be relevant to the development or progression of cardiomyopathy. 

## 2. Materials and Methods

**Figure 1:**
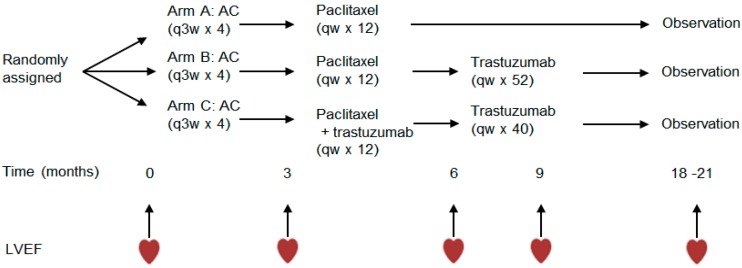
Treatment Arms and cardiac monitoring of N9831 clinical trial. A, Doxorubicin 60 mg/m^2^; C, Cyclophosphamide 600 mg/m^2^; paclitaxel 80 mg/m^2^; trastuzumab 4 mg/kg loading dose followed by 2 mg/kg weekly (qw); LVEF, left ventricular ejection fraction.

N9831 Clinical Trial: N9831 was a pivotal clinical trial that led to the use of trastuzumab as the standard of care for early HER2+ breast cancer. Patients in the N9831 trial were required to have histologically confirmed adenocarcinoma of the breast with 3+ immunohistochemical staining for HER2 or amplification of the HER2 gene by fluorescence in situ hybridization (≥2.0 ratio) and with either lymph node-positive or high-risk lymph node-negative disease to be eligible for the study. The trial compared adjuvant chemotherapy only (Arm A) vs. adjuvant chemotherapy followed by trastuzumab, either sequentially (Arm B) or concurrently (Arm C), in operable HER2+ breast cancer [8,14]. Patients received serial echocardiograms (ECHO) or multigated acquisition scans (MUGA) for up to 6-years: at baseline, at 3, 6, and 9 months after registration, and after completion of chemotherapy (Figure 1). Long-term cardiac safety analysis was completed in 2016 [15]. The most common cardiac symptom was decline in LVEF by ≥10 points, observed in 26.2% of patients in Arm A (chemotherapy only) and 37.3% of patients who received trastuzumab (Arms B and C). Prevalence of congestive heart failure (CHF) was also significantly higher in patients receiving trastuzumab (3%) compared to those receiving chemotherapy only (0.9%) [15]. The majority of patients who developed CHF received cardiac medications, which included diuretics, beta-blockers, and angiotensin-converting enzyme inhibitors. 

DNA extraction and genotyping: Genomic DNA was available for a total of 1446 patients from the trial. DNA was isolated from peripheral blood with the Flexigene kit (Qiagen Inc, Germantown, MD, USA) as per the manufacturer’s instructions, normalized to 15 ng/μL and shipped to Affymetrix (Affymetrix Inc, Santa Clara, CA, USA) for full service genotyping. Each 96-well plate contained one duplicate patient sample and two DNA samples routinely used as positive controls by Affymetrix. Genotyping was performed using a customized Axiom genotyping array (Affymetrix Inc, Santa Clara, CA, USA) covering a total of 762,792 single nucleotide polymorphisms (SNP)s. 

A total of 16 duplicate controls were nested within 1462 DNA samples (1446 unique samples, one duplicate pair per 96-well plate) yielding 100% genotyping concordance across 793,571 SNPs. Primary analyses were confined to White/non-Hispanic with complete LVEF data. A total of 188 patients were reported as non-White/Hispanic and principal components analyses identified a further 27 outliers, and 40 patients were missing either baseline or post-treatment LVEF, leaving 1191 patients for analyses (Arm A, N = 391; Arms B + C, N = 800), Supplementary Figure S1. Custom shell and R programming was employed to put these data in PLINK format, and all quality control (QC) was done using PLINK 1.07. 

No samples had a call-rate under 95%. 13,987 SNPs had a call-rate under 95% and were removed from further analyses. Of the remaining 779,584 SNPs, 160,721 had MAF < 1%.

Deviation of the genotype distributions from Hardy–Weinberg equilibrium was tested in those patients whose LVEF did not drop by >10% to below 50%. All SNPs with Fisher’s exact test for Hardy–Weinberg Equilibrium *p* < 1.0 × 10**^−^**^4^ were excluded.

Principal components were calculated on 277,190 independent SNPs (none within a moving window of 50 SNPs could have a variance inflation factor (VIF) > 2) to assess correlation with self-reported race. The set of independent SNPs was also used to determine relatedness. There was no cryptic relatedness apart from duplicates; in total, 18 non-control pairs of samples were considered identical based on high PI_HAT (a PLINK statistic based on estimated IBD) and concordance values.

Gene and SNP selection: In this study, we focus on known DCM genes from the current literature. We report single marker association of common genetic variants (MAF > 0.01) at 72 loci (Table 1), of which 71 are listed in the review of DCM genetic architecture [4] and one additional gene, obscurin (*OBSCN*, more recently identified as a DCM gene) [16] and gene-based analyses which include both common (MAF > 0.01) and rare (MAF < 0.01) SNPs. The Affymetrix Axiom genotyping GWAS platform has the option to include custom-based SNPs on a GWAS backbone. We included custom SNPs for all 71 genes in the Hershberger DCM review [4]. We did not include custom SNPs at the *OBSCN* locus, as the array was designed prior to publication of [16]. The study included a total of 15,203 variants at these 72 loci (median SNPs per gene = 68, range 1–3512, interquartile range = 178), of which, 7018 had MAF > 0.01. Each gene and the number of SNPs per gene are listed in Supplementary Table S1. 

Definition of cardiotoxicity: Several oncology and cardiology organizations provide definitions for cardiotoxicity that encompass overt clinical events and subclinical injury, although there is no universally accepted clinical cut point [17]. The 2014 American Society of Echocardiography and the European Association of cardiovascular imaging consensus defined CTRCD as a decrease in the LVEF of >10%, to <53% [11]. Reports of cardiotoxicity in the literature range in LVEF from <50% to <55%, in some cases requiring decreases of >15% or 20% [18]. We aimed to avoid the arbitrary nature of this definition by using as our primary endpoint, the maximum decline in LVEF observed from baseline during follow-up until three months after discontinuation of trastuzumab or until two years post-treatment, whichever was earliest. 

Statistical analyses: Single SNP statistical analyses were performed for 7033 common variants (MAF ≥ 0.01), using R version 3.1.1, PLINK version 1.07. Linear regression was used with change in LVEF (lowest recorded LVEF—baseline LVEF) as the outcome variable and the number of copies of the minor allele of the variant of interest as the primary predictor variable. Analyses were adjusted for age, baseline LVEF, anti-hypertensive medications and the first two principal components in the 800 patients in Arms BC who received chemotherapy (doxorubicin, cyclophosphamide and paclitaxel) and trastuzumab.

The study included a total of 15,203 variants at 72 genes/SNP-sets (median SNPs per gene = 68, range 1–3512, interquartile range = 178), of which, 7018 had MAF > 0.01. Each gene and the number of SNPs in each gene set are listed in Supplementary Table S1. Gene-based statistical analyses were performed by aggregation of individual test-score statistics for each of the 72 gene sets to compute gene-based level *p*-values, while adjusting for age, baseline LVEF, anti-hypertensive medications and the first two principal components with the SNP-set (Sequence) Kernel Association Test (SKAT). Three variations of this test were performed: SKAT [19], SKAT-O [20] and SKAT-common/rare [21] under: (1) Rare variant non-burden (more powerful when a large fraction of the variants in a gene are non-causal or the effects of causal variants are in different directions); (2) Rare variant optimized burden (more powerful when most variants in a region are causal and the effects are in the same direction) and non-burden tests; (3) combination of rare and common variants respectively (weighting rare and common variants equally).

## 3. Results

### 3.1. Single Marker Analyses of Common Variants

In total, 13/72 genes: *VCL*, *DMD*, *OBSCN*, *RYR2*, *TPM1*, *KCNQ1*, *JAG1*, *SGCD*, *SCN5A*, *RBM20*, *SCN4B*, *TTN* and *CACNA1C* showed at least one SNP (MAF > 0.01) with evidence of association with chemotherapy- and trastuzumab-induced decline in LVEF, *p* < 0.05: (Table 2). 

The most significant association was a *DMD* intronic variant, rs12559939, *p* = 0.0005. This association is supported by a highly correlated (r^2^ = 0.90, D’ = 0.95) flanking intronic SNP within 3 kb, rs141927233, *p* = 0.0006, MAF = 0.19, both with relatively small effect size, β = 1.48 and 1.45, respectively. As our analysis was based on an additive model, and the change in LVEF response variable was negative or zero (by definition), this would suggest that for these SNPs each copy of the minor allele results in a smaller decline in LVEF following combination doxorubicin and trastuzumab, i.e., if the association is true, the minor allele is protective against therapy-induced decline in LVEF.

**Figure 2:**
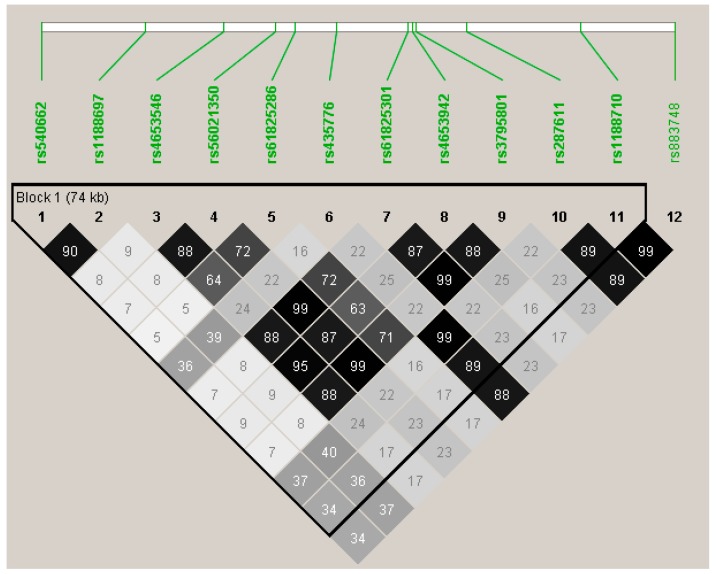
*OBSCN* variants, *p* < 0.05, linkage disequilibrium plot. Three missense variants were significant at *OBSCN* following Bonferroni correction for testing 55 variants at this locus: rs56021350/Thr4399Met, rs4653942/Arg4534His and rs1188710/Gln5891Glu, (*p* = 0.0009, 0.0007 and 0.0008 respectively). rs56021350 and rs4653942 are in linkage disequilibrium, but clearly, rs1188710 is not correlated with these variants, suggesting multiple independent variants at this locus.

We next looked within the common variant analyses for associated missense variants. Missense variants in two genes, *OBSCN* and *TTN*, were significantly associated at the *p* < 0.05 level with decline in LVEF. In total, 12 of the 55 *OBSCN* variants were associated with change in LVEF, seven of which were missense variants (Table 2) with minor allele frequencies ranging from 0.14 to 0.50; all had small estimated effect sizes, ranging from −1.43 to 1.05. Under Bonferroni correction, genotyping 55 SNPs at this locus would require a *p*-value of 0.0009 to remain significant after correction. Three missense variants reached this criteria: rs56021350 Thr4399Met (*p* = 0.0009), rs4653942 Arg4534His (0.0007), and rs1188710 Gln5891Glu, *p* = 0.0008). The minor allele at each variant was associated with a greater decline in LVEF following therapy. Linkage disequilibrium values show some correlation between these variants (Figure 2). rs56021350 and rs4653942 (MAF = 0.18 and 0.20) are correlated, r^2^ = 0.87, but clearly this signal is independent of rs1188710 (MAF = 0.47), suggesting, if these associations are true, there are at least two common, independent missense variants, each with negative effect on LVEF following doxorubicin and trastuzumab treatment.

**Figure 3:**
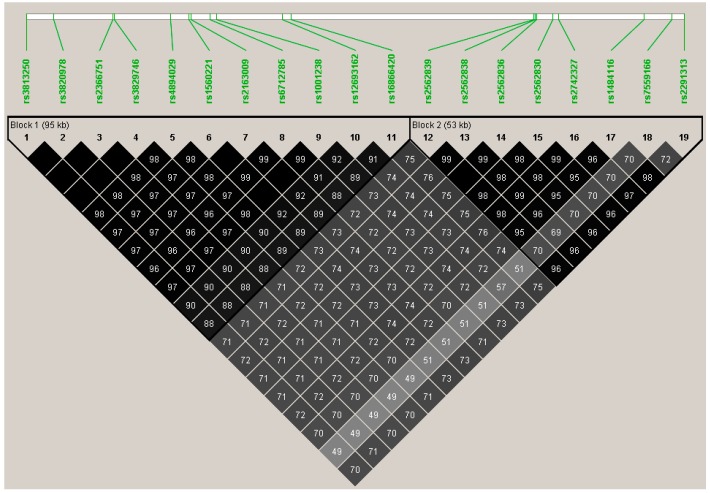
*TTN* variants, *p* < 0.05, linkage disequilibrium plot. Two missense variants were significant at *TTN*, but did not withstand Bonferroni correction for testing 275 variants at this locus: rs3829746/Ile26134Val and rs1001238/Asn17060Asp, (*p* = 0.04, r^2^ = 0.98).

In total, 2/19 associated variants at *TTN* were missense variants, rs3829746 Ile26134 and rs1001238 Ans17060Asp, *p* = 0.04. None remained significant after correction for multiple testing, but linkage disequilibrium analyses showed all variants to be in high linkage disequilibrium (Figure 3), suggesting they are not independent tests. Again, the effect of the minor allele(s) is positive (β ranging 0.79–1.08), suggesting a protective effect against decline of LVEF following therapy. 

### 3.2. Gene-Based Analyses

We next moved to examine rare variant gene-based significance. As these analyses are exploratory, we used both the non-burden sequence kernel association test, SKAT, (which is more powerful when a large fraction of variants in a region are non-causal or the effects of causal variants are in different directions) and the optimal unified burden and non-burden test, SKAT-O. Both tests performed similarly. Seven genes: *ILK*, *TCAP*, *DSC2*, *VCL*, *DSG2*, *FXN*, *DSP*, *KCNQ1* were significant at the nominal *p* < 0.05 level with SKAT-O, all of which were also significant with SKAT, with the exception of *DSG2* (Table 3).

Under the expectation that cardiac-modifying variants could be common with small effects, or both rare and common, we also used the rare/common function in SKAT, weighting rare and common variants equally. Seven genes were significant under the rare/common function, three of which were already identified under rare variant scenarios, *TCAP*, *DSC2* and *VCL*, (*p* = 0.025, 0.008 and 0.026 respectively). The rare/common function of SKAT also identified four additional genes, *NEXN*, *KCNJ2*, *DMD* and *OBSCN* (*p* = 0.044, 0.031, 0.009, 0.019), two of which were not identified in the initial single marker analysis of common variants (*NEXN* and *KCNJ2*).

## 4. Discussion

The genomic architecture of dilated cardiomyopathy is complex, with a high degree of phenotypic variability that could be accounted for by cardiac modifying variants. As an exploratory effort to identify putative modifying variants, we conducted a genetic association study of decline in LVEF following treatment with combination doxorubicin (known to induce cardiomyopathy in animal models and humans) and trastuzumab (a targeted therapy for ERBB2, crucial in prevention of dilated cardiomyopathy in mice [7] and known cardiotoxicity in clinical trials [10,15]) in 800 patients from a breast cancer clinical trial across 72 genes that are causative of cardiomyopathies.

Perhaps the strongest result from these analyses is the association with obscurin (*OBSCN*), a large gene (two giant isoforms, >100 exons, spanning 170 kb). Initially screened as a candidate for hypertrophic cardiomyopathy (HCM), Arimura et al. [22] identified variant, *OBSCN* Arg4344Gln (within Ig48–49 domain) in a 19-year old affected male. Functional analyses demonstrated that the Arg4344Gln variant affected binding of obscurin to the Z9–Z10 domains of Titin [22]. Our own single marker, common variant analyses identified associations with decline in LVEF with two missense variants in this domain: rs56021350/Thr4399Met and rs61825301/His4489Gln. Both variants were present at MAF = 0.18 in 800 patients treated with doxorubicin and trastuzumab, with the minor allele associated with larger decline in LVEF, *p* = 0.001, following treatment. Our study also observed association with rs3795801/Gly4666Ser, MAF = 0.18, *p* = 0.001, again with the minor allele associated with larger decline in LVEF following treatment. This variant maps to the calmodulin binding region (Ig51/52) domain and was also identified in the Arimura study [22] of 144 unrelated HCM patients, but disregarded because it was present in the SNP database. 

*OBSCN* was also recently identified as causative of dilated cardiomyopathy (DCM) [16] based on the observation of five potentially disease-causing mutations in four of 30 patients screened by whole exome sequencing. Marston et al. [16] reported that 15% of the potentially disease-causing variants were in the *OBSCN* gene which the authors likened to the frequency of truncating mutations in *TTN*, that have been proposed as a major causative gene of DCM, suggesting mutations in *OBCSN* may also be significant contributors to DCM burden. Our single marker analyses of common variants and also our gene-based analyses (including 38 common and 44 rare variants) are in agreement, and we further suggest that common and rare variants in *OBSCN* may contribute to DCM burden or perhaps modify disease progression/outcome. 

In a study of 312 DCM patients, *TTN* truncating variants were reported in 25% of familial and 18% of sporadic cases [23]. A subsequent study identified *TTN* truncating variants in 6/17 DCM families [24], not all of which segregated with disease, illustrating the difficulty of determining variant pathogenicity. We had hoped that our exploratory study might shine some light on causality at this locus, but we observed only minimal evidence for the association of common variants, despite the large coding region (>300 exons) and that our analyses included 275 common variants. The association we did observe, appeared to be from variants with a positive value of beta (suggesting lesser decline in LVEF following treatment), all in high linkage disequilibrium, including 17 non-coding and two missense variants, (*p* = 0.019–0.047). If this signal was to be real, the predicted effect on LVEF would be protective against doxorubicin and trastuzumab. 

In summary, our data are suggestive of genetic modifying variants that may increase risk of, or protect against development and/or progression of cardiomyopathy. Several of the associated variants in our study have been previously identified in sequencing studies of familial cardiomyopathy, but likely discarded because they were present in public SNP databases, even at low frequency. All associated common variants (MAF > 0.01) in this study are shown in Table 2. Given the heterogeneity observed within DCM, even within family members carrying the same “causative” variant, a potential strategy would be to ask whether those family members with the worst outcome (earliest onset) were also positive for modifying alleles in the same gene, reported to have negative impact on LVEF.

The limitations of the study are the exploratory nature and testing of multiple genes under multiple scenarios of rare and common variants. Given that several of the associated ‘modifying’ variants are coding, perhaps the next steps are testing in model organisms. This functional testing would also discern whether specific variants are modifiers of the effects of doxorubicin, trastuzumab or combination therapy.

## Figures and Tables

**Table 1:** Genetic variants were tested for association with decline in LVEF in the following genes.

Gene Symbol	Gene Name
*ABCC9*	ATP-binding cassette, sub-family C, member 9
*ACTC1*	Actin, Alpha, Cardiac Muscle 1
*ACTN2*	Actinin Alpha 2
*AKAP9*	A-Kinase Anchoring Protein 9
*ANK2*	Ankyrin 2
*ANKRD1*	Ankyrin Repeat Domain 1
*BAG3*	BCL2 Associated Athanogene 3
*CACNA1C*	Calcium Voltage-Gated Channel Subunit Alpha 1 C
*CAV3*	Caveolin 3
*CRYAB*	Crystallin Alpha B
*CSRP3*	Cysteine And Glycine Rich Protein 3
*DES*	Desmin
*DMD*	Dystrophin
*DSC2*	Desmocollin 2
*DSG2*	Desmoglein 2
*DSP*	Desmoplakin
*ELN*	Elastin
*EMD*	Emerin
*EYA4*	EYA Transcriptional Coactivator And Phosphatase 4
*FXN*	Frataxin
*GATA4*	GATA Binding Protein 4
*GLA*	Galactosidase Alpha
*ILK*	Integrin Linked Kinase
*JAG1*	Jagged 1
*KCNE1*	Potassium Voltage-Gated Channel Subfamily E Regulatory Subunit 1
*KCNE2*	Potassium Voltage-Gated Channel Subfamily E Regulatory Subunit 2
*KCNH2*	Potassium Voltage-Gated Channel Subfamily H Member 2
*KCNJ2*	Potassium Voltage-Gated Channel Subfamily J Member 2
*KCNJ5*	Potassium Voltage-Gated Channel Subfamily J Member 5
*KCNQ1*	Potassium Voltage-Gated Channel Subfamily Q Member 1
*LAMA4*	Laminin Subunit Alpha 4
*LAMP3*	Lysosomal Associated Membrane Protein 3
*LDB3*	LIM Domain Binding 3
*LMNA*	Lamin A/C
*MURC*	Muscle Related Coiled-Coil Protein
*MYBP3*	Myosin binding protein 3
*MYH6*	Myosin Heavy Chain 6
*MYH7*	Myosin Heavy Chain 7
*MYL2*	Myosin Light Chain 2
*MYL3*	Myosin Light Chain 3
*MYLK2*	Myosin Light Chain Kinase 2
*MYOZ2*	Myozenin 2
*MYPN*	Myopalladin
*NEBL*	Nebulette
*NEXN*	Nexilin F-Actin Binding Protein
*NKX2-5*	NK2 Homeobox 5
*NOTCH1*	Notch 1
*NOTCH2*	Notch 2
*OBSCN*	Obscurin
*PDLIM3*	PDZ And LIM Domain 3
*PKP2*	Plakophilin 2
*PLN*	Phospholamban
*PRKAG2*	Protein Kinase AMP-Activated Non-Catalytic Subunit Gamma 2
*PSEN1*	Presenilin 1
*PSEN2*	Presenilin 2
*RAF1*	Raf-1 Proto-Oncogene, Serine/Threonine Kinase
*RBM20*	RNA Binding Motif Protein 20
*RYR2*	Ryanodine Receptor 2
*SCN4B*	Sodium Voltage-Gated Channel Beta Subunit 4
*SCN5A*	Sodium Voltage-Gated Channel Alpha Subunit 5
*SGCD*	Sarcoglycan Delta
*SNTA1*	Syntrophin Alpha 1
*TAZ*	Tafazzin
*TCAP*	Titin-Cap
*TMEM43*	Transmembrane Protein 43
*TMPO*	Thymopoietin
*TNNC1*	Troponin C1, Slow Skeletal And Cardiac Type
*TNNI3*	Troponin I3, Cardiac Type
*TNNT2*	Troponin T2, Cardiac Type
*TPM1*	Tropomyosin 1 (Alpha)
*TTN*	Titin
*VCL*	Vinculin

**Table 2:** Common variants associated with decline in LVEF following treatment with doxorubicin and trastuzumab, *p* < 0.05. Variants associated (*p* < 0.05) with a decline in left ventricular ejection fraction (LVEF), and minor allele frequency (MAF) > 0.01. Analyses were performed for the association of single nucleotide polymorphisms (SNPs) with the maximum decline in LVEF up to two years post-treatment, by linear regression under an additive model, adjusting for baseline LVEF, age and use of anti-hypertensive medication. Variants with effects that increase the maximum decline of LVEF have negative beta values and variants which lessen the maximum decline in LVEF have positive beta values.

Gene	Size (Kb)	Number SNPs	SNP ID	SNP Effect	Beta (95% CI)	MAF	*p*-Value
*VCL*	122	105	rs12250729	intron	0.84 (0.10 to 1.57)	0.25	0.0259
			rs76974852	intron	−3.34 (−5.52 to −1.15)	0.02	0.0029
			rs111748583	downstream	−3.66 (−6.22 to −1.11)	0.02	0.0051
*DMD*	2220	432	rs140820221	intron	−1.94 (−3.37 to −0.52)	0.05	0.0078
			rs1795571	intron	−0.86 (−1.70 to −0.03)	0.13	0.0438
			rs331317	intron	0.85 (0.21 to 1.49)	0.47	0.0090
			rs72626080	intron	1.87 (0.76 to 2.97)	0.09	0.0010
			rs2050074	intron	1.28 (0.34 to 2.22)	0.13	0.0077
			rs2050076	intron	1.32 (0.37 to 2.27)	0.13	0.0065
			rs12559939	intron	1.48 (0.65 to 2.30)	0.19	0.0005
			rs141927233	intron	1.45 (0.63 to 2.28)	0.19	0.0006
			rs73623943	intron	1.18 (0.44 to 1.93)	0.24	0.0020
*OBSCN*	171	55	rs540662	intron	1.05 (0.34 to 1.76)	0.29	0.0038
			rs1188697	Val2720Met	1.02 (0.32 to 1.72)	0.31	0.0045
			rs4653546	intron	−1.20 (−2.03 to −0.38)	0.19	0.0044
			rs56021350	Thr4399Met	−1.42 (−2.26 to −0.59)	0.18	0.0009
			rs61825286	intron	−1.39 (−2.35 to −0.44)	0.14	0.0044
			rs435776	Gly4039Arg	−1.09 (−1.76 to −0.43)	0.50	0.0014
			rs61825301	His4489Gln	−1.41 (−2.25 to −0.58)	0.18	0.0010
			rs4653942	Arg4534His	−1.43 (−2.26 to −0.61)	0.20	0.0007
			rs3795801	Gly4666Ser	−1.39 (−2.23 to −0.56)	0.18	0.0011
			rs287611	intron	−1.10 (−1.76 to −0.43)	0.49	0.0013
			rs1188710	Gln5891Glu	−1.13 (−1.79 to −0.47)	0.47	0.0008
			rs883748	intron	−1.16 (-1.82 to −0.50)	0.47	0.0006
*RYR2*	792	273	rs80107454	intron	−2.89 (−4.59 to −1.19)	0.04	0.0009
			rs2253083	intron	2.10 (0.55 to 3.65)	0.04	0.0081
*TPM1*	29	81	rs5813188	intron	1.42 (0.49 to 2.34)	0.13	0.0027
			rs8026502	intron	1.42 (0.49 to 2.34)	0.13	0.0027
			rs79854225	intron	1.26 (0.28 to 2.24)	0.12	0.0121
			rs57645645	intron	1.42 (0.49 to 2.34)	0.13	0.0027
			rs73431508	intron	1.31 (0.38 to 2.23)	0.14	0.0058
			rs12441488	intron	1.42 (0.49 to 2.34)	0.13	0.0027
*KCNQ1*	404	881	rs80056995	intron	−1.94 (−3.41 to −0.46)	0.05	0.0102
			rs16928363	intron	−1.84 (−3.30 to −0.37)	0.05	0.0142
			rs2237868	intron	−1.84 (−3.30 to −0.37)	0.05	0.0142
			rs74392867	intron	−1.92 (−3.39 to −0.45)	0.05	0.0106
			rs79295543	intron	−1.94 (−3.41 to −0.46)	0.05	0.0102
			rs77059665	intron	−2.29 (−3.90 to −0.69)	0.04	0.0051
			rs28730663	intron	−2.71 (−4.62 to −0.80)	0.03	0.0056
			rs35237966	intron	−0.94 (−1.61 to −0.26)	0.39	0.0065
			rs72844252	intron	−1.57 (−2.71 to −0.42)	0.09	0.0074
			rs231880	intron	−0.70 (−1.39 to −0.01)	0.35	0.0466
			rs71476688	intron	2.59 (0.87 to 4.32)	0.03	0.0033
			rs34861825	intron	1.38 (0.40 to 2.36)	0.16	0.0061
			rs12419030	intron	0.89 (0.17 to 1.61)	0.31	0.0160
			rs12419347	intron	0.91 (0.19 to 1.63)	0.31	0.0139
*JAG1*	36	18	rs3748480	intron	1.52 (0.51 to 2.53)	0.12	0.0033
*SGCD*	1060	1851	rs6860238	intron	2.24 (0.71 to 3.78)	0.05	0.0043
*SCN5A*	98	243	rs11129796	intron	−1.44 (−2.67 to −0.20)	0.09	0.0228
			rs9832586	intron	−1.28 (−2.35 to −0.21)	0.10	0.0190
			rs7430407	synonymous	−1.43 (−2.44 to −0.41)	0.11	0.0060
			rs6790619	intron	−1.44 (−2.70 to −0.17)	0.07	0.0264
			rs7645173	intron	−1.52 (−2.81 to −0.24)	0.07	0.0206
			rs9311190	intron	−1.56 (−2.85 to −0.27)	0.07	0.0180
			rs11711097	intron	−0.85 (−1.64 to −0.07)	0.22	0.0340
			rs7432532	intron	−1.45 (−2.47 to −0.43)	0.11	0.0053
			rs7426433	intron	−1.50 (−2.52 to −0.48)	0.11	0.0040
			rs7433889	intron	−1.13 (−2.10 to −0.17)	0.13	0.0213
			rs6599214	intron	−1.21 (−2.17 to −0.25)	0.13	0.0142
			rs6599215	intron	−1.27 (−2.23 to −0.31)	0.13	0.0099
			rs6599216	intron	−1.21 (−2.17 to −0.26)	0.13	0.0126
			rs6599217	intron	−1.20 (−2.15 to −0.25)	0.13	0.0142
			rs6599218	intron	−1.20 (−2.15 to −0.24)	0.13	0.0142
			rs7613045	intron	−1.36 (−2.33 to −0.40)	0.12	0.0059
			rs63200660	intron	−1.19 (−2.14 to −0.25)	0.13	0.0137
			rs6599221	intron	−1.30 (−2.59 to −0.01)	0.07	0.0478
			rs7627488	downstream	−2.99 (−5.55 to −0.43)	0.02	0.0224
			rs7636280	downstream	−2.96 (−5.68 to −0.23)	0.01	0.0337
*RBM20*	195	57	rs7069694	intron	−0.79 (−1.45 to −0.13)	0.41	0.0198
			rs2181407	intron	−0.76 (−1.47 to −0.06)	0.28	0.0342
			rs17831429	intron	−2.68 (−4.58 to −0.78)	0.03	0.0058
*SCN4B*	20	8	rs955917	intron	1.07 (0.22 to 1.93)	0.17	0.0140
*TTN*	281	275	rs3813250	synonymous	0.85 (0.06 to 1.63)	0.22	0.0349
			rs3820978	downstream	0.79 (0.01 to 1.58)	0.22	0.0486
			rs2366751	synonymous	0.81 (0.03 to 1.60)	0.22	0.0432
			rs3829746	Ile26134Val	0.81 (0.03 to 1.60)	0.22	0.0432
			rs4894029	synonymous	0.81 (0.02 to 1.59)	0.22	0.0453
			rs1560221	synonymous	0.82 (0.04 to 1.60)	0.23	0.0406
			rs2163009	synonymous	0.82 (0.04 to 1.61)	0.23	0.0399
			rs6712785	intron	0.82 (0.03 to 1.60)	0.22	0.0421
			rs1001238	Asn17060Asp	0.82 (0.04 to 1.61)	0.23	0.0399
			rs12693162	downstream	0.86 (0.06 to 1.65)	0.21	0.0344
			rs16866420	intron	0.82 (0.02 to 1.60)	0.21	0.0437
			rs2562839	synonymous	0.83 (0.01 to 1.64)	0.20	0.0467
			rs2562838	synonymous	0.83 (0.02 to 1.64)	0.20	0.0449
			rs2562836	synonymous	0.83 (0.01 to 1.64)	0.20	0.0467
			rs2562830	intron	0.82 (0.01 to 1.63)	0.20	0.0472
			rs2742327	intron	0.82 (0.02 to 1.63)	0.20	0.0460
			rs1484116	intron	0.88 (0.08 to 1.68)	0.20	0.0314
			rs7559166	intron	1.08 (0.19 to 1.98)	0.15	0.0191
			rs2291313	intron	0.86 (0.06 to 1.67)	0.20	0.0366
*CACNA1C*	645	216	rs1009281	intron	−0.76 (−1.41 to −0.12)	0.48	0.0204
			rs11832738	intron	−0.81 (−1.49 to −0.12)	0.30	0.0215

**Table 3:** Rare and common variant gene-based analyses of decline in LVEF following treatment with doxorubicin and trastuzumab, *p* < 0.05. Gene-based analyses were performed with SKAT, SKAT-O and SKAT CR (common/rare) functions. N.Marker All represents the total number of variants. N.Marker Test represents the number of variants used in the gene-based analyses (monomorphic variants are excluded). For the SKAT common/rare function, N.Marker.Rare is the number of analyzed variants with MAF < 0.025 and for N.Marker.Common, the number of analyzed variants with MAF < 0.025.

Gene	N.Marker All	N.Marker Test	SKAT *p*-Value	SKAT-O *p*-Value	SKAT CR *p*-Value	SKAT CR N.Marker.Rare	SKAT CR N.Marker.Common
*ILK*	40	31	0.032	0.011	0.070	2	29
*TCAP*	9	2	0.011	0.011	0.025	1	1
*DSC2*	72	12	0.006	0.012	0.008	6	6
*VCL*	403	195	0.014	0.018	0.026	99	96
*DSG2*	118	25	0.454	0.028	0.395	12	13
*FXN*	16	11	0.042	0.038	0.202	0	11
*DSP*	208	43	0.021	0.042	0.097	27	16
*KCNQ1*	1795	1063	0.024	0.044	0.153	220	843
*NEXN*	9	8	0.065	0.110	0.044	4	4
*KCNJ2*	49	2	0.159	0.222	0.031	1	1
*DMD*	460	443	0.267	0.444	0.009	16	427
*OBSCN*	136	82	0.428	0.632	0.019	38	44

## Supplementary Materials

The following are available online, Table S1: 72 known cardiomyopathy genes and SNPs used in common and rare variant analyses. Figure S1: N9831 PCA plot, Arms A, B and C GWAS data.
