# Supplementary material for: Breast Cancer Clinical Trial of Chemotherapy and Trastuzumab: Potential Tool to Identify Cardiac Modifying Variants of Dilated Cardiomyopathy
Source: J Cardiovasc Dev Dis. 2017 May 4;4(2):6. doi: 10.3390/jcdd4020006 (PMC5715703; doi:10.3390/jcdd4020006)
Supplement: Supplementary file 1 [file jcdd-04-00006-s001.zip › Supplementary Figure 1.pdf]

Supplementary Figure 1: N9831 PCA plot, Arms A, B and C GWAS data.

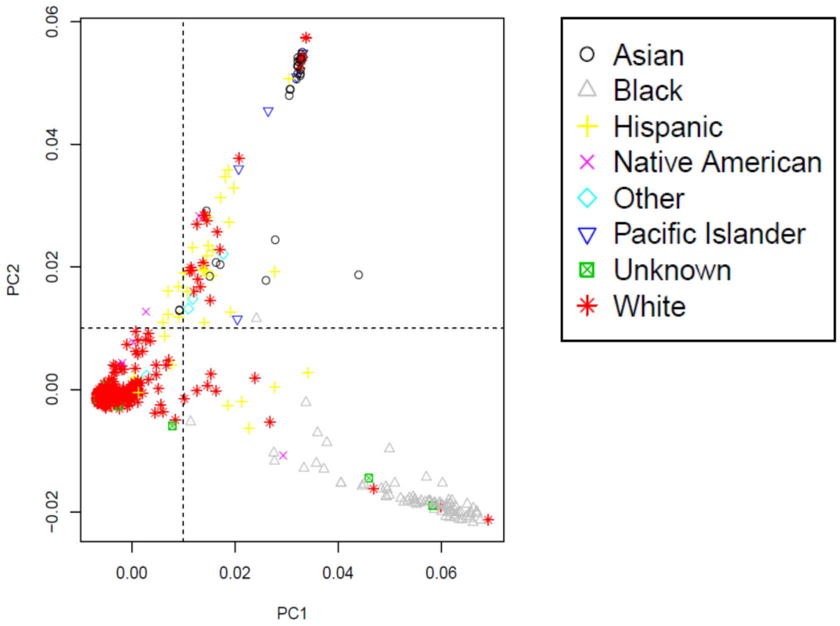

Supplementary Figure 1 Legend: The first and second principal components separate patients based on race. Self-reported non-Caucasians were excluded, as were Caucasians outside of their expected cluster (lower left quadrant).
