# Supplementary material for: Breast Cancer Clinical Trial of Chemotherapy and Trastuzumab: Potential Tool to Identify Cardiac Modifying Variants of Dilated Cardiomyopathy
Source: J Cardiovasc Dev Dis. 2017 May 4;4(2):6. doi: 10.3390/jcdd4020006 (PMC5715703; doi:10.3390/jcdd4020006)
Supplement: Supplementary file 1 [file jcdd-04-00006-s001.zip › Supplementary Table 1.pdf]

gene	dbSNP.RS.ID	hg19 coordinates	SNP
ABCC9	rs17630922	chr12:21,950,324-22,094,336	AX-11332151
ABCC9	---	chr12:21,950,324-22,094,336	AX-90043439
ABCC9	rs829060	chr12:21,950,324-22,094,336	AX-16901287
ABCC9	rs10841895	chr12:21,950,324-22,094,336	AX-122744505
ABCC9	rs10841895	chr12:21,950,324-22,094,336	AX-148499213
ABCC9	rs829061	chr12:21,950,324-22,094,336	AX-148316894
ABCC9	rs829061	chr12:21,950,324-22,094,336	AX-156270652
ABCC9	rs7966926	chr12:21,950,324-22,094,336	AX-30632217
ABCC9	rs113591229	chr12:21,950,324-22,094,336	AX-148920908
ABCC9	rs113591229	chr12:21,950,324-22,094,336	AX-156283775
ABCC9	rs7956490	chr12:21,950,324-22,094,336	AX-39362191
ABCC9	rs4148679	chr12:21,950,324-22,094,336	AX-16901305
ABCC9	rs540590379	chr12:21,950,324-22,094,336	AX-156285425
ABCC9	rs113117014	chr12:21,950,324-22,094,336	AX-148784013
ABCC9	rs113117014	chr12:21,950,324-22,094,336	AX-156270653
ABCC9	rs9668454	chr12:21,950,324-22,094,336	AX-12666550
ABCC9	rs2638441	chr12:21,950,324-22,094,336	AX-16901313
ABCC9	rs199804188	chr12:21,950,324-22,094,336	AX-156285841
ABCC9	rs28459490	chr12:21,950,324-22,094,336	AX-148732246
ABCC9	rs7309274	chr12:21,950,324-22,094,336	AX-16901320
ABCC9	rs4148678	chr12:21,950,324-22,094,336	AX-113409058
ABCC9	rs4148678	chr12:21,950,324-22,094,336	AX-113965543
ABCC9	rs16924332	chr12:21,950,324-22,094,336	AX-11290180
ABCC9	rs7136997	chr12:21,950,324-22,094,336	AX-113006537
ABCC9	rs7136997	chr12:21,950,324-22,094,336	AX-113415296
ABCC9	rs2726120	chr12:21,950,324-22,094,336	AX-16901336
ABCC9	rs111696716	chr12:21,950,324-22,094,336	AX-119373074
ABCC9	rs111696716	chr12:21,950,324-22,094,336	AX-151334701
ABCC9	rs4148677	chr12:21,950,324-22,094,336	AX-11496310
ABCC9	rs829074	chr12:21,950,324-22,094,336	AX-16901341
ABCC9	rs56178300	chr12:21,950,324-22,094,336	AX-123090341
ABCC9	rs829075	chr12:21,950,324-22,094,336	AX-148924082
ABCC9	rs829075	chr12:21,950,324-22,094,336	AX-156266224
ABCC9	rs2161062	chr12:21,950,324-22,094,336	AX-156266225
ABCC9	rs1873638	chr12:21,950,324-22,094,336	AX-16901344
ABCC9	rs73070690	chr12:21,950,324-22,094,336	AX-30632299
ABCC9	rs17631144	chr12:21,950,324-22,094,336	AX-11332166
ABCC9	rs1283809	chr12:21,950,324-22,094,336	AX-11228808
ABCC9	rs1283810	chr12:21,950,324-22,094,336	AX-12447798
ABCC9	rs1283811	chr12:21,950,324-22,094,336	AX-11228809
ABCC9	rs146942382	chr12:21,950,324-22,094,336	AX-83012390
ABCC9	rs1283812	chr12:21,950,324-22,094,336	AX-88317235
ABCC9	rs61928479	chr12:21,950,324-22,094,336	AX-121177167
ABCC9	rs11046202	chr12:21,950,324-22,094,336	AX-11139910
ABCC9	rs2112080	chr12:21,950,324-22,094,336	AX-16901354
ABCC9	rs1283815	chr12:21,950,324-22,094,336	AX-119467012
ABCC9	rs1283815	chr12:21,950,324-22,094,336	AX-123026069
ABCC9	rs1283816	chr12:21,950,324-22,094,336	AX-12447801
ABCC9	rs1283817	chr12:21,950,324-22,094,336	AX-16901361
ABCC9	rs3782667	chr12:21,950,324-22,094,336	AX-16901364
ABCC9	rs11046203	chr12:21,950,324-22,094,336	AX-39362239
ABCC9	rs1967534	chr12:21,950,324-22,094,336	AX-11356552
ABCC9	rs1517284	chr12:21,950,324-22,094,336	AX-16901377
ABCC9	rs7974190	chr12:21,950,324-22,094,336	AX-16901380

ABCC9	rs829068	chr12:21,950,324-22,094,336	AX-16901381
ABCC9	rs829069	chr12:21,950,324-22,094,336	AX-16901383
ABCC9	rs2098574	chr12:21,950,324-22,094,336	AX-11367169
ABCC9	rs864360	chr12:21,950,324-22,094,336	AX-16901386
ABCC9	rs1299731	chr12:21,950,324-22,094,336	AX-112926248
ABCC9	rs34811342	chr12:21,950,324-22,094,336	AX-151118150
ABCC9	rs704175	chr12:21,950,324-22,094,336	AX-11603324
ABCC9	rs12825295	chr12:21,950,324-22,094,336	AX-148260452
ABCC9	rs12825295	chr12:21,950,324-22,094,336	AX-156270654
ABCC9	rs704176	chr12:21,950,324-22,094,336	AX-39362253
ABCC9	rs12369421	chr12:21,950,324-22,094,336	AX-16901406
ABCC9	rs11046205	chr12:21,950,324-22,094,336	AX-96077695
ABCC9	rs11046205	chr12:21,950,324-22,094,336	AX-96097798
ABCC9	rs67543549	chr12:21,950,324-22,094,336	AX-30632441
ABCC9	rs704177	chr12:21,950,324-22,094,336	AX-16901417
ABCC9	rs704178	chr12:21,950,324-22,094,336	AX-16901418
ABCC9	rs704179	chr12:21,950,324-22,094,336	AX-11603328
ABCC9	rs704180	chr12:21,950,324-22,094,336	AX-39362259
ABCC9	rs829072	chr12:21,950,324-22,094,336	AX-156283776
ABCC9	rs861202	chr12:21,950,324-22,094,336	AX-156289418
ABCC9	rs1643235	chr12:21,950,324-22,094,336	AX-16901428
ABCC9	rs11836934	chr12:21,950,324-22,094,336	AX-50783510
ABCC9	rs55849264	chr12:21,950,324-22,094,336	AX-16901441
ABCC9	---	chr12:21,950,324-22,094,336	AX-151121197
ABCC9	rs829079	chr12:21,950,324-22,094,336	AX-11668469
ABCC9	rs829080	chr12:21,950,324-22,094,336	AX-11668470
ABCC9	rs138878517	chr12:21,950,324-22,094,336	AX-122466762
ABCC9	rs143553602	chr12:21,950,324-22,094,336	AX-156285692
ABCC9	rs1283822	chr12:21,950,324-22,094,336	AX-16901460
ABCC9	rs704182	chr12:21,950,324-22,094,336	AX-113000328
ABCC9	rs704182	chr12:21,950,324-22,094,336	AX-113409062
ABCC9	rs4148674	chr12:21,950,324-22,094,336	AX-16901465
ABCC9	rs4148673	chr12:21,950,324-22,094,336	AX-11496308
ABCC9	rs7966849	chr12:21,950,324-22,094,336	AX-16901470
ABCC9	rs7966768	chr12:21,950,324-22,094,336	AX-11656727
ABCC9	rs4148672	chr12:21,950,324-22,094,336	AX-107753769
ABCC9	rs4148672	chr12:21,950,324-22,094,336	AX-156289419
ABCC9	rs2307024	chr12:21,950,324-22,094,336	AX-16901472
ABCC9	---	chr12:21,950,324-22,094,336	AX-86727263
ABCC9	rs4296081	chr12:21,950,324-22,094,336	AX-156270656
ABCC9	rs11046212	chr12:21,950,324-22,094,336	AX-112926250
ABCC9	rs11046212	chr12:21,950,324-22,094,336	AX-113482901
ABCC9	rs704185	chr12:21,950,324-22,094,336	AX-16901473
ABCC9	---	chr12:21,950,324-22,094,336	AX-39362331
ABCC9	rs11046213	chr12:21,950,324-22,094,336	AX-16901476
ABCC9	rs75649931	chr12:21,950,324-22,094,336	AX-16901477
ABCC9	rs2161063	chr12:21,950,324-22,094,336	AX-16901479
ABCC9	rs76857194	chr12:21,950,324-22,094,336	AX-30632595
ABCC9	rs73254540	chr12:21,950,324-22,094,336	AX-16901484
ABCC9	rs11046215	chr12:21,950,324-22,094,336	AX-12405181
ABCC9	rs10841900	chr12:21,950,324-22,094,336	AX-16901489
ABCC9	rs704187	chr12:21,950,324-22,094,336	AX-16901492
ABCC9	rs704189	chr12:21,950,324-22,094,336	AX-11603336
ABCC9	rs12230539	chr12:21,950,324-22,094,336	AX-112926379
ABCC9	rs12230539	chr12:21,950,324-22,094,336	AX-113000417

ABCC9	rs11835804	chr12:21,950,324-22,094,336	AX-113417350
ABCC9	rs11835804	chr12:21,950,324-22,094,336	AX-113491400
ABCC9	rs1972613	chr12:21,950,324-22,094,336	AX-115138575
ABCC9	rs1972613	chr12:21,950,324-22,094,336	AX-148187130
ABCC9	rs704190	chr12:21,950,324-22,094,336	AX-16901499
ABCC9	rs704191	chr12:21,950,324-22,094,336	AX-16901503
ABCC9	rs704192	chr12:21,950,324-22,094,336	AX-16901504
ABCC9	rs137881774	chr12:21,950,324-22,094,336	AX-30632637
ABCC9	rs704193	chr12:21,950,324-22,094,336	AX-11603341
ABCC9	rs141281214	chr12:21,950,324-22,094,336	AX-86597744
ABCC9	rs704194	chr12:21,950,324-22,094,336	AX-112926251
ABCC9	rs704194	chr12:21,950,324-22,094,336	AX-113965545
ABCC9	rs697252	chr12:21,950,324-22,094,336	AX-12616117
ABCC9	---	chr12:21,950,324-22,094,336	AX-151121898
ABCC9	---	chr12:21,950,324-22,094,336	AX-156286326
ABCC9	rs71444142	chr12:21,950,324-22,094,336	AX-107728867
ABCC9	rs71444142	chr12:21,950,324-22,094,336	AX-156298635
ABCC9	rs3782668	chr12:21,950,324-22,094,336	AX-16901532
ABCC9	rs1283798	chr12:21,950,324-22,094,336	AX-16901535
ABCC9	rs1965033	chr12:21,950,324-22,094,336	AX-148242915
ABCC9	rs1965033	chr12:21,950,324-22,094,336	AX-156270657
ABCC9	rs7958833	chr12:21,950,324-22,094,336	AX-121101825
ABCC9	rs7958833	chr12:21,950,324-22,094,336	AX-156279932
ABCC9	rs10841902	chr12:21,950,324-22,094,336	AX-16901541
ABCC9	rs71053351	chr12:21,950,324-22,094,336	AX-151213342
ABCC9	rs4148669	chr12:21,950,324-22,094,336	AX-16901542
ABCC9	rs1492137	chr12:21,950,324-22,094,336	AX-11268601
ABCC9	rs10841904	chr12:21,950,324-22,094,336	AX-113925008
ABCC9	rs74503836	chr12:21,950,324-22,094,336	AX-30632721
ABCC9	rs10841905	chr12:21,950,324-22,094,336	AX-113442455
ABCC9	rs10841905	chr12:21,950,324-22,094,336	AX-113516673
ABCC9	rs10841906	chr12:21,950,324-22,094,336	AX-113033946
ABCC9	rs10841906	chr12:21,950,324-22,094,336	AX-113442460
ABCC9	rs139991988	chr12:21,950,324-22,094,336	AX-120579410
ABCC9	rs148174226	chr12:21,950,324-22,094,336	AX-83250493
ABCC9	rs704205	chr12:21,950,324-22,094,336	AX-11603351
ABCC9	---	chr12:21,950,324-22,094,336	AX-16901563
ABCC9	rs4148668	chr12:21,950,324-22,094,336	AX-16901567
ABCC9	rs4148667	chr12:21,950,324-22,094,336	AX-39362443
ABCC9	rs4148666	chr12:21,950,324-22,094,336	AX-11496307
ABCC9	rs35711602	chr12:21,950,324-22,094,336	AX-16901572
ABCC9	rs12304313	chr12:21,950,324-22,094,336	AX-12432886
ABCC9	rs11836595	chr12:21,950,324-22,094,336	AX-112931159
ABCC9	rs11836595	chr12:21,950,324-22,094,336	AX-113487886
ABCC9	rs74067832	chr12:21,950,324-22,094,336	AX-16901573
ABCC9	rs74067833	chr12:21,950,324-22,094,336	AX-16901574
ABCC9	rs77923631	chr12:21,950,324-22,094,336	AX-16901583
ABCC9	rs77360959	chr12:21,950,324-22,094,336	AX-16901584
ABCC9	rs7301876	chr12:21,950,324-22,094,336	AX-113409064
ABCC9	rs7301876	chr12:21,950,324-22,094,336	AX-113965547
ABCC9	rs1356369	chr12:21,950,324-22,094,336	AX-12459438
ABCC9	rs79757822	chr12:21,950,324-22,094,336	AX-16901585
ABCC9	rs4148665	chr12:21,950,324-22,094,336	AX-16901589
ABCC9	rs4148664	chr12:21,950,324-22,094,336	AX-11496305
ABCC9	rs1356368	chr12:21,950,324-22,094,336	AX-39362475

ABCC9	rs1283802	chr12:21,950,324-22,094,336	AX-11228807
ABCC9	rs1388698	chr12:21,950,324-22,094,336	AX-113409065
ABCC9	rs1283808	chr12:21,950,324-22,094,336	AX-112959838
ABCC9	rs1283808	chr12:21,950,324-22,094,336	AX-92777905
ABCC9	rs77026371	chr12:21,950,324-22,094,336	AX-148700748
ABCC9	rs77026371	chr12:21,950,324-22,094,336	AX-156270658
ABCC9	rs7314764	chr12:21,950,324-22,094,336	AX-16901596
ABCC9	rs704206	chr12:21,950,324-22,094,336	AX-16901598
ABCC9	rs7137730	chr12:21,950,324-22,094,336	AX-16901599
ABCC9	rs7308718	chr12:21,950,324-22,094,336	AX-16901601
ABCC9	rs7138546	chr12:21,950,324-22,094,336	AX-16901602
ABCC9	rs1352909	chr12:21,950,324-22,094,336	AX-113891689
ABCC9	rs1352909	chr12:21,950,324-22,094,336	AX-113965549
ABCC9	rs5796929	chr12:21,950,324-22,094,336	AX-151269049
ABCC9	rs150036969	chr12:21,950,324-22,094,336	AX-83242746
ABCC9	rs61001398	chr12:21,950,324-22,094,336	AX-16901608
ABCC9	rs201087758	chr12:21,950,324-22,094,336	AX-156286034
ABCC9	rs704208	chr12:21,950,324-22,094,336	AX-16901614
ABCC9	rs71444143	chr12:21,950,324-22,094,336	AX-107769538
ABCC9	rs71444143	chr12:21,950,324-22,094,336	AX-156298481
ABCC9	rs4762717	chr12:21,950,324-22,094,336	AX-16901619
ABCC9	rs704209	chr12:21,950,324-22,094,336	AX-16901620
ABCC9	rs2131136	chr12:21,950,324-22,094,336	AX-12518246
ABCC9	rs1283807	chr12:21,950,324-22,094,336	AX-12447797
ABCC9	rs704210	chr12:21,950,324-22,094,336	AX-113094257
ABCC9	rs704210	chr12:21,950,324-22,094,336	AX-123026070
ABCC9	rs704211	chr12:21,950,324-22,094,336	AX-156270659
ABCC9	---	chr12:21,950,324-22,094,336	AX-122289052
ABCC9	---	chr12:21,950,324-22,094,336	AX-148725230
ABCC9	rs2032775	chr12:21,950,324-22,094,336	AX-12514555
ABCC9	rs704212	chr12:21,950,324-22,094,336	AX-12618816
ABCC9	rs4148661	chr12:21,950,324-22,094,336	AX-16901628
ABCC9	rs2292772	chr12:21,950,324-22,094,336	AX-16901631
ABCC9	rs2292771	chr12:21,950,324-22,094,336	AX-16901632
ABCC9	rs1914361	chr12:21,950,324-22,094,336	AX-16901633
ABCC9	rs704215	chr12:21,950,324-22,094,336	AX-16901634
ABCC9	rs704217	chr12:21,950,324-22,094,336	AX-16901637
ABCC9	rs7978092	chr12:21,950,324-22,094,336	AX-11657531
ABCC9	rs74067850	chr12:21,950,324-22,094,336	AX-16901644
ABCC9	rs72211208	chr12:21,950,324-22,094,336	AX-156284770
ABCC9	rs72211208	chr12:21,950,324-22,094,336	AX-97256391
ABCC9	rs113142980	chr12:21,950,324-22,094,336	AX-148911748
ABCC9	rs113142980	chr12:21,950,324-22,094,336	AX-156270660
ABCC9	rs59061211	chr12:21,950,324-22,094,336	AX-156270661
ABCC9	rs1283806	chr12:21,950,324-22,094,336	AX-113403892
ABCC9	rs1283806	chr12:21,950,324-22,094,336	AX-113477785
ABCC9	rs1283805	chr12:21,950,324-22,094,336	AX-113403893
ABCC9	rs1283805	chr12:21,950,324-22,094,336	AX-113960497
ABCC9	rs1283804	chr12:21,950,324-22,094,336	AX-113886627
ABCC9	rs1283804	chr12:21,950,324-22,094,336	AX-113960498
ABCC9	rs10743426	chr12:21,950,324-22,094,336	AX-113409069
ABCC9	rs10743426	chr12:21,950,324-22,094,336	AX-113965552
ABCC9	rs10841907	chr12:21,950,324-22,094,336	AX-95859985
ABCC9	rs34243384	chr12:21,950,324-22,094,336	AX-16901662
ABCC9	rs11046224	chr12:21,950,324-22,094,336	AX-113068328

ABCC9	rs11046224	chr12:21,950,324-22,094,336	AX-148204580
ABCC9	rs148096888	chr12:21,950,324-22,094,336	AX-148902887
ABCC9	rs148096888	chr12:21,950,324-22,094,336	AX-156283777
ABCC9	rs10770862	chr12:21,950,324-22,094,336	AX-112998313
ABCC9	rs10770862	chr12:21,950,324-22,094,336	AX-113889747
ABCC9	rs138809747	chr12:21,950,324-22,094,336	AX-148902888
ABCC9	rs138809747	chr12:21,950,324-22,094,336	AX-156297638
ABCC9	rs146375843	chr12:21,950,324-22,094,336	AX-151337532
ABCC9	rs146375843	chr12:21,950,324-22,094,336	AX-151460409
ABCC9	rs61921496	chr12:21,950,324-22,094,336	AX-148205342
ABCC9	rs61921496	chr12:21,950,324-22,094,336	AX-156283778
ABCC9	rs11046226	chr12:21,950,324-22,094,336	AX-151188210
ABCC9	rs11046226	chr12:21,950,324-22,094,336	AX-151250351
ABCC9	rs11046227	chr12:21,950,324-22,094,336	AX-105135049
ABCC9	rs11046227	chr12:21,950,324-22,094,336	AX-148203408
ABCC9	rs140731339	chr12:21,950,324-22,094,336	AX-121668767
ABCC9	rs140731339	chr12:21,950,324-22,094,336	AX-151189940
ABCC9	rs10770863	chr12:21,950,324-22,094,336	AX-112959849
ABCC9	rs10770863	chr12:21,950,324-22,094,336	AX-113999019
ABCC9	rs149126386	chr12:21,950,324-22,094,336	AX-148904725
ABCC9	rs149126386	chr12:21,950,324-22,094,336	AX-156283779
ABCC9	rs11046228	chr12:21,950,324-22,094,336	AX-112998314
ABCC9	rs11046228	chr12:21,950,324-22,094,336	AX-113889748
ABCC9	rs10841908	chr12:21,950,324-22,094,336	AX-105168000
ABCC9	rs143789188	chr12:21,950,324-22,094,336	AX-148587974
ABCC9	rs143789188	chr12:21,950,324-22,094,336	AX-156283780
ABCC9	rs189137911	chr12:21,950,324-22,094,336	AX-112076761
ABCC9	rs189137911	chr12:21,950,324-22,094,336	AX-148559092
ABCC9	rs10841910	chr12:21,950,324-22,094,336	AX-113889749
ABCC9	rs10841910	chr12:21,950,324-22,094,336	AX-97567512
ABCC9	rs2193881	chr12:21,950,324-22,094,336	AX-114345411
ABCC9	rs2193881	chr12:21,950,324-22,094,336	AX-156289422
ABCC9	rs11046229	chr12:21,950,324-22,094,336	AX-120130312
ABCC9	rs11046229	chr12:21,950,324-22,094,336	AX-148216452
ABCC9	rs7972391	chr12:21,950,324-22,094,336	AX-148118548
ABCC9	rs7972391	chr12:21,950,324-22,094,336	AX-149014133
ABCC9	rs140413681	chr12:21,950,324-22,094,336	AX-148068669
ABCC9	rs140413681	chr12:21,950,324-22,094,336	AX-156283781
ABCC9	rs4148660	chr12:21,950,324-22,094,336	AX-16901678
ABCC9	rs11046230	chr12:21,950,324-22,094,336	AX-16901679
ABCC9	rs1421602	chr12:21,950,324-22,094,336	AX-16901682
ABCC9	rs75280226	chr12:21,950,324-22,094,336	AX-16901683
ABCC9	rs4148657	chr12:21,950,324-22,094,336	AX-16901686
ABCC9	rs139620148	chr12:21,950,324-22,094,336	AX-86575108
ABCC9	rs10770865	chr12:21,950,324-22,094,336	AX-16901688
ABCC9	rs4148656	chr12:21,950,324-22,094,336	AX-113409070
ABCC9	rs4148656	chr12:21,950,324-22,094,336	AX-113965553
ABCC9	rs4148655	chr12:21,950,324-22,094,336	AX-113965554
ABCC9	rs72559752	chr12:21,950,324-22,094,336	AX-16901692
ABCC9	rs4148654	chr12:21,950,324-22,094,336	AX-16901693
ABCC9	rs2418021	chr12:21,950,324-22,094,336	AX-11390821
ABCC9	rs12366938	chr12:21,950,324-22,094,336	AX-16901695
ABCC9	---	chr12:21,950,324-22,094,336	AX-90043125
ABCC9	rs1517276	chr12:21,950,324-22,094,336	AX-16901701
ABCC9	rs1344569	chr12:21,950,324-22,094,336	AX-16901705

ABCC9	rs4148651	chr12:21,950,324-22,094,336	AX-147868449
ABCC9	rs4148651	chr12:21,950,324-22,094,336	AX-147963730
ABCC9	rs139975827	chr12:21,950,324-22,094,336	AX-120994491
ABCC9	---	chr12:21,950,324-22,094,336	AX-120836624
ABCC9	rs12821892	chr12:21,950,324-22,094,336	AX-119730425
ABCC9	rs12821892	chr12:21,950,324-22,094,336	AX-120771628
ABCC9	rs181534345	chr12:21,950,324-22,094,336	AX-96639807
ABCC9	rs181534345	chr12:21,950,324-22,094,336	AX-96934187
ABCC9	rs185890370	chr12:21,950,324-22,094,336	AX-105027452
ABCC9	rs185890370	chr12:21,950,324-22,094,336	AX-105051134
ABCC9	rs58386780	chr12:21,950,324-22,094,336	AX-16901709
ABCC9	rs3759236	chr12:21,950,324-22,094,336	AX-16901710
ABCC9	rs4148650	chr12:21,950,324-22,094,336	AX-112926257
ABCC9	rs4148650	chr12:21,950,324-22,094,336	AX-113482908
ABCC9	---	chr12:21,950,324-22,094,336	AX-90027609
ABCC9	rs4148649	chr12:21,950,324-22,094,336	AX-12567567
ABCC9	rs34413975	chr12:21,950,324-22,094,336	AX-12552403
ABCC9	rs10770867	chr12:21,950,324-22,094,336	AX-16901716
ABCC9	rs11046234	chr12:21,950,324-22,094,336	AX-11139914
ABCC9	rs7136887	chr12:21,950,324-22,094,336	AX-113891693
ABCC9	rs7137250	chr12:21,950,324-22,094,336	AX-16901718
ABCC9	rs79470178	chr12:21,950,324-22,094,336	AX-16901720
ABCC9	rs11046235	chr12:21,950,324-22,094,336	AX-156270662
ABCC9	rs7300572	chr12:21,950,324-22,094,336	AX-112917884
ABCC9	rs7300572	chr12:21,950,324-22,094,336	AX-113957314
ABCC9	rs10770870	chr12:21,950,324-22,094,336	AX-113883421
ABCC9	rs10770870	chr12:21,950,324-22,094,336	AX-113957315
ABCC9	rs10770871	chr12:21,950,324-22,094,336	AX-16901722
ABCC9	rs113615977	chr12:21,950,324-22,094,336	AX-16901723
ABCC9	rs74693025	chr12:21,950,324-22,094,336	AX-30633043
ABCC9	rs76728072	chr12:21,950,324-22,094,336	AX-61431845
ABCC9	rs2900493	chr12:21,950,324-22,094,336	AX-112994720
ABCC9	rs113677041	chr12:21,950,324-22,094,336	AX-16901725
ABCC9	rs2277404	chr12:21,950,324-22,094,336	AX-11380820
ABCC9	rs2176394	chr12:21,950,324-22,094,336	AX-112994721
ABCC9	rs2277405	chr12:21,950,324-22,094,336	AX-113477300
ABCC9	rs870135	chr12:21,950,324-22,094,336	AX-16901727
ABCC9	rs870134	chr12:21,950,324-22,094,336	AX-39362633
ABCC9	rs1356370	chr12:21,950,324-22,094,336	AX-39362635
ABCC9	rs74067884	chr12:21,950,324-22,094,336	AX-50119962
ABCC9	rs200851499	chr12:21,950,324-22,094,336	AX-151434925
ABCC9	rs200851499	chr12:21,950,324-22,094,336	AX-156284968
ABCC9	rs7308081	chr12:21,950,324-22,094,336	AX-16901730
ABCC9	rs2138723	chr12:21,950,324-22,094,336	AX-16901733
ABCC9	rs7977434	chr12:21,950,324-22,094,336	AX-113561219
ABCC9	rs7977434	chr12:21,950,324-22,094,336	AX-156270663
ABCC9	rs12371738	chr12:21,950,324-22,094,336	AX-156270664
ABCC9	rs4762722	chr12:21,950,324-22,094,336	AX-39362687
ABCC9	rs74067890	chr12:21,950,324-22,094,336	AX-156279933
ABCC9	rs74067890	chr12:21,950,324-22,094,336	AX-156295491
ABCC9	rs4586220	chr12:21,950,324-22,094,336	AX-12575284
ABCC9	rs35677639	chr12:21,950,324-22,094,336	AX-16901757
ABCC9	rs7316271	chr12:21,950,324-22,094,336	AX-11620839
ABCC9	rs12315609	chr12:21,950,324-22,094,336	AX-39362709
ABCC9	rs11046238	chr12:21,950,324-22,094,336	AX-12405183

ACTC1	rs533021	chr15:35,080,297-35,087,927	AX-11543492
ACTC1	rs8037241	chr15:35,080,297-35,087,927	AX-12895725
ACTC1	rs589759	chr15:35,080,297-35,087,927	AX-107770170
ACTC1	rs589759	chr15:35,080,297-35,087,927	AX-123097322
ACTC1	rs34323254	chr15:35,080,297-35,087,927	AX-88766889
ACTC1	rs34323254	chr15:35,080,297-35,087,927	AX-88809934
ACTC1	rs1370155	chr15:35,080,297-35,087,927	AX-31584229
ACTC1	rs1370154	chr15:35,080,297-35,087,927	AX-12895731
ACTC1	rs74009720	chr15:35,080,297-35,087,927	AX-12895732
ACTC1	rs121912674	chr15:35,080,297-35,087,927	AX-88754026
ACTC1	rs267606629	chr15:35,080,297-35,087,927	AX-90055002
ACTC1	rs2307494	chr15:35,080,297-35,087,927	AX-31584239
ACTC1	---	chr15:35,080,297-35,087,927	AX-90030603
ACTC1	rs121912673	chr15:35,080,297-35,087,927	AX-88797070
ACTC1	rs543030688	chr15:35,080,297-35,087,927	AX-90055003
ACTC1	---	chr15:35,080,297-35,087,927	AX-90055004
ACTC1	---	chr15:35,080,297-35,087,927	AX-90055005
ACTC1	rs121912675	chr15:35,080,297-35,087,927	AX-88754027
ACTC1	---	chr15:35,080,297-35,087,927	AX-90055006
ACTC1	rs115415793	chr15:35,080,297-35,087,927	AX-31584241
ACTC1	rs62006139	chr15:35,080,297-35,087,927	AX-107799845
ACTC1	rs3729755	chr15:35,080,297-35,087,927	AX-12895738
ACTC1	---	chr15:35,080,297-35,087,927	AX-90030266
ACTC1	---	chr15:35,080,297-35,087,927	AX-90026123
ACTC1	---	chr15:35,080,297-35,087,927	AX-90072209
ACTC1	---	chr15:35,080,297-35,087,927	AX-90026124
ACTC1	rs267606628	chr15:35,080,297-35,087,927	AX-90026125
ACTC1	rs78697034	chr15:35,080,297-35,087,927	AX-12895739
ACTC1	rs2070664	chr15:35,080,297-35,087,927	AX-12895742
ACTC1	---	chr15:35,080,297-35,087,927	AX-90035570
ACTC1	rs193922680	chr15:35,080,297-35,087,927	AX-88797084
ACTC1	---	chr15:35,080,297-35,087,927	AX-90026126
ACTC1	---	chr15:35,080,297-35,087,927	AX-90048292
ACTC1	rs121912676	chr15:35,080,297-35,087,927	AX-88754028
ACTC1	---	chr15:35,080,297-35,087,927	AX-90052309
ACTC1	rs7165006	chr15:35,080,297-35,087,927	AX-12895744
ACTC1	rs7166290	chr15:35,080,297-35,087,927	AX-31584249
ACTC1	rs77350268	chr15:35,080,297-35,087,927	AX-31584251
ACTC1	rs7166484	chr15:35,080,297-35,087,927	AX-11611462
ACTC1	rs115586078	chr15:35,080,297-35,087,927	AX-31584253
ACTC1	rs28595759	chr15:35,080,297-35,087,927	AX-31584255
ACTC1	rs11629614	chr15:35,080,297-35,087,927	AX-62358117
ACTC1	rs77247635	chr15:35,080,297-35,087,927	AX-31584257
ACTC1	rs78848103	chr15:35,080,297-35,087,927	AX-12895746
ACTC1	---	chr15:35,080,297-35,087,927	AX-156278706
ACTC1	---	chr15:35,080,297-35,087,927	AX-156269309
ACTN2	rs121434525	chr1:236,849,770-236,927,558	AX-83357182
ACTN2	rs707221	chr1:236,849,770-236,927,558	AX-16947809
ACTN2	rs75716382	chr1:236,849,770-236,927,558	AX-16947820
ACTN2	rs115432678	chr1:236,849,770-236,927,558	AX-30720971
ACTN2	rs71721494	chr1:236,849,770-236,927,558	AX-156298314
ACTN2	rs7530968	chr1:236,849,770-236,927,558	AX-114085232
ACTN2	rs6656267	chr1:236,849,770-236,927,558	AX-51282400
ACTN2	rs10925200	chr1:236,849,770-236,927,558	AX-119579607
ACTN2	rs10925200	chr1:236,849,770-236,927,558	AX-148266783

ACTN2	rs72760272	chr1:236,849,770-236,927,558	AX-30721005
ACTN2	rs10465610	chr1:236,849,770-236,927,558	AX-30721007
ACTN2	rs3905125	chr1:236,849,770-236,927,558	AX-39409587
ACTN2	rs10802556	chr1:236,849,770-236,927,558	AX-156270947
ACTN2	rs707214	chr1:236,849,770-236,927,558	AX-11604873
ACTN2	rs140066235	chr1:236,849,770-236,927,558	AX-151221942
ACTN2	rs12739634	chr1:236,849,770-236,927,558	AX-50103992
ACTN2	rs819645	chr1:236,849,770-236,927,558	AX-156270951
ACTN2	rs819647	chr1:236,849,770-236,927,558	AX-156270952
ACTN2	rs4659703	chr1:236,849,770-236,927,558	AX-11515869
ACTN2	rs12067644	chr1:236,849,770-236,927,558	AX-30721045
ACTN2	rs12037834	chr1:236,849,770-236,927,558	AX-156270953
ACTN2	rs3910035	chr1:236,849,770-236,927,558	AX-16947872
ACTN2	rs12067001	chr1:236,849,770-236,927,558	AX-156270955
ACTN2	rs4659704	chr1:236,849,770-236,927,558	AX-113889821
ACTN2	rs4659704	chr1:236,849,770-236,927,558	AX-113963585
ACTN2	rs4659705	chr1:236,849,770-236,927,558	AX-30721069
ACTN2	rs819723	chr1:236,849,770-236,927,558	AX-96636384
ACTN2	rs71559976	chr1:236,849,770-236,927,558	AX-156285052
ACTN2	rs71559976	chr1:236,849,770-236,927,558	AX-30721071
ACTN2	rs112267838	chr1:236,849,770-236,927,558	AX-148501150
ACTN2	rs111507537	chr1:236,849,770-236,927,558	AX-113092712
ACTN2	rs111507537	chr1:236,849,770-236,927,558	AX-94348327
ACTN2	rs61831762	chr1:236,849,770-236,927,558	AX-120674319
ACTN2	---	chr1:236,849,770-236,927,558	AX-156270527
ACTN2	rs35601280	chr1:236,849,770-236,927,558	AX-113082702
ACTN2	rs35601280	chr1:236,849,770-236,927,558	AX-113601052
ACTN2	rs1418071	chr1:236,849,770-236,927,558	AX-39409623
ACTN2	rs202081364	chr1:236,849,770-236,927,558	AX-156284293
ACTN2	rs78688460	chr1:236,849,770-236,927,558	AX-16947874
ACTN2	rs111888885	chr1:236,849,770-236,927,558	AX-120159998
ACTN2	rs111888885	chr1:236,849,770-236,927,558	AX-156270529
ACTN2	rs2275399	chr1:236,849,770-236,927,558	AX-30721101
ACTN2	rs1366989	chr1:236,849,770-236,927,558	AX-11256886
ACTN2	rs397516579	chr1:236,849,770-236,927,558	AX-90034278
ACTN2	rs41269355	chr1:236,849,770-236,927,558	AX-30721103
ACTN2	rs1652226	chr1:236,849,770-236,927,558	AX-11280238
ACTN2	rs111438104	chr1:236,849,770-236,927,558	AX-148774889
ACTN2	rs111438104	chr1:236,849,770-236,927,558	AX-98053950
ACTN2	rs1341863	chr1:236,849,770-236,927,558	AX-16947877
ACTN2	rs1341862	chr1:236,849,770-236,927,558	AX-39409631
ACTN2	rs66469169	chr1:236,849,770-236,927,558	AX-151302312
ACTN2	rs112967359	chr1:236,849,770-236,927,558	AX-156270591
ACTN2	rs1622066	chr1:236,849,770-236,927,558	AX-30721115
ACTN2	rs1773441	chr1:236,849,770-236,927,558	AX-39409663
ACTN2	rs12081658	chr1:236,849,770-236,927,558	AX-16947888
ACTN2	rs2564764	chr1:236,849,770-236,927,558	AX-39409675
ACTN2	---	chr1:236,849,770-236,927,558	AX-156270959
ACTN2	rs2994382	chr1:236,849,770-236,927,558	AX-16947896
ACTN2	rs4659707	chr1:236,849,770-236,927,558	AX-148495629
ACTN2	rs76345693	chr1:236,849,770-236,927,558	AX-16947900
ACTN2	rs819640	chr1:236,849,770-236,927,558	AX-16947904
ACTN2	rs819641	chr1:236,849,770-236,927,558	AX-11667994
ACTN2	rs819725	chr1:236,849,770-236,927,558	AX-105054677
ACTN2	rs2039744	chr1:236,849,770-236,927,558	AX-11362196

ACTN2	rs4659708	chr1:236,849,770-236,927,558	AX-16947916
ACTN2	rs11355106	chr1:236,849,770-236,927,558	AX-107811671
ACTN2	rs12141939	chr1:236,849,770-236,927,558	AX-156270964
ACTN2	rs12138531	chr1:236,849,770-236,927,558	AX-148151140
ACTN2	rs3768125	chr1:236,849,770-236,927,558	AX-16947925
ACTN2	rs3768126	chr1:236,849,770-236,927,558	AX-30721157
ACTN2	rs2288601	chr1:236,849,770-236,927,558	AX-16947927
ACTN2	rs2288602	chr1:236,849,770-236,927,558	AX-30721161
ACTN2	rs2385493	chr1:236,849,770-236,927,558	AX-156270973
ACTN2	rs12087874	chr1:236,849,770-236,927,558	AX-156270965
ACTN2	rs2385494	chr1:236,849,770-236,927,558	AX-11388684
ACTN2	---	chr1:236,849,770-236,927,558	AX-156284434
ACTN2	rs2891794	chr1:236,849,770-236,927,558	AX-149098849
ACTN2	rs2385495	chr1:236,849,770-236,927,558	AX-148163191
ACTN2	rs2385495	chr1:236,849,770-236,927,558	AX-88790734
ACTN2	rs2385496	chr1:236,849,770-236,927,558	AX-156270966
ACTN2	rs2385497	chr1:236,849,770-236,927,558	AX-156270967
ACTN2	rs2385497	chr1:236,849,770-236,927,558	AX-156289615
ACTN2	rs7555260	chr1:236,849,770-236,927,558	AX-156289616
ACTN2	rs2891795	chr1:236,849,770-236,927,558	AX-11425203
ACTN2	rs116111094	chr1:236,849,770-236,927,558	AX-30721169
ACTN2	rs12137069	chr1:236,849,770-236,927,558	AX-120559650
ACTN2	rs61831786	chr1:236,849,770-236,927,558	AX-30721173
ACTN2	rs12090481	chr1:236,849,770-236,927,558	AX-30721177
ACTN2	rs148628512	chr1:236,849,770-236,927,558	AX-151193722
ACTN2	rs12737976	chr1:236,849,770-236,927,558	AX-30721181
ACTN2	---	chr1:236,849,770-236,927,558	AX-39409715
ACTN2	rs34583860	chr1:236,849,770-236,927,558	AX-16947935
ACTN2	rs34827377	chr1:236,849,770-236,927,558	AX-83244981
ACTN2	rs75222641	chr1:236,849,770-236,927,558	AX-30721199
ACTN2	rs10802557	chr1:236,849,770-236,927,558	AX-39409725
ACTN2	rs10802558	chr1:236,849,770-236,927,558	AX-11122969
ACTN2	rs7518515	chr1:236,849,770-236,927,558	AX-156270969
ACTN2	rs7518515	chr1:236,849,770-236,927,558	AX-156289618
ACTN2	rs2297956	chr1:236,849,770-236,927,558	AX-11383145
ACTN2	rs2297955	chr1:236,849,770-236,927,558	AX-11383144
ACTN2	rs4659712	chr1:236,849,770-236,927,558	AX-123026530
ACTN2	rs12143369	chr1:236,849,770-236,927,558	AX-11192801
ACTN2	rs10925213	chr1:236,849,770-236,927,558	AX-16947958
ACTN2	rs6677567	chr1:236,849,770-236,927,558	AX-151330830
ACTN2	rs2297860	chr1:236,849,770-236,927,558	AX-30721229
ACTN2	rs34975493	chr1:236,849,770-236,927,558	AX-39409745
ACTN2	rs3768127	chr1:236,849,770-236,927,558	AX-156289622
ACTN2	rs10925214	chr1:236,849,770-236,927,558	AX-113117883
ACTN2	rs78597259	chr1:236,849,770-236,927,558	AX-121845229
ACTN2	rs114373110	chr1:236,849,770-236,927,558	AX-156297589
ACTN2	rs76712595	chr1:236,849,770-236,927,558	AX-121853004
ACTN2	rs77166823	chr1:236,849,770-236,927,558	AX-148066482
ACTN2	rs10802561	chr1:236,849,770-236,927,558	AX-156270978
ACTN2	rs35311500	chr1:236,849,770-236,927,558	AX-156271214
ACTN2	rs12730862	chr1:236,849,770-236,927,558	AX-156270981
ACTN2	rs4348712	chr1:236,849,770-236,927,558	AX-148244317
ACTN2	rs1051251	chr1:236,849,770-236,927,558	AX-84960862
ACTN2	rs7522310	chr1:236,849,770-236,927,558	AX-16947978
AKAP9	rs2282971	chr7:91,570,189-91,739,987	AX-15777352

AKAP9	rs142401936	chr7:91,570,189-91,739,987	AX-83389422
AKAP9	rs35669569	chr7:91,570,189-91,739,987	AX-11467942
AKAP9	rs6972637	chr7:91,570,189-91,739,987	AX-11598196
AKAP9	rs35963176	chr7:91,570,189-91,739,987	AX-12558140
AKAP9	rs144888041	chr7:91,570,189-91,739,987	AX-82975857
AKAP9	---	chr7:91,570,189-91,739,987	AX-90080276
AKAP9	rs4729017	chr7:91,570,189-91,739,987	AX-15777422
AKAP9	rs6964587	chr7:91,570,189-91,739,987	AX-11597604
AKAP9	---	chr7:91,570,189-91,739,987	AX-90071991
AKAP9	rs1989779	chr7:91,570,189-91,739,987	AX-11357935
AKAP9	rs372572545	chr7:91,570,189-91,739,987	AX-94356858
AKAP9	rs13245393	chr7:91,570,189-91,739,987	AX-15777452
AKAP9	---	chr7:91,570,189-91,739,987	AX-86655148
AKAP9	rs146797353	chr7:91,570,189-91,739,987	AX-15777468
AKAP9	rs80306000	chr7:91,570,189-91,739,987	AX-15777502
AKAP9	rs150379637	chr7:91,570,189-91,739,987	AX-83443886
AKAP9	rs121908566	chr7:91,570,189-91,739,987	AX-86580018
AKAP9	---	chr7:91,570,189-91,739,987	AX-94356859
AKAP9	---	chr7:91,570,189-91,739,987	AX-38374467
AKAP9	rs146305558	chr7:91,570,189-91,739,987	AX-82974783
AKAP9	rs150016098	chr7:91,570,189-91,739,987	AX-83545043
AKAP9	rs151021935	chr7:91,570,189-91,739,987	AX-82900534
AKAP9	rs539680958	chr7:91,570,189-91,739,987	AX-90045982
AKAP9	---	chr7:91,570,189-91,739,987	AX-86636799
AKAP9	rs2285686	chr7:91,570,189-91,739,987	AX-86656338
AKAP9	---	chr7:91,570,189-91,739,987	AX-94356860
AKAP9	rs10236397	chr7:91,570,189-91,739,987	AX-15777573
AKAP9	---	chr7:91,570,189-91,739,987	AX-86644792
AKAP9	rs61757671	chr7:91,570,189-91,739,987	AX-83108620
AKAP9	rs73226383	chr7:91,570,189-91,739,987	AX-83252503
AKAP9	rs76177450	chr7:91,570,189-91,739,987	AX-83111269
AKAP9	---	chr7:91,570,189-91,739,987	AX-86700376
AKAP9	rs34581834	chr7:91,570,189-91,739,987	AX-11449387
AKAP9	rs61757672	chr7:91,570,189-91,739,987	AX-83404812
AKAP9	rs35759833	chr7:91,570,189-91,739,987	AX-36360277
AKAP9	rs144875383	chr7:91,570,189-91,739,987	AX-82906465
AKAP9	rs6960867	chr7:91,570,189-91,739,987	AX-11597305
AKAP9	rs1063242	chr7:91,570,189-91,739,987	AX-83577138
AKAP9	rs28927678	chr7:91,570,189-91,739,987	AX-15777621
AKAP9	---	chr7:91,570,189-91,739,987	AX-86585755
AKAP9	rs145355395	chr7:91,570,189-91,739,987	AX-86644214
AKAP9	rs61757663	chr7:91,570,189-91,739,987	AX-83499669
AKAP9	---	chr7:91,570,189-91,739,987	AX-94356861
AKAP9	rs1063243	chr7:91,570,189-91,739,987	AX-15777641
AKAP9	rs34327395	chr7:91,570,189-91,739,987	AX-11445071
AKAP9	rs141856443	chr7:91,570,189-91,739,987	AX-83468071
AKAP9	rs62465453	chr7:91,570,189-91,739,987	AX-36360331
AKAP9	rs57816416	chr7:91,570,189-91,739,987	AX-36360333
AKAP9	rs7785971	chr7:91,570,189-91,739,987	AX-112222477
AKAP9	rs2049900	chr7:91,570,189-91,739,987	AX-15777665
ANK2	rs72669658	chr4:113,739,239-114,304,896	AX-14447068
ANK2	rs7681443	chr4:113,739,239-114,304,896	AX-11638431
ANK2	rs17615061	chr4:113,739,239-114,304,896	AX-34511795
ANK2	rs72669667	chr4:113,739,239-114,304,896	AX-14447093
ANK2	rs17045043	chr4:113,739,239-114,304,896	AX-41331273

ANK2	rs80172156	chr4:113,739,239-114,304,896	AX-14447117
ANK2	rs10461149	chr4:113,739,239-114,304,896	AX-14447133
ANK2	rs11098176	chr4:113,739,239-114,304,896	AX-12406834
ANK2	rs17045062	chr4:113,739,239-114,304,896	AX-14447144
ANK2	rs17672142	chr4:113,739,239-114,304,896	AX-11334345
ANK2	rs17672170	chr4:113,739,239-114,304,896	AX-11334347
ANK2	rs1448224	chr4:113,739,239-114,304,896	AX-11264543
ANK2	rs1448221	chr4:113,739,239-114,304,896	AX-11264542
ANK2	rs78771853	chr4:113,739,239-114,304,896	AX-66179256
ANK2	rs17045076	chr4:113,739,239-114,304,896	AX-11300743
ANK2	rs62317087	chr4:113,739,239-114,304,896	AX-14447179
ANK2	rs17672625	chr4:113,739,239-114,304,896	AX-11334375
ANK2	rs73840923	chr4:113,739,239-114,304,896	AX-14447206
ANK2	rs72669682	chr4:113,739,239-114,304,896	AX-14447213
ANK2	rs75876568	chr4:113,739,239-114,304,896	AX-34511933
ANK2	rs62317090	chr4:113,739,239-114,304,896	AX-14447225
ANK2	rs17045102	chr4:113,739,239-114,304,896	AX-41331333
ANK2	rs28691029	chr4:113,739,239-114,304,896	AX-14447255
ANK2	rs10016365	chr4:113,739,239-114,304,896	AX-11087538
ANK2	rs78842575	chr4:113,739,239-114,304,896	AX-14447278
ANK2	rs80143944	chr4:113,739,239-114,304,896	AX-14447288
ANK2	rs115302470	chr4:113,739,239-114,304,896	AX-38158151
ANK2	rs72669685	chr4:113,739,239-114,304,896	AX-34511981
ANK2	rs60365916	chr4:113,739,239-114,304,896	AX-14447299
ANK2	rs77583732	chr4:113,739,239-114,304,896	AX-14447301
ANK2	rs72669686	chr4:113,739,239-114,304,896	AX-34511983
ANK2	rs4404544	chr4:113,739,239-114,304,896	AX-11504287
ANK2	rs72892477	chr4:113,739,239-114,304,896	AX-14447319
ANK2	rs77023571	chr4:113,739,239-114,304,896	AX-14447329
ANK2	rs75994832	chr4:113,739,239-114,304,896	AX-14447334
ANK2	rs10030826	chr4:113,739,239-114,304,896	AX-11088409
ANK2	rs17617357	chr4:113,739,239-114,304,896	AX-34512019
ANK2	rs949758	chr4:113,739,239-114,304,896	AX-11690112
ANK2	rs11940222	chr4:113,739,239-114,304,896	AX-14447371
ANK2	rs72894426	chr4:113,739,239-114,304,896	AX-14447378
ANK2	rs57162454	chr4:113,739,239-114,304,896	AX-66179591
ANK2	rs11930182	chr4:113,739,239-114,304,896	AX-12423296
ANK2	rs7664907	chr4:113,739,239-114,304,896	AX-41331403
ANK2	rs58298152	chr4:113,739,239-114,304,896	AX-14447418
ANK2	rs115148989	chr4:113,739,239-114,304,896	AX-14447420
ANK2	rs75870428	chr4:113,739,239-114,304,896	AX-14447423
ANK2	rs6820742	chr4:113,739,239-114,304,896	AX-11587792
ANK2	rs12501268	chr4:113,739,239-114,304,896	AX-11210656
ANK2	rs1354679	chr4:113,739,239-114,304,896	AX-14447435
ANK2	rs72894462	chr4:113,739,239-114,304,896	AX-14447436
ANK2	rs28444313	chr4:113,739,239-114,304,896	AX-41331425
ANK2	rs12508918	chr4:113,739,239-114,304,896	AX-11211194
ANK2	rs17680403	chr4:113,739,239-114,304,896	AX-34512147
ANK2	rs2055655	chr4:113,739,239-114,304,896	AX-14447465
ANK2	rs17045344	chr4:113,739,239-114,304,896	AX-12487347
ANK2	rs78977334	chr4:113,739,239-114,304,896	AX-14447472
ANK2	rs17618891	chr4:113,739,239-114,304,896	AX-11331553
ANK2	rs17680801	chr4:113,739,239-114,304,896	AX-11334809
ANK2	rs1395112	chr4:113,739,239-114,304,896	AX-11259534
ANK2	rs10029917	chr4:113,739,239-114,304,896	AX-34512205

ANK2	rs13103796	chr4:113,739,239-114,304,896	AX-14447512
ANK2	rs9994849	chr4:113,739,239-114,304,896	AX-11714691
ANK2	rs10015472	chr4:113,739,239-114,304,896	AX-11087485
ANK2	rs75173266	chr4:113,739,239-114,304,896	AX-66179837
ANK2	rs9685098	chr4:113,739,239-114,304,896	AX-14447540
ANK2	rs7654153	chr4:113,739,239-114,304,896	AX-11636568
ANK2	rs17045456	chr4:113,739,239-114,304,896	AX-41331493
ANK2	rs77422045	chr4:113,739,239-114,304,896	AX-14447544
ANK2	rs17045462	chr4:113,739,239-114,304,896	AX-11300781
ANK2	rs6856101	chr4:113,739,239-114,304,896	AX-11590204
ANK2	rs78095449	chr4:113,739,239-114,304,896	AX-14447547
ANK2	rs11098185	chr4:113,739,239-114,304,896	AX-11143554
ANK2	rs313936	chr4:113,739,239-114,304,896	AX-11436104
ANK2	rs3822280	chr4:113,739,239-114,304,896	AX-50400559
ANK2	rs56334442	chr4:113,739,239-114,304,896	AX-34512253
ANK2	rs80077887	chr4:113,739,239-114,304,896	AX-14447550
ANK2	rs1026975	chr4:113,739,239-114,304,896	AX-11103231
ANK2	rs931838	chr4:113,739,239-114,304,896	AX-11680692
ANK2	rs313985	chr4:113,739,239-114,304,896	AX-11436109
ANK2	rs313984	chr4:113,739,239-114,304,896	AX-34512269
ANK2	rs313979	chr4:113,739,239-114,304,896	AX-11436108
ANK2	rs116785665	chr4:113,739,239-114,304,896	AX-66179917
ANK2	rs72671580	chr4:113,739,239-114,304,896	AX-14447571
ANK2	rs11726017	chr4:113,739,239-114,304,896	AX-41331525
ANK2	rs7690846	chr4:113,739,239-114,304,896	AX-11639084
ANK2	rs644851	chr4:113,739,239-114,304,896	AX-14447574
ANK2	rs582050	chr4:113,739,239-114,304,896	AX-11547791
ANK2	rs4834311	chr4:113,739,239-114,304,896	AX-11529795
ANK2	rs2279891	chr4:113,739,239-114,304,896	AX-11381121
ANK2	rs2279893	chr4:113,739,239-114,304,896	AX-11381123
ANK2	rs78209121	chr4:113,739,239-114,304,896	AX-14447587
ANK2	rs7694725	chr4:113,739,239-114,304,896	AX-11639344
ANK2	rs67235248	chr4:113,739,239-114,304,896	AX-34512345
ANK2	rs72671592	chr4:113,739,239-114,304,896	AX-14447601
ANK2	rs72906144	chr4:113,739,239-114,304,896	AX-14447603
ANK2	rs313968	chr4:113,739,239-114,304,896	AX-41331543
ANK2	rs115137877	chr4:113,739,239-114,304,896	AX-14447608
ANK2	rs74597555	chr4:113,739,239-114,304,896	AX-14447651
ANK2	rs626267	chr4:113,739,239-114,304,896	AX-14447656
ANK2	rs9996681	chr4:113,739,239-114,304,896	AX-14447676
ANK2	rs313958	chr4:113,739,239-114,304,896	AX-11436107
ANK2	rs73841214	chr4:113,739,239-114,304,896	AX-34512447
ANK2	rs7682237	chr4:113,739,239-114,304,896	AX-14447733
ANK2	rs594604	chr4:113,739,239-114,304,896	AX-41331679
ANK2	rs62313807	chr4:113,739,239-114,304,896	AX-14447756
ANK2	rs1506062	chr4:113,739,239-114,304,896	AX-14447791
ANK2	rs17683210	chr4:113,739,239-114,304,896	AX-41331719
ANK2	rs2202535	chr4:113,739,239-114,304,896	AX-11374750
ANK2	rs13102062	chr4:113,739,239-114,304,896	AX-34512579
ANK2	rs6854397	chr4:113,739,239-114,304,896	AX-11590097
ANK2	rs17579913	chr4:113,739,239-114,304,896	AX-34512617
ANK2	rs12644844	chr4:113,739,239-114,304,896	AX-41331749
ANK2	rs13101312	chr4:113,739,239-114,304,896	AX-11239537
ANK2	rs403618	chr4:113,739,239-114,304,896	AX-34512655
ANK2	rs17444886	chr4:113,739,239-114,304,896	AX-11324509

ANK2	rs17590579	chr4:113,739,239-114,304,896	AX-11330250
ANK2	rs404950	chr4:113,739,239-114,304,896	AX-11489617
ANK2	rs4833422	chr4:113,739,239-114,304,896	AX-11529723
ANK2	rs10012958	chr4:113,739,239-114,304,896	AX-34512713
ANK2	rs62313181	chr4:113,739,239-114,304,896	AX-14447949
ANK2	rs1485585	chr4:113,739,239-114,304,896	AX-34512745
ANK2	rs3214164	chr4:113,739,239-114,304,896	AX-83199127
ANK2	rs116498616	chr4:113,739,239-114,304,896	AX-14448012
ANK2	rs28507635	chr4:113,739,239-114,304,896	AX-34512819
ANK2	rs1468223	chr4:113,739,239-114,304,896	AX-11266337
ANK2	rs115423781	chr4:113,739,239-114,304,896	AX-34512863
ANK2	rs10488901	chr4:113,739,239-114,304,896	AX-11109973
ANK2	rs112818121	chr4:113,739,239-114,304,896	AX-14448070
ANK2	rs56075451	chr4:113,739,239-114,304,896	AX-34512883
ANK2	rs362464	chr4:113,739,239-114,304,896	AX-11475801
ANK2	rs362461	chr4:113,739,239-114,304,896	AX-14448083
ANK2	---	chr4:113,739,239-114,304,896	AX-92618746
ANK2	rs362508	chr4:113,739,239-114,304,896	AX-34512895
ANK2	rs3025734	chr4:113,739,239-114,304,896	AX-11432155
ANK2	rs29336	chr4:113,739,239-114,304,896	AX-14448147
ANK2	rs29407	chr4:113,739,239-114,304,896	AX-41331935
ANK2	rs72675218	chr4:113,739,239-114,304,896	AX-34512977
ANK2	---	chr4:113,739,239-114,304,896	AX-94355464
ANK2	rs29388	chr4:113,739,239-114,304,896	AX-41331959
ANK2	rs29385	chr4:113,739,239-114,304,896	AX-11427953
ANK2	rs29327	chr4:113,739,239-114,304,896	AX-11427693
ANK2	rs200990814	chr4:113,739,239-114,304,896	AX-83365001
ANK2	rs17629658	chr4:113,739,239-114,304,896	AX-41332021
ANK2	rs29308	chr4:113,739,239-114,304,896	AX-14448244
ANK2	rs6837523	chr4:113,739,239-114,304,896	AX-11588962
ANK2	rs75257820	chr4:113,739,239-114,304,896	AX-38158313
ANK2	rs35388528	chr4:113,739,239-114,304,896	AX-11463100
ANK2	rs7684392	chr4:113,739,239-114,304,896	AX-14448286
ANK2	rs13126750	chr4:113,739,239-114,304,896	AX-41332057
ANK2	rs56256480	chr4:113,739,239-114,304,896	AX-34513123
ANK2	rs10005957	chr4:113,739,239-114,304,896	AX-11086888
ANK2	rs2272229	chr4:113,739,239-114,304,896	AX-14448335
ANK2	---	chr4:113,739,239-114,304,896	AX-92520897
ANK2	rs56148912	chr4:113,739,239-114,304,896	AX-14448353
ANK2	rs72544141	chr4:113,739,239-114,304,896	AX-82920125
ANK2	rs2272235	chr4:113,739,239-114,304,896	AX-14448359
ANK2	rs35249198	chr4:113,739,239-114,304,896	AX-11460743
ANK2	rs138842207	chr4:113,739,239-114,304,896	AX-82935989
ANK2	---	chr4:113,739,239-114,304,896	AX-94372215
ANK2	---	chr4:113,739,239-114,304,896	AX-90048094
ANK2	rs201005444	chr4:113,739,239-114,304,896	AX-83125938
ANK2	rs200765866	chr4:113,739,239-114,304,896	AX-83319883
ANK2	rs149645600	chr4:113,739,239-114,304,896	AX-83173944
ANK2	rs61734477	chr4:113,739,239-114,304,896	AX-34513173
ANK2	rs28377576	chr4:113,739,239-114,304,896	AX-41332073
ANK2	rs141191319	chr4:113,739,239-114,304,896	AX-83442077
ANK2	rs145895389	chr4:113,739,239-114,304,896	AX-83190304
ANK2	rs3733617	chr4:113,739,239-114,304,896	AX-41332077
ANK2	---	chr4:113,739,239-114,304,896	AX-94355466
ANK2	rs138928206	chr4:113,739,239-114,304,896	AX-83391944

ANK2	rs149043752	chr4:113,739,239-114,304,896	AX-83205280
ANK2	rs36210417	chr4:113,739,239-114,304,896	AX-83284707
ANK2	rs34270799	chr4:113,739,239-114,304,896	AX-11444097
ANK2	---	chr4:113,739,239-114,304,896	AX-94355467
ANK2	rs142908806	chr4:113,739,239-114,304,896	AX-83126514
ANK2	rs180843436	chr4:113,739,239-114,304,896	AX-86594126
ANK2	---	chr4:113,739,239-114,304,896	AX-86643123
ANK2	rs45570339	chr4:113,739,239-114,304,896	AX-34513201
ANK2	rs35728190	chr4:113,739,239-114,304,896	AX-34513203
ANK2	rs143228029	chr4:113,739,239-114,304,896	AX-86577334
ANK2	rs66785829	chr4:113,739,239-114,304,896	AX-83250290
ANK2	---	chr4:113,739,239-114,304,896	AX-90057636
ANK2	rs35530544	chr4:113,739,239-114,304,896	AX-12556504
ANK2	rs121912705	chr4:113,739,239-114,304,896	AX-83491348
ANK2	rs148654834	chr4:113,739,239-114,304,896	AX-82949484
ANK2	---	chr4:113,739,239-114,304,896	AX-90028844
ANK2	---	chr4:113,739,239-114,304,896	AX-86656492
ANK2	rs121912706	chr4:113,739,239-114,304,896	AX-83219620
ANK2	rs45454496	chr4:113,739,239-114,304,896	AX-83126387
ANK2	rs377555678	chr4:113,739,239-114,304,896	AX-92653851
ANK2	rs3733618	chr4:113,739,239-114,304,896	AX-11476680
ANK2	rs35446871	chr4:113,739,239-114,304,896	AX-11464119
ANKRD1	rs3939	chr10:92,671,857-92,681,032	AX-16427927
ANKRD1	rs17102362	chr10:92,671,857-92,681,032	AX-16427929
ANKRD1	rs11186367	chr10:92,671,857-92,681,032	AX-16427931
ANKRD1	rs35550482	chr10:92,671,857-92,681,032	AX-83558735
ANKRD1	rs377074932	chr10:92,671,857-92,681,032	AX-92011114
ANKRD1	---	chr10:92,671,857-92,681,032	AX-92009177
ANKRD1	rs3839929	chr10:92,671,857-92,681,032	AX-11484162
ANKRD1	rs150275749	chr10:92,671,857-92,681,032	AX-148792606
ANKRD1	rs150275749	chr10:92,671,857-92,681,032	AX-156297636
ANKRD1	---	chr10:92,671,857-92,681,032	AX-90061121
ANKRD1	rs4933200	chr10:92,671,857-92,681,032	AX-16427936
ANKRD1	rs201380490	chr10:92,671,857-92,681,032	AX-151333967
ANKRD1	rs201380490	chr10:92,671,857-92,681,032	AX-97324804
ANKRD1	---	chr10:92,671,857-92,681,032	AX-90025762
ANKRD1	rs147683124	chr10:92,671,857-92,681,032	AX-151406221
ANKRD1	rs147683124	chr10:92,671,857-92,681,032	AX-156284845
ANKRD1	rs59146072	chr10:92,671,857-92,681,032	AX-16427942
ANKRD1	rs10881853	chr10:92,671,857-92,681,032	AX-156278644
ANKRD1	rs10881853	chr10:92,671,857-92,681,032	AX-156294850
ANKRD1	rs145387010	chr10:92,671,857-92,681,032	AX-83104394
ANKRD1	rs142354133	chr10:92,671,857-92,681,032	AX-90037652
ANKRD1	rs11595794	chr10:92,671,857-92,681,032	AX-91978877
ANKRD1	rs11595794	chr10:92,671,857-92,681,032	AX-96645103
ANKRD1	rs10881854	chr10:92,671,857-92,681,032	AX-153734036
ANKRD1	rs201949081	chr10:92,671,857-92,681,032	AX-151220963
ANKRD1	rs201949081	chr10:92,671,857-92,681,032	AX-156283636
ANKRD1	rs56177217	chr10:92,671,857-92,681,032	AX-16427946
ANKRD1	rs114435632	chr10:92,671,857-92,681,032	AX-16427947
ANKRD1	rs10047267	chr10:92,671,857-92,681,032	AX-113228201
ANKRD1	rs10047267	chr10:92,671,857-92,681,032	AX-156279456
ANKRD1	rs150797476	chr10:92,671,857-92,681,032	AX-86589160
ANKRD1	---	chr10:92,671,857-92,681,032	AX-90027313
ANKRD1	---	chr10:92,671,857-92,681,032	AX-90054648

ANKRD1	rs10881855	chr10:92,671,857-92,681,032	AX-11129106
BAG3	rs11199059	chr10:121,410,882-121,437,329	AX-156288737
BAG3	rs112168846	chr10:121,410,882-121,437,329	AX-156278488
BAG3	rs2577336	chr10:121,410,882-121,437,329	AX-156288738
BAG3	rs196335	chr10:121,410,882-121,437,329	AX-147886140
BAG3	rs55725412	chr10:121,410,882-121,437,329	AX-29454795
BAG3	rs55980078	chr10:121,410,882-121,437,329	AX-29454797
BAG3	rs11199060	chr10:121,410,882-121,437,329	AX-114338869
BAG3	rs196336	chr10:121,410,882-121,437,329	AX-38734997
BAG3	rs12250770	chr10:121,410,882-121,437,329	AX-29454809
BAG3	rs196337	chr10:121,410,882-121,437,329	AX-29454811
BAG3	rs72840788	chr10:121,410,882-121,437,329	AX-156279413
BAG3	rs72840788	chr10:121,410,882-121,437,329	AX-156295121
BAG3	rs12413642	chr10:121,410,882-121,437,329	AX-148072000
BAG3	rs196339	chr10:121,410,882-121,437,329	AX-156269215
BAG3	rs196340	chr10:121,410,882-121,437,329	AX-156269216
BAG3	rs196340	chr10:121,410,882-121,437,329	AX-156288739
BAG3	rs12571609	chr10:121,410,882-121,437,329	AX-29454819
BAG3	rs196341	chr10:121,410,882-121,437,329	AX-38735005
BAG3	rs3847489	chr10:121,410,882-121,437,329	AX-38735007
BAG3	rs196342	chr10:121,410,882-121,437,329	AX-38735009
BAG3	rs196343	chr10:121,410,882-121,437,329	AX-38735015
BAG3	rs7906577	chr10:121,410,882-121,437,329	AX-14988655
BAG3	rs11598594	chr10:121,410,882-121,437,329	AX-38735017
BAG3	rs7086437	chr10:121,410,882-121,437,329	AX-156269217
BAG3	rs7086437	chr10:121,410,882-121,437,329	AX-156288740
BAG3	rs196344	chr10:121,410,882-121,437,329	AX-156269218
BAG3	rs196344	chr10:121,410,882-121,437,329	AX-156288741
BAG3	rs17099139	chr10:121,410,882-121,437,329	AX-14988683
BAG3	rs125585	chr10:121,410,882-121,437,329	AX-148222155
BAG3	rs125585	chr10:121,410,882-121,437,329	AX-96552066
BAG3	rs3892879	chr10:121,410,882-121,437,329	AX-75165424
BAG3	rs196345	chr10:121,410,882-121,437,329	AX-11356356
BAG3	rs4752339	chr10:121,410,882-121,437,329	AX-156279414
BAG3	rs4752339	chr10:121,410,882-121,437,329	AX-156295122
BAG3	rs2577338	chr10:121,410,882-121,437,329	AX-156279415
BAG3	rs150785296	chr10:121,410,882-121,437,329	AX-120899331
BAG3	rs150785296	chr10:121,410,882-121,437,329	AX-88775186
BAG3	rs11597623	chr10:121,410,882-121,437,329	AX-38735025
BAG3	rs2478804	chr10:121,410,882-121,437,329	AX-11394198
BAG3	rs171909	chr10:121,410,882-121,437,329	AX-114075874
BAG3	rs171909	chr10:121,410,882-121,437,329	AX-156278504
BAG3	rs185504	chr10:121,410,882-121,437,329	AX-148706064
BAG3	rs2577339	chr10:121,410,882-121,437,329	AX-122632357
BAG3	rs2577339	chr10:121,410,882-121,437,329	AX-92741757
BAG3	rs145846357	chr10:121,410,882-121,437,329	AX-156285567
BAG3	rs61869036	chr10:121,410,882-121,437,329	AX-151352290
BAG3	rs17099142	chr10:121,410,882-121,437,329	AX-11305970
BAG3	rs7894233	chr10:121,410,882-121,437,329	AX-14988764
BAG3	rs196316	chr10:121,410,882-121,437,329	AX-156269219
BAG3	rs196316	chr10:121,410,882-121,437,329	AX-156288742
BAG3	rs7909225	chr10:121,410,882-121,437,329	AX-14988767
BAG3	rs12358294	chr10:121,410,882-121,437,329	AX-14988768
BAG3	rs201794358	chr10:121,410,882-121,437,329	AX-156285718
BAG3	rs201794358	chr10:121,410,882-121,437,329	AX-84771300

BAG3	rs117550412	chr10:121,410,882-121,437,329	AX-151352295
BAG3	rs117550412	chr10:121,410,882-121,437,329	AX-156269220
BAG3	rs2577340	chr10:121,410,882-121,437,329	AX-11399311
BAG3	---	chr10:121,410,882-121,437,329	AX-156285455
BAG3	rs171910	chr10:121,410,882-121,437,329	AX-156269221
BAG3	rs171910	chr10:121,410,882-121,437,329	AX-156288743
BAG3	rs196318	chr10:121,410,882-121,437,329	AX-38735033
BAG3	rs11267763	chr10:121,410,882-121,437,329	AX-156284379
BAG3	rs196319	chr10:121,410,882-121,437,329	AX-105106414
BAG3	rs196320	chr10:121,410,882-121,437,329	AX-156288744
BAG3	rs7095308	chr10:121,410,882-121,437,329	AX-12620474
BAG3	rs11199063	chr10:121,410,882-121,437,329	AX-123081592
BAG3	rs11199063	chr10:121,410,882-121,437,329	AX-123081593
BAG3	rs17617337	chr10:121,410,882-121,437,329	AX-12501922
BAG3	rs10886529	chr10:121,410,882-121,437,329	AX-29454849
BAG3	rs196321	chr10:121,410,882-121,437,329	AX-29454851
BAG3	rs7099687	chr10:121,410,882-121,437,329	AX-38735037
BAG3	rs196322	chr10:121,410,882-121,437,329	AX-38735039
BAG3	rs4752340	chr10:121,410,882-121,437,329	AX-11523414
BAG3	rs196323	chr10:121,410,882-121,437,329	AX-29454853
BAG3	rs76040550	chr10:121,410,882-121,437,329	AX-60440441
BAG3	rs196325	chr10:121,410,882-121,437,329	AX-38735045
BAG3	rs2240777	chr10:121,410,882-121,437,329	AX-38735049
BAG3	rs387906874	chr10:121,410,882-121,437,329	AX-86577437
BAG3	rs35434411	chr10:121,410,882-121,437,329	AX-29454863
BAG3	---	chr10:121,410,882-121,437,329	AX-90055336
BAG3	---	chr10:121,410,882-121,437,329	AX-82917848
BAG3	---	chr10:121,410,882-121,437,329	AX-90066008
BAG3	---	chr10:121,410,882-121,437,329	AX-90061549
BAG3	rs387906875	chr10:121,410,882-121,437,329	AX-90026452
BAG3	rs2234962	chr10:121,410,882-121,437,329	AX-11377100
BAG3	rs196327	chr10:121,410,882-121,437,329	AX-29454865
BAG3	---	chr10:121,410,882-121,437,329	AX-83084262
BAG3	rs121918312	chr10:121,410,882-121,437,329	AX-90040280
BAG3	---	chr10:121,410,882-121,437,329	AX-90080924
BAG3	rs397514506	chr10:121,410,882-121,437,329	AX-86581118
BAG3	rs117671123	chr10:121,410,882-121,437,329	AX-83440281
BAG3	---	chr10:121,410,882-121,437,329	AX-90080035
BAG3	---	chr10:121,410,882-121,437,329	AX-90060850
BAG3	rs196328	chr10:121,410,882-121,437,329	AX-11356351
BAG3	rs2072361	chr10:121,410,882-121,437,329	AX-12516433
BAG3	rs196329	chr10:121,410,882-121,437,329	AX-12512319
BAG3	rs196330	chr10:121,410,882-121,437,329	AX-29454869
BAG3	rs3892668	chr10:121,410,882-121,437,329	AX-38735055
BAG3	rs35188721	chr10:121,410,882-121,437,329	AX-29454873
BAG3	rs196331	chr10:121,410,882-121,437,329	AX-156269225
BAG3	rs196331	chr10:121,410,882-121,437,329	AX-156288746
BAG3	rs196332	chr10:121,410,882-121,437,329	AX-38735057
BAG3	rs72842207	chr10:121,410,882-121,437,329	AX-29454877
BAG3	rs196333	chr10:121,410,882-121,437,329	AX-38735059
BAG3	rs196334	chr10:121,410,882-121,437,329	AX-29454879
BAG3	rs35060630	chr10:121,410,882-121,437,329	AX-119918573
BAG3	rs148952976	chr10:121,410,882-121,437,329	AX-156285402
BAG3	rs182954206	chr10:121,410,882-121,437,329	AX-148477583
BAG3	rs182954206	chr10:121,410,882-121,437,329	AX-156283659

BAG3	rs186876032	chr10:121,410,882-121,437,329	AX-148651517
BAG3	rs186876032	chr10:121,410,882-121,437,329	AX-156283660
BAG3	rs139363651	chr10:121,410,882-121,437,329	AX-105087410
BAG3	rs139363651	chr10:121,410,882-121,437,329	AX-119866661
BAG3	rs113882598	chr10:121,410,882-121,437,329	AX-147984483
BAG3	rs113882598	chr10:121,410,882-121,437,329	AX-153799477
BAG3	rs12146226	chr10:121,410,882-121,437,329	AX-120368804
BAG3	rs12146226	chr10:121,410,882-121,437,329	AX-153804989
BAG3	rs12572016	chr10:121,410,882-121,437,329	AX-121895095
BAG3	rs12572016	chr10:121,410,882-121,437,329	AX-156279416
BAG3	rs196294	chr10:121,410,882-121,437,329	AX-156265649
BAG3	rs196294	chr10:121,410,882-121,437,329	AX-156265684
BAG3	---	chr10:121,410,882-121,437,329	AX-90033111
BAG3	rs3858339	chr10:121,410,882-121,437,329	AX-14988972
BAG3	rs3858340	chr10:121,410,882-121,437,329	AX-83202757
BAG3	rs196295	chr10:121,410,882-121,437,329	AX-38735065
BAG3	rs397516881	chr10:121,410,882-121,437,329	AX-90031587
BAG3	rs397514507	chr10:121,410,882-121,437,329	AX-90060870
BAG3	---	chr10:121,410,882-121,437,329	AX-90035141
BAG3	rs387906876	chr10:121,410,882-121,437,329	AX-90026453
BAG3	rs8946	chr10:121,410,882-121,437,329	AX-38735067
BAG3	rs71758126	chr10:121,410,882-121,437,329	AX-107864675
BAG3	rs59447464	chr10:121,410,882-121,437,329	AX-156298235
BAG3	rs200363755	chr10:121,410,882-121,437,329	AX-84758074
BAG3	rs200363755	chr10:121,410,882-121,437,329	AX-84758075
BAG3	rs11199065	chr10:121,410,882-121,437,329	AX-29454889
BAG3	rs57771257	chr10:121,410,882-121,437,329	AX-14989015
BAG3	rs57771257	chr10:121,410,882-121,437,329	AX-156297664
CACNA1C	rs10848607	chr12:2,162,416-2,807,115	AX-30622253
CACNA1C	rs71454851	chr12:2,162,416-2,807,115	AX-30622481
CACNA1C	rs12318330	chr12:2,162,416-2,807,115	AX-30622533
CACNA1C	rs56018122	chr12:2,162,416-2,807,115	AX-16895281
CACNA1C	rs7972947	chr12:2,162,416-2,807,115	AX-11657168
CACNA1C	rs11837432	chr12:2,162,416-2,807,115	AX-39358069
CACNA1C	rs2238012	chr12:2,162,416-2,807,115	AX-11377435
CACNA1C	rs7310591	chr12:2,162,416-2,807,115	AX-11620420
CACNA1C	rs11062098	chr12:2,162,416-2,807,115	AX-39358153
CACNA1C	rs2238014	chr12:2,162,416-2,807,115	AX-39358163
CACNA1C	rs2238015	chr12:2,162,416-2,807,115	AX-11377436
CACNA1C	rs4765884	chr12:2,162,416-2,807,115	AX-16895628
CACNA1C	rs75616013	chr12:2,162,416-2,807,115	AX-16895653
CACNA1C	rs2238018	chr12:2,162,416-2,807,115	AX-50105879
CACNA1C	rs117492792	chr12:2,162,416-2,807,115	AX-16895765
CACNA1C	rs2283276	chr12:2,162,416-2,807,115	AX-39358375
CACNA1C	rs55966742	chr12:2,162,416-2,807,115	AX-16896299
CACNA1C	rs11062110	chr12:2,162,416-2,807,115	AX-11141129
CACNA1C	rs10774022	chr12:2,162,416-2,807,115	AX-11120703
CACNA1C	rs35386560	chr12:2,162,416-2,807,115	AX-30624717
CACNA1C	rs727268	chr12:2,162,416-2,807,115	AX-37571019
CACNA1C	rs2283280	chr12:2,162,416-2,807,115	AX-39358833
CACNA1C	rs6489349	chr12:2,162,416-2,807,115	AX-30625027
CACNA1C	rs55724489	chr12:2,162,416-2,807,115	AX-30625665
CACNA1C	rs78657525	chr12:2,162,416-2,807,115	AX-16897394
CACNA1C	rs73048397	chr12:2,162,416-2,807,115	AX-30626043
CACNA1C	rs34534613	chr12:2,162,416-2,807,115	AX-83563236

CACNA1C	rs121912776	chr12:2,162,416-2,807,115	AX-88752786
CACNA1C	rs7297252	chr12:2,162,416-2,807,115	AX-39359245
CACNA1C	rs111520377	chr12:2,162,416-2,807,115	AX-16897770
CACNA1C	rs2239133	chr12:2,162,416-2,807,115	AX-39359367
CACNA1C	rs118114122	chr12:2,162,416-2,807,115	AX-30626541
CACNA1C	rs75557252	chr12:2,162,416-2,807,115	AX-30626579
CACNA1C	rs1122864	chr12:2,162,416-2,807,115	AX-16898085
CACNA1C	rs117233773	chr12:2,162,416-2,807,115	AX-16898501
CACNA1C	rs76328006	chr12:2,162,416-2,807,115	AX-30627709
CACNA1C	rs73050442	chr12:2,162,416-2,807,115	AX-16898999
CACNA1C	rs77574474	chr12:2,162,416-2,807,115	AX-16899140
CACNA1C	rs2238041	chr12:2,162,416-2,807,115	AX-11377441
CACNA1C	rs4765900	chr12:2,162,416-2,807,115	AX-30629527
CACNA1C	rs56197809	chr12:2,162,416-2,807,115	AX-30629685
CACNA1C	rs74062239	chr12:2,162,416-2,807,115	AX-30629711
CACNA1C	rs117224316	chr12:2,162,416-2,807,115	AX-16899818
CACNA1C	rs10774029	chr12:2,162,416-2,807,115	AX-39360869
CACNA1C	rs11062139	chr12:2,162,416-2,807,115	AX-50753929
CACNA1C	rs61909092	chr12:2,162,416-2,807,115	AX-50106505
CACNA1C	rs2239015	chr12:2,162,416-2,807,115	AX-11377532
CACNA1C	rs7298845	chr12:2,162,416-2,807,115	AX-11619600
CACNA1C	rs61909094	chr12:2,162,416-2,807,115	AX-16900251
CACNA1C	rs7953823	chr12:2,162,416-2,807,115	AX-12645685
CACNA1C	rs74238853	chr12:2,162,416-2,807,115	AX-30630551
CACNA1C	rs2239017	chr12:2,162,416-2,807,115	AX-16900429
CACNA1C	rs7303140	chr12:2,162,416-2,807,115	AX-39361367
CACNA1C	rs2283288	chr12:2,162,416-2,807,115	AX-39361597
CACNA1C	rs78557449	chr12:2,162,416-2,807,115	AX-30631639
CACNA1C	rs73033311	chr12:2,162,416-2,807,115	AX-51189938
CACNA1C	rs2239022	chr12:2,162,416-2,807,115	AX-30632099
CACNA1C	rs11062155	chr12:2,162,416-2,807,115	AX-39362231
CACNA1C	rs77372211	chr12:2,162,416-2,807,115	AX-16901372
CACNA1C	rs77893928	chr12:2,162,416-2,807,115	AX-16901912
CACNA1C	rs2239032	chr12:2,162,416-2,807,115	AX-39363085
CACNA1C	rs145942808	chr12:2,162,416-2,807,115	AX-90067681
CACNA1C	rs111361579	chr12:2,162,416-2,807,115	AX-90038797
CACNA1C	rs113937823	chr12:2,162,416-2,807,115	AX-90067683
CACNA1C	rs7313380	chr12:2,162,416-2,807,115	AX-39363103
CACNA1C	rs76618989	chr12:2,162,416-2,807,115	AX-16902064
CACNA1C	rs193242952	chr12:2,162,416-2,807,115	AX-90038799
CACNA1C	rs569801785	chr12:2,162,416-2,807,115	AX-90053051
CACNA1C	rs547084113	chr12:2,162,416-2,807,115	AX-90057076
CACNA1C	rs117899250	chr12:2,162,416-2,807,115	AX-61432177
CACNA1C	rs3794297	chr12:2,162,416-2,807,115	AX-11481121
CACNA1C	rs117433140	chr12:2,162,416-2,807,115	AX-16902137
CACNA1C	rs1006737	chr12:2,162,416-2,807,115	AX-11090634
CACNA1C	rs12370782	chr12:2,162,416-2,807,115	AX-55955297
CACNA1C	rs4765905	chr12:2,162,416-2,807,115	AX-39363485
CACNA1C	rs73033370	chr12:2,162,416-2,807,115	AX-30634627
CACNA1C	rs7975229	chr12:2,162,416-2,807,115	AX-30634809
CACNA1C	rs78006379	chr12:2,162,416-2,807,115	AX-16902592
CACNA1C	rs61907761	chr12:2,162,416-2,807,115	AX-30635053
CACNA1C	rs2283292	chr12:2,162,416-2,807,115	AX-39364147
CACNA1C	rs115481484	chr12:2,162,416-2,807,115	AX-16903137
CACNA1C	rs79122070	chr12:2,162,416-2,807,115	AX-16903202

CACNA1C	rs74411248	chr12:2,162,416-2,807,115	AX-16903417
CACNA1C	rs61907778	chr12:2,162,416-2,807,115	AX-30636361
CACNA1C	rs75739143	chr12:2,162,416-2,807,115	AX-30637165
CACNA1C	rs79899792	chr12:2,162,416-2,807,115	AX-16903867
CACNA1C	rs56177308	chr12:2,162,416-2,807,115	AX-30637231
CACNA1C	rs73033382	chr12:2,162,416-2,807,115	AX-30637575
CACNA1C	rs75726475	chr12:2,162,416-2,807,115	AX-61434449
CACNA1C	rs4765913	chr12:2,162,416-2,807,115	AX-16904518
CACNA1C	rs2283293	chr12:2,162,416-2,807,115	AX-11381511
CACNA1C	rs2108570	chr12:2,162,416-2,807,115	AFFX-KIT-000131
CACNA1C	rs3819526	chr12:2,162,416-2,807,115	AX-39366299
CACNA1C	rs3819531	chr12:2,162,416-2,807,115	AX-30639227
CACNA1C	---	chr12:2,162,416-2,807,115	AX-61435066
CACNA1C	rs11062188	chr12:2,162,416-2,807,115	AX-39366393
CACNA1C	rs1544503	chr12:2,162,416-2,807,115	AX-30639313
CACNA1C	rs7963869	chr12:2,162,416-2,807,115	AX-30639463
CACNA1C	rs57009736	chr12:2,162,416-2,807,115	AX-30639683
CACNA1C	---	chr12:2,162,416-2,807,115	AX-39367021
CACNA1C	rs75784032	chr12:2,162,416-2,807,115	AX-37571167
CACNA1C	rs75559616	chr12:2,162,416-2,807,115	AX-30640255
CACNA1C	rs73035417	chr12:2,162,416-2,807,115	AX-30640273
CACNA1C	rs11062196	chr12:2,162,416-2,807,115	AX-39367207
CACNA1C	rs60656607	chr12:2,162,416-2,807,115	AX-30640511
CACNA1C	rs740418	chr12:2,162,416-2,807,115	AX-30640657
CACNA1C	rs2238076	chr12:2,162,416-2,807,115	AX-30640685
CACNA1C	rs116992907	chr12:2,162,416-2,807,115	AX-16906444
CACNA1C	rs11062203	chr12:2,162,416-2,807,115	AX-11141138
CACNA1C	rs12425203	chr12:2,162,416-2,807,115	AX-39367581
CACNA1C	rs886899	chr12:2,162,416-2,807,115	AX-30641557
CACNA1C	rs112653186	chr12:2,162,416-2,807,115	AX-30642663
CACNA1C	rs59241311	chr12:2,162,416-2,807,115	AX-30642917
CACNA1C	rs61907810	chr12:2,162,416-2,807,115	AX-30643137
CACNA1C	rs73037262	chr12:2,162,416-2,807,115	AX-30643191
CACNA1C	rs10774041	chr12:2,162,416-2,807,115	AX-30643291
CACNA1C	rs77663948	chr12:2,162,416-2,807,115	AX-16908190
CACNA1C	rs12823424	chr12:2,162,416-2,807,115	AX-11228320
CACNA1C	rs35407591	chr12:2,162,416-2,807,115	AX-39368941
CACNA1C	rs758116	chr12:2,162,416-2,807,115	AX-39369065
CACNA1C	rs112259837	chr12:2,162,416-2,807,115	AX-30643809
CACNA1C	rs11062222	chr12:2,162,416-2,807,115	AX-11141141
CACNA1C	rs11062223	chr12:2,162,416-2,807,115	AX-39369205
CACNA1C	rs1009281	chr12:2,162,416-2,807,115	AX-50108273
CACNA1C	rs73040095	chr12:2,162,416-2,807,115	AX-30644109
CACNA1C	rs994900	chr12:2,162,416-2,807,115	AX-50108312
CACNA1C	rs16929388	chr12:2,162,416-2,807,115	AX-11290583
CACNA1C	rs4765678	chr12:2,162,416-2,807,115	AX-39369531
CACNA1C	rs2239074	chr12:2,162,416-2,807,115	AX-11377541
CACNA1C	rs11613063	chr12:2,162,416-2,807,115	AX-11162477
CACNA1C	rs73042126	chr12:2,162,416-2,807,115	AX-30644903
CACNA1C	rs1860097	chr12:2,162,416-2,807,115	AX-11347427
CACNA1C	rs77768770	chr12:2,162,416-2,807,115	AX-30645089
CACNA1C	rs10774043	chr12:2,162,416-2,807,115	AX-39369881
CACNA1C	rs4765930	chr12:2,162,416-2,807,115	AX-39369923
CACNA1C	rs73042133	chr12:2,162,416-2,807,115	AX-30645309
CACNA1C	rs111915616	chr12:2,162,416-2,807,115	AX-30645363

CACNA1C	rs17801205	chr12:2,162,416-2,807,115	AX-30645365
CACNA1C	rs16927947	chr12:2,162,416-2,807,115	AX-39370005
CACNA1C	rs111856877	chr12:2,162,416-2,807,115	AX-30645511
CACNA1C	rs2239078	chr12:2,162,416-2,807,115	AX-39370047
CACNA1C	rs1076346	chr12:2,162,416-2,807,115	AX-39370307
CACNA1C	rs2239080	chr12:2,162,416-2,807,115	AX-39370319
CACNA1C	rs1544514	chr12:2,162,416-2,807,115	AX-39370431
CACNA1C	rs1544515	chr12:2,162,416-2,807,115	AX-30646109
CACNA1C	rs117010860	chr12:2,162,416-2,807,115	AX-16909562
CACNA1C	rs2239081	chr12:2,162,416-2,807,115	AX-11377543
CACNA1C	rs7135609	chr12:2,162,416-2,807,115	AX-16909686
CACNA1C	rs61909374	chr12:2,162,416-2,807,115	AX-16909714
CACNA1C	rs10774049	chr12:2,162,416-2,807,115	AX-30646597
CACNA1C	rs11062236	chr12:2,162,416-2,807,115	AX-39370861
CACNA1C	rs73042174	chr12:2,162,416-2,807,115	AX-30647159
CACNA1C	rs16929447	chr12:2,162,416-2,807,115	AX-11290588
CACNA1C	rs2239084	chr12:2,162,416-2,807,115	AX-39371097
CACNA1C	rs61909380	chr12:2,162,416-2,807,115	AX-30647409
CACNA1C	rs11836925	chr12:2,162,416-2,807,115	AX-39371201
CACNA1C	rs182565355	chr12:2,162,416-2,807,115	AX-90038801
CACNA1C	rs61909383	chr12:2,162,416-2,807,115	AX-16910374
CACNA1C	rs11062241	chr12:2,162,416-2,807,115	AX-11141142
CACNA1C	rs2239087	chr12:2,162,416-2,807,115	AX-11377545
CACNA1C	rs11062242	chr12:2,162,416-2,807,115	AX-11141143
CACNA1C	rs1860095	chr12:2,162,416-2,807,115	AX-39371487
CACNA1C	rs61477807	chr12:2,162,416-2,807,115	AX-16910571
CACNA1C	rs201756421	chr12:2,162,416-2,807,115	AX-83084164
CACNA1C	rs2239090	chr12:2,162,416-2,807,115	AX-39371815
CACNA1C	rs7306916	chr12:2,162,416-2,807,115	AX-11620171
CACNA1C	rs73046231	chr12:2,162,416-2,807,115	AX-30649035
CACNA1C	rs1015287	chr12:2,162,416-2,807,115	AX-11096334
CACNA1C	rs12319670	chr12:2,162,416-2,807,115	AX-12433283
CACNA1C	rs16929482	chr12:2,162,416-2,807,115	AX-30649177
CACNA1C	rs16929486	chr12:2,162,416-2,807,115	AX-11290592
CACNA1C	rs1004207	chr12:2,162,416-2,807,115	AX-30649325
CACNA1C	rs73046238	chr12:2,162,416-2,807,115	AX-30649329
CACNA1C	rs79346899	chr12:2,162,416-2,807,115	AX-30649353
CACNA1C	rs2239095	chr12:2,162,416-2,807,115	AX-30649481
CACNA1C	rs77858125	chr12:2,162,416-2,807,115	AX-30649611
CACNA1C	rs17223925	chr12:2,162,416-2,807,115	AX-39372387
CACNA1C	---	chr12:2,162,416-2,807,115	AX-37571247
CACNA1C	rs80315385	chr12:2,162,416-2,807,115	AX-88795801
CACNA1C	rs79891110	chr12:2,162,416-2,807,115	AX-90069400
CACNA1C	rs1860098	chr12:2,162,416-2,807,115	AX-30649913
CACNA1C	rs2239097	chr12:2,162,416-2,807,115	AX-11377547
CACNA1C	rs11062250	chr12:2,162,416-2,807,115	AX-39372695
CACNA1C	rs56343862	chr12:2,162,416-2,807,115	AX-30650283
CACNA1C	rs2239098	chr12:2,162,416-2,807,115	AX-30650433
CACNA1C	rs2239100	chr12:2,162,416-2,807,115	AX-30650463
CACNA1C	rs118020744	chr12:2,162,416-2,807,115	AX-30651073
CACNA1C	rs4765951	chr12:2,162,416-2,807,115	AX-39373187
CACNA1C	rs75392488	chr12:2,162,416-2,807,115	AX-30651145
CACNA1C	rs74481870	chr12:2,162,416-2,807,115	AX-30651189
CACNA1C	rs75933123	chr12:2,162,416-2,807,115	AX-30651203
CACNA1C	rs12231350	chr12:2,162,416-2,807,115	AX-12431078

CACNA1C	rs2239114	chr12:2,162,416-2,807,115	AX-30652167
CACNA1C	rs73046287	chr12:2,162,416-2,807,115	AX-30652265
CACNA1C	rs12318263	chr12:2,162,416-2,807,115	AX-30652523
CACNA1C	rs77005317	chr12:2,162,416-2,807,115	AX-30652531
CACNA1C	rs121912775	chr12:2,162,416-2,807,115	AX-83443797
CACNA1C	rs148852483	chr12:2,162,416-2,807,115	AX-90038802
CACNA1C	rs192403768	chr12:2,162,416-2,807,115	AX-90067688
CACNA1C	rs2370597	chr12:2,162,416-2,807,115	AX-30653741
CACNA1C	rs2370600	chr12:2,162,416-2,807,115	AX-16912991
CACNA1C	rs538906614	chr12:2,162,416-2,807,115	AX-90029277
CACNA1C	rs79468509	chr12:2,162,416-2,807,115	AX-30654507
CACNA1C	rs75384169	chr12:2,162,416-2,807,115	AX-30654709
CACNA1C	rs10744566	chr12:2,162,416-2,807,115	AX-30654937
CACNA1C	rs117358463	chr12:2,162,416-2,807,115	AX-30654955
CACNA1C	rs1034937	chr12:2,162,416-2,807,115	AX-50723378
CACNA1C	rs11832738	chr12:2,162,416-2,807,115	AX-39375527
CACNA1C	rs215976	chr12:2,162,416-2,807,115	AX-39375647
CACNA1C	rs112532048	chr12:2,162,416-2,807,115	AX-83557311
CACNA1C	rs141030741	chr12:2,162,416-2,807,115	AX-83577141
CACNA1C	rs11062282	chr12:2,162,416-2,807,115	AX-39375765
CACNA1C	rs77189734	chr12:2,162,416-2,807,115	AX-30655885
CACNA1C	rs77802779	chr12:2,162,416-2,807,115	AX-16914611
CACNA1C	rs215979	chr12:2,162,416-2,807,115	AX-30656061
CACNA1C	rs56269265	chr12:2,162,416-2,807,115	AX-16914722
CACNA1C	rs79174814	chr12:2,162,416-2,807,115	AX-37571289
CACNA1C	rs111316465	chr12:2,162,416-2,807,115	AX-90067690
CACNA1C	rs758560	chr12:2,162,416-2,807,115	AX-11631643
CACNA1C	---	chr12:2,162,416-2,807,115	AX-90064536
CACNA1C	rs215993	chr12:2,162,416-2,807,115	AX-39376511
CACNA1C	rs2239120	chr12:2,162,416-2,807,115	AX-30657057
CACNA1C	rs216008	chr12:2,162,416-2,807,115	AX-11371382
CACNA1C	rs2238091	chr12:2,162,416-2,807,115	AX-39376725
CACNA1C	rs2238092	chr12:2,162,416-2,807,115	AX-11377449
CACNA1C	rs11609212	chr12:2,162,416-2,807,115	AX-11162209
CACNA1C	rs2532614	chr12:2,162,416-2,807,115	AX-30657577
CACNA1C	rs216013	chr12:2,162,416-2,807,115	AX-11371389
CACNA1C	rs34935905	chr12:2,162,416-2,807,115	AX-11455496
CACNA1C	rs116790827	chr12:2,162,416-2,807,115	AX-16915594
CACNA1C	rs758563	chr12:2,162,416-2,807,115	AX-39377301
CACNA1C	rs11062287	chr12:2,162,416-2,807,115	AX-11141148
CACNA1C	rs12821981	chr12:2,162,416-2,807,115	AX-39377463
CACNA1C	rs11062288	chr12:2,162,416-2,807,115	AX-11141149
CACNA1C	rs2238096	chr12:2,162,416-2,807,115	AX-12522223
CACNA1C	rs2239124	chr12:2,162,416-2,807,115	AX-12522265
CACNA1C	rs117660151	chr12:2,162,416-2,807,115	AX-37571317
CACNA1C	rs216031	chr12:2,162,416-2,807,115	AX-39378003
CACNA1C	rs56180838	chr12:2,162,416-2,807,115	AX-30660265
CACNA1C	---	chr12:2,162,416-2,807,115	AX-37571325
CACNA1C	rs2302728	chr12:2,162,416-2,807,115	AX-39378629
CACNA1C	---	chr12:2,162,416-2,807,115	AX-90061547
CACNA1C	rs41276710	chr12:2,162,416-2,807,115	AX-16916903
CACNA1C	rs7295250	chr12:2,162,416-2,807,115	AX-16916960
CACNA1C	rs11062301	chr12:2,162,416-2,807,115	AX-39378683
CACNA1C	rs10744567	chr12:2,162,416-2,807,115	AX-39378869
CACNA1C	rs4765967	chr12:2,162,416-2,807,115	AX-39378945

CACNA1C	rs72552065	chr12:2,162,416-2,807,115	AX-30662239
CACNA1C	rs374425919	chr12:2,162,416-2,807,115	AX-86732821
CACNA1C	rs542995722	chr12:2,162,416-2,807,115	AX-86664321
CACNA1C	rs10848683	chr12:2,162,416-2,807,115	AX-83206799
CACNA1C	rs10774053	chr12:2,162,416-2,807,115	AX-83502864
CACNA1C	---	chr12:2,162,416-2,807,115	AX-90078808
CACNA1C	rs201777030	chr12:2,162,416-2,807,115	AX-83358209
CACNA1C	rs182208896	chr12:2,162,416-2,807,115	AX-83167753
CACNA1C	rs185788586	chr12:2,162,416-2,807,115	AX-83203111
CACNA1C	rs58010731	chr12:2,162,416-2,807,115	AX-30662893
CACNA1C	rs201090446	chr12:2,162,416-2,807,115	AX-83471542
CACNA1C	---	chr12:2,162,416-2,807,115	AX-90056234
CACNA1C	rs7957163	chr12:2,162,416-2,807,115	AX-11656039
CAV3	---	chr3:8,775,486-8,788,451	AX-90075807
CAV3	rs1974763	chr3:8,775,486-8,788,451	AX-14399479
CAV3	---	chr3:8,775,486-8,788,451	AX-90055867
CAV3	rs199476324	chr3:8,775,486-8,788,451	AX-88798926
CAV3	---	chr3:8,775,486-8,788,451	AX-90055868
CAV3	rs116840782	chr3:8,775,486-8,788,451	AX-90041969
CAV3	rs116840785	chr3:8,775,486-8,788,451	AX-90041970
CAV3	---	chr3:8,775,486-8,788,451	AX-90055869
CAV3	---	chr3:8,775,486-8,788,451	AX-41301885
CAV3	rs116840787	chr3:8,775,486-8,788,451	AX-90041971
CAV3	rs1558991	chr3:8,775,486-8,788,451	AX-41301889
CAV3	rs73021987	chr3:8,775,486-8,788,451	AX-34451441
CAV3	rs237870	chr3:8,775,486-8,788,451	AX-41301949
CAV3	rs56112390	chr3:8,775,486-8,788,451	AX-34451581
CAV3	rs237879	chr3:8,775,486-8,788,451	AX-41302013
CAV3	rs116840788	chr3:8,775,486-8,788,451	AX-90070857
CAV3	---	chr3:8,775,486-8,788,451	AX-90055870
CAV3	---	chr3:8,775,486-8,788,451	AX-90055871
CAV3	rs116840793	chr3:8,775,486-8,788,451	AX-90041973
CAV3	rs199476327	chr3:8,775,486-8,788,451	AX-90042041
CAV3	rs199476328	chr3:8,775,486-8,788,451	AX-88798927
CAV3	rs116840794	chr3:8,775,486-8,788,451	AX-90041974
CAV3	rs72546667	chr3:8,775,486-8,788,451	AX-34451731
CAV3	rs116840795	chr3:8,775,486-8,788,451	AX-90041975
CAV3	rs116840796	chr3:8,775,486-8,788,451	AX-90041976
CAV3	rs116840800	chr3:8,775,486-8,788,451	AX-90053863
CAV3	rs199476332	chr3:8,775,486-8,788,451	AX-90042043
CAV3	rs121909280	chr3:8,775,486-8,788,451	AX-90041985
CAV3	rs199476333	chr3:8,775,486-8,788,451	AX-88798928
CAV3	rs116840776	chr3:8,775,486-8,788,451	AX-83482048
CAV3	rs199476334	chr3:8,775,486-8,788,451	AX-88755884
CAV3	rs121909282	chr3:8,775,486-8,788,451	AX-90070871
CAV3	rs104893715	chr3:8,775,486-8,788,451	AX-90041948
CAV3	rs28936685	chr3:8,775,486-8,788,451	AX-83201032
CAV3	---	chr3:8,775,486-8,788,451	AX-90037922
CAV3	rs199476337	chr3:8,775,486-8,788,451	AX-88798930
CAV3	---	chr3:8,775,486-8,788,451	AX-90044715
CAV3	rs116840805	chr3:8,775,486-8,788,451	AX-88755684
CAV3	rs104893713	chr3:8,775,486-8,788,451	AX-88755629
CRYAB	rs370803064	chr11:111,779,350-111,782,473	AX-86555447
CRYAB	rs141638421	chr11:111,779,350-111,782,473	AX-86663673
CRYAB	rs150516929	chr11:111,779,350-111,782,473	AX-83121827

CRYAB	rs104894202	chr11:111,779,350-111,782,473	AX-90069252
CRYAB	---	chr11:111,779,350-111,782,473	AX-90049428
CRYAB	rs387907336	chr11:111,779,350-111,782,473	AX-90054649
CRYAB	rs104894201	chr11:111,779,350-111,782,473	AX-90069207
CRYAB	rs281865142	chr11:111,779,350-111,782,473	AX-86546262
CRYAB	rs387907339	chr11:111,779,350-111,782,473	AX-90027568
CRYAB	rs41302377	chr11:111,779,350-111,782,473	AX-11493712
CRYAB	rs2070894	chr11:111,779,350-111,782,473	AX-38955309
CRYAB	rs11603779	chr11:111,779,350-111,782,473	AX-38955311
CRYAB	rs139750142	chr11:111,779,350-111,782,473	AX-90069330
CRYAB	rs387907338	chr11:111,779,350-111,782,473	AX-90054650
CRYAB	---	chr11:111,779,350-111,782,473	AX-29854187
CRYAB	rs2234704	chr11:111,779,350-111,782,473	AX-11377089
CRYAB	rs281865141	chr11:111,779,350-111,782,473	AX-90042937
CRYAB	rs387907337	chr11:111,779,350-111,782,473	AX-90058716
CSRP3	rs45584634	chr11:19,203,577-19,232,118	AX-11511242
CSRP3	rs45599440	chr11:19,203,577-19,232,118	AX-29981119
CSRP3	rs45549043	chr11:19,203,577-19,232,118	AX-29981127
CSRP3	rs72904149	chr11:19,203,577-19,232,118	AX-29981131
CSRP3	rs11025046	chr11:19,203,577-19,232,118	AX-113890034
CSRP3	rs11025046	chr11:19,203,577-19,232,118	AX-156265780
CSRP3	rs11025047	chr11:19,203,577-19,232,118	AX-148603579
CSRP3	rs11025047	chr11:19,203,577-19,232,118	AX-156265781
CSRP3	rs9888275	chr11:19,203,577-19,232,118	AX-156279657
CSRP3	rs9888275	chr11:19,203,577-19,232,118	AX-156295286
CSRP3	rs9888224	chr11:19,203,577-19,232,118	AX-147884629
CSRP3	rs9888226	chr11:19,203,577-19,232,118	AX-29981137
CSRP3	rs9888225	chr11:19,203,577-19,232,118	AX-11708543
CSRP3	rs9888227	chr11:19,203,577-19,232,118	AX-39021971
CSRP3	rs7130460	chr11:19,203,577-19,232,118	AX-11608742
CSRP3	rs7102888	chr11:19,203,577-19,232,118	AX-11606938
CSRP3	rs11025048	chr11:19,203,577-19,232,118	AX-11138669
CSRP3	rs2288251	chr11:19,203,577-19,232,118	AX-16550407
CSRP3	rs7103903	chr11:19,203,577-19,232,118	AX-39021977
CSRP3	rs76668953	chr11:19,203,577-19,232,118	AX-16550412
CSRP3	rs9888186	chr11:19,203,577-19,232,118	AX-16550413
CSRP3	rs11025049	chr11:19,203,577-19,232,118	AX-29981151
CSRP3	rs10833068	chr11:19,203,577-19,232,118	AX-29981153
CSRP3	rs10833069	chr11:19,203,577-19,232,118	AX-11125473
CSRP3	rs13451	chr11:19,203,577-19,232,118	AX-11254741
CSRP3	rs138218523	chr11:19,203,577-19,232,118	AX-83289750
CSRP3	rs1077688	chr11:19,203,577-19,232,118	AX-12395649
CSRP3	rs79400310	chr11:19,203,577-19,232,118	AX-16550417
CSRP3	rs3781799	chr11:19,203,577-19,232,118	AX-11480241
CSRP3	rs3781800	chr11:19,203,577-19,232,118	AX-29981169
CSRP3	rs1077687	chr11:19,203,577-19,232,118	AX-11120890
CSRP3	rs1077686	chr11:19,203,577-19,232,118	AX-29981171
CSRP3	rs10833070	chr11:19,203,577-19,232,118	AX-156265782
CSRP3	rs10833070	chr11:19,203,577-19,232,118	AX-156286507
CSRP3	rs10833071	chr11:19,203,577-19,232,118	AX-11125474
CSRP3	rs6483581	chr11:19,203,577-19,232,118	AX-11567278
CSRP3	---	chr11:19,203,577-19,232,118	AX-86721715
CSRP3	rs137852764	chr11:19,203,577-19,232,118	AX-86611575
CSRP3	rs368392588	chr11:19,203,577-19,232,118	AX-86547908
CSRP3	rs104894204	chr11:19,203,577-19,232,118	AX-88752614

CSRP3	rs7124801	chr11:19,203,577-19,232,118	AX-39021981
CSRP3	rs145300736	chr11:19,203,577-19,232,118	AX-86714984
CSRP3	rs137852765	chr11:19,203,577-19,232,118	AX-86713998
CSRP3	rs104894205	chr11:19,203,577-19,232,118	AX-88795660
CSRP3	---	chr11:19,203,577-19,232,118	AX-90080578
CSRP3	rs2288250	chr11:19,203,577-19,232,118	AX-29981185
CSRP3	rs10766529	chr11:19,203,577-19,232,118	AX-29981187
CSRP3	rs10766530	chr11:19,203,577-19,232,118	AX-11120082
CSRP3	rs7128257	chr11:19,203,577-19,232,118	AX-156265783
CSRP3	rs7128257	chr11:19,203,577-19,232,118	AX-156286508
CSRP3	rs10766531	chr11:19,203,577-19,232,118	AX-29981193
CSRP3	rs10766532	chr11:19,203,577-19,232,118	AX-16550429
CSRP3	rs4757768	chr11:19,203,577-19,232,118	AX-29981199
CSRP3	rs7106581	chr11:19,203,577-19,232,118	AX-29981207
CSRP3	rs721067	chr11:19,203,577-19,232,118	AX-16550439
CSRP3	rs56384149	chr11:19,203,577-19,232,118	AX-16550440
CSRP3	rs10833072	chr11:19,203,577-19,232,118	AX-29981221
CSRP3	rs7933460	chr11:19,203,577-19,232,118	AX-16550448
CSRP3	rs7933466	chr11:19,203,577-19,232,118	AX-11654600
CSRP3	rs7933605	chr11:19,203,577-19,232,118	AX-29981229
CSRP3	rs45550635	chr11:19,203,577-19,232,118	AX-83584025
CSRP3	rs11025051	chr11:19,203,577-19,232,118	AX-29981233
CSRP3	rs12798277	chr11:19,203,577-19,232,118	AX-11227431
CSRP3	rs61876753	chr11:19,203,577-19,232,118	AX-29981237
CSRP3	rs112996135	chr11:19,203,577-19,232,118	AX-156265784
CSRP3	rs112996135	chr11:19,203,577-19,232,118	AX-156286509
CSRP3	rs55915915	chr11:19,203,577-19,232,118	AX-148350813
CSRP3	rs55915915	chr11:19,203,577-19,232,118	AX-156269986
CSRP3	rs58281741	chr11:19,203,577-19,232,118	AX-122343434
CSRP3	rs58281741	chr11:19,203,577-19,232,118	AX-156265785
CSRP3	rs12225248	chr11:19,203,577-19,232,118	AX-156265786
CSRP3	rs12225248	chr11:19,203,577-19,232,118	AX-156286510
CSRP3	rs11828825	chr11:19,203,577-19,232,118	AX-11177661
CSRP3	rs9804624	chr11:19,203,577-19,232,118	AX-148463984
CSRP3	rs9804624	chr11:19,203,577-19,232,118	AX-156265787
CSRP3	rs12290313	chr11:19,203,577-19,232,118	AX-39022003
CSRP3	rs12290229	chr11:19,203,577-19,232,118	AX-156265788
CSRP3	rs12290229	chr11:19,203,577-19,232,118	AX-156286511
CSRP3	rs7102081	chr11:19,203,577-19,232,118	AX-11606890
CSRP3	rs11025052	chr11:19,203,577-19,232,118	AX-16550465
CSRP3	rs10833073	chr11:19,203,577-19,232,118	AX-29981255
CSRP3	---	chr11:19,203,577-19,232,118	AX-96583850
CSRP3	rs188131336	chr11:19,203,577-19,232,118	AX-11421524
CSRP3	rs28716481	chr11:19,203,577-19,232,118	AX-96904888
CSRP3	---	chr11:19,203,577-19,232,118	AX-96560978
CSRP3	---	chr11:19,203,577-19,232,118	AX-96928758
CSRP3	rs139942624	chr11:19,203,577-19,232,118	AX-105038362
CSRP3	rs139942624	chr11:19,203,577-19,232,118	AX-156283676
CSRP3	rs149825099	chr11:19,203,577-19,232,118	AX-148715939
CSRP3	rs144769994	chr11:19,203,577-19,232,118	AX-148721018
CSRP3	rs7951347	chr11:19,203,577-19,232,118	AX-156265789
CSRP3	rs7951347	chr11:19,203,577-19,232,118	AX-156286512
CSRP3	rs35236689	chr11:19,203,577-19,232,118	AX-11460553
CSRP3	rs66540504	chr11:19,203,577-19,232,118	AX-107760041
CSRP3	rs66540504	chr11:19,203,577-19,232,118	AX-156297687

CSRP3	rs11025054	chr11:19,203,577-19,232,118	AX-29981257
CSRP3	rs77055095	chr11:19,203,577-19,232,118	AX-29981261
CSRP3	rs12276811	chr11:19,203,577-19,232,118	AX-29981263
CSRP3	rs74896206	chr11:19,203,577-19,232,118	AX-16550476
CSRP3	rs77820686	chr11:19,203,577-19,232,118	AX-16550477
CSRP3	rs72904176	chr11:19,203,577-19,232,118	AX-29981267
CSRP3	rs1346117	chr11:19,203,577-19,232,118	AX-39022017
CSRP3	rs10741762	chr11:19,203,577-19,232,118	AX-16550479
CSRP3	rs75264737	chr11:19,203,577-19,232,118	AX-16550480
CSRP3	rs10766533	chr11:19,203,577-19,232,118	AX-16550483
CSRP3	rs10741763	chr11:19,203,577-19,232,118	AX-39022023
CSRP3	rs55646411	chr11:19,203,577-19,232,118	AX-113133775
CSRP3	rs55646411	chr11:19,203,577-19,232,118	AX-114063203
CSRP3	rs10766534	chr11:19,203,577-19,232,118	AX-11120083
CSRP3	rs10766535	chr11:19,203,577-19,232,118	AX-88733909
CSRP3	rs10766535	chr11:19,203,577-19,232,118	AX-88776954
CSRP3	rs10766536	chr11:19,203,577-19,232,118	AX-114098224
CSRP3	rs10741764	chr11:19,203,577-19,232,118	AX-29981289
CSRP3	rs11025055	chr11:19,203,577-19,232,118	AX-156265790
CSRP3	rs57725010	chr11:19,203,577-19,232,118	AX-16550494
CSRP3	rs7104244	chr11:19,203,577-19,232,118	AX-39022033
CSRP3	rs72904181	chr11:19,203,577-19,232,118	AX-16550499
CSRP3	rs11025056	chr11:19,203,577-19,232,118	AX-11138670
CSRP3	rs11025057	chr11:19,203,577-19,232,118	AX-11138671
CSRP3	rs11604807	chr11:19,203,577-19,232,118	AX-11161901
CSRP3	rs793279	chr11:19,203,577-19,232,118	AX-39022035
CSRP3	rs7112377	chr11:19,203,577-19,232,118	AX-39022037
DES	rs62636490	chr2:220,283,099-220,291,461	AX-88798310
DES	rs267607497	chr2:220,283,099-220,291,461	AX-90071579
DES	---	chr2:220,283,099-220,291,461	AX-90072974
DES	rs267607499	chr2:220,283,099-220,291,461	AX-90055700
DES	---	chr2:220,283,099-220,291,461	AX-90031525
DES	rs111548596	chr2:220,283,099-220,291,461	AX-96734049
DES	rs111548596	chr2:220,283,099-220,291,461	AX-97386147
DES	rs12991025	chr2:220,283,099-220,291,461	AX-33536297
DES	rs41272699	chr2:220,283,099-220,291,461	AX-40847343
DES	rs377337947	chr2:220,283,099-220,291,461	AX-86656733
DES	---	chr2:220,283,099-220,291,461	AX-90028684
DES	rs267607484	chr2:220,283,099-220,291,461	AX-90057584
DES	---	chr2:220,283,099-220,291,461	AX-90057067
DES	---	chr2:220,283,099-220,291,461	AX-90044214
DES	---	chr2:220,283,099-220,291,461	AX-90032852
DES	---	chr2:220,283,099-220,291,461	AX-90051480
DES	rs397516698	chr2:220,283,099-220,291,461	AX-90057014
DES	rs267607483	chr2:220,283,099-220,291,461	AX-90029154
DES	rs41272701	chr2:220,283,099-220,291,461	AX-33536299
DES	---	chr2:220,283,099-220,291,461	AX-90064091
DES	rs1058261	chr2:220,283,099-220,291,461	AX-13848347
DES	rs62636491	chr2:220,283,099-220,291,461	AX-86592544
DES	rs570056785	chr2:220,283,099-220,291,461	AX-90078716
DES	rs34337334	chr2:220,283,099-220,291,461	AX-83322569
DES	---	chr2:220,283,099-220,291,461	AX-90041675
DES	rs57496341	chr2:220,283,099-220,291,461	AX-90033010
DES	rs12920	chr2:220,283,099-220,291,461	AX-40847345
DES	rs267607482	chr2:220,283,099-220,291,461	AX-90056485

DES	rs57639980	chr2:220,283,099-220,291,461	AX-90070553
DES	rs61368398	chr2:220,283,099-220,291,461	AX-90041678
DES	rs58898021	chr2:220,283,099-220,291,461	AX-90070557
DES	rs121913000	chr2:220,283,099-220,291,461	AX-90041829
DES	---	chr2:220,283,099-220,291,461	AX-90080671
DES	rs62636494	chr2:220,283,099-220,291,461	AX-90041683
DES	rs1058284	chr2:220,283,099-220,291,461	AX-11116618
DES	---	chr2:220,283,099-220,291,461	AX-90065883
DES	rs57955682	chr2:220,283,099-220,291,461	AX-90041670
DES	rs121913004	chr2:220,283,099-220,291,461	AX-90041666
DES	rs62636493	chr2:220,283,099-220,291,461	AX-90041682
DES	---	chr2:220,283,099-220,291,461	AX-90041830
DES	rs61130669	chr2:220,283,099-220,291,461	AX-90041677
DES	rs57694264	chr2:220,283,099-220,291,461	AX-90070554
DES	rs121913003	chr2:220,283,099-220,291,461	AX-90041679
DES	rs2854895	chr2:220,283,099-220,291,461	AX-148092115
DES	rs4674393	chr2:220,283,099-220,291,461	AX-33536307
DES	rs72963600	chr2:220,283,099-220,291,461	AX-33536309
DES	rs112203191	chr2:220,283,099-220,291,461	AX-96924364
DES	rs112203191	chr2:220,283,099-220,291,461	AX-97463232
DES	rs12621188	chr2:220,283,099-220,291,461	AX-33536319
DES	rs62635763	chr2:220,283,099-220,291,461	AX-90059145
DES	rs2854903	chr2:220,283,099-220,291,461	AX-13848350
DES	rs10804273	chr2:220,283,099-220,291,461	AX-148300441
DES	rs4674394	chr2:220,283,099-220,291,461	AX-96904764
DES	rs4674394	chr2:220,283,099-220,291,461	AX-96928655
DES	rs58898098	chr2:220,283,099-220,291,461	AX-33536333
DES	---	chr2:220,283,099-220,291,461	AX-90027722
DES	rs121913005	chr2:220,283,099-220,291,461	AX-90070716
DES	rs267607498	chr2:220,283,099-220,291,461	AX-90061566
DES	---	chr2:220,283,099-220,291,461	AX-90060570
DES	rs267607485	chr2:220,283,099-220,291,461	AX-90063031
DES	---	chr2:220,283,099-220,291,461	AX-90026817
DES	rs267607496	chr2:220,283,099-220,291,461	AX-90064321
DES	rs73991549	chr2:220,283,099-220,291,461	AX-33536337
DES	rs267607491	chr2:220,283,099-220,291,461	AX-90065291
DES	rs267607487	chr2:220,283,099-220,291,461	AX-90055704
DES	---	chr2:220,283,099-220,291,461	AX-90059812
DES	rs11685408	chr2:220,283,099-220,291,461	AX-123048664
DES	rs11685408	chr2:220,283,099-220,291,461	AX-123048665
DMD	rs16989352	chrX:31,137,345-33,357,726	AX-42750299
DMD	---	chrX:31,137,345-33,357,726	AX-90079083
DMD	rs5927689	chrX:31,137,345-33,357,726	AX-16350243
DMD	rs2170963	chrX:31,137,345-33,357,726	AX-11372240
DMD	rs1484852	chrX:31,137,345-33,357,726	AX-11267966
DMD	rs201217593	chrX:31,137,345-33,357,726	AX-86641226
DMD	rs5927694	chrX:31,137,345-33,357,726	AX-11550838
DMD	rs6631256	chrX:31,137,345-33,357,726	AX-11575604
DMD	rs41312092	chrX:31,137,345-33,357,726	AX-37241203
DMD	rs73462347	chrX:31,137,345-33,357,726	AX-37241209
DMD	rs2178539	chrX:31,137,345-33,357,726	AX-12519927
DMD	rs5972331	chrX:31,137,345-33,357,726	AX-37241217
DMD	rs6631263	chrX:31,137,345-33,357,726	AX-16350280
DMD	rs1317640	chrX:31,137,345-33,357,726	AX-11242977
DMD	rs147083316	chrX:31,137,345-33,357,726	AX-38622815

DMD	---	chrX:31,137,345-33,357,726	AX-86629050
DMD	rs141392048	chrX:31,137,345-33,357,726	AX-83159561
DMD	rs2293668	chrX:31,137,345-33,357,726	AX-12524761
DMD	rs10482232	chrX:31,137,345-33,357,726	AX-11108947
DMD	rs73617055	chrX:31,137,345-33,357,726	AX-37241249
DMD	rs12389409	chrX:31,137,345-33,357,726	AX-42750445
DMD	rs5971552	chrX:31,137,345-33,357,726	AX-11553315
DMD	rs2141755	chrX:31,137,345-33,357,726	AX-11369985
DMD	rs5926996	chrX:31,137,345-33,357,726	AX-11550766
DMD	rs5927708	chrX:31,137,345-33,357,726	AX-11550843
DMD	rs7889559	chrX:31,137,345-33,357,726	AX-11652008
DMD	rs5971553	chrX:31,137,345-33,357,726	AX-51290750
DMD	rs12855949	chrX:31,137,345-33,357,726	AX-11229241
DMD	rs1921381	chrX:31,137,345-33,357,726	AX-11352865
DMD	rs5972341	chrX:31,137,345-33,357,726	AX-42750471
DMD	rs77986513	chrX:31,137,345-33,357,726	AX-38521067
DMD	rs10127097	chrX:31,137,345-33,357,726	AX-11094554
DMD	rs2404500	chrX:31,137,345-33,357,726	AX-12528284
DMD	rs12840452	chrX:31,137,345-33,357,726	AX-11228851
DMD	rs5927001	chrX:31,137,345-33,357,726	AX-11550769
DMD	rs1921396	chrX:31,137,345-33,357,726	AX-42750491
DMD	rs12843613	chrX:31,137,345-33,357,726	AX-42750495
DMD	rs5972346	chrX:31,137,345-33,357,726	AX-11553363
DMD	rs56157001	chrX:31,137,345-33,357,726	AX-37241363
DMD	rs1573952	chrX:31,137,345-33,357,726	AX-11276969
DMD	rs17340630	chrX:31,137,345-33,357,726	AX-37241381
DMD	rs17338423	chrX:31,137,345-33,357,726	AX-11320215
DMD	rs12015000	chrX:31,137,345-33,357,726	AX-11186550
DMD	rs189123449	chrX:31,137,345-33,357,726	AX-38622939
DMD	rs138287985	chrX:31,137,345-33,357,726	AX-83125647
DMD	rs151056989	chrX:31,137,345-33,357,726	AX-38622955
DMD	rs146998764	chrX:31,137,345-33,357,726	AX-38622961
DMD	rs55803048	chrX:31,137,345-33,357,726	AX-37241423
DMD	rs139539984	chrX:31,137,345-33,357,726	AX-38622971
DMD	rs5972363	chrX:31,137,345-33,357,726	AX-16350326
DMD	rs11095206	chrX:31,137,345-33,357,726	AX-11143365
DMD	rs5927716	chrX:31,137,345-33,357,726	AX-11550847
DMD	rs12559712	chrX:31,137,345-33,357,726	AX-11214864
DMD	rs1950114	chrX:31,137,345-33,357,726	AX-11355254
DMD	rs2178537	chrX:31,137,345-33,357,726	AX-51282660
DMD	rs12836134	chrX:31,137,345-33,357,726	AX-42750589
DMD	rs12008262	chrX:31,137,345-33,357,726	AX-16350346
DMD	rs138321724	chrX:31,137,345-33,357,726	AX-38623035
DMD	rs12557091	chrX:31,137,345-33,357,726	AX-11214718
DMD	rs5927721	chrX:31,137,345-33,357,726	AX-11550849
DMD	rs12353755	chrX:31,137,345-33,357,726	AX-50801994
DMD	rs12844827	chrX:31,137,345-33,357,726	AX-11228967
DMD	rs5971568	chrX:31,137,345-33,357,726	AX-37241571
DMD	rs5927726	chrX:31,137,345-33,357,726	AX-12592169
DMD	rs142296114	chrX:31,137,345-33,357,726	AX-38623131
DMD	rs5927012	chrX:31,137,345-33,357,726	AX-12592130
DMD	rs2733459	chrX:31,137,345-33,357,726	AX-16350368
DMD	rs2178618	chrX:31,137,345-33,357,726	AX-12519930
DMD	rs61633449	chrX:31,137,345-33,357,726	AX-37241659
DMD	rs2704904	chrX:31,137,345-33,357,726	AX-11406018

DMD	rs2704908	chrX:31,137,345-33,357,726	AX-11406019
DMD	rs2692984	chrX:31,137,345-33,357,726	AX-42750685
DMD	rs16998181	chrX:31,137,345-33,357,726	AX-42750687
DMD	rs7062234	chrX:31,137,345-33,357,726	AX-12619366
DMD	rs2704909	chrX:31,137,345-33,357,726	AX-11406020
DMD	rs17338507	chrX:31,137,345-33,357,726	AX-12498520
DMD	rs2733466	chrX:31,137,345-33,357,726	AX-12536551
DMD	rs6527086	chrX:31,137,345-33,357,726	AX-11569753
DMD	rs1921968	chrX:31,137,345-33,357,726	AX-11352913
DMD	rs7054748	chrX:31,137,345-33,357,726	AX-42750725
DMD	rs17330006	chrX:31,137,345-33,357,726	AX-11319827
DMD	rs17338535	chrX:31,137,345-33,357,726	AX-37241763
DMD	rs4829222	chrX:31,137,345-33,357,726	AX-11529315
DMD	rs1112432	chrX:31,137,345-33,357,726	AX-11145290
DMD	rs7064141	chrX:31,137,345-33,357,726	AX-11604382
DMD	rs17340798	chrX:31,137,345-33,357,726	AX-11320313
DMD	rs12014023	chrX:31,137,345-33,357,726	AX-12425759
DMD	rs59882236	chrX:31,137,345-33,357,726	AX-37241877
DMD	rs1800280	chrX:31,137,345-33,357,726	AX-11344601
DMD	rs1800278	chrX:31,137,345-33,357,726	AX-11344600
DMD	rs41305353	chrX:31,137,345-33,357,726	AX-83535731
DMD	rs128627256	chrX:31,137,345-33,357,726	AX-90042539
DMD	rs17338604	chrX:31,137,345-33,357,726	AX-12498525
DMD	rs6628610	chrX:31,137,345-33,357,726	AX-11575503
DMD	rs16989681	chrX:31,137,345-33,357,726	AX-42750833
DMD	rs116594347	chrX:31,137,345-33,357,726	AX-38521119
DMD	rs5927762	chrX:31,137,345-33,357,726	AX-11550857
DMD	rs5927017	chrX:31,137,345-33,357,726	AX-42750853
DMD	rs5927764	chrX:31,137,345-33,357,726	AX-11550859
DMD	rs7881088	chrX:31,137,345-33,357,726	AX-11651708
DMD	rs5972404	chrX:31,137,345-33,357,726	AX-12593323
DMD	rs17338611	chrX:31,137,345-33,357,726	AX-37242051
DMD	rs5927779	chrX:31,137,345-33,357,726	AX-11550861
DMD	rs716354	chrX:31,137,345-33,357,726	AX-11611227
DMD	---	chrX:31,137,345-33,357,726	AX-94359750
DMD	rs112870072	chrX:31,137,345-33,357,726	AX-38623599
DMD	rs34542798	chrX:31,137,345-33,357,726	AX-11448738
DMD	rs62587358	chrX:31,137,345-33,357,726	AX-37242253
DMD	rs5971586	chrX:31,137,345-33,357,726	AX-37242255
DMD	rs1801188	chrX:31,137,345-33,357,726	AX-16350533
DMD	rs73210126	chrX:31,137,345-33,357,726	AX-37242289
DMD	rs5927030	chrX:31,137,345-33,357,726	AX-16350546
DMD	rs1540705	chrX:31,137,345-33,357,726	AX-16350556
DMD	rs17283259	chrX:31,137,345-33,357,726	AX-16350581
DMD	rs766671	chrX:31,137,345-33,357,726	AX-16350584
DMD	rs140658704	chrX:31,137,345-33,357,726	AX-38623703
DMD	rs5927822	chrX:31,137,345-33,357,726	AX-11550867
DMD	rs73210155	chrX:31,137,345-33,357,726	AX-37242407
DMD	rs151244052	chrX:31,137,345-33,357,726	AX-83283778
DMD	rs5927825	chrX:31,137,345-33,357,726	AX-11550868
DMD	rs73210174	chrX:31,137,345-33,357,726	AX-37242463
DMD	rs56678497	chrX:31,137,345-33,357,726	AX-37242465
DMD	rs4433306	chrX:31,137,345-33,357,726	AX-12572140
DMD	rs73213848	chrX:31,137,345-33,357,726	AX-37242479
DMD	rs2646305	chrX:31,137,345-33,357,726	AX-16350600

DMD	rs112041498	chrX:31,137,345-33,357,726	AX-38623737
DMD	rs10521979	chrX:31,137,345-33,357,726	AX-11116224
DMD	rs112516305	chrX:31,137,345-33,357,726	AX-16350608
DMD	rs5972445	chrX:31,137,345-33,357,726	AX-11553372
DMD	rs5927042	chrX:31,137,345-33,357,726	AX-16350620
DMD	rs6628646	chrX:31,137,345-33,357,726	AX-12604531
DMD	rs73213882	chrX:31,137,345-33,357,726	AX-37242649
DMD	rs73464049	chrX:31,137,345-33,357,726	AX-37242701
DMD	rs72466590	chrX:31,137,345-33,357,726	AX-83491701
DMD	rs73619010	chrX:31,137,345-33,357,726	AX-37242707
DMD	rs148452632	chrX:31,137,345-33,357,726	AX-38623813
DMD	rs2952922	chrX:31,137,345-33,357,726	AX-16350656
DMD	rs17341038	chrX:31,137,345-33,357,726	AX-11320320
DMD	rs56279781	chrX:31,137,345-33,357,726	AX-37242789
DMD	rs5972452	chrX:31,137,345-33,357,726	AX-12593328
DMD	rs1800275	chrX:31,137,345-33,357,726	AX-11344598
DMD	rs1545662	chrX:31,137,345-33,357,726	AX-42751255
DMD	rs12557076	chrX:31,137,345-33,357,726	AX-42751261
DMD	rs147024633	chrX:31,137,345-33,357,726	AX-69543356
DMD	rs7052181	chrX:31,137,345-33,357,726	AX-11603957
DMD	rs7892505	chrX:31,137,345-33,357,726	AX-11652117
DMD	rs10521982	chrX:31,137,345-33,357,726	AX-16350683
DMD	rs66520172	chrX:31,137,345-33,357,726	AX-37242865
DMD	rs16998240	chrX:31,137,345-33,357,726	AX-11296687
DMD	rs55787823	chrX:31,137,345-33,357,726	AX-37242897
DMD	rs1463495	chrX:31,137,345-33,357,726	AX-16350695
DMD	rs6527132	chrX:31,137,345-33,357,726	AX-11569755
DMD	rs140820221	chrX:31,137,345-33,357,726	AX-38623953
DMD	rs1350477	chrX:31,137,345-33,357,726	AX-12459142
DMD	rs73619021	chrX:31,137,345-33,357,726	AX-37242949
DMD	rs3761604	chrX:31,137,345-33,357,726	AX-12560331
DMD	rs1800273	chrX:31,137,345-33,357,726	AX-11344597
DMD	rs113404060	chrX:31,137,345-33,357,726	AX-38624041
DMD	rs114484622	chrX:31,137,345-33,357,726	AX-38521259
DMD	rs146890759	chrX:31,137,345-33,357,726	AX-38624069
DMD	rs5972470	chrX:31,137,345-33,357,726	AX-11553384
DMD	rs16990005	chrX:31,137,345-33,357,726	AX-11296079
DMD	rs16990008	chrX:31,137,345-33,357,726	AX-42751385
DMD	rs1293908	chrX:31,137,345-33,357,726	AX-11232980
DMD	rs5972472	chrX:31,137,345-33,357,726	AX-11553385
DMD	rs12007475	chrX:31,137,345-33,357,726	AX-42751399
DMD	rs12014251	chrX:31,137,345-33,357,726	AX-42751403
DMD	rs1293891	chrX:31,137,345-33,357,726	AX-11232972
DMD	rs1293905	chrX:31,137,345-33,357,726	AX-42751417
DMD	rs12010294	chrX:31,137,345-33,357,726	AX-11186419
DMD	rs1293906	chrX:31,137,345-33,357,726	AX-11232979
DMD	rs12008458	chrX:31,137,345-33,357,726	AX-42751425
DMD	rs1293875	chrX:31,137,345-33,357,726	AX-37243081
DMD	rs17270765	chrX:31,137,345-33,357,726	AX-42751455
DMD	rs2253300	chrX:31,137,345-33,357,726	AX-11379020
DMD	rs5927924	chrX:31,137,345-33,357,726	AX-11550887
DMD	rs1293924	chrX:31,137,345-33,357,726	AX-16350765
DMD	rs144824129	chrX:31,137,345-33,357,726	AX-38624163
DMD	rs149058642	chrX:31,137,345-33,357,726	AX-38624173
DMD	rs66707838	chrX:31,137,345-33,357,726	AX-16350775

DMD	rs7054546	chrX:31,137,345-33,357,726	AX-16350777
DMD	rs12556642	chrX:31,137,345-33,357,726	AX-37243155
DMD	rs35992741	chrX:31,137,345-33,357,726	AX-37243161
DMD	rs12842666	chrX:31,137,345-33,357,726	AX-11228911
DMD	rs41469547	chrX:31,137,345-33,357,726	AX-42751491
DMD	rs7059099	chrX:31,137,345-33,357,726	AX-11604200
DMD	rs6631472	chrX:31,137,345-33,357,726	AX-11575629
DMD	rs62589974	chrX:31,137,345-33,357,726	AX-37243173
DMD	rs16998256	chrX:31,137,345-33,357,726	AX-11296689
DMD	rs6418637	chrX:31,137,345-33,357,726	AX-11562950
DMD	rs6628673	chrX:31,137,345-33,357,726	AX-42751497
DMD	rs16998260	chrX:31,137,345-33,357,726	AX-11296690
DMD	rs1718043	chrX:31,137,345-33,357,726	AX-11312706
DMD	rs73619026	chrX:31,137,345-33,357,726	AX-37243225
DMD	rs2141753	chrX:31,137,345-33,357,726	AX-11369984
DMD	rs56663344	chrX:31,137,345-33,357,726	AX-37243239
DMD	rs2897164	chrX:31,137,345-33,357,726	AX-11425634
DMD	rs5972488	chrX:31,137,345-33,357,726	AX-11553387
DMD	rs6631486	chrX:31,137,345-33,357,726	AX-11575631
DMD	rs1795578	chrX:31,137,345-33,357,726	AX-11344345
DMD	rs12840034	chrX:31,137,345-33,357,726	AX-11228840
DMD	rs1718039	chrX:31,137,345-33,357,726	AX-11312700
DMD	rs6631492	chrX:31,137,345-33,357,726	AX-11575633
DMD	rs7882019	chrX:31,137,345-33,357,726	AX-11651736
DMD	rs6653578	chrX:31,137,345-33,357,726	AX-12605048
DMD	rs1795571	chrX:31,137,345-33,357,726	AX-16350810
DMD	rs16990111	chrX:31,137,345-33,357,726	AX-11296095
DMD	rs12843752	chrX:31,137,345-33,357,726	AX-50823730
DMD	rs61549924	chrX:31,137,345-33,357,726	AX-37243277
DMD	rs16990116	chrX:31,137,345-33,357,726	AX-11296097
DMD	rs1718041	chrX:31,137,345-33,357,726	AX-11312702
DMD	rs1795592	chrX:31,137,345-33,357,726	AX-11344348
DMD	rs1795595	chrX:31,137,345-33,357,726	AX-11344351
DMD	rs146532983	chrX:31,137,345-33,357,726	AX-38624337
DMD	rs73219215	chrX:31,137,345-33,357,726	AX-37243297
DMD	rs10127060	chrX:31,137,345-33,357,726	AX-11094552
DMD	rs11095222	chrX:31,137,345-33,357,726	AX-11143368
DMD	rs5927938	chrX:31,137,345-33,357,726	AX-11550890
DMD	rs1317098	chrX:31,137,345-33,357,726	AX-11242732
DMD	rs6628677	chrX:31,137,345-33,357,726	AX-12604533
DMD	rs7065982	chrX:31,137,345-33,357,726	AX-11604466
DMD	rs2701794	chrX:31,137,345-33,357,726	AX-11405790
DMD	rs1795579	chrX:31,137,345-33,357,726	AX-11344346
DMD	rs1718053	chrX:31,137,345-33,357,726	AX-11312713
DMD	rs55672607	chrX:31,137,345-33,357,726	AX-37243385
DMD	rs59399365	chrX:31,137,345-33,357,726	AX-37243391
DMD	rs1718047	chrX:31,137,345-33,357,726	AX-11312709
DMD	rs72626020	chrX:31,137,345-33,357,726	AX-37243417
DMD	rs112141433	chrX:31,137,345-33,357,726	AX-16350840
DMD	rs1795588	chrX:31,137,345-33,357,726	AX-11344347
DMD	rs12833101	chrX:31,137,345-33,357,726	AX-11228674
DMD	rs12560135	chrX:31,137,345-33,357,726	AX-11214891
DMD	rs16990140	chrX:31,137,345-33,357,726	AX-11296101
DMD	rs2606664	chrX:31,137,345-33,357,726	AX-11400918
DMD	rs2685908	chrX:31,137,345-33,357,726	AX-11405022

DMD	rs2606683	chrX:31,137,345-33,357,726	AX-11400925
DMD	rs2685909	chrX:31,137,345-33,357,726	AX-11405023
DMD	rs1456734	chrX:31,137,345-33,357,726	AX-11265333
DMD	rs5927942	chrX:31,137,345-33,357,726	AX-12592181
DMD	rs2685891	chrX:31,137,345-33,357,726	AX-11405015
DMD	rs7879462	chrX:31,137,345-33,357,726	AX-11651651
DMD	rs2685892	chrX:31,137,345-33,357,726	AX-12535012
DMD	rs2606667	chrX:31,137,345-33,357,726	AX-11400920
DMD	rs2685893	chrX:31,137,345-33,357,726	AX-37243483
DMD	rs2606668	chrX:31,137,345-33,357,726	AX-37243485
DMD	rs58151710	chrX:31,137,345-33,357,726	AX-37243493
DMD	rs2685896	chrX:31,137,345-33,357,726	AX-11405016
DMD	rs2606672	chrX:31,137,345-33,357,726	AX-11400922
DMD	rs145272492	chrX:31,137,345-33,357,726	AX-38624445
DMD	rs7058785	chrX:31,137,345-33,357,726	AX-37243511
DMD	rs5972504	chrX:31,137,345-33,357,726	AX-11553390
DMD	rs73458061	chrX:31,137,345-33,357,726	AX-37243513
DMD	rs1456737	chrX:31,137,345-33,357,726	AX-42751629
DMD	rs2685902	chrX:31,137,345-33,357,726	AX-11405019
DMD	rs874830	chrX:31,137,345-33,357,726	AX-12653297
DMD	rs2854966	chrX:31,137,345-33,357,726	AX-11419258
DMD	rs55686834	chrX:31,137,345-33,357,726	AX-37243547
DMD	rs331368	chrX:31,137,345-33,357,726	AX-11438214
DMD	rs331362	chrX:31,137,345-33,357,726	AX-37243587
DMD	rs61661964	chrX:31,137,345-33,357,726	AX-37243597
DMD	rs143066970	chrX:31,137,345-33,357,726	AX-38624543
DMD	rs111826558	chrX:31,137,345-33,357,726	AX-38624569
DMD	rs966451	chrX:31,137,345-33,357,726	AX-16350886
DMD	rs5927948	chrX:31,137,345-33,357,726	AX-11550891
DMD	rs1456727	chrX:31,137,345-33,357,726	AX-11265330
DMD	rs1456729	chrX:31,137,345-33,357,726	AX-42751699
DMD	rs331349	chrX:31,137,345-33,357,726	AX-37243705
DMD	rs12400794	chrX:31,137,345-33,357,726	AX-37243731
DMD	rs1160301	chrX:31,137,345-33,357,726	AX-11161773
DMD	rs1408983	chrX:31,137,345-33,357,726	AX-37243775
DMD	rs331331	chrX:31,137,345-33,357,726	AX-11438205
DMD	rs331323	chrX:31,137,345-33,357,726	AX-11438204
DMD	rs62590662	chrX:31,137,345-33,357,726	AX-37243801
DMD	rs331321	chrX:31,137,345-33,357,726	AX-11438202
DMD	rs12007028	chrX:31,137,345-33,357,726	AX-37243817
DMD	rs331318	chrX:31,137,345-33,357,726	AX-11438201
DMD	rs331317	chrX:31,137,345-33,357,726	AX-11438200
DMD	rs331316	chrX:31,137,345-33,357,726	AX-11438199
DMD	rs189117	chrX:31,137,345-33,357,726	AX-11350753
DMD	rs331313	chrX:31,137,345-33,357,726	AX-37243837
DMD	rs115862380	chrX:31,137,345-33,357,726	AX-38521359
DMD	rs5927966	chrX:31,137,345-33,357,726	AX-37243875
DMD	rs143091919	chrX:31,137,345-33,357,726	AX-38624739
DMD	rs2295329	chrX:31,137,345-33,357,726	AX-16350969
DMD	rs1293821	chrX:31,137,345-33,357,726	AX-11232947
DMD	rs5927972	chrX:31,137,345-33,357,726	AX-11550895
DMD	rs5927974	chrX:31,137,345-33,357,726	AX-16350981
DMD	rs1801187	chrX:31,137,345-33,357,726	AX-11344698
DMD	---	chrX:31,137,345-33,357,726	AX-59487755
DMD	rs1963703	chrX:31,137,345-33,357,726	AX-37244003

DMD	rs5972548	chrX:31,137,345-33,357,726	AX-16351002
DMD	rs16990296	chrX:31,137,345-33,357,726	AX-16351017
DMD	rs73619075	chrX:31,137,345-33,357,726	AX-37244131
DMD	rs10521994	chrX:31,137,345-33,357,726	AX-16351052
DMD	rs147474070	chrX:31,137,345-33,357,726	AX-83293608
DMD	rs372444729	chrX:31,137,345-33,357,726	AX-86605601
DMD	rs7053451	chrX:31,137,345-33,357,726	AX-11604001
DMD	rs16990375	chrX:31,137,345-33,357,726	AX-16351089
DMD	rs2405829	chrX:31,137,345-33,357,726	AX-11390020
DMD	rs2076366	chrX:31,137,345-33,357,726	AX-12516630
DMD	rs1800269	chrX:31,137,345-33,357,726	AX-83186220
DMD	rs56027026	chrX:31,137,345-33,357,726	AX-37244275
DMD	rs5972576	chrX:31,137,345-33,357,726	AX-37244281
DMD	rs6527202	chrX:31,137,345-33,357,726	AX-42752017
DMD	rs3827462	chrX:31,137,345-33,357,726	AX-42752033
DMD	rs228390	chrX:31,137,345-33,357,726	AX-12524149
DMD	rs72468667	chrX:31,137,345-33,357,726	AX-83516999
DMD	rs228405	chrX:31,137,345-33,357,726	AX-12524159
DMD	rs228406	chrX:31,137,345-33,357,726	AX-11381582
DMD	rs72468681	chrX:31,137,345-33,357,726	AX-37244385
DMD	rs228312	chrX:31,137,345-33,357,726	AX-42752071
DMD	rs228324	chrX:31,137,345-33,357,726	AX-11381502
DMD	rs228327	chrX:31,137,345-33,357,726	AX-12524111
DMD	rs228336	chrX:31,137,345-33,357,726	AX-12524118
DMD	rs5928002	chrX:31,137,345-33,357,726	AX-37244471
DMD	rs228354	chrX:31,137,345-33,357,726	AX-12524131
DMD	rs73207771	chrX:31,137,345-33,357,726	AX-37244501
DMD	rs228355	chrX:31,137,345-33,357,726	AX-42752159
DMD	rs10126490	chrX:31,137,345-33,357,726	AX-16351169
DMD	rs228378	chrX:31,137,345-33,357,726	AX-11381555
DMD	rs140474271	chrX:31,137,345-33,357,726	AX-38625135
DMD	rs1435733	chrX:31,137,345-33,357,726	AX-11263359
DMD	rs4829123	chrX:31,137,345-33,357,726	AX-11529304
DMD	rs808578	chrX:31,137,345-33,357,726	AX-42752223
DMD	rs808576	chrX:31,137,345-33,357,726	AX-11664668
DMD	rs7057273	chrX:31,137,345-33,357,726	AX-11604133
DMD	rs4829261	chrX:31,137,345-33,357,726	AX-11529323
DMD	rs147822019	chrX:31,137,345-33,357,726	AX-83429594
DMD	rs808573	chrX:31,137,345-33,357,726	AX-12650165
DMD	rs373286166	chrX:31,137,345-33,357,726	AX-86603800
DMD	rs370644567	chrX:31,137,345-33,357,726	AX-86686738
DMD	rs5927083	chrX:31,137,345-33,357,726	AX-12592141
DMD	rs5928027	chrX:31,137,345-33,357,726	AX-16351205
DMD	rs808547	chrX:31,137,345-33,357,726	AX-16351217
DMD	rs140340626	chrX:31,137,345-33,357,726	AX-83138089
DMD	rs808536	chrX:31,137,345-33,357,726	AX-37244701
DMD	rs5928040	chrX:31,137,345-33,357,726	AX-11550903
DMD	rs808520	chrX:31,137,345-33,357,726	AX-11664624
DMD	rs142762831	chrX:31,137,345-33,357,726	AX-38625259
DMD	rs149352277	chrX:31,137,345-33,357,726	AX-38625261
DMD	rs808513	chrX:31,137,345-33,357,726	AX-11664618
DMD	rs57110381	chrX:31,137,345-33,357,726	AX-37244757
DMD	rs5927089	chrX:31,137,345-33,357,726	AX-12592144
DMD	rs5928062	chrX:31,137,345-33,357,726	AX-42752391
DMD	rs1800266	chrX:31,137,345-33,357,726	AX-83006747

DMD	rs57916323	chrX:31,137,345-33,357,726	AX-37244917
DMD	rs112427626	chrX:31,137,345-33,357,726	AX-38625433
DMD	rs5972643	chrX:31,137,345-33,357,726	AX-42752449
DMD	rs5972644	chrX:31,137,345-33,357,726	AX-11553401
DMD	rs5972645	chrX:31,137,345-33,357,726	AX-11553402
DMD	---	chrX:31,137,345-33,357,726	AX-37244987
DMD	rs5928067	chrX:31,137,345-33,357,726	AX-11550906
DMD	rs146155628	chrX:31,137,345-33,357,726	AX-38625483
DMD	rs5928069	chrX:31,137,345-33,357,726	AX-37245029
DMD	---	chrX:31,137,345-33,357,726	AX-86589295
DMD	rs1800265	chrX:31,137,345-33,357,726	AX-42752503
DMD	---	chrX:31,137,345-33,357,726	AX-90035334
DMD	rs141379267	chrX:31,137,345-33,357,726	AX-38625581
DMD	rs6653885	chrX:31,137,345-33,357,726	AX-37245177
DMD	rs12116231	chrX:31,137,345-33,357,726	AX-12428588
DMD	rs2748307	chrX:31,137,345-33,357,726	AX-12536874
DMD	rs2748318	chrX:31,137,345-33,357,726	AX-42752595
DMD	rs2855695	chrX:31,137,345-33,357,726	AX-37245221
DMD	rs5972668	chrX:31,137,345-33,357,726	AX-16351343
DMD	rs5928076	chrX:31,137,345-33,357,726	AX-37245237
DMD	rs141199105	chrX:31,137,345-33,357,726	AX-38625665
DMD	rs2855689	chrX:31,137,345-33,357,726	AX-12541998
DMD	rs7067109	chrX:31,137,345-33,357,726	AX-11604514
DMD	rs112382490	chrX:31,137,345-33,357,726	AX-38625703
DMD	rs5927099	chrX:31,137,345-33,357,726	AX-37245311
DMD	rs2855686	chrX:31,137,345-33,357,726	AX-42752655
DMD	---	chrX:31,137,345-33,357,726	AX-94376502
DMD	rs1158629	chrX:31,137,345-33,357,726	AX-11160471
DMD	---	chrX:31,137,345-33,357,726	AX-94376503
DMD	rs139319218	chrX:31,137,345-33,357,726	AX-38625769
DMD	rs5928080	chrX:31,137,345-33,357,726	AX-11550909
DMD	rs4829269	chrX:31,137,345-33,357,726	AX-51297334
DMD	rs66760061	chrX:31,137,345-33,357,726	AX-37245393
DMD	rs5972684	chrX:31,137,345-33,357,726	AX-12593340
DMD	rs5928089	chrX:31,137,345-33,357,726	AX-11550910
DMD	rs7879662	chrX:31,137,345-33,357,726	AX-11651658
DMD	rs17283421	chrX:31,137,345-33,357,726	AX-16351390
DMD	rs5972690	chrX:31,137,345-33,357,726	AX-11553410
DMD	rs3764763	chrX:31,137,345-33,357,726	AX-11478981
DMD	rs7880016	chrX:31,137,345-33,357,726	AX-11651676
DMD	rs5972699	chrX:31,137,345-33,357,726	AX-12593341
DMD	rs9887516	chrX:31,137,345-33,357,726	AX-11708518
DMD	rs5928095	chrX:31,137,345-33,357,726	AX-11550913
DMD	rs5005249	chrX:31,137,345-33,357,726	AX-16351405
DMD	rs5928099	chrX:31,137,345-33,357,726	AX-16351408
DMD	rs12395380	chrX:31,137,345-33,357,726	AX-11203446
DMD	rs12559198	chrX:31,137,345-33,357,726	AX-11214832
DMD	rs17338877	chrX:31,137,345-33,357,726	AX-12498528
DMD	rs7061782	chrX:31,137,345-33,357,726	AX-11604290
DMD	rs5928102	chrX:31,137,345-33,357,726	AX-11550914
DMD	rs6628748	chrX:31,137,345-33,357,726	AX-11575514
DMD	rs5928104	chrX:31,137,345-33,357,726	AX-12592199
DMD	rs17338898	chrX:31,137,345-33,357,726	AX-16351435
DMD	rs5928109	chrX:31,137,345-33,357,726	AX-16351441
DMD	rs67636309	chrX:31,137,345-33,357,726	AX-37245605

DMD	rs5928111	chrX:31,137,345-33,357,726	AX-11550916
DMD	rs1321394	chrX:31,137,345-33,357,726	AX-11244660
DMD	rs1321395	chrX:31,137,345-33,357,726	AX-12454033
DMD	rs17330215	chrX:31,137,345-33,357,726	AX-37245633
DMD	rs10522017	chrX:31,137,345-33,357,726	AX-11116233
DMD	rs6628752	chrX:31,137,345-33,357,726	AX-42752791
DMD	rs1570029	chrX:31,137,345-33,357,726	AX-12470120
DMD	rs5927113	chrX:31,137,345-33,357,726	AX-11550788
DMD	rs5972715	chrX:31,137,345-33,357,726	AX-37245685
DMD	rs113605304	chrX:31,137,345-33,357,726	AX-16351459
DMD	rs73466863	chrX:31,137,345-33,357,726	AX-37245711
DMD	rs2180648	chrX:31,137,345-33,357,726	AX-11372994
DMD	rs5972721	chrX:31,137,345-33,357,726	AX-16351479
DMD	rs6527243	chrX:31,137,345-33,357,726	AX-12601571
DMD	rs55790705	chrX:31,137,345-33,357,726	AX-37245817
DMD	rs6628755	chrX:31,137,345-33,357,726	AX-16351491
DMD	rs4639663	chrX:31,137,345-33,357,726	AX-11514707
DMD	rs7886739	chrX:31,137,345-33,357,726	AX-16351507
DMD	rs6631712	chrX:31,137,345-33,357,726	AX-16351515
DMD	rs4345727	chrX:31,137,345-33,357,726	AX-11502012
DMD	rs5972737	chrX:31,137,345-33,357,726	AX-11553417
DMD	rs2765379	chrX:31,137,345-33,357,726	AX-11409504
DMD	rs5972739	chrX:31,137,345-33,357,726	AX-37246019
DMD	rs2765381	chrX:31,137,345-33,357,726	AX-11409505
DMD	rs59903175	chrX:31,137,345-33,357,726	AX-37246033
DMD	rs143027254	chrX:31,137,345-33,357,726	AX-38626199
DMD	rs73188288	chrX:31,137,345-33,357,726	AX-37246061
DMD	rs6631726	chrX:31,137,345-33,357,726	AX-11575642
DMD	rs2765387	chrX:31,137,345-33,357,726	AX-16351553
DMD	rs6631731	chrX:31,137,345-33,357,726	AX-12604616
DMD	rs5972743	chrX:31,137,345-33,357,726	AX-42753017
DMD	rs6418647	chrX:31,137,345-33,357,726	AX-11562951
DMD	rs5928167	chrX:31,137,345-33,357,726	AX-11550924
DMD	rs72626075	chrX:31,137,345-33,357,726	AX-16351569
DMD	rs2094147	chrX:31,137,345-33,357,726	AX-42753075
DMD	rs2207086	chrX:31,137,345-33,357,726	AX-37246201
DMD	rs7064140	chrX:31,137,345-33,357,726	AX-16351601
DMD	rs2024614	chrX:31,137,345-33,357,726	AX-11360633
DMD	rs10127390	chrX:31,137,345-33,357,726	AX-37246331
DMD	rs5972769	chrX:31,137,345-33,357,726	AX-11553420
DMD	rs5928193	chrX:31,137,345-33,357,726	AX-11550929
DMD	rs5928201	chrX:31,137,345-33,357,726	AX-16351654
DMD	rs150494081	chrX:31,137,345-33,357,726	AX-38626449
DMD	rs5928207	chrX:31,137,345-33,357,726	AX-42753213
DMD	rs62591018	chrX:31,137,345-33,357,726	AX-37246517
DMD	rs12392440	chrX:31,137,345-33,357,726	AX-11203395
DMD	rs72626080	chrX:31,137,345-33,357,726	AX-37246595
DMD	rs2050074	chrX:31,137,345-33,357,726	AX-11363222
DMD	rs2050076	chrX:31,137,345-33,357,726	AX-37246597
DMD	rs12396164	chrX:31,137,345-33,357,726	AX-11203460
DMD	rs61142109	chrX:31,137,345-33,357,726	AX-37246627
DMD	rs5972802	chrX:31,137,345-33,357,726	AX-11553424
DMD	rs5972805	chrX:31,137,345-33,357,726	AX-11553425
DMD	rs12559939	chrX:31,137,345-33,357,726	AX-42753275
DMD	rs3946125	chrX:31,137,345-33,357,726	AX-42753279

DMD	rs141927233	chrX:31,137,345-33,357,726	AX-38626571
DMD	rs73623943	chrX:31,137,345-33,357,726	AX-16351731
DMD	rs5972815	chrX:31,137,345-33,357,726	AX-11553427
DMD	rs17341316	chrX:31,137,345-33,357,726	AX-11320333
DMD	rs58029094	chrX:31,137,345-33,357,726	AX-16351753
DSC2	rs200056085	chr18:28,645,942-28,682,388	AX-83241216
DSC2	---	chr18:28,645,942-28,682,388	AX-94364075
DSC2	rs143342988	chr18:28,645,942-28,682,388	AX-83035166
DSC2	rs141873745	chr18:28,645,942-28,682,388	AX-92009411
DSC2	rs147109895	chr18:28,645,942-28,682,388	AX-83116253
DSC2	---	chr18:28,645,942-28,682,388	AX-90029836
DSC2	rs370325533	chr18:28,645,942-28,682,388	AX-91989192
DSC2	rs142410803	chr18:28,645,942-28,682,388	AX-94350052
DSC2	---	chr18:28,645,942-28,682,388	AX-94362747
DSC2	rs143413607	chr18:28,645,942-28,682,388	AX-83417692
DSC2	rs200197897	chr18:28,645,942-28,682,388	AX-94350053
DSC2	---	chr18:28,645,942-28,682,388	AX-37822253
DSC2	---	chr18:28,645,942-28,682,388	AX-94376966
DSC2	rs1617629	chr18:28,645,942-28,682,388	AX-92010094
DSC2	---	chr18:28,645,942-28,682,388	AX-94376967
DSC2	rs377272752	chr18:28,645,942-28,682,388	AX-83310104
DSC2	---	chr18:28,645,942-28,682,388	AX-91997384
DSC2	---	chr18:28,645,942-28,682,388	AX-91998086
DSC2	rs1893963	chr18:28,645,942-28,682,388	AX-11351031
DSC2	rs549251334	chr18:28,645,942-28,682,388	AX-94376968
DSC2	rs151024019	chr18:28,645,942-28,682,388	AX-82948638
DSC2	---	chr18:28,645,942-28,682,388	AX-94376969
DSC2	---	chr18:28,645,942-28,682,388	AX-90077026
DSC2	rs147742157	chr18:28,645,942-28,682,388	AX-83118879
DSC2	rs4799304	chr18:28,645,942-28,682,388	AX-40291185
DSC2	rs397514043	chr18:28,645,942-28,682,388	AX-90076806
DSC2	---	chr18:28,645,942-28,682,388	AX-94376970
DSC2	---	chr18:28,645,942-28,682,388	AX-90062073
DSC2	---	chr18:28,645,942-28,682,388	AX-90060810
DSC2	rs148185335	chr18:28,645,942-28,682,388	AX-83362316
DSC2	rs140232809	chr18:28,645,942-28,682,388	AX-83411190
DSC2	rs201845641	chr18:28,645,942-28,682,388	AX-83136981
DSC2	rs150318400	chr18:28,645,942-28,682,388	AX-83595186
DSC2	rs10084050	chr18:28,645,942-28,682,388	AX-11091635
DSC2	---	chr18:28,645,942-28,682,388	AX-94379498
DSC2	rs397514041	chr18:28,645,942-28,682,388	AX-90071521
DSC2	rs144242114	chr18:28,645,942-28,682,388	AX-92010028
DSC2	---	chr18:28,645,942-28,682,388	AX-94362749
DSC2	---	chr18:28,645,942-28,682,388	AX-94379500
DSC2	---	chr18:28,645,942-28,682,388	AX-94362751
DSC2	---	chr18:28,645,942-28,682,388	AX-94379502
DSC2	---	chr18:28,645,942-28,682,388	AX-90078050
DSC2	rs139399951	chr18:28,645,942-28,682,388	AX-83387887
DSC2	---	chr18:28,645,942-28,682,388	AX-90026250
DSC2	rs368299411	chr18:28,645,942-28,682,388	AX-86659072
DSC2	rs35717505	chr18:28,645,942-28,682,388	AX-83367721
DSC2	---	chr18:28,645,942-28,682,388	AX-94379503
DSC2	rs145560678	chr18:28,645,942-28,682,388	AX-82986464
DSC2	---	chr18:28,645,942-28,682,388	AX-94379505
DSC2	rs200802591	chr18:28,645,942-28,682,388	AX-92007862

DSC2	---	chr18:28,645,942-28,682,388	AX-90026251
DSC2	rs373201722	chr18:28,645,942-28,682,388	AX-94379506
DSC2	---	chr18:28,645,942-28,682,388	AX-90048252
DSC2	---	chr18:28,645,942-28,682,388	AX-94379507
DSC2	rs397514042	chr18:28,645,942-28,682,388	AX-90034283
DSC2	---	chr18:28,645,942-28,682,388	AX-94362758
DSC2	---	chr18:28,645,942-28,682,388	AX-90029043
DSC2	rs142331975	chr18:28,645,942-28,682,388	AX-88754523
DSC2	---	chr18:28,645,942-28,682,388	AX-94362759
DSC2	---	chr18:28,645,942-28,682,388	AX-90026253
DSC2	---	chr18:28,645,942-28,682,388	AX-86555619
DSC2	rs373305929	chr18:28,645,942-28,682,388	AX-91983978
DSC2	rs144799937	chr18:28,645,942-28,682,388	AX-82978325
DSC2	---	chr18:28,645,942-28,682,388	AX-94379510
DSC2	rs138749562	chr18:28,645,942-28,682,388	AX-83287256
DSC2	rs180908546	chr18:28,645,942-28,682,388	AX-83249840
DSC2	---	chr18:28,645,942-28,682,388	AX-94380933
DSC2	rs12954874	chr18:28,645,942-28,682,388	AX-12449745
DSC2	rs397517408	chr18:28,645,942-28,682,388	AX-90080326
DSC2	rs17799852	chr18:28,645,942-28,682,388	AX-11340932
DSC2	---	chr18:28,645,942-28,682,388	AX-94362761
DSC2	---	chr18:28,645,942-28,682,388	AX-90060259
DSG2	---	chr18:29,078,027-29,128,814	AX-90055676
DSG2	rs368809971	chr18:29,078,027-29,128,814	AX-92015941
DSG2	rs372174546	chr18:29,078,027-29,128,814	AX-91992207
DSG2	rs117873584	chr18:29,078,027-29,128,814	AX-13286717
DSG2	rs1460602	chr18:29,078,027-29,128,814	AX-40292119
DSG2	rs8093731	chr18:29,078,027-29,128,814	AX-11665253
DSG2	---	chr18:29,078,027-29,128,814	AX-94379512
DSG2	---	chr18:29,078,027-29,128,814	AX-90055677
DSG2	rs121913008	chr18:29,078,027-29,128,814	AX-88797546
DSG2	rs121913006	chr18:29,078,027-29,128,814	AX-88797544
DSG2	---	chr18:29,078,027-29,128,814	AX-94362763
DSG2	---	chr18:29,078,027-29,128,814	AX-90030628
DSG2	rs113451409	chr18:29,078,027-29,128,814	AX-83112681
DSG2	---	chr18:29,078,027-29,128,814	AX-90045229
DSG2	---	chr18:29,078,027-29,128,814	AX-94380934
DSG2	rs397516709	chr18:29,078,027-29,128,814	AX-90036087
DSG2	---	chr18:29,078,027-29,128,814	AX-94362764
DSG2	---	chr18:29,078,027-29,128,814	AX-94379515
DSG2	---	chr18:29,078,027-29,128,814	AX-90026798
DSG2	---	chr18:29,078,027-29,128,814	AX-90033474
DSG2	---	chr18:29,078,027-29,128,814	AX-90028115
DSG2	rs72938894	chr18:29,078,027-29,128,814	AX-13286783
DSG2	rs200997703	chr18:29,078,027-29,128,814	AX-83042470
DSG2	rs369869320	chr18:29,078,027-29,128,814	AX-91991603
DSG2	---	chr18:29,078,027-29,128,814	AX-94379516
DSG2	rs121913011	chr18:29,078,027-29,128,814	AX-88754503
DSG2	---	chr18:29,078,027-29,128,814	AX-94362767
DSG2	---	chr18:29,078,027-29,128,814	AX-90074427
DSG2	---	chr18:29,078,027-29,128,814	AX-90080849
DSG2	---	chr18:29,078,027-29,128,814	AX-94364076
DSG2	rs62095193	chr18:29,078,027-29,128,814	AX-91983016
DSG2	rs2230233	chr18:29,078,027-29,128,814	AX-12521924
DSG2	---	chr18:29,078,027-29,128,814	AX-94360221

DSG2	---	chr18:29,078,027-29,128,814	AX-11376787
DSG2	---	chr18:29,078,027-29,128,814	AX-90049768
DSG2	---	chr18:29,078,027-29,128,814	AX-90058651
DSG2	---	chr18:29,078,027-29,128,814	AX-90055684
DSG2	---	chr18:29,078,027-29,128,814	AX-94360222
DSG2	rs121913007	chr18:29,078,027-29,128,814	AX-88754500
DSG2	rs201040643	chr18:29,078,027-29,128,814	AX-83098438
DSG2	---	chr18:29,078,027-29,128,814	AX-90055685
DSG2	---	chr18:29,078,027-29,128,814	AX-90063195
DSG2	rs121913012	chr18:29,078,027-29,128,814	AX-88797549
DSG2	---	chr18:29,078,027-29,128,814	AX-94360223
DSG2	rs191564916	chr18:29,078,027-29,128,814	AX-83429278
DSG2	rs2704041	chr18:29,078,027-29,128,814	AX-13286797
DSG2	rs1941938	chr18:29,078,027-29,128,814	AX-13286804
DSG2	---	chr18:29,078,027-29,128,814	AX-91984008
DSG2	rs139326669	chr18:29,078,027-29,128,814	AX-83030529
DSG2	---	chr18:29,078,027-29,128,814	AX-94356320
DSG2	rs369214871	chr18:29,078,027-29,128,814	AX-94373071
DSG2	rs193922639	chr18:29,078,027-29,128,814	AX-83218771
DSG2	---	chr18:29,078,027-29,128,814	AX-90077630
DSG2	---	chr18:29,078,027-29,128,814	AX-94363960
DSG2	rs74699553	chr18:29,078,027-29,128,814	AX-32423527
DSG2	rs116675326	chr18:29,078,027-29,128,814	AX-37822465
DSG2	rs370509593	chr18:29,078,027-29,128,814	AX-94373072
DSG2	---	chr18:29,078,027-29,128,814	AX-94356323
DSG2	---	chr18:29,078,027-29,128,814	AX-90028108
DSG2	---	chr18:29,078,027-29,128,814	AX-94373074
DSG2	rs375679311	chr18:29,078,027-29,128,814	AX-91997174
DSG2	---	chr18:29,078,027-29,128,814	AX-94373075
DSG2	---	chr18:29,078,027-29,128,814	AX-94356326
DSG2	rs121913009	chr18:29,078,027-29,128,814	AX-88797547
DSG2	rs2230235	chr18:29,078,027-29,128,814	AX-40292159
DSG2	rs200509948	chr18:29,078,027-29,128,814	AX-82983150
DSG2	---	chr18:29,078,027-29,128,814	AX-86715751
DSG2	---	chr18:29,078,027-29,128,814	AX-90026801
DSG2	---	chr18:29,078,027-29,128,814	AX-90026802
DSG2	---	chr18:29,078,027-29,128,814	AX-86614126
DSG2	rs199681901	chr18:29,078,027-29,128,814	AX-83349431
DSG2	---	chr18:29,078,027-29,128,814	AX-94356327
DSG2	rs368257724	chr18:29,078,027-29,128,814	AX-92016681
DSG2	rs375527314	chr18:29,078,027-29,128,814	AX-94373078
DSG2	rs35743180	chr18:29,078,027-29,128,814	AX-32423553
DSG2	rs16962075	chr18:29,078,027-29,128,814	AX-13286824
DSG2	rs397514038	chr18:29,078,027-29,128,814	AX-90028054
DSG2	rs201564919	chr18:29,078,027-29,128,814	AX-82970993
DSG2	---	chr18:29,078,027-29,128,814	AX-90042975
DSG2	---	chr18:29,078,027-29,128,814	AX-90074695
DSG2	---	chr18:29,078,027-29,128,814	AX-94363961
DSG2	rs141388237	chr18:29,078,027-29,128,814	AX-94366807
DSG2	rs79241126	chr18:29,078,027-29,128,814	AX-37822467
DSG2	rs202063433	chr18:29,078,027-29,128,814	AX-94366808
DSG2	---	chr18:29,078,027-29,128,814	AX-94373079
DSG2	rs2278792	chr18:29,078,027-29,128,814	AX-83039959
DSG2	---	chr18:29,078,027-29,128,814	AX-94356330
DSG2	rs114544564	chr18:29,078,027-29,128,814	AX-63156066

DSG2	---	chr18:29,078,027-29,128,814	AX-90026804
DSG2	---	chr18:29,078,027-29,128,814	AX-90026805
DSG2	---	chr18:29,078,027-29,128,814	AX-94357517
DSG2	rs1042769	chr18:29,078,027-29,128,814	AX-11106355
DSG2	rs16962093	chr18:29,078,027-29,128,814	AX-40292183
DSG2	rs370316475	chr18:29,078,027-29,128,814	AX-94373081
DSG2	rs34417028	chr18:29,078,027-29,128,814	AX-83245992
DSG2	rs371498622	chr18:29,078,027-29,128,814	AX-92016870
DSG2	---	chr18:29,078,027-29,128,814	AX-94356332
DSG2	---	chr18:29,078,027-29,128,814	AX-94373083
DSG2	rs142841727	chr18:29,078,027-29,128,814	AX-83454748
DSG2	---	chr18:29,078,027-29,128,814	AX-90028913
DSG2	rs146402368	chr18:29,078,027-29,128,814	AX-94366809
DSG2	rs373055076	chr18:29,078,027-29,128,814	AX-91981324
DSG2	---	chr18:29,078,027-29,128,814	AX-94373084
DSG2	---	chr18:29,078,027-29,128,814	AX-94373085
DSG2	---	chr18:29,078,027-29,128,814	AX-90079441
DSG2	---	chr18:29,078,027-29,128,814	AX-94363962
DSG2	rs200830807	chr18:29,078,027-29,128,814	AX-91990430
DSG2	---	chr18:29,078,027-29,128,814	AX-94380712
DSG2	rs8095704	chr18:29,078,027-29,128,814	AX-56740029
DSG2	rs397516707	chr18:29,078,027-29,128,814	AX-91977748
DSG2	---	chr18:29,078,027-29,128,814	AX-94373086
DSG2	rs201786158	chr18:29,078,027-29,128,814	AX-86581709
DSG2	---	chr18:29,078,027-29,128,814	AX-94356337
DSG2	rs149617776	chr18:29,078,027-29,128,814	AX-82964339
DSG2	---	chr18:29,078,027-29,128,814	AX-90061230
DSG2	rs79068489	chr18:29,078,027-29,128,814	AX-63156073
DSG2	---	chr18:29,078,027-29,128,814	AX-94347793
DSG2	rs1791235	chr18:29,078,027-29,128,814	AX-11344096
DSP	rs71559180	chr6:7,541,870-7,586,946	AX-88756407
DSP	rs121912998	chr6:7,541,870-7,586,946	AX-83545517
DSP	rs77445784	chr6:7,541,870-7,586,946	AX-92014316
DSP	rs36087964	chr6:7,541,870-7,586,946	AX-15441508
DSP	rs140403872	chr6:7,541,870-7,586,946	AX-92011841
DSP	rs2294454	chr6:7,541,870-7,586,946	AX-35880593
DSP	rs3823183	chr6:7,541,870-7,586,946	AX-35880621
DSP	rs7743265	chr6:7,541,870-7,586,946	AX-42028857
DSP	rs2744365	chr6:7,541,870-7,586,946	AX-11408647
DSP	rs140965835	chr6:7,541,870-7,586,946	AX-83484318
DSP	rs188516326	chr6:7,541,870-7,586,946	AX-83303821
DSP	rs200473206	chr6:7,541,870-7,586,946	AX-86656640
DSP	rs10484325	chr6:7,541,870-7,586,946	AX-11109241
DSP	rs28763958	chr6:7,541,870-7,586,946	AX-11423614
DSP	---	chr6:7,541,870-7,586,946	AX-90033555
DSP	---	chr6:7,541,870-7,586,946	AX-94361831
DSP	---	chr6:7,541,870-7,586,946	AX-90025615
DSP	---	chr6:7,541,870-7,586,946	AX-94360227
DSP	rs147315869	chr6:7,541,870-7,586,946	AX-83104694
DSP	rs397516955	chr6:7,541,870-7,586,946	AX-90054501
DSP	rs2744376	chr6:7,541,870-7,586,946	AX-11408649
DSP	---	chr6:7,541,870-7,586,946	AX-94360228
DSP	---	chr6:7,541,870-7,586,946	AX-90045731
DSP	---	chr6:7,541,870-7,586,946	AX-94360229
DSP	rs28521706	chr6:7,541,870-7,586,946	AX-94348604

DSP	---	chr6:7,541,870-7,586,946	AX-90025617
DSP	---	chr6:7,541,870-7,586,946	AX-90063113
DSP	rs3778337	chr6:7,541,870-7,586,946	AX-11479999
DSP	---	chr6:7,541,870-7,586,946	AX-90078416
DSP	---	chr6:7,541,870-7,586,946	AX-94364080
DSP	rs121912991	chr6:7,541,870-7,586,946	AX-90042190
DSP	---	chr6:7,541,870-7,586,946	AX-94380830
DSP	---	chr6:7,541,870-7,586,946	AX-94376980
DSP	---	chr6:7,541,870-7,586,946	AX-90061977
DSP	---	chr6:7,541,870-7,586,946	AX-94360231
DSP	---	chr6:7,541,870-7,586,946	AX-94376982
DSP	rs150422458	chr6:7,541,870-7,586,946	AX-91994253
DSP	---	chr6:7,541,870-7,586,946	AX-94360233
DSP	rs397516914	chr6:7,541,870-7,586,946	AX-91983369
DSP	---	chr6:7,541,870-7,586,946	AX-90054503
DSP	---	chr6:7,541,870-7,586,946	AX-94376984
DSP	---	chr6:7,541,870-7,586,946	AX-94360235
DSP	---	chr6:7,541,870-7,586,946	AX-90032391
DSP	---	chr6:7,541,870-7,586,946	AX-90025619
DSP	rs34543842	chr6:7,541,870-7,586,946	AX-94363898
DSP	rs28763961	chr6:7,541,870-7,586,946	AX-35881649
DSP	rs35820473	chr6:7,541,870-7,586,946	AX-91986597
DSP	---	chr6:7,541,870-7,586,946	AX-90025620
DSP	---	chr6:7,541,870-7,586,946	AX-94360236
DSP	---	chr6:7,541,870-7,586,946	AX-90029769
DSP	rs148147581	chr6:7,541,870-7,586,946	AX-83125345
DSP	---	chr6:7,541,870-7,586,946	AX-90071973
DSP	---	chr6:7,541,870-7,586,946	AX-94363968
DSP	rs34239595	chr6:7,541,870-7,586,946	AX-83527226
DSP	---	chr6:7,541,870-7,586,946	AX-90025621
DSP	---	chr6:7,541,870-7,586,946	AX-94356342
DSP	---	chr6:7,541,870-7,586,946	AX-94356343
DSP	---	chr6:7,541,870-7,586,946	AX-90028176
DSP	---	chr6:7,541,870-7,586,946	AX-94356344
DSP	rs121912995	chr6:7,541,870-7,586,946	AX-90071078
DSP	---	chr6:7,541,870-7,586,946	AX-90037148
DSP	rs2076304	chr6:7,541,870-7,586,946	AX-42029245
DSP	---	chr6:7,541,870-7,586,946	AX-94373095
DSP	---	chr6:7,541,870-7,586,946	AX-90037335
DSP	rs28763963	chr6:7,541,870-7,586,946	AX-94348606
DSP	rs139071827	chr6:7,541,870-7,586,946	AX-92010164
DSP	rs150339369	chr6:7,541,870-7,586,946	AX-83138871
DSP	rs372906646	chr6:7,541,870-7,586,946	AX-94356346
DSP	rs121912994	chr6:7,541,870-7,586,946	AX-90071077
DSP	---	chr6:7,541,870-7,586,946	AX-94379530
DSP	---	chr6:7,541,870-7,586,946	AX-94379531
DSP	---	chr6:7,541,870-7,586,946	AX-90073366
DSP	---	chr6:7,541,870-7,586,946	AX-94364188
DSP	---	chr6:7,541,870-7,586,946	AX-94379532
DSP	---	chr6:7,541,870-7,586,946	AX-94362783
DSP	---	chr6:7,541,870-7,586,946	AX-94364189
DSP	rs1016835	chr6:7,541,870-7,586,946	AX-15442436
DSP	---	chr6:7,541,870-7,586,946	AX-94379534
DSP	rs146407262	chr6:7,541,870-7,586,946	AX-15442437
DSP	rs375891215	chr6:7,541,870-7,586,946	AX-91999040

DSP	---	chr6:7,541,870-7,586,946	AX-94362785
DSP	rs142494121	chr6:7,541,870-7,586,946	AX-83448715
DSP	---	chr6:7,541,870-7,586,946	AX-90072105
DSP	---	chr6:7,541,870-7,586,946	AX-94364190
DSP	rs80325569	chr6:7,541,870-7,586,946	AX-15442448
DSP	---	chr6:7,541,870-7,586,946	AX-90027780
DSP	rs2064217	chr6:7,541,870-7,586,946	AX-15442452
DSP	rs190421916	chr6:7,541,870-7,586,946	AX-94368169
DSP	rs397516928	chr6:7,541,870-7,586,946	AX-91995716
DSP	---	chr6:7,541,870-7,586,946	AX-90080782
DSP	rs397516929	chr6:7,541,870-7,586,946	AX-90036528
DSP	---	chr6:7,541,870-7,586,946	AX-90046552
DSP	---	chr6:7,541,870-7,586,946	AX-94380940
DSP	---	chr6:7,541,870-7,586,946	AX-90080600
DSP	---	chr6:7,541,870-7,586,946	AX-94364192
DSP	---	chr6:7,541,870-7,586,946	AX-91978296
DSP	rs372440854	chr6:7,541,870-7,586,946	AX-91978458
DSP	---	chr6:7,541,870-7,586,946	AX-91996956
DSP	---	chr6:7,541,870-7,586,946	AX-90054507
DSP	rs41302881	chr6:7,541,870-7,586,946	AX-94365360
DSP	rs28763964	chr6:7,541,870-7,586,946	AX-35881979
DSP	---	chr6:7,541,870-7,586,946	AX-94379536
DSP	---	chr6:7,541,870-7,586,946	AX-94361832
DSP	rs535202724	chr6:7,541,870-7,586,946	AX-94361833
DSP	---	chr6:7,541,870-7,586,946	AX-90054508
DSP	---	chr6:7,541,870-7,586,946	AX-90027914
DSP	rs121912997	chr6:7,541,870-7,586,946	AX-90071080
DSP	---	chr6:7,541,870-7,586,946	AX-94379537
DSP	---	chr6:7,541,870-7,586,946	AX-90065543
DSP	---	chr6:7,541,870-7,586,946	AX-83570239
DSP	---	chr6:7,541,870-7,586,946	AX-94364193
DSP	rs41302883	chr6:7,541,870-7,586,946	AX-94348610
DSP	---	chr6:7,541,870-7,586,946	AX-94364194
DSP	---	chr6:7,541,870-7,586,946	AX-94362788
DSP	---	chr6:7,541,870-7,586,946	AX-94364195
DSP	rs148478829	chr6:7,541,870-7,586,946	AX-92012902
DSP	rs200745877	chr6:7,541,870-7,586,946	AX-83304309
DSP	rs75537269	chr6:7,541,870-7,586,946	AX-83464051
DSP	---	chr6:7,541,870-7,586,946	AX-90034758
DSP	---	chr6:7,541,870-7,586,946	AX-83532255
DSP	rs140029036	chr6:7,541,870-7,586,946	AX-92013669
DSP	---	chr6:7,541,870-7,586,946	AX-94379539
DSP	rs148041814	chr6:7,541,870-7,586,946	AX-83310860
DSP	rs375919492	chr6:7,541,870-7,586,946	AX-91989668
DSP	rs2076299	chr6:7,541,870-7,586,946	AX-11365962
DSP	rs375150075	chr6:7,541,870-7,586,946	AX-92001803
DSP	rs28763966	chr6:7,541,870-7,586,946	AX-42029347
DSP	rs141227126	chr6:7,541,870-7,586,946	AX-83301791
DSP	---	chr6:7,541,870-7,586,946	AX-94362790
DSP	rs28763967	chr6:7,541,870-7,586,946	AX-11423616
DSP	rs28763968	chr6:7,541,870-7,586,946	AX-11423617
DSP	rs200421954	chr6:7,541,870-7,586,946	AX-83313067
DSP	---	chr6:7,541,870-7,586,946	AX-90058880
DSP	rs200243976	chr6:7,541,870-7,586,946	AX-94368170
DSP	---	chr6:7,541,870-7,586,946	AX-90025624

DSP	---	chr6:7,541,870-7,586,946	AX-94379541
DSP	rs142803672	chr6:7,541,870-7,586,946	AX-83570959
DSP	rs147415451	chr6:7,541,870-7,586,946	AX-83057070
DSP	---	chr6:7,541,870-7,586,946	AX-94362792
DSP	---	chr6:7,541,870-7,586,946	AX-90079683
DSP	rs6929069	chr6:7,541,870-7,586,946	AX-11595086
DSP	rs142885240	chr6:7,541,870-7,586,946	AX-83338834
DSP	rs376186141	chr6:7,541,870-7,586,946	AX-91980345
DSP	rs397516946	chr6:7,541,870-7,586,946	AX-92011042
DSP	---	chr6:7,541,870-7,586,946	AX-94371725
DSP	rs377715841	chr6:7,541,870-7,586,946	AX-86731812
DSP	rs562015789	chr6:7,541,870-7,586,946	AX-94371726
DSP	---	chr6:7,541,870-7,586,946	AX-90054510
DSP	rs144392839	chr6:7,541,870-7,586,946	AX-83546477
DSP	rs121912996	chr6:7,541,870-7,586,946	AX-90071079
DSP	---	chr6:7,541,870-7,586,946	AX-94380659
DSP	---	chr6:7,541,870-7,586,946	AX-92015551
DSP	---	chr6:7,541,870-7,586,946	AX-90081112
DSP	---	chr6:7,541,870-7,586,946	AX-90025626
DSP	rs41302885	chr6:7,541,870-7,586,946	AX-50506002
DSP	rs144263721	chr6:7,541,870-7,586,946	AX-83250868
DSP	---	chr6:7,541,870-7,586,946	AX-94371728
DSP	rs28763969	chr6:7,541,870-7,586,946	AX-11423618
DSP	---	chr6:7,541,870-7,586,946	AX-94371729
DSP	rs34476546	chr6:7,541,870-7,586,946	AX-94380651
DSP	rs397516953	chr6:7,541,870-7,586,946	AX-92008157
DSP	rs149070106	chr6:7,541,870-7,586,946	AX-92007671
DSP	---	chr6:7,541,870-7,586,946	AX-83523868
DSP	---	chr6:7,541,870-7,586,946	AX-90054512
DSP	---	chr6:7,541,870-7,586,946	AX-83191772
DSP	rs34238014	chr6:7,541,870-7,586,946	AX-94363892
DSP	---	chr6:7,541,870-7,586,946	AX-90054513
DSP	rs34334797	chr6:7,541,870-7,586,946	AX-94363907
DSP	rs28931610	chr6:7,541,870-7,586,946	AX-90066946
DSP	rs387906618	chr6:7,541,870-7,586,946	AX-90033565
DSP	rs2076300	chr6:7,541,870-7,586,946	AX-15442621
DSP	---	chr6:7,541,870-7,586,946	AX-90048484
DSP	rs78843072	chr6:7,541,870-7,586,946	AX-92015274
DSP	---	chr6:7,541,870-7,586,946	AX-90051633
DSP	---	chr6:7,541,870-7,586,946	AX-94380660
DSP	---	chr6:7,541,870-7,586,946	AX-94354980
DSP	rs142078450	chr6:7,541,870-7,586,946	AX-83345048
DSP	---	chr6:7,541,870-7,586,946	AX-90047474
DSP	---	chr6:7,541,870-7,586,946	AX-94357852
DSP	rs371129517	chr6:7,541,870-7,586,946	AX-91983157
DSP	---	chr6:7,541,870-7,586,946	AX-90025629
DSP	---	chr6:7,541,870-7,586,946	AX-94354981
DSP	rs116888866	chr6:7,541,870-7,586,946	AX-15442634
DSP	rs35379048	chr6:7,541,870-7,586,946	AX-11462949
DSP	---	chr6:7,541,870-7,586,946	AX-90025630
DSP	rs144275591	chr6:7,541,870-7,586,946	AX-91980898
DSP	---	chr6:7,541,870-7,586,946	AX-94371732
DSP	---	chr6:7,541,870-7,586,946	AX-94371733
DSP	---	chr6:7,541,870-7,586,946	AX-94354984
DSP	---	chr6:7,541,870-7,586,946	AX-35882151

DSP	rs201397978	chr6:7,541,870-7,586,946	AX-94354080
DSP	---	chr6:7,541,870-7,586,946	AX-94371735
DSP	---	chr6:7,541,870-7,586,946	AX-90079854
DSP	---	chr6:7,541,870-7,586,946	AX-94380662
DSP	rs34884895	chr6:7,541,870-7,586,946	AX-83505480
DSP	---	chr6:7,541,870-7,586,946	AX-94354986
DSP	rs397516964	chr6:7,541,870-7,586,946	AX-92009212
DSP	rs377148997	chr6:7,541,870-7,586,946	AX-94354987
DSP	rs138329459	chr6:7,541,870-7,586,946	AX-83481875
DSP	rs201826850	chr6:7,541,870-7,586,946	AX-94354083
DSP	rs2744380	chr6:7,541,870-7,586,946	AX-15442638
DSP	---	chr6:7,541,870-7,586,946	AX-94354988
DSP	rs121912999	chr6:7,541,870-7,586,946	AX-88756898
DSP	---	chr6:7,541,870-7,586,946	AX-83352285
DSP	rs374137476	chr6:7,541,870-7,586,946	AX-94354989
DSP	rs397516972	chr6:7,541,870-7,586,946	AX-91976947
DSP	rs28763971	chr6:7,541,870-7,586,946	AX-11423619
DSP	rs11558732	chr6:7,541,870-7,586,946	AX-84785970
ELN	rs4717865	chr7:73,442,427-73,484,236	AX-42245581
ELN	rs145612009	chr7:73,442,427-73,484,236	AX-83535722
ELN	---	chr7:73,442,427-73,484,236	AX-90050524
ELN	rs2071307	chr7:73,442,427-73,484,236	AX-11365338
ELN	---	chr7:73,442,427-73,484,236	AX-86565231
ELN	rs2856728	chr7:73,442,427-73,484,236	AFFX-KIT-000161
ELN	rs17855988	chr7:73,442,427-73,484,236	AX-82904042
ELN	---	chr7:73,442,427-73,484,236	AX-94379929
ELN	rs41511151	chr7:73,442,427-73,484,236	AX-83369784
ELN	rs8326	chr7:73,442,427-73,484,236	AX-36311377
EMD	---	chrX:153,607,597-153,609,883	AX-90073408
EYA4	rs117683183	chr6:133,561,736-133,853,258	AX-67483179
EYA4	rs533729	chr6:133,561,736-133,853,258	AX-15245245
EYA4	rs111288063	chr6:133,561,736-133,853,258	AX-15245281
EYA4	rs11154720	chr6:133,561,736-133,853,258	AX-11147536
EYA4	rs2145348	chr6:133,561,736-133,853,258	AX-15245293
EYA4	rs211432	chr6:133,561,736-133,853,258	AX-12517663
EYA4	rs62430314	chr6:133,561,736-133,853,258	AX-15245335
EYA4	rs9373047	chr6:133,561,736-133,853,258	AX-35561619
EYA4	rs950374	chr6:133,561,736-133,853,258	AX-12661813
EYA4	rs11757185	chr6:133,561,736-133,853,258	AX-12418655
EYA4	rs112430404	chr6:133,561,736-133,853,258	AX-38319533
EYA4	rs77564299	chr6:133,561,736-133,853,258	AX-15245424
EYA4	rs12201595	chr6:133,561,736-133,853,258	AX-41853649
EYA4	rs34353709	chr6:133,561,736-133,853,258	AX-41853685
EYA4	rs11753937	chr6:133,561,736-133,853,258	AX-11172939
EYA4	rs9402503	chr6:133,561,736-133,853,258	AX-35561755
EYA4	rs4895961	chr6:133,561,736-133,853,258	AX-11534338
EYA4	rs9399061	chr6:133,561,736-133,853,258	AX-15245512
EYA4	rs77910661	chr6:133,561,736-133,853,258	AX-38319559
EYA4	rs2031596	chr6:133,561,736-133,853,258	AX-11361340
EYA4	rs17062411	chr6:133,561,736-133,853,258	AX-11302385
EYA4	rs7760570	chr6:133,561,736-133,853,258	AX-11643730
EYA4	rs159418	chr6:133,561,736-133,853,258	AX-12470822
EYA4	rs465147	chr6:133,561,736-133,853,258	AX-15245640
EYA4	rs34512762	chr6:133,561,736-133,853,258	AX-11448241
EYA4	rs117503067	chr6:133,561,736-133,853,258	AX-38319577

EYA4	rs17062478	chr6:133,561,736-133,853,258	AX-11302391
EYA4	rs1014980	chr6:133,561,736-133,853,258	AX-12383164
EYA4	rs212788	chr6:133,561,736-133,853,258	AX-12518154
EYA4	rs150462	chr6:133,561,736-133,853,258	AX-35562001
EYA4	rs13205584	chr6:133,561,736-133,853,258	AX-11244293
EYA4	rs380367	chr6:133,561,736-133,853,258	AX-15245779
EYA4	rs79709397	chr6:133,561,736-133,853,258	AX-15245797
EYA4	rs3777864	chr6:133,561,736-133,853,258	AX-15245811
EYA4	rs73544945	chr6:133,561,736-133,853,258	AX-15245825
EYA4	rs73544950	chr6:133,561,736-133,853,258	AX-15245836
EYA4	rs9493627	chr6:133,561,736-133,853,258	AX-11689931
EYA4	rs41286200	chr6:133,561,736-133,853,258	AX-11492697
EYA4	rs212764	chr6:133,561,736-133,853,258	AX-11369057
EYA4	rs3777866	chr6:133,561,736-133,853,258	AX-15245853
EYA4	rs62429425	chr6:133,561,736-133,853,258	AX-15245866
EYA4	rs35762882	chr6:133,561,736-133,853,258	AX-12557383
EYA4	rs144415484	chr6:133,561,736-133,853,258	AX-83017592
EYA4	rs6905892	chr6:133,561,736-133,853,258	AX-41853983
EYA4	rs3777868	chr6:133,561,736-133,853,258	AX-15245889
EYA4	rs1883388	chr6:133,561,736-133,853,258	AX-11349867
EYA4	rs56253925	chr6:133,561,736-133,853,258	AX-35562229
EYA4	rs2277083	chr6:133,561,736-133,853,258	AX-15245944
EYA4	rs79506917	chr6:133,561,736-133,853,258	AX-15245950
EYA4	rs56208430	chr6:133,561,736-133,853,258	AX-35562241
EYA4	---	chr6:133,561,736-133,853,258	AX-90037092
EYA4	rs17643040	chr6:133,561,736-133,853,258	AX-15245982
EYA4	rs9483586	chr6:133,561,736-133,853,258	AX-15245989
FXN	rs7871596	chr9:71,650,479-71,715,094	AX-16248256
FXN	rs59907886	chr9:71,650,479-71,715,094	AX-16248262
FXN	---	chr9:71,650,479-71,715,094	AX-90065756
FXN	---	chr9:71,650,479-71,715,094	AX-86548923
FXN	rs9411179	chr9:71,650,479-71,715,094	AX-42628793
FXN	rs4304388	chr9:71,650,479-71,715,094	AX-42628795
FXN	rs76901520	chr9:71,650,479-71,715,094	AX-37027661
FXN	rs3793465	chr9:71,650,479-71,715,094	AX-42628805
FXN	rs143340609	chr9:71,650,479-71,715,094	AX-83566421
FXN	rs143396368	chr9:71,650,479-71,715,094	AX-90042447
FXN	rs4745581	chr9:71,650,479-71,715,094	AX-16248321
FXN	rs56657504	chr9:71,650,479-71,715,094	AX-37027731
FXN	rs7859021	chr9:71,650,479-71,715,094	AX-42628845
FXN	rs10125005	chr9:71,650,479-71,715,094	AX-11094444
FXN	rs11145194	chr9:71,650,479-71,715,094	AX-42628855
FXN	rs11145204	chr9:71,650,479-71,715,094	AX-42628857
GATA4	rs35813172	chr8:11,561,717-11,617,509	AX-156282735
GATA4	rs35813172	chr8:11,561,717-11,617,509	AX-156297257
GATA4	rs34888744	chr8:11,561,717-11,617,509	AX-36426389
GATA4	rs62489318	chr8:11,561,717-11,617,509	AX-36426395
GATA4	rs77480632	chr8:11,561,717-11,617,509	AX-121257441
GATA4	rs77480632	chr8:11,561,717-11,617,509	AX-156293760
GATA4	rs62489319	chr8:11,561,717-11,617,509	AX-156277049
GATA4	rs62489319	chr8:11,561,717-11,617,509	AX-156293761
GATA4	rs73203483	chr8:11,561,717-11,617,509	AX-36426449
GATA4	rs4269498	chr8:11,561,717-11,617,509	AX-42309109
GATA4	rs10088151	chr8:11,561,717-11,617,509	AX-42309129
GATA4	rs199922907	chr8:11,561,717-11,617,509	AX-90067531

GATA4	---	chr8:11,561,717-11,617,509	AX-90028636
GATA4	rs202213149	chr8:11,561,717-11,617,509	AX-90067532
GATA4	---	chr8:11,561,717-11,617,509	AX-90054527
GATA4	---	chr8:11,561,717-11,617,509	AX-90057739
GATA4	---	chr8:11,561,717-11,617,509	AX-90072439
GATA4	rs104894074	chr8:11,561,717-11,617,509	AX-88800140
GATA4	rs56348550	chr8:11,561,717-11,617,509	AX-91995986
GATA4	rs56348550	chr8:11,561,717-11,617,509	AX-92021113
GATA4	---	chr8:11,561,717-11,617,509	AX-90056235
GATA4	rs387906769	chr8:11,561,717-11,617,509	AX-90063886
GATA4	rs4489290	chr8:11,561,717-11,617,509	AX-156268564
GATA4	rs4489290	chr8:11,561,717-11,617,509	AX-156288299
GATA4	rs7817412	chr8:11,561,717-11,617,509	AX-156268565
GATA4	rs7817412	chr8:11,561,717-11,617,509	AX-156288300
GATA4	rs10096526	chr8:11,561,717-11,617,509	AX-156268566
GATA4	rs10096526	chr8:11,561,717-11,617,509	AX-156288301
GATA4	rs13282985	chr8:11,561,717-11,617,509	AX-156268567
GATA4	rs13249227	chr8:11,561,717-11,617,509	AX-122308974
GATA4	rs13249227	chr8:11,561,717-11,617,509	AX-96665892
GATA4	rs111721287	chr8:11,561,717-11,617,509	AX-156268568
GATA4	rs13267318	chr8:11,561,717-11,617,509	AX-42309209
GATA4	rs28473684	chr8:11,561,717-11,617,509	AX-36426689
GATA4	rs6990313	chr8:11,561,717-11,617,509	AX-147869167
GATA4	rs6990313	chr8:11,561,717-11,617,509	AX-147964355
GATA4	rs2409813	chr8:11,561,717-11,617,509	AX-120962202
GATA4	rs13278753	chr8:11,561,717-11,617,509	AX-42309249
GATA4	rs61265429	chr8:11,561,717-11,617,509	AX-36426739
GATA4	rs10112464	chr8:11,561,717-11,617,509	AX-15830516
GATA4	rs10105409	chr8:11,561,717-11,617,509	AX-42309269
GATA4	rs2409812	chr8:11,561,717-11,617,509	AX-15830547
GATA4	rs7837066	chr8:11,561,717-11,617,509	AX-156277050
GATA4	rs7837066	chr8:11,561,717-11,617,509	AX-96620463
GATA4	rs4410868	chr8:11,561,717-11,617,509	AX-15830614
GATA4	rs6601604	chr8:11,561,717-11,617,509	AX-11574698
GATA4	rs28394863	chr8:11,561,717-11,617,509	AX-36426913
GATA4	rs6601605	chr8:11,561,717-11,617,509	AX-11574699
GATA4	rs12546793	chr8:11,561,717-11,617,509	AX-156277051
GATA4	rs12546793	chr8:11,561,717-11,617,509	AX-156293762
GATA4	rs13270069	chr8:11,561,717-11,617,509	AX-15830737
GATA4	rs59792049	chr8:11,561,717-11,617,509	AX-15830757
GATA4	rs10112596	chr8:11,561,717-11,617,509	AX-15830759
GATA4	rs7833127	chr8:11,561,717-11,617,509	AX-15830760
GATA4	rs12550668	chr8:11,561,717-11,617,509	AX-11214291
GATA4	rs2409808	chr8:11,561,717-11,617,509	AX-11390262
GATA4	rs73203495	chr8:11,561,717-11,617,509	AX-36427001
GATA4	rs11994925	chr8:11,561,717-11,617,509	AX-156277052
GATA4	rs11994925	chr8:11,561,717-11,617,509	AX-156293763
GATA4	rs73663201	chr8:11,561,717-11,617,509	AX-36427003
GATA4	rs7846104	chr8:11,561,717-11,617,509	AX-156277053
GATA4	rs7846104	chr8:11,561,717-11,617,509	AX-92524696
GATA4	rs7833508	chr8:11,561,717-11,617,509	AX-151278433
GATA4	rs7833508	chr8:11,561,717-11,617,509	AX-156277054
GATA4	rs9329247	chr8:11,561,717-11,617,509	AX-15830785
GATA4	rs2409807	chr8:11,561,717-11,617,509	AX-42309389
GATA4	rs10106250	chr8:11,561,717-11,617,509	AX-42309397

GATA4	rs62489341	chr8:11,561,717-11,617,509	AX-15830825
GATA4	rs62489342	chr8:11,561,717-11,617,509	AX-36427063
GATA4	rs73203500	chr8:11,561,717-11,617,509	AX-36427067
GATA4	rs60197293	chr8:11,561,717-11,617,509	AX-36427073
GATA4	rs2898292	chr8:11,561,717-11,617,509	AX-42309413
GATA4	rs59035560	chr8:11,561,717-11,617,509	AX-156268569
GATA4	rs59035560	chr8:11,561,717-11,617,509	AX-156288303
GATA4	rs76971783	chr8:11,561,717-11,617,509	AX-36427091
GATA4	rs56745056	chr8:11,561,717-11,617,509	AX-36427093
GATA4	rs10086064	chr8:11,561,717-11,617,509	AX-42309427
GATA4	rs4840579	chr8:11,561,717-11,617,509	AX-12583314
GATA4	rs35558975	chr8:11,561,717-11,617,509	AX-36427107
GATA4	rs6986736	chr8:11,561,717-11,617,509	AX-42309433
GATA4	rs34960871	chr8:11,561,717-11,617,509	AX-36427109
GATA4	rs13277376	chr8:11,561,717-11,617,509	AX-156277055
GATA4	rs13277376	chr8:11,561,717-11,617,509	AX-156293764
GATA4	rs2409806	chr8:11,561,717-11,617,509	AX-148371899
GATA4	rs2409806	chr8:11,561,717-11,617,509	AX-156268570
GATA4	rs2001470	chr8:11,561,717-11,617,509	AX-42309473
GATA4	rs7840794	chr8:11,561,717-11,617,509	AX-148480544
GATA4	rs7840794	chr8:11,561,717-11,617,509	AX-148526330
GATA4	rs2001469	chr8:11,561,717-11,617,509	AX-148239469
GATA4	rs2001469	chr8:11,561,717-11,617,509	AX-156268571
GATA4	rs12541921	chr8:11,561,717-11,617,509	AX-156268572
GATA4	rs12541921	chr8:11,561,717-11,617,509	AX-96702732
GATA4	rs11250159	chr8:11,561,717-11,617,509	AX-11153523
GATA4	rs199521840	chr8:11,561,717-11,617,509	AX-156286428
GATA4	rs10109678	chr8:11,561,717-11,617,509	AX-36427217
GATA4	rs10096189	chr8:11,561,717-11,617,509	AX-156268573
GATA4	rs10096189	chr8:11,561,717-11,617,509	AX-156288304
GATA4	rs17153694	chr8:11,561,717-11,617,509	AX-42309517
GATA4	rs13278982	chr8:11,561,717-11,617,509	AX-36427229
GATA4	rs34421088	chr8:11,561,717-11,617,509	AX-156268574
GATA4	rs10503426	chr8:11,561,717-11,617,509	AX-11112629
GATA4	rs28488522	chr8:11,561,717-11,617,509	AX-36427247
GATA4	rs111589866	chr8:11,561,717-11,617,509	AX-38410671
GATA4	rs17153698	chr8:11,561,717-11,617,509	AX-147862748
GATA4	rs17153698	chr8:11,561,717-11,617,509	AX-147956931
GATA4	rs4613960	chr8:11,561,717-11,617,509	AX-12575841
GATA4	rs6983129	chr8:11,561,717-11,617,509	AX-11599025
GATA4	rs62489347	chr8:11,561,717-11,617,509	AX-15831110
GATA4	rs35928010	chr8:11,561,717-11,617,509	AX-156268575
GATA4	rs35726503	chr8:11,561,717-11,617,509	AX-156282736
GATA4	rs35726503	chr8:11,561,717-11,617,509	AX-156297259
GATA4	rs62489350	chr8:11,561,717-11,617,509	AX-148862103
GATA4	rs62489350	chr8:11,561,717-11,617,509	AX-156282737
GATA4	rs11250160	chr8:11,561,717-11,617,509	AX-156293765
GATA4	rs73205114	chr8:11,561,717-11,617,509	AX-156277057
GATA4	---	chr8:11,561,717-11,617,509	AX-119986538
GATA4	---	chr8:11,561,717-11,617,509	AX-156277059
GATA4	---	chr8:11,561,717-11,617,509	AX-151457597
GATA4	---	chr8:11,561,717-11,617,509	AX-156283374
GATA4	rs149549675	chr8:11,561,717-11,617,509	AX-151452223
GATA4	rs149549675	chr8:11,561,717-11,617,509	AX-156284557
GATA4	rs11250162	chr8:11,561,717-11,617,509	AX-156277060

GATA4	rs11250162	chr8:11,561,717-11,617,509	AX-92632995
GATA4	rs138014561	chr8:11,561,717-11,617,509	AX-151322242
GATA4	rs57341427	chr8:11,561,717-11,617,509	AX-151371732
GATA4	rs57341427	chr8:11,561,717-11,617,509	AX-156277061
GATA4	rs10101661	chr8:11,561,717-11,617,509	AX-11092853
GATA4	rs12544504	chr8:11,561,717-11,617,509	AX-15831160
GATA4	rs59101725	chr8:11,561,717-11,617,509	AX-156293767
GATA4	rs34962960	chr8:11,561,717-11,617,509	AX-11455978
GATA4	rs558791663	chr8:11,561,717-11,617,509	AX-151407410
GATA4	rs558791663	chr8:11,561,717-11,617,509	AX-156277063
GATA4	rs12541318	chr8:11,561,717-11,617,509	AX-42309615
GATA4	rs1390950	chr8:11,561,717-11,617,509	AX-42309633
GATA4	rs2898295	chr8:11,561,717-11,617,509	AX-42309637
GATA4	rs7838131	chr8:11,561,717-11,617,509	AX-42309639
GATA4	rs10091294	chr8:11,561,717-11,617,509	AX-42309643
GATA4	rs11250163	chr8:11,561,717-11,617,509	AX-42309651
GATA4	rs62489351	chr8:11,561,717-11,617,509	AX-156277064
GATA4	rs62489351	chr8:11,561,717-11,617,509	AX-156293768
GATA4	rs6992190	chr8:11,561,717-11,617,509	AX-156293769
GATA4	rs34814359	chr8:11,561,717-11,617,509	AX-156268576
GATA4	rs34814359	chr8:11,561,717-11,617,509	AX-156288307
GATA4	rs7827193	chr8:11,561,717-11,617,509	AX-11648298
GATA4	rs4840580	chr8:11,561,717-11,617,509	AX-153743117
GATA4	rs141325404	chr8:11,561,717-11,617,509	AX-122418309
GATA4	rs141325404	chr8:11,561,717-11,617,509	AX-122602044
GATA4	rs200975160	chr8:11,561,717-11,617,509	AX-151125236
GATA4	rs200975160	chr8:11,561,717-11,617,509	AX-156298199
GATA4	rs7832153	chr8:11,561,717-11,617,509	AX-156268577
GATA4	rs7832153	chr8:11,561,717-11,617,509	AX-156288308
GATA4	rs7845076	chr8:11,561,717-11,617,509	AX-156282738
GATA4	rs7845076	chr8:11,561,717-11,617,509	AX-156297260
GATA4	rs11988920	chr8:11,561,717-11,617,509	AX-42309685
GATA4	rs11989007	chr8:11,561,717-11,617,509	AX-122983142
GATA4	rs11989007	chr8:11,561,717-11,617,509	AX-122983143
GATA4	rs2132713	chr8:11,561,717-11,617,509	AX-42309693
GATA4	rs2132714	chr8:11,561,717-11,617,509	AX-42309695
GATA4	rs10103483	chr8:11,561,717-11,617,509	AX-156282739
GATA4	rs10103483	chr8:11,561,717-11,617,509	AX-156297261
GATA4	rs10090884	chr8:11,561,717-11,617,509	AX-42309705
GATA4	rs66494852	chr8:11,561,717-11,617,509	AX-156282740
GATA4	rs66494852	chr8:11,561,717-11,617,509	AX-156297262
GATA4	rs62489352	chr8:11,561,717-11,617,509	AX-36427459
GATA4	rs201647948	chr8:11,561,717-11,617,509	AX-151287844
GATA4	rs201647948	chr8:11,561,717-11,617,509	AX-156284628
GATA4	rs11785912	chr8:11,561,717-11,617,509	AX-42309715
GATA4	rs9644751	chr8:11,561,717-11,617,509	AX-42309721
GATA4	rs73666003	chr8:11,561,717-11,617,509	AX-36427493
GATA4	rs13275657	chr8:11,561,717-11,617,509	AX-42309731
GATA4	---	chr8:11,561,717-11,617,509	AX-36427541
GATA4	rs79676816	chr8:11,561,717-11,617,509	AX-156277066
GATA4	rs79676816	chr8:11,561,717-11,617,509	AX-156293770
GATA4	rs66535756	chr8:11,561,717-11,617,509	AX-36427561
GATA4	rs4841585	chr8:11,561,717-11,617,509	AX-148793393
GATA4	rs4841585	chr8:11,561,717-11,617,509	AX-156277067
GATA4	rs4841586	chr8:11,561,717-11,617,509	AX-156293771

GATA4	rs17153710	chr8:11,561,717-11,617,509	AX-42309761
GATA4	rs904006	chr8:11,561,717-11,617,509	AX-15831402
GATA4	rs76234685	chr8:11,561,717-11,617,509	AX-36427591
GATA4	rs28626371	chr8:11,561,717-11,617,509	AX-156277069
GATA4	rs28626371	chr8:11,561,717-11,617,509	AX-156293772
GATA4	rs28709984	chr8:11,561,717-11,617,509	AX-36427615
GATA4	rs2029969	chr8:11,561,717-11,617,509	AX-42309771
GATA4	rs28628715	chr8:11,561,717-11,617,509	AX-156277070
GATA4	rs28628715	chr8:11,561,717-11,617,509	AX-156293773
GATA4	rs2173117	chr8:11,561,717-11,617,509	AX-11372412
GATA4	rs73666006	chr8:11,561,717-11,617,509	AX-15831464
GATA4	rs2409814	chr8:11,561,717-11,617,509	AX-11390264
GATA4	rs2409814	chr8:11,561,717-11,617,509	AX-156277071
GATA4	rs3735819	chr8:11,561,717-11,617,509	AX-42309791
GATA4	rs10503425	chr8:11,561,717-11,617,509	AX-42309795
GATA4	---	chr8:11,561,717-11,617,509	AX-90061990
GATA4	---	chr8:11,561,717-11,617,509	AX-90063914
GATA4	---	chr8:11,561,717-11,617,509	AX-90057705
GATA4	---	chr8:11,561,717-11,617,509	AX-90054529
GATA4	rs398122402	chr8:11,561,717-11,617,509	AX-90054530
GATA4	---	chr8:11,561,717-11,617,509	AX-90031941
GATA4	---	chr8:11,561,717-11,617,509	AX-90077772
GATA4	---	chr8:11,561,717-11,617,509	AX-90054531
GATA4	---	chr8:11,561,717-11,617,509	AX-90025647
GATA4	---	chr8:11,561,717-11,617,509	AX-90025648
GATA4	---	chr8:11,561,717-11,617,509	AX-90025649
GATA4	---	chr8:11,561,717-11,617,509	AX-90025650
GATA4	---	chr8:11,561,717-11,617,509	AX-90064001
GATA4	---	chr8:11,561,717-11,617,509	AX-90025651
GATA4	---	chr8:11,561,717-11,617,509	AX-90025652
GATA4	---	chr8:11,561,717-11,617,509	AX-90025653
GATA4	---	chr8:11,561,717-11,617,509	AX-86640751
GATA4	---	chr8:11,561,717-11,617,509	AX-90025654
GATA4	rs73539818	chr8:11,561,717-11,617,509	AX-15831484
GATA4	rs3779664	chr8:11,561,717-11,617,509	AX-42309803
GATA4	rs3735818	chr8:11,561,717-11,617,509	AX-42309807
GATA4	rs3735817	chr8:11,561,717-11,617,509	AX-15831490
GATA4	rs3735816	chr8:11,561,717-11,617,509	AX-122675115
GATA4	rs3735816	chr8:11,561,717-11,617,509	AX-156282741
GATA4	rs3735815	chr8:11,561,717-11,617,509	AX-113398363
GATA4	rs3735814	chr8:11,561,717-11,617,509	AX-15831491
GATA4	---	chr8:11,561,717-11,617,509	AX-90054540
GATA4	---	chr8:11,561,717-11,617,509	AX-90054542
GATA4	---	chr8:11,561,717-11,617,509	AX-90054543
GATA4	rs387906771	chr8:11,561,717-11,617,509	AX-90054544
GATA4	---	chr8:11,561,717-11,617,509	AX-90054545
GATA4	---	chr8:11,561,717-11,617,509	AX-90054546
GATA4	---	chr8:11,561,717-11,617,509	AX-90025662
GATA4	---	chr8:11,561,717-11,617,509	AX-88757094
GATA4	---	chr8:11,561,717-11,617,509	AX-90065516
GATA4	---	chr8:11,561,717-11,617,509	AX-90054548
GATA4	rs3729848	chr8:11,561,717-11,617,509	AX-11476406
GATA4	rs3729849	chr8:11,561,717-11,617,509	AX-36427661
GATA4	rs7006733	chr8:11,561,717-11,617,509	AX-11600838
GATA4	rs2645396	chr8:11,561,717-11,617,509	AX-156277072

GATA4	rs2645396	chr8:11,561,717-11,617,509	AX-156293774
GATA4	rs10435714	chr8:11,561,717-11,617,509	AX-120708331
GATA4	rs10435714	chr8:11,561,717-11,617,509	AX-96648078
GATA4	rs10435718	chr8:11,561,717-11,617,509	AX-148267050
GATA4	rs10435718	chr8:11,561,717-11,617,509	AX-156268578
GATA4	rs34477636	chr8:11,561,717-11,617,509	AX-156268579
GATA4	rs12549359	chr8:11,561,717-11,617,509	AX-156288309
GATA4	rs2740434	chr8:11,561,717-11,617,509	AX-42309823
GATA4	rs2645398	chr8:11,561,717-11,617,509	AX-15831515
GATA4	rs17153740	chr8:11,561,717-11,617,509	AX-156268580
GATA4	rs17153740	chr8:11,561,717-11,617,509	AX-156288310
GATA4	rs2645399	chr8:11,561,717-11,617,509	AX-113868531
GATA4	rs2645399	chr8:11,561,717-11,617,509	AX-156288311
GATA4	rs28407037	chr8:11,561,717-11,617,509	AX-42309829
GATA4	rs2645400	chr8:11,561,717-11,617,509	AX-15831530
GATA4	rs17153743	chr8:11,561,717-11,617,509	AX-42309835
GATA4	rs73205195	chr8:11,561,717-11,617,509	AX-148784498
GATA4	rs73205195	chr8:11,561,717-11,617,509	AX-156268581
GATA4	rs35471251	chr8:11,561,717-11,617,509	AX-151469965
GATA4	rs35471251	chr8:11,561,717-11,617,509	AX-156284217
GATA4	rs36038176	chr8:11,561,717-11,617,509	AX-148518402
GATA4	rs36038176	chr8:11,561,717-11,617,509	AX-156268582
GATA4	rs11784693	chr8:11,561,717-11,617,509	AX-120720185
GATA4	rs11784693	chr8:11,561,717-11,617,509	AX-156297264
GATA4	rs11250164	chr8:11,561,717-11,617,509	AX-114330302
GATA4	rs11250164	chr8:11,561,717-11,617,509	AX-156297265
GATA4	rs804284	chr8:11,561,717-11,617,509	AX-42309857
GATA4	rs59433946	chr8:11,561,717-11,617,509	AX-36427731
GATA4	rs78129649	chr8:11,561,717-11,617,509	AX-156277073
GATA4	rs78129649	chr8:11,561,717-11,617,509	AX-156293775
GATA4	rs59431377	chr8:11,561,717-11,617,509	AX-36427737
GATA4	rs28565241	chr8:11,561,717-11,617,509	AX-36427743
GATA4	rs804283	chr8:11,561,717-11,617,509	AX-15831565
GATA4	rs810738	chr8:11,561,717-11,617,509	AX-15831567
GATA4	rs17153747	chr8:11,561,717-11,617,509	AX-12494808
GATA4	rs17153752	chr8:11,561,717-11,617,509	AX-42309859
GATA4	rs17153755	chr8:11,561,717-11,617,509	AX-11310792
GATA4	rs28665608	chr8:11,561,717-11,617,509	AX-36427757
GATA4	rs17153757	chr8:11,561,717-11,617,509	AX-36427759
GATA4	rs7826055	chr8:11,561,717-11,617,509	AX-15831579
GATA4	rs804282	chr8:11,561,717-11,617,509	AX-11661987
GATA4	rs79945119	chr8:11,561,717-11,617,509	AX-156293777
GATA4	rs804281	chr8:11,561,717-11,617,509	AX-148314768
GATA4	rs804281	chr8:11,561,717-11,617,509	AX-156277076
GATA4	rs73666008	chr8:11,561,717-11,617,509	AX-15831588
GATA4	rs13262643	chr8:11,561,717-11,617,509	AX-15831589
GATA4	rs67176365	chr8:11,561,717-11,617,509	AX-36427783
GATA4	rs13264774	chr8:11,561,717-11,617,509	AX-11247161
GATA4	rs13273672	chr8:11,561,717-11,617,509	AX-92585919
GATA4	rs387906772	chr8:11,561,717-11,617,509	AX-90054549
GATA4	rs56298569	chr8:11,561,717-11,617,509	AX-88800131
GATA4	---	chr8:11,561,717-11,617,509	AX-90054551
GATA4	---	chr8:11,561,717-11,617,509	AX-90062044
GATA4	rs804280	chr8:11,561,717-11,617,509	AX-11661981
GATA4	rs3729851	chr8:11,561,717-11,617,509	AX-11476408

GATA4	rs11987175	chr8:11,561,717-11,617,509	AX-36427797
GATA4	rs12677897	chr8:11,561,717-11,617,509	AX-42309887
GATA4	rs59648461	chr8:11,561,717-11,617,509	AX-15831605
GATA4	rs56700770	chr8:11,561,717-11,617,509	AX-151336365
GATA4	rs11987347	chr8:11,561,717-11,617,509	AX-151412576
GATA4	rs11987347	chr8:11,561,717-11,617,509	AX-156268584
GATA4	rs28438570	chr8:11,561,717-11,617,509	AX-156282742
GATA4	rs28438570	chr8:11,561,717-11,617,509	AX-156297266
GATA4	rs28550523	chr8:11,561,717-11,617,509	AX-15831612
GATA4	rs2645458	chr8:11,561,717-11,617,509	AX-42309903
GATA4	rs2645457	chr8:11,561,717-11,617,509	AX-11402989
GATA4	rs4841588	chr8:11,561,717-11,617,509	AX-156277078
GATA4	rs4841588	chr8:11,561,717-11,617,509	AX-156293779
GATA4	rs111272281	chr8:11,561,717-11,617,509	AX-107845365
GATA4	rs111272281	chr8:11,561,717-11,617,509	AX-156284728
GATA4	rs13250578	chr8:11,561,717-11,617,509	AX-156293780
GATA4	rs3729852	chr8:11,561,717-11,617,509	AX-156293781
GATA4	rs3729853	chr8:11,561,717-11,617,509	AX-36427871
GATA4	rs3729854	chr8:11,561,717-11,617,509	AX-11476409
GATA4	rs115372595	chr8:11,561,717-11,617,509	AX-68526405
GATA4	rs3729855	chr8:11,561,717-11,617,509	AX-15831621
GATA4	---	chr8:11,561,717-11,617,509	AX-90058045
GATA4	---	chr8:11,561,717-11,617,509	AX-90080066
GATA4	rs368489876	chr8:11,561,717-11,617,509	AX-86710452
GATA4	---	chr8:11,561,717-11,617,509	AX-90071479
GATA4	---	chr8:11,561,717-11,617,509	AX-94378677
GATA4	rs3729856	chr8:11,561,717-11,617,509	AX-11476410
GATA4	rs114868912	chr8:11,561,717-11,617,509	AX-36427877
GATA4	---	chr8:11,561,717-11,617,509	AX-36427879
GATA4	rs3757949	chr8:11,561,717-11,617,509	AX-107888280
GATA4	rs3757949	chr8:11,561,717-11,617,509	AX-156288312
GATA4	rs73541819	chr8:11,561,717-11,617,509	AX-15831636
GATA4	rs2645456	chr8:11,561,717-11,617,509	AX-36427887
GATA4	rs809205	chr8:11,561,717-11,617,509	AX-42309923
GATA4	rs745379	chr8:11,561,717-11,617,509	AX-36427901
GATA4	---	chr8:11,561,717-11,617,509	AX-90032250
GATA4	---	chr8:11,561,717-11,617,509	AX-90054552
GATA4	---	chr8:11,561,717-11,617,509	AX-90059735
GATA4	rs115099192	chr8:11,561,717-11,617,509	AX-38410685
GATA4	rs55633527	chr8:11,561,717-11,617,509	AX-83555177
GATA4	---	chr8:11,561,717-11,617,509	AX-90054553
GATA4	---	chr8:11,561,717-11,617,509	AX-90034720
GATA4	---	chr8:11,561,717-11,617,509	AX-90037085
GATA4	---	chr8:11,561,717-11,617,509	AX-90056773
GATA4	rs3729857	chr8:11,561,717-11,617,509	AX-42309929
GATA4	rs867858	chr8:11,561,717-11,617,509	AX-11670611
GATA4	rs1062219	chr8:11,561,717-11,617,509	AX-115089357
GATA4	rs884662	chr8:11,561,717-11,617,509	AX-113868018
GATA4	rs904018	chr8:11,561,717-11,617,509	AX-115089358
GATA4	rs904018	chr8:11,561,717-11,617,509	AX-156277080
GATA4	rs12825	chr8:11,561,717-11,617,509	AX-11228376
GATA4	rs804290	chr8:11,561,717-11,617,509	AX-42309945
GATA4	rs11785481	chr8:11,561,717-11,617,509	AX-11175451
GATA4	---	chr8:11,561,717-11,617,509	AX-42309947
GATA4	rs1062270	chr8:11,561,717-11,617,509	AX-42309949

GATA4	---	chr8:11,561,717-11,617,509	AX-42309955
GLA	rs144060196	chrX:100,652,779-100,663,001	AX-94371110
GLA	rs375661583	chrX:100,652,779-100,663,001	AX-86576904
GLA	---	chrX:100,652,779-100,663,001	AX-94376463
GLA	---	chrX:100,652,779-100,663,001	AX-86667712
GLA	rs147737890	chrX:100,652,779-100,663,001	AX-92022941
GLA	rs142217511	chrX:100,652,779-100,663,001	AX-94354356
GLA	rs28935490	chrX:100,652,779-100,663,001	AX-83005752
GLA	rs375538532	chrX:100,652,779-100,663,001	AX-86551236
GLA	rs140329381	chrX:100,652,779-100,663,001	AX-94303868
GLA	---	chrX:100,652,779-100,663,001	AX-86701523
GLA	---	chrX:100,652,779-100,663,001	AX-86683286
GLA	rs147026639	chrX:100,652,779-100,663,001	AX-94354361
GLA	rs2071397	chrX:100,652,779-100,663,001	AX-11365353
GLA	rs113959597	chrX:100,652,779-100,663,001	AX-88801032
GLA	---	chrX:100,652,779-100,663,001	AX-83202120
GLA	rs150228150	chrX:100,652,779-100,663,001	AX-94371113
GLA	rs138886989	chrX:100,652,779-100,663,001	AX-83068458
GLA	rs149391489	chrX:100,652,779-100,663,001	AX-83164487
GLA	rs143768851	chrX:100,652,779-100,663,001	AX-94371109
GLA	rs148158093	chrX:100,652,779-100,663,001	AX-83504480
GLA	---	chrX:100,652,779-100,663,001	AX-86553962
GLA	rs142449183	chrX:100,652,779-100,663,001	AX-94371107
ILK	---	chr11:6,624,964-6,632,099	AX-30202297
ILK	rs114115159	chr11:6,624,964-6,632,099	AX-83097218
ILK	rs55802517	chr11:6,624,964-6,632,099	AX-156270565
ILK	rs55802517	chr11:6,624,964-6,632,099	AX-156289360
ILK	rs12272856	chr11:6,624,964-6,632,099	AX-30202395
ILK	rs4758439	chr11:6,624,964-6,632,099	AX-16665976
ILK	rs4758440	chr11:6,624,964-6,632,099	AX-156270566
ILK	rs4758440	chr11:6,624,964-6,632,099	AX-156289361
ILK	rs72055783	chr11:6,624,964-6,632,099	AX-122530927
ILK	rs4758441	chr11:6,624,964-6,632,099	AX-11523874
ILK	rs4758442	chr11:6,624,964-6,632,099	AX-88734406
ILK	rs4758442	chr11:6,624,964-6,632,099	AX-88777451
ILK	rs11368764	chr11:6,624,964-6,632,099	AX-156286739
ILK	rs6578765	chr11:6,624,964-6,632,099	AX-148076073
ILK	rs6578766	chr11:6,624,964-6,632,099	AX-156266130
ILK	rs6578766	chr11:6,624,964-6,632,099	AX-156286740
ILK	rs11605114	chr11:6,624,964-6,632,099	AX-11161930
ILK	rs66682453	chr11:6,624,964-6,632,099	AX-156284829
ILK	rs7937456	chr11:6,624,964-6,632,099	AX-30202471
ILK	rs7938444	chr11:6,624,964-6,632,099	AX-156286741
ILK	rs79548722	chr11:6,624,964-6,632,099	AX-156283669
ILK	rs2555175	chr11:6,624,964-6,632,099	AX-11398311
ILK	rs68155159	chr11:6,624,964-6,632,099	AX-107796408
ILK	rs68155159	chr11:6,624,964-6,632,099	AX-156298436
ILK	rs2243625	chr11:6,624,964-6,632,099	AX-16665999
ILK	rs2255405	chr11:6,624,964-6,632,099	AX-12523011
ILK	rs11602107	chr11:6,624,964-6,632,099	AX-30202553
ILK	rs3741271	chr11:6,624,964-6,632,099	AX-30202599
ILK	rs2255538	chr11:6,624,964-6,632,099	AX-39139949
ILK	rs2048092	chr11:6,624,964-6,632,099	AX-39139957
ILK	rs1043388	chr11:6,624,964-6,632,099	AX-39139961
ILK	---	chr11:6,624,964-6,632,099	AX-113737992

ILK	---	chr11:6,624,964-6,632,099	AX-156297637
ILK	rs11314683	chr11:6,624,964-6,632,099	AX-83524345
ILK	rs11826498	chr11:6,624,964-6,632,099	AX-156265651
ILK	rs11826498	chr11:6,624,964-6,632,099	AX-156265686
ILK	rs140322345	chr11:6,624,964-6,632,099	AX-83563699
ILK	rs1043390	chr11:6,624,964-6,632,099	AX-30202767
ILK	rs2292195	chr11:6,624,964-6,632,099	AX-11382504
ILK	---	chr11:6,624,964-6,632,099	AX-86675509
JAG1	rs8708	chr20:10,618,332-10,654,694	AX-13471697
JAG1	rs45534738	chr20:10,618,332-10,654,694	AX-11510724
JAG1	rs138007561	chr20:10,618,332-10,654,694	AX-83275550
JAG1	rs6040050	chr20:10,618,332-10,654,694	AX-40493131
JAG1	rs75617279	chr20:10,618,332-10,654,694	AX-83241589
JAG1	rs145895196	chr20:10,618,332-10,654,694	AX-83309298
JAG1	rs35761929	chr20:10,618,332-10,654,694	AX-32837803
JAG1	rs147809756	chr20:10,618,332-10,654,694	AX-83231580
JAG1	rs1801140	chr20:10,618,332-10,654,694	AX-11344690
JAG1	rs35851937	chr20:10,618,332-10,654,694	AX-32837827
JAG1	rs544547916	chr20:10,618,332-10,654,694	AX-83286749
JAG1	rs35308216	chr20:10,618,332-10,654,694	AX-32837833
JAG1	rs6077864	chr20:10,618,332-10,654,694	AX-32837841
JAG1	rs35793014	chr20:10,618,332-10,654,694	AX-12557509
JAG1	---	chr20:10,618,332-10,654,694	AX-40493151
JAG1	rs6040055	chr20:10,618,332-10,654,694	AX-11556489
JAG1	rs75809680	chr20:10,618,332-10,654,694	AX-13471708
JAG1	rs62185094	chr20:10,618,332-10,654,694	AX-13471712
JAG1	rs2273061	chr20:10,618,332-10,654,694	AX-40493157
JAG1	rs3748480	chr20:10,618,332-10,654,694	AX-11477884
JAG1	rs6133987	chr20:10,618,332-10,654,694	AX-40493189
JAG1	rs2235810	chr20:10,618,332-10,654,694	AX-32837919
KCNE1	rs73363349	chr21:35,818,986-35,828,107	AX-33245987
KCNE1	rs11909074	chr21:35,818,986-35,828,107	AX-40688999
KCNE1	---	chr21:35,818,986-35,828,107	AX-90031412
KCNE1	rs142511345	chr21:35,818,986-35,828,107	AX-86686153
KCNE1	---	chr21:35,818,986-35,828,107	AX-90064816
KCNE1	---	chr21:35,818,986-35,828,107	AX-86631626
KCNE1	---	chr21:35,818,986-35,828,107	AX-90033348
KCNE1	rs1805128	chr21:35,818,986-35,828,107	AX-11344915
KCNE1	rs199473360	chr21:35,818,986-35,828,107	AX-90034239
KCNE1	---	chr21:35,818,986-35,828,107	AX-90061783
KCNE1	rs74315445	chr21:35,818,986-35,828,107	AX-86728661
KCNE1	rs74315446	chr21:35,818,986-35,828,107	AX-90039555
KCNE1	---	chr21:35,818,986-35,828,107	AX-90062070
KCNE1	rs79654911	chr21:35,818,986-35,828,107	AX-90070522
KCNE1	---	chr21:35,818,986-35,828,107	AX-90028861
KCNE1	---	chr21:35,818,986-35,828,107	AX-90061015
KCNE1	rs147187721	chr21:35,818,986-35,828,107	AX-86632330
KCNE1	rs199473644	chr21:35,818,986-35,828,107	AX-86707171
KCNE1	---	chr21:35,818,986-35,828,107	AX-90035711
KCNE1	---	chr21:35,818,986-35,828,107	AX-90028144
KCNE1	---	chr21:35,818,986-35,828,107	AX-90056334
KCNE1	rs1805127	chr21:35,818,986-35,828,107	AX-13660397
KCNE1	rs17173510	chr21:35,818,986-35,828,107	AX-11312351
KCNE1	---	chr21:35,818,986-35,828,107	AX-86593770
KCNE1	---	chr21:35,818,986-35,828,107	AX-90030238

KCNE1	---	chr21:35,818,986-35,828,107	AX-90034575
KCNE1	rs144917638	chr21:35,818,986-35,828,107	AX-86676005
KCNE1	rs199473348	chr21:35,818,986-35,828,107	AX-86652928
KCNE1	---	chr21:35,818,986-35,828,107	AX-90046829
KCNE1	---	chr21:35,818,986-35,828,107	AX-90044154
KCNE1	rs55857049	chr21:35,818,986-35,828,107	AX-33246011
KCNE1	rs79687087	chr21:35,818,986-35,828,107	AX-13660399
KCNE1	rs1547356	chr21:35,818,986-35,828,107	AX-13660400
KCNE1	rs1547357	chr21:35,818,986-35,828,107	AX-40689013
KCNE1	rs9976263	chr21:35,818,986-35,828,107	AX-13660409
KCNE1	rs7277999	chr21:35,818,986-35,828,107	AX-12626610
KCNE2	rs4816481	chr21:35,736,323-35,743,440	AX-11528518
KCNE2	rs11702830	chr21:35,736,323-35,743,440	AX-11169026
KCNE2	rs12481733	chr21:35,736,323-35,743,440	AX-40688763
KCNE2	rs12483695	chr21:35,736,323-35,743,440	AX-40688765
KCNE2	rs2234916	chr21:35,736,323-35,743,440	AX-82888319
KCNE2	---	chr21:35,736,323-35,743,440	AX-13660085
KCNE2	---	chr21:35,736,323-35,743,440	AX-90032433
KCNE2	rs142153692	chr21:35,736,323-35,743,440	AX-83278271
KCNE2	---	chr21:35,736,323-35,743,440	AX-90058849
KCNE2	rs74315449	chr21:35,736,323-35,743,440	AX-86687780
KCNE2	rs148968498	chr21:35,736,323-35,743,440	AX-86741030
KCNE2	---	chr21:35,736,323-35,743,440	AX-90048538
KCNE2	---	chr21:35,736,323-35,743,440	AX-86687985
KCNE2	rs74315447	chr21:35,736,323-35,743,440	AX-83413041
KCNE2	rs74315448	chr21:35,736,323-35,743,440	AX-83476889
KCNE2	rs16991654	chr21:35,736,323-35,743,440	AX-90037861
KCNE2	---	chr21:35,736,323-35,743,440	AX-86556188
KCNE2	---	chr21:35,736,323-35,743,440	AX-90028179
KCNE2	rs141423405	chr21:35,736,323-35,743,440	AX-86683323
KCNE2	rs199473365	chr21:35,736,323-35,743,440	AX-86688906
KCNE2	rs74424227	chr21:35,736,323-35,743,440	AX-90068833
KCNE2	---	chr21:35,736,323-35,743,440	AX-86580727
KCNE2	rs45610936	chr21:35,736,323-35,743,440	AX-86681645
KCNH2	---	chr7:150,642,044-150,675,402	AX-90063331
KCNH2	---	chr7:150,642,044-150,675,402	AX-90043291
KCNH2	---	chr7:150,642,044-150,675,402	AX-90064023
KCNH2	---	chr7:150,642,044-150,675,402	AX-90033511
KCNH2	rs146664701	chr7:150,642,044-150,675,402	AX-121467398
KCNH2	rs146664701	chr7:150,642,044-150,675,402	AX-156284945
KCNH2	---	chr7:150,642,044-150,675,402	AX-90051494
KCNH2	---	chr7:150,642,044-150,675,402	AX-90058581
KCNH2	rs199473028	chr7:150,642,044-150,675,402	AX-86726114
KCNH2	---	chr7:150,642,044-150,675,402	AX-90036272
KCNH2	---	chr7:150,642,044-150,675,402	AX-90033529
KCNH2	---	chr7:150,642,044-150,675,402	AX-90079290
KCNH2	---	chr7:150,642,044-150,675,402	AX-90050717
KCNH2	---	chr7:150,642,044-150,675,402	AX-90045457
KCNH2	---	chr7:150,642,044-150,675,402	AX-90033198
KCNH2	---	chr7:150,642,044-150,675,402	AX-90049724
KCNH2	rs36210421	chr7:150,642,044-150,675,402	AX-83580519
KCNH2	---	chr7:150,642,044-150,675,402	AX-90064890
KCNH2	---	chr7:150,642,044-150,675,402	AX-90057928
KCNH2	---	chr7:150,642,044-150,675,402	AX-90052579
KCNH2	---	chr7:150,642,044-150,675,402	AX-90060220

KCNH2	---	chr7:150,642,044-150,675,402	AX-90045070
KCNH2	---	chr7:150,642,044-150,675,402	AX-90046038
KCNH2	---	chr7:150,642,044-150,675,402	AX-90073625
KCNH2	---	chr7:150,642,044-150,675,402	AX-90078481
KCNH2	---	chr7:150,642,044-150,675,402	AX-90062458
KCNH2	---	chr7:150,642,044-150,675,402	AX-90064191
KCNH2	---	chr7:150,642,044-150,675,402	AX-90080816
KCNH2	---	chr7:150,642,044-150,675,402	AX-86663258
KCNH2	---	chr7:150,642,044-150,675,402	AX-90072742
KCNH2	---	chr7:150,642,044-150,675,402	AX-86550370
KCNH2	---	chr7:150,642,044-150,675,402	AX-90078192
KCNH2	rs121912509	chr7:150,642,044-150,675,402	AX-90071171
KCNH2	---	chr7:150,642,044-150,675,402	AX-90065520
KCNH2	---	chr7:150,642,044-150,675,402	AX-90060317
KCNH2	---	chr7:150,642,044-150,675,402	AX-90075976
KCNH2	---	chr7:150,642,044-150,675,402	AX-90080662
KCNH2	rs149955375	chr7:150,642,044-150,675,402	AX-83052666
KCNH2	---	chr7:150,642,044-150,675,402	AX-90033904
KCNH2	---	chr7:150,642,044-150,675,402	AX-90074113
KCNH2	---	chr7:150,642,044-150,675,402	AX-90029917
KCNH2	---	chr7:150,642,044-150,675,402	AX-90064063
KCNH2	---	chr7:150,642,044-150,675,402	AX-90064477
KCNH2	---	chr7:150,642,044-150,675,402	AX-90078860
KCNH2	---	chr7:150,642,044-150,675,402	AX-90056038
KCNH2	---	chr7:150,642,044-150,675,402	AX-90073908
KCNH2	rs6948860	chr7:150,642,044-150,675,402	AX-96744308
KCNH2	rs6948860	chr7:150,642,044-150,675,402	AX-96746519
KCNH2	rs1805123	chr7:150,642,044-150,675,402	AX-42143543
KCNH2	---	chr7:150,642,044-150,675,402	AX-90078194
KCNH2	---	chr7:150,642,044-150,675,402	AX-90060780
KCNH2	---	chr7:150,642,044-150,675,402	AX-90073776
KCNH2	---	chr7:150,642,044-150,675,402	AX-90065694
KCNH2	rs199473432	chr7:150,642,044-150,675,402	AX-90030835
KCNH2	---	chr7:150,642,044-150,675,402	AX-90043042
KCNH2	rs143512106	chr7:150,642,044-150,675,402	AX-83336883
KCNH2	---	chr7:150,642,044-150,675,402	AX-90030926
KCNH2	---	chr7:150,642,044-150,675,402	AX-90052213
KCNH2	---	chr7:150,642,044-150,675,402	AX-90034191
KCNH2	---	chr7:150,642,044-150,675,402	AX-90043266
KCNH2	rs2968860	chr7:150,642,044-150,675,402	AX-96986338
KCNH2	---	chr7:150,642,044-150,675,402	AX-90062016
KCNH2	---	chr7:150,642,044-150,675,402	AX-90064923
KCNH2	---	chr7:150,642,044-150,675,402	AX-90065534
KCNH2	---	chr7:150,642,044-150,675,402	AX-86616384
KCNH2	---	chr7:150,642,044-150,675,402	AX-90055887
KCNH2	---	chr7:150,642,044-150,675,402	AX-90064918
KCNH2	---	chr7:150,642,044-150,675,402	AX-90027859
KCNH2	---	chr7:150,642,044-150,675,402	AX-90081211
KCNH2	---	chr7:150,642,044-150,675,402	AX-90060567
KCNH2	---	chr7:150,642,044-150,675,402	AX-90059390
KCNH2	---	chr7:150,642,044-150,675,402	AX-90057280
KCNH2	---	chr7:150,642,044-150,675,402	AX-90031876
KCNH2	---	chr7:150,642,044-150,675,402	AX-90057233
KCNH2	---	chr7:150,642,044-150,675,402	AX-90050053
KCNH2	---	chr7:150,642,044-150,675,402	AX-90036551

KCNH2	rs121912506	chr7:150,642,044-150,675,402	AX-90071168
KCNH2	rs121912510	chr7:150,642,044-150,675,402	AX-90071172
KCNH2	---	chr7:150,642,044-150,675,402	AX-90029310
KCNH2	---	chr7:150,642,044-150,675,402	AX-90027455
KCNH2	---	chr7:150,642,044-150,675,402	AX-90061346
KCNH2	---	chr7:150,642,044-150,675,402	AX-90029742
KCNH2	---	chr7:150,642,044-150,675,402	AX-90075325
KCNH2	---	chr7:150,642,044-150,675,402	AX-90033828
KCNH2	---	chr7:150,642,044-150,675,402	AX-90032062
KCNH2	---	chr7:150,642,044-150,675,402	AX-90058144
KCNH2	---	chr7:150,642,044-150,675,402	AX-90061116
KCNH2	---	chr7:150,642,044-150,675,402	AX-90058919
KCNH2	---	chr7:150,642,044-150,675,402	AX-90063349
KCNH2	---	chr7:150,642,044-150,675,402	AX-90078993
KCNH2	rs138498207	chr7:150,642,044-150,675,402	AX-83204515
KCNH2	---	chr7:150,642,044-150,675,402	AX-90057696
KCNH2	---	chr7:150,642,044-150,675,402	AX-90062650
KCNH2	---	chr7:150,642,044-150,675,402	AX-90028120
KCNH2	---	chr7:150,642,044-150,675,402	AX-90065341
KCNH2	---	chr7:150,642,044-150,675,402	AX-90052255
KCNH2	---	chr7:150,642,044-150,675,402	AX-90056144
KCNH2	---	chr7:150,642,044-150,675,402	AX-90037313
KCNH2	---	chr7:150,642,044-150,675,402	AX-90031790
KCNH2	---	chr7:150,642,044-150,675,402	AX-90081441
KCNH2	---	chr7:150,642,044-150,675,402	AX-90028483
KCNH2	rs121912512	chr7:150,642,044-150,675,402	AX-86642816
KCNH2	---	chr7:150,642,044-150,675,402	AX-90028601
KCNH2	---	chr7:150,642,044-150,675,402	AX-90032275
KCNH2	---	chr7:150,642,044-150,675,402	AX-90076090
KCNH2	---	chr7:150,642,044-150,675,402	AX-90049020
KCNH2	---	chr7:150,642,044-150,675,402	AX-90065769
KCNH2	---	chr7:150,642,044-150,675,402	AX-90077955
KCNH2	---	chr7:150,642,044-150,675,402	AX-90055943
KCNH2	---	chr7:150,642,044-150,675,402	AX-90028110
KCNH2	---	chr7:150,642,044-150,675,402	AX-90077270
KCNH2	---	chr7:150,642,044-150,675,402	AX-90059692
KCNH2	---	chr7:150,642,044-150,675,402	AX-90032469
KCNH2	---	chr7:150,642,044-150,675,402	AX-90048117
KCNH2	rs4725983	chr7:150,642,044-150,675,402	AX-112919529
KCNH2	rs2072413	chr7:150,642,044-150,675,402	AX-96922360
KCNH2	rs2072412	chr7:150,642,044-150,675,402	AX-113404865
KCNH2	---	chr7:150,642,044-150,675,402	AX-90059365
KCNH2	rs199473532	chr7:150,642,044-150,675,402	AX-86638542
KCNH2	---	chr7:150,642,044-150,675,402	AX-90033349
KCNH2	---	chr7:150,642,044-150,675,402	AX-90059002
KCNH2	---	chr7:150,642,044-150,675,402	AX-90065339
KCNH2	---	chr7:150,642,044-150,675,402	AX-90036018
KCNH2	---	chr7:150,642,044-150,675,402	AX-90032569
KCNH2	---	chr7:150,642,044-150,675,402	AX-90034407
KCNH2	---	chr7:150,642,044-150,675,402	AX-90027463
KCNH2	---	chr7:150,642,044-150,675,402	AX-90030612
KCNH2	---	chr7:150,642,044-150,675,402	AX-90081405
KCNH2	---	chr7:150,642,044-150,675,402	AX-90079999
KCNH2	---	chr7:150,642,044-150,675,402	AX-90050866
KCNH2	---	chr7:150,642,044-150,675,402	AX-90057492

KCNH2	---	chr7:150,642,044-150,675,402	AX-90064104
KCNH2	---	chr7:150,642,044-150,675,402	AX-90061762
KCNH2	---	chr7:150,642,044-150,675,402	AX-90030973
KCNH2	---	chr7:150,642,044-150,675,402	AX-90032267
KCNH2	---	chr7:150,642,044-150,675,402	AX-90031265
KCNH2	rs1137617	chr7:150,642,044-150,675,402	AX-36091005
KCNH2	---	chr7:150,642,044-150,675,402	AX-90056511
KCNH2	---	chr7:150,642,044-150,675,402	AX-90056415
KCNH2	---	chr7:150,642,044-150,675,402	AX-90037272
KCNH2	---	chr7:150,642,044-150,675,402	AX-90032575
KCNH2	---	chr7:150,642,044-150,675,402	AX-90060704
KCNH2	---	chr7:150,642,044-150,675,402	AX-90033275
KCNH2	---	chr7:150,642,044-150,675,402	AX-90060108
KCNH2	---	chr7:150,642,044-150,675,402	AX-90062356
KCNH2	---	chr7:150,642,044-150,675,402	AX-90072213
KCNH2	---	chr7:150,642,044-150,675,402	AX-90063426
KCNH2	---	chr7:150,642,044-150,675,402	AX-90056444
KCNH2	---	chr7:150,642,044-150,675,402	AX-90074169
KCNH2	---	chr7:150,642,044-150,675,402	AX-90034461
KCNH2	---	chr7:150,642,044-150,675,402	AX-90057951
KCNH2	---	chr7:150,642,044-150,675,402	AX-90064630
KCNH2	---	chr7:150,642,044-150,675,402	AX-90031330
KCNH2	---	chr7:150,642,044-150,675,402	AX-90058307
KCNH2	---	chr7:150,642,044-150,675,402	AX-90065098
KCNH2	---	chr7:150,642,044-150,675,402	AX-90058354
KCNH2	---	chr7:150,642,044-150,675,402	AX-90064088
KCNH2	---	chr7:150,642,044-150,675,402	AX-90030711
KCNH2	---	chr7:150,642,044-150,675,402	AX-90036983
KCNH2	---	chr7:150,642,044-150,675,402	AX-90030562
KCNH2	---	chr7:150,642,044-150,675,402	AX-90062514
KCNH2	---	chr7:150,642,044-150,675,402	AX-90032159
KCNH2	---	chr7:150,642,044-150,675,402	AX-90061157
KCNH2	---	chr7:150,642,044-150,675,402	AX-90034675
KCNH2	---	chr7:150,642,044-150,675,402	AX-90055940
KCNH2	---	chr7:150,642,044-150,675,402	AX-90059132
KCNH2	---	chr7:150,642,044-150,675,402	AX-90058615
KCNH2	---	chr7:150,642,044-150,675,402	AX-90033491
KCNH2	---	chr7:150,642,044-150,675,402	AX-90064169
KCNH2	---	chr7:150,642,044-150,675,402	AX-90057534
KCNH2	---	chr7:150,642,044-150,675,402	AX-90027810
KCNH2	---	chr7:150,642,044-150,675,402	AX-90060477
KCNH2	---	chr7:150,642,044-150,675,402	AX-90063669
KCNH2	---	chr7:150,642,044-150,675,402	AX-90057843
KCNH2	---	chr7:150,642,044-150,675,402	AX-90060083
KCNH2	---	chr7:150,642,044-150,675,402	AX-90063258
KCNH2	---	chr7:150,642,044-150,675,402	AX-90062948
KCNH2	---	chr7:150,642,044-150,675,402	AX-90061229
KCNH2	---	chr7:150,642,044-150,675,402	AX-90030659
KCNH2	---	chr7:150,642,044-150,675,402	AX-90032622
KCNH2	---	chr7:150,642,044-150,675,402	AX-90036389
KCNH2	---	chr7:150,642,044-150,675,402	AX-90064251
KCNH2	---	chr7:150,642,044-150,675,402	AX-90061502
KCNH2	---	chr7:150,642,044-150,675,402	AX-90034873
KCNH2	---	chr7:150,642,044-150,675,402	AX-90036724
KCNH2	---	chr7:150,642,044-150,675,402	AX-90059245

KCNH2	---	chr7:150,642,044-150,675,402	AX-90060342
KCNH2	---	chr7:150,642,044-150,675,402	AX-90032692
KCNH2	---	chr7:150,642,044-150,675,402	AX-90032517
KCNH2	---	chr7:150,642,044-150,675,402	AX-90059511
KCNH2	---	chr7:150,642,044-150,675,402	AX-90057263
KCNH2	---	chr7:150,642,044-150,675,402	AX-90062609
KCNH2	---	chr7:150,642,044-150,675,402	AX-90034547
KCNH2	---	chr7:150,642,044-150,675,402	AX-90060469
KCNH2	---	chr7:150,642,044-150,675,402	AX-90055952
KCNH2	---	chr7:150,642,044-150,675,402	AX-90059790
KCNH2	---	chr7:150,642,044-150,675,402	AX-90061672
KCNH2	---	chr7:150,642,044-150,675,402	AX-90032117
KCNH2	---	chr7:150,642,044-150,675,402	AX-90028657
KCNH2	---	chr7:150,642,044-150,675,402	AX-90031242
KCNH2	---	chr7:150,642,044-150,675,402	AX-90036831
KCNH2	---	chr7:150,642,044-150,675,402	AX-90033489
KCNH2	---	chr7:150,642,044-150,675,402	AX-90060702
KCNH2	rs121912508	chr7:150,642,044-150,675,402	AX-90042285
KCNH2	---	chr7:150,642,044-150,675,402	AX-90059314
KCNH2	---	chr7:150,642,044-150,675,402	AX-90062339
KCNH2	---	chr7:150,642,044-150,675,402	AX-90077203
KCNH2	---	chr7:150,642,044-150,675,402	AX-90060262
KCNH2	---	chr7:150,642,044-150,675,402	AX-90033431
KCNH2	---	chr7:150,642,044-150,675,402	AX-90027003
KCNH2	---	chr7:150,642,044-150,675,402	AX-90027895
KCNH2	---	chr7:150,642,044-150,675,402	AX-90034315
KCNH2	---	chr7:150,642,044-150,675,402	AX-90031478
KCNH2	---	chr7:150,642,044-150,675,402	AX-90033518
KCNH2	---	chr7:150,642,044-150,675,402	AX-90029359
KCNH2	---	chr7:150,642,044-150,675,402	AX-90048694
KCNH2	---	chr7:150,642,044-150,675,402	AX-90033408
KCNH2	---	chr7:150,642,044-150,675,402	AX-90057794
KCNH2	---	chr7:150,642,044-150,675,402	AX-90064589
KCNH2	---	chr7:150,642,044-150,675,402	AX-90061046
KCNH2	rs1805121	chr7:150,642,044-150,675,402	AX-42143551
KCNH2	---	chr7:150,642,044-150,675,402	AX-90062659
KCNH2	---	chr7:150,642,044-150,675,402	AX-90065681
KCNH2	---	chr7:150,642,044-150,675,402	AX-90057683
KCNH2	---	chr7:150,642,044-150,675,402	AX-90061401
KCNH2	---	chr7:150,642,044-150,675,402	AX-90027273
KCNH2	rs121912516	chr7:150,642,044-150,675,402	AX-90042288
KCNH2	---	chr7:150,642,044-150,675,402	AX-90079142
KCNH2	---	chr7:150,642,044-150,675,402	AX-90046014
KCNH2	rs199472918	chr7:150,642,044-150,675,402	AX-86687248
KCNH2	---	chr7:150,642,044-150,675,402	AX-90043947
KCNH2	rs375872367	chr7:150,642,044-150,675,402	AX-86633551
KCNH2	---	chr7:150,642,044-150,675,402	AX-90036205
KCNH2	---	chr7:150,642,044-150,675,402	AX-90031820
KCNH2	rs202102276	chr7:150,642,044-150,675,402	AX-82911540
KCNH2	---	chr7:150,642,044-150,675,402	AX-90034168
KCNH2	---	chr7:150,642,044-150,675,402	AX-90063071
KCNH2	---	chr7:150,642,044-150,675,402	AX-90027511
KCNH2	rs2072411	chr7:150,642,044-150,675,402	AX-96971248
KCNH2	rs2072411	chr7:150,642,044-150,675,402	AX-96999353
KCNH2	rs35117381	chr7:150,642,044-150,675,402	AX-96986536

KCNH2	rs112283042	chr7:150,642,044-150,675,402	AX-97471321
KCNH2	---	chr7:150,642,044-150,675,402	AX-90060121
KCNH2	rs1805120	chr7:150,642,044-150,675,402	AX-96880250
KCNH2	rs1805120	chr7:150,642,044-150,675,402	AX-96882384
KCNH2	---	chr7:150,642,044-150,675,402	AX-90028673
KCNH2	---	chr7:150,642,044-150,675,402	AX-90027940
KCNH2	---	chr7:150,642,044-150,675,402	AX-90035258
KCNH2	---	chr7:150,642,044-150,675,402	AX-90036651
KCNH2	---	chr7:150,642,044-150,675,402	AX-90063654
KCNH2	---	chr7:150,642,044-150,675,402	AX-90035821
KCNH2	---	chr7:150,642,044-150,675,402	AX-86569549
KCNH2	---	chr7:150,642,044-150,675,402	AX-90038075
KCNH2	---	chr7:150,642,044-150,675,402	AX-90063472
KCNH2	---	chr7:150,642,044-150,675,402	AX-36091011
KCNH2	---	chr7:150,642,044-150,675,402	AX-90029751
KCNH2	---	chr7:150,642,044-150,675,402	AX-90059832
KCNH2	---	chr7:150,642,044-150,675,402	AX-90078241
KCNH2	---	chr7:150,642,044-150,675,402	AX-90027988
KCNH2	---	chr7:150,642,044-150,675,402	AX-90033609
KCNH2	---	chr7:150,642,044-150,675,402	AX-90031614
KCNH2	---	chr7:150,642,044-150,675,402	AX-90058188
KCNH2	---	chr7:150,642,044-150,675,402	AX-90077738
KCNH2	---	chr7:150,642,044-150,675,402	AX-90063655
KCNH2	---	chr7:150,642,044-150,675,402	AX-90059576
KCNH2	---	chr7:150,642,044-150,675,402	AX-90062312
KCNH2	---	chr7:150,642,044-150,675,402	AX-90031094
KCNH2	---	chr7:150,642,044-150,675,402	AX-90029985
KCNH2	---	chr7:150,642,044-150,675,402	AX-90074611
KCNH2	---	chr7:150,642,044-150,675,402	AX-90064939
KCNH2	---	chr7:150,642,044-150,675,402	AX-90063944
KCNH2	---	chr7:150,642,044-150,675,402	AX-90042865
KCNH2	---	chr7:150,642,044-150,675,402	AX-86568064
KCNH2	---	chr7:150,642,044-150,675,402	AX-90033762
KCNH2	---	chr7:150,642,044-150,675,402	AX-90047511
KCNH2	---	chr7:150,642,044-150,675,402	AX-90028037
KCNH2	---	chr7:150,642,044-150,675,402	AX-90036375
KCNH2	---	chr7:150,642,044-150,675,402	AX-90057557
KCNH2	---	chr7:150,642,044-150,675,402	AX-90066127
KCNH2	---	chr7:150,642,044-150,675,402	AX-90064004
KCNH2	---	chr7:150,642,044-150,675,402	AX-90028281
KCNH2	---	chr7:150,642,044-150,675,402	AX-90044037
KCNH2	---	chr7:150,642,044-150,675,402	AX-90027445
KCNH2	---	chr7:150,642,044-150,675,402	AX-90034523
KCNH2	---	chr7:150,642,044-150,675,402	AX-90059076
KCNH2	---	chr7:150,642,044-150,675,402	AX-90057184
KCNH2	---	chr7:150,642,044-150,675,402	AX-90037283
KCNH2	---	chr7:150,642,044-150,675,402	AX-90058263
KCNH2	---	chr7:150,642,044-150,675,402	AX-90036795
KCNH2	---	chr7:150,642,044-150,675,402	AX-90037346
KCNH2	---	chr7:150,642,044-150,675,402	AX-90058468
KCNH2	---	chr7:150,642,044-150,675,402	AX-90043586
KCNH2	---	chr7:150,642,044-150,675,402	AX-90029877
KCNH2	rs2269000	chr7:150,642,044-150,675,402	AX-36091015
KCNH2	rs2968862	chr7:150,642,044-150,675,402	AX-112919530
KCNH2	rs2269001	chr7:150,642,044-150,675,402	AX-42143555

KCNH2	rs887586	chr7:150,642,044-150,675,402	AX-36091027
KCNH2	rs876088	chr7:150,642,044-150,675,402	AX-96924278
KCNH2	rs141096923	chr7:150,642,044-150,675,402	AX-96785711
KCNH2	rs141096923	chr7:150,642,044-150,675,402	AX-97448176
KCNH2	rs882156	chr7:150,642,044-150,675,402	AX-97389618
KCNH2	---	chr7:150,642,044-150,675,402	AX-90064350
KCNH2	---	chr7:150,642,044-150,675,402	AX-90036282
KCNH2	rs138776684	chr7:150,642,044-150,675,402	AX-83426361
KCNH2	---	chr7:150,642,044-150,675,402	AX-90030777
KCNH2	---	chr7:150,642,044-150,675,402	AX-90050167
KCNH2	---	chr7:150,642,044-150,675,402	AX-90077043
KCNH2	---	chr7:150,642,044-150,675,402	AX-90027632
KCNH2	---	chr7:150,642,044-150,675,402	AX-90035380
KCNH2	---	chr7:150,642,044-150,675,402	AX-90077438
KCNH2	rs199473505	chr7:150,642,044-150,675,402	AX-82969773
KCNH2	---	chr7:150,642,044-150,675,402	AX-86553087
KCNH2	---	chr7:150,642,044-150,675,402	AX-90057690
KCNH2	---	chr7:150,642,044-150,675,402	AX-90062833
KCNH2	rs199472885	chr7:150,642,044-150,675,402	AX-90042353
KCNH2	---	chr7:150,642,044-150,675,402	AX-90046840
KCNH2	---	chr7:150,642,044-150,675,402	AX-90080799
KCNH2	rs4725385	chr7:150,642,044-150,675,402	AX-36091041
KCNH2	---	chr7:150,642,044-150,675,402	AX-90058240
KCNH2	---	chr7:150,642,044-150,675,402	AX-90079424
KCNH2	---	chr7:150,642,044-150,675,402	AX-90064673
KCNH2	---	chr7:150,642,044-150,675,402	AX-90028568
KCNH2	---	chr7:150,642,044-150,675,402	AX-90027329
KCNH2	---	chr7:150,642,044-150,675,402	AX-90035058
KCNH2	---	chr7:150,642,044-150,675,402	AX-90030397
KCNH2	---	chr7:150,642,044-150,675,402	AX-90060051
KCNH2	---	chr7:150,642,044-150,675,402	AX-90063487
KCNH2	---	chr7:150,642,044-150,675,402	AX-90063381
KCNH2	---	chr7:150,642,044-150,675,402	AX-90029992
KCNH2	---	chr7:150,642,044-150,675,402	AX-90034497
KCNH2	rs758890	chr7:150,642,044-150,675,402	AX-156282450
KCNH2	rs41307316	chr7:150,642,044-150,675,402	AX-36091053
KCNH2	---	chr7:150,642,044-150,675,402	AX-90074642
KCNH2	---	chr7:150,642,044-150,675,402	AX-90079871
KCNH2	rs139544114	chr7:150,642,044-150,675,402	AX-83308502
KCNH2	---	chr7:150,642,044-150,675,402	AX-90051612
KCNH2	rs199472864	chr7:150,642,044-150,675,402	AX-86554923
KCNH2	---	chr7:150,642,044-150,675,402	AX-90066121
KCNH2	---	chr7:150,642,044-150,675,402	AX-90052225
KCNH2	---	chr7:150,642,044-150,675,402	AX-90033975
KCNH2	---	chr7:150,642,044-150,675,402	AX-90033138
KCNH2	rs150988911	chr7:150,642,044-150,675,402	AX-86726286
KCNH2	---	chr7:150,642,044-150,675,402	AX-90033783
KCNH2	---	chr7:150,642,044-150,675,402	AX-90048381
KCNH2	---	chr7:150,642,044-150,675,402	AX-90031003
KCNH2	---	chr7:150,642,044-150,675,402	AX-90063628
KCNH2	---	chr7:150,642,044-150,675,402	AX-90031415
KCNH2	---	chr7:150,642,044-150,675,402	AX-90036079
KCNH2	---	chr7:150,642,044-150,675,402	AX-90036144
KCNH2	---	chr7:150,642,044-150,675,402	AX-90060257
KCNH2	rs56282717	chr7:150,642,044-150,675,402	AX-36091055

KCNH2	rs12668582	chr7:150,642,044-150,675,402	AX-113961427
KCNH2	rs6947240	chr7:150,642,044-150,675,402	AX-113882354
KCNH2	rs6947240	chr7:150,642,044-150,675,402	AX-113956138
KCNH2	rs55851300	chr7:150,642,044-150,675,402	AX-151150024
KCNH2	rs55851300	chr7:150,642,044-150,675,402	AX-156297907
KCNH2	rs73167623	chr7:150,642,044-150,675,402	AX-113429276
KCNH2	rs73167623	chr7:150,642,044-150,675,402	AX-113985538
KCNH2	rs34657537	chr7:150,642,044-150,675,402	AX-36091061
KCNH2	rs3823587	chr7:150,642,044-150,675,402	AX-156276348
KCNH2	rs3823587	chr7:150,642,044-150,675,402	AX-156293264
KCNH2	rs3807377	chr7:150,642,044-150,675,402	AX-36091063
KCNH2	rs3807376	chr7:150,642,044-150,675,402	AX-96978614
KCNH2	rs3807376	chr7:150,642,044-150,675,402	AX-96993770
KCNH2	rs35760656	chr7:150,642,044-150,675,402	AX-156293265
KCNH2	rs3778874	chr7:150,642,044-150,675,402	AX-96970068
KCNH2	rs3778874	chr7:150,642,044-150,675,402	AX-97002447
KCNH2	rs4725386	chr7:150,642,044-150,675,402	AX-36091077
KCNH2	rs7789146	chr7:150,642,044-150,675,402	AX-36091089
KCNH2	rs7789585	chr7:150,642,044-150,675,402	AX-156276350
KCNH2	rs2907947	chr7:150,642,044-150,675,402	AX-36091097
KCNH2	rs11766022	chr7:150,642,044-150,675,402	AX-36091099
KCNH2	rs2968857	chr7:150,642,044-150,675,402	AX-42143565
KCNH2	---	chr7:150,642,044-150,675,402	AX-96761932
KCNH2	---	chr7:150,642,044-150,675,402	AX-96794302
KCNH2	rs201403582	chr7:150,642,044-150,675,402	AX-96872347
KCNH2	rs139397678	chr7:150,642,044-150,675,402	AX-96906308
KCNH2	rs139397678	chr7:150,642,044-150,675,402	AX-96925882
KCNH2	rs112948920	chr7:150,642,044-150,675,402	AX-151385995
KCNH2	rs112948920	chr7:150,642,044-150,675,402	AX-156286056
KCNH2	rs12540183	chr7:150,642,044-150,675,402	AX-112946285
KCNH2	rs13244889	chr7:150,642,044-150,675,402	AX-36091109
KCNH2	rs41308978	chr7:150,642,044-150,675,402	AX-36091115
KCNH2	rs3807375	chr7:150,642,044-150,675,402	AX-42143569
KCNH2	rs10236214	chr7:150,642,044-150,675,402	AX-96902416
KCNH2	rs11763131	chr7:150,642,044-150,675,402	AX-112993600
KCNH2	rs11763131	chr7:150,642,044-150,675,402	AX-113402324
KCNH2	rs3807374	chr7:150,642,044-150,675,402	AX-96911629
KCNH2	rs3807374	chr7:150,642,044-150,675,402	AX-96922612
KCNH2	rs4725984	chr7:150,642,044-150,675,402	AX-84652118
KCNH2	rs4725984	chr7:150,642,044-150,675,402	AX-95820782
KCNH2	rs3807373	chr7:150,642,044-150,675,402	AX-113407685
KCNH2	rs3807373	chr7:150,642,044-150,675,402	AX-113964184
KCNH2	rs3807372	chr7:150,642,044-150,675,402	AX-42143581
KCNH2	rs3778873	chr7:150,642,044-150,675,402	AX-42143583
KCNH2	rs3778872	chr7:150,642,044-150,675,402	AX-42143585
KCNH2	rs3778871	chr7:150,642,044-150,675,402	AX-36091143
KCNH2	rs3778870	chr7:150,642,044-150,675,402	AX-36091147
KCNH2	rs3778869	chr7:150,642,044-150,675,402	AX-36091149
KCNH2	rs3800779	chr7:150,642,044-150,675,402	AX-36091151
KCNH2	rs748693	chr7:150,642,044-150,675,402	AX-42143589
KCNH2	rs116065480	chr7:150,642,044-150,675,402	AX-38397259
KCNH2	---	chr7:150,642,044-150,675,402	AX-90080885
KCNH2	---	chr7:150,642,044-150,675,402	AX-90064213
KCNH2	---	chr7:150,642,044-150,675,402	AX-90056673
KCNH2	---	chr7:150,642,044-150,675,402	AX-90064164

KCNH2	---	chr7:150,642,044-150,675,402	AX-90029048
KCNH2	---	chr7:150,642,044-150,675,402	AX-90059757
KCNH2	---	chr7:150,642,044-150,675,402	AX-90031668
KCNH2	---	chr7:150,642,044-150,675,402	AX-90029760
KCNH2	---	chr7:150,642,044-150,675,402	AX-90027828
KCNH2	---	chr7:150,642,044-150,675,402	AX-90073920
KCNH2	---	chr7:150,642,044-150,675,402	AX-90059860
KCNH2	---	chr7:150,642,044-150,675,402	AX-90057678
KCNH2	---	chr7:150,642,044-150,675,402	AX-90066134
KCNH2	---	chr7:150,642,044-150,675,402	AX-90057594
KCNH2	---	chr7:150,642,044-150,675,402	AX-90066049
KCNH2	---	chr7:150,642,044-150,675,402	AX-90060874
KCNH2	---	chr7:150,642,044-150,675,402	AX-90062360
KCNH2	---	chr7:150,642,044-150,675,402	AX-90060208
KCNH2	---	chr7:150,642,044-150,675,402	AX-90032199
KCNH2	---	chr7:150,642,044-150,675,402	AX-90064271
KCNH2	---	chr7:150,642,044-150,675,402	AX-90059398
KCNH2	---	chr7:150,642,044-150,675,402	AX-90059625
KCNH2	---	chr7:150,642,044-150,675,402	AX-90028040
KCNH2	---	chr7:150,642,044-150,675,402	AX-90034468
KCNH2	---	chr7:150,642,044-150,675,402	AX-90058289
KCNH2	---	chr7:150,642,044-150,675,402	AX-90031151
KCNH2	---	chr7:150,642,044-150,675,402	AX-90028147
KCNH2	---	chr7:150,642,044-150,675,402	AX-90029466
KCNH2	---	chr7:150,642,044-150,675,402	AX-90036415
KCNH2	---	chr7:150,642,044-150,675,402	AX-90027394
KCNH2	---	chr7:150,642,044-150,675,402	AX-90029254
KCNH2	---	chr7:150,642,044-150,675,402	AX-90058133
KCNH2	---	chr7:150,642,044-150,675,402	AX-90064576
KCNH2	---	chr7:150,642,044-150,675,402	AX-90027503
KCNH2	---	chr7:150,642,044-150,675,402	AX-90059183
KCNH2	---	chr7:150,642,044-150,675,402	AX-90062406
KCNH2	---	chr7:150,642,044-150,675,402	AX-90036977
KCNH2	---	chr7:150,642,044-150,675,402	AX-90031687
KCNH2	---	chr7:150,642,044-150,675,402	AX-90056049
KCNH2	---	chr7:150,642,044-150,675,402	AX-90066099
KCNH2	---	chr7:150,642,044-150,675,402	AX-90064283
KCNH2	---	chr7:150,642,044-150,675,402	AX-90027823
KCNH2	---	chr7:150,642,044-150,675,402	AX-90061167
KCNH2	---	chr7:150,642,044-150,675,402	AX-90034498
KCNH2	---	chr7:150,642,044-150,675,402	AX-90035307
KCNH2	---	chr7:150,642,044-150,675,402	AX-90034584
KCNH2	---	chr7:150,642,044-150,675,402	AX-90034082
KCNH2	---	chr7:150,642,044-150,675,402	AX-90031834
KCNH2	---	chr7:150,642,044-150,675,402	AX-90036686
KCNH2	---	chr7:150,642,044-150,675,402	AX-90034522
KCNH2	---	chr7:150,642,044-150,675,402	AX-90031326
KCNH2	rs41309002	chr7:150,642,044-150,675,402	AX-36091159
KCNH2	rs3807370	chr7:150,642,044-150,675,402	AX-112998957
KCNH2	rs3807370	chr7:150,642,044-150,675,402	AX-113407687
KCNH2	rs1036145	chr7:150,642,044-150,675,402	AX-42143597
KCNH2	rs35277497	chr7:150,642,044-150,675,402	AX-96880943
KCNH2	---	chr7:150,642,044-150,675,402	AX-90080698
KCNH2	---	chr7:150,642,044-150,675,402	AX-90059502
KCNH2	---	chr7:150,642,044-150,675,402	AX-90056168

KCNH2	---	chr7:150,642,044-150,675,402	AX-90027129
KCNH2	rs189034123	chr7:150,642,044-150,675,402	AX-88788298
KCNJ2	---	chr17:68,164,814-68,176,183	AX-90062412
KCNJ2	---	chr17:68,164,814-68,176,183	AX-90056836
KCNJ2	rs104894580	chr17:68,164,814-68,176,183	AX-88797417
KCNJ2	---	chr17:68,164,814-68,176,183	AX-90055648
KCNJ2	---	chr17:68,164,814-68,176,183	AX-90055649
KCNJ2	rs104894575	chr17:68,164,814-68,176,183	AX-88797415
KCNJ2	---	chr17:68,164,814-68,176,183	AX-90055651
KCNJ2	---	chr17:68,164,814-68,176,183	AX-90036629
KCNJ2	---	chr17:68,164,814-68,176,183	AX-90026767
KCNJ2	---	chr17:68,164,814-68,176,183	AX-90058621
KCNJ2	---	chr17:68,164,814-68,176,183	AX-90055653
KCNJ2	---	chr17:68,164,814-68,176,183	AX-90036385
KCNJ2	---	chr17:68,164,814-68,176,183	AX-90026770
KCNJ2	rs147750704	chr17:68,164,814-68,176,183	AX-83234881
KCNJ2	---	chr17:68,164,814-68,176,183	AX-90076023
KCNJ2	---	chr17:68,164,814-68,176,183	AX-90057151
KCNJ2	---	chr17:68,164,814-68,176,183	AX-90055656
KCNJ2	---	chr17:68,164,814-68,176,183	AX-90055657
KCNJ2	---	chr17:68,164,814-68,176,183	AX-90026773
KCNJ2	---	chr17:68,164,814-68,176,183	AX-90026774
KCNJ2	---	chr17:68,164,814-68,176,183	AX-90063584
KCNJ2	---	chr17:68,164,814-68,176,183	AX-90026775
KCNJ2	---	chr17:68,164,814-68,176,183	AX-90035762
KCNJ2	---	chr17:68,164,814-68,176,183	AX-90055661
KCNJ2	---	chr17:68,164,814-68,176,183	AX-90026777
KCNJ2	---	chr17:68,164,814-68,176,183	AX-90051933
KCNJ2	rs104894584	chr17:68,164,814-68,176,183	AX-90070100
KCNJ2	rs104894581	chr17:68,164,814-68,176,183	AX-88754373
KCNJ2	---	chr17:68,164,814-68,176,183	AX-90055663
KCNJ2	---	chr17:68,164,814-68,176,183	AX-90026779
KCNJ2	---	chr17:68,164,814-68,176,183	AX-90055665
KCNJ2	---	chr17:68,164,814-68,176,183	AX-90055667
KCNJ2	rs104894578	chr17:68,164,814-68,176,183	AX-88754371
KCNJ2	---	chr17:68,164,814-68,176,183	AX-90055668
KCNJ2	---	chr17:68,164,814-68,176,183	AX-90063815
KCNJ2	---	chr17:68,164,814-68,176,183	AX-90026784
KCNJ2	---	chr17:68,164,814-68,176,183	AX-90026785
KCNJ2	---	chr17:68,164,814-68,176,183	AX-90056816
KCNJ2	---	chr17:68,164,814-68,176,183	AX-90033614
KCNJ2	rs104894582	chr17:68,164,814-68,176,183	AX-88754374
KCNJ2	---	chr17:68,164,814-68,176,183	AX-90055671
KCNJ2	---	chr17:68,164,814-68,176,183	AX-90026787
KCNJ2	---	chr17:68,164,814-68,176,183	AX-90055673
KCNJ2	---	chr17:68,164,814-68,176,183	AX-90055674
KCNJ2	---	chr17:68,164,814-68,176,183	AX-90055675
KCNJ2	---	chr17:68,164,814-68,176,183	AX-90042581
KCNJ2	---	chr17:68,164,814-68,176,183	AX-90032889
KCNJ2	rs144022753	chr17:68,164,814-68,176,183	AX-86649114
KCNJ2	rs141069645	chr17:68,164,814-68,176,183	AX-83167392
KCNJ5	rs34444372	chr11:128,761,313-128,787,951	AX-16517489
KCNJ5	rs61910641	chr11:128,761,313-128,787,951	AX-29921921
KCNJ5	rs7924416	chr11:128,761,313-128,787,951	AX-11654077
KCNJ5	rs76097649	chr11:128,761,313-128,787,951	AX-16517491

KCNJ5	rs2846674	chr11:128,761,313-128,787,951	AX-11417620
KCNJ5	rs2846700	chr11:128,761,313-128,787,951	AX-16517494
KCNJ5	rs76403033	chr11:128,761,313-128,787,951	AX-16517497
KCNJ5	rs78838391	chr11:128,761,313-128,787,951	AX-29921959
KCNJ5	rs4937389	chr11:128,761,313-128,787,951	AX-29921971
KCNJ5	rs138330651	chr11:128,761,313-128,787,951	AX-83413531
KCNJ5	rs78864180	chr11:128,761,313-128,787,951	AX-29922011
KCNJ5	rs4937390	chr11:128,761,313-128,787,951	AX-38991737
KCNJ5	rs11221510	chr11:128,761,313-128,787,951	AX-38991739
KCNJ5	rs6590357	chr11:128,761,313-128,787,951	AX-38991741
KCNJ5	---	chr11:128,761,313-128,787,951	AX-90031902
KCNJ5	---	chr11:128,761,313-128,787,951	AX-90058864
KCNJ5	---	chr11:128,761,313-128,787,951	AX-90060975
KCNJ5	rs7118824	chr11:128,761,313-128,787,951	AX-38991743
KCNJ5	rs1940455	chr11:128,761,313-128,787,951	AX-38991757
KCNQ1	---	chr11:2,466,221-2,870,340	AX-90065706
KCNQ1	rs397508108	chr11:2,466,221-2,870,340	AX-90074515
KCNQ1	---	chr11:2,466,221-2,870,340	AX-90034009
KCNQ1	---	chr11:2,466,221-2,870,340	AX-90036019
KCNQ1	---	chr11:2,466,221-2,870,340	AX-90060580
KCNQ1	rs120074191	chr11:2,466,221-2,870,340	AX-90069286
KCNQ1	---	chr11:2,466,221-2,870,340	AX-90077327
KCNQ1	---	chr11:2,466,221-2,870,340	AX-90059309
KCNQ1	---	chr11:2,466,221-2,870,340	AX-90056544
KCNQ1	rs112134401	chr11:2,466,221-2,870,340	AX-96804368
KCNQ1	rs112134401	chr11:2,466,221-2,870,340	AX-96823840
KCNQ1	rs78615661	chr11:2,466,221-2,870,340	AX-96877969
KCNQ1	rs78615661	chr11:2,466,221-2,870,340	AX-97351222
KCNQ1	rs76429041	chr11:2,466,221-2,870,340	AX-96924470
KCNQ1	rs76429041	chr11:2,466,221-2,870,340	AX-97469116
KCNQ1	rs12287029	chr11:2,466,221-2,870,340	AX-96561648
KCNQ1	rs12287029	chr11:2,466,221-2,870,340	AX-96645217
KCNQ1	rs12288463	chr11:2,466,221-2,870,340	AX-30006657
KCNQ1	rs111289280	chr11:2,466,221-2,870,340	AX-30006659
KCNQ1	rs7110663	chr11:2,466,221-2,870,340	AX-30006681
KCNQ1	rs7932717	chr11:2,466,221-2,870,340	AX-30006687
KCNQ1	rs2023818	chr11:2,466,221-2,870,340	AX-30006697
KCNQ1	rs11827153	chr11:2,466,221-2,870,340	AX-113403079
KCNQ1	rs74048614	chr11:2,466,221-2,870,340	AX-30006719
KCNQ1	rs11824286	chr11:2,466,221-2,870,340	AX-30006723
KCNQ1	rs7115494	chr11:2,466,221-2,870,340	AX-113963125
KCNQ1	rs67832597	chr11:2,466,221-2,870,340	AX-96783349
KCNQ1	---	chr11:2,466,221-2,870,340	AX-30006747
KCNQ1	rs112292464	chr11:2,466,221-2,870,340	AX-96783350
KCNQ1	rs112292464	chr11:2,466,221-2,870,340	AX-97421361
KCNQ1	rs34273392	chr11:2,466,221-2,870,340	AX-30006753
KCNQ1	rs67751613	chr11:2,466,221-2,870,340	AX-96881783
KCNQ1	rs67751613	chr11:2,466,221-2,870,340	AX-96883934
KCNQ1	rs800336	chr11:2,466,221-2,870,340	AX-39035879
KCNQ1	rs800337	chr11:2,466,221-2,870,340	AX-30006777
KCNQ1	rs800338	chr11:2,466,221-2,870,340	AX-30006781
KCNQ1	rs202212291	chr11:2,466,221-2,870,340	AX-156285290
KCNQ1	rs202212291	chr11:2,466,221-2,870,340	AX-96645310
KCNQ1	rs7945198	chr11:2,466,221-2,870,340	AX-30006795
KCNQ1	rs739677	chr11:2,466,221-2,870,340	AX-39035889

KCNQ1	rs2005693	chr11:2,466,221-2,870,340	AX-96084843
KCNQ1	rs2005693	chr11:2,466,221-2,870,340	AX-97352594
KCNQ1	rs201426302	chr11:2,466,221-2,870,340	AX-96644022
KCNQ1	rs5789256	chr11:2,466,221-2,870,340	AX-151160962
KCNQ1	rs5789256	chr11:2,466,221-2,870,340	AX-96933794
KCNQ1	rs74048621	chr11:2,466,221-2,870,340	AX-30006817
KCNQ1	rs12274567	chr11:2,466,221-2,870,340	AX-39035895
KCNQ1	rs1811815	chr11:2,466,221-2,870,340	AX-39035897
KCNQ1	rs926189	chr11:2,466,221-2,870,340	AX-112920257
KCNQ1	rs926189	chr11:2,466,221-2,870,340	AX-113959667
KCNQ1	rs34780268	chr11:2,466,221-2,870,340	AX-88736678
KCNQ1	rs34780268	chr11:2,466,221-2,870,340	AX-96768801
KCNQ1	rs10832090	chr11:2,466,221-2,870,340	AX-96561643
KCNQ1	rs112948720	chr11:2,466,221-2,870,340	AX-96560456
KCNQ1	rs112948720	chr11:2,466,221-2,870,340	AX-96644025
KCNQ1	rs59954955	chr11:2,466,221-2,870,340	AX-96563261
KCNQ1	rs59954955	chr11:2,466,221-2,870,340	AX-96646830
KCNQ1	rs56714380	chr11:2,466,221-2,870,340	AX-96560123
KCNQ1	rs56714380	chr11:2,466,221-2,870,340	AX-96643692
KCNQ1	rs10741622	chr11:2,466,221-2,870,340	AX-96563948
KCNQ1	rs10741622	chr11:2,466,221-2,870,340	AX-96647517
KCNQ1	rs57209697	chr11:2,466,221-2,870,340	AX-96565417
KCNQ1	rs57209697	chr11:2,466,221-2,870,340	AX-96648986
KCNQ1	rs58807875	chr11:2,466,221-2,870,340	AX-122166023
KCNQ1	rs7129506	chr11:2,466,221-2,870,340	AX-96564849
KCNQ1	rs34565276	chr11:2,466,221-2,870,340	AX-13016117
KCNQ1	rs60404945	chr11:2,466,221-2,870,340	AX-96645438
KCNQ1	rs56403874	chr11:2,466,221-2,870,340	AX-156279806
KCNQ1	rs12271931	chr11:2,466,221-2,870,340	AX-96561786
KCNQ1	rs12271931	chr11:2,466,221-2,870,340	AX-96645355
KCNQ1	rs73417589	chr11:2,466,221-2,870,340	AX-96959054
KCNQ1	rs73417589	chr11:2,466,221-2,870,340	AX-97269031
KCNQ1	rs76760338	chr11:2,466,221-2,870,340	AX-156270457
KCNQ1	rs76760338	chr11:2,466,221-2,870,340	AX-156289275
KCNQ1	rs78937903	chr11:2,466,221-2,870,340	AX-30006985
KCNQ1	rs11022922	chr11:2,466,221-2,870,340	AX-39035985
KCNQ1	rs74048626	chr11:2,466,221-2,870,340	AX-96961092
KCNQ1	rs74048626	chr11:2,466,221-2,870,340	AX-97433721
KCNQ1	rs73417596	chr11:2,466,221-2,870,340	AX-30007049
KCNQ1	rs7948689	chr11:2,466,221-2,870,340	AX-96870306
KCNQ1	rs7948689	chr11:2,466,221-2,870,340	AX-96893885
KCNQ1	rs7948693	chr11:2,466,221-2,870,340	AX-96809907
KCNQ1	rs7951832	chr11:2,466,221-2,870,340	AX-39036007
KCNQ1	rs12574110	chr11:2,466,221-2,870,340	AX-96836571
KCNQ1	rs12574110	chr11:2,466,221-2,870,340	AX-96860032
KCNQ1	rs16928285	chr11:2,466,221-2,870,340	AX-39036019
KCNQ1	rs78427034	chr11:2,466,221-2,870,340	AX-30007133
KCNQ1	rs3864884	chr11:2,466,221-2,870,340	AX-39036027
KCNQ1	rs112600033	chr11:2,466,221-2,870,340	AX-30007201
KCNQ1	rs2075873	chr11:2,466,221-2,870,340	AX-39036049
KCNQ1	rs2074238	chr11:2,466,221-2,870,340	AX-83391590
KCNQ1	rs2074239	chr11:2,466,221-2,870,340	AX-39036087
KCNQ1	rs7939542	chr11:2,466,221-2,870,340	AX-39036091
KCNQ1	rs76503999	chr11:2,466,221-2,870,340	AX-96993607
KCNQ1	rs76503999	chr11:2,466,221-2,870,340	AX-97303415

KCNQ1	rs16928297	chr11:2,466,221-2,870,340	AX-39036107
KCNQ1	rs112344603	chr11:2,466,221-2,870,340	AX-37510487
KCNQ1	rs113127436	chr11:2,466,221-2,870,340	AX-156265987
KCNQ1	rs113127436	chr11:2,466,221-2,870,340	AX-156286649
KCNQ1	rs12280952	chr11:2,466,221-2,870,340	AX-30007443
KCNQ1	rs12296050	chr11:2,466,221-2,870,340	AX-16566350
KCNQ1	rs74771060	chr11:2,466,221-2,870,340	AX-16566375
KCNQ1	rs58560659	chr11:2,466,221-2,870,340	AX-96791297
KCNQ1	rs58560659	chr11:2,466,221-2,870,340	AX-97348523
KCNQ1	rs58558735	chr11:2,466,221-2,870,340	AX-30007585
KCNQ1	rs11821100	chr11:2,466,221-2,870,340	AX-30007597
KCNQ1	rs58770749	chr11:2,466,221-2,870,340	AX-16566424
KCNQ1	rs60807509	chr11:2,466,221-2,870,340	AX-30007609
KCNQ1	rs61873498	chr11:2,466,221-2,870,340	AX-30007611
KCNQ1	rs56075656	chr11:2,466,221-2,870,340	AX-16566435
KCNQ1	rs111903332	chr11:2,466,221-2,870,340	AX-30007653
KCNQ1	rs11604259	chr11:2,466,221-2,870,340	AX-96938042
KCNQ1	rs11604259	chr11:2,466,221-2,870,340	AX-97429368
KCNQ1	rs11601717	chr11:2,466,221-2,870,340	AX-96984168
KCNQ1	rs11601717	chr11:2,466,221-2,870,340	AX-96986293
KCNQ1	rs11604319	chr11:2,466,221-2,870,340	AX-96981965
KCNQ1	rs28649383	chr11:2,466,221-2,870,340	AX-97339893
KCNQ1	rs28724284	chr11:2,466,221-2,870,340	AX-120741853
KCNQ1	rs28724284	chr11:2,466,221-2,870,340	AX-122991447
KCNQ1	rs36093649	chr11:2,466,221-2,870,340	AX-96875880
KCNQ1	rs36093649	chr11:2,466,221-2,870,340	AX-96886628
KCNQ1	rs60009089	chr11:2,466,221-2,870,340	AX-96745768
KCNQ1	rs58680149	chr11:2,466,221-2,870,340	AX-144899885
KCNQ1	rs59809566	chr11:2,466,221-2,870,340	AX-156286650
KCNQ1	rs76534570	chr11:2,466,221-2,870,340	AX-16566497
KCNQ1	rs34657034	chr11:2,466,221-2,870,340	AX-156284400
KCNQ1	rs11604486	chr11:2,466,221-2,870,340	AX-112923818
KCNQ1	---	chr11:2,466,221-2,870,340	AX-113964885
KCNQ1	rs11604980	chr11:2,466,221-2,870,340	AX-30007737
KCNQ1	rs7122546	chr11:2,466,221-2,870,340	AX-112925549
KCNQ1	rs7122546	chr11:2,466,221-2,870,340	AX-113964886
KCNQ1	rs7122557	chr11:2,466,221-2,870,340	AX-112999612
KCNQ1	rs7122557	chr11:2,466,221-2,870,340	AX-113408371
KCNQ1	rs7119581	chr11:2,466,221-2,870,340	AX-30007757
KCNQ1	rs7103046	chr11:2,466,221-2,870,340	AX-113408372
KCNQ1	rs7126330	chr11:2,466,221-2,870,340	AX-113408373
KCNQ1	rs7126330	chr11:2,466,221-2,870,340	AX-113964888
KCNQ1	rs16928325	chr11:2,466,221-2,870,340	AX-39036295
KCNQ1	rs3815063	chr11:2,466,221-2,870,340	AX-16566550
KCNQ1	rs3815064	chr11:2,466,221-2,870,340	AX-113408374
KCNQ1	rs3815064	chr11:2,466,221-2,870,340	AX-113482214
KCNQ1	rs5028646	chr11:2,466,221-2,870,340	AX-39036325
KCNQ1	rs75293264	chr11:2,466,221-2,870,340	AX-16566571
KCNQ1	rs16928333	chr11:2,466,221-2,870,340	AX-39036357
KCNQ1	rs2015013	chr11:2,466,221-2,870,340	AX-96971342
KCNQ1	rs2015013	chr11:2,466,221-2,870,340	AX-96999434
KCNQ1	rs718579	chr11:2,466,221-2,870,340	AX-113889345
KCNQ1	rs35869609	chr11:2,466,221-2,870,340	AX-96830761
KCNQ1	rs200616757	chr11:2,466,221-2,870,340	AX-96562889
KCNQ1	rs200616757	chr11:2,466,221-2,870,340	AX-96646458

KCNQ1	rs10832126	chr11:2,466,221-2,870,340	AX-112999619
KCNQ1	rs10832126	chr11:2,466,221-2,870,340	AX-113891028
KCNQ1	---	chr11:2,466,221-2,870,340	AX-16566625
KCNQ1	rs2079046	chr11:2,466,221-2,870,340	AX-16566646
KCNQ1	rs12576156	chr11:2,466,221-2,870,340	AX-39036393
KCNQ1	rs757092	chr11:2,466,221-2,870,340	AX-39036399
KCNQ1	rs12288422	chr11:2,466,221-2,870,340	AX-96880016
KCNQ1	rs60502601	chr11:2,466,221-2,870,340	AX-96987783
KCNQ1	rs60502601	chr11:2,466,221-2,870,340	AX-97367066
KCNQ1	rs34847331	chr11:2,466,221-2,870,340	AX-148131020
KCNQ1	rs34847331	chr11:2,466,221-2,870,340	AX-156265989
KCNQ1	rs150893539	chr11:2,466,221-2,870,340	AX-156285106
KCNQ1	rs10832131	chr11:2,466,221-2,870,340	AX-112925552
KCNQ1	rs10832131	chr11:2,466,221-2,870,340	AX-112999620
KCNQ1	rs5789257	chr11:2,466,221-2,870,340	AX-11547311
KCNQ1	rs12576239	chr11:2,466,221-2,870,340	AX-16566753
KCNQ1	rs11022996	chr11:2,466,221-2,870,340	AX-16566754
KCNQ1	rs10832134	chr11:2,466,221-2,870,340	AX-11125418
KCNQ1	rs11022998	chr11:2,466,221-2,870,340	AX-16566769
KCNQ1	rs56403720	chr11:2,466,221-2,870,340	AX-16566822
KCNQ1	rs144866270	chr11:2,466,221-2,870,340	AX-16566837
KCNQ1	rs144866270	chr11:2,466,221-2,870,340	AX-96743565
KCNQ1	rs12801583	chr11:2,466,221-2,870,340	AX-30008219
KCNQ1	rs4337018	chr11:2,466,221-2,870,340	AX-156265991
KCNQ1	rs4578370	chr11:2,466,221-2,870,340	AX-11512289
KCNQ1	rs73419519	chr11:2,466,221-2,870,340	AX-30008277
KCNQ1	rs113255961	chr11:2,466,221-2,870,340	AX-30008281
KCNQ1	rs7106063	chr11:2,466,221-2,870,340	AX-30008291
KCNQ1	rs7105460	chr11:2,466,221-2,870,340	AX-123022895
KCNQ1	rs7105460	chr11:2,466,221-2,870,340	AX-123022896
KCNQ1	rs2188956	chr11:2,466,221-2,870,340	AX-30008323
KCNQ1	rs7947797	chr11:2,466,221-2,870,340	AX-96909616
KCNQ1	rs7947797	chr11:2,466,221-2,870,340	AX-96924889
KCNQ1	rs75421421	chr11:2,466,221-2,870,340	AX-97255812
KCNQ1	rs75082082	chr11:2,466,221-2,870,340	AX-156295395
KCNQ1	rs35955303	chr11:2,466,221-2,870,340	AX-120696694
KCNQ1	rs35955303	chr11:2,466,221-2,870,340	AX-156295396
KCNQ1	rs7125401	chr11:2,466,221-2,870,340	AX-30008367
KCNQ1	---	chr11:2,466,221-2,870,340	AX-97418171
KCNQ1	---	chr11:2,466,221-2,870,340	AX-96560894
KCNQ1	---	chr11:2,466,221-2,870,340	AX-96644463
KCNQ1	rs2079043	chr11:2,466,221-2,870,340	AX-30008427
KCNQ1	---	chr11:2,466,221-2,870,340	AX-30008431
KCNQ1	rs4929989	chr11:2,466,221-2,870,340	AX-113480493
KCNQ1	rs1080015	chr11:2,466,221-2,870,340	AX-39036601
KCNQ1	rs1080016	chr11:2,466,221-2,870,340	AX-156286651
KCNQ1	rs1080017	chr11:2,466,221-2,870,340	AX-156265993
KCNQ1	rs1080017	chr11:2,466,221-2,870,340	AX-156286652
KCNQ1	rs112638737	chr11:2,466,221-2,870,340	AX-156283666
KCNQ1	rs112638737	chr11:2,466,221-2,870,340	AX-96770973
KCNQ1	rs11023040	chr11:2,466,221-2,870,340	AX-96646679
KCNQ1	rs7928116	chr11:2,466,221-2,870,340	AX-156279809
KCNQ1	rs7928116	chr11:2,466,221-2,870,340	AX-156295398
KCNQ1	rs179392	chr11:2,466,221-2,870,340	AX-113912022
KCNQ1	rs179393	chr11:2,466,221-2,870,340	AX-30008523

KCNQ1	rs179394	chr11:2,466,221-2,870,340	AX-30008529
KCNQ1	rs4930114	chr11:2,466,221-2,870,340	AX-113503520
KCNQ1	rs4930114	chr11:2,466,221-2,870,340	AX-113912020
KCNQ1	rs179395	chr11:2,466,221-2,870,340	AX-151214667
KCNQ1	rs179395	chr11:2,466,221-2,870,340	AX-156283667
KCNQ1	rs179396	chr11:2,466,221-2,870,340	AX-113020755
KCNQ1	rs179396	chr11:2,466,221-2,870,340	AX-113912023
KCNQ1	rs2741959	chr11:2,466,221-2,870,340	AX-96943595
KCNQ1	rs2741959	chr11:2,466,221-2,870,340	AX-97481385
KCNQ1	rs2741960	chr11:2,466,221-2,870,340	AX-96867162
KCNQ1	rs2741960	chr11:2,466,221-2,870,340	AX-96895127
KCNQ1	rs12574553	chr11:2,466,221-2,870,340	AX-96876997
KCNQ1	rs144201211	chr11:2,466,221-2,870,340	AX-96561246
KCNQ1	rs7119560	chr11:2,466,221-2,870,340	AX-97316671
KCNQ1	rs7119560	chr11:2,466,221-2,870,340	AX-97430535
KCNQ1	rs7119686	chr11:2,466,221-2,870,340	AX-112994385
KCNQ1	rs179401	chr11:2,466,221-2,870,340	AX-148353620
KCNQ1	rs179401	chr11:2,466,221-2,870,340	AX-156279810
KCNQ1	rs7926983	chr11:2,466,221-2,870,340	AX-156279811
KCNQ1	rs7926983	chr11:2,466,221-2,870,340	AX-156295399
KCNQ1	rs7945539	chr11:2,466,221-2,870,340	AX-156279812
KCNQ1	rs7945539	chr11:2,466,221-2,870,340	AX-156295400
KCNQ1	rs2188199	chr11:2,466,221-2,870,340	AX-96803925
KCNQ1	rs2188199	chr11:2,466,221-2,870,340	AX-96823409
KCNQ1	rs2188200	chr11:2,466,221-2,870,340	AX-156295401
KCNQ1	rs2188202	chr11:2,466,221-2,870,340	AX-156279814
KCNQ1	rs2188202	chr11:2,466,221-2,870,340	AX-156295402
KCNQ1	rs76995564	chr11:2,466,221-2,870,340	AX-156279815
KCNQ1	rs76995564	chr11:2,466,221-2,870,340	AX-156295403
KCNQ1	rs10766136	chr11:2,466,221-2,870,340	AX-113403080
KCNQ1	rs10766136	chr11:2,466,221-2,870,340	AX-113476972
KCNQ1	rs114847525	chr11:2,466,221-2,870,340	AX-96834328
KCNQ1	rs114847525	chr11:2,466,221-2,870,340	AX-96861913
KCNQ1	rs7120338	chr11:2,466,221-2,870,340	AX-96767859
KCNQ1	rs7120338	chr11:2,466,221-2,870,340	AX-96791565
KCNQ1	rs7106498	chr11:2,466,221-2,870,340	AX-112946496
KCNQ1	rs10832175	chr11:2,466,221-2,870,340	AX-96886863
KCNQ1	rs10832175	chr11:2,466,221-2,870,340	AX-97348032
KCNQ1	rs10832179	chr11:2,466,221-2,870,340	AX-113020757
KCNQ1	rs10832179	chr11:2,466,221-2,870,340	AX-113912024
KCNQ1	rs1116249	chr11:2,466,221-2,870,340	AX-97287019
KCNQ1	rs1116249	chr11:2,466,221-2,870,340	AX-97400153
KCNQ1	rs11517737	chr11:2,466,221-2,870,340	AX-96790058
KCNQ1	rs11517737	chr11:2,466,221-2,870,340	AX-97308597
KCNQ1	rs11511852	chr11:2,466,221-2,870,340	AX-13539000
KCNQ1	rs11511852	chr11:2,466,221-2,870,340	AX-96808340
KCNQ1	rs148324871	chr11:2,466,221-2,870,340	AX-96829427
KCNQ1	rs35474939	chr11:2,466,221-2,870,340	AX-96726965
KCNQ1	rs75753584	chr11:2,466,221-2,870,340	AX-148895673
KCNQ1	rs75753584	chr11:2,466,221-2,870,340	AX-156270458
KCNQ1	rs179405	chr11:2,466,221-2,870,340	AX-96869364
KCNQ1	rs179405	chr11:2,466,221-2,870,340	AX-97307842
KCNQ1	rs7111657	chr11:2,466,221-2,870,340	AX-96857678
KCNQ1	rs73419534	chr11:2,466,221-2,870,340	AX-96889198
KCNQ1	rs73419534	chr11:2,466,221-2,870,340	AX-97471987

KCNQ1	rs2157761	chr11:2,466,221-2,870,340	AX-30009057
KCNQ1	rs179406	chr11:2,466,221-2,870,340	AX-11344292
KCNQ1	rs2106844	chr11:2,466,221-2,870,340	AX-96561253
KCNQ1	rs59732535	chr11:2,466,221-2,870,340	AX-16567497
KCNQ1	rs4930117	chr11:2,466,221-2,870,340	AX-96565331
KCNQ1	rs4930117	chr11:2,466,221-2,870,340	AX-96648900
KCNQ1	rs179407	chr11:2,466,221-2,870,340	AX-11344294
KCNQ1	rs2106203	chr11:2,466,221-2,870,340	AX-30009111
KCNQ1	rs2106204	chr11:2,466,221-2,870,340	AX-96937092
KCNQ1	rs2106204	chr11:2,466,221-2,870,340	AX-96964788
KCNQ1	rs56103600	chr11:2,466,221-2,870,340	AX-16567507
KCNQ1	rs61026321	chr11:2,466,221-2,870,340	AX-16567508
KCNQ1	rs179409	chr11:2,466,221-2,870,340	AX-16567525
KCNQ1	rs11023094	chr11:2,466,221-2,870,340	AX-11138560
KCNQ1	rs35574774	chr11:2,466,221-2,870,340	AX-30009163
KCNQ1	rs11023096	chr11:2,466,221-2,870,340	AX-11138561
KCNQ1	rs4929992	chr11:2,466,221-2,870,340	AX-39037009
KCNQ1	rs3819506	chr11:2,466,221-2,870,340	AX-12562743
KCNQ1	rs3819508	chr11:2,466,221-2,870,340	AX-30009201
KCNQ1	rs3819509	chr11:2,466,221-2,870,340	AX-30009203
KCNQ1	rs12283619	chr11:2,466,221-2,870,340	AX-96840815
KCNQ1	rs12283619	chr11:2,466,221-2,870,340	AX-97413645
KCNQ1	rs3819510	chr11:2,466,221-2,870,340	AX-96968331
KCNQ1	rs3819510	chr11:2,466,221-2,870,340	AX-97000703
KCNQ1	rs3819511	chr11:2,466,221-2,870,340	AX-11483174
KCNQ1	rs3819512	chr11:2,466,221-2,870,340	AX-96947906
KCNQ1	rs3819512	chr11:2,466,221-2,870,340	AX-96954211
KCNQ1	rs3819513	chr11:2,466,221-2,870,340	AX-96815978
KCNQ1	rs3819513	chr11:2,466,221-2,870,340	AX-97475286
KCNQ1	rs2237864	chr11:2,466,221-2,870,340	AX-30009287
KCNQ1	rs2237865	chr11:2,466,221-2,870,340	AX-96877657
KCNQ1	rs2237865	chr11:2,466,221-2,870,340	AX-97447677
KCNQ1	rs2237866	chr11:2,466,221-2,870,340	AX-12522212
KCNQ1	rs2012618	chr11:2,466,221-2,870,340	AX-11359847
KCNQ1	rs200596173	chr11:2,466,221-2,870,340	AX-97432800
KCNQ1	rs2097618	chr11:2,466,221-2,870,340	AX-96813449
KCNQ1	rs2097618	chr11:2,466,221-2,870,340	AX-96815565
KCNQ1	rs2012360	chr11:2,466,221-2,870,340	AX-96968561
KCNQ1	---	chr11:2,466,221-2,870,340	AX-16567677
KCNQ1	rs7952222	chr11:2,466,221-2,870,340	AX-112920259
KCNQ1	rs7952222	chr11:2,466,221-2,870,340	AX-113476973
KCNQ1	rs80056995	chr11:2,466,221-2,870,340	AX-30009419
KCNQ1	rs16928363	chr11:2,466,221-2,870,340	AX-11290497
KCNQ1	rs909413	chr11:2,466,221-2,870,340	AX-112920260
KCNQ1	rs874185	chr11:2,466,221-2,870,340	AX-97279673
KCNQ1	rs2011766	chr11:2,466,221-2,870,340	AX-39037081
KCNQ1	rs73419557	chr11:2,466,221-2,870,340	AX-30009459
KCNQ1	rs2011750	chr11:2,466,221-2,870,340	AX-39037083
KCNQ1	rs7924915	chr11:2,466,221-2,870,340	AX-113476975
KCNQ1	rs7924915	chr11:2,466,221-2,870,340	AX-113885780
KCNQ1	rs2237867	chr11:2,466,221-2,870,340	AX-30009533
KCNQ1	rs2237868	chr11:2,466,221-2,870,340	AX-30009541
KCNQ1	rs140146748	chr11:2,466,221-2,870,340	AX-96761399
KCNQ1	rs74847994	chr11:2,466,221-2,870,340	AX-156279816
KCNQ1	rs74847994	chr11:2,466,221-2,870,340	AX-156295404

KCNQ1	rs77307322	chr11:2,466,221-2,870,340	AX-96949210
KCNQ1	rs77307322	chr11:2,466,221-2,870,340	AX-96951328
KCNQ1	rs16928366	chr11:2,466,221-2,870,340	AX-96755733
KCNQ1	rs6578270	chr11:2,466,221-2,870,340	AX-30009559
KCNQ1	rs74899747	chr11:2,466,221-2,870,340	AX-30009563
KCNQ1	rs74392867	chr11:2,466,221-2,870,340	AX-30009569
KCNQ1	rs2237869	chr11:2,466,221-2,870,340	AX-96561656
KCNQ1	rs2237869	chr11:2,466,221-2,870,340	AX-96645225
KCNQ1	---	chr11:2,466,221-2,870,340	AX-37510515
KCNQ1	---	chr11:2,466,221-2,870,340	AX-97361556
KCNQ1	rs2237871	chr11:2,466,221-2,870,340	AX-113408381
KCNQ1	rs2237871	chr11:2,466,221-2,870,340	AX-113964892
KCNQ1	rs2237872	chr11:2,466,221-2,870,340	AX-96645530
KCNQ1	rs74048650	chr11:2,466,221-2,870,340	AX-156289276
KCNQ1	---	chr11:2,466,221-2,870,340	AX-113482220
KCNQ1	---	chr11:2,466,221-2,870,340	AX-113891029
KCNQ1	rs736610	chr11:2,466,221-2,870,340	AX-96564030
KCNQ1	rs757085	chr11:2,466,221-2,870,340	AX-96840998
KCNQ1	rs757085	chr11:2,466,221-2,870,340	AX-96855954
KCNQ1	rs729980	chr11:2,466,221-2,870,340	AX-96784291
KCNQ1	rs729980	chr11:2,466,221-2,870,340	AX-97357006
KCNQ1	rs729979	chr11:2,466,221-2,870,340	AX-112924590
KCNQ1	rs729979	chr11:2,466,221-2,870,340	AX-113481271
KCNQ1	rs73419575	chr11:2,466,221-2,870,340	AX-156279817
KCNQ1	rs73419575	chr11:2,466,221-2,870,340	AX-156295405
KCNQ1	rs10741643	chr11:2,466,221-2,870,340	AX-39037185
KCNQ1	rs1005135	chr11:2,466,221-2,870,340	AX-30009659
KCNQ1	rs1005623	chr11:2,466,221-2,870,340	AX-96815177
KCNQ1	rs72847655	chr11:2,466,221-2,870,340	AX-30009679
KCNQ1	---	chr11:2,466,221-2,870,340	AX-96560265
KCNQ1	rs35973912	chr11:2,466,221-2,870,340	AX-30009695
KCNQ1	rs146122513	chr11:2,466,221-2,870,340	AX-96866696
KCNQ1	rs34164775	chr11:2,466,221-2,870,340	AX-96941469
KCNQ1	rs11826873	chr11:2,466,221-2,870,340	AX-96563867
KCNQ1	rs11826873	chr11:2,466,221-2,870,340	AX-96647436
KCNQ1	rs7110139	chr11:2,466,221-2,870,340	AX-30009705
KCNQ1	rs72847658	chr11:2,466,221-2,870,340	AX-96565436
KCNQ1	rs2898873	chr11:2,466,221-2,870,340	AX-30009729
KCNQ1	rs7935227	chr11:2,466,221-2,870,340	AX-39037209
KCNQ1	rs56331974	chr11:2,466,221-2,870,340	AX-30009749
KCNQ1	rs79295543	chr11:2,466,221-2,870,340	AX-30009755
KCNQ1	rs200988578	chr11:2,466,221-2,870,340	AX-96994243
KCNQ1	rs10400365	chr11:2,466,221-2,870,340	AX-30009763
KCNQ1	rs73419593	chr11:2,466,221-2,870,340	AX-156265994
KCNQ1	rs73419593	chr11:2,466,221-2,870,340	AX-156286653
KCNQ1	rs73419595	chr11:2,466,221-2,870,340	AX-30009777
KCNQ1	rs77059665	chr11:2,466,221-2,870,340	AX-156265995
KCNQ1	rs77059665	chr11:2,466,221-2,870,340	AX-156286654
KCNQ1	rs150949289	chr11:2,466,221-2,870,340	AX-96813387
KCNQ1	rs149377170	chr11:2,466,221-2,870,340	AX-151137338
KCNQ1	rs77686561	chr11:2,466,221-2,870,340	AX-30009843
KCNQ1	rs2283150	chr11:2,466,221-2,870,340	AX-96953475
KCNQ1	rs2283151	chr11:2,466,221-2,870,340	AX-30009869
KCNQ1	rs4930119	chr11:2,466,221-2,870,340	AX-39037255
KCNQ1	rs150878926	chr11:2,466,221-2,870,340	AX-97411972

KCNQ1	rs7111699	chr11:2,466,221-2,870,340	AX-96774631
KCNQ1	rs7111699	chr11:2,466,221-2,870,340	AX-96785402
KCNQ1	rs4930120	chr11:2,466,221-2,870,340	AX-88783175
KCNQ1	rs4930120	chr11:2,466,221-2,870,340	AX-96904070
KCNQ1	rs7122214	chr11:2,466,221-2,870,340	AX-92430257
KCNQ1	rs7122214	chr11:2,466,221-2,870,340	AX-96965175
KCNQ1	rs7122318	chr11:2,466,221-2,870,340	AX-11608236
KCNQ1	rs143820001	chr11:2,466,221-2,870,340	AX-148860779
KCNQ1	rs143820001	chr11:2,466,221-2,870,340	AX-92379004
KCNQ1	rs2283152	chr11:2,466,221-2,870,340	AX-107885436
KCNQ1	rs2283152	chr11:2,466,221-2,870,340	AX-113960358
KCNQ1	rs2283153	chr11:2,466,221-2,870,340	AX-96884694
KCNQ1	rs117305030	chr11:2,466,221-2,870,340	AX-37510527
KCNQ1	rs1010173	chr11:2,466,221-2,870,340	AX-97415696
KCNQ1	rs2283154	chr11:2,466,221-2,870,340	AX-96888829
KCNQ1	rs2283154	chr11:2,466,221-2,870,340	AX-97317538
KCNQ1	rs573946448	chr11:2,466,221-2,870,340	AX-151441171
KCNQ1	rs573946448	chr11:2,466,221-2,870,340	AX-156286195
KCNQ1	rs7952607	chr11:2,466,221-2,870,340	AX-156270460
KCNQ1	rs7952607	chr11:2,466,221-2,870,340	AX-156289277
KCNQ1	rs2299616	chr11:2,466,221-2,870,340	AX-30010015
KCNQ1	rs115332873	chr11:2,466,221-2,870,340	AX-156278489
KCNQ1	rs2299617	chr11:2,466,221-2,870,340	AX-39037339
KCNQ1	rs179426	chr11:2,466,221-2,870,340	AX-39037351
KCNQ1	rs141747786	chr11:2,466,221-2,870,340	AX-96876057
KCNQ1	rs141747786	chr11:2,466,221-2,870,340	AX-97447749
KCNQ1	rs76361309	chr11:2,466,221-2,870,340	AX-96908893
KCNQ1	rs76361309	chr11:2,466,221-2,870,340	AX-97298797
KCNQ1	rs28730663	chr11:2,466,221-2,870,340	AX-30010157
KCNQ1	---	chr11:2,466,221-2,870,340	AX-90034908
KCNQ1	---	chr11:2,466,221-2,870,340	AX-86615252
KCNQ1	---	chr11:2,466,221-2,870,340	AX-90062322
KCNQ1	---	chr11:2,466,221-2,870,340	AX-90059884
KCNQ1	---	chr11:2,466,221-2,870,340	AX-90045608
KCNQ1	---	chr11:2,466,221-2,870,340	AX-90062842
KCNQ1	---	chr11:2,466,221-2,870,340	AX-86666855
KCNQ1	rs120074192	chr11:2,466,221-2,870,340	AX-88752648
KCNQ1	---	chr11:2,466,221-2,870,340	AX-90055496
KCNQ1	---	chr11:2,466,221-2,870,340	AX-90064144
KCNQ1	---	chr11:2,466,221-2,870,340	AX-30010163
KCNQ1	---	chr11:2,466,221-2,870,340	AX-90059604
KCNQ1	---	chr11:2,466,221-2,870,340	AX-90026612
KCNQ1	---	chr11:2,466,221-2,870,340	AX-90036520
KCNQ1	rs397508110	chr11:2,466,221-2,870,340	AX-90079144
KCNQ1	rs143709408	chr11:2,466,221-2,870,340	AX-83344090
KCNQ1	---	chr11:2,466,221-2,870,340	AX-90057004
KCNQ1	---	chr11:2,466,221-2,870,340	AX-90059217
KCNQ1	---	chr11:2,466,221-2,870,340	AX-90059317
KCNQ1	---	chr11:2,466,221-2,870,340	AX-90061265
KCNQ1	rs199528268	chr11:2,466,221-2,870,340	AX-96564799
KCNQ1	rs199528268	chr11:2,466,221-2,870,340	AX-96648368
KCNQ1	rs179428	chr11:2,466,221-2,870,340	AX-39037437
KCNQ1	rs179429	chr11:2,466,221-2,870,340	AX-39037441
KCNQ1	rs179430	chr11:2,466,221-2,870,340	AX-39037445
KCNQ1	rs146130910	chr11:2,466,221-2,870,340	AX-96830779

KCNQ1	rs146130910	chr11:2,466,221-2,870,340	AX-97330087
KCNQ1	rs1557647	chr11:2,466,221-2,870,340	AX-39037459
KCNQ1	rs179431	chr11:2,466,221-2,870,340	AX-39037471
KCNQ1	rs7119884	chr11:2,466,221-2,870,340	AX-39037475
KCNQ1	rs2522006	chr11:2,466,221-2,870,340	AX-113885781
KCNQ1	rs2522007	chr11:2,466,221-2,870,340	AX-96648983
KCNQ1	rs2522008	chr11:2,466,221-2,870,340	AX-96564129
KCNQ1	rs11823023	chr11:2,466,221-2,870,340	AX-96976956
KCNQ1	rs11823023	chr11:2,466,221-2,870,340	AX-97474623
KCNQ1	rs72847667	chr11:2,466,221-2,870,340	AX-30010345
KCNQ1	rs111815403	chr11:2,466,221-2,870,340	AX-30010347
KCNQ1	rs179432	chr11:2,466,221-2,870,340	AX-30010357
KCNQ1	rs179433	chr11:2,466,221-2,870,340	AX-30010359
KCNQ1	rs179434	chr11:2,466,221-2,870,340	AX-30010361
KCNQ1	rs179435	chr11:2,466,221-2,870,340	AX-96853558
KCNQ1	rs179435	chr11:2,466,221-2,870,340	AX-97358518
KCNQ1	rs179436	chr11:2,466,221-2,870,340	AX-30010385
KCNQ1	rs179437	chr11:2,466,221-2,870,340	AX-30010445
KCNQ1	rs2074247	chr11:2,466,221-2,870,340	AX-96563751
KCNQ1	rs2074247	chr11:2,466,221-2,870,340	AX-96647320
KCNQ1	rs72847668	chr11:2,466,221-2,870,340	AX-30010473
KCNQ1	rs2074248	chr11:2,466,221-2,870,340	AX-30010475
KCNQ1	rs757086	chr11:2,466,221-2,870,340	AX-84661815
KCNQ1	rs56169764	chr11:2,466,221-2,870,340	AX-96750310
KCNQ1	rs56169764	chr11:2,466,221-2,870,340	AX-97385429
KCNQ1	rs112818110	chr11:2,466,221-2,870,340	AX-30010529
KCNQ1	rs72847670	chr11:2,466,221-2,870,340	AX-30010561
KCNQ1	rs72847671	chr11:2,466,221-2,870,340	AX-30010565
KCNQ1	rs115894051	chr11:2,466,221-2,870,340	AX-37510541
KCNQ1	rs58978657	chr11:2,466,221-2,870,340	AX-30010649
KCNQ1	rs72847673	chr11:2,466,221-2,870,340	AX-148807367
KCNQ1	rs72847673	chr11:2,466,221-2,870,340	AX-156265996
KCNQ1	rs75458755	chr11:2,466,221-2,870,340	AX-151421214
KCNQ1	rs75458755	chr11:2,466,221-2,870,340	AX-156265997
KCNQ1	rs10832270	chr11:2,466,221-2,870,340	AX-96561688
KCNQ1	rs10832270	chr11:2,466,221-2,870,340	AX-96645257
KCNQ1	rs10832272	chr11:2,466,221-2,870,340	AX-96560209
KCNQ1	rs10832272	chr11:2,466,221-2,870,340	AX-96643778
KCNQ1	rs72847674	chr11:2,466,221-2,870,340	AX-30010685
KCNQ1	rs179440	chr11:2,466,221-2,870,340	AX-96772647
KCNQ1	rs179440	chr11:2,466,221-2,870,340	AX-96783429
KCNQ1	rs60705296	chr11:2,466,221-2,870,340	AX-96831420
KCNQ1	rs60705296	chr11:2,466,221-2,870,340	AX-97364607
KCNQ1	rs72847675	chr11:2,466,221-2,870,340	AX-156295406
KCNQ1	rs79630929	chr11:2,466,221-2,870,340	AX-30010717
KCNQ1	rs72847676	chr11:2,466,221-2,870,340	AX-30010725
KCNQ1	rs179441	chr11:2,466,221-2,870,340	AX-39037677
KCNQ1	rs75436503	chr11:2,466,221-2,870,340	AX-30010741
KCNQ1	rs112106242	chr11:2,466,221-2,870,340	AX-156270461
KCNQ1	rs112106242	chr11:2,466,221-2,870,340	AX-156289278
KCNQ1	rs111655717	chr11:2,466,221-2,870,340	AX-30010749
KCNQ1	rs56314930	chr11:2,466,221-2,870,340	AX-30010755
KCNQ1	rs1997619	chr11:2,466,221-2,870,340	AX-96776414
KCNQ1	rs1997619	chr11:2,466,221-2,870,340	AX-96782933
KCNQ1	rs9299929	chr11:2,466,221-2,870,340	AX-39037705

KCNQ1	rs72847677	chr11:2,466,221-2,870,340	AX-30010823
KCNQ1	rs75670474	chr11:2,466,221-2,870,340	AX-156270462
KCNQ1	rs75670474	chr11:2,466,221-2,870,340	AX-156289279
KCNQ1	rs3944148	chr11:2,466,221-2,870,340	AX-112994387
KCNQ1	rs3944148	chr11:2,466,221-2,870,340	AX-113403082
KCNQ1	rs16928429	chr11:2,466,221-2,870,340	AX-30010865
KCNQ1	rs111229068	chr11:2,466,221-2,870,340	AX-30010869
KCNQ1	rs2157762	chr11:2,466,221-2,870,340	AX-96560653
KCNQ1	rs2157762	chr11:2,466,221-2,870,340	AX-96644222
KCNQ1	---	chr11:2,466,221-2,870,340	AX-30010897
KCNQ1	rs7924846	chr11:2,466,221-2,870,340	AX-39037757
KCNQ1	rs6578271	chr11:2,466,221-2,870,340	AX-96562685
KCNQ1	rs6578271	chr11:2,466,221-2,870,340	AX-96646254
KCNQ1	---	chr11:2,466,221-2,870,340	AX-96785739
KCNQ1	rs28808586	chr11:2,466,221-2,870,340	AX-96957261
KCNQ1	rs141644896	chr11:2,466,221-2,870,340	AX-144919209
KCNQ1	rs12575355	chr11:2,466,221-2,870,340	AX-96957158
KCNQ1	rs7129955	chr11:2,466,221-2,870,340	AX-112994389
KCNQ1	rs7130209	chr11:2,466,221-2,870,340	AX-16568639
KCNQ1	rs56271700	chr11:2,466,221-2,870,340	AX-144888913
KCNQ1	rs2188197	chr11:2,466,221-2,870,340	AX-115170317
KCNQ1	rs2188197	chr11:2,466,221-2,870,340	AX-96653332
KCNQ1	rs2188198	chr11:2,466,221-2,870,340	AX-84663247
KCNQ1	rs2188198	chr11:2,466,221-2,870,340	AX-97480243
KCNQ1	rs2283156	chr11:2,466,221-2,870,340	AX-156270463
KCNQ1	rs2283156	chr11:2,466,221-2,870,340	AX-156289280
KCNQ1	rs2283158	chr11:2,466,221-2,870,340	AX-39037879
KCNQ1	rs2283161	chr11:2,466,221-2,870,340	AX-12524108
KCNQ1	rs12797687	chr11:2,466,221-2,870,340	AX-113899828
KCNQ1	rs12797687	chr11:2,466,221-2,870,340	AX-113973693
KCNQ1	rs12292841	chr11:2,466,221-2,870,340	AX-11199366
KCNQ1	rs9704526	chr11:2,466,221-2,870,340	AX-96562442
KCNQ1	rs9704526	chr11:2,466,221-2,870,340	AX-96646011
KCNQ1	rs2283162	chr11:2,466,221-2,870,340	AX-16568763
KCNQ1	rs34960665	chr11:2,466,221-2,870,340	AX-97304162
KCNQ1	rs34226314	chr11:2,466,221-2,870,340	AX-12551737
KCNQ1	rs12291654	chr11:2,466,221-2,870,340	AX-113959669
KCNQ1	rs12283510	chr11:2,466,221-2,870,340	AX-39037931
KCNQ1	rs11023261	chr11:2,466,221-2,870,340	AX-39037953
KCNQ1	rs4539307	chr11:2,466,221-2,870,340	AX-11509588
KCNQ1	rs9299930	chr11:2,466,221-2,870,340	AX-39037985
KCNQ1	rs11023267	chr11:2,466,221-2,870,340	AX-11138571
KCNQ1	---	chr11:2,466,221-2,870,340	AX-96847159
KCNQ1	---	chr11:2,466,221-2,870,340	AX-97258530
KCNQ1	rs374755711	chr11:2,466,221-2,870,340	AX-144929990
KCNQ1	rs200154711	chr11:2,466,221-2,870,340	AX-96893292
KCNQ1	rs201229057	chr11:2,466,221-2,870,340	AX-144929900
KCNQ1	rs202125097	chr11:2,466,221-2,870,340	AX-144930160
KCNQ1	rs10430833	chr11:2,466,221-2,870,340	AX-11106480
KCNQ1	rs11828404	chr11:2,466,221-2,870,340	AX-96839875
KCNQ1	rs11828404	chr11:2,466,221-2,870,340	AX-97318161
KCNQ1	rs11826219	chr11:2,466,221-2,870,340	AX-30011419
KCNQ1	rs2283163	chr11:2,466,221-2,870,340	AX-30011441
KCNQ1	rs73404640	chr11:2,466,221-2,870,340	AX-156270464
KCNQ1	rs73404640	chr11:2,466,221-2,870,340	AX-156289281

KCNQ1	rs4930122	chr11:2,466,221-2,870,340	AX-96868766
KCNQ1	rs4930122	chr11:2,466,221-2,870,340	AX-96896740
KCNQ1	rs4930123	chr11:2,466,221-2,870,340	AX-30011477
KCNQ1	rs75501030	chr11:2,466,221-2,870,340	AX-30011481
KCNQ1	rs73404645	chr11:2,466,221-2,870,340	AX-156270465
KCNQ1	rs73404645	chr11:2,466,221-2,870,340	AX-156289282
KCNQ1	rs73404647	chr11:2,466,221-2,870,340	AX-16568934
KCNQ1	rs74393603	chr11:2,466,221-2,870,340	AX-37510579
KCNQ1	rs11023290	chr11:2,466,221-2,870,340	AX-96743347
KCNQ1	rs11023290	chr11:2,466,221-2,870,340	AX-97343555
KCNQ1	rs2283164	chr11:2,466,221-2,870,340	AX-39038153
KCNQ1	rs2283165	chr11:2,466,221-2,870,340	AX-30011561
KCNQ1	rs2283166	chr11:2,466,221-2,870,340	AX-96562530
KCNQ1	rs2283166	chr11:2,466,221-2,870,340	AX-96646099
KCNQ1	rs2283167	chr11:2,466,221-2,870,340	AX-30011597
KCNQ1	rs2283168	chr11:2,466,221-2,870,340	AX-30011613
KCNQ1	rs2283169	chr11:2,466,221-2,870,340	AX-95851477
KCNQ1	rs7936703	chr11:2,466,221-2,870,340	AX-96565356
KCNQ1	rs7936703	chr11:2,466,221-2,870,340	AX-96648925
KCNQ1	rs73404652	chr11:2,466,221-2,870,340	AX-30011651
KCNQ1	rs73404655	chr11:2,466,221-2,870,340	AX-96907266
KCNQ1	rs73404655	chr11:2,466,221-2,870,340	AX-96926834
KCNQ1	rs28451516	chr11:2,466,221-2,870,340	AX-30011675
KCNQ1	rs142215501	chr11:2,466,221-2,870,340	AX-96565492
KCNQ1	rs146770373	chr11:2,466,221-2,870,340	AX-148762290
KCNQ1	rs146770373	chr11:2,466,221-2,870,340	AX-156283668
KCNQ1	rs7941421	chr11:2,466,221-2,870,340	AX-88746076
KCNQ1	rs7941421	chr11:2,466,221-2,870,340	AX-97407724
KCNQ1	rs7944551	chr11:2,466,221-2,870,340	AX-90038356
KCNQ1	rs7944551	chr11:2,466,221-2,870,340	AX-96875030
KCNQ1	rs7944557	chr11:2,466,221-2,870,340	AX-96560358
KCNQ1	rs7944557	chr11:2,466,221-2,870,340	AX-96643927
KCNQ1	rs2283170	chr11:2,466,221-2,870,340	AX-96562615
KCNQ1	rs12802897	chr11:2,466,221-2,870,340	AX-30011807
KCNQ1	rs12803012	chr11:2,466,221-2,870,340	AX-96984771
KCNQ1	rs12803012	chr11:2,466,221-2,870,340	AX-96986903
KCNQ1	rs4929995	chr11:2,466,221-2,870,340	AX-30011823
KCNQ1	rs74048684	chr11:2,466,221-2,870,340	AX-96985801
KCNQ1	rs74048684	chr11:2,466,221-2,870,340	AX-97305777
KCNQ1	rs12363216	chr11:2,466,221-2,870,340	AX-30011833
KCNQ1	rs7101739	chr11:2,466,221-2,870,340	AX-96564436
KCNQ1	rs7101739	chr11:2,466,221-2,870,340	AX-96648005
KCNQ1	rs12221520	chr11:2,466,221-2,870,340	AX-30011879
KCNQ1	rs11600454	chr11:2,466,221-2,870,340	AX-30011885
KCNQ1	rs12279846	chr11:2,466,221-2,870,340	AX-30011895
KCNQ1	rs7942492	chr11:2,466,221-2,870,340	AX-96979802
KCNQ1	rs7942492	chr11:2,466,221-2,870,340	AX-97338084
KCNQ1	rs80111484	chr11:2,466,221-2,870,340	AX-96987980
KCNQ1	rs80111484	chr11:2,466,221-2,870,340	AX-97386871
KCNQ1	rs12280123	chr11:2,466,221-2,870,340	AX-30011925
KCNQ1	rs11820485	chr11:2,466,221-2,870,340	AX-96889784
KCNQ1	rs11820485	chr11:2,466,221-2,870,340	AX-97454947
KCNQ1	rs11820026	chr11:2,466,221-2,870,340	AX-96817846
KCNQ1	rs11820026	chr11:2,466,221-2,870,340	AX-97480886
KCNQ1	rs75606621	chr11:2,466,221-2,870,340	AX-30011947

KCNQ1	rs11822186	chr11:2,466,221-2,870,340	AX-30011949
KCNQ1	rs6578273	chr11:2,466,221-2,870,340	AX-39038411
KCNQ1	rs7118348	chr11:2,466,221-2,870,340	AX-96945867
KCNQ1	rs7118348	chr11:2,466,221-2,870,340	AX-97422813
KCNQ1	rs35951054	chr11:2,466,221-2,870,340	AX-30012027
KCNQ1	rs73404673	chr11:2,466,221-2,870,340	AX-30012061
KCNQ1	rs73404674	chr11:2,466,221-2,870,340	AX-156279819
KCNQ1	rs179484	chr11:2,466,221-2,870,340	AX-96562018
KCNQ1	rs179484	chr11:2,466,221-2,870,340	AX-96645587
KCNQ1	rs113962056	chr11:2,466,221-2,870,340	AX-96951438
KCNQ1	rs113962056	chr11:2,466,221-2,870,340	AX-97415482
KCNQ1	rs179486	chr11:2,466,221-2,870,340	AX-30012089
KCNQ1	rs7119191	chr11:2,466,221-2,870,340	AX-30012095
KCNQ1	rs76003132	chr11:2,466,221-2,870,340	AX-30012113
KCNQ1	rs6578274	chr11:2,466,221-2,870,340	AX-96564844
KCNQ1	rs114339307	chr11:2,466,221-2,870,340	AX-97339384
KCNQ1	rs114339307	chr11:2,466,221-2,870,340	AX-97453275
KCNQ1	rs148166405	chr11:2,466,221-2,870,340	AX-151399065
KCNQ1	rs148166405	chr11:2,466,221-2,870,340	AX-96965720
KCNQ1	rs145699003	chr11:2,466,221-2,870,340	AX-96961598
KCNQ1	rs145699003	chr11:2,466,221-2,870,340	AX-97339392
KCNQ1	rs188052441	chr11:2,466,221-2,870,340	AX-96754944
KCNQ1	rs188052441	chr11:2,466,221-2,870,340	AX-97443430
KCNQ1	rs192149751	chr11:2,466,221-2,870,340	AX-96864017
KCNQ1	rs192149751	chr11:2,466,221-2,870,340	AX-97307136
KCNQ1	rs72482705	chr11:2,466,221-2,870,340	AX-96560530
KCNQ1	rs72482705	chr11:2,466,221-2,870,340	AX-96644099
KCNQ1	rs73404676	chr11:2,466,221-2,870,340	AX-30012149
KCNQ1	rs73404678	chr11:2,466,221-2,870,340	AX-96740091
KCNQ1	rs73404678	chr11:2,466,221-2,870,340	AX-97418243
KCNQ1	rs28730752	chr11:2,466,221-2,870,340	AX-30012157
KCNQ1	---	chr11:2,466,221-2,870,340	AX-90034552
KCNQ1	---	chr11:2,466,221-2,870,340	AX-90036143
KCNQ1	---	chr11:2,466,221-2,870,340	AX-90027416
KCNQ1	rs397508112	chr11:2,466,221-2,870,340	AX-90051412
KCNQ1	---	chr11:2,466,221-2,870,340	AX-90030622
KCNQ1	---	chr11:2,466,221-2,870,340	AX-90050963
KCNQ1	---	chr11:2,466,221-2,870,340	AX-90061436
KCNQ1	---	chr11:2,466,221-2,870,340	AX-90060483
KCNQ1	---	chr11:2,466,221-2,870,340	AX-90065739
KCNQ1	---	chr11:2,466,221-2,870,340	AX-90056800
KCNQ1	---	chr11:2,466,221-2,870,340	AX-90076686
KCNQ1	---	chr11:2,466,221-2,870,340	AX-90059820
KCNQ1	---	chr11:2,466,221-2,870,340	AX-90060527
KCNQ1	---	chr11:2,466,221-2,870,340	AX-90034166
KCNQ1	---	chr11:2,466,221-2,870,340	AX-90061070
KCNQ1	---	chr11:2,466,221-2,870,340	AX-86618250
KCNQ1	---	chr11:2,466,221-2,870,340	AX-90055801
KCNQ1	rs199473394	chr11:2,466,221-2,870,340	AX-86732896
KCNQ1	---	chr11:2,466,221-2,870,340	AX-86569551
KCNQ1	---	chr11:2,466,221-2,870,340	AX-90028973
KCNQ1	---	chr11:2,466,221-2,870,340	AX-90029912
KCNQ1	---	chr11:2,466,221-2,870,340	AX-90027758
KCNQ1	---	chr11:2,466,221-2,870,340	AX-90035023
KCNQ1	rs397508116	chr11:2,466,221-2,870,340	AX-90050245

KCNQ1	---	chr11:2,466,221-2,870,340	AX-90031719
KCNQ1	---	chr11:2,466,221-2,870,340	AX-90031807
KCNQ1	---	chr11:2,466,221-2,870,340	AX-90037131
KCNQ1	---	chr11:2,466,221-2,870,340	AX-86684434
KCNQ1	---	chr11:2,466,221-2,870,340	AX-90026917
KCNQ1	---	chr11:2,466,221-2,870,340	AX-90076459
KCNQ1	---	chr11:2,466,221-2,870,340	AX-90061696
KCNQ1	---	chr11:2,466,221-2,870,340	AX-90027762
KCNQ1	---	chr11:2,466,221-2,870,340	AX-90030449
KCNQ1	---	chr11:2,466,221-2,870,340	AX-90061240
KCNQ1	rs397508119	chr11:2,466,221-2,870,340	AX-90034815
KCNQ1	---	chr11:2,466,221-2,870,340	AX-90030969
KCNQ1	---	chr11:2,466,221-2,870,340	AX-90061769
KCNQ1	---	chr11:2,466,221-2,870,340	AX-90056088
KCNQ1	---	chr11:2,466,221-2,870,340	AX-90030589
KCNQ1	rs112946114	chr11:2,466,221-2,870,340	AX-30012173
KCNQ1	rs73404679	chr11:2,466,221-2,870,340	AX-30012179
KCNQ1	rs7946012	chr11:2,466,221-2,870,340	AX-39038493
KCNQ1	---	chr11:2,466,221-2,870,340	AX-90064000
KCNQ1	---	chr11:2,466,221-2,870,340	AX-90066080
KCNQ1	---	chr11:2,466,221-2,870,340	AX-90057795
KCNQ1	---	chr11:2,466,221-2,870,340	AX-90063397
KCNQ1	rs151344631	chr11:2,466,221-2,870,340	AX-86694782
KCNQ1	---	chr11:2,466,221-2,870,340	AX-90079106
KCNQ1	---	chr11:2,466,221-2,870,340	AX-90055498
KCNQ1	---	chr11:2,466,221-2,870,340	AX-90061893
KCNQ1	rs17215479	chr11:2,466,221-2,870,340	AX-86572909
KCNQ1	---	chr11:2,466,221-2,870,340	AX-90027959
KCNQ1	---	chr11:2,466,221-2,870,340	AX-90034486
KCNQ1	---	chr11:2,466,221-2,870,340	AX-90029615
KCNQ1	rs4930126	chr11:2,466,221-2,870,340	AX-144899892
KCNQ1	rs4930126	chr11:2,466,221-2,870,340	AX-144921502
KCNQ1	---	chr11:2,466,221-2,870,340	AX-90062784
KCNQ1	---	chr11:2,466,221-2,870,340	AX-90031845
KCNQ1	---	chr11:2,466,221-2,870,340	AX-90059310
KCNQ1	---	chr11:2,466,221-2,870,340	AX-90033121
KCNQ1	---	chr11:2,466,221-2,870,340	AX-90036123
KCNQ1	---	chr11:2,466,221-2,870,340	AX-90028262
KCNQ1	---	chr11:2,466,221-2,870,340	AX-90029242
KCNQ1	---	chr11:2,466,221-2,870,340	AX-90028089
KCNQ1	---	chr11:2,466,221-2,870,340	AX-90065237
KCNQ1	---	chr11:2,466,221-2,870,340	AX-90063417
KCNQ1	rs12786951	chr11:2,466,221-2,870,340	AX-96812811
KCNQ1	rs12786951	chr11:2,466,221-2,870,340	AX-96814890
KCNQ1	rs12574935	chr11:2,466,221-2,870,340	AX-30012257
KCNQ1	rs41282926	chr11:2,466,221-2,870,340	AX-30012273
KCNQ1	rs4930127	chr11:2,466,221-2,870,340	AX-96562310
KCNQ1	rs4930127	chr11:2,466,221-2,870,340	AX-96645879
KCNQ1	---	chr11:2,466,221-2,870,340	AX-90056218
KCNQ1	---	chr11:2,466,221-2,870,340	AX-90064678
KCNQ1	---	chr11:2,466,221-2,870,340	AX-90061212
KCNQ1	---	chr11:2,466,221-2,870,340	AX-90060517
KCNQ1	---	chr11:2,466,221-2,870,340	AX-90028222
KCNQ1	---	chr11:2,466,221-2,870,340	AX-90045693
KCNQ1	---	chr11:2,466,221-2,870,340	AX-90032453

KCNQ1	rs120074193	chr11:2,466,221-2,870,340	AX-90040402
KCNQ1	rs120074194	chr11:2,466,221-2,870,340	AX-90069289
KCNQ1	---	chr11:2,466,221-2,870,340	AX-90034229
KCNQ1	rs120074180	chr11:2,466,221-2,870,340	AX-90069280
KCNQ1	---	chr11:2,466,221-2,870,340	AX-90058717
KCNQ1	rs199472728	chr11:2,466,221-2,870,340	AX-86553315
KCNQ1	---	chr11:2,466,221-2,870,340	AX-90078041
KCNQ1	---	chr11:2,466,221-2,870,340	AX-90060190
KCNQ1	---	chr11:2,466,221-2,870,340	AX-90071870
KCNQ1	---	chr11:2,466,221-2,870,340	AX-90063284
KCNQ1	---	chr11:2,466,221-2,870,340	AX-90034345
KCNQ1	---	chr11:2,466,221-2,870,340	AX-90065983
KCNQ1	---	chr11:2,466,221-2,870,340	AX-90032613
KCNQ1	---	chr11:2,466,221-2,870,340	AX-90027347
KCNQ1	---	chr11:2,466,221-2,870,340	AX-90032458
KCNQ1	---	chr11:2,466,221-2,870,340	AX-90058297
KCNQ1	---	chr11:2,466,221-2,870,340	AX-90072722
KCNQ1	---	chr11:2,466,221-2,870,340	AX-90059828
KCNQ1	rs199473464	chr11:2,466,221-2,870,340	AX-90059342
KCNQ1	rs199472736	chr11:2,466,221-2,870,340	AX-90066122
KCNQ1	rs199472737	chr11:2,466,221-2,870,340	AX-86678907
KCNQ1	---	chr11:2,466,221-2,870,340	AX-90032143
KCNQ1	---	chr11:2,466,221-2,870,340	AX-90027829
KCNQ1	---	chr11:2,466,221-2,870,340	AX-90036038
KCNQ1	---	chr11:2,466,221-2,870,340	AX-90036226
KCNQ1	---	chr11:2,466,221-2,870,340	AX-90040397
KCNQ1	---	chr11:2,466,221-2,870,340	AX-90028921
KCNQ1	---	chr11:2,466,221-2,870,340	AX-90066174
KCNQ1	---	chr11:2,466,221-2,870,340	AX-90064227
KCNQ1	---	chr11:2,466,221-2,870,340	AX-90076054
KCNQ1	rs120074195	chr11:2,466,221-2,870,340	AX-90069290
KCNQ1	rs397508131	chr11:2,466,221-2,870,340	AX-90062035
KCNQ1	rs397508130	chr11:2,466,221-2,870,340	AX-90065243
KCNQ1	rs56209304	chr11:2,466,221-2,870,340	AX-30012303
KCNQ1	rs2283171	chr11:2,466,221-2,870,340	AX-39038573
KCNQ1	rs1076147	chr11:2,466,221-2,870,340	AX-30012341
KCNQ1	rs73404683	chr11:2,466,221-2,870,340	AX-30012343
KCNQ1	rs10832305	chr11:2,466,221-2,870,340	AX-97272169
KCNQ1	rs10832305	chr11:2,466,221-2,870,340	AX-97385256
KCNQ1	rs11824323	chr11:2,466,221-2,870,340	AX-95861383
KCNQ1	rs11824323	chr11:2,466,221-2,870,340	AX-96817434
KCNQ1	rs12282937	chr11:2,466,221-2,870,340	AX-39038623
KCNQ1	rs4930128	chr11:2,466,221-2,870,340	AX-39038625
KCNQ1	rs61871514	chr11:2,466,221-2,870,340	AX-30012447
KCNQ1	rs11023355	chr11:2,466,221-2,870,340	AX-30012473
KCNQ1	rs75392110	chr11:2,466,221-2,870,340	AX-30012487
KCNQ1	rs2283172	chr11:2,466,221-2,870,340	AX-75174842
KCNQ1	rs2283172	chr11:2,466,221-2,870,340	AX-96564696
KCNQ1	rs73404690	chr11:2,466,221-2,870,340	AX-30012561
KCNQ1	rs7932175	chr11:2,466,221-2,870,340	AX-96563007
KCNQ1	rs7932175	chr11:2,466,221-2,870,340	AX-96646576
KCNQ1	rs59171663	chr11:2,466,221-2,870,340	AX-96903234
KCNQ1	rs56320672	chr11:2,466,221-2,870,340	AX-156279820
KCNQ1	rs61871515	chr11:2,466,221-2,870,340	AX-30012609
KCNQ1	rs61426093	chr11:2,466,221-2,870,340	AX-96831751

KCNQ1	rs61426093	chr11:2,466,221-2,870,340	AX-97300114
KCNQ1	rs61871516	chr11:2,466,221-2,870,340	AX-96796711
KCNQ1	rs61871516	chr11:2,466,221-2,870,340	AX-97327899
KCNQ1	rs2283173	chr11:2,466,221-2,870,340	AX-96560450
KCNQ1	rs2283174	chr11:2,466,221-2,870,340	AX-96561819
KCNQ1	rs61871517	chr11:2,466,221-2,870,340	AX-30012669
KCNQ1	rs2012323	chr11:2,466,221-2,870,340	AX-39038765
KCNQ1	rs397508133	chr11:2,466,221-2,870,340	AX-90030467
KCNQ1	rs387906290	chr11:2,466,221-2,870,340	AX-90034549
KCNQ1	---	chr11:2,466,221-2,870,340	AX-90031571
KCNQ1	---	chr11:2,466,221-2,870,340	AX-90063091
KCNQ1	---	chr11:2,466,221-2,870,340	AX-90057018
KCNQ1	---	chr11:2,466,221-2,870,340	AX-90060041
KCNQ1	---	chr11:2,466,221-2,870,340	AX-90058093
KCNQ1	rs120074182	chr11:2,466,221-2,870,340	AX-90069281
KCNQ1	---	chr11:2,466,221-2,870,340	AX-90065091
KCNQ1	---	chr11:2,466,221-2,870,340	AX-90055805
KCNQ1	---	chr11:2,466,221-2,870,340	AX-90064179
KCNQ1	---	chr11:2,466,221-2,870,340	AX-90032110
KCNQ1	---	chr11:2,466,221-2,870,340	AX-90034064
KCNQ1	---	chr11:2,466,221-2,870,340	AX-90032865
KCNQ1	---	chr11:2,466,221-2,870,340	AX-90059355
KCNQ1	---	chr11:2,466,221-2,870,340	AX-90033467
KCNQ1	---	chr11:2,466,221-2,870,340	AX-90059957
KCNQ1	---	chr11:2,466,221-2,870,340	AX-90056242
KCNQ1	---	chr11:2,466,221-2,870,340	AX-90033313
KCNQ1	---	chr11:2,466,221-2,870,340	AX-90058765
KCNQ1	---	chr11:2,466,221-2,870,340	AX-90031589
KCNQ1	---	chr11:2,466,221-2,870,340	AX-90056591
KCNQ1	---	chr11:2,466,221-2,870,340	AX-90060171
KCNQ1	---	chr11:2,466,221-2,870,340	AX-90027384
KCNQ1	---	chr11:2,466,221-2,870,340	AX-90043786
KCNQ1	---	chr11:2,466,221-2,870,340	AX-90073197
KCNQ1	---	chr11:2,466,221-2,870,340	AX-90058105
KCNQ1	---	chr11:2,466,221-2,870,340	AX-90056960
KCNQ1	rs397508069	chr11:2,466,221-2,870,340	AX-90043602
KCNQ1	---	chr11:2,466,221-2,870,340	AX-90055806
KCNQ1	---	chr11:2,466,221-2,870,340	AX-90063304
KCNQ1	---	chr11:2,466,221-2,870,340	AX-90033477
KCNQ1	---	chr11:2,466,221-2,870,340	AX-90029343
KCNQ1	---	chr11:2,466,221-2,870,340	AX-90027730
KCNQ1	---	chr11:2,466,221-2,870,340	AX-90064380
KCNQ1	---	chr11:2,466,221-2,870,340	AX-90028289
KCNQ1	---	chr11:2,466,221-2,870,340	AX-90027684
KCNQ1	---	chr11:2,466,221-2,870,340	AX-90056546
KCNQ1	---	chr11:2,466,221-2,870,340	AX-90060626
KCNQ1	---	chr11:2,466,221-2,870,340	AX-90060738
KCNQ1	rs199876246	chr11:2,466,221-2,870,340	AX-151350651
KCNQ1	rs56750705	chr11:2,466,221-2,870,340	AX-97476613
KCNQ1	---	chr11:2,466,221-2,870,340	AX-39038791
KCNQ1	---	chr11:2,466,221-2,870,340	AX-90056001
KCNQ1	rs120074183	chr11:2,466,221-2,870,340	AX-90069282
KCNQ1	---	chr11:2,466,221-2,870,340	AX-90030281
KCNQ1	---	chr11:2,466,221-2,870,340	AX-90028919
KCNQ1	---	chr11:2,466,221-2,870,340	AX-90064580

KCNQ1	---	chr11:2,466,221-2,870,340	AX-90030563
KCNQ1	---	chr11:2,466,221-2,870,340	AX-90032432
KCNQ1	---	chr11:2,466,221-2,870,340	AX-90029608
KCNQ1	---	chr11:2,466,221-2,870,340	AX-90030915
KCNQ1	---	chr11:2,466,221-2,870,340	AX-90036263
KCNQ1	---	chr11:2,466,221-2,870,340	AX-90073150
KCNQ1	---	chr11:2,466,221-2,870,340	AX-90033459
KCNQ1	rs397508075	chr11:2,466,221-2,870,340	AX-90034324
KCNQ1	---	chr11:2,466,221-2,870,340	AX-90031628
KCNQ1	---	chr11:2,466,221-2,870,340	AX-90062426
KCNQ1	rs12720458	chr11:2,466,221-2,870,340	AX-90038259
KCNQ1	---	chr11:2,466,221-2,870,340	AX-90059564
KCNQ1	---	chr11:2,466,221-2,870,340	AX-90059337
KCNQ1	---	chr11:2,466,221-2,870,340	AX-90035646
KCNQ1	---	chr11:2,466,221-2,870,340	AX-90031994
KCNQ1	---	chr11:2,466,221-2,870,340	AX-90034033
KCNQ1	---	chr11:2,466,221-2,870,340	AX-90059210
KCNQ1	---	chr11:2,466,221-2,870,340	AX-90060433
KCNQ1	---	chr11:2,466,221-2,870,340	AX-90063249
KCNQ1	---	chr11:2,466,221-2,870,340	AX-90031328
KCNQ1	---	chr11:2,466,221-2,870,340	AX-90030080
KCNQ1	---	chr11:2,466,221-2,870,340	AX-90073425
KCNQ1	rs397508078	chr11:2,466,221-2,870,340	AX-90027967
KCNQ1	---	chr11:2,466,221-2,870,340	AX-90040340
KCNQ1	rs56413900	chr11:2,466,221-2,870,340	AX-30012837
KCNQ1	rs56859078	chr11:2,466,221-2,870,340	AX-96886752
KCNQ1	rs56859078	chr11:2,466,221-2,870,340	AX-97357452
KCNQ1	rs756851	chr11:2,466,221-2,870,340	AX-96645469
KCNQ1	rs12808620	chr11:2,466,221-2,870,340	AX-96560769
KCNQ1	---	chr11:2,466,221-2,870,340	AX-90056717
KCNQ1	---	chr11:2,466,221-2,870,340	AX-90063846
KCNQ1	---	chr11:2,466,221-2,870,340	AX-90074656
KCNQ1	---	chr11:2,466,221-2,870,340	AX-90031139
KCNQ1	---	chr11:2,466,221-2,870,340	AX-90075418
KCNQ1	---	chr11:2,466,221-2,870,340	AX-90065684
KCNQ1	---	chr11:2,466,221-2,870,340	AX-90062263
KCNQ1	---	chr11:2,466,221-2,870,340	AX-90029227
KCNQ1	---	chr11:2,466,221-2,870,340	AX-90065657
KCNQ1	---	chr11:2,466,221-2,870,340	AX-90065329
KCNQ1	---	chr11:2,466,221-2,870,340	AX-90066245
KCNQ1	---	chr11:2,466,221-2,870,340	AX-90049319
KCNQ1	rs199472776	chr11:2,466,221-2,870,340	AX-83450931
KCNQ1	---	chr11:2,466,221-2,870,340	AX-90062050
KCNQ1	---	chr11:2,466,221-2,870,340	AX-90072360
KCNQ1	rs73404698	chr11:2,466,221-2,870,340	AX-30012977
KCNQ1	rs112193996	chr11:2,466,221-2,870,340	AX-30012997
KCNQ1	rs11828659	chr11:2,466,221-2,870,340	AX-39038901
KCNQ1	rs7946404	chr11:2,466,221-2,870,340	AX-97434433
KCNQ1	rs59161111	chr11:2,466,221-2,870,340	AX-30013003
KCNQ1	---	chr11:2,466,221-2,870,340	AX-90057278
KCNQ1	---	chr11:2,466,221-2,870,340	AX-90042660
KCNQ1	---	chr11:2,466,221-2,870,340	AX-90035146
KCNQ1	---	chr11:2,466,221-2,870,340	AX-90074661
KCNQ1	---	chr11:2,466,221-2,870,340	AX-90064095
KCNQ1	---	chr11:2,466,221-2,870,340	AX-90072232

KCNQ1	---	chr11:2,466,221-2,870,340	AX-90055899
KCNQ1	rs140452381	chr11:2,466,221-2,870,340	AX-86687312
KCNQ1	---	chr11:2,466,221-2,870,340	AX-90064921
KCNQ1	rs199472783	chr11:2,466,221-2,870,340	AX-86555070
KCNQ1	rs10766203	chr11:2,466,221-2,870,340	AX-39038909
KCNQ1	rs12804487	chr11:2,466,221-2,870,340	AX-96560722
KCNQ1	rs12804487	chr11:2,466,221-2,870,340	AX-96644291
KCNQ1	rs36008556	chr11:2,466,221-2,870,340	AX-96752583
KCNQ1	rs35421868	chr11:2,466,221-2,870,340	AX-113407398
KCNQ1	rs35421868	chr11:2,466,221-2,870,340	AX-113963898
KCNQ1	rs112180245	chr11:2,466,221-2,870,340	AX-30013067
KCNQ1	rs73413288	chr11:2,466,221-2,870,340	AX-30013095
KCNQ1	rs1024381	chr11:2,466,221-2,870,340	AX-97422233
KCNQ1	rs2283175	chr11:2,466,221-2,870,340	AX-39038947
KCNQ1	rs2283177	chr11:2,466,221-2,870,340	AX-30013125
KCNQ1	rs75863300	chr11:2,466,221-2,870,340	AX-96797621
KCNQ1	rs75863300	chr11:2,466,221-2,870,340	AX-96830117
KCNQ1	rs2283178	chr11:2,466,221-2,870,340	AX-30013147
KCNQ1	rs74049370	chr11:2,466,221-2,870,340	AX-156279821
KCNQ1	rs74049370	chr11:2,466,221-2,870,340	AX-156295409
KCNQ1	rs7933215	chr11:2,466,221-2,870,340	AX-156279822
KCNQ1	rs7933215	chr11:2,466,221-2,870,340	AX-156295410
KCNQ1	rs2283179	chr11:2,466,221-2,870,340	AX-39038955
KCNQ1	rs2299618	chr11:2,466,221-2,870,340	AX-30013243
KCNQ1	rs2157760	chr11:2,466,221-2,870,340	AX-39038993
KCNQ1	rs77625359	chr11:2,466,221-2,870,340	AX-30013355
KCNQ1	rs2188194	chr11:2,466,221-2,870,340	AX-30013367
KCNQ1	---	chr11:2,466,221-2,870,340	AX-16569961
KCNQ1	rs3032764	chr11:2,466,221-2,870,340	AX-97407567
KCNQ1	rs1116714	chr11:2,466,221-2,870,340	AX-11148435
KCNQ1	rs7936654	chr11:2,466,221-2,870,340	AX-16569979
KCNQ1	rs1116715	chr11:2,466,221-2,870,340	AX-96819365
KCNQ1	rs1116715	chr11:2,466,221-2,870,340	AX-97261312
KCNQ1	rs4930129	chr11:2,466,221-2,870,340	AX-112925554
KCNQ1	rs4930129	chr11:2,466,221-2,870,340	AX-112999622
KCNQ1	rs4930130	chr11:2,466,221-2,870,340	AX-96945448
KCNQ1	rs4930130	chr11:2,466,221-2,870,340	AX-97442442
KCNQ1	rs4930131	chr11:2,466,221-2,870,340	AX-16570045
KCNQ1	rs12277672	chr11:2,466,221-2,870,340	AX-16570052
KCNQ1	rs12279024	chr11:2,466,221-2,870,340	AX-11198877
KCNQ1	rs11822631	chr11:2,466,221-2,870,340	AX-12420364
KCNQ1	rs2188195	chr11:2,466,221-2,870,340	AX-39039127
KCNQ1	rs10832352	chr11:2,466,221-2,870,340	AX-113400621
KCNQ1	rs10832352	chr11:2,466,221-2,870,340	AX-113474479
KCNQ1	rs11023421	chr11:2,466,221-2,870,340	AX-16570092
KCNQ1	rs7934636	chr11:2,466,221-2,870,340	AX-113891033
KCNQ1	rs11023425	chr11:2,466,221-2,870,340	AX-30013617
KCNQ1	rs11023427	chr11:2,466,221-2,870,340	AX-16570110
KCNQ1	rs7944932	chr11:2,466,221-2,870,340	AX-30013629
KCNQ1	rs7947990	chr11:2,466,221-2,870,340	AX-96879166
KCNQ1	rs7947990	chr11:2,466,221-2,870,340	AX-97290314
KCNQ1	rs4929999	chr11:2,466,221-2,870,340	AX-16570132
KCNQ1	rs4930000	chr11:2,466,221-2,870,340	AX-12586273
KCNQ1	rs76698782	chr11:2,466,221-2,870,340	AX-16570139
KCNQ1	rs7121454	chr11:2,466,221-2,870,340	AX-96833040

KCNQ1	rs7121454	chr11:2,466,221-2,870,340	AX-96864817
KCNQ1	rs7104842	chr11:2,466,221-2,870,340	AX-30013739
KCNQ1	rs114404527	chr11:2,466,221-2,870,340	AX-96847088
KCNQ1	rs114404527	chr11:2,466,221-2,870,340	AX-97266296
KCNQ1	rs12797641	chr11:2,466,221-2,870,340	AX-148165073
KCNQ1	rs12797641	chr11:2,466,221-2,870,340	AX-156270467
KCNQ1	rs12799862	chr11:2,466,221-2,870,340	AX-105142913
KCNQ1	rs11023433	chr11:2,466,221-2,870,340	AX-156270468
KCNQ1	rs11023433	chr11:2,466,221-2,870,340	AX-92393588
KCNQ1	rs143548626	chr11:2,466,221-2,870,340	AX-144905831
KCNQ1	rs143548626	chr11:2,466,221-2,870,340	AX-144927827
KCNQ1	rs61871520	chr11:2,466,221-2,870,340	AX-96945314
KCNQ1	rs61871520	chr11:2,466,221-2,870,340	AX-96955854
KCNQ1	rs11529572	chr11:2,466,221-2,870,340	AX-148191045
KCNQ1	rs11529572	chr11:2,466,221-2,870,340	AX-156283274
KCNQ1	rs7947972	chr11:2,466,221-2,870,340	AX-96906174
KCNQ1	rs7947972	chr11:2,466,221-2,870,340	AX-97273599
KCNQ1	rs7947981	chr11:2,466,221-2,870,340	AX-96740403
KCNQ1	rs7947981	chr11:2,466,221-2,870,340	AX-96746960
KCNQ1	rs58827264	chr11:2,466,221-2,870,340	AX-156270469
KCNQ1	rs58827264	chr11:2,466,221-2,870,340	AX-156289284
KCNQ1	rs7130232	chr11:2,466,221-2,870,340	AX-39039293
KCNQ1	rs75431610	chr11:2,466,221-2,870,340	AX-16570259
KCNQ1	rs9667198	chr11:2,466,221-2,870,340	AX-30013853
KCNQ1	rs111331313	chr11:2,466,221-2,870,340	AX-96826894
KCNQ1	rs111331313	chr11:2,466,221-2,870,340	AX-97447318
KCNQ1	rs11023451	chr11:2,466,221-2,870,340	AX-88780469
KCNQ1	rs7108478	chr11:2,466,221-2,870,340	AX-12620934
KCNQ1	rs73415327	chr11:2,466,221-2,870,340	AX-156270470
KCNQ1	rs73415327	chr11:2,466,221-2,870,340	AX-156289285
KCNQ1	rs73415330	chr11:2,466,221-2,870,340	AX-123055617
KCNQ1	rs73415330	chr11:2,466,221-2,870,340	AX-123055618
KCNQ1	rs7104624	chr11:2,466,221-2,870,340	AX-12620810
KCNQ1	rs4930137	chr11:2,466,221-2,870,340	AX-16570329
KCNQ1	rs4930138	chr11:2,466,221-2,870,340	AX-16570342
KCNQ1	rs4930139	chr11:2,466,221-2,870,340	AX-11536730
KCNQ1	rs117893171	chr11:2,466,221-2,870,340	AX-16570371
KCNQ1	rs1965606	chr11:2,466,221-2,870,340	AX-123022913
KCNQ1	rs1965606	chr11:2,466,221-2,870,340	AX-123022914
KCNQ1	rs10766212	chr11:2,466,221-2,870,340	AX-11120065
KCNQ1	rs7943271	chr11:2,466,221-2,870,340	AX-96775494
KCNQ1	rs7943271	chr11:2,466,221-2,870,340	AX-97473695
KCNQ1	rs7942590	chr11:2,466,221-2,870,340	AX-12645351
KCNQ1	rs7943802	chr11:2,466,221-2,870,340	AX-30014125
KCNQ1	rs80102379	chr11:2,466,221-2,870,340	AX-16570452
KCNQ1	rs2106467	chr11:2,466,221-2,870,340	AX-16570510
KCNQ1	rs55724397	chr11:2,466,221-2,870,340	AX-16570533
KCNQ1	rs2106466	chr11:2,466,221-2,870,340	AX-113883372
KCNQ1	rs2106465	chr11:2,466,221-2,870,340	AX-16570555
KCNQ1	rs10766217	chr11:2,466,221-2,870,340	AX-123046838
KCNQ1	rs10766218	chr11:2,466,221-2,870,340	AX-16570583
KCNQ1	rs2106464	chr11:2,466,221-2,870,340	AX-123022921
KCNQ1	rs2106464	chr11:2,466,221-2,870,340	AX-123022922
KCNQ1	rs2106463	chr11:2,466,221-2,870,340	AX-123050150
KCNQ1	rs2106463	chr11:2,466,221-2,870,340	AX-123050151

KCNQ1	rs11023485	chr11:2,466,221-2,870,340	AX-11138588
KCNQ1	rs4930141	chr11:2,466,221-2,870,340	AX-30014369
KCNQ1	rs7944966	chr11:2,466,221-2,870,340	AX-96562464
KCNQ1	rs77402029	chr11:2,466,221-2,870,340	AX-96729340
KCNQ1	rs77402029	chr11:2,466,221-2,870,340	AX-96757637
KCNQ1	rs7945093	chr11:2,466,221-2,870,340	AX-11655310
KCNQ1	rs7929804	chr11:2,466,221-2,870,340	AX-39039711
KCNQ1	rs2412058	chr11:2,466,221-2,870,340	AX-11390411
KCNQ1	rs78583424	chr11:2,466,221-2,870,340	AX-96803993
KCNQ1	rs78583424	chr11:2,466,221-2,870,340	AX-96823482
KCNQ1	---	chr11:2,466,221-2,870,340	AX-151178419
KCNQ1	---	chr11:2,466,221-2,870,340	AX-156284962
KCNQ1	rs2412057	chr11:2,466,221-2,870,340	AX-96564665
KCNQ1	rs2412057	chr11:2,466,221-2,870,340	AX-96648234
KCNQ1	rs11023499	chr11:2,466,221-2,870,340	AX-96564922
KCNQ1	rs11023499	chr11:2,466,221-2,870,340	AX-96648491
KCNQ1	rs201092224	chr11:2,466,221-2,870,340	AX-96975337
KCNQ1	rs201092224	chr11:2,466,221-2,870,340	AX-96994766
KCNQ1	rs73415358	chr11:2,466,221-2,870,340	AX-96912126
KCNQ1	rs73415358	chr11:2,466,221-2,870,340	AX-97262854
KCNQ1	rs7128307	chr11:2,466,221-2,870,340	AX-96910336
KCNQ1	rs7128307	chr11:2,466,221-2,870,340	AX-96921310
KCNQ1	rs11529573	chr11:2,466,221-2,870,340	AX-96560375
KCNQ1	rs11529573	chr11:2,466,221-2,870,340	AX-96643944
KCNQ1	rs7937711	chr11:2,466,221-2,870,340	AX-39039821
KCNQ1	rs2157899	chr11:2,466,221-2,870,340	AX-96879482
KCNQ1	rs2157899	chr11:2,466,221-2,870,340	AX-97290663
KCNQ1	rs11493163	chr11:2,466,221-2,870,340	AX-30014573
KCNQ1	rs141101139	chr11:2,466,221-2,870,340	AX-96842090
KCNQ1	rs141101139	chr11:2,466,221-2,870,340	AX-96852756
KCNQ1	rs7939222	chr11:2,466,221-2,870,340	AX-30014583
KCNQ1	rs10741669	chr11:2,466,221-2,870,340	AX-12394249
KCNQ1	rs73415376	chr11:2,466,221-2,870,340	AX-16570785
KCNQ1	rs10734233	chr11:2,466,221-2,870,340	AX-16570791
KCNQ1	rs10766228	chr11:2,466,221-2,870,340	AX-113891032
KCNQ1	rs72850242	chr11:2,466,221-2,870,340	AX-16570797
KCNQ1	rs73415381	chr11:2,466,221-2,870,340	AX-16570867
KCNQ1	rs7129523	chr11:2,466,221-2,870,340	AX-97364649
KCNQ1	rs7129523	chr11:2,466,221-2,870,340	AX-97478897
KCNQ1	rs7943674	chr11:2,466,221-2,870,340	AX-113416158
KCNQ1	rs7943674	chr11:2,466,221-2,870,340	AX-113490252
KCNQ1	rs7118939	chr11:2,466,221-2,870,340	AX-113896575
KCNQ1	rs7103309	chr11:2,466,221-2,870,340	AX-113021917
KCNQ1	rs7103309	chr11:2,466,221-2,870,340	AX-113913149
KCNQ1	rs144940002	chr11:2,466,221-2,870,340	AX-156298503
KCNQ1	rs144940002	chr11:2,466,221-2,870,340	AX-16570902
KCNQ1	rs12575958	chr11:2,466,221-2,870,340	AX-11215712
KCNQ1	rs2412056	chr11:2,466,221-2,870,340	AX-112933308
KCNQ1	rs2412056	chr11:2,466,221-2,870,340	AX-113490081
KCNQ1	rs57775947	chr11:2,466,221-2,870,340	AX-156284589
KCNQ1	rs10741670	chr11:2,466,221-2,870,340	AX-113007162
KCNQ1	rs10832405	chr11:2,466,221-2,870,340	AX-113891034
KCNQ1	rs10832405	chr11:2,466,221-2,870,340	AX-113964896
KCNQ1	rs12365197	chr11:2,466,221-2,870,340	AX-30014879
KCNQ1	rs11023535	chr11:2,466,221-2,870,340	AX-30014881

KCNQ1	rs11023536	chr11:2,466,221-2,870,340	AX-30014887
KCNQ1	rs10766236	chr11:2,466,221-2,870,340	AX-30014969
KCNQ1	rs11023540	chr11:2,466,221-2,870,340	AX-123022931
KCNQ1	rs11023540	chr11:2,466,221-2,870,340	AX-123022932
KCNQ1	rs10430888	chr11:2,466,221-2,870,340	AX-16571029
KCNQ1	rs4271371	chr11:2,466,221-2,870,340	AX-113414786
KCNQ1	rs4271371	chr11:2,466,221-2,870,340	AX-113971232
KCNQ1	rs10430889	chr11:2,466,221-2,870,340	AX-156295411
KCNQ1	rs10766242	chr11:2,466,221-2,870,340	AX-123049780
KCNQ1	rs35469749	chr11:2,466,221-2,870,340	AX-30015105
KCNQ1	rs141868481	chr11:2,466,221-2,870,340	AX-96797959
KCNQ1	rs10832430	chr11:2,466,221-2,870,340	AX-11125433
KCNQ1	rs201811937	chr11:2,466,221-2,870,340	AX-97486665
KCNQ1	rs9667408	chr11:2,466,221-2,870,340	AX-96914134
KCNQ1	rs9667408	chr11:2,466,221-2,870,340	AX-96920725
KCNQ1	rs144791799	chr11:2,466,221-2,870,340	AX-148764606
KCNQ1	rs144791799	chr11:2,466,221-2,870,340	AX-96737434
KCNQ1	rs7102138	chr11:2,466,221-2,870,340	AX-96854864
KCNQ1	rs7102138	chr11:2,466,221-2,870,340	AX-97290641
KCNQ1	rs11023582	chr11:2,466,221-2,870,340	AX-105149108
KCNQ1	rs11023582	chr11:2,466,221-2,870,340	AX-96783719
KCNQ1	rs116374321	chr11:2,466,221-2,870,340	AX-96934306
KCNQ1	rs116374321	chr11:2,466,221-2,870,340	AX-96966275
KCNQ1	rs115614041	chr11:2,466,221-2,870,340	AX-144888935
KCNQ1	rs115614041	chr11:2,466,221-2,870,340	AX-144909228
KCNQ1	rs7936778	chr11:2,466,221-2,870,340	AX-113480494
KCNQ1	rs7936778	chr11:2,466,221-2,870,340	AX-113595452
KCNQ1	rs11023583	chr11:2,466,221-2,870,340	AX-105105895
KCNQ1	rs11023583	chr11:2,466,221-2,870,340	AX-112995156
KCNQ1	rs1119488	chr11:2,466,221-2,870,340	AX-16571401
KCNQ1	rs756852	chr11:2,466,221-2,870,340	AX-96563613
KCNQ1	rs76483719	chr11:2,466,221-2,870,340	AX-54513750
KCNQ1	rs74501871	chr11:2,466,221-2,870,340	AX-16571481
KCNQ1	rs7124833	chr11:2,466,221-2,870,340	AX-30015603
KCNQ1	rs7108035	chr11:2,466,221-2,870,340	AX-16571490
KCNQ1	rs2412055	chr11:2,466,221-2,870,340	AX-112991952
KCNQ1	rs2412055	chr11:2,466,221-2,870,340	AX-113400622
KCNQ1	rs11824124	chr11:2,466,221-2,870,340	AX-30015739
KCNQ1	rs202108554	chr11:2,466,221-2,870,340	AX-96822933
KCNQ1	rs202108554	chr11:2,466,221-2,870,340	AX-97469739
KCNQ1	rs463337	chr11:2,466,221-2,870,340	AX-30015897
KCNQ1	rs466597	chr11:2,466,221-2,870,340	AX-11516313
KCNQ1	rs170762	chr11:2,466,221-2,870,340	AX-30015949
KCNQ1	rs34514246	chr11:2,466,221-2,870,340	AX-16571691
KCNQ1	rs12422173	chr11:2,466,221-2,870,340	AX-11204893
KCNQ1	rs231349	chr11:2,466,221-2,870,340	AX-12525681
KCNQ1	rs6578283	chr11:2,466,221-2,870,340	AX-11573324
KCNQ1	rs231348	chr11:2,466,221-2,870,340	AX-39040657
KCNQ1	rs231347	chr11:2,466,221-2,870,340	AX-39040677
KCNQ1	rs231346	chr11:2,466,221-2,870,340	AX-30016051
KCNQ1	rs231345	chr11:2,466,221-2,870,340	AX-30016055
KCNQ1	rs231344	chr11:2,466,221-2,870,340	AX-11384371
KCNQ1	rs150171639	chr11:2,466,221-2,870,340	AX-151356191
KCNQ1	rs150171639	chr11:2,466,221-2,870,340	AX-156298579
KCNQ1	rs76888915	chr11:2,466,221-2,870,340	AX-30016101

KCNQ1	rs231343	chr11:2,466,221-2,870,340	AX-39040771
KCNQ1	rs57308681	chr11:2,466,221-2,870,340	AX-30016109
KCNQ1	rs231342	chr11:2,466,221-2,870,340	AX-30016117
KCNQ1	rs231341	chr11:2,466,221-2,870,340	AX-30016127
KCNQ1	rs189137	chr11:2,466,221-2,870,340	AX-50051364
KCNQ1	rs35237966	chr11:2,466,221-2,870,340	AX-96563795
KCNQ1	rs35237966	chr11:2,466,221-2,870,340	AX-96647364
KCNQ1	rs151216	chr11:2,466,221-2,870,340	AX-96971226
KCNQ1	rs151216	chr11:2,466,221-2,870,340	AX-97408781
KCNQ1	rs79245945	chr11:2,466,221-2,870,340	AX-96827073
KCNQ1	rs151215	chr11:2,466,221-2,870,340	AX-96645723
KCNQ1	rs12573965	chr11:2,466,221-2,870,340	AX-30016231
KCNQ1	rs11820621	chr11:2,466,221-2,870,340	AX-39040835
KCNQ1	rs231364	chr11:2,466,221-2,870,340	AX-39040839
KCNQ1	rs739502	chr11:2,466,221-2,870,340	AX-30016271
KCNQ1	rs739502	chr11:2,466,221-2,870,340	AX-91989312
KCNQ1	rs28730758	chr11:2,466,221-2,870,340	AX-39040849
KCNQ1	---	chr11:2,466,221-2,870,340	AX-90061527
KCNQ1	---	chr11:2,466,221-2,870,340	AX-90062389
KCNQ1	rs17215465	chr11:2,466,221-2,870,340	AX-39040853
KCNQ1	---	chr11:2,466,221-2,870,340	AX-90072635
KCNQ1	---	chr11:2,466,221-2,870,340	AX-90046047
KCNQ1	---	chr11:2,466,221-2,870,340	AX-90045672
KCNQ1	rs397508091	chr11:2,466,221-2,870,340	AX-90056611
KCNQ1	rs397508093	chr11:2,466,221-2,870,340	AX-90060042
KCNQ1	rs760419	chr11:2,466,221-2,870,340	AX-91987526
KCNQ1	rs760419	chr11:2,466,221-2,870,340	AX-92012653
KCNQ1	rs2075868	chr11:2,466,221-2,870,340	AX-96742019
KCNQ1	rs2075868	chr11:2,466,221-2,870,340	AX-96748577
KCNQ1	rs743647	chr11:2,466,221-2,870,340	AX-30016303
KCNQ1	rs9666537	chr11:2,466,221-2,870,340	AX-96562522
KCNQ1	rs9666538	chr11:2,466,221-2,870,340	AX-96767722
KCNQ1	rs9666538	chr11:2,466,221-2,870,340	AX-96791425
KCNQ1	rs12271234	chr11:2,466,221-2,870,340	AX-30016397
KCNQ1	rs16928525	chr11:2,466,221-2,870,340	AX-39040955
KCNQ1	rs10832514	chr11:2,466,221-2,870,340	AX-39040963
KCNQ1	---	chr11:2,466,221-2,870,340	AX-39040967
KCNQ1	rs12418076	chr11:2,466,221-2,870,340	AX-39040973
KCNQ1	rs12294861	chr11:2,466,221-2,870,340	AX-39040983
KCNQ1	rs1459825	chr11:2,466,221-2,870,340	AX-39040991
KCNQ1	rs61870801	chr11:2,466,221-2,870,340	AX-30016509
KCNQ1	rs151212	chr11:2,466,221-2,870,340	AX-16572098
KCNQ1	rs4930005	chr11:2,466,221-2,870,340	AX-39041001
KCNQ1	rs231362	chr11:2,466,221-2,870,340	AX-82894743
KCNQ1	rs231361	chr11:2,466,221-2,870,340	AX-39041057
KCNQ1	rs231360	chr11:2,466,221-2,870,340	AX-96645636
KCNQ1	rs144613775	chr11:2,466,221-2,870,340	AX-96561539
KCNQ1	rs61870802	chr11:2,466,221-2,870,340	AX-96562215
KCNQ1	rs61870802	chr11:2,466,221-2,870,340	AX-96645784
KCNQ1	rs35889184	chr11:2,466,221-2,870,340	AX-30016609
KCNQ1	---	chr11:2,466,221-2,870,340	AX-96822151
KCNQ1	rs231359	chr11:2,466,221-2,870,340	AX-39041157
KCNQ1	rs7105073	chr11:2,466,221-2,870,340	AX-75178552
KCNQ1	rs7105073	chr11:2,466,221-2,870,340	AX-96905916
KCNQ1	rs7128926	chr11:2,466,221-2,870,340	AX-16572309

KCNQ1	rs9666604	chr11:2,466,221-2,870,340	AX-11699928
KCNQ1	rs16928538	chr11:2,466,221-2,870,340	AX-11290516
KCNQ1	rs11023738	chr11:2,466,221-2,870,340	AX-11138598
KCNQ1	rs72850276	chr11:2,466,221-2,870,340	AX-16572371
KCNQ1	rs111698879	chr11:2,466,221-2,870,340	AX-96563066
KCNQ1	rs111698879	chr11:2,466,221-2,870,340	AX-96646635
KCNQ1	rs7941377	chr11:2,466,221-2,870,340	AX-16572374
KCNQ1	rs231358	chr11:2,466,221-2,870,340	AX-39041377
KCNQ1	rs35931322	chr11:2,466,221-2,870,340	AX-96970021
KCNQ1	rs35931322	chr11:2,466,221-2,870,340	AX-97366730
KCNQ1	rs231357	chr11:2,466,221-2,870,340	AX-39041419
KCNQ1	rs231356	chr11:2,466,221-2,870,340	AX-39041431
KCNQ1	rs231355	chr11:2,466,221-2,870,340	AX-97318688
KCNQ1	rs231354	chr11:2,466,221-2,870,340	AX-39041465
KCNQ1	rs75643572	chr11:2,466,221-2,870,340	AX-30017263
KCNQ1	rs231353	chr11:2,466,221-2,870,340	AX-96562104
KCNQ1	rs11023793	chr11:2,466,221-2,870,340	AX-39041549
KCNQ1	rs7127825	chr11:2,466,221-2,870,340	AX-112994390
KCNQ1	rs7127825	chr11:2,466,221-2,870,340	AX-113885782
KCNQ1	---	chr11:2,466,221-2,870,340	AX-39041571
KCNQ1	rs2283189	chr11:2,466,221-2,870,340	AX-30017417
KCNQ1	rs231351	chr11:2,466,221-2,870,340	AX-39041575
KCNQ1	rs2283190	chr11:2,466,221-2,870,340	AX-39041583
KCNQ1	rs7939976	chr11:2,466,221-2,870,340	AX-39041599
KCNQ1	rs146937958	chr11:2,466,221-2,870,340	AX-151426790
KCNQ1	rs146937958	chr11:2,466,221-2,870,340	AX-156284647
KCNQ1	rs231350	chr11:2,466,221-2,870,340	AX-30017489
KCNQ1	rs11821797	chr11:2,466,221-2,870,340	AX-39041631
KCNQ1	rs12275726	chr11:2,466,221-2,870,340	AX-39041645
KCNQ1	rs16928561	chr11:2,466,221-2,870,340	AX-39041673
KCNQ1	rs7927129	chr11:2,466,221-2,870,340	AX-39041675
KCNQ1	rs457704	chr11:2,466,221-2,870,340	AX-30017565
KCNQ1	rs72850281	chr11:2,466,221-2,870,340	AX-30017569
KCNQ1	rs11023831	chr11:2,466,221-2,870,340	AX-39041705
KCNQ1	rs462402	chr11:2,466,221-2,870,340	AX-16572890
KCNQ1	rs463924	chr11:2,466,221-2,870,340	AX-16572902
KCNQ1	rs2283193	chr11:2,466,221-2,870,340	AX-30017599
KCNQ1	rs74472063	chr11:2,466,221-2,870,340	AX-156270471
KCNQ1	rs74472063	chr11:2,466,221-2,870,340	AX-156289286
KCNQ1	rs78344341	chr11:2,466,221-2,870,340	AX-30017625
KCNQ1	rs624471	chr11:2,466,221-2,870,340	AX-30017641
KCNQ1	rs10832548	chr11:2,466,221-2,870,340	AX-30017653
KCNQ1	rs10741690	chr11:2,466,221-2,870,340	AX-30017661
KCNQ1	rs11023834	chr11:2,466,221-2,870,340	AX-30017679
KCNQ1	rs201941541	chr11:2,466,221-2,870,340	AX-96905644
KCNQ1	rs201941541	chr11:2,466,221-2,870,340	AX-96929529
KCNQ1	rs35957135	chr11:2,466,221-2,870,340	AX-151177011
KCNQ1	rs35957135	chr11:2,466,221-2,870,340	AX-156289287
KCNQ1	rs12277353	chr11:2,466,221-2,870,340	AX-96940940
KCNQ1	rs12277353	chr11:2,466,221-2,870,340	AX-96960079
KCNQ1	rs364930	chr11:2,466,221-2,870,340	AX-96949054
KCNQ1	rs364930	chr11:2,466,221-2,870,340	AX-96955350
KCNQ1	rs80269905	chr11:2,466,221-2,870,340	AX-96732474
KCNQ1	rs145762501	chr11:2,466,221-2,870,340	AX-96732503
KCNQ1	rs12360708	chr11:2,466,221-2,870,340	AX-96774910

KCNQ1	rs12360708	chr11:2,466,221-2,870,340	AX-96781428
KCNQ1	rs7940500	chr11:2,466,221-2,870,340	AX-30017731
KCNQ1	rs11023840	chr11:2,466,221-2,870,340	AX-30017735
KCNQ1	rs2283194	chr11:2,466,221-2,870,340	AX-39041751
KCNQ1	rs231842	chr11:2,466,221-2,870,340	AX-97309245
KCNQ1	rs55786794	chr11:2,466,221-2,870,340	AX-156270472
KCNQ1	rs55786794	chr11:2,466,221-2,870,340	AX-156289288
KCNQ1	rs231841	chr11:2,466,221-2,870,340	AX-96645660
KCNQ1	rs231840	chr11:2,466,221-2,870,340	AX-96750002
KCNQ1	rs2283196	chr11:2,466,221-2,870,340	AX-30017861
KCNQ1	---	chr11:2,466,221-2,870,340	AX-156270473
KCNQ1	---	chr11:2,466,221-2,870,340	AX-156289289
KCNQ1	rs7109371	chr11:2,466,221-2,870,340	AX-30017871
KCNQ1	rs71473716	chr11:2,466,221-2,870,340	AX-30017875
KCNQ1	rs3216306	chr11:2,466,221-2,870,340	AX-88815609
KCNQ1	rs61870825	chr11:2,466,221-2,870,340	AX-30017891
KCNQ1	rs86392	chr11:2,466,221-2,870,340	AX-30017897
KCNQ1	rs78131	chr11:2,466,221-2,870,340	AX-96979470
KCNQ1	rs78131	chr11:2,466,221-2,870,340	AX-96990347
KCNQ1	rs7938807	chr11:2,466,221-2,870,340	AX-30017927
KCNQ1	rs7939695	chr11:2,466,221-2,870,340	AX-113885783
KCNQ1	rs7939695	chr11:2,466,221-2,870,340	AX-113959670
KCNQ1	rs11023855	chr11:2,466,221-2,870,340	AX-39041831
KCNQ1	rs2283200	chr11:2,466,221-2,870,340	AX-39041833
KCNQ1	rs7103496	chr11:2,466,221-2,870,340	AX-30017985
KCNQ1	rs231903	chr11:2,466,221-2,870,340	AX-96943019
KCNQ1	rs34617956	chr11:2,466,221-2,870,340	AX-96565456
KCNQ1	rs231901	chr11:2,466,221-2,870,340	AX-39041849
KCNQ1	rs73419350	chr11:2,466,221-2,870,340	AX-96844472
KCNQ1	rs73419350	chr11:2,466,221-2,870,340	AX-97364073
KCNQ1	rs129074	chr11:2,466,221-2,870,340	AX-50051717
KCNQ1	rs231848	chr11:2,466,221-2,870,340	AX-97260959
KCNQ1	rs142279615	chr11:2,466,221-2,870,340	AX-96973990
KCNQ1	rs142279615	chr11:2,466,221-2,870,340	AX-97440342
KCNQ1	rs4930149	chr11:2,466,221-2,870,340	AX-39041909
KCNQ1	rs231847	chr11:2,466,221-2,870,340	AX-30018173
KCNQ1	rs75429592	chr11:2,466,221-2,870,340	AX-30018181
KCNQ1	rs231846	chr11:2,466,221-2,870,340	AX-96776973
KCNQ1	rs231846	chr11:2,466,221-2,870,340	AX-97344129
KCNQ1	rs231845	chr11:2,466,221-2,870,340	AX-112924609
KCNQ1	rs231845	chr11:2,466,221-2,870,340	AX-112998691
KCNQ1	rs231844	chr11:2,466,221-2,870,340	AX-96992813
KCNQ1	rs2283202	chr11:2,466,221-2,870,340	AX-39041941
KCNQ1	rs10766310	chr11:2,466,221-2,870,340	AX-30018227
KCNQ1	rs57264152	chr11:2,466,221-2,870,340	AX-30018229
KCNQ1	rs10766311	chr11:2,466,221-2,870,340	AX-96562612
KCNQ1	rs10766311	chr11:2,466,221-2,870,340	AX-96646181
KCNQ1	rs58762055	chr11:2,466,221-2,870,340	AX-30018235
KCNQ1	rs231888	chr11:2,466,221-2,870,340	AX-96644704
KCNQ1	rs231887	chr11:2,466,221-2,870,340	AX-39041967
KCNQ1	rs10832572	chr11:2,466,221-2,870,340	AX-39041973
KCNQ1	rs74046888	chr11:2,466,221-2,870,340	AX-30018259
KCNQ1	rs558301516	chr11:2,466,221-2,870,340	AX-96563331
KCNQ1	rs558301516	chr11:2,466,221-2,870,340	AX-96646900
KCNQ1	rs67886747	chr11:2,466,221-2,870,340	AX-30018275

KCNQ1	rs2283205	chr11:2,466,221-2,870,340	AX-96971954
KCNQ1	rs2283205	chr11:2,466,221-2,870,340	AX-97000052
KCNQ1	rs72850292	chr11:2,466,221-2,870,340	AX-30018287
KCNQ1	rs2283206	chr11:2,466,221-2,870,340	AX-30018361
KCNQ1	rs231918	chr11:2,466,221-2,870,340	AX-96775885
KCNQ1	rs231918	chr11:2,466,221-2,870,340	AX-97407864
KCNQ1	rs11600199	chr11:2,466,221-2,870,340	AX-39042035
KCNQ1	rs2283208	chr11:2,466,221-2,870,340	AX-39042045
KCNQ1	rs231917	chr11:2,466,221-2,870,340	AX-96562556
KCNQ1	rs231917	chr11:2,466,221-2,870,340	AX-96646125
KCNQ1	rs129072	chr11:2,466,221-2,870,340	AX-30018463
KCNQ1	rs7949421	chr11:2,466,221-2,870,340	AX-96960431
KCNQ1	rs7949421	chr11:2,466,221-2,870,340	AX-97383751
KCNQ1	rs231916	chr11:2,466,221-2,870,340	AX-39042131
KCNQ1	rs189161	chr11:2,466,221-2,870,340	AX-30018609
KCNQ1	rs2283212	chr11:2,466,221-2,870,340	AX-96561335
KCNQ1	rs2283212	chr11:2,466,221-2,870,340	AX-96644904
KCNQ1	rs231915	chr11:2,466,221-2,870,340	AX-39042153
KCNQ1	rs231914	chr11:2,466,221-2,870,340	AX-39042165
KCNQ1	rs11023925	chr11:2,466,221-2,870,340	AX-30018667
KCNQ1	rs231913	chr11:2,466,221-2,870,340	AX-96793823
KCNQ1	rs231913	chr11:2,466,221-2,870,340	AX-97260699
KCNQ1	rs231912	chr11:2,466,221-2,870,340	AX-30018705
KCNQ1	rs231911	chr11:2,466,221-2,870,340	AX-30018707
KCNQ1	rs170786	chr11:2,466,221-2,870,340	AX-39042191
KCNQ1	rs2283214	chr11:2,466,221-2,870,340	AX-39042193
KCNQ1	rs231910	chr11:2,466,221-2,870,340	AX-39042205
KCNQ1	rs78599729	chr11:2,466,221-2,870,340	AX-30018731
KCNQ1	rs231909	chr11:2,466,221-2,870,340	AX-30018735
KCNQ1	rs231908	chr11:2,466,221-2,870,340	AX-96564166
KCNQ1	rs78677456	chr11:2,466,221-2,870,340	AX-30018751
KCNQ1	rs72844252	chr11:2,466,221-2,870,340	AX-30018753
KCNQ1	---	chr11:2,466,221-2,870,340	AX-39042227
KCNQ1	rs231906	chr11:2,466,221-2,870,340	AX-92330902
KCNQ1	rs231905	chr11:2,466,221-2,870,340	AX-30018765
KCNQ1	rs2283217	chr11:2,466,221-2,870,340	AX-96563987
KCNQ1	rs2283217	chr11:2,466,221-2,870,340	AX-96647556
KCNQ1	rs2283218	chr11:2,466,221-2,870,340	AX-96561980
KCNQ1	rs1110724	chr11:2,466,221-2,870,340	AX-96560539
KCNQ1	rs1110724	chr11:2,466,221-2,870,340	AX-96644108
KCNQ1	---	chr11:2,466,221-2,870,340	AX-30018827
KCNQ1	---	chr11:2,466,221-2,870,340	AX-96561107
KCNQ1	rs55641944	chr11:2,466,221-2,870,340	AX-30018839
KCNQ1	rs55641944	chr11:2,466,221-2,870,340	AX-96644054
KCNQ1	rs77376282	chr11:2,466,221-2,870,340	AX-30018841
KCNQ1	rs422316	chr11:2,466,221-2,870,340	AX-39042291
KCNQ1	rs422314	chr11:2,466,221-2,870,340	AX-96799631
KCNQ1	rs422314	chr11:2,466,221-2,870,340	AX-96827729
KCNQ1	rs2283220	chr11:2,466,221-2,870,340	AX-30018851
KCNQ1	rs11023936	chr11:2,466,221-2,870,340	AX-113476977
KCNQ1	rs11023936	chr11:2,466,221-2,870,340	AX-113885784
KCNQ1	rs2283221	chr11:2,466,221-2,870,340	AX-113403085
KCNQ1	rs2283221	chr11:2,466,221-2,870,340	AX-113476978
KCNQ1	rs2283222	chr11:2,466,221-2,870,340	AX-39042297
KCNQ1	rs2839990	chr11:2,466,221-2,870,340	AX-96980208

KCNQ1	rs2839990	chr11:2,466,221-2,870,340	AX-96991048
KCNQ1	rs450852	chr11:2,466,221-2,870,340	AX-39042309
KCNQ1	rs11023938	chr11:2,466,221-2,870,340	AX-30018923
KCNQ1	rs72844254	chr11:2,466,221-2,870,340	AX-96561292
KCNQ1	rs75636381	chr11:2,466,221-2,870,340	AX-37510641
KCNQ1	rs108961	chr11:2,466,221-2,870,340	AX-96565001
KCNQ1	rs108961	chr11:2,466,221-2,870,340	AX-96648570
KCNQ1	rs17744036	chr11:2,466,221-2,870,340	AX-39042363
KCNQ1	rs231880	chr11:2,466,221-2,870,340	AX-39042367
KCNQ1	rs12269744	chr11:2,466,221-2,870,340	AX-96562988
KCNQ1	rs12269744	chr11:2,466,221-2,870,340	AX-96646557
KCNQ1	rs1079714	chr11:2,466,221-2,870,340	AX-39042399
KCNQ1	rs231879	chr11:2,466,221-2,870,340	AX-39042415
KCNQ1	rs7114479	chr11:2,466,221-2,870,340	AX-96562319
KCNQ1	rs7114479	chr11:2,466,221-2,870,340	AX-96645888
KCNQ1	rs116164476	chr11:2,466,221-2,870,340	AX-96960909
KCNQ1	rs116164476	chr11:2,466,221-2,870,340	AX-97448247
KCNQ1	rs231878	chr11:2,466,221-2,870,340	AX-30019113
KCNQ1	rs2237875	chr11:2,466,221-2,870,340	AX-96771651
KCNQ1	rs2237875	chr11:2,466,221-2,870,340	AX-96786677
KCNQ1	rs78704061	chr11:2,466,221-2,870,340	AX-30019137
KCNQ1	rs2237876	chr11:2,466,221-2,870,340	AX-39042491
KCNQ1	rs74465173	chr11:2,466,221-2,870,340	AX-30019185
KCNQ1	rs2237877	chr11:2,466,221-2,870,340	AX-39042519
KCNQ1	rs2237878	chr11:2,466,221-2,870,340	AX-11377422
KCNQ1	rs74623003	chr11:2,466,221-2,870,340	AX-30019213
KCNQ1	rs231900	chr11:2,466,221-2,870,340	AX-96565391
KCNQ1	rs1116809	chr11:2,466,221-2,870,340	AX-97314503
KCNQ1	rs231899	chr11:2,466,221-2,870,340	AX-39042567
KCNQ1	rs1124811	chr11:2,466,221-2,870,340	AX-30019259
KCNQ1	rs16928624	chr11:2,466,221-2,870,340	AX-30019297
KCNQ1	rs74049745	chr11:2,466,221-2,870,340	AX-30019307
KCNQ1	rs7394507	chr11:2,466,221-2,870,340	AX-96810724
KCNQ1	rs147019170	chr11:2,466,221-2,870,340	AX-96838394
KCNQ1	rs231897	chr11:2,466,221-2,870,340	AX-30019345
KCNQ1	rs9888189	chr11:2,466,221-2,870,340	AX-144899903
KCNQ1	rs9888189	chr11:2,466,221-2,870,340	AX-86899498
KCNQ1	rs9888191	chr11:2,466,221-2,870,340	AX-88734096
KCNQ1	rs191678395	chr11:2,466,221-2,870,340	AX-92471715
KCNQ1	rs191678395	chr11:2,466,221-2,870,340	AX-96565163
KCNQ1	rs184356106	chr11:2,466,221-2,870,340	AX-105126776
KCNQ1	rs184356106	chr11:2,466,221-2,870,340	AX-96565075
KCNQ1	rs188794780	chr11:2,466,221-2,870,340	AX-96561624
KCNQ1	rs181943535	chr11:2,466,221-2,870,340	AX-96825534
KCNQ1	rs181943535	chr11:2,466,221-2,870,340	AX-97373820
KCNQ1	rs186376420	chr11:2,466,221-2,870,340	AX-96793650
KCNQ1	rs186376420	chr11:2,466,221-2,870,340	AX-97259369
KCNQ1	rs199596974	chr11:2,466,221-2,870,340	AX-96983009
KCNQ1	rs199596974	chr11:2,466,221-2,870,340	AX-97244934
KCNQ1	rs113369546	chr11:2,466,221-2,870,340	AX-96560628
KCNQ1	rs113369546	chr11:2,466,221-2,870,340	AX-96644197
KCNQ1	rs231896	chr11:2,466,221-2,870,340	AX-92479482
KCNQ1	rs231896	chr11:2,466,221-2,870,340	AX-96561489
KCNQ1	rs112425312	chr11:2,466,221-2,870,340	AX-148758485
KCNQ1	rs12287110	chr11:2,466,221-2,870,340	AX-144899904

KCNQ1	rs12287110	chr11:2,466,221-2,870,340	AX-144921510
KCNQ1	rs231895	chr11:2,466,221-2,870,340	AX-96648692
KCNQ1	rs231894	chr11:2,466,221-2,870,340	AX-96563890
KCNQ1	rs231893	chr11:2,466,221-2,870,340	AX-30019411
KCNQ1	rs231892	chr11:2,466,221-2,870,340	AX-30019433
KCNQ1	rs75898263	chr11:2,466,221-2,870,340	AX-30019449
KCNQ1	rs55763223	chr11:2,466,221-2,870,340	AX-30019457
KCNQ1	rs1971929	chr11:2,466,221-2,870,340	AX-16574066
KCNQ1	rs71476688	chr11:2,466,221-2,870,340	AX-30019467
KCNQ1	rs2056892	chr11:2,466,221-2,870,340	AX-39042679
KCNQ1	rs2018355	chr11:2,466,221-2,870,340	AX-30019475
KCNQ1	rs84178	chr11:2,466,221-2,870,340	AX-11669065
KCNQ1	rs151291	chr11:2,466,221-2,870,340	AX-39042699
KCNQ1	rs151292	chr11:2,466,221-2,870,340	AX-30019525
KCNQ1	rs74049749	chr11:2,466,221-2,870,340	AX-30019527
KCNQ1	rs11023994	chr11:2,466,221-2,870,340	AX-30019551
KCNQ1	rs231890	chr11:2,466,221-2,870,340	AX-39042727
KCNQ1	rs7948952	chr11:2,466,221-2,870,340	AX-11655528
KCNQ1	rs34441414	chr11:2,466,221-2,870,340	AX-96852331
KCNQ1	rs11023996	chr11:2,466,221-2,870,340	AX-11138612
KCNQ1	rs151219	chr11:2,466,221-2,870,340	AX-96563196
KCNQ1	rs151219	chr11:2,466,221-2,870,340	AX-96646765
KCNQ1	rs12797350	chr11:2,466,221-2,870,340	AX-96821929
KCNQ1	rs12797350	chr11:2,466,221-2,870,340	AX-97477018
KCNQ1	rs7924946	chr11:2,466,221-2,870,340	AX-30019585
KCNQ1	rs231889	chr11:2,466,221-2,870,340	AX-39042749
KCNQ1	rs115640247	chr11:2,466,221-2,870,340	AX-30019597
KCNQ1	rs12804860	chr11:2,466,221-2,870,340	AX-96970224
KCNQ1	rs12804860	chr11:2,466,221-2,870,340	AX-97469235
KCNQ1	rs9645650	chr11:2,466,221-2,870,340	AX-30019621
KCNQ1	rs11023999	chr11:2,466,221-2,870,340	AX-11138613
KCNQ1	rs56084107	chr11:2,466,221-2,870,340	AX-96560069
KCNQ1	rs56084107	chr11:2,466,221-2,870,340	AX-96643638
KCNQ1	rs6578296	chr11:2,466,221-2,870,340	AX-11573325
KCNQ1	rs200347667	chr11:2,466,221-2,870,340	AX-96561785
KCNQ1	rs201141910	chr11:2,466,221-2,870,340	AX-96644268
KCNQ1	rs179785	chr11:2,466,221-2,870,340	AX-16574207
KCNQ1	rs463535	chr11:2,466,221-2,870,340	AX-39042845
KCNQ1	rs179784	chr11:2,466,221-2,870,340	AX-113406651
KCNQ1	rs12789600	chr11:2,466,221-2,870,340	AX-96936792
KCNQ1	rs12789600	chr11:2,466,221-2,870,340	AX-96964492
KCNQ1	rs34861825	chr11:2,466,221-2,870,340	AX-96803943
KCNQ1	rs34861825	chr11:2,466,221-2,870,340	AX-96823424
KCNQ1	rs548566	chr11:2,466,221-2,870,340	AX-84610499
KCNQ1	rs548566	chr11:2,466,221-2,870,340	AX-84622116
KCNQ1	rs59777294	chr11:2,466,221-2,870,340	AX-96762679
KCNQ1	rs231877	chr11:2,466,221-2,870,340	AX-30019793
KCNQ1	rs231876	chr11:2,466,221-2,870,340	AX-97363964
KCNQ1	rs231875	chr11:2,466,221-2,870,340	AX-113476979
KCNQ1	rs231875	chr11:2,466,221-2,870,340	AX-113885785
KCNQ1	rs11024017	chr11:2,466,221-2,870,340	AX-39042891
KCNQ1	rs4430486	chr11:2,466,221-2,870,340	AX-39042893
KCNQ1	rs231873	chr11:2,466,221-2,870,340	AX-39042895
KCNQ1	rs4255520	chr11:2,466,221-2,870,340	AX-11498550
KCNQ1	rs79291597	chr11:2,466,221-2,870,340	AX-16574296

KCNQ1	rs75297165	chr11:2,466,221-2,870,340	AX-156270474
KCNQ1	rs75297165	chr11:2,466,221-2,870,340	AX-156289290
KCNQ1	rs74530907	chr11:2,466,221-2,870,340	AX-30019871
KCNQ1	rs74565893	chr11:2,466,221-2,870,340	AX-30019873
KCNQ1	rs151288	chr11:2,466,221-2,870,340	AX-16574301
KCNQ1	rs117143472	chr11:2,466,221-2,870,340	AX-54514078
KCNQ1	rs231872	chr11:2,466,221-2,870,340	AX-96747664
KCNQ1	rs67004488	chr11:2,466,221-2,870,340	AX-30019903
KCNQ1	rs12795571	chr11:2,466,221-2,870,340	AX-39042941
KCNQ1	rs63934	chr11:2,466,221-2,870,340	AX-39042943
KCNQ1	rs163160	chr11:2,466,221-2,870,340	AX-39042951
KCNQ1	rs2075870	chr11:2,466,221-2,870,340	AX-91979435
KCNQ1	rs2075870	chr11:2,466,221-2,870,340	AX-92004562
KCNQ1	---	chr11:2,466,221-2,870,340	AX-90074940
KCNQ1	---	chr11:2,466,221-2,870,340	AX-90060585
KCNQ1	---	chr11:2,466,221-2,870,340	AX-90048835
KCNQ1	---	chr11:2,466,221-2,870,340	AX-90063438
KCNQ1	---	chr11:2,466,221-2,870,340	AX-90061731
KCNQ1	---	chr11:2,466,221-2,870,340	AX-83128858
KCNQ1	---	chr11:2,466,221-2,870,340	AX-90062594
KCNQ1	---	chr11:2,466,221-2,870,340	AX-83404979
KCNQ1	---	chr11:2,466,221-2,870,340	AX-90032494
KCNQ1	---	chr11:2,466,221-2,870,340	AX-90059571
KCNQ1	---	chr11:2,466,221-2,870,340	AX-90066194
KCNQ1	---	chr11:2,466,221-2,870,340	AX-90030972
KCNQ1	---	chr11:2,466,221-2,870,340	AX-90064332
KCNQ1	rs120074188	chr11:2,466,221-2,870,340	AX-86554371
KCNQ1	---	chr11:2,466,221-2,870,340	AX-90033827
KCNQ1	rs199472792	chr11:2,466,221-2,870,340	AX-90033722
KCNQ1	rs397508097	chr11:2,466,221-2,870,340	AX-90058111
KCNQ1	rs11024034	chr11:2,466,221-2,870,340	AX-39042955
KCNQ1	rs163158	chr11:2,466,221-2,870,340	AX-39042963
KCNQ1	rs2237882	chr11:2,466,221-2,870,340	AX-96646417
KCNQ1	rs12419355	chr11:2,466,221-2,870,340	AX-50052041
KCNQ1	rs4930007	chr11:2,466,221-2,870,340	AX-96563217
KCNQ1	rs4930007	chr11:2,466,221-2,870,340	AX-96646786
KCNQ1	rs12420943	chr11:2,466,221-2,870,340	AX-30020091
KCNQ1	rs163152	chr11:2,466,221-2,870,340	AX-30020097
KCNQ1	rs233438	chr11:2,466,221-2,870,340	AX-30020099
KCNQ1	rs73419399	chr11:2,466,221-2,870,340	AX-30020101
KCNQ1	rs233437	chr11:2,466,221-2,870,340	AX-156270476
KCNQ1	rs233437	chr11:2,466,221-2,870,340	AX-156289292
KCNQ1	rs80305020	chr11:2,466,221-2,870,340	AX-96935753
KCNQ1	rs79969904	chr11:2,466,221-2,870,340	AX-156270478
KCNQ1	rs79969904	chr11:2,466,221-2,870,340	AX-156289294
KCNQ1	rs56182401	chr11:2,466,221-2,870,340	AX-156265998
KCNQ1	rs336982	chr11:2,466,221-2,870,340	AX-30020131
KCNQ1	rs12801888	chr11:2,466,221-2,870,340	AX-96922730
KCNQ1	rs12801888	chr11:2,466,221-2,870,340	AX-97409020
KCNQ1	rs35993725	chr11:2,466,221-2,870,340	AX-30020157
KCNQ1	rs11024045	chr11:2,466,221-2,870,340	AX-96925420
KCNQ1	rs11024045	chr11:2,466,221-2,870,340	AX-97393330
KCNQ1	rs11826085	chr11:2,466,221-2,870,340	AX-30020159
KCNQ1	rs114274484	chr11:2,466,221-2,870,340	AX-30020163
KCNQ1	rs233435	chr11:2,466,221-2,870,340	AX-30020187

KCNQ1	---	chr11:2,466,221-2,870,340	AX-90029713
KCNQ1	---	chr11:2,466,221-2,870,340	AX-90034978
KCNQ1	---	chr11:2,466,221-2,870,340	AX-90031745
KCNQ1	---	chr11:2,466,221-2,870,340	AX-90035459
KCNQ1	---	chr11:2,466,221-2,870,340	AX-90035648
KCNQ1	---	chr11:2,466,221-2,870,340	AX-86692996
KCNQ1	---	chr11:2,466,221-2,870,340	AX-90029008
KCNQ1	---	chr11:2,466,221-2,870,340	AX-90033747
KCNQ1	rs1057128	chr11:2,466,221-2,870,340	AX-39043031
KCNQ1	---	chr11:2,466,221-2,870,340	AX-90065743
KCNQ1	---	chr11:2,466,221-2,870,340	AX-90056435
KCNQ1	---	chr11:2,466,221-2,870,340	AX-90027834
KCNQ1	---	chr11:2,466,221-2,870,340	AX-90026922
KCNQ1	---	chr11:2,466,221-2,870,340	AX-90062886
KCNQ1	---	chr11:2,466,221-2,870,340	AX-90073910
KCNQ1	rs163150	chr11:2,466,221-2,870,340	AX-39043033
KCNQ1	rs163149	chr11:2,466,221-2,870,340	AX-96647902
KCNQ1	rs28730759	chr11:2,466,221-2,870,340	AX-30020233
KCNQ1	rs12417409	chr11:2,466,221-2,870,340	AX-30020237
KCNQ1	rs56021860	chr11:2,466,221-2,870,340	AX-30020243
KCNQ1	rs55812754	chr11:2,466,221-2,870,340	AX-30020245
KCNQ1	rs3852523	chr11:2,466,221-2,870,340	AX-96924058
KCNQ1	rs3852523	chr11:2,466,221-2,870,340	AX-97462314
KCNQ1	---	chr11:2,466,221-2,870,340	AX-90027181
KCNQ1	---	chr11:2,466,221-2,870,340	AX-90064048
KCNQ1	---	chr11:2,466,221-2,870,340	AX-90034966
KCNQ1	---	chr11:2,466,221-2,870,340	AX-90032671
KCNQ1	---	chr11:2,466,221-2,870,340	AX-90036194
KCNQ1	---	chr11:2,466,221-2,870,340	AX-90036011
KCNQ1	---	chr11:2,466,221-2,870,340	AX-90080407
KCNQ1	---	chr11:2,466,221-2,870,340	AX-90036260
KCNQ1	---	chr11:2,466,221-2,870,340	AX-90075052
KCNQ1	---	chr11:2,466,221-2,870,340	AX-90032413
KCNQ1	---	chr11:2,466,221-2,870,340	AX-90048605
KCNQ1	rs81204	chr11:2,466,221-2,870,340	AX-39043047
KCNQ1	rs163147	chr11:2,466,221-2,870,340	AX-30020269
KCNQ1	rs81205	chr11:2,466,221-2,870,340	AX-30020273
KCNQ1	rs144087038	chr11:2,466,221-2,870,340	AX-96644259
KCNQ1	rs17221854	chr11:2,466,221-2,870,340	AX-90067145
KCNQ1	---	chr11:2,466,221-2,870,340	AX-90065594
KCNQ1	rs199472811	chr11:2,466,221-2,870,340	AX-90038747
KCNQ1	---	chr11:2,466,221-2,870,340	AX-90060455
KCNQ1	rs120074189	chr11:2,466,221-2,870,340	AX-90040398
KCNQ1	---	chr11:2,466,221-2,870,340	AX-90032152
KCNQ1	---	chr11:2,466,221-2,870,340	AX-90058855
KCNQ1	---	chr11:2,466,221-2,870,340	AX-90059569
KCNQ1	---	chr11:2,466,221-2,870,340	AX-86544952
KCNQ1	---	chr11:2,466,221-2,870,340	AX-90030124
KCNQ1	---	chr11:2,466,221-2,870,340	AX-90043599
KCNQ1	---	chr11:2,466,221-2,870,340	AX-90036328
KCNQ1	---	chr11:2,466,221-2,870,340	AX-90032818
KCNQ1	---	chr11:2,466,221-2,870,340	AX-90060552
KCNQ1	---	chr11:2,466,221-2,870,340	AX-90028870
KCNQ1	rs2237884	chr11:2,466,221-2,870,340	AX-96562900
KCNQ1	rs5789271	chr11:2,466,221-2,870,340	AX-96645675

KCNQ1	rs12794000	chr11:2,466,221-2,870,340	AX-30020331
KCNQ1	rs74049757	chr11:2,466,221-2,870,340	AX-30020351
KCNQ1	rs112297325	chr11:2,466,221-2,870,340	AX-96927262
KCNQ1	rs112297325	chr11:2,466,221-2,870,340	AX-97439908
KCNQ1	rs234886	chr11:2,466,221-2,870,340	AX-39043077
KCNQ1	rs74655828	chr11:2,466,221-2,870,340	AX-30020399
KCNQ1	rs79929817	chr11:2,466,221-2,870,340	AX-30020465
KCNQ1	rs233426	chr11:2,466,221-2,870,340	AX-113821678
KCNQ1	rs233426	chr11:2,466,221-2,870,340	AX-156265999
KCNQ1	rs234872	chr11:2,466,221-2,870,340	AX-96563655
KCNQ1	rs234872	chr11:2,466,221-2,870,340	AX-96647224
KCNQ1	rs10832699	chr11:2,466,221-2,870,340	AX-96734507
KCNQ1	rs10832699	chr11:2,466,221-2,870,340	AX-97361404
KCNQ1	rs2411883	chr11:2,466,221-2,870,340	AX-30020507
KCNQ1	rs234873	chr11:2,466,221-2,870,340	AX-39043155
KCNQ1	---	chr11:2,466,221-2,870,340	AX-96807012
KCNQ1	---	chr11:2,466,221-2,870,340	AX-96822158
KCNQ1	rs76092236	chr11:2,466,221-2,870,340	AX-30020517
KCNQ1	rs11024084	chr11:2,466,221-2,870,340	AX-30020525
KCNQ1	rs3899907	chr11:2,466,221-2,870,340	AX-156270479
KCNQ1	rs3899907	chr11:2,466,221-2,870,340	AX-156289295
KCNQ1	rs33915190	chr11:2,466,221-2,870,340	AX-96949397
KCNQ1	rs33915190	chr11:2,466,221-2,870,340	AX-97410935
KCNQ1	rs12271773	chr11:2,466,221-2,870,340	AX-30020533
KCNQ1	rs147129460	chr11:2,466,221-2,870,340	AX-96563021
KCNQ1	rs147129460	chr11:2,466,221-2,870,340	AX-96646590
KCNQ1	rs149145	chr11:2,466,221-2,870,340	AX-96734541
KCNQ1	rs149145	chr11:2,466,221-2,870,340	AX-96754136
KCNQ1	rs2237885	chr11:2,466,221-2,870,340	AX-112999633
KCNQ1	rs149373	chr11:2,466,221-2,870,340	AX-30020545
KCNQ1	rs234875	chr11:2,466,221-2,870,340	AX-30020547
KCNQ1	rs2237886	chr11:2,466,221-2,870,340	AX-39043197
KCNQ1	rs33985488	chr11:2,466,221-2,870,340	AX-96843504
KCNQ1	rs149419928	chr11:2,466,221-2,870,340	AX-96829103
KCNQ1	rs199870336	chr11:2,466,221-2,870,340	AX-96562869
KCNQ1	rs11024104	chr11:2,466,221-2,870,340	AX-30020625
KCNQ1	rs163163	chr11:2,466,221-2,870,340	AX-96898619
KCNQ1	rs163163	chr11:2,466,221-2,870,340	AX-97429710
KCNQ1	rs61869764	chr11:2,466,221-2,870,340	AX-96949724
KCNQ1	rs61869764	chr11:2,466,221-2,870,340	AX-97334293
KCNQ1	rs61479768	chr11:2,466,221-2,870,340	AX-96561039
KCNQ1	rs201833716	chr11:2,466,221-2,870,340	AX-96563474
KCNQ1	rs201833716	chr11:2,466,221-2,870,340	AX-96647043
KCNQ1	---	chr11:2,466,221-2,870,340	AX-30020667
KCNQ1	rs234877	chr11:2,466,221-2,870,340	AX-30020673
KCNQ1	rs35569550	chr11:2,466,221-2,870,340	AX-30020679
KCNQ1	rs336986	chr11:2,466,221-2,870,340	AX-30020681
KCNQ1	rs115950318	chr11:2,466,221-2,870,340	AX-37510671
KCNQ1	rs2074200	chr11:2,466,221-2,870,340	AX-39043269
KCNQ1	rs234879	chr11:2,466,221-2,870,340	AX-30020729
KCNQ1	rs234880	chr11:2,466,221-2,870,340	AX-30020737
KCNQ1	rs116777634	chr11:2,466,221-2,870,340	AX-156270480
KCNQ1	rs116777634	chr11:2,466,221-2,870,340	AX-156289296
KCNQ1	rs79264824	chr11:2,466,221-2,870,340	AX-156270481
KCNQ1	rs79264824	chr11:2,466,221-2,870,340	AX-156289297

KCNQ1	rs234881	chr11:2,466,221-2,870,340	AX-30020743
KCNQ1	rs234882	chr11:2,466,221-2,870,340	AX-96563159
KCNQ1	rs3835012	chr11:2,466,221-2,870,340	AX-88766573
KCNQ1	---	chr11:2,466,221-2,870,340	AX-113175402
KCNQ1	rs75353552	chr11:2,466,221-2,870,340	AX-30020785
KCNQ1	rs233442	chr11:2,466,221-2,870,340	AX-30020795
KCNQ1	---	chr11:2,466,221-2,870,340	AX-30020797
KCNQ1	rs123379	chr11:2,466,221-2,870,340	AX-30020805
KCNQ1	rs3852525	chr11:2,466,221-2,870,340	AX-96561987
KCNQ1	rs3852525	chr11:2,466,221-2,870,340	AX-96645556
KCNQ1	rs233440	chr11:2,466,221-2,870,340	AX-96560688
KCNQ1	rs233440	chr11:2,466,221-2,870,340	AX-96644257
KCNQ1	rs78013	chr11:2,466,221-2,870,340	AX-96561798
KCNQ1	rs116463395	chr11:2,466,221-2,870,340	AX-30020831
KCNQ1	rs151316	chr11:2,466,221-2,870,340	AX-39043311
KCNQ1	rs79677993	chr11:2,466,221-2,870,340	AX-30020841
KCNQ1	rs61869807	chr11:2,466,221-2,870,340	AX-156270482
KCNQ1	rs61869807	chr11:2,466,221-2,870,340	AX-156289298
KCNQ1	rs11024137	chr11:2,466,221-2,870,340	AX-39043319
KCNQ1	rs7112108	chr11:2,466,221-2,870,340	AX-30020867
KCNQ1	rs163172	chr11:2,466,221-2,870,340	AX-30020873
KCNQ1	rs78768485	chr11:2,466,221-2,870,340	AX-30020897
KCNQ1	rs163171	chr11:2,466,221-2,870,340	AX-39043347
KCNQ1	rs75546812	chr11:2,466,221-2,870,340	AX-30020903
KCNQ1	rs115693586	chr11:2,466,221-2,870,340	AX-30020907
KCNQ1	rs163170	chr11:2,466,221-2,870,340	AX-30020915
KCNQ1	rs151290	chr11:2,466,221-2,870,340	AX-96091048
KCNQ1	rs78878449	chr11:2,466,221-2,870,340	AX-156270483
KCNQ1	rs78878449	chr11:2,466,221-2,870,340	AX-156289299
KCNQ1	rs78868563	chr11:2,466,221-2,870,340	AX-96809634
KCNQ1	rs78868563	chr11:2,466,221-2,870,340	AX-97251336
KCNQ1	rs163169	chr11:2,466,221-2,870,340	AX-30020991
KCNQ1	rs17744869	chr11:2,466,221-2,870,340	AX-96564545
KCNQ1	rs17744869	chr11:2,466,221-2,870,340	AX-96648114
KCNQ1	rs163168	chr11:2,466,221-2,870,340	AX-30021005
KCNQ1	---	chr11:2,466,221-2,870,340	AX-96560930
KCNQ1	---	chr11:2,466,221-2,870,340	AX-96644499
KCNQ1	rs151289	chr11:2,466,221-2,870,340	AX-96828427
KCNQ1	rs151289	chr11:2,466,221-2,870,340	AX-97325734
KCNQ1	rs163167	chr11:2,466,221-2,870,340	AX-156278925
KCNQ1	rs163167	chr11:2,466,221-2,870,340	AX-156294965
KCNQ1	rs2074196	chr11:2,466,221-2,870,340	AX-39043399
KCNQ1	rs163166	chr11:2,466,221-2,870,340	AX-113897492
KCNQ1	rs2074197	chr11:2,466,221-2,870,340	AX-39043413
KCNQ1	rs11602964	chr11:2,466,221-2,870,340	AX-39043429
KCNQ1	rs163165	chr11:2,466,221-2,870,340	AX-30021043
KCNQ1	rs74602888	chr11:2,466,221-2,870,340	AX-30021047
KCNQ1	rs10766379	chr11:2,466,221-2,870,340	AX-39043433
KCNQ1	rs163164	chr11:2,466,221-2,870,340	AX-112934182
KCNQ1	rs163164	chr11:2,466,221-2,870,340	AX-113973362
KCNQ1	rs3852527	chr11:2,466,221-2,870,340	AX-96915223
KCNQ1	rs3852527	chr11:2,466,221-2,870,340	AX-96917395
KCNQ1	rs3852528	chr11:2,466,221-2,870,340	AX-39043451
KCNQ1	rs3852529	chr11:2,466,221-2,870,340	AX-30021091
KCNQ1	rs72844287	chr11:2,466,221-2,870,340	AX-30021115

KCNQ1	rs11601574	chr11:2,466,221-2,870,340	AX-96734371
KCNQ1	rs11601574	chr11:2,466,221-2,870,340	AX-97262621
KCNQ1	rs59809880	chr11:2,466,221-2,870,340	AX-30021145
KCNQ1	rs76173700	chr11:2,466,221-2,870,340	AX-30021159
KCNQ1	rs11024175	chr11:2,466,221-2,870,340	AX-30021163
KCNQ1	rs7943684	chr11:2,466,221-2,870,340	AX-30021173
KCNQ1	rs58945252	chr11:2,466,221-2,870,340	AX-96753165
KCNQ1	rs58945252	chr11:2,466,221-2,870,340	AX-97465952
KCNQ1	rs8181588	chr11:2,466,221-2,870,340	AX-39043503
KCNQ1	rs9971505	chr11:2,466,221-2,870,340	AX-30021203
KCNQ1	rs112466472	chr11:2,466,221-2,870,340	AX-30021207
KCNQ1	rs2237888	chr11:2,466,221-2,870,340	AX-55765344
KCNQ1	rs2237888	chr11:2,466,221-2,870,340	AX-97441900
KCNQ1	rs4930008	chr11:2,466,221-2,870,340	AX-30021245
KCNQ1	rs11024184	chr11:2,466,221-2,870,340	AX-30021259
KCNQ1	rs71029160	chr11:2,466,221-2,870,340	AX-96993166
KCNQ1	rs234842	chr11:2,466,221-2,870,340	AX-96847035
KCNQ1	rs121256	chr11:2,466,221-2,870,340	AX-30021323
KCNQ1	rs2237890	chr11:2,466,221-2,870,340	AX-96875845
KCNQ1	rs2237890	chr11:2,466,221-2,870,340	AX-96886584
KCNQ1	rs123381	chr11:2,466,221-2,870,340	AX-96563609
KCNQ1	rs123381	chr11:2,466,221-2,870,340	AX-96647178
KCNQ1	rs7480855	chr11:2,466,221-2,870,340	AX-39043547
KCNQ1	rs234844	chr11:2,466,221-2,870,340	AX-97001277
KCNQ1	rs234844	chr11:2,466,221-2,870,340	AX-97294274
KCNQ1	rs72844290	chr11:2,466,221-2,870,340	AX-96846859
KCNQ1	rs233446	chr11:2,466,221-2,870,340	AX-39043561
KCNQ1	rs233447	chr11:2,466,221-2,870,340	AX-50052117
KCNQ1	rs12285496	chr11:2,466,221-2,870,340	AX-39043563
KCNQ1	rs163177	chr11:2,466,221-2,870,340	AX-39043573
KCNQ1	rs12284372	chr11:2,466,221-2,870,340	AX-39043581
KCNQ1	rs16928694	chr11:2,466,221-2,870,340	AX-39043583
KCNQ1	rs2237892	chr11:2,466,221-2,870,340	AX-50943236
KCNQ1	rs2237893	chr11:2,466,221-2,870,340	AX-39043589
KCNQ1	rs233448	chr11:2,466,221-2,870,340	AX-39043603
KCNQ1	rs57845237	chr11:2,466,221-2,870,340	AX-96976476
KCNQ1	rs57845237	chr11:2,466,221-2,870,340	AX-97382568
KCNQ1	rs151293	chr11:2,466,221-2,870,340	AX-96847405
KCNQ1	rs151293	chr11:2,466,221-2,870,340	AX-97368630
KCNQ1	rs12294675	chr11:2,466,221-2,870,340	AX-39043657
KCNQ1	rs11606985	chr11:2,466,221-2,870,340	AX-39043671
KCNQ1	rs233449	chr11:2,466,221-2,870,340	AX-39043675
KCNQ1	rs233450	chr11:2,466,221-2,870,340	AX-39043679
KCNQ1	rs77450170	chr11:2,466,221-2,870,340	AX-30021525
KCNQ1	rs163182	chr11:2,466,221-2,870,340	AX-92330395
KCNQ1	rs163182	chr11:2,466,221-2,870,340	AX-92330903
KCNQ1	rs163183	chr11:2,466,221-2,870,340	AX-39043683
KCNQ1	rs77239547	chr11:2,466,221-2,870,340	AX-30021539
KCNQ1	rs7111394	chr11:2,466,221-2,870,340	AX-39043693
KCNQ1	---	chr11:2,466,221-2,870,340	AX-156269162
KCNQ1	---	chr11:2,466,221-2,870,340	AX-156288714
KCNQ1	rs234850	chr11:2,466,221-2,870,340	AX-30021579
KCNQ1	rs139647931	chr11:2,466,221-2,870,340	AX-97434768
KCNQ1	rs202159835	chr11:2,466,221-2,870,340	AX-96954198
KCNQ1	rs233451	chr11:2,466,221-2,870,340	AX-96943686

KCNQ1	rs60513508	chr11:2,466,221-2,870,340	AX-30021599
KCNQ1	rs163184	chr11:2,466,221-2,870,340	AX-96743197
KCNQ1	rs163184	chr11:2,466,221-2,870,340	AX-96745408
KCNQ1	rs2283228	chr11:2,466,221-2,870,340	AX-11381501
KCNQ1	rs3854430	chr11:2,466,221-2,870,340	AX-39043757
KCNQ1	rs79444906	chr11:2,466,221-2,870,340	AX-54514305
KCNQ1	rs234852	chr11:2,466,221-2,870,340	AX-96841176
KCNQ1	rs234852	chr11:2,466,221-2,870,340	AX-97395462
KCNQ1	rs234853	chr11:2,466,221-2,870,340	AX-39043761
KCNQ1	rs234854	chr11:2,466,221-2,870,340	AX-39043763
KCNQ1	rs234855	chr11:2,466,221-2,870,340	AX-96905207
KCNQ1	rs234855	chr11:2,466,221-2,870,340	AX-97279159
KCNQ1	rs78503325	chr11:2,466,221-2,870,340	AX-16575160
KCNQ1	rs234856	chr11:2,466,221-2,870,340	AX-97425379
KCNQ1	rs234857	chr11:2,466,221-2,870,340	AX-39043795
KCNQ1	---	chr11:2,466,221-2,870,340	AX-96904285
KCNQ1	rs234859	chr11:2,466,221-2,870,340	AX-96817701
KCNQ1	rs234859	chr11:2,466,221-2,870,340	AX-97464981
KCNQ1	rs72844296	chr11:2,466,221-2,870,340	AX-96561777
KCNQ1	rs72844296	chr11:2,466,221-2,870,340	AX-96645346
KCNQ1	rs234860	chr11:2,466,221-2,870,340	AX-39043805
KCNQ1	rs234861	chr11:2,466,221-2,870,340	AX-97315472
KCNQ1	rs234862	chr11:2,466,221-2,870,340	AX-96563873
KCNQ1	rs234862	chr11:2,466,221-2,870,340	AX-96647442
KCNQ1	---	chr11:2,466,221-2,870,340	AX-96929444
KCNQ1	---	chr11:2,466,221-2,870,340	AX-97361848
KCNQ1	rs234863	chr11:2,466,221-2,870,340	AX-96732014
KCNQ1	rs234863	chr11:2,466,221-2,870,340	AX-97359249
KCNQ1	rs11819853	chr11:2,466,221-2,870,340	AX-30021849
KCNQ1	rs77886408	chr11:2,466,221-2,870,340	AX-96755878
KCNQ1	rs77886408	chr11:2,466,221-2,870,340	AX-97293393
KCNQ1	---	chr11:2,466,221-2,870,340	AX-96956780
KCNQ1	rs2237895	chr11:2,466,221-2,870,340	AX-30021863
KCNQ1	rs60808706	chr11:2,466,221-2,870,340	AX-30021869
KCNQ1	rs234864	chr11:2,466,221-2,870,340	AX-96941825
KCNQ1	rs234865	chr11:2,466,221-2,870,340	AX-39043847
KCNQ1	rs234866	chr11:2,466,221-2,870,340	AX-30021893
KCNQ1	rs2299620	chr11:2,466,221-2,870,340	AX-113959671
KCNQ1	rs72847582	chr11:2,466,221-2,870,340	AX-30021919
KCNQ1	rs2237897	chr11:2,466,221-2,870,340	AX-50943240
KCNQ1	rs234867	chr11:2,466,221-2,870,340	AX-96565358
KCNQ1	rs234867	chr11:2,466,221-2,870,340	AX-96648927
KCNQ1	rs458069	chr11:2,466,221-2,870,340	AX-96562473
KCNQ1	rs458069	chr11:2,466,221-2,870,340	AX-96646042
KCNQ1	rs234868	chr11:2,466,221-2,870,340	AX-96563668
KCNQ1	rs234868	chr11:2,466,221-2,870,340	AX-96647237
KCNQ1	rs73421349	chr11:2,466,221-2,870,340	AX-30021927
KCNQ1	---	chr11:2,466,221-2,870,340	AX-39043869
KCNQ1	---	chr11:2,466,221-2,870,340	AX-30021929
KCNQ1	rs72847583	chr11:2,466,221-2,870,340	AX-30021933
KCNQ1	rs234869	chr11:2,466,221-2,870,340	AX-96560637
KCNQ1	rs234869	chr11:2,466,221-2,870,340	AX-96644206
KCNQ1	rs72847584	chr11:2,466,221-2,870,340	AX-96879760
KCNQ1	rs72847584	chr11:2,466,221-2,870,340	AX-96886216
KCNQ1	rs234870	chr11:2,466,221-2,870,340	AX-96739550

KCNQ1	rs234870	chr11:2,466,221-2,870,340	AX-97438273
KCNQ1	rs36157111	chr11:2,466,221-2,870,340	AX-96562205
KCNQ1	rs36157111	chr11:2,466,221-2,870,340	AX-96645774
KCNQ1	rs76863355	chr11:2,466,221-2,870,340	AX-30021947
KCNQ1	rs2583416	chr11:2,466,221-2,870,340	AX-30021949
KCNQ1	rs113030218	chr11:2,466,221-2,870,340	AX-96841510
KCNQ1	rs113030218	chr11:2,466,221-2,870,340	AX-96856469
KCNQ1	---	chr11:2,466,221-2,870,340	AX-96561359
KCNQ1	rs145254979	chr11:2,466,221-2,870,340	AX-97464433
KCNQ1	---	chr11:2,466,221-2,870,340	AX-151370684
KCNQ1	---	chr11:2,466,221-2,870,340	AX-156298531
KCNQ1	rs57823802	chr11:2,466,221-2,870,340	AX-151321457
KCNQ1	rs57823802	chr11:2,466,221-2,870,340	AX-156286374
KCNQ1	rs58579655	chr11:2,466,221-2,870,340	AX-30021997
KCNQ1	rs77919905	chr11:2,466,221-2,870,340	AX-37510719
KCNQ1	rs4930012	chr11:2,466,221-2,870,340	AX-39043879
KCNQ1	rs10741726	chr11:2,466,221-2,870,340	AX-39043885
KCNQ1	rs12284138	chr11:2,466,221-2,870,340	AX-39043889
KCNQ1	rs201591401	chr11:2,466,221-2,870,340	AX-96949594
KCNQ1	rs79774694	chr11:2,466,221-2,870,340	AX-30022017
KCNQ1	rs4930013	chr11:2,466,221-2,870,340	AX-39043891
KCNQ1	rs2283229	chr11:2,466,221-2,870,340	AX-39043895
KCNQ1	rs11024304	chr11:2,466,221-2,870,340	AX-39043905
KCNQ1	rs433052	chr11:2,466,221-2,870,340	AX-39043907
KCNQ1	rs2005833	chr11:2,466,221-2,870,340	AX-96643819
KCNQ1	rs2237898	chr11:2,466,221-2,870,340	AX-39043915
KCNQ1	rs2001170	chr11:2,466,221-2,870,340	AX-30022101
KCNQ1	rs2237899	chr11:2,466,221-2,870,340	AX-39043937
KCNQ1	rs12277369	chr11:2,466,221-2,870,340	AX-96560674
KCNQ1	rs35877729	chr11:2,466,221-2,870,340	AX-96977499
KCNQ1	rs35877729	chr11:2,466,221-2,870,340	AX-97405641
KCNQ1	rs2237900	chr11:2,466,221-2,870,340	AX-30022153
KCNQ1	rs72847587	chr11:2,466,221-2,870,340	AX-30022161
KCNQ1	rs12419030	chr11:2,466,221-2,870,340	AX-30022163
KCNQ1	rs34075935	chr11:2,466,221-2,870,340	AX-30022165
KCNQ1	rs12419347	chr11:2,466,221-2,870,340	AX-39043959
KCNQ1	rs12281297	chr11:2,466,221-2,870,340	AX-30022177
KCNQ1	rs79788804	chr11:2,466,221-2,870,340	AX-30022185
KCNQ1	rs3852520	chr11:2,466,221-2,870,340	AX-30022187
KCNQ1	---	chr11:2,466,221-2,870,340	AX-90061500
KCNQ1	---	chr11:2,466,221-2,870,340	AX-90042994
KCNQ1	---	chr11:2,466,221-2,870,340	AX-83102818
KCNQ1	---	chr11:2,466,221-2,870,340	AX-90073628
KCNQ1	---	chr11:2,466,221-2,870,340	AX-90058758
KCNQ1	---	chr11:2,466,221-2,870,340	AX-90063375
KCNQ1	---	chr11:2,466,221-2,870,340	AX-90081540
KCNQ1	rs34150427	chr11:2,466,221-2,870,340	AX-83416849
KCNQ1	---	chr11:2,466,221-2,870,340	AX-147942546
KCNQ1	rs8234	chr11:2,466,221-2,870,340	AX-39043981
KCNQ1	rs10798	chr11:2,466,221-2,870,340	AX-39043985
LAMA4	rs7758715	chr6:112,429,134-112,575,917	AX-151195108
LAMA4	rs7758715	chr6:112,429,134-112,575,917	AX-156275970
LAMA4	rs7738331	chr6:112,429,134-112,575,917	AX-156275971
LAMA4	rs7758910	chr6:112,429,134-112,575,917	AX-156275972
LAMA4	rs7758910	chr6:112,429,134-112,575,917	AX-156292992

LAMA4	rs6568718	chr6:112,429,134-112,575,917	AX-11572609
LAMA4	rs199529261	chr6:112,429,134-112,575,917	AX-151465361
LAMA4	rs199529261	chr6:112,429,134-112,575,917	AX-156297787
LAMA4	rs3734291	chr6:112,429,134-112,575,917	AX-156275973
LAMA4	rs3734291	chr6:112,429,134-112,575,917	AX-156292993
LAMA4	rs12204892	chr6:112,429,134-112,575,917	AX-147878709
LAMA4	rs12204892	chr6:112,429,134-112,575,917	AX-147972500
LAMA4	rs112343659	chr6:112,429,134-112,575,917	AX-151213403
LAMA4	---	chr6:112,429,134-112,575,917	AX-151296457
LAMA4	---	chr6:112,429,134-112,575,917	AX-156298055
LAMA4	rs73541478	chr6:112,429,134-112,575,917	AX-15194976
LAMA4	rs73541481	chr6:112,429,134-112,575,917	AX-15194977
LAMA4	rs73541482	chr6:112,429,134-112,575,917	AX-15194978
LAMA4	rs73541483	chr6:112,429,134-112,575,917	AX-15194980
LAMA4	rs11962253	chr6:112,429,134-112,575,917	AX-15194981
LAMA4	rs11153341	chr6:112,429,134-112,575,917	AX-148275933
LAMA4	rs11153341	chr6:112,429,134-112,575,917	AX-156275974
LAMA4	rs6941214	chr6:112,429,134-112,575,917	AX-156275975
LAMA4	rs6942296	chr6:112,429,134-112,575,917	AX-156275976
LAMA4	rs6942296	chr6:112,429,134-112,575,917	AX-156292995
LAMA4	rs6921882	chr6:112,429,134-112,575,917	AX-41820509
LAMA4	rs3777947	chr6:112,429,134-112,575,917	AX-15194987
LAMA4	---	chr6:112,429,134-112,575,917	AX-15194988
LAMA4	---	chr6:112,429,134-112,575,917	AX-88815315
LAMA4	rs3777946	chr6:112,429,134-112,575,917	AX-156275977
LAMA4	rs3777945	chr6:112,429,134-112,575,917	AX-15194989
LAMA4	rs3777944	chr6:112,429,134-112,575,917	AX-156275978
LAMA4	rs3777944	chr6:112,429,134-112,575,917	AX-156292997
LAMA4	rs3777943	chr6:112,429,134-112,575,917	AX-15194990
LAMA4	rs11961355	chr6:112,429,134-112,575,917	AX-15194992
LAMA4	rs10456878	chr6:112,429,134-112,575,917	AX-35507873
LAMA4	rs12191016	chr6:112,429,134-112,575,917	AX-41820515
LAMA4	rs3734290	chr6:112,429,134-112,575,917	AX-35507877
LAMA4	rs3734289	chr6:112,429,134-112,575,917	AX-15194994
LAMA4	---	chr6:112,429,134-112,575,917	AX-151201376
LAMA4	rs6916947	chr6:112,429,134-112,575,917	AX-12614227
LAMA4	rs6917142	chr6:112,429,134-112,575,917	AX-15194998
LAMA4	rs11969913	chr6:112,429,134-112,575,917	AX-41820517
LAMA4	rs1050353	chr6:112,429,134-112,575,917	AX-15194999
LAMA4	rs73541486	chr6:112,429,134-112,575,917	AX-15195002
LAMA4	rs4947169	chr6:112,429,134-112,575,917	AX-11538071
LAMA4	rs147507413	chr6:112,429,134-112,575,917	AX-119778444
LAMA4	rs73541488	chr6:112,429,134-112,575,917	AX-35507879
LAMA4	rs9481223	chr6:112,429,134-112,575,917	AX-156282397
LAMA4	rs12190980	chr6:112,429,134-112,575,917	AX-156275979
LAMA4	rs12190980	chr6:112,429,134-112,575,917	AX-156292998
LAMA4	rs7764311	chr6:112,429,134-112,575,917	AX-41820523
LAMA4	rs6908892	chr6:112,429,134-112,575,917	AX-15195004
LAMA4	rs73541491	chr6:112,429,134-112,575,917	AX-15195006
LAMA4	rs73541494	chr6:112,429,134-112,575,917	AX-15195008
LAMA4	rs1160798	chr6:112,429,134-112,575,917	AX-15195009
LAMA4	rs3216857	chr6:112,429,134-112,575,917	AX-83596595
LAMA4	---	chr6:112,429,134-112,575,917	AX-83386956
LAMA4	---	chr6:112,429,134-112,575,917	AX-83087924
LAMA4	---	chr6:112,429,134-112,575,917	AX-90044665

LAMA4	rs73541498	chr6:112,429,134-112,575,917	AX-15195010
LAMA4	rs73541501	chr6:112,429,134-112,575,917	AX-156275980
LAMA4	rs73541501	chr6:112,429,134-112,575,917	AX-156292999
LAMA4	rs73543603	chr6:112,429,134-112,575,917	AX-15195014
LAMA4	rs73530790	chr6:112,429,134-112,575,917	AX-15195015
LAMA4	rs35679345	chr6:112,429,134-112,575,917	AX-41820525
LAMA4	rs73530792	chr6:112,429,134-112,575,917	AX-15195017
LAMA4	rs73530795	chr6:112,429,134-112,575,917	AX-15195019
LAMA4	rs764196	chr6:112,429,134-112,575,917	AX-12635178
LAMA4	rs61459671	chr6:112,429,134-112,575,917	AX-15195020
LAMA4	rs111944498	chr6:112,429,134-112,575,917	AX-151396609
LAMA4	rs111944498	chr6:112,429,134-112,575,917	AX-156284674
LAMA4	rs10452631	chr6:112,429,134-112,575,917	AX-35507889
LAMA4	rs9384821	chr6:112,429,134-112,575,917	AX-11578137
LAMA4	rs9384821	chr6:112,429,134-112,575,917	AX-148309517
LAMA4	rs111326675	chr6:112,429,134-112,575,917	AX-148133459
LAMA4	rs111326675	chr6:112,429,134-112,575,917	AX-148693942
LAMA4	rs10452616	chr6:112,429,134-112,575,917	AX-41820529
LAMA4	rs10452617	chr6:112,429,134-112,575,917	AX-41820531
LAMA4	rs10452633	chr6:112,429,134-112,575,917	AX-41820533
LAMA4	rs10452635	chr6:112,429,134-112,575,917	AX-41820537
LAMA4	rs7763664	chr6:112,429,134-112,575,917	AX-41820539
LAMA4	rs4945897	chr6:112,429,134-112,575,917	AX-15195024
LAMA4	rs77699322	chr6:112,429,134-112,575,917	AX-15195027
LAMA4	rs73532603	chr6:112,429,134-112,575,917	AX-15195028
LAMA4	rs73532606	chr6:112,429,134-112,575,917	AX-15195029
LAMA4	rs73532607	chr6:112,429,134-112,575,917	AX-156275981
LAMA4	rs73532607	chr6:112,429,134-112,575,917	AX-156293000
LAMA4	rs73532610	chr6:112,429,134-112,575,917	AX-156275982
LAMA4	rs73532610	chr6:112,429,134-112,575,917	AX-156293001
LAMA4	rs11153342	chr6:112,429,134-112,575,917	AX-15195032
LAMA4	rs11962711	chr6:112,429,134-112,575,917	AX-15195033
LAMA4	rs73532613	chr6:112,429,134-112,575,917	AX-15195034
LAMA4	rs2032564	chr6:112,429,134-112,575,917	AX-119282865
LAMA4	rs146929668	chr6:112,429,134-112,575,917	AX-151424688
LAMA4	rs969138	chr6:112,429,134-112,575,917	AX-11700644
LAMA4	rs73532615	chr6:112,429,134-112,575,917	AX-15195037
LAMA4	rs969139	chr6:112,429,134-112,575,917	AX-12666891
LAMA4	rs11153343	chr6:112,429,134-112,575,917	AX-15195044
LAMA4	rs73532618	chr6:112,429,134-112,575,917	AX-15195048
LAMA4	rs77886563	chr6:112,429,134-112,575,917	AX-15195049
LAMA4	rs58881810	chr6:112,429,134-112,575,917	AX-156275983
LAMA4	rs58881810	chr6:112,429,134-112,575,917	AX-156293002
LAMA4	rs2157550	chr6:112,429,134-112,575,917	AX-15195053
LAMA4	rs66482680	chr6:112,429,134-112,575,917	AX-156286139
LAMA4	rs9387059	chr6:112,429,134-112,575,917	AX-15195054
LAMA4	rs9320393	chr6:112,429,134-112,575,917	AX-15195055
LAMA4	rs34752945	chr6:112,429,134-112,575,917	AX-11452386
LAMA4	rs200754221	chr6:112,429,134-112,575,917	AX-92597935
LAMA4	rs34172193	chr6:112,429,134-112,575,917	AX-11442390
LAMA4	rs9487823	chr6:112,429,134-112,575,917	AX-15195059
LAMA4	rs9320394	chr6:112,429,134-112,575,917	AX-15195064
LAMA4	rs141988342	chr6:112,429,134-112,575,917	AX-86602627
LAMA4	rs138232283	chr6:112,429,134-112,575,917	AX-82998154
LAMA4	rs764587	chr6:112,429,134-112,575,917	AX-15195069

LAMA4	rs3734287	chr6:112,429,134-112,575,917	AX-15195071
LAMA4	rs60125059	chr6:112,429,134-112,575,917	AX-15195072
LAMA4	---	chr6:112,429,134-112,575,917	AX-94359501
LAMA4	rs117675557	chr6:112,429,134-112,575,917	AX-15195073
LAMA4	rs13191503	chr6:112,429,134-112,575,917	AX-41820547
LAMA4	rs13191507	chr6:112,429,134-112,575,917	AX-11243702
LAMA4	rs145091507	chr6:112,429,134-112,575,917	AX-38311795
LAMA4	rs200187282	chr6:112,429,134-112,575,917	AX-156284189
LAMA4	rs720179	chr6:112,429,134-112,575,917	AX-123112886
LAMA4	rs2345806	chr6:112,429,134-112,575,917	AX-123116113
LAMA4	rs9320395	chr6:112,429,134-112,575,917	AX-156275984
LAMA4	rs9320395	chr6:112,429,134-112,575,917	AX-156293003
LAMA4	rs62413966	chr6:112,429,134-112,575,917	AX-15195080
LAMA4	rs2032565	chr6:112,429,134-112,575,917	AX-15195081
LAMA4	rs2032566	chr6:112,429,134-112,575,917	AX-156297021
LAMA4	rs1050349	chr6:112,429,134-112,575,917	AX-15195082
LAMA4	rs2032567	chr6:112,429,134-112,575,917	AX-11361428
LAMA4	rs2032568	chr6:112,429,134-112,575,917	AX-15195084
LAMA4	rs9487824	chr6:112,429,134-112,575,917	AX-11689651
LAMA4	rs75417684	chr6:112,429,134-112,575,917	AX-156275985
LAMA4	rs75417684	chr6:112,429,134-112,575,917	AX-156293004
LAMA4	rs6924043	chr6:112,429,134-112,575,917	AX-15195087
LAMA4	rs7764213	chr6:112,429,134-112,575,917	AX-156275986
LAMA4	rs7764213	chr6:112,429,134-112,575,917	AX-156293005
LAMA4	rs4947170	chr6:112,429,134-112,575,917	AX-41820561
LAMA4	rs7765769	chr6:112,429,134-112,575,917	AX-15195088
LAMA4	rs142627658	chr6:112,429,134-112,575,917	AX-120772060
LAMA4	rs41289902	chr6:112,429,134-112,575,917	AX-11492993
LAMA4	rs372615994	chr6:112,429,134-112,575,917	AX-86678671
LAMA4	rs2072019	chr6:112,429,134-112,575,917	AX-15195090
LAMA4	rs2072020	chr6:112,429,134-112,575,917	AX-15195091
LAMA4	rs7748075	chr6:112,429,134-112,575,917	AX-41820563
LAMA4	rs1016825	chr6:112,429,134-112,575,917	AX-156275987
LAMA4	rs1016825	chr6:112,429,134-112,575,917	AX-156293006
LAMA4	rs2072026	chr6:112,429,134-112,575,917	AX-11365436
LAMA4	rs35772073	chr6:112,429,134-112,575,917	AX-83389632
LAMA4	rs180931319	chr6:112,429,134-112,575,917	AX-83182553
LAMA4	rs7742931	chr6:112,429,134-112,575,917	AX-12638910
LAMA4	rs73532640	chr6:112,429,134-112,575,917	AX-15195096
LAMA4	rs60600759	chr6:112,429,134-112,575,917	AX-148134898
LAMA4	rs60600759	chr6:112,429,134-112,575,917	AX-156283278
LAMA4	rs763247	chr6:112,429,134-112,575,917	AX-35507945
LAMA4	rs6911058	chr6:112,429,134-112,575,917	AX-15195097
LAMA4	rs744006	chr6:112,429,134-112,575,917	AX-15195102
LAMA4	rs13205383	chr6:112,429,134-112,575,917	AX-11244284
LAMA4	rs28360618	chr6:112,429,134-112,575,917	AX-35507947
LAMA4	rs77129409	chr6:112,429,134-112,575,917	AX-119977833
LAMA4	rs77129409	chr6:112,429,134-112,575,917	AX-156285526
LAMA4	rs3822941	chr6:112,429,134-112,575,917	AX-15195103
LAMA4	rs62413967	chr6:112,429,134-112,575,917	AX-15195104
LAMA4	rs3777942	chr6:112,429,134-112,575,917	AX-15195106
LAMA4	rs13197881	chr6:112,429,134-112,575,917	AX-15195107
LAMA4	rs13198339	chr6:112,429,134-112,575,917	AX-35507949
LAMA4	rs13214535	chr6:112,429,134-112,575,917	AX-15195109
LAMA4	rs73536582	chr6:112,429,134-112,575,917	AX-15195112

LAMA4	rs7775823	chr6:112,429,134-112,575,917	AX-156275989
LAMA4	rs7775823	chr6:112,429,134-112,575,917	AX-156293008
LAMA4	rs147481114	chr6:112,429,134-112,575,917	AX-107872025
LAMA4	rs11969673	chr6:112,429,134-112,575,917	AX-148064592
LAMA4	rs11969673	chr6:112,429,134-112,575,917	AX-156282400
LAMA4	rs12213786	chr6:112,429,134-112,575,917	AX-12430612
LAMA4	rs79611412	chr6:112,429,134-112,575,917	AX-120594429
LAMA4	rs79611412	chr6:112,429,134-112,575,917	AX-156282401
LAMA4	rs202042801	chr6:112,429,134-112,575,917	AX-121862424
LAMA4	rs202042801	chr6:112,429,134-112,575,917	AX-156284651
LAMA4	rs3734286	chr6:112,429,134-112,575,917	AX-15195114
LAMA4	rs146868519	chr6:112,429,134-112,575,917	AX-82908102
LAMA4	rs76951179	chr6:112,429,134-112,575,917	AX-15195115
LAMA4	rs9487828	chr6:112,429,134-112,575,917	AX-41820575
LAMA4	rs117827295	chr6:112,429,134-112,575,917	AX-15195116
LAMA4	rs62413968	chr6:112,429,134-112,575,917	AX-15195118
LAMA4	rs142273488	chr6:112,429,134-112,575,917	AX-15195119
LAMA4	rs142273488	chr6:112,429,134-112,575,917	AX-156298535
LAMA4	rs35470720	chr6:112,429,134-112,575,917	AX-15195120
LAMA4	rs7758334	chr6:112,429,134-112,575,917	AX-15195122
LAMA4	rs9487829	chr6:112,429,134-112,575,917	AX-156275990
LAMA4	rs73536588	chr6:112,429,134-112,575,917	AX-15195124
LAMA4	rs12192292	chr6:112,429,134-112,575,917	AX-15195125
LAMA4	rs76303939	chr6:112,429,134-112,575,917	AX-15195126
LAMA4	rs9487830	chr6:112,429,134-112,575,917	AX-41820579
LAMA4	rs139115761	chr6:112,429,134-112,575,917	AX-151431705
LAMA4	rs59008060	chr6:112,429,134-112,575,917	AX-15195130
LAMA4	rs11153344	chr6:112,429,134-112,575,917	AX-11147439
LAMA4	rs147120087	chr6:112,429,134-112,575,917	AX-156285168
LAMA4	rs9320396	chr6:112,429,134-112,575,917	AX-11680870
LAMA4	rs12196086	chr6:112,429,134-112,575,917	AX-15195132
LAMA4	rs34956253	chr6:112,429,134-112,575,917	AX-156298059
LAMA4	rs201802315	chr6:112,429,134-112,575,917	AX-151205877
LAMA4	rs538726706	chr6:112,429,134-112,575,917	AX-86707759
LAMA4	rs2277084	chr6:112,429,134-112,575,917	AX-15195133
LAMA4	rs143269044	chr6:112,429,134-112,575,917	AX-83411311
LAMA4	---	chr6:112,429,134-112,575,917	AX-94376252
LAMA4	rs6568719	chr6:112,429,134-112,575,917	AX-156275993
LAMA4	rs6568719	chr6:112,429,134-112,575,917	AX-156293012
LAMA4	rs3798360	chr6:112,429,134-112,575,917	AX-35507969
LAMA4	rs3798359	chr6:112,429,134-112,575,917	AX-156275994
LAMA4	rs3798359	chr6:112,429,134-112,575,917	AX-156293013
LAMA4	rs3798357	chr6:112,429,134-112,575,917	AX-15195135
LAMA4	rs12200170	chr6:112,429,134-112,575,917	AX-148190001
LAMA4	rs34449915	chr6:112,429,134-112,575,917	AX-156297947
LAMA4	rs34449915	chr6:112,429,134-112,575,917	AX-35507977
LAMA4	rs2227237	chr6:112,429,134-112,575,917	AX-156282402
LAMA4	rs2227237	chr6:112,429,134-112,575,917	AX-156297022
LAMA4	rs2213838	chr6:112,429,134-112,575,917	AX-35507979
LAMA4	rs7739355	chr6:112,429,134-112,575,917	AX-11642242
LAMA4	rs3752577	chr6:112,429,134-112,575,917	AX-11478212
LAMA4	rs201962680	chr6:112,429,134-112,575,917	AX-151222587
LAMA4	rs35776093	chr6:112,429,134-112,575,917	AX-122237151
LAMA4	rs35776093	chr6:112,429,134-112,575,917	AX-38311811
LAMA4	---	chr6:112,429,134-112,575,917	AX-151121970

LAMA4	rs9487832	chr6:112,429,134-112,575,917	AX-35507993
LAMA4	rs9487833	chr6:112,429,134-112,575,917	AX-15195145
LAMA4	rs9487834	chr6:112,429,134-112,575,917	AX-35507995
LAMA4	rs7739729	chr6:112,429,134-112,575,917	AX-41820591
LAMA4	rs2282852	chr6:112,429,134-112,575,917	AX-41820593
LAMA4	---	chr6:112,429,134-112,575,917	AX-113354342
LAMA4	---	chr6:112,429,134-112,575,917	AX-156293014
LAMA4	rs78366040	chr6:112,429,134-112,575,917	AX-35507999
LAMA4	rs6913656	chr6:112,429,134-112,575,917	AX-41820597
LAMA4	rs9320398	chr6:112,429,134-112,575,917	AX-148017270
LAMA4	rs9320398	chr6:112,429,134-112,575,917	AX-153828158
LAMA4	rs12201150	chr6:112,429,134-112,575,917	AX-147985620
LAMA4	rs9487835	chr6:112,429,134-112,575,917	AX-35508003
LAMA4	rs6919131	chr6:112,429,134-112,575,917	AX-15195147
LAMA4	rs763246	chr6:112,429,134-112,575,917	AX-151111484
LAMA4	rs763246	chr6:112,429,134-112,575,917	AX-156275996
LAMA4	rs5879140	chr6:112,429,134-112,575,917	AX-122079796
LAMA4	rs5879140	chr6:112,429,134-112,575,917	AX-156285085
LAMA4	rs1060515	chr6:112,429,134-112,575,917	AX-119943693
LAMA4	rs1060515	chr6:112,429,134-112,575,917	AX-123107050
LAMA4	rs7766236	chr6:112,429,134-112,575,917	AX-11644108
LAMA4	rs17073430	chr6:112,429,134-112,575,917	AX-41820601
LAMA4	rs138153075	chr6:112,429,134-112,575,917	AX-83069603
LAMA4	rs143587921	chr6:112,429,134-112,575,917	AX-83356297
LAMA4	rs113345234	chr6:112,429,134-112,575,917	AX-156293015
LAMA4	rs971402	chr6:112,429,134-112,575,917	AX-12667071
LAMA4	rs971403	chr6:112,429,134-112,575,917	AX-11701043
LAMA4	rs971404	chr6:112,429,134-112,575,917	AX-12667072
LAMA4	rs971405	chr6:112,429,134-112,575,917	AX-41820607
LAMA4	rs11961480	chr6:112,429,134-112,575,917	AX-15195153
LAMA4	rs11963821	chr6:112,429,134-112,575,917	AX-148778790
LAMA4	rs11963821	chr6:112,429,134-112,575,917	AX-92487023
LAMA4	rs11968750	chr6:112,429,134-112,575,917	AX-122452610
LAMA4	rs11968750	chr6:112,429,134-112,575,917	AX-149272758
LAMA4	rs183462896	chr6:112,429,134-112,575,917	AX-105132129
LAMA4	rs11963846	chr6:112,429,134-112,575,917	AX-15195157
LAMA4	rs2051649	chr6:112,429,134-112,575,917	AX-11363372
LAMA4	rs6940879	chr6:112,429,134-112,575,917	AX-11595945
LAMA4	rs9387060	chr6:112,429,134-112,575,917	AX-11684910
LAMA4	rs1050348	chr6:112,429,134-112,575,917	AX-11112638
LAMA4	rs12210552	chr6:112,429,134-112,575,917	AX-35508021
LAMA4	rs77199140	chr6:112,429,134-112,575,917	AX-35508023
LAMA4	rs2345807	chr6:112,429,134-112,575,917	AX-35508027
LAMA4	rs56227047	chr6:112,429,134-112,575,917	AX-11682093
LAMA4	rs6917024	chr6:112,429,134-112,575,917	AX-15195177
LAMA4	rs73766947	chr6:112,429,134-112,575,917	AX-15195178
LAMA4	rs6917763	chr6:112,429,134-112,575,917	AX-11594265
LAMA4	---	chr6:112,429,134-112,575,917	AX-94376253
LAMA4	rs73538516	chr6:112,429,134-112,575,917	AX-15195180
LAMA4	rs9398298	chr6:112,429,134-112,575,917	AX-15195181
LAMA4	rs9398299	chr6:112,429,134-112,575,917	AX-15195183
LAMA4	rs2157544	chr6:112,429,134-112,575,917	AX-15195184
LAMA4	rs2157545	chr6:112,429,134-112,575,917	AX-15195185
LAMA4	rs3777935	chr6:112,429,134-112,575,917	AX-15195186
LAMA4	rs73766949	chr6:112,429,134-112,575,917	AX-156275999

LAMA4	rs73766949	chr6:112,429,134-112,575,917	AX-156293016
LAMA4	rs9487839	chr6:112,429,134-112,575,917	AX-156276000
LAMA4	rs9400520	chr6:112,429,134-112,575,917	AX-156276001
LAMA4	rs7757339	chr6:112,429,134-112,575,917	AX-156276002
LAMA4	rs7757339	chr6:112,429,134-112,575,917	AX-156293017
LAMA4	rs7757340	chr6:112,429,134-112,575,917	AX-156276003
LAMA4	rs6568720	chr6:112,429,134-112,575,917	AX-11572610
LAMA4	rs2213839	chr6:112,429,134-112,575,917	AX-15195191
LAMA4	rs6568721	chr6:112,429,134-112,575,917	AX-15195192
LAMA4	rs6899453	chr6:112,429,134-112,575,917	AX-15195195
LAMA4	rs1158747	chr6:112,429,134-112,575,917	AX-15195196
LAMA4	rs7752846	chr6:112,429,134-112,575,917	AX-15195198
LAMA4	rs7752759	chr6:112,429,134-112,575,917	AX-15195199
LAMA4	rs4947172	chr6:112,429,134-112,575,917	AX-41820629
LAMA4	rs929124	chr6:112,429,134-112,575,917	AX-156297023
LAMA4	rs79223854	chr6:112,429,134-112,575,917	AX-15195200
LAMA4	rs73766951	chr6:112,429,134-112,575,917	AX-15195204
LAMA4	rs6926862	chr6:112,429,134-112,575,917	AX-41820633
LAMA4	rs6907109	chr6:112,429,134-112,575,917	AX-41820635
LAMA4	rs4526233	chr6:112,429,134-112,575,917	AX-148142798
LAMA4	rs4526233	chr6:112,429,134-112,575,917	AX-92512163
LAMA4	rs4469330	chr6:112,429,134-112,575,917	AX-15195205
LAMA4	rs58039820	chr6:112,429,134-112,575,917	AX-156276004
LAMA4	rs58039820	chr6:112,429,134-112,575,917	AX-156293019
LAMA4	rs3777934	chr6:112,429,134-112,575,917	AX-41820637
LAMA4	rs1894681	chr6:112,429,134-112,575,917	AX-15195207
LAMA4	rs12192658	chr6:112,429,134-112,575,917	AX-11194895
LAMA4	rs17073485	chr6:112,429,134-112,575,917	AX-15195209
LAMA4	rs10872076	chr6:112,429,134-112,575,917	AX-11184088
LAMA4	rs10872076	chr6:112,429,134-112,575,917	AX-122876931
LAMA4	rs73764703	chr6:112,429,134-112,575,917	AX-156276005
LAMA4	rs73764703	chr6:112,429,134-112,575,917	AX-156293020
LAMA4	rs73538594	chr6:112,429,134-112,575,917	AX-15195212
LAMA4	rs17823433	chr6:112,429,134-112,575,917	AX-12505528
LAMA4	rs9481229	chr6:112,429,134-112,575,917	AX-35508069
LAMA4	rs9374309	chr6:112,429,134-112,575,917	AX-15195218
LAMA4	rs6908219	chr6:112,429,134-112,575,917	AX-11593606
LAMA4	rs2301512	chr6:112,429,134-112,575,917	AX-156276006
LAMA4	rs2301512	chr6:112,429,134-112,575,917	AX-156293021
LAMA4	rs2301513	chr6:112,429,134-112,575,917	AX-15195220
LAMA4	rs2301514	chr6:112,429,134-112,575,917	AX-156293022
LAMA4	rs9400521	chr6:112,429,134-112,575,917	AX-35508075
LAMA4	rs74928940	chr6:112,429,134-112,575,917	AX-15195224
LAMA4	rs6938044	chr6:112,429,134-112,575,917	AX-15195225
LAMA4	rs116042905	chr6:112,429,134-112,575,917	AX-156276008
LAMA4	rs116042905	chr6:112,429,134-112,575,917	AX-156293023
LAMA4	rs17073495	chr6:112,429,134-112,575,917	AX-41820649
LAMA4	rs9387061	chr6:112,429,134-112,575,917	AX-85015220
LAMA4	rs9387061	chr6:112,429,134-112,575,917	AX-88783738
LAMA4	---	chr6:112,429,134-112,575,917	AX-91988579
LAMA4	---	chr6:112,429,134-112,575,917	AX-92009974
LAMA4	rs75357508	chr6:112,429,134-112,575,917	AX-15195227
LAMA4	rs56982552	chr6:112,429,134-112,575,917	AX-156276009
LAMA4	rs56982552	chr6:112,429,134-112,575,917	AX-156293024
LAMA4	rs79730073	chr6:112,429,134-112,575,917	AX-35508087

LAMA4	rs114753589	chr6:112,429,134-112,575,917	AX-156282404
LAMA4	rs114753589	chr6:112,429,134-112,575,917	AX-156297024
LAMA4	---	chr6:112,429,134-112,575,917	AX-94355636
LAMA4	rs2072029	chr6:112,429,134-112,575,917	AX-15195232
LAMA4	rs116799545	chr6:112,429,134-112,575,917	AX-156276010
LAMA4	rs116799545	chr6:112,429,134-112,575,917	AX-156293025
LAMA4	rs6901052	chr6:112,429,134-112,575,917	AX-15195234
LAMA4	rs764071	chr6:112,429,134-112,575,917	AX-35508101
LAMA4	rs2269646	chr6:112,429,134-112,575,917	AX-15195235
LAMA4	rs9487840	chr6:112,429,134-112,575,917	AX-148298295
LAMA4	rs9487840	chr6:112,429,134-112,575,917	AX-148678404
LAMA4	rs7745663	chr6:112,429,134-112,575,917	AX-35508105
LAMA4	rs2072021	chr6:112,429,134-112,575,917	AX-12516417
LAMA4	---	chr6:112,429,134-112,575,917	AX-94372387
LAMA4	rs2072022	chr6:112,429,134-112,575,917	AX-11365434
LAMA4	rs2072023	chr6:112,429,134-112,575,917	AX-41820651
LAMA4	rs2237237	chr6:112,429,134-112,575,917	AX-15195241
LAMA4	rs11434202	chr6:112,429,134-112,575,917	AX-122472642
LAMA4	rs2237238	chr6:112,429,134-112,575,917	AX-15195243
LAMA4	rs2237239	chr6:112,429,134-112,575,917	AX-156276011
LAMA4	rs2237240	chr6:112,429,134-112,575,917	AX-120763607
LAMA4	rs3777932	chr6:112,429,134-112,575,917	AX-15195247
LAMA4	rs6930838	chr6:112,429,134-112,575,917	AX-156276012
LAMA4	rs6930838	chr6:112,429,134-112,575,917	AX-156293028
LAMA4	rs9487841	chr6:112,429,134-112,575,917	AX-156276013
LAMA4	rs3777930	chr6:112,429,134-112,575,917	AX-15195249
LAMA4	rs3777929	chr6:112,429,134-112,575,917	AX-12561031
LAMA4	rs3777928	chr6:112,429,134-112,575,917	AX-11479973
LAMA4	rs6568723	chr6:112,429,134-112,575,917	AX-12602978
LAMA4	rs6568724	chr6:112,429,134-112,575,917	AX-15195251
LAMA4	rs200286621	chr6:112,429,134-112,575,917	AX-151126030
LAMA4	rs11757069	chr6:112,429,134-112,575,917	AX-156276014
LAMA4	rs11757069	chr6:112,429,134-112,575,917	AX-156293030
LAMA4	rs9487842	chr6:112,429,134-112,575,917	AX-156276015
LAMA4	rs9487842	chr6:112,429,134-112,575,917	AX-156293031
LAMA4	rs4947174	chr6:112,429,134-112,575,917	AX-156293033
LAMA4	rs7758871	chr6:112,429,134-112,575,917	AX-15195252
LAMA4	rs7738951	chr6:112,429,134-112,575,917	AX-15195254
LAMA4	rs11153345	chr6:112,429,134-112,575,917	AX-41820659
LAMA4	rs62413990	chr6:112,429,134-112,575,917	AX-15195257
LAMA4	rs6912145	chr6:112,429,134-112,575,917	AX-15195260
LAMA4	rs2157546	chr6:112,429,134-112,575,917	AX-15195262
LAMA4	rs3948760	chr6:112,429,134-112,575,917	AX-41820661
LAMA4	rs4947175	chr6:112,429,134-112,575,917	AX-15195263
LAMA4	rs4945898	chr6:112,429,134-112,575,917	AX-15195264
LAMA4	rs2237241	chr6:112,429,134-112,575,917	AX-15195266
LAMA4	rs115769014	chr6:112,429,134-112,575,917	AX-15195269
LAMA4	rs2237242	chr6:112,429,134-112,575,917	AX-41820665
LAMA4	---	chr6:112,429,134-112,575,917	AX-86685336
LAMA4	rs4947176	chr6:112,429,134-112,575,917	AX-15195270
LAMA4	---	chr6:112,429,134-112,575,917	AX-147989553
LAMA4	---	chr6:112,429,134-112,575,917	AX-156276018
LAMA4	rs6935374	chr6:112,429,134-112,575,917	AX-114067398
LAMA4	rs6935374	chr6:112,429,134-112,575,917	AX-156283279
LAMA4	rs6915838	chr6:112,429,134-112,575,917	AX-15195273

LAMA4	rs9487846	chr6:112,429,134-112,575,917	AX-41820667
LAMA4	rs11757455	chr6:112,429,134-112,575,917	AX-11173195
LAMA4	rs4947177	chr6:112,429,134-112,575,917	AX-12586878
LAMA4	rs4947178	chr6:112,429,134-112,575,917	AX-41820671
LAMA4	rs6926485	chr6:112,429,134-112,575,917	AX-41820673
LAMA4	rs6904691	chr6:112,429,134-112,575,917	AX-156276019
LAMA4	rs6904691	chr6:112,429,134-112,575,917	AX-156293034
LAMA4	rs6904212	chr6:112,429,134-112,575,917	AX-123092076
LAMA4	rs2237243	chr6:112,429,134-112,575,917	AX-107717418
LAMA4	rs2237243	chr6:112,429,134-112,575,917	AX-156293035
LAMA4	rs80310126	chr6:112,429,134-112,575,917	AX-15195278
LAMA4	rs74977957	chr6:112,429,134-112,575,917	AX-15195279
LAMA4	rs9374311	chr6:112,429,134-112,575,917	AX-11683973
LAMA4	rs2237244	chr6:112,429,134-112,575,917	AX-11377332
LAMA4	rs9398301	chr6:112,429,134-112,575,917	AX-11685756
LAMA4	rs57700212	chr6:112,429,134-112,575,917	AX-156286143
LAMA4	rs57432793	chr6:112,429,134-112,575,917	AX-156276020
LAMA4	rs60315490	chr6:112,429,134-112,575,917	AX-15195281
LAMA4	rs2237245	chr6:112,429,134-112,575,917	AX-51283308
LAMA4	rs11413588	chr6:112,429,134-112,575,917	AX-11156669
LAMA4	rs201712365	chr6:112,429,134-112,575,917	AX-151365842
LAMA4	rs201712365	chr6:112,429,134-112,575,917	AX-156297727
LAMA4	rs10585917	chr6:112,429,134-112,575,917	AX-151356844
LAMA4	rs138554247	chr6:112,429,134-112,575,917	AX-151355712
LAMA4	rs138554247	chr6:112,429,134-112,575,917	AX-15195283
LAMA4	rs6939619	chr6:112,429,134-112,575,917	AX-41820681
LAMA4	rs6916501	chr6:112,429,134-112,575,917	AX-156276021
LAMA4	rs6916501	chr6:112,429,134-112,575,917	AX-156293036
LAMA4	rs74760631	chr6:112,429,134-112,575,917	AX-15195285
LAMA4	rs6917160	chr6:112,429,134-112,575,917	AX-41820685
LAMA4	rs6916694	chr6:112,429,134-112,575,917	AX-41820687
LAMA4	rs3777925	chr6:112,429,134-112,575,917	AX-11479972
LAMA4	rs6939808	chr6:112,429,134-112,575,917	AX-41820689
LAMA4	rs59596549	chr6:112,429,134-112,575,917	AX-15195286
LAMA4	rs7754329	chr6:112,429,134-112,575,917	AX-11643287
LAMA4	rs59690635	chr6:112,429,134-112,575,917	AX-15195287
LAMA4	rs67517586	chr6:112,429,134-112,575,917	AX-151355663
LAMA4	rs67517586	chr6:112,429,134-112,575,917	AX-156298640
LAMA4	rs140099023	chr6:112,429,134-112,575,917	AX-151355671
LAMA4	rs140099023	chr6:112,429,134-112,575,917	AX-156297984
LAMA4	rs77748844	chr6:112,429,134-112,575,917	AX-35508141
LAMA4	rs73542519	chr6:112,429,134-112,575,917	AX-15195291
LAMA4	rs140805629	chr6:112,429,134-112,575,917	AX-151459779
LAMA4	rs73542521	chr6:112,429,134-112,575,917	AX-15195292
LAMA4	rs73542523	chr6:112,429,134-112,575,917	AX-15195293
LAMA4	rs73542524	chr6:112,429,134-112,575,917	AX-15195294
LAMA4	rs73542527	chr6:112,429,134-112,575,917	AX-15195295
LAMA4	rs73542530	chr6:112,429,134-112,575,917	AX-15195297
LAMA4	rs17073562	chr6:112,429,134-112,575,917	AX-15195298
LAMA4	rs73542534	chr6:112,429,134-112,575,917	AX-15195299
LAMA4	rs73542539	chr6:112,429,134-112,575,917	AX-156276022
LAMA4	rs73542539	chr6:112,429,134-112,575,917	AX-156293037
LAMA4	rs73542543	chr6:112,429,134-112,575,917	AX-15195300
LAMA4	rs73542545	chr6:112,429,134-112,575,917	AX-35508147
LAMA4	rs17073563	chr6:112,429,134-112,575,917	AX-41820691

LAMA4	rs12190908	chr6:112,429,134-112,575,917	AX-11194779
LAMA4	rs77759681	chr6:112,429,134-112,575,917	AX-15195301
LAMA4	rs77329639	chr6:112,429,134-112,575,917	AX-15195302
LAMA4	rs73542549	chr6:112,429,134-112,575,917	AX-15195303
LAMA4	rs138176093	chr6:112,429,134-112,575,917	AX-83492439
LAMA4	rs17073565	chr6:112,429,134-112,575,917	AX-41820693
LAMA4	rs2239849	chr6:112,429,134-112,575,917	AX-12522319
LAMA4	rs6909615	chr6:112,429,134-112,575,917	AX-11593702
LAMA4	rs73542553	chr6:112,429,134-112,575,917	AX-15195304
LAMA4	rs17073566	chr6:112,429,134-112,575,917	AX-41820695
LAMA4	rs75956673	chr6:112,429,134-112,575,917	AX-15195305
LAMA4	rs73542558	chr6:112,429,134-112,575,917	AX-15195306
LAMA4	rs73542561	chr6:112,429,134-112,575,917	AX-15195307
LAMA4	rs9487847	chr6:112,429,134-112,575,917	AX-15195308
LAMA4	rs17073570	chr6:112,429,134-112,575,917	AX-41820697
LAMA4	rs147329384	chr6:112,429,134-112,575,917	AX-151285642
LAMA4	rs17073572	chr6:112,429,134-112,575,917	AX-11303612
LAMA4	rs2237246	chr6:112,429,134-112,575,917	AX-11377333
LAMA4	rs2237247	chr6:112,429,134-112,575,917	AX-12522161
LAMA4	rs17073574	chr6:112,429,134-112,575,917	AX-15195311
LAMA4	rs6921607	chr6:112,429,134-112,575,917	AX-12614387
LAMA4	rs2282853	chr6:112,429,134-112,575,917	AX-15195314
LAMA4	rs113210444	chr6:112,429,134-112,575,917	AX-148825784
LAMA4	rs113210444	chr6:112,429,134-112,575,917	AX-156283280
LAMA4	rs9487850	chr6:112,429,134-112,575,917	AX-148176049
LAMA4	rs9487850	chr6:112,429,134-112,575,917	AX-156276023
LAMA4	rs9487851	chr6:112,429,134-112,575,917	AX-148105273
LAMA4	rs9487851	chr6:112,429,134-112,575,917	AX-156276024
LAMA4	rs73764707	chr6:112,429,134-112,575,917	AX-15195320
LAMA4	rs17073584	chr6:112,429,134-112,575,917	AX-41820703
LAMA4	rs60571526	chr6:112,429,134-112,575,917	AX-15195322
LAMA4	rs11153346	chr6:112,429,134-112,575,917	AX-107738859
LAMA4	rs11153346	chr6:112,429,134-112,575,917	AX-156297026
LAMA4	rs4945899	chr6:112,429,134-112,575,917	AX-11537982
LAMA4	rs73764709	chr6:112,429,134-112,575,917	AX-15195323
LAMA4	rs34072237	chr6:112,429,134-112,575,917	AX-11440686
LAMA4	rs12198087	chr6:112,429,134-112,575,917	AX-11195297
LAMA4	rs73544339	chr6:112,429,134-112,575,917	AX-156293038
LAMA4	rs73544339	chr6:112,429,134-112,575,917	AX-84922494
LAMA4	rs28360619	chr6:112,429,134-112,575,917	AX-11414670
LAMA4	rs75992359	chr6:112,429,134-112,575,917	AX-15195328
LAMA4	rs78278111	chr6:112,429,134-112,575,917	AX-156276025
LAMA4	rs78278111	chr6:112,429,134-112,575,917	AX-156293039
LAMA4	rs9374312	chr6:112,429,134-112,575,917	AX-11683974
LAMA4	rs11435641	chr6:112,429,134-112,575,917	AX-121888412
LAMA4	rs11435641	chr6:112,429,134-112,575,917	AX-156293040
LAMA4	rs2213840	chr6:112,429,134-112,575,917	AX-11375566
LAMA4	rs73764712	chr6:112,429,134-112,575,917	AX-15195332
LAMA4	rs73764713	chr6:112,429,134-112,575,917	AX-15195335
LAMA4	rs2068770	chr6:112,429,134-112,575,917	AX-15195336
LAMA4	rs35349917	chr6:112,429,134-112,575,917	AX-41820711
LAMA4	rs7763270	chr6:112,429,134-112,575,917	AX-41820713
LAMA4	rs9487853	chr6:112,429,134-112,575,917	AX-15195338
LAMA4	rs2237248	chr6:112,429,134-112,575,917	AX-12522162
LAMA4	rs2237249	chr6:112,429,134-112,575,917	AX-15195339

LAMA4	rs73764715	chr6:112,429,134-112,575,917	AX-15195340
LAMA4	rs140359426	chr6:112,429,134-112,575,917	AX-122403091
LAMA4	rs2345808	chr6:112,429,134-112,575,917	AX-15195346
LAMA4	rs2157547	chr6:112,429,134-112,575,917	AX-15195348
LAMA4	rs73764716	chr6:112,429,134-112,575,917	AX-15195349
LAMA4	rs12111523	chr6:112,429,134-112,575,917	AX-15195352
LAMA4	rs7450379	chr6:112,429,134-112,575,917	AX-11625117
LAMA4	rs58543421	chr6:112,429,134-112,575,917	AX-15195355
LAMA4	rs2345809	chr6:112,429,134-112,575,917	AX-35508205
LAMA4	rs73764717	chr6:112,429,134-112,575,917	AX-15195357
LAMA4	rs2157548	chr6:112,429,134-112,575,917	AX-12519139
LAMA4	rs2157548	chr6:112,429,134-112,575,917	AX-156276026
LAMA4	rs75022233	chr6:112,429,134-112,575,917	AX-15195358
LAMA4	rs9387063	chr6:112,429,134-112,575,917	AX-11684911
LAMA4	rs77652041	chr6:112,429,134-112,575,917	AX-15195360
LAMA4	rs6900576	chr6:112,429,134-112,575,917	AX-148222290
LAMA4	rs6900576	chr6:112,429,134-112,575,917	AX-148391767
LAMA4	rs140424860	chr6:112,429,134-112,575,917	AX-122132569
LAMA4	rs140424860	chr6:112,429,134-112,575,917	AX-156283281
LAMA4	rs150011219	chr6:112,429,134-112,575,917	AX-148924087
LAMA4	rs150011219	chr6:112,429,134-112,575,917	AX-149056379
LAMA4	rs12661406	chr6:112,429,134-112,575,917	AX-41820735
LAMA4	rs11153347	chr6:112,429,134-112,575,917	AX-41820737
LAMA4	rs1006497	chr6:112,429,134-112,575,917	AX-15195362
LAMA4	rs2345810	chr6:112,429,134-112,575,917	AX-15195367
LAMA4	rs145415561	chr6:112,429,134-112,575,917	AX-107726849
LAMA4	rs145415561	chr6:112,429,134-112,575,917	AX-156297879
LAMA4	rs4947179	chr6:112,429,134-112,575,917	AX-11538072
LAMA4	rs9320400	chr6:112,429,134-112,575,917	AX-15195369
LAMA4	rs9487857	chr6:112,429,134-112,575,917	AX-15195372
LAMA4	rs9487858	chr6:112,429,134-112,575,917	AX-156276027
LAMA4	rs9487858	chr6:112,429,134-112,575,917	AX-156293041
LAMA4	rs73764718	chr6:112,429,134-112,575,917	AX-15195373
LAMA4	rs73764719	chr6:112,429,134-112,575,917	AX-15195375
LAMA4	rs78532275	chr6:112,429,134-112,575,917	AX-35508243
LAMA4	rs75558316	chr6:112,429,134-112,575,917	AX-15195381
LAMA4	rs6568726	chr6:112,429,134-112,575,917	AX-41820747
LAMA4	rs80110510	chr6:112,429,134-112,575,917	AX-15195382
LAMA4	rs6906525	chr6:112,429,134-112,575,917	AX-156276028
LAMA4	rs6906525	chr6:112,429,134-112,575,917	AX-156293042
LAMA4	rs58986775	chr6:112,429,134-112,575,917	AX-15195384
LAMA4	rs73764720	chr6:112,429,134-112,575,917	AX-35508247
LAMA4	rs60955874	chr6:112,429,134-112,575,917	AX-15195385
LAMA4	rs9481237	chr6:112,429,134-112,575,917	AX-41820753
LAMA4	rs73764721	chr6:112,429,134-112,575,917	AX-15195388
LAMA4	rs7764836	chr6:112,429,134-112,575,917	AX-15195391
LAMA4	rs28360620	chr6:112,429,134-112,575,917	AX-15195396
LAMA4	rs9398302	chr6:112,429,134-112,575,917	AX-11685757
LAMA4	rs73764722	chr6:112,429,134-112,575,917	AX-15195398
LAMA4	rs12206703	chr6:112,429,134-112,575,917	AX-41820761
LAMA4	rs73764723	chr6:112,429,134-112,575,917	AX-15195400
LAMA4	rs12206921	chr6:112,429,134-112,575,917	AX-12430426
LAMA4	rs7775097	chr6:112,429,134-112,575,917	AX-41820763
LAMA4	rs4947180	chr6:112,429,134-112,575,917	AX-120394958
LAMA4	rs1573756	chr6:112,429,134-112,575,917	AX-15195402

LAMA4	rs9487860	chr6:112,429,134-112,575,917	AX-156276030
LAMA4	rs9487860	chr6:112,429,134-112,575,917	AX-156293043
LAMA4	rs9487861	chr6:112,429,134-112,575,917	AX-11689653
LAMA4	rs2072024	chr6:112,429,134-112,575,917	AX-11365435
LAMA4	rs9481239	chr6:112,429,134-112,575,917	AX-153825375
LAMA4	rs9481239	chr6:112,429,134-112,575,917	AX-156293044
LAMA4	rs9481240	chr6:112,429,134-112,575,917	AX-122977004
LAMA4	rs73764724	chr6:112,429,134-112,575,917	AX-35508257
LAMA4	rs9487862	chr6:112,429,134-112,575,917	AX-156276031
LAMA4	rs9487862	chr6:112,429,134-112,575,917	AX-96692701
LAMA4	rs74688603	chr6:112,429,134-112,575,917	AX-148650686
LAMA4	rs74688603	chr6:112,429,134-112,575,917	AX-156283282
LAMA4	rs113792282	chr6:112,429,134-112,575,917	AX-148422303
LAMA4	rs113792282	chr6:112,429,134-112,575,917	AX-156283283
LAMA4	rs62414016	chr6:112,429,134-112,575,917	AX-35508259
LAMA4	rs2051651	chr6:112,429,134-112,575,917	AX-120209236
LAMA4	rs2051651	chr6:112,429,134-112,575,917	AX-156293045
LAMA4	rs35006512	chr6:112,429,134-112,575,917	AX-156276032
LAMA4	rs17073600	chr6:112,429,134-112,575,917	AX-156276033
LAMA4	rs17073600	chr6:112,429,134-112,575,917	AX-156293046
LAMA4	rs60697308	chr6:112,429,134-112,575,917	AX-148103414
LAMA4	rs60697308	chr6:112,429,134-112,575,917	AX-148497866
LAMA4	---	chr6:112,429,134-112,575,917	AX-148496073
LAMA4	---	chr6:112,429,134-112,575,917	AX-156283284
LAMA4	rs6926573	chr6:112,429,134-112,575,917	AX-41820773
LAMA4	rs6909113	chr6:112,429,134-112,575,917	AX-148317403
LAMA4	rs6909113	chr6:112,429,134-112,575,917	AX-149519030
LAMA4	rs62414019	chr6:112,429,134-112,575,917	AX-156283285
LAMA4	rs62414019	chr6:112,429,134-112,575,917	AX-88319044
LAMA4	rs62414020	chr6:112,429,134-112,575,917	AX-151258456
LAMA4	rs62414020	chr6:112,429,134-112,575,917	AX-156276034
LAMA4	rs5879143	chr6:112,429,134-112,575,917	AX-120745698
LAMA4	rs5879143	chr6:112,429,134-112,575,917	AX-156293048
LAMA4	rs58010224	chr6:112,429,134-112,575,917	AX-156276035
LAMA4	rs58010224	chr6:112,429,134-112,575,917	AX-156293049
LAMA4	rs9320402	chr6:112,429,134-112,575,917	AX-15195415
LAMA4	rs6921858	chr6:112,429,134-112,575,917	AX-15195417
LAMA4	rs62414021	chr6:112,429,134-112,575,917	AX-35508269
LAMA4	rs1894682	chr6:112,429,134-112,575,917	AX-12509514
LAMA4	rs6932642	chr6:112,429,134-112,575,917	AX-156276036
LAMA4	rs6932642	chr6:112,429,134-112,575,917	AX-156293050
LAMA4	rs147318270	chr6:112,429,134-112,575,917	AX-151459910
LAMA4	rs147318270	chr6:112,429,134-112,575,917	AX-156297874
LAMA4	rs11153348	chr6:112,429,134-112,575,917	AX-11147440
LAMA4	rs9487863	chr6:112,429,134-112,575,917	AX-122219224
LAMA4	rs9487863	chr6:112,429,134-112,575,917	AX-122977005
LAMA4	rs11759718	chr6:112,429,134-112,575,917	AX-35508281
LAMA4	rs17757451	chr6:112,429,134-112,575,917	AX-156276037
LAMA4	rs17757451	chr6:112,429,134-112,575,917	AX-156293051
LAMA4	rs3208829	chr6:112,429,134-112,575,917	AX-15195421
LAMA4	rs72952403	chr6:112,429,134-112,575,917	AX-35508287
LAMA4	rs7766787	chr6:112,429,134-112,575,917	AX-11644144
LAMP3	rs116723189	chr3:182,840,003-182,880,667	AX-34197187
LAMP3	rs3821517	chr3:182,840,003-182,880,667	AX-14225123
LAMP3	rs491841	chr3:182,840,003-182,880,667	AX-14225124

LAMP3	rs3821520	chr3:182,840,003-182,880,667	AX-34197209
LAMP3	rs79447920	chr3:182,840,003-182,880,667	AX-14225130
LAMP3	rs515818	chr3:182,840,003-182,880,667	AX-12588829
LAMP3	rs150432783	chr3:182,840,003-182,880,667	AX-94353877
LAMP3	rs482912	chr3:182,840,003-182,880,667	AX-11529303
LAMP3	rs522235	chr3:182,840,003-182,880,667	AX-11542884
LAMP3	---	chr3:182,840,003-182,880,667	AX-86611420
LAMP3	rs831256	chr3:182,840,003-182,880,667	AX-41179981
LAMP3	rs675924	chr3:182,840,003-182,880,667	AX-41179983
LAMP3	rs75470872	chr3:182,840,003-182,880,667	AX-34197235
LAMP3	rs955861	chr3:182,840,003-182,880,667	AX-11694561
LAMP3	rs7639990	chr3:182,840,003-182,880,667	AX-11635517
LAMP3	rs79155994	chr3:182,840,003-182,880,667	AX-14225161
LAMP3	rs76730564	chr3:182,840,003-182,880,667	AX-14225163
LAMP3	rs77611354	chr3:182,840,003-182,880,667	AX-14225164
LAMP3	rs10513791	chr3:182,840,003-182,880,667	AX-11114590
LAMP3	rs74965417	chr3:182,840,003-182,880,667	AX-34197249
LAMP3	rs75413779	chr3:182,840,003-182,880,667	AX-34197253
LAMP3	rs7648312	chr3:182,840,003-182,880,667	AX-14225171
LAMP3	rs41265435	chr3:182,840,003-182,880,667	AX-41180005
LAMP3	rs16833752	chr3:182,840,003-182,880,667	AX-41180009
LAMP3	rs115281513	chr3:182,840,003-182,880,667	AX-82974566
LAMP3	rs514636	chr3:182,840,003-182,880,667	AX-11542418
LAMP3	rs79275625	chr3:182,840,003-182,880,667	AX-34197279
LAMP3	rs114318380	chr3:182,840,003-182,880,667	AX-34197289
LAMP3	rs6414500	chr3:182,840,003-182,880,667	AX-11562816
LAMP3	rs500288	chr3:182,840,003-182,880,667	AX-11541085
LAMP3	rs653316	chr3:182,840,003-182,880,667	AX-14225181
LAMP3	rs2280563	chr3:182,840,003-182,880,667	AX-12523975
LAMP3	rs580116	chr3:182,840,003-182,880,667	AX-11547494
LAMP3	rs12493054	chr3:182,840,003-182,880,667	AX-11210034
LAMP3	rs76634661	chr3:182,840,003-182,880,667	AX-34197309
LAMP3	rs657261	chr3:182,840,003-182,880,667	AX-14225193
LAMP3	rs71314285	chr3:182,840,003-182,880,667	AX-34197319
LAMP3	rs6443856	chr3:182,840,003-182,880,667	AX-14225200
LDB3	rs34106786	chr10:88,426,551-88,495,824	AX-92691399
LDB3	rs34499525	chr10:88,426,551-88,495,824	AX-29753111
LDB3	rs2675693	chr10:88,426,551-88,495,824	AX-38900235
LDB3	rs34346910	chr10:88,426,551-88,495,824	AX-29753113
LDB3	rs34959905	chr10:88,426,551-88,495,824	AX-156294799
LDB3	rs4256897	chr10:88,426,551-88,495,824	AX-156278571
LDB3	rs4256897	chr10:88,426,551-88,495,824	AX-156294800
LDB3	rs2675692	chr10:88,426,551-88,495,824	AX-156279440
LDB3	rs2675692	chr10:88,426,551-88,495,824	AX-156295137
LDB3	rs2803555	chr10:88,426,551-88,495,824	AX-38900241
LDB3	rs11812601	chr10:88,426,551-88,495,824	AX-92611981
LDB3	rs2803556	chr10:88,426,551-88,495,824	AX-156278572
LDB3	rs2803556	chr10:88,426,551-88,495,824	AX-156294801
LDB3	rs2803558	chr10:88,426,551-88,495,824	AX-156278573
LDB3	rs2803558	chr10:88,426,551-88,495,824	AX-156294802
LDB3	rs12259201	chr10:88,426,551-88,495,824	AX-29753129
LDB3	rs3740342	chr10:88,426,551-88,495,824	AX-38900243
LDB3	---	chr10:88,426,551-88,495,824	AX-92729928
LDB3	rs200764076	chr10:88,426,551-88,495,824	AX-156298264
LDB3	rs200764076	chr10:88,426,551-88,495,824	AX-29753135

LDB3	rs10887639	chr10:88,426,551-88,495,824	AX-156278574
LDB3	rs10887639	chr10:88,426,551-88,495,824	AX-156294803
LDB3	rs10887640	chr10:88,426,551-88,495,824	AX-29753139
LDB3	---	chr10:88,426,551-88,495,824	AX-151160014
LDB3	rs4492716	chr10:88,426,551-88,495,824	AX-29753147
LDB3	rs12570315	chr10:88,426,551-88,495,824	AX-156278575
LDB3	rs2354363	chr10:88,426,551-88,495,824	AX-38900249
LDB3	rs12569813	chr10:88,426,551-88,495,824	AX-156278576
LDB3	rs12569813	chr10:88,426,551-88,495,824	AX-156294805
LDB3	rs2244769	chr10:88,426,551-88,495,824	AX-38900253
LDB3	rs2803560	chr10:88,426,551-88,495,824	AX-38900255
LDB3	rs11202117	chr10:88,426,551-88,495,824	AX-84609964
LDB3	rs11202117	chr10:88,426,551-88,495,824	AX-84621581
LDB3	rs11202118	chr10:88,426,551-88,495,824	AX-92583436
LDB3	rs11202118	chr10:88,426,551-88,495,824	AX-92794459
LDB3	rs11202119	chr10:88,426,551-88,495,824	AX-92616480
LDB3	rs34677208	chr10:88,426,551-88,495,824	AX-92457859
LDB3	rs34677208	chr10:88,426,551-88,495,824	AX-92668882
LDB3	rs11202120	chr10:88,426,551-88,495,824	AX-156278579
LDB3	rs10788522	chr10:88,426,551-88,495,824	AX-156278580
LDB3	rs10788522	chr10:88,426,551-88,495,824	AX-156294807
LDB3	rs11202121	chr10:88,426,551-88,495,824	AX-38900259
LDB3	rs67431774	chr10:88,426,551-88,495,824	AX-29753167
LDB3	rs2354362	chr10:88,426,551-88,495,824	AX-29753173
LDB3	rs2354361	chr10:88,426,551-88,495,824	AX-156294808
LDB3	rs2354360	chr10:88,426,551-88,495,824	AX-92581825
LDB3	rs2354360	chr10:88,426,551-88,495,824	AX-92792848
LDB3	rs2354359	chr10:88,426,551-88,495,824	AX-156269247
LDB3	rs2354359	chr10:88,426,551-88,495,824	AX-156288764
LDB3	rs2354358	chr10:88,426,551-88,495,824	AX-156288765
LDB3	rs2354357	chr10:88,426,551-88,495,824	AX-29753179
LDB3	rs7895178	chr10:88,426,551-88,495,824	AX-92455633
LDB3	rs2247689	chr10:88,426,551-88,495,824	AX-38900265
LDB3	rs7894381	chr10:88,426,551-88,495,824	AX-29753183
LDB3	rs1806471	chr10:88,426,551-88,495,824	AX-156279441
LDB3	rs1806471	chr10:88,426,551-88,495,824	AX-156295138
LDB3	rs2803561	chr10:88,426,551-88,495,824	AX-156279442
LDB3	rs2803561	chr10:88,426,551-88,495,824	AX-156295139
LDB3	---	chr10:88,426,551-88,495,824	AX-38900267
LDB3	rs2803563	chr10:88,426,551-88,495,824	AX-156279443
LDB3	rs2803563	chr10:88,426,551-88,495,824	AX-156295140
LDB3	rs2803564	chr10:88,426,551-88,495,824	AX-156295141
LDB3	rs2675700	chr10:88,426,551-88,495,824	AX-156279445
LDB3	rs10887642	chr10:88,426,551-88,495,824	AX-29753199
LDB3	rs2354356	chr10:88,426,551-88,495,824	AX-156278582
LDB3	rs2354356	chr10:88,426,551-88,495,824	AX-156294809
LDB3	rs10788523	chr10:88,426,551-88,495,824	AX-29753203
LDB3	rs11819261	chr10:88,426,551-88,495,824	AX-29753205
LDB3	rs12771857	chr10:88,426,551-88,495,824	AX-156278583
LDB3	rs12771857	chr10:88,426,551-88,495,824	AX-156294810
LDB3	rs11592193	chr10:88,426,551-88,495,824	AX-92537192
LDB3	rs11592193	chr10:88,426,551-88,495,824	AX-92748215
LDB3	rs7074015	chr10:88,426,551-88,495,824	AX-29753213
LDB3	rs7475311	chr10:88,426,551-88,495,824	AX-29753215
LDB3	rs114576982	chr10:88,426,551-88,495,824	AX-156278487

LDB3	rs114576982	chr10:88,426,551-88,495,824	AX-95862165
LDB3	---	chr10:88,426,551-88,495,824	AX-151227712
LDB3	---	chr10:88,426,551-88,495,824	AX-156297706
LDB3	rs12240576	chr10:88,426,551-88,495,824	AX-156278585
LDB3	rs12268509	chr10:88,426,551-88,495,824	AX-156278586
LDB3	rs12268509	chr10:88,426,551-88,495,824	AX-156294813
LDB3	rs12240646	chr10:88,426,551-88,495,824	AX-29753223
LDB3	rs10887643	chr10:88,426,551-88,495,824	AX-29753225
LDB3	rs61857111	chr10:88,426,551-88,495,824	AX-29753227
LDB3	rs61857112	chr10:88,426,551-88,495,824	AX-156278587
LDB3	rs61857113	chr10:88,426,551-88,495,824	AX-156294815
LDB3	rs10788524	chr10:88,426,551-88,495,824	AX-38900271
LDB3	rs2803566	chr10:88,426,551-88,495,824	AX-38900273
LDB3	rs12241060	chr10:88,426,551-88,495,824	AX-156278589
LDB3	rs12241060	chr10:88,426,551-88,495,824	AX-156294816
LDB3	rs10887644	chr10:88,426,551-88,495,824	AX-156278590
LDB3	rs10887645	chr10:88,426,551-88,495,824	AX-29753233
LDB3	rs2803567	chr10:88,426,551-88,495,824	AX-38900275
LDB3	rs10788525	chr10:88,426,551-88,495,824	AX-38900277
LDB3	rs2803568	chr10:88,426,551-88,495,824	AX-156278591
LDB3	rs2803568	chr10:88,426,551-88,495,824	AX-156294818
LDB3	rs10887646	chr10:88,426,551-88,495,824	AX-156294819
LDB3	rs4462249	chr10:88,426,551-88,495,824	AX-38900285
LDB3	rs4934242	chr10:88,426,551-88,495,824	AX-148615102
LDB3	rs12254069	chr10:88,426,551-88,495,824	AX-38900293
LDB3	---	chr10:88,426,551-88,495,824	AX-38900295
LDB3	rs45591834	chr10:88,426,551-88,495,824	AX-83363529
LDB3	rs3740343	chr10:88,426,551-88,495,824	AX-38900297
LDB3	rs3740344	chr10:88,426,551-88,495,824	AX-29753245
LDB3	rs60835566	chr10:88,426,551-88,495,824	AX-29753247
LDB3	rs45617137	chr10:88,426,551-88,495,824	AX-29753253
LDB3	rs2803570	chr10:88,426,551-88,495,824	AX-38900299
LDB3	rs111775171	chr10:88,426,551-88,495,824	AX-151271668
LDB3	rs111775171	chr10:88,426,551-88,495,824	AX-156285871
LDB3	rs2248537	chr10:88,426,551-88,495,824	AX-107874302
LDB3	rs2248538	chr10:88,426,551-88,495,824	AX-156278593
LDB3	rs2248538	chr10:88,426,551-88,495,824	AX-156294820
LDB3	rs2248643	chr10:88,426,551-88,495,824	AX-156278594
LDB3	rs2675686	chr10:88,426,551-88,495,824	AX-156278595
LDB3	rs45487699	chr10:88,426,551-88,495,824	AX-83414002
LDB3	---	chr10:88,426,551-88,495,824	AX-83190212
LDB3	rs121908337	chr10:88,426,551-88,495,824	AX-90069129
LDB3	rs55815121	chr10:88,426,551-88,495,824	AX-29753275
LDB3	rs56165849	chr10:88,426,551-88,495,824	AX-29753277
LDB3	rs4933404	chr10:88,426,551-88,495,824	AX-29753279
LDB3	rs10749541	chr10:88,426,551-88,495,824	AX-29753281
LDB3	rs58051699	chr10:88,426,551-88,495,824	AX-29753283
LDB3	rs4934243	chr10:88,426,551-88,495,824	AX-38900303
LDB3	rs4934244	chr10:88,426,551-88,495,824	AX-29753291
LDB3	rs11202124	chr10:88,426,551-88,495,824	AX-38900305
LDB3	rs12244569	chr10:88,426,551-88,495,824	AX-114212614
LDB3	rs12244569	chr10:88,426,551-88,495,824	AX-156278596
LDB3	rs12244574	chr10:88,426,551-88,495,824	AX-113731303
LDB3	rs12244574	chr10:88,426,551-88,495,824	AX-156294822
LDB3	rs12251540	chr10:88,426,551-88,495,824	AX-38900311

LDB3	rs11599515	chr10:88,426,551-88,495,824	AX-29753295
LDB3	rs10887647	chr10:88,426,551-88,495,824	AX-29753297
LDB3	rs11594919	chr10:88,426,551-88,495,824	AX-29753299
LDB3	rs35605324	chr10:88,426,551-88,495,824	AX-29753301
LDB3	rs4934245	chr10:88,426,551-88,495,824	AX-38900313
LDB3	rs4934246	chr10:88,426,551-88,495,824	AX-29753305
LDB3	rs10887648	chr10:88,426,551-88,495,824	AX-156278597
LDB3	rs10887648	chr10:88,426,551-88,495,824	AX-156294823
LDB3	rs4934247	chr10:88,426,551-88,495,824	AX-114212615
LDB3	rs4934247	chr10:88,426,551-88,495,824	AX-156278598
LDB3	rs4934248	chr10:88,426,551-88,495,824	AX-113731306
LDB3	rs4934248	chr10:88,426,551-88,495,824	AX-156278599
LDB3	rs76076271	chr10:88,426,551-88,495,824	AX-156278600
LDB3	rs76076271	chr10:88,426,551-88,495,824	AX-156294824
LDB3	rs4934249	chr10:88,426,551-88,495,824	AX-156278601
LDB3	rs4934249	chr10:88,426,551-88,495,824	AX-156294825
LDB3	rs4934250	chr10:88,426,551-88,495,824	AX-113247830
LDB3	rs4934250	chr10:88,426,551-88,495,824	AX-156278602
LDB3	rs79039198	chr10:88,426,551-88,495,824	AX-113731307
LDB3	rs79102492	chr10:88,426,551-88,495,824	AX-114212616
LDB3	rs11593608	chr10:88,426,551-88,495,824	AX-38900315
LDB3	rs7893160	chr10:88,426,551-88,495,824	AX-156279446
LDB3	rs7893160	chr10:88,426,551-88,495,824	AX-156295143
LDB3	rs7923558	chr10:88,426,551-88,495,824	AX-29753323
LDB3	rs35422936	chr10:88,426,551-88,495,824	AX-29753327
LDB3	rs3740345	chr10:88,426,551-88,495,824	AX-38900319
LDB3	rs3802662	chr10:88,426,551-88,495,824	AX-156278605
LDB3	rs3802662	chr10:88,426,551-88,495,824	AX-156294828
LDB3	rs3802663	chr10:88,426,551-88,495,824	AX-38900323
LDB3	rs111318302	chr10:88,426,551-88,495,824	AX-29753337
LDB3	rs11202126	chr10:88,426,551-88,495,824	AX-29753339
LDB3	rs1986382	chr10:88,426,551-88,495,824	AX-38900327
LDB3	rs1986383	chr10:88,426,551-88,495,824	AX-148073586
LDB3	rs1986383	chr10:88,426,551-88,495,824	AX-156278606
LDB3	rs113972163	chr10:88,426,551-88,495,824	AX-120525321
LDB3	rs113972163	chr10:88,426,551-88,495,824	AX-148654791
LDB3	rs1578895	chr10:88,426,551-88,495,824	AX-156278607
LDB3	rs1578895	chr10:88,426,551-88,495,824	AX-156294829
LDB3	rs73344172	chr10:88,426,551-88,495,824	AX-156278608
LDB3	---	chr10:88,426,551-88,495,824	AX-156278609
LDB3	---	chr10:88,426,551-88,495,824	AX-156294830
LDB3	rs121908338	chr10:88,426,551-88,495,824	AX-83137332
LDB3	rs76615432	chr10:88,426,551-88,495,824	AX-29753347
LDB3	rs111941601	chr10:88,426,551-88,495,824	AX-156278610
LDB3	rs111941601	chr10:88,426,551-88,495,824	AX-156294831
LDB3	rs79106435	chr10:88,426,551-88,495,824	AX-29753355
LDB3	rs144097163	chr10:88,426,551-88,495,824	AX-120855529
LDB3	rs144097163	chr10:88,426,551-88,495,824	AX-121508691
LDB3	rs79025854	chr10:88,426,551-88,495,824	AX-29753361
LDB3	rs6586023	chr10:88,426,551-88,495,824	AX-38900333
LDB3	rs200829736	chr10:88,426,551-88,495,824	AX-151141337
LDB3	rs200829736	chr10:88,426,551-88,495,824	AX-156298190
LDB3	rs11331035	chr10:88,426,551-88,495,824	AX-107827328
LDB3	rs11331035	chr10:88,426,551-88,495,824	AX-156278611
LDB3	rs4934251	chr10:88,426,551-88,495,824	AX-38900337

LDB3	rs12251655	chr10:88,426,551-88,495,824	AX-38900339
LDB3	rs72848117	chr10:88,426,551-88,495,824	AX-29753373
LDB3	rs78559934	chr10:88,426,551-88,495,824	AX-29753375
LDB3	rs11312118	chr10:88,426,551-88,495,824	AX-107885510
LDB3	rs34423165	chr10:88,426,551-88,495,824	AX-83591867
LDB3	rs73344181	chr10:88,426,551-88,495,824	AX-29753379
LDB3	rs2675691	chr10:88,426,551-88,495,824	AX-38900343
LDB3	rs7069570	chr10:88,426,551-88,495,824	AX-156278613
LDB3	rs7069570	chr10:88,426,551-88,495,824	AX-156294832
LDB3	rs117434536	chr10:88,426,551-88,495,824	AX-29753389
LDB3	rs7086308	chr10:88,426,551-88,495,824	AX-156279447
LDB3	rs7086308	chr10:88,426,551-88,495,824	AX-156295144
LDB3	rs7085298	chr10:88,426,551-88,495,824	AX-156279448
LDB3	rs7085298	chr10:88,426,551-88,495,824	AX-156295145
LDB3	---	chr10:88,426,551-88,495,824	AX-121378617
LDB3	rs2803572	chr10:88,426,551-88,495,824	AX-38900351
LDB3	rs4933405	chr10:88,426,551-88,495,824	AX-38900355
LDB3	rs137917288	chr10:88,426,551-88,495,824	AX-151272077
LDB3	rs4933406	chr10:88,426,551-88,495,824	AX-29753399
LDB3	rs142137148	chr10:88,426,551-88,495,824	AX-148628830
LDB3	rs142137148	chr10:88,426,551-88,495,824	AX-156283631
LDB3	rs12414832	chr10:88,426,551-88,495,824	AX-96773365
LDB3	rs7088948	chr10:88,426,551-88,495,824	AX-29753405
LDB3	rs4933407	chr10:88,426,551-88,495,824	AX-156294833
LDB3	rs80257700	chr10:88,426,551-88,495,824	AX-29753407
LDB3	rs17106945	chr10:88,426,551-88,495,824	AX-38900357
LDB3	rs121908335	chr10:88,426,551-88,495,824	AX-90069128
LDB3	rs41306868	chr10:88,426,551-88,495,824	AX-29753413
LDB3	rs75224783	chr10:88,426,551-88,495,824	AX-29753417
LDB3	rs78137867	chr10:88,426,551-88,495,824	AX-29753427
LDB3	rs112644179	chr10:88,426,551-88,495,824	AX-29753429
LDB3	rs7908829	chr10:88,426,551-88,495,824	AX-29753431
LDB3	rs10887650	chr10:88,426,551-88,495,824	AX-75179613
LDB3	rs10887650	chr10:88,426,551-88,495,824	AX-84662076
LDB3	rs12258339	chr10:88,426,551-88,495,824	AX-29753435
LDB3	rs12260726	chr10:88,426,551-88,495,824	AX-38900361
LDB3	rs57219964	chr10:88,426,551-88,495,824	AX-29753455
LDB3	rs115120690	chr10:88,426,551-88,495,824	AX-156278614
LDB3	rs115120690	chr10:88,426,551-88,495,824	AX-92455242
LDB3	rs111668579	chr10:88,426,551-88,495,824	AX-156294834
LDB3	rs17106956	chr10:88,426,551-88,495,824	AX-38900365
LDB3	---	chr10:88,426,551-88,495,824	AX-91995800
LDB3	---	chr10:88,426,551-88,495,824	AX-92020927
LDB3	rs138251566	chr10:88,426,551-88,495,824	AX-86664758
LDB3	rs17106959	chr10:88,426,551-88,495,824	AX-156279449
LDB3	rs17106959	chr10:88,426,551-88,495,824	AX-156295146
LDB3	rs17426017	chr10:88,426,551-88,495,824	AX-38900371
LDB3	rs17426050	chr10:88,426,551-88,495,824	AX-107775749
LDB3	rs17426050	chr10:88,426,551-88,495,824	AX-156295147
LDB3	rs17106968	chr10:88,426,551-88,495,824	AX-156278616
LDB3	rs17106968	chr10:88,426,551-88,495,824	AX-156294835
LDB3	rs78837280	chr10:88,426,551-88,495,824	AX-29753471
LDB3	rs10788526	chr10:88,426,551-88,495,824	AX-88907637
LDB3	rs3740347	chr10:88,426,551-88,495,824	AX-147797807
LDB3	rs3740347	chr10:88,426,551-88,495,824	AX-147891694

LDB3	rs11597201	chr10:88,426,551-88,495,824	AX-156278617
LDB3	rs11597201	chr10:88,426,551-88,495,824	AX-156294836
LDB3	rs10887651	chr10:88,426,551-88,495,824	AX-29753479
LDB3	rs7907134	chr10:88,426,551-88,495,824	AX-29753481
LDB3	rs11202136	chr10:88,426,551-88,495,824	AX-122990539
LDB3	rs11202136	chr10:88,426,551-88,495,824	AX-122990540
LDB3	rs61857119	chr10:88,426,551-88,495,824	AX-122990542
LDB3	rs4934252	chr10:88,426,551-88,495,824	AX-38900387
LDB3	rs114459062	chr10:88,426,551-88,495,824	AX-122004029
LDB3	rs114459062	chr10:88,426,551-88,495,824	AX-148678546
LDB3	rs115462341	chr10:88,426,551-88,495,824	AX-29753487
LDB3	rs12263789	chr10:88,426,551-88,495,824	AX-148200208
LDB3	rs12263789	chr10:88,426,551-88,495,824	AX-148271944
LDB3	rs59709955	chr10:88,426,551-88,495,824	AX-29753491
LDB3	rs6586024	chr10:88,426,551-88,495,824	AX-38900389
LDB3	rs6586025	chr10:88,426,551-88,495,824	AX-38900391
LDB3	rs78214136	chr10:88,426,551-88,495,824	AX-156279450
LDB3	rs78214136	chr10:88,426,551-88,495,824	AX-156295148
LDB3	rs55851450	chr10:88,426,551-88,495,824	AX-29753499
LDB3	rs74150398	chr10:88,426,551-88,495,824	AX-156279451
LDB3	rs74150398	chr10:88,426,551-88,495,824	AX-156295149
LDB3	rs12218046	chr10:88,426,551-88,495,824	AX-29753507
LDB3	---	chr10:88,426,551-88,495,824	AX-29753513
LDB3	rs138951890	chr10:88,426,551-88,495,824	AX-83156469
LDB3	rs374426474	chr10:88,426,551-88,495,824	AX-94379840
LDB3	---	chr10:88,426,551-88,495,824	AX-86555906
LDB3	rs7910307	chr10:88,426,551-88,495,824	AX-29753515
LDB3	rs6586026	chr10:88,426,551-88,495,824	AX-29753517
LDB3	rs6586027	chr10:88,426,551-88,495,824	AX-29753519
LDB3	---	chr10:88,426,551-88,495,824	AX-86572756
LDB3	rs11202138	chr10:88,426,551-88,495,824	AX-38900395
LDB3	rs76870416	chr10:88,426,551-88,495,824	AX-29753533
LDB3	rs12770250	chr10:88,426,551-88,495,824	AX-119662868
LDB3	rs12770250	chr10:88,426,551-88,495,824	AX-92496214
LDB3	rs7909928	chr10:88,426,551-88,495,824	AX-148413163
LDB3	rs7909928	chr10:88,426,551-88,495,824	AX-156278618
LDB3	---	chr10:88,426,551-88,495,824	AX-151194499
LDB3	---	chr10:88,426,551-88,495,824	AX-156285620
LDB3	rs35569375	chr10:88,426,551-88,495,824	AX-29753541
LDB3	rs71487278	chr10:88,426,551-88,495,824	AX-151210155
LDB3	rs12570979	chr10:88,426,551-88,495,824	AX-122546023
LDB3	rs7897549	chr10:88,426,551-88,495,824	AX-29753551
LDB3	rs4408238	chr10:88,426,551-88,495,824	AX-38900403
LDB3	rs4525132	chr10:88,426,551-88,495,824	AX-16419567
LDB3	rs4620630	chr10:88,426,551-88,495,824	AX-38900405
LDB3	rs143925234	chr10:88,426,551-88,495,824	AX-148519622
LDB3	rs75262790	chr10:88,426,551-88,495,824	AX-148300758
LDB3	rs12771609	chr10:88,426,551-88,495,824	AX-148247504
LDB3	rs12771609	chr10:88,426,551-88,495,824	AX-96883440
LDB3	rs4611110	chr10:88,426,551-88,495,824	AX-156278619
LDB3	rs4611110	chr10:88,426,551-88,495,824	AX-156294838
LDB3	rs4556455	chr10:88,426,551-88,495,824	AX-156294839
LDB3	rs58389996	chr10:88,426,551-88,495,824	AX-123012587
LDB3	rs58389996	chr10:88,426,551-88,495,824	AX-148225684
LDB3	rs7918367	chr10:88,426,551-88,495,824	AX-148221726

LDB3	rs7918367	chr10:88,426,551-88,495,824	AX-92575197
LDB3	rs7092188	chr10:88,426,551-88,495,824	AX-38900411
LDB3	rs45514002	chr10:88,426,551-88,495,824	AX-60793033
LDB3	rs549994359	chr10:88,426,551-88,495,824	AX-86669239
LDB3	rs45577134	chr10:88,426,551-88,495,824	AX-83004192
LDB3	rs34647099	chr10:88,426,551-88,495,824	AX-11450527
LDB3	rs17335168	chr10:88,426,551-88,495,824	AX-11320068
LDB3	rs6586029	chr10:88,426,551-88,495,824	AX-148028412
LDB3	rs6586029	chr10:88,426,551-88,495,824	AX-96910085
LDB3	rs7901128	chr10:88,426,551-88,495,824	AX-151319274
LDB3	rs7901128	chr10:88,426,551-88,495,824	AX-156283632
LDB3	rs201310813	chr10:88,426,551-88,495,824	AX-120340158
LDB3	rs201310813	chr10:88,426,551-88,495,824	AX-156286415
LDB3	rs6586031	chr10:88,426,551-88,495,824	AX-38900419
LDB3	rs11202140	chr10:88,426,551-88,495,824	AX-148694902
LDB3	rs7908765	chr10:88,426,551-88,495,824	AX-11653125
LDB3	rs74534603	chr10:88,426,551-88,495,824	AX-16419577
LDB3	rs35496319	chr10:88,426,551-88,495,824	AX-151194276
LDB3	rs35496319	chr10:88,426,551-88,495,824	AX-156297877
LDB3	rs67467603	chr10:88,426,551-88,495,824	AX-120937137
LDB3	rs74150399	chr10:88,426,551-88,495,824	AX-29753569
LDB3	rs12262050	chr10:88,426,551-88,495,824	AX-148668636
LDB3	rs12262050	chr10:88,426,551-88,495,824	AX-156278621
LDB3	rs61857148	chr10:88,426,551-88,495,824	AX-148006751
LDB3	rs61857148	chr10:88,426,551-88,495,824	AX-153819006
LDB3	rs58418100	chr10:88,426,551-88,495,824	AX-148086318
LDB3	rs58418100	chr10:88,426,551-88,495,824	AX-156283633
LDB3	rs7916248	chr10:88,426,551-88,495,824	AX-156278622
LDB3	rs7916248	chr10:88,426,551-88,495,824	AX-156294840
LDB3	rs59412880	chr10:88,426,551-88,495,824	AX-151320159
LDB3	rs59412880	chr10:88,426,551-88,495,824	AX-156283634
LDB3	rs7899324	chr10:88,426,551-88,495,824	AX-148251372
LDB3	rs7899324	chr10:88,426,551-88,495,824	AX-156278623
LDB3	rs7899337	chr10:88,426,551-88,495,824	AX-151117236
LDB3	rs7899337	chr10:88,426,551-88,495,824	AX-156278624
LDB3	rs34218952	chr10:88,426,551-88,495,824	AX-12551711
LDB3	rs45516103	chr10:88,426,551-88,495,824	AX-11510513
LDB3	rs35222021	chr10:88,426,551-88,495,824	AX-156278625
LDB3	rs35222021	chr10:88,426,551-88,495,824	AX-156294841
LDB3	rs74150400	chr10:88,426,551-88,495,824	AX-84769178
LMNA	---	chr1:156,084,461-156,109,880	AX-90078922
LMNA	---	chr1:156,084,461-156,109,880	AX-90026405
LMNA	---	chr1:156,084,461-156,109,880	AX-90072999
LMNA	rs267607614	chr1:156,084,461-156,109,880	AX-90057716
LMNA	rs56694480	chr1:156,084,461-156,109,880	AX-90070303
LMNA	rs267607601	chr1:156,084,461-156,109,880	AX-90026406
LMNA	---	chr1:156,084,461-156,109,880	AX-90036615
LMNA	rs267607627	chr1:156,084,461-156,109,880	AX-90056943
LMNA	rs57983345	chr1:156,084,461-156,109,880	AX-90070316
LMNA	---	chr1:156,084,461-156,109,880	AX-90027631
LMNA	rs60446065	chr1:156,084,461-156,109,880	AX-90041458
LMNA	rs58436778	chr1:156,084,461-156,109,880	AX-90070322
LMNA	rs267607608	chr1:156,084,461-156,109,880	AX-90030363
LMNA	---	chr1:156,084,461-156,109,880	AX-90032303
LMNA	rs59931416	chr1:156,084,461-156,109,880	AX-90041455

LMNA	rs60695352	chr1:156,084,461-156,109,880	AX-90041462
LMNA	rs397517895	chr1:156,084,461-156,109,880	AX-90056472
LMNA	rs60290646	chr1:156,084,461-156,109,880	AX-88797813
LMNA	rs28928903	chr1:156,084,461-156,109,880	AX-90041411
LMNA	rs58922911	chr1:156,084,461-156,109,880	AX-90070330
LMNA	rs28928900	chr1:156,084,461-156,109,880	AX-90070296
LMNA	rs56793579	chr1:156,084,461-156,109,880	AX-90070305
LMNA	---	chr1:156,084,461-156,109,880	AX-90058773
LMNA	---	chr1:156,084,461-156,109,880	AX-90062394
LMNA	---	chr1:156,084,461-156,109,880	AX-90028866
LMNA	rs59270054	chr1:156,084,461-156,109,880	AX-88754765
LMNA	---	chr1:156,084,461-156,109,880	AX-90028887
LMNA	---	chr1:156,084,461-156,109,880	AX-88754763
LMNA	rs267607560	chr1:156,084,461-156,109,880	AX-90037359
LMNA	rs267607568	chr1:156,084,461-156,109,880	AX-90026407
LMNA	---	chr1:156,084,461-156,109,880	AX-90032547
LMNA	---	chr1:156,084,461-156,109,880	AX-88754772
LMNA	---	chr1:156,084,461-156,109,880	AX-90079932
LMNA	---	chr1:156,084,461-156,109,880	AX-90072289
LMNA	rs547915	chr1:156,084,461-156,109,880	AX-11544386
LMNA	rs481995	chr1:156,084,461-156,109,880	AX-148398638
LMNA	rs481995	chr1:156,084,461-156,109,880	AX-156266010
LMNA	rs675661	chr1:156,084,461-156,109,880	AX-148604618
LMNA	rs675661	chr1:156,084,461-156,109,880	AX-86897221
LMNA	rs582690	chr1:156,084,461-156,109,880	AX-39099859
LMNA	rs584025	chr1:156,084,461-156,109,880	AX-39099861
LMNA	rs517606	chr1:156,084,461-156,109,880	AX-148378277
LMNA	rs517606	chr1:156,084,461-156,109,880	AX-156270489
LMNA	rs503815	chr1:156,084,461-156,109,880	AX-30126917
LMNA	rs609625	chr1:156,084,461-156,109,880	AX-156266011
LMNA	rs609625	chr1:156,084,461-156,109,880	AX-156286662
LMNA	rs501791	chr1:156,084,461-156,109,880	AX-16629042
LMNA	rs610918	chr1:156,084,461-156,109,880	AX-30126921
LMNA	rs653969	chr1:156,084,461-156,109,880	AX-156266012
LMNA	rs653969	chr1:156,084,461-156,109,880	AX-156286663
LMNA	rs521354	chr1:156,084,461-156,109,880	AX-156266013
LMNA	rs521354	chr1:156,084,461-156,109,880	AX-156286664
LMNA	rs60880750	chr1:156,084,461-156,109,880	AX-151284027
LMNA	rs60880750	chr1:156,084,461-156,109,880	AX-156285358
LMNA	rs6686943	chr1:156,084,461-156,109,880	AX-115127940
LMNA	rs6686943	chr1:156,084,461-156,109,880	AX-115162260
LMNA	rs693671	chr1:156,084,461-156,109,880	AX-39099865
LMNA	rs9427236	chr1:156,084,461-156,109,880	AX-39099867
LMNA	rs2993269	chr1:156,084,461-156,109,880	AX-30126945
LMNA	rs500940	chr1:156,084,461-156,109,880	AX-105168673
LMNA	rs500940	chr1:156,084,461-156,109,880	AX-156266014
LMNA	rs671728	chr1:156,084,461-156,109,880	AX-148463046
LMNA	rs672200	chr1:156,084,461-156,109,880	AX-156266015
LMNA	rs672200	chr1:156,084,461-156,109,880	AX-156286665
LMNA	rs2993268	chr1:156,084,461-156,109,880	AX-30126955
LMNA	rs2993267	chr1:156,084,461-156,109,880	AX-30126957
LMNA	rs4661147	chr1:156,084,461-156,109,880	AX-50055237
LMNA	rs2485676	chr1:156,084,461-156,109,880	AX-39099873
LMNA	rs111616133	chr1:156,084,461-156,109,880	AX-148263576
LMNA	rs111616133	chr1:156,084,461-156,109,880	AX-151285710

LMNA	rs544627	chr1:156,084,461-156,109,880	AX-148470750
LMNA	rs544627	chr1:156,084,461-156,109,880	AX-156279832
LMNA	rs471679	chr1:156,084,461-156,109,880	AX-151245862
LMNA	rs471679	chr1:156,084,461-156,109,880	AX-156279833
LMNA	rs581342	chr1:156,084,461-156,109,880	AX-148430678
LMNA	rs581342	chr1:156,084,461-156,109,880	AX-156279834
LMNA	rs2485675	chr1:156,084,461-156,109,880	AX-148467916
LMNA	rs2485675	chr1:156,084,461-156,109,880	AX-156279835
LMNA	rs2485674	chr1:156,084,461-156,109,880	AX-123008043
LMNA	rs2485674	chr1:156,084,461-156,109,880	AX-156279836
LMNA	rs2485673	chr1:156,084,461-156,109,880	AX-148463064
LMNA	rs150950685	chr1:156,084,461-156,109,880	AX-156285865
LMNA	rs2485672	chr1:156,084,461-156,109,880	AX-148477606
LMNA	rs2485672	chr1:156,084,461-156,109,880	AX-156279838
LMNA	rs2475758	chr1:156,084,461-156,109,880	AX-156279839
LMNA	rs2475758	chr1:156,084,461-156,109,880	AX-156295417
LMNA	rs6657367	chr1:156,084,461-156,109,880	AX-30126967
LMNA	rs593987	chr1:156,084,461-156,109,880	AX-113007831
LMNA	rs594028	chr1:156,084,461-156,109,880	AX-113402749
LMNA	rs594028	chr1:156,084,461-156,109,880	AX-113959340
LMNA	rs622834	chr1:156,084,461-156,109,880	AX-39099875
LMNA	rs623189	chr1:156,084,461-156,109,880	AX-30126975
LMNA	rs2430417	chr1:156,084,461-156,109,880	AX-156266016
LMNA	rs113448831	chr1:156,084,461-156,109,880	AX-148754045
LMNA	rs113448831	chr1:156,084,461-156,109,880	AX-156266017
LMNA	rs573739	chr1:156,084,461-156,109,880	AX-156266018
LMNA	rs573739	chr1:156,084,461-156,109,880	AX-156286666
LMNA	rs573735	chr1:156,084,461-156,109,880	AX-156266019
LMNA	rs573735	chr1:156,084,461-156,109,880	AX-156286667
LMNA	rs59351839	chr1:156,084,461-156,109,880	AX-30126983
LMNA	rs16837187	chr1:156,084,461-156,109,880	AX-120364795
LMNA	rs16837187	chr1:156,084,461-156,109,880	AX-156266020
LMNA	rs528636	chr1:156,084,461-156,109,880	AX-39099879
LMNA	rs543235	chr1:156,084,461-156,109,880	AX-30126987
LMNA	rs79200804	chr1:156,084,461-156,109,880	AX-156266021
LMNA	rs79200804	chr1:156,084,461-156,109,880	AX-156286668
LMNA	---	chr1:156,084,461-156,109,880	AX-30126989
LMNA	rs16837193	chr1:156,084,461-156,109,880	AX-30126997
LMNA	rs665979	chr1:156,084,461-156,109,880	AX-156265645
LMNA	rs665979	chr1:156,084,461-156,109,880	AX-156265680
LMNA	rs513043	chr1:156,084,461-156,109,880	AX-30127003
LMNA	rs666869	chr1:156,084,461-156,109,880	AX-39099881
LMNA	rs509551	chr1:156,084,461-156,109,880	AX-39099883
LMNA	rs508641	chr1:156,084,461-156,109,880	AX-115124977
LMNA	rs508641	chr1:156,084,461-156,109,880	AX-115163986
LMNA	rs113265975	chr1:156,084,461-156,109,880	AX-151323505
LMNA	rs113265975	chr1:156,084,461-156,109,880	AX-156286316
LMNA	rs267607569	chr1:156,084,461-156,109,880	AX-90060400
LMNA	---	chr1:156,084,461-156,109,880	AX-90073624
LMNA	---	chr1:156,084,461-156,109,880	AX-90056249
LMNA	---	chr1:156,084,461-156,109,880	AX-90063106
LMNA	rs61726478	chr1:156,084,461-156,109,880	AX-90041480
LMNA	---	chr1:156,084,461-156,109,880	AX-90026892
LMNA	---	chr1:156,084,461-156,109,880	AX-90055778
LMNA	rs267607649	chr1:156,084,461-156,109,880	AX-90032945

LMNA	---	chr1:156,084,461-156,109,880	AX-90055779
LMNA	rs61661343	chr1:156,084,461-156,109,880	AX-88754771
LMNA	rs58912633	chr1:156,084,461-156,109,880	AX-90070328
LMNA	rs60310264	chr1:156,084,461-156,109,880	AX-90041457
LMNA	rs80356805	chr1:156,084,461-156,109,880	AX-30127011
LMNA	---	chr1:156,084,461-156,109,880	AX-90071777
LMNA	rs28933093	chr1:156,084,461-156,109,880	AX-90041412
LMNA	rs267607594	chr1:156,084,461-156,109,880	AX-90031444
LMNA	rs267607595	chr1:156,084,461-156,109,880	AX-90051413
LMNA	rs267607542	chr1:156,084,461-156,109,880	AX-90036117
LMNA	rs577492	chr1:156,084,461-156,109,880	AX-12590963
LMNA	rs480886	chr1:156,084,461-156,109,880	AX-113443708
LMNA	rs480886	chr1:156,084,461-156,109,880	AX-153404679
LMNA	rs682267	chr1:156,084,461-156,109,880	AX-148346302
LMNA	rs682267	chr1:156,084,461-156,109,880	AX-92735375
LMNA	rs2485669	chr1:156,084,461-156,109,880	AX-122953736
LMNA	rs2485669	chr1:156,084,461-156,109,880	AX-144916330
LMNA	rs60659614	chr1:156,084,461-156,109,880	AX-151271232
LMNA	rs60659614	chr1:156,084,461-156,109,880	AX-156269758
LMNA	rs2485668	chr1:156,084,461-156,109,880	AX-39099893
LMNA	rs2485667	chr1:156,084,461-156,109,880	AX-11456315
LMNA	rs2485667	chr1:156,084,461-156,109,880	AX-149286709
LMNA	rs2430416	chr1:156,084,461-156,109,880	AX-156270490
LMNA	rs2430416	chr1:156,084,461-156,109,880	AX-92442759
LMNA	rs2430415	chr1:156,084,461-156,109,880	AX-148460408
LMNA	rs2430415	chr1:156,084,461-156,109,880	AX-156270491
LMNA	rs2908712	chr1:156,084,461-156,109,880	AX-105052667
LMNA	rs2908712	chr1:156,084,461-156,109,880	AX-118438758
LMNA	rs2993266	chr1:156,084,461-156,109,880	AX-151279467
LMNA	rs10158206	chr1:156,084,461-156,109,880	AX-156266022
LMNA	rs10159320	chr1:156,084,461-156,109,880	AX-156266023
LMNA	rs10159320	chr1:156,084,461-156,109,880	AX-156286670
LMNA	rs10158218	chr1:156,084,461-156,109,880	AX-115127792
LMNA	rs10158218	chr1:156,084,461-156,109,880	AX-115161084
LMNA	rs201479816	chr1:156,084,461-156,109,880	AX-119325321
LMNA	rs111340905	chr1:156,084,461-156,109,880	AX-113609617
LMNA	rs111340905	chr1:156,084,461-156,109,880	AX-92435440
LMNA	rs2485666	chr1:156,084,461-156,109,880	AX-156266024
LMNA	rs2485666	chr1:156,084,461-156,109,880	AX-92569803
LMNA	rs2485665	chr1:156,084,461-156,109,880	AX-144909254
LMNA	rs2430414	chr1:156,084,461-156,109,880	AX-148527670
LMNA	rs2430414	chr1:156,084,461-156,109,880	AX-156266025
LMNA	rs113044442	chr1:156,084,461-156,109,880	AX-123031939
LMNA	rs113044442	chr1:156,084,461-156,109,880	AX-148624245
LMNA	rs56702163	chr1:156,084,461-156,109,880	AX-30127023
LMNA	rs569025	chr1:156,084,461-156,109,880	AX-30127025
LMNA	rs78499673	chr1:156,084,461-156,109,880	AX-156266026
LMNA	rs78499673	chr1:156,084,461-156,109,880	AX-156286671
LMNA	rs267607583	chr1:156,084,461-156,109,880	AX-90055294
LMNA	rs61726479	chr1:156,084,461-156,109,880	AX-88754773
LMNA	rs267607626	chr1:156,084,461-156,109,880	AX-86553269
LMNA	rs59026483	chr1:156,084,461-156,109,880	AX-88754762
LMNA	rs267607571	chr1:156,084,461-156,109,880	AX-90027147
LMNA	---	chr1:156,084,461-156,109,880	AX-88797802
LMNA	---	chr1:156,084,461-156,109,880	AX-90032009

LMNA	---	chr1:156,084,461-156,109,880	AX-88754751
LMNA	---	chr1:156,084,461-156,109,880	AX-90059451
LMNA	rs267607541	chr1:156,084,461-156,109,880	AX-90053763
LMNA	rs61195471	chr1:156,084,461-156,109,880	AX-88754770
LMNA	---	chr1:156,084,461-156,109,880	AX-88777223
LMNA	---	chr1:156,084,461-156,109,880	AX-90037328
LMNA	rs267607629	chr1:156,084,461-156,109,880	AX-90056726
LMNA	---	chr1:156,084,461-156,109,880	AX-90051650
LMNA	rs267607572	chr1:156,084,461-156,109,880	AX-90026410
LMNA	---	chr1:156,084,461-156,109,880	AX-91995886
LMNA	---	chr1:156,084,461-156,109,880	AX-92021013
LMNA	rs11264443	chr1:156,084,461-156,109,880	AX-30127027
LMNA	---	chr1:156,084,461-156,109,880	AX-90027883
LMNA	rs267607584	chr1:156,084,461-156,109,880	AX-90026412
LMNA	---	chr1:156,084,461-156,109,880	AX-90026413
LMNA	rs28928901	chr1:156,084,461-156,109,880	AX-90038279
LMNA	rs58034145	chr1:156,084,461-156,109,880	AX-90070317
LMNA	rs60682848	chr1:156,084,461-156,109,880	AX-88797814
LMNA	rs61214927	chr1:156,084,461-156,109,880	AX-90070355
LMNA	---	chr1:156,084,461-156,109,880	AX-90057791
LMNA	rs57207746	chr1:156,084,461-156,109,880	AX-90041424
LMNA	rs267607573	chr1:156,084,461-156,109,880	AX-90055299
LMNA	rs267607587	chr1:156,084,461-156,109,880	AX-90026415
LMNA	rs58850446	chr1:156,084,461-156,109,880	AX-90041441
LMNA	---	chr1:156,084,461-156,109,880	AX-90070418
LMNA	rs59332535	chr1:156,084,461-156,109,880	AX-90070336
LMNA	---	chr1:156,084,461-156,109,880	AX-90061820
LMNA	rs58048078	chr1:156,084,461-156,109,880	AX-90041432
LMNA	---	chr1:156,084,461-156,109,880	AX-90078610
LMNA	---	chr1:156,084,461-156,109,880	AX-90055301
LMNA	rs267607625	chr1:156,084,461-156,109,880	AX-90058390
LMNA	rs267607593	chr1:156,084,461-156,109,880	AX-90055302
LMNA	rs57048196	chr1:156,084,461-156,109,880	AX-90031110
LMNA	rs267607630	chr1:156,084,461-156,109,880	AX-90030042
LMNA	rs267607631	chr1:156,084,461-156,109,880	AX-90036878
LMNA	rs267607632	chr1:156,084,461-156,109,880	AX-90059621
LMNA	---	chr1:156,084,461-156,109,880	AX-39099897
LMNA	rs11264445	chr1:156,084,461-156,109,880	AX-147853047
LMNA	rs11264445	chr1:156,084,461-156,109,880	AX-147947224
LMNA	---	chr1:156,084,461-156,109,880	AX-90033587
LMNA	---	chr1:156,084,461-156,109,880	AX-90078138
LMNA	rs538089	chr1:156,084,461-156,109,880	AX-30127033
LMNA	---	chr1:156,084,461-156,109,880	AX-90050242
LMNA	---	chr1:156,084,461-156,109,880	AX-90065828
LMNA	rs61616775	chr1:156,084,461-156,109,880	AX-90041477
LMNA	rs267607633	chr1:156,084,461-156,109,880	AX-90057072
LMNA	rs59885338	chr1:156,084,461-156,109,880	AX-88797811
LMNA	rs150924946	chr1:156,084,461-156,109,880	AX-83451856
LMNA	rs267607591	chr1:156,084,461-156,109,880	AX-90031345
LMNA	rs267607596	chr1:156,084,461-156,109,880	AX-90033210
LMNA	rs61527854	chr1:156,084,461-156,109,880	AX-90070360
LMNA	---	chr1:156,084,461-156,109,880	AX-90028872
LMNA	---	chr1:156,084,461-156,109,880	AX-90058647
LMNA	rs2485664	chr1:156,084,461-156,109,880	AX-113476645
LMNA	rs2485664	chr1:156,084,461-156,109,880	AX-113885432

LMNA	rs76017998	chr1:156,084,461-156,109,880	AX-30127035
LMNA	---	chr1:156,084,461-156,109,880	AX-39099901
LMNA	rs267607645	chr1:156,084,461-156,109,880	AX-90031567
LMNA	rs56816490	chr1:156,084,461-156,109,880	AX-90070306
LMNA	rs56771886	chr1:156,084,461-156,109,880	AX-90053762
LMNA	rs267607554	chr1:156,084,461-156,109,880	AX-90026418
LMNA	rs56851164	chr1:156,084,461-156,109,880	AX-86688414
LMNA	---	chr1:156,084,461-156,109,880	AX-90026419
LMNA	rs386134243	chr1:156,084,461-156,109,880	AX-90035738
LMNA	rs58105277	chr1:156,084,461-156,109,880	AX-90041433
LMNA	rs267607548	chr1:156,084,461-156,109,880	AX-90026420
LMNA	rs267607555	chr1:156,084,461-156,109,880	AX-90034814
LMNA	rs267607610	chr1:156,084,461-156,109,880	AX-90031691
LMNA	---	chr1:156,084,461-156,109,880	AX-90073604
LMNA	rs267607623	chr1:156,084,461-156,109,880	AX-90055306
LMNA	---	chr1:156,084,461-156,109,880	AX-90027271
LMNA	rs267607617	chr1:156,084,461-156,109,880	AX-90055307
LMNA	rs267607567	chr1:156,084,461-156,109,880	AX-90055308
LMNA	rs267607634	chr1:156,084,461-156,109,880	AX-90031533
LMNA	rs58389804	chr1:156,084,461-156,109,880	AX-90082074
LMNA	---	chr1:156,084,461-156,109,880	AX-90074346
LMNA	rs397517889	chr1:156,084,461-156,109,880	AX-90031126
LMNA	---	chr1:156,084,461-156,109,880	AX-90026895
LMNA	---	chr1:156,084,461-156,109,880	AX-90035641
LMNA	rs121912495	chr1:156,084,461-156,109,880	AX-90041532
LMNA	---	chr1:156,084,461-156,109,880	AX-90034738
LMNA	---	chr1:156,084,461-156,109,880	AX-90027423
LMNA	rs534807	chr1:156,084,461-156,109,880	AX-116090745
LMNA	rs534807	chr1:156,084,461-156,109,880	AX-147961263
LMNA	rs16837198	chr1:156,084,461-156,109,880	AX-30127039
LMNA	rs267607561	chr1:156,084,461-156,109,880	AX-86674420
LMNA	rs58672172	chr1:156,084,461-156,109,880	AX-90070325
LMNA	rs61094188	chr1:156,084,461-156,109,880	AX-90041468
LMNA	rs267607647	chr1:156,084,461-156,109,880	AX-90057752
LMNA	rs267607618	chr1:156,084,461-156,109,880	AX-90055310
LMNA	---	chr1:156,084,461-156,109,880	AX-90079740
LMNA	rs150840924	chr1:156,084,461-156,109,880	AX-86621648
LMNA	rs62636506	chr1:156,084,461-156,109,880	AX-86638940
LMNA	rs121912493	chr1:156,084,461-156,109,880	AX-90041530
LMNA	---	chr1:156,084,461-156,109,880	AX-90078879
LMNA	rs58541611	chr1:156,084,461-156,109,880	AX-90041437
LMNA	rs505058	chr1:156,084,461-156,109,880	AX-39099903
LMNA	---	chr1:156,084,461-156,109,880	AX-90059650
LMNA	rs58932704	chr1:156,084,461-156,109,880	AX-86736819
LMNA	rs267607598	chr1:156,084,461-156,109,880	AX-90027802
LMNA	rs267607597	chr1:156,084,461-156,109,880	AX-90064435
LMNA	---	chr1:156,084,461-156,109,880	AX-90075774
LMNA	rs60992550	chr1:156,084,461-156,109,880	AX-90069071
LMNA	rs61235244	chr1:156,084,461-156,109,880	AX-90070357
LMNA	rs476000	chr1:156,084,461-156,109,880	AX-39099905
LMNA	rs267607600	chr1:156,084,461-156,109,880	AX-90029474
LMNA	---	chr1:156,084,461-156,109,880	AX-90061149
LMNA	rs267607642	chr1:156,084,461-156,109,880	AX-90065601
LMNA	rs61282106	chr1:156,084,461-156,109,880	AX-90070358
LMNA	---	chr1:156,084,461-156,109,880	AX-90067165

LMNA	rs267607578	chr1:156,084,461-156,109,880	AX-90056791
LMNA	---	chr1:156,084,461-156,109,880	AX-90075099
LMNA	rs57747780	chr1:156,084,461-156,109,880	AX-90070313
LMNA	rs57920071	chr1:156,084,461-156,109,880	AX-90070315
LMNA	---	chr1:156,084,461-156,109,880	AX-90056776
LMNA	---	chr1:156,084,461-156,109,880	AX-90027673
LMNA	---	chr1:156,084,461-156,109,880	AX-90036151
LMNA	---	chr1:156,084,461-156,109,880	AX-90061381
LMNA	rs56699480	chr1:156,084,461-156,109,880	AX-90041418
LMNA	rs267607640	chr1:156,084,461-156,109,880	AX-90027761
LMNA	---	chr1:156,084,461-156,109,880	AX-90063138
LMNA	rs553016	chr1:156,084,461-156,109,880	AX-39099907
LMNA	rs60556110	chr1:156,084,461-156,109,880	AX-90048799
LMNA	rs57730570	chr1:156,084,461-156,109,880	AX-90035675
LMNA	rs267607580	chr1:156,084,461-156,109,880	AX-90049209
LMNA	rs267607553	chr1:156,084,461-156,109,880	AX-90046858
LMNA	rs267607565	chr1:156,084,461-156,109,880	AX-90035351
LMNA	rs57877560	chr1:156,084,461-156,109,880	AX-90070314
LMNA	---	chr1:156,084,461-156,109,880	AX-90062851
LMNA	rs267607557	chr1:156,084,461-156,109,880	AX-90029987
LMNA	rs58362413	chr1:156,084,461-156,109,880	AX-90041435
LMNA	rs201583907	chr1:156,084,461-156,109,880	AX-83038703
LMNA	rs57318642	chr1:156,084,461-156,109,880	AX-90041425
LMNA	---	chr1:156,084,461-156,109,880	AX-86713456
LMNA	---	chr1:156,084,461-156,109,880	AX-90026897
LMNA	---	chr1:156,084,461-156,109,880	AX-90035052
LMNA	---	chr1:156,084,461-156,109,880	AX-90058322
LMNA	rs121912494	chr1:156,084,461-156,109,880	AX-90041531
LMNA	rs60580541	chr1:156,084,461-156,109,880	AX-90041460
LMNA	rs60934003	chr1:156,084,461-156,109,880	AX-90041464
LMNA	rs267607592	chr1:156,084,461-156,109,880	AX-90029430
LMNA	---	chr1:156,084,461-156,109,880	AX-90030229
LMNA	rs267607539	chr1:156,084,461-156,109,880	AX-90033395
LMNA	rs267607581	chr1:156,084,461-156,109,880	AX-90065095
LMNA	rs267607547	chr1:156,084,461-156,109,880	AX-90065867
LMNA	---	chr1:156,084,461-156,109,880	AX-90062651
LMNA	---	chr1:156,084,461-156,109,880	AX-90070359
LMNA	rs56673169	chr1:156,084,461-156,109,880	AX-90070302
LMNA	rs267607613	chr1:156,084,461-156,109,880	AX-90057893
LMNA	rs4641	chr1:156,084,461-156,109,880	AX-83578059
LMNA	---	chr1:156,084,461-156,109,880	AX-90060582
LMNA	rs520973	chr1:156,084,461-156,109,880	AX-39099915
LMNA	rs520910	chr1:156,084,461-156,109,880	AX-30127041
LMNA	rs61224243	chr1:156,084,461-156,109,880	AX-90070356
LMNA	---	chr1:156,084,461-156,109,880	AX-90027440
LMNA	rs267607556	chr1:156,084,461-156,109,880	AX-90065400
LMNA	---	chr1:156,084,461-156,109,880	AX-86616208
LMNA	rs59886214	chr1:156,084,461-156,109,880	AX-88754767
LMNA	rs61064130	chr1:156,084,461-156,109,880	AX-90070352
LMNA	rs59267781	chr1:156,084,461-156,109,880	AX-90041448
LMNA	rs140455668	chr1:156,084,461-156,109,880	AX-86617665
LMNA	---	chr1:156,084,461-156,109,880	AX-90030464
LMNA	rs267607612	chr1:156,084,461-156,109,880	AX-90058146
LMNA	---	chr1:156,084,461-156,109,880	AX-90058246
LMNA	rs368386019	chr1:156,084,461-156,109,880	AX-86547734

LMNA	rs267607544	chr1:156,084,461-156,109,880	AX-90059330
LMNA	---	chr1:156,084,461-156,109,880	AX-84989008
LMNA	rs80264244	chr1:156,084,461-156,109,880	AX-30127045
LMNA	rs7339	chr1:156,084,461-156,109,880	AX-147869531
LMNA	rs7339	chr1:156,084,461-156,109,880	AX-84989430
LMNA	rs74116489	chr1:156,084,461-156,109,880	AX-121928444
LMNA	rs74116489	chr1:156,084,461-156,109,880	AX-156266027
LMNA	rs111601180	chr1:156,084,461-156,109,880	AX-156268994
LMNA	rs111601180	chr1:156,084,461-156,109,880	AX-156288596
MURC	---	chr9:103,340,336-103,350,173	AX-83305959
MURC	rs200457015	chr9:103,340,336-103,350,173	AX-83259807
MURC	---	chr9:103,340,336-103,350,173	AX-86648823
MURC	rs370997194	chr9:103,340,336-103,350,173	AX-86652965
MURC	---	chr9:103,340,336-103,350,173	AX-90063588
MURC	rs10989164	chr9:103,340,336-103,350,173	AX-11136615
MYBP3	rs12046163	chr1:236,849,770-236,927,558	AX-156280020
MYBP3	rs12046163	chr1:236,849,770-236,927,558	AX-156295547
MYBP3	rs644401	chr1:236,849,770-236,927,558	AX-16947808
MYBP3	rs707220	chr1:236,849,770-236,927,558	AX-30720957
MYBP3	rs707219	chr1:236,849,770-236,927,558	AX-39409565
MYBP3	rs79277266	chr1:236,849,770-236,927,558	AX-16947813
MYBP3	rs12724121	chr1:236,849,770-236,927,558	AX-30720959
MYBP3	rs189051620	chr1:236,849,770-236,927,558	AX-151153472
MYBP3	rs189051620	chr1:236,849,770-236,927,558	AX-156283210
MYBP3	rs79542980	chr1:236,849,770-236,927,558	AX-16947819
MYBP3	rs4659701	chr1:236,849,770-236,927,558	AX-50103981
MYBP3	rs697660	chr1:236,849,770-236,927,558	AX-39409567
MYBP3	rs75356518	chr1:236,849,770-236,927,558	AX-16947822
MYBP3	rs1768048	chr1:236,849,770-236,927,558	AX-156270945
MYBP3	rs12142143	chr1:236,849,770-236,927,558	AX-30720975
MYBP3	rs3851907	chr1:236,849,770-236,927,558	AX-39409569
MYBP3	rs17648708	chr1:236,849,770-236,927,558	AX-11333100
MYBP3	rs3906614	chr1:236,849,770-236,927,558	AX-39409571
MYBP3	rs35643176	chr1:236,849,770-236,927,558	AX-30720977
MYBP3	rs9943223	chr1:236,849,770-236,927,558	AX-39409575
MYBP3	rs6688531	chr1:236,849,770-236,927,558	AX-148582234
MYBP3	rs6688531	chr1:236,849,770-236,927,558	AX-156270946
MYBP3	rs71721494	chr1:236,849,770-236,927,558	AX-16947839
MYBP3	rs75441357	chr1:236,849,770-236,927,558	AX-16947841
MYBP3	rs7530968	chr1:236,849,770-236,927,558	AX-113568711
MYBP3	rs10802555	chr1:236,849,770-236,927,558	AX-107906340
MYBP3	rs12736189	chr1:236,849,770-236,927,558	AX-16947848
MYBP3	rs707217	chr1:236,849,770-236,927,558	AX-39409589
MYBP3	rs10802556	chr1:236,849,770-236,927,558	AX-156289606
MYBP3	rs707216	chr1:236,849,770-236,927,558	AX-11604875
MYBP3	rs16834213	chr1:236,849,770-236,927,558	AX-16947851
MYBP3	rs78913129	chr1:236,849,770-236,927,558	AX-30721021
MYBP3	rs707213	chr1:236,849,770-236,927,558	AX-39409595
MYBP3	rs10754581	chr1:236,849,770-236,927,558	AX-156270948
MYBP3	rs10754581	chr1:236,849,770-236,927,558	AX-156289607
MYBP3	rs10925201	chr1:236,849,770-236,927,558	AX-156270949
MYBP3	rs12025491	chr1:236,849,770-236,927,558	AX-11186853
MYBP3	rs707211	chr1:236,849,770-236,927,558	AX-39409601
MYBP3	rs697658	chr1:236,849,770-236,927,558	AX-11598491
MYBP3	rs111688304	chr1:236,849,770-236,927,558	AX-113120727

MYBP3	rs111688304	chr1:236,849,770-236,927,558	AX-114050383
MYBP3	rs79108886	chr1:236,849,770-236,927,558	AX-30721029
MYBP3	rs707210	chr1:236,849,770-236,927,558	AX-16947864
MYBP3	rs6672960	chr1:236,849,770-236,927,558	AX-11577504
MYBP3	---	chr1:236,849,770-236,927,558	AX-156286039
MYBP3	rs12130358	chr1:236,849,770-236,927,558	AX-156270950
MYBP3	---	chr1:236,849,770-236,927,558	AX-105090306
MYBP3	rs7539291	chr1:236,849,770-236,927,558	AX-148423773
MYBP3	rs7539291	chr1:236,849,770-236,927,558	AX-156270484
MYBP3	rs819645	chr1:236,849,770-236,927,558	AX-122591189
MYBP3	---	chr1:236,849,770-236,927,558	AX-119954925
MYBP3	rs819646	chr1:236,849,770-236,927,558	AX-119369222
MYBP3	rs819646	chr1:236,849,770-236,927,558	AX-153809006
MYBP3	rs819647	chr1:236,849,770-236,927,558	AX-156289608
MYBP3	rs4659702	chr1:236,849,770-236,927,558	AX-30721041
MYBP3	rs12403616	chr1:236,849,770-236,927,558	AX-39409613
MYBP3	rs12037773	chr1:236,849,770-236,927,558	AX-30721057
MYBP3	rs7516579	chr1:236,849,770-236,927,558	AX-156270954
MYBP3	rs7516579	chr1:236,849,770-236,927,558	AX-156289609
MYBP3	rs4659706	chr1:236,849,770-236,927,558	AX-151160186
MYBP3	rs112267838	chr1:236,849,770-236,927,558	AX-105112175
MYBP3	rs61831762	chr1:236,849,770-236,927,558	AX-113126688
MYBP3	rs35235564	chr1:236,849,770-236,927,558	AX-11460533
MYBP3	rs1613395	chr1:236,849,770-236,927,558	AX-16947875
MYBP3	---	chr1:236,849,770-236,927,558	AX-12459972
MYBP3	rs10925204	chr1:236,849,770-236,927,558	AX-113120728
MYBP3	rs10925204	chr1:236,849,770-236,927,558	AX-113568712
MYBP3	rs61643696	chr1:236,849,770-236,927,558	AX-37445855
MYBP3	rs1773444	chr1:236,849,770-236,927,558	AX-12504019
MYBP3	rs1341861	chr1:236,849,770-236,927,558	AX-39409633
MYBP3	rs1341860	chr1:236,849,770-236,927,558	AX-39409635
MYBP3	rs66469169	chr1:236,849,770-236,927,558	AX-156284584
MYBP3	rs112736198	chr1:236,849,770-236,927,558	AX-156270585
MYBP3	rs112967359	chr1:236,849,770-236,927,558	AX-151408412
MYBP3	rs1652224	chr1:236,849,770-236,927,558	AX-113575750
MYBP3	rs1652224	chr1:236,849,770-236,927,558	AX-11442068
MYBP3	rs947449	chr1:236,849,770-236,927,558	AX-113568713
MYBP3	rs947449	chr1:236,849,770-236,927,558	AX-113603858
MYBP3	---	chr1:236,849,770-236,927,558	AX-151130937
MYBP3	---	chr1:236,849,770-236,927,558	AX-151411135
MYBP3	rs1622149	chr1:236,849,770-236,927,558	AX-113603859
MYBP3	rs1622149	chr1:236,849,770-236,927,558	AX-114050385
MYBP3	rs2914919	chr1:236,849,770-236,927,558	AX-39409657
MYBP3	rs2995046	chr1:236,849,770-236,927,558	AX-39409659
MYBP3	rs1652223	chr1:236,849,770-236,927,558	AX-11280237
MYBP3	rs2794748	chr1:236,849,770-236,927,558	AX-113557744
MYBP3	rs2794748	chr1:236,849,770-236,927,558	AX-114074373
MYBP3	rs12751677	chr1:236,849,770-236,927,558	AX-39409667
MYBP3	rs1934690	chr1:236,849,770-236,927,558	AX-148780390
MYBP3	rs1934690	chr1:236,849,770-236,927,558	AX-149312612
MYBP3	rs1934689	chr1:236,849,770-236,927,558	AX-148780500
MYBP3	rs1934689	chr1:236,849,770-236,927,558	AX-156270957
MYBP3	rs200821726	chr1:236,849,770-236,927,558	AX-156284720
MYBP3	---	chr1:236,849,770-236,927,558	AX-11604865
MYBP3	rs2564765	chr1:236,849,770-236,927,558	AX-16947895

MYBP3	rs36115575	chr1:236,849,770-236,927,558	AX-156270958
MYBP3	---	chr1:236,849,770-236,927,558	AX-113214714
MYBP3	rs819639	chr1:236,849,770-236,927,558	AX-12651762
MYBP3	rs707206	chr1:236,849,770-236,927,558	AX-16947907
MYBP3	rs819725	chr1:236,849,770-236,927,558	AX-122962783
MYBP3	rs819642	chr1:236,849,770-236,927,558	AX-16947908
MYBP3	rs16834283	chr1:236,849,770-236,927,558	AX-11282186
MYBP3	rs707205	chr1:236,849,770-236,927,558	AX-11604863
MYBP3	rs12048865	chr1:236,849,770-236,927,558	AX-39409687
MYBP3	rs707204	chr1:236,849,770-236,927,558	AX-39409689
MYBP3	rs12569148	chr1:236,849,770-236,927,558	AX-16947911
MYBP3	rs16834301	chr1:236,849,770-236,927,558	AX-156270961
MYBP3	rs16834301	chr1:236,849,770-236,927,558	AX-156289613
MYBP3	rs6428968	chr1:236,849,770-236,927,558	AX-16947914
MYBP3	rs2288600	chr1:236,849,770-236,927,558	AX-12524429
MYBP3	rs4659709	chr1:236,849,770-236,927,558	AX-16947917
MYBP3	rs3768123	chr1:236,849,770-236,927,558	AX-16947918
MYBP3	rs76145476	chr1:236,849,770-236,927,558	AX-16947919
MYBP3	rs11355106	chr1:236,849,770-236,927,558	AX-156289614
MYBP3	rs7542508	chr1:236,849,770-236,927,558	AX-39409701
MYBP3	rs10925209	chr1:236,849,770-236,927,558	AX-11131996
MYBP3	rs12164643	chr1:236,849,770-236,927,558	AX-16947921
MYBP3	rs12137719	chr1:236,849,770-236,927,558	AX-16947923
MYBP3	rs12139278	chr1:236,849,770-236,927,558	AX-148157195
MYBP3	rs12139278	chr1:236,849,770-236,927,558	AX-156270963
MYBP3	rs12141939	chr1:236,849,770-236,927,558	AX-148148828
MYBP3	rs202167251	chr1:236,849,770-236,927,558	AX-156285778
MYBP3	rs12119844	chr1:236,849,770-236,927,558	AX-156270962
MYBP3	rs12138531	chr1:236,849,770-236,927,558	AX-148055127
MYBP3	rs4659710	chr1:236,849,770-236,927,558	AX-148164463
MYBP3	rs4659710	chr1:236,849,770-236,927,558	AX-92461107
MYBP3	rs4659711	chr1:236,849,770-236,927,558	AX-12576856
MYBP3	---	chr1:236,849,770-236,927,558	AX-42810105
MYBP3	rs7544174	chr1:236,849,770-236,927,558	AX-12631547
MYBP3	rs2385493	chr1:236,849,770-236,927,558	AX-151171827
MYBP3	rs2891794	chr1:236,849,770-236,927,558	AX-148157140
MYBP3	rs2385496	chr1:236,849,770-236,927,558	AX-148161915
MYBP3	rs7555260	chr1:236,849,770-236,927,558	AX-156270968
MYBP3	rs12137069	chr1:236,849,770-236,927,558	AX-156289617
MYBP3	rs140346044	chr1:236,849,770-236,927,558	AX-156285352
MYBP3	rs35956798	chr1:236,849,770-236,927,558	AX-16947936
MYBP3	rs7553165	chr1:236,849,770-236,927,558	AX-11629303
MYBP3	rs4659466	chr1:236,849,770-236,927,558	AX-16947944
MYBP3	rs12048046	chr1:236,849,770-236,927,558	AX-39409735
MYBP3	rs3818884	chr1:236,849,770-236,927,558	AX-16947947
MYBP3	rs3818883	chr1:236,849,770-236,927,558	AX-11483133
MYBP3	rs112529745	chr1:236,849,770-236,927,558	AX-16947948
MYBP3	rs4659712	chr1:236,849,770-236,927,558	AX-123026531
MYBP3	rs10925212	chr1:236,849,770-236,927,558	AX-123026532
MYBP3	rs10925212	chr1:236,849,770-236,927,558	AX-92755613
MYBP3	rs2385498	chr1:236,849,770-236,927,558	AX-16947951
MYBP3	rs149494099	chr1:236,849,770-236,927,558	AX-151433191
MYBP3	rs149494099	chr1:236,849,770-236,927,558	AX-156285368
MYBP3	rs141984611	chr1:236,849,770-236,927,558	AX-151453442
MYBP3	rs3738544	chr1:236,849,770-236,927,558	AX-16947954

MYBP3	rs3738545	chr1:236,849,770-236,927,558	AX-16947955
MYBP3	---	chr1:236,849,770-236,927,558	AX-90031268
MYBP3	rs4659713	chr1:236,849,770-236,927,558	AX-16947956
MYBP3	rs6668769	chr1:236,849,770-236,927,558	AX-156289620
MYBP3	rs111391247	chr1:236,849,770-236,927,558	AX-151206248
MYBP3	rs111391247	chr1:236,849,770-236,927,558	AX-156284767
MYBP3	rs6677567	chr1:236,849,770-236,927,558	AX-92545609
MYBP3	rs10802559	chr1:236,849,770-236,927,558	AX-156270972
MYBP3	rs10737811	chr1:236,849,770-236,927,558	AX-11117721
MYBP3	---	chr1:236,849,770-236,927,558	AX-90060085
MYBP3	rs60012915	chr1:236,849,770-236,927,558	AX-16947962
MYBP3	rs2297858	chr1:236,849,770-236,927,558	AX-30721225
MYBP3	rs2297859	chr1:236,849,770-236,927,558	AX-113705113
MYBP3	rs2297859	chr1:236,849,770-236,927,558	AX-156295549
MYBP3	rs116518554	chr1:236,849,770-236,927,558	AX-156270974
MYBP3	rs116518554	chr1:236,849,770-236,927,558	AX-156289621
MYBP3	rs2297861	chr1:236,849,770-236,927,558	AX-30721233
MYBP3	rs3768127	chr1:236,849,770-236,927,558	AX-156270975
MYBP3	rs78597259	chr1:236,849,770-236,927,558	AX-156271211
MYBP3	rs12728992	chr1:236,849,770-236,927,558	AX-120532796
MYBP3	rs10925221	chr1:236,849,770-236,927,558	AX-12401077
MYBP3	rs143296056	chr1:236,849,770-236,927,558	AX-148054739
MYBP3	rs35311500	chr1:236,849,770-236,927,558	AX-151373865
MYBP3	rs10925222	chr1:236,849,770-236,927,558	AX-148292691
MYBP3	rs10925222	chr1:236,849,770-236,927,558	AX-156270979
MYBP3	rs12729393	chr1:236,849,770-236,927,558	AX-30721241
MYBP3	rs7534128	chr1:236,849,770-236,927,558	AX-156270980
MYBP3	rs7534128	chr1:236,849,770-236,927,558	AX-156289624
MYBP3	rs11811121	chr1:236,849,770-236,927,558	AX-39409755
MYBP3	rs370677725	chr1:236,849,770-236,927,558	AX-86605346
MYBP3	rs200469353	chr1:236,849,770-236,927,558	AX-86561448
MYBP3	rs12730862	chr1:236,849,770-236,927,558	AX-156289625
MYBP3	rs10925223	chr1:236,849,770-236,927,558	AX-156270982
MYBP3	rs10925223	chr1:236,849,770-236,927,558	AX-92508984
MYBP3	rs4348712	chr1:236,849,770-236,927,558	AX-156270983
MYBP3	rs12735607	chr1:236,849,770-236,927,558	AX-39409757
MYBP3	---	chr1:236,849,770-236,927,558	AX-90046968
MYBP3	rs2282366	chr1:236,849,770-236,927,558	AX-16947972
MYBP3	rs201400656	chr1:236,849,770-236,927,558	AX-105084400
MYBP3	rs201400656	chr1:236,849,770-236,927,558	AX-16947973
MYBP3	rs12063382	chr1:236,849,770-236,927,558	AX-30721255
MYBP3	rs199921605	chr1:236,849,770-236,927,558	AX-156284164
MYBP3	rs1051253	chr1:236,849,770-236,927,558	AX-42810107
MYBP3	rs1803032	chr1:236,849,770-236,927,558	AX-147808280
MYBP3	rs7532533	chr1:236,849,770-236,927,558	AX-16947977
MYH6	rs28488316	chr14:23,851,199-23,877,486	AX-114207420
MYH6	rs453361	chr14:23,851,199-23,877,486	AX-39732455
MYH6	---	chr14:23,851,199-23,877,486	AX-31312035
MYH6	rs10135780	chr14:23,851,199-23,877,486	AX-31312037
MYH6	rs17091268	chr14:23,851,199-23,877,486	AX-39732457
MYH6	rs17091278	chr14:23,851,199-23,877,486	AX-31312039
MYH6	rs138720701	chr14:23,851,199-23,877,486	AX-88753529
MYH6	rs35182223	chr14:23,851,199-23,877,486	AX-156271306
MYH6	rs35182223	chr14:23,851,199-23,877,486	AX-156289835
MYH6	rs11624298	chr14:23,851,199-23,877,486	AX-39732459

MYH6	rs73604564	chr14:23,851,199-23,877,486	AX-113242641
MYH6	rs73604564	chr14:23,851,199-23,877,486	AX-156271307
MYH6	rs8006357	chr14:23,851,199-23,877,486	AX-11659411
MYH6	rs8004990	chr14:23,851,199-23,877,486	AX-39732463
MYH6	rs178637	chr14:23,851,199-23,877,486	AX-12725249
MYH6	rs178638	chr14:23,851,199-23,877,486	AX-39732465
MYH6	---	chr14:23,851,199-23,877,486	AX-156284976
MYH6	rs58671554	chr14:23,851,199-23,877,486	AX-156298612
MYH6	rs17091352	chr14:23,851,199-23,877,486	AX-39732467
MYH6	rs382872	chr14:23,851,199-23,877,486	AX-39732469
MYH6	rs28730764	chr14:23,851,199-23,877,486	AX-120377837
MYH6	rs28730764	chr14:23,851,199-23,877,486	AX-156271308
MYH6	rs45520434	chr14:23,851,199-23,877,486	AX-156271309
MYH6	rs396024	chr14:23,851,199-23,877,486	AX-156265652
MYH6	rs396024	chr14:23,851,199-23,877,486	AX-156265687
MYH6	rs178640	chr14:23,851,199-23,877,486	AX-91983191
MYH6	rs178640	chr14:23,851,199-23,877,486	AX-92008318
MYH6	rs61742476	chr14:23,851,199-23,877,486	AX-83575136
MYH6	rs2071634	chr14:23,851,199-23,877,486	AX-92449788
MYH6	rs2071634	chr14:23,851,199-23,877,486	AX-92660811
MYH6	rs17091383	chr14:23,851,199-23,877,486	AX-39732473
MYH6	rs17091385	chr14:23,851,199-23,877,486	AX-113725977
MYH6	rs17091385	chr14:23,851,199-23,877,486	AX-147878856
MYH6	rs59556853	chr14:23,851,199-23,877,486	AX-31312061
MYH6	rs12893772	chr14:23,851,199-23,877,486	AX-11230781
MYH6	rs178641	chr14:23,851,199-23,877,486	AX-31312063
MYH6	rs60867292	chr14:23,851,199-23,877,486	AX-31312065
MYH6	rs34855944	chr14:23,851,199-23,877,486	AX-31312069
MYH6	rs178642	chr14:23,851,199-23,877,486	AX-39732479
MYH6	rs8022522	chr14:23,851,199-23,877,486	AX-39732481
MYH6	---	chr14:23,851,199-23,877,486	AX-86650469
MYH6	rs137983703	chr14:23,851,199-23,877,486	AX-86644152
MYH6	rs145611185	chr14:23,851,199-23,877,486	AX-82890843
MYH6	---	chr14:23,851,199-23,877,486	AX-90054783
MYH6	rs148558068	chr14:23,851,199-23,877,486	AX-83474886
MYH6	rs34935550	chr14:23,851,199-23,877,486	AX-31312087
MYH6	---	chr14:23,851,199-23,877,486	AX-90054784
MYH6	rs112405990	chr14:23,851,199-23,877,486	AX-31312093
MYH6	rs10147618	chr14:23,851,199-23,877,486	AX-39732487
MYH6	rs365990	chr14:23,851,199-23,877,486	AX-39732489
MYH6	rs267606904	chr14:23,851,199-23,877,486	AX-86699436
MYH6	rs17091453	chr14:23,851,199-23,877,486	AX-39732491
MYH6	rs143978652	chr14:23,851,199-23,877,486	AX-83485655
MYH6	rs28730772	chr14:23,851,199-23,877,486	AX-31312109
MYH6	rs28730773	chr14:23,851,199-23,877,486	AX-31312113
MYH6	---	chr14:23,851,199-23,877,486	AX-86624252
MYH6	rs201193346	chr14:23,851,199-23,877,486	AX-83463949
MYH6	rs267606906	chr14:23,851,199-23,877,486	AX-90025900
MYH6	rs267606903	chr14:23,851,199-23,877,486	AX-90054786
MYH6	rs445754	chr14:23,851,199-23,877,486	AX-39732493
MYH6	rs10149522	chr14:23,851,199-23,877,486	AX-39732495
MYH6	rs10149618	chr14:23,851,199-23,877,486	AX-113725979
MYH6	rs10149618	chr14:23,851,199-23,877,486	AX-156271310
MYH6	rs422068	chr14:23,851,199-23,877,486	AX-122834568
MYH6	rs422068	chr14:23,851,199-23,877,486	AX-156271311

MYH6	rs9323314	chr14:23,851,199-23,877,486	AX-96945480
MYH6	rs267606907	chr14:23,851,199-23,877,486	AX-86544964
MYH6	rs452036	chr14:23,851,199-23,877,486	AX-83027825
MYH6	---	chr14:23,851,199-23,877,486	AX-90025902
MYH6	---	chr14:23,851,199-23,877,486	AX-86626993
MYH6	rs17256246	chr14:23,851,199-23,877,486	AX-39732503
MYH6	rs412768	chr14:23,851,199-23,877,486	AX-92574406
MYH6	rs412768	chr14:23,851,199-23,877,486	AX-92785429
MYH6	rs28730775	chr14:23,851,199-23,877,486	AX-31312125
MYH6	rs17091535	chr14:23,851,199-23,877,486	AX-39732509
MYH6	rs142992009	chr14:23,851,199-23,877,486	AX-83104128
MYH6	rs367663906	chr14:23,851,199-23,877,486	AX-86600385
MYH6	---	chr14:23,851,199-23,877,486	AX-90060714
MYH6	rs439735	chr14:23,851,199-23,877,486	AX-39732511
MYH6	rs17091555	chr14:23,851,199-23,877,486	AX-39732513
MYH6	rs403739	chr14:23,851,199-23,877,486	AX-148202797
MYH6	rs403739	chr14:23,851,199-23,877,486	AX-156280247
MYH6	rs403720	chr14:23,851,199-23,877,486	AX-31312135
MYH6	rs376439	chr14:23,851,199-23,877,486	AX-31312137
MYH6	---	chr14:23,851,199-23,877,486	AX-90054788
MYH6	rs28730776	chr14:23,851,199-23,877,486	AX-31312141
MYH6	rs57166503	chr14:23,851,199-23,877,486	AX-31312143
MYH6	rs28671012	chr14:23,851,199-23,877,486	AX-113872552
MYH6	rs28671012	chr14:23,851,199-23,877,486	AX-156271312
MYH6	rs432256	chr14:23,851,199-23,877,486	AX-31312149
MYH6	rs28730777	chr14:23,851,199-23,877,486	AX-31312153
MYH6	rs78107039	chr14:23,851,199-23,877,486	AX-31312161
MYH6	rs17091623	chr14:23,851,199-23,877,486	AX-39732517
MYH6	rs17091631	chr14:23,851,199-23,877,486	AX-31312163
MYH6	rs434273	chr14:23,851,199-23,877,486	AX-31312167
MYH6	---	chr14:23,851,199-23,877,486	AX-90054789
MYH6	rs11850295	chr14:23,851,199-23,877,486	AX-39732519
MYH6	rs388914	chr14:23,851,199-23,877,486	AX-39732521
MYH6	rs12889823	chr14:23,851,199-23,877,486	AX-39732525
MYH6	---	chr14:23,851,199-23,877,486	AX-90025905
MYH6	rs142027794	chr14:23,851,199-23,877,486	AX-83078661
MYH6	rs440466	chr14:23,851,199-23,877,486	AX-39732527
MYH6	rs2277473	chr14:23,851,199-23,877,486	AX-39732529
MYH6	rs2277474	chr14:23,851,199-23,877,486	AX-39732531
MYH6	rs77135504	chr14:23,851,199-23,877,486	AX-92392643
MYH6	rs77135504	chr14:23,851,199-23,877,486	AX-92603666
MYH6	rs76358702	chr14:23,851,199-23,877,486	AX-31312183
MYH6	rs7148253	chr14:23,851,199-23,877,486	AX-39732535
MYH6	rs7148648	chr14:23,851,199-23,877,486	AX-11610120
MYH6	rs7153499	chr14:23,851,199-23,877,486	AX-39732537
MYH6	rs7147244	chr14:23,851,199-23,877,486	AX-39732539
MYH6	rs28711516	chr14:23,851,199-23,877,486	AX-11422655
MYH6	---	chr14:23,851,199-23,877,486	AX-90056644
MYH6	rs28730780	chr14:23,851,199-23,877,486	AX-31312199
MYH6	rs58008413	chr14:23,851,199-23,877,486	AX-84745521
MYH6	rs58008413	chr14:23,851,199-23,877,486	AX-84745522
MYH7	---	chr14:23,881,947-23,904,870	AX-90048898
MYH7	rs2284651	chr14:23,881,947-23,904,870	AX-39732559
MYH7	rs7149517	chr14:23,881,947-23,904,870	AX-123053734
MYH7	rs7149517	chr14:23,881,947-23,904,870	AX-123053735

MYH7	rs12882128	chr14:23,881,947-23,904,870	AX-50150993
MYH7	rs7145543	chr14:23,881,947-23,904,870	AX-148258897
MYH7	rs7145543	chr14:23,881,947-23,904,870	AX-148678562
MYH7	rs7145023	chr14:23,881,947-23,904,870	AX-148421872
MYH7	rs7145023	chr14:23,881,947-23,904,870	AX-156280248
MYH7	rs2331979	chr14:23,881,947-23,904,870	AX-39732567
MYH7	---	chr14:23,881,947-23,904,870	AX-90025906
MYH7	---	chr14:23,881,947-23,904,870	AX-90025907
MYH7	rs397516254	chr14:23,881,947-23,904,870	AX-90033043
MYH7	rs200728597	chr14:23,881,947-23,904,870	AX-91989501
MYH7	---	chr14:23,881,947-23,904,870	AX-94379421
MYH7	rs45523233	chr14:23,881,947-23,904,870	AX-91996517
MYH7	---	chr14:23,881,947-23,904,870	AX-94362673
MYH7	rs3729833	chr14:23,881,947-23,904,870	AX-39732569
MYH7	rs121913652	chr14:23,881,947-23,904,870	AX-90069769
MYH7	rs376668612	chr14:23,881,947-23,904,870	AX-92013743
MYH7	rs368706722	chr14:23,881,947-23,904,870	AX-91980766
MYH7	rs765021	chr14:23,881,947-23,904,870	AX-31312261
MYH7	rs765020	chr14:23,881,947-23,904,870	AX-31312263
MYH7	rs58290801	chr14:23,881,947-23,904,870	AX-31312265
MYH7	rs765019	chr14:23,881,947-23,904,870	AX-31312267
MYH7	rs3729832	chr14:23,881,947-23,904,870	AX-39732573
MYH7	---	chr14:23,881,947-23,904,870	AX-94362676
MYH7	---	chr14:23,881,947-23,904,870	AX-94379427
MYH7	---	chr14:23,881,947-23,904,870	AX-91996825
MYH7	---	chr14:23,881,947-23,904,870	AX-90025909
MYH7	---	chr14:23,881,947-23,904,870	AX-94379428
MYH7	---	chr14:23,881,947-23,904,870	AX-90025910
MYH7	rs3729831	chr14:23,881,947-23,904,870	AX-92002128
MYH7	---	chr14:23,881,947-23,904,870	AX-90067763
MYH7	rs368848344	chr14:23,881,947-23,904,870	AX-92025059
MYH7	---	chr14:23,881,947-23,904,870	AX-94379429
MYH7	---	chr14:23,881,947-23,904,870	AX-90031615
MYH7	---	chr14:23,881,947-23,904,870	AX-94379430
MYH7	---	chr14:23,881,947-23,904,870	AX-90062449
MYH7	rs121913654	chr14:23,881,947-23,904,870	AX-90069770
MYH7	---	chr14:23,881,947-23,904,870	AX-90031784
MYH7	rs201171029	chr14:23,881,947-23,904,870	AX-92010787
MYH7	---	chr14:23,881,947-23,904,870	AX-90077185
MYH7	rs200939753	chr14:23,881,947-23,904,870	AX-88753563
MYH7	---	chr14:23,881,947-23,904,870	AX-90054796
MYH7	rs139222507	chr14:23,881,947-23,904,870	AX-88796575
MYH7	rs397516241	chr14:23,881,947-23,904,870	AX-90054797
MYH7	rs267606909	chr14:23,881,947-23,904,870	AX-90054798
MYH7	---	chr14:23,881,947-23,904,870	AX-94379431
MYH7	rs45582836	chr14:23,881,947-23,904,870	AX-91989866
MYH7	---	chr14:23,881,947-23,904,870	AX-90062165
MYH7	---	chr14:23,881,947-23,904,870	AX-90025914
MYH7	rs545585809	chr14:23,881,947-23,904,870	AX-90025915
MYH7	---	chr14:23,881,947-23,904,870	AX-90029395
MYH7	---	chr14:23,881,947-23,904,870	AX-94362682
MYH7	rs367543052	chr14:23,881,947-23,904,870	AX-90078512
MYH7	---	chr14:23,881,947-23,904,870	AX-94362683
MYH7	rs193922390	chr14:23,881,947-23,904,870	AX-86894418
MYH7	rs121913650	chr14:23,881,947-23,904,870	AX-88796562

MYH7	---	chr14:23,881,947-23,904,870	AX-90066045
MYH7	rs3729830	chr14:23,881,947-23,904,870	AX-92023968
MYH7	rs3729830	chr14:23,881,947-23,904,870	AX-92385436
MYH7	rs373219734	chr14:23,881,947-23,904,870	AX-92708497
MYH7	rs45464193	chr14:23,881,947-23,904,870	AX-88778788
MYH7	---	chr14:23,881,947-23,904,870	AX-86652175
MYH7	---	chr14:23,881,947-23,904,870	AX-94364182
MYH7	---	chr14:23,881,947-23,904,870	AX-94362684
MYH7	---	chr14:23,881,947-23,904,870	AX-90057814
MYH7	rs370328209	chr14:23,881,947-23,904,870	AX-90058980
MYH7	---	chr14:23,881,947-23,904,870	AX-91993802
MYH7	---	chr14:23,881,947-23,904,870	AX-90036360
MYH7	rs397516233	chr14:23,881,947-23,904,870	AX-90054801
MYH7	rs141122361	chr14:23,881,947-23,904,870	AX-86611677
MYH7	---	chr14:23,881,947-23,904,870	AX-94356300
MYH7	---	chr14:23,881,947-23,904,870	AX-94356301
MYH7	rs373514686	chr14:23,881,947-23,904,870	AX-91976277
MYH7	---	chr14:23,881,947-23,904,870	AX-90036400
MYH7	rs45476496	chr14:23,881,947-23,904,870	AX-91984829
MYH7	---	chr14:23,881,947-23,904,870	AX-90033363
MYH7	rs7140196	chr14:23,881,947-23,904,870	AX-88778790
MYH7	---	chr14:23,881,947-23,904,870	AX-94373052
MYH7	rs200601164	chr14:23,881,947-23,904,870	AX-91984042
MYH7	---	chr14:23,881,947-23,904,870	AX-94363355
MYH7	---	chr14:23,881,947-23,904,870	AX-90025918
MYH7	---	chr14:23,881,947-23,904,870	AX-94356303
MYH7	rs7140721	chr14:23,881,947-23,904,870	AX-156271314
MYH7	rs7140721	chr14:23,881,947-23,904,870	AX-156289838
MYH7	rs76614781	chr14:23,881,947-23,904,870	AX-31312287
MYH7	rs3729829	chr14:23,881,947-23,904,870	AX-11476404
MYH7	rs3729828	chr14:23,881,947-23,904,870	AX-156271315
MYH7	rs3729828	chr14:23,881,947-23,904,870	AX-156289839
MYH7	rs3729827	chr14:23,881,947-23,904,870	AX-39732585
MYH7	---	chr14:23,881,947-23,904,870	AX-94356304
MYH7	---	chr14:23,881,947-23,904,870	AX-90037308
MYH7	rs2754155	chr14:23,881,947-23,904,870	AX-91985388
MYH7	rs397516222	chr14:23,881,947-23,904,870	AX-90025919
MYH7	rs397516220	chr14:23,881,947-23,904,870	AX-90077347
MYH7	---	chr14:23,881,947-23,904,870	AX-94373055
MYH7	rs45503601	chr14:23,881,947-23,904,870	AX-92014335
MYH7	rs3729825	chr14:23,881,947-23,904,870	AX-39732587
MYH7	---	chr14:23,881,947-23,904,870	AX-90062719
MYH7	---	chr14:23,881,947-23,904,870	AX-90055831
MYH7	rs45544633	chr14:23,881,947-23,904,870	AX-88778791
MYH7	rs397516218	chr14:23,881,947-23,904,870	AX-90054805
MYH7	rs3729823	chr14:23,881,947-23,904,870	AX-39732589
MYH7	---	chr14:23,881,947-23,904,870	AX-90054806
MYH7	---	chr14:23,881,947-23,904,870	AX-94373056
MYH7	rs139646545	chr14:23,881,947-23,904,870	AX-83357911
MYH7	---	chr14:23,881,947-23,904,870	AX-94373057
MYH7	rs578166720	chr14:23,881,947-23,904,870	AX-91993119
MYH7	rs397516214	chr14:23,881,947-23,904,870	AX-91980477
MYH7	---	chr14:23,881,947-23,904,870	AX-90035198
MYH7	---	chr14:23,881,947-23,904,870	AX-90054808
MYH7	---	chr14:23,881,947-23,904,870	AX-94356308

MYH7	rs368078397	chr14:23,881,947-23,904,870	AX-92016277
MYH7	rs3729822	chr14:23,881,947-23,904,870	AX-92013029
MYH7	rs397516211	chr14:23,881,947-23,904,870	AX-90058918
MYH7	---	chr14:23,881,947-23,904,870	AX-90064591
MYH7	rs397516208	chr14:23,881,947-23,904,870	AX-90025924
MYH7	rs397516207	chr14:23,881,947-23,904,870	AX-90030768
MYH7	rs145213771	chr14:23,881,947-23,904,870	AX-86580629
MYH7	---	chr14:23,881,947-23,904,870	AX-94356309
MYH7	rs201895208	chr14:23,881,947-23,904,870	AX-88796622
MYH7	---	chr14:23,881,947-23,904,870	AX-90025925
MYH7	rs397516202	chr14:23,881,947-23,904,870	AX-86894494
MYH7	---	chr14:23,881,947-23,904,870	AX-94356310
MYH7	---	chr14:23,881,947-23,904,870	AX-94373061
MYH7	---	chr14:23,881,947-23,904,870	AX-94373062
MYH7	rs397516201	chr14:23,881,947-23,904,870	AX-90025926
MYH7	---	chr14:23,881,947-23,904,870	AX-92008545
MYH7	---	chr14:23,881,947-23,904,870	AX-94373063
MYH7	rs45451303	chr14:23,881,947-23,904,870	AX-84691226
MYH7	---	chr14:23,881,947-23,904,870	AX-90025927
MYH7	rs370403289	chr14:23,881,947-23,904,870	AX-86693884
MYH7	---	chr14:23,881,947-23,904,870	AX-90078284
MYH7	---	chr14:23,881,947-23,904,870	AX-94356314
MYH7	---	chr14:23,881,947-23,904,870	AX-90057071
MYH7	---	chr14:23,881,947-23,904,870	AX-94373065
MYH7	---	chr14:23,881,947-23,904,870	AX-92012367
MYH7	rs397516198	chr14:23,881,947-23,904,870	AX-90035422
MYH7	---	chr14:23,881,947-23,904,870	AX-92544772
MYH7	rs7159367	chr14:23,881,947-23,904,870	AX-84715599
MYH7	rs7159367	chr14:23,881,947-23,904,870	AX-84715600
MYH7	rs10136106	chr14:23,881,947-23,904,870	AX-84683129
MYH7	rs12894524	chr14:23,881,947-23,904,870	AX-84717413
MYH7	rs12894524	chr14:23,881,947-23,904,870	AX-84717414
MYH7	rs28631169	chr14:23,881,947-23,904,870	AX-31312311
MYH7	rs34598192	chr14:23,881,947-23,904,870	AX-84690381
MYH7	rs34598192	chr14:23,881,947-23,904,870	AX-84727267
MYH7	rs144420313	chr14:23,881,947-23,904,870	AX-92024004
MYH7	---	chr14:23,881,947-23,904,870	AX-90025928
MYH7	rs45501694	chr14:23,881,947-23,904,870	AX-31312315
MYH7	rs150292548	chr14:23,881,947-23,904,870	AX-91977945
MYH7	rs397516196	chr14:23,881,947-23,904,870	AX-90064407
MYH7	rs371335299	chr14:23,881,947-23,904,870	AX-91986743
MYH7	rs2277475	chr14:23,881,947-23,904,870	AX-39732599
MYH7	---	chr14:23,881,947-23,904,870	AX-86604297
MYH7	---	chr14:23,881,947-23,904,870	AX-94373068
MYH7	---	chr14:23,881,947-23,904,870	AX-94373069
MYH7	---	chr14:23,881,947-23,904,870	AX-90054814
MYH7	rs377745688	chr14:23,881,947-23,904,870	AX-92020956
MYH7	---	chr14:23,881,947-23,904,870	AX-94360278
MYH7	rs370750044	chr14:23,881,947-23,904,870	AX-92021926
MYH7	---	chr14:23,881,947-23,904,870	AX-90027952
MYH7	rs397516190	chr14:23,881,947-23,904,870	AX-90051860
MYH7	---	chr14:23,881,947-23,904,870	AX-94362685
MYH7	---	chr14:23,881,947-23,904,870	AX-90025930
MYH7	rs397516182	chr14:23,881,947-23,904,870	AX-92000066
MYH7	---	chr14:23,881,947-23,904,870	AX-94362686

MYH7	---	chr14:23,881,947-23,904,870	AX-90025931
MYH7	---	chr14:23,881,947-23,904,870	AX-94362687
MYH7	rs397516187	chr14:23,881,947-23,904,870	AX-90035849
MYH7	---	chr14:23,881,947-23,904,870	AX-90025932
MYH7	---	chr14:23,881,947-23,904,870	AX-94379438
MYH7	---	chr14:23,881,947-23,904,870	AX-94362689
MYH7	---	chr14:23,881,947-23,904,870	AX-94379440
MYH7	---	chr14:23,881,947-23,904,870	AX-94362691
MYH7	rs397516186	chr14:23,881,947-23,904,870	AX-92000700
MYH7	---	chr14:23,881,947-23,904,870	AX-94379442
MYH7	---	chr14:23,881,947-23,904,870	AX-90054818
MYH7	---	chr14:23,881,947-23,904,870	AX-90054819
MYH7	---	chr14:23,881,947-23,904,870	AX-94362693
MYH7	rs564923630	chr14:23,881,947-23,904,870	AX-83360089
MYH7	---	chr14:23,881,947-23,904,870	AX-94362694
MYH7	---	chr14:23,881,947-23,904,870	AX-90078119
MYH7	rs367546859	chr14:23,881,947-23,904,870	AX-90033653
MYH7	---	chr14:23,881,947-23,904,870	AX-94379445
MYH7	rs12147533	chr14:23,881,947-23,904,870	AX-39732601
MYH7	rs743567	chr14:23,881,947-23,904,870	AX-12725262
MYH7	rs17092021	chr14:23,881,947-23,904,870	AX-39732603
MYH7	---	chr14:23,881,947-23,904,870	AX-94362696
MYH7	---	chr14:23,881,947-23,904,870	AX-94380932
MYH7	---	chr14:23,881,947-23,904,870	AX-90025935
MYH7	---	chr14:23,881,947-23,904,870	AX-94379447
MYH7	---	chr14:23,881,947-23,904,870	AX-90054821
MYH7	rs397516179	chr14:23,881,947-23,904,870	AX-90025937
MYH7	---	chr14:23,881,947-23,904,870	AX-86556573
MYH7	rs138294643	chr14:23,881,947-23,904,870	AX-91978035
MYH7	---	chr14:23,881,947-23,904,870	AX-90032508
MYH7	---	chr14:23,881,947-23,904,870	AX-86632466
MYH7	rs45611033	chr14:23,881,947-23,904,870	AX-84775859
MYH7	---	chr14:23,881,947-23,904,870	AX-90025938
MYH7	rs551897533	chr14:23,881,947-23,904,870	AX-90054824
MYH7	rs200743586	chr14:23,881,947-23,904,870	AX-156285851
MYH7	rs200743586	chr14:23,881,947-23,904,870	AX-84738745
MYH7	rs8019322	chr14:23,881,947-23,904,870	AX-156271316
MYH7	rs8019322	chr14:23,881,947-23,904,870	AX-156289840
MYH7	---	chr14:23,881,947-23,904,870	AX-90058737
MYH7	---	chr14:23,881,947-23,904,870	AX-90054825
MYH7	---	chr14:23,881,947-23,904,870	AX-94379448
MYH7	rs145379951	chr14:23,881,947-23,904,870	AX-92007819
MYH7	rs71413987	chr14:23,881,947-23,904,870	AX-88796203
MYH7	rs7157716	chr14:23,881,947-23,904,870	AX-92409255
MYH7	rs7157716	chr14:23,881,947-23,904,870	AX-92620278
MYH7	rs145532615	chr14:23,881,947-23,904,870	AX-82939071
MYH7	rs7157087	chr14:23,881,947-23,904,870	AX-12622736
MYH7	rs7155989	chr14:23,881,947-23,904,870	AX-39732607
MYH7	---	chr14:23,881,947-23,904,870	AX-94379449
MYH7	---	chr14:23,881,947-23,904,870	AX-94362700
MYH7	---	chr14:23,881,947-23,904,870	AX-90025941
MYH7	---	chr14:23,881,947-23,904,870	AX-83160331
MYH7	---	chr14:23,881,947-23,904,870	AX-94379452
MYH7	---	chr14:23,881,947-23,904,870	AX-90025943
MYH7	---	chr14:23,881,947-23,904,870	AX-90025944

MYH7	rs121913629	chr14:23,881,947-23,904,870	AX-88796548
MYH7	rs121913639	chr14:23,881,947-23,904,870	AX-88753510
MYH7	---	chr14:23,881,947-23,904,870	AX-90054830
MYH7	rs397516172	chr14:23,881,947-23,904,870	AX-90074234
MYH7	---	chr14:23,881,947-23,904,870	AX-90025946
MYH7	rs397516171	chr14:23,881,947-23,904,870	AX-86894447
MYH7	---	chr14:23,881,947-23,904,870	AX-94362703
MYH7	---	chr14:23,881,947-23,904,870	AX-90044438
MYH7	---	chr14:23,881,947-23,904,870	AX-90054833
MYH7	rs397516170	chr14:23,881,947-23,904,870	AX-90054834
MYH7	rs575013686	chr14:23,881,947-23,904,870	AX-94362704
MYH7	rs121913628	chr14:23,881,947-23,904,870	AX-88753502
MYH7	---	chr14:23,881,947-23,904,870	AX-90054835
MYH7	---	chr14:23,881,947-23,904,870	AX-94362705
MYH7	---	chr14:23,881,947-23,904,870	AX-90034651
MYH7	rs397516168	chr14:23,881,947-23,904,870	AX-92014681
MYH7	rs121913631	chr14:23,881,947-23,904,870	AX-86894467
MYH7	rs267606908	chr14:23,881,947-23,904,870	AX-86894496
MYH7	---	chr14:23,881,947-23,904,870	AX-90054837
MYH7	rs397516165	chr14:23,881,947-23,904,870	AX-90028965
MYH7	---	chr14:23,881,947-23,904,870	AX-90025953
MYH7	---	chr14:23,881,947-23,904,870	AX-90054839
MYH7	---	chr14:23,881,947-23,904,870	AX-90054840
MYH7	---	chr14:23,881,947-23,904,870	AX-90054841
MYH7	---	chr14:23,881,947-23,904,870	AX-90025957
MYH7	rs397516161	chr14:23,881,947-23,904,870	AX-90025958
MYH7	rs45457293	chr14:23,881,947-23,904,870	AX-31312347
MYH7	rs10150821	chr14:23,881,947-23,904,870	AX-148362304
MYH7	rs10150821	chr14:23,881,947-23,904,870	AX-156271317
MYH7	rs368653983	chr14:23,881,947-23,904,870	AX-91990758
MYH7	---	chr14:23,881,947-23,904,870	AX-90031370
MYH7	---	chr14:23,881,947-23,904,870	AX-91994164
MYH7	---	chr14:23,881,947-23,904,870	AX-90025959
MYH7	---	chr14:23,881,947-23,904,870	AX-90050956
MYH7	---	chr14:23,881,947-23,904,870	AX-90054845
MYH7	---	chr14:23,881,947-23,904,870	AX-94357216
MYH7	---	chr14:23,881,947-23,904,870	AX-90054847
MYH7	rs397516159	chr14:23,881,947-23,904,870	AX-92005596
MYH7	rs36211715	chr14:23,881,947-23,904,870	AX-86894468
MYH7	---	chr14:23,881,947-23,904,870	AX-94357886
MYH7	rs138049878	chr14:23,881,947-23,904,870	AX-88753528
MYH7	rs202141173	chr14:23,881,947-23,904,870	AX-88746622
MYH7	---	chr14:23,881,947-23,904,870	AX-90028321
MYH7	---	chr14:23,881,947-23,904,870	AX-90054849
MYH7	rs558673680	chr14:23,881,947-23,904,870	AX-94357217
MYH7	---	chr14:23,881,947-23,904,870	AX-90032425
MYH7	---	chr14:23,881,947-23,904,870	AX-90033141
MYH7	---	chr14:23,881,947-23,904,870	AX-83469943
MYH7	---	chr14:23,881,947-23,904,870	AX-90054851
MYH7	---	chr14:23,881,947-23,904,870	AX-94357757
MYH7	---	chr14:23,881,947-23,904,870	AX-94374507
MYH7	rs397516157	chr14:23,881,947-23,904,870	AX-90054852
MYH7	---	chr14:23,881,947-23,904,870	AX-90054853
MYH7	---	chr14:23,881,947-23,904,870	AX-90054854
MYH7	rs397516156	chr14:23,881,947-23,904,870	AX-90059293

MYH7	---	chr14:23,881,947-23,904,870	AX-90025971
MYH7	---	chr14:23,881,947-23,904,870	AX-90058600
MYH7	---	chr14:23,881,947-23,904,870	AX-90055972
MYH7	rs397516153	chr14:23,881,947-23,904,870	AX-90025972
MYH7	---	chr14:23,881,947-23,904,870	AX-94373968
MYH7	---	chr14:23,881,947-23,904,870	AX-90025974
MYH7	---	chr14:23,881,947-23,904,870	AX-92022818
MYH7	---	chr14:23,881,947-23,904,870	AX-90054863
MYH7	---	chr14:23,881,947-23,904,870	AX-90054864
MYH7	rs11848507	chr14:23,881,947-23,904,870	AX-91987735
MYH7	---	chr14:23,881,947-23,904,870	AX-90025980
MYH7	---	chr14:23,881,947-23,904,870	AX-90063359
MYH7	rs376754645	chr14:23,881,947-23,904,870	AX-86711923
MYH7	rs145677314	chr14:23,881,947-23,904,870	AX-86554725
MYH7	---	chr14:23,881,947-23,904,870	AX-94373969
MYH7	rs139882431	chr14:23,881,947-23,904,870	AX-91997847
MYH7	---	chr14:23,881,947-23,904,870	AX-86579693
MYH7	---	chr14:23,881,947-23,904,870	AX-90025982
MYH7	---	chr14:23,881,947-23,904,870	AX-90054868
MYH7	---	chr14:23,881,947-23,904,870	AX-90025984
MYH7	---	chr14:23,881,947-23,904,870	AX-90025985
MYH7	---	chr14:23,881,947-23,904,870	AX-90034671
MYH7	rs121913634	chr14:23,881,947-23,904,870	AX-88796551
MYH7	---	chr14:23,881,947-23,904,870	AX-90054872
MYH7	---	chr14:23,881,947-23,904,870	AX-86894497
MYH7	---	chr14:23,881,947-23,904,870	AX-91994588
MYH7	rs121913643	chr14:23,881,947-23,904,870	AX-88753514
MYH7	---	chr14:23,881,947-23,904,870	AX-90025988
MYH7	---	chr14:23,881,947-23,904,870	AX-90054874
MYH7	rs2854797	chr14:23,881,947-23,904,870	AX-94363663
MYH7	---	chr14:23,881,947-23,904,870	AX-94357220
MYH7	---	chr14:23,881,947-23,904,870	AX-94379456
MYH7	---	chr14:23,881,947-23,904,870	AX-90025990
MYH7	---	chr14:23,881,947-23,904,870	AX-90054876
MYH7	---	chr14:23,881,947-23,904,870	AX-90054877
MYH7	---	chr14:23,881,947-23,904,870	AX-94357768
MYH7	---	chr14:23,881,947-23,904,870	AX-94374518
MYH7	---	chr14:23,881,947-23,904,870	AX-94379457
MYH7	---	chr14:23,881,947-23,904,870	AX-90054879
MYH7	---	chr14:23,881,947-23,904,870	AX-90054880
MYH7	---	chr14:23,881,947-23,904,870	AX-90054881
MYH7	---	chr14:23,881,947-23,904,870	AX-94377160
MYH7	---	chr14:23,881,947-23,904,870	AX-90025997
MYH7	---	chr14:23,881,947-23,904,870	AX-90025998
MYH7	---	chr14:23,881,947-23,904,870	AX-90054884
MYH7	---	chr14:23,881,947-23,904,870	AX-90027801
MYH7	---	chr14:23,881,947-23,904,870	AX-90026000
MYH7	---	chr14:23,881,947-23,904,870	AX-90054886
MYH7	---	chr14:23,881,947-23,904,870	AX-91981128
MYH7	---	chr14:23,881,947-23,904,870	AX-94362708
MYH7	rs397516135	chr14:23,881,947-23,904,870	AX-90026002
MYH7	---	chr14:23,881,947-23,904,870	AX-86894470
MYH7	rs3729818	chr14:23,881,947-23,904,870	AX-156271318
MYH7	rs3729818	chr14:23,881,947-23,904,870	AX-156289841
MYH7	---	chr14:23,881,947-23,904,870	AX-94362710

MYH7	rs121913641	chr14:23,881,947-23,904,870	AX-86894471
MYH7	rs121913637	chr14:23,881,947-23,904,870	AX-86894472
MYH7	rs121913638	chr14:23,881,947-23,904,870	AX-86894473
MYH7	---	chr14:23,881,947-23,904,870	AX-90026005
MYH7	---	chr14:23,881,947-23,904,870	AX-90026006
MYH7	rs3181426	chr14:23,881,947-23,904,870	AX-91991442
MYH7	---	chr14:23,881,947-23,904,870	AX-90029090
MYH7	---	chr14:23,881,947-23,904,870	AX-94362711
MYH7	---	chr14:23,881,947-23,904,870	AX-90026008
MYH7	rs28475411	chr14:23,881,947-23,904,870	AX-31312369
MYH7	---	chr14:23,881,947-23,904,870	AX-94371743
MYH7	---	chr14:23,881,947-23,904,870	AX-94360205
MYH7	---	chr14:23,881,947-23,904,870	AX-90026011
MYH7	rs371898076	chr14:23,881,947-23,904,870	AX-86661074
MYH7	---	chr14:23,881,947-23,904,870	AX-94360374
MYH7	---	chr14:23,881,947-23,904,870	AX-94377124
MYH7	rs146474860	chr14:23,881,947-23,904,870	AX-91981264
MYH7	---	chr14:23,881,947-23,904,870	AX-90056510
MYH7	---	chr14:23,881,947-23,904,870	AX-90026013
MYH7	---	chr14:23,881,947-23,904,870	AX-94360206
MYH7	---	chr14:23,881,947-23,904,870	AX-90054899
MYH7	---	chr14:23,881,947-23,904,870	AX-90054900
MYH7	rs121913646	chr14:23,881,947-23,904,870	AX-88796561
MYH7	---	chr14:23,881,947-23,904,870	AX-90054901
MYH7	---	chr14:23,881,947-23,904,870	AX-90063022
MYH7	---	chr14:23,881,947-23,904,870	AX-90026017
MYH7	rs541143322	chr14:23,881,947-23,904,870	AX-94360207
MYH7	---	chr14:23,881,947-23,904,870	AX-90026018
MYH7	---	chr14:23,881,947-23,904,870	AX-90026019
MYH7	---	chr14:23,881,947-23,904,870	AX-92011321
MYH7	---	chr14:23,881,947-23,904,870	AX-94360208
MYH7	---	chr14:23,881,947-23,904,870	AX-90058458
MYH7	rs121913627	chr14:23,881,947-23,904,870	AX-86894426
MYH7	---	chr14:23,881,947-23,904,870	AX-90054905
MYH7	---	chr14:23,881,947-23,904,870	AX-94360209
MYH7	---	chr14:23,881,947-23,904,870	AX-90035330
MYH7	---	chr14:23,881,947-23,904,870	AX-92016045
MYH7	---	chr14:23,881,947-23,904,870	AX-90057200
MYH7	rs3729816	chr14:23,881,947-23,904,870	AX-39732613
MYH7	---	chr14:23,881,947-23,904,870	AX-90026022
MYH7	---	chr14:23,881,947-23,904,870	AX-92002487
MYH7	rs397516121	chr14:23,881,947-23,904,870	AX-91985024
MYH7	rs201860580	chr14:23,881,947-23,904,870	AX-91980271
MYH7	---	chr14:23,881,947-23,904,870	AX-90054908
MYH7	---	chr14:23,881,947-23,904,870	AX-90026024
MYH7	---	chr14:23,881,947-23,904,870	AX-94376960
MYH7	---	chr14:23,881,947-23,904,870	AX-90035361
MYH7	---	chr14:23,881,947-23,904,870	AX-86575331
MYH7	rs149386750	chr14:23,881,947-23,904,870	AX-91977504
MYH7	---	chr14:23,881,947-23,904,870	AX-90026025
MYH7	rs564101364	chr14:23,881,947-23,904,870	AX-90026026
MYH7	---	chr14:23,881,947-23,904,870	AX-94376961
MYH7	---	chr14:23,881,947-23,904,870	AX-90027736
MYH7	---	chr14:23,881,947-23,904,870	AX-90054912
MYH7	rs2069543	chr14:23,881,947-23,904,870	AX-92483022

MYH7	rs2069543	chr14:23,881,947-23,904,870	AX-92694045
MYH7	rs121913642	chr14:23,881,947-23,904,870	AX-88796558
MYH7	---	chr14:23,881,947-23,904,870	AX-90054913
MYH7	---	chr14:23,881,947-23,904,870	AX-90064698
MYH7	rs182949516	chr14:23,881,947-23,904,870	AX-92020327
MYH7	rs1951154	chr14:23,881,947-23,904,870	AX-39732615
MYH7	rs2754163	chr14:23,881,947-23,904,870	AX-39732617
MYH7	---	chr14:23,881,947-23,904,870	AX-90065764
MYH7	---	chr14:23,881,947-23,904,870	AX-94376962
MYH7	---	chr14:23,881,947-23,904,870	AX-90054914
MYH7	---	chr14:23,881,947-23,904,870	AX-90064888
MYH7	---	chr14:23,881,947-23,904,870	AX-90026030
MYH7	rs121913636	chr14:23,881,947-23,904,870	AX-88796552
MYH7	rs397516110	chr14:23,881,947-23,904,870	AX-90026031
MYH7	---	chr14:23,881,947-23,904,870	AX-90026032
MYH7	rs3729813	chr14:23,881,947-23,904,870	AX-91993639
MYH7	---	chr14:23,881,947-23,904,870	AX-90026033
MYH7	---	chr14:23,881,947-23,904,870	AX-91984495
MYH7	---	chr14:23,881,947-23,904,870	AX-90026034
MYH7	rs3218715	chr14:23,881,947-23,904,870	AX-88778797
MYH7	rs267606911	chr14:23,881,947-23,904,870	AX-90054920
MYH7	---	chr14:23,881,947-23,904,870	AX-94360213
MYH7	---	chr14:23,881,947-23,904,870	AX-94374678
MYH7	rs121913651	chr14:23,881,947-23,904,870	AX-88753518
MYH7	---	chr14:23,881,947-23,904,870	AX-90026037
MYH7	---	chr14:23,881,947-23,904,870	AX-94360214
MYH7	---	chr14:23,881,947-23,904,870	AX-90028810
MYH7	---	chr14:23,881,947-23,904,870	AX-90056313
MYH7	rs3729814	chr14:23,881,947-23,904,870	AX-113725984
MYH7	rs3729814	chr14:23,881,947-23,904,870	AX-156266484
MYH7	---	chr14:23,881,947-23,904,870	AX-94376965
MYH7	rs397516106	chr14:23,881,947-23,904,870	AX-90060225
MYH7	---	chr14:23,881,947-23,904,870	AX-90064272
MYH7	---	chr14:23,881,947-23,904,870	AX-90055942
MYH7	rs4981473	chr14:23,881,947-23,904,870	AX-92015496
MYH7	rs45508293	chr14:23,881,947-23,904,870	AX-92010687
MYH7	rs397516105	chr14:23,881,947-23,904,870	AX-94360279
MYH7	rs397516103	chr14:23,881,947-23,904,870	AX-90035303
MYH7	rs397516101	chr14:23,881,947-23,904,870	AX-90026038
MYH7	---	chr14:23,881,947-23,904,870	AX-94357309
MYH7	---	chr14:23,881,947-23,904,870	AX-94374059
MYH7	---	chr14:23,881,947-23,904,870	AX-90054925
MYH7	---	chr14:23,881,947-23,904,870	AX-90054926
MYH7	---	chr14:23,881,947-23,904,870	AX-94362712
MYH7	---	chr14:23,881,947-23,904,870	AX-88735753
MYH7	---	chr14:23,881,947-23,904,870	AX-90026043
MYH7	rs148808089	chr14:23,881,947-23,904,870	AX-86560384
MYH7	rs121913653	chr14:23,881,947-23,904,870	AX-86663839
MYH7	rs397516098	chr14:23,881,947-23,904,870	AX-90054929
MYH7	---	chr14:23,881,947-23,904,870	AX-90054930
MYH7	---	chr14:23,881,947-23,904,870	AX-90026046
MYH7	---	chr14:23,881,947-23,904,870	AX-90026047
MYH7	---	chr14:23,881,947-23,904,870	AX-94379463
MYH7	rs397516097	chr14:23,881,947-23,904,870	AX-90054933
MYH7	---	chr14:23,881,947-23,904,870	AX-94362714

MYH7	rs141519877	chr14:23,881,947-23,904,870	AX-92018287
MYH7	---	chr14:23,881,947-23,904,870	AX-90054934
MYH7	---	chr14:23,881,947-23,904,870	AX-90026050
MYH7	rs150885220	chr14:23,881,947-23,904,870	AX-90083208
MYH7	rs397516095	chr14:23,881,947-23,904,870	AX-90026051
MYH7	---	chr14:23,881,947-23,904,870	AX-90054937
MYH7	---	chr14:23,881,947-23,904,870	AX-90026053
MYH7	---	chr14:23,881,947-23,904,870	AX-88753500
MYH7	rs3218714	chr14:23,881,947-23,904,870	AX-86894429
MYH7	---	chr14:23,881,947-23,904,870	AX-90026054
MYH7	rs139506719	chr14:23,881,947-23,904,870	AX-92015083
MYH7	---	chr14:23,881,947-23,904,870	AX-94379465
MYH7	---	chr14:23,881,947-23,904,870	AX-92001716
MYH7	---	chr14:23,881,947-23,904,870	AX-90034050
MYH7	---	chr14:23,881,947-23,904,870	AX-90026055
MYH7	---	chr14:23,881,947-23,904,870	AX-90036361
MYH7	---	chr14:23,881,947-23,904,870	AX-90026057
MYH7	---	chr14:23,881,947-23,904,870	AX-90054943
MYH7	---	chr14:23,881,947-23,904,870	AX-90026059
MYH7	---	chr14:23,881,947-23,904,870	AX-90054945
MYH7	---	chr14:23,881,947-23,904,870	AX-94362716
MYH7	---	chr14:23,881,947-23,904,870	AX-90026061
MYH7	---	chr14:23,881,947-23,904,870	AX-90062778
MYH7	rs2231126	chr14:23,881,947-23,904,870	AX-91985266
MYH7	rs2231126	chr14:23,881,947-23,904,870	AX-92010393
MYH7	---	chr14:23,881,947-23,904,870	AX-94379467
MYH7	rs397516089	chr14:23,881,947-23,904,870	AX-90026062
MYH7	rs735711	chr14:23,881,947-23,904,870	AX-91976699
MYH7	rs735711	chr14:23,881,947-23,904,870	AX-92001826
MYH7	rs397516088	chr14:23,881,947-23,904,870	AX-90026063
MYH7	rs735712	chr14:23,881,947-23,904,870	AX-11623071
MYH7	---	chr14:23,881,947-23,904,870	AX-90026064
MYH7	---	chr14:23,881,947-23,904,870	AX-90060265
MYH7	rs121913640	chr14:23,881,947-23,904,870	AX-88753511
MYH7	---	chr14:23,881,947-23,904,870	AX-94362718
MYH7	---	chr14:23,881,947-23,904,870	AX-92001154
MYH7	rs202199570	chr14:23,881,947-23,904,870	AX-92022693
MYH7	rs3729810	chr14:23,881,947-23,904,870	AX-92553889
MYH7	---	chr14:23,881,947-23,904,870	AX-90026065
MYH7	rs372731424	chr14:23,881,947-23,904,870	AX-86733324
MYH7	rs2231124	chr14:23,881,947-23,904,870	AX-31312405
MYH7	---	chr14:23,881,947-23,904,870	AX-94362719
MYH7	---	chr14:23,881,947-23,904,870	AX-90059888
MYH7	---	chr14:23,881,947-23,904,870	AX-94362720
MYH7	rs376897125	chr14:23,881,947-23,904,870	AX-86735700
MYH7	---	chr14:23,881,947-23,904,870	AX-90026066
MYH7	---	chr14:23,881,947-23,904,870	AX-94362721
MYH7	---	chr14:23,881,947-23,904,870	AX-94379472
MYH7	---	chr14:23,881,947-23,904,870	AX-90026067
MYH7	rs45580436	chr14:23,881,947-23,904,870	AX-39732627
MYH7	rs397516272	chr14:23,881,947-23,904,870	AX-90033718
MYH7	---	chr14:23,881,947-23,904,870	AX-90026068
MYH7	---	chr14:23,881,947-23,904,870	AX-90026069
MYH7	---	chr14:23,881,947-23,904,870	AX-92020270
MYH7	---	chr14:23,881,947-23,904,870	AX-90026070

MYH7	rs372245426	chr14:23,881,947-23,904,870	AX-83540097
MYH7	---	chr14:23,881,947-23,904,870	AX-94362723
MYH7	---	chr14:23,881,947-23,904,870	AX-90026071
MYH7	rs397516269	chr14:23,881,947-23,904,870	AX-90054957
MYH7	---	chr14:23,881,947-23,904,870	AX-90054958
MYH7	rs121913633	chr14:23,881,947-23,904,870	AX-88796550
MYH7	---	chr14:23,881,947-23,904,870	AX-94362724
MYH7	---	chr14:23,881,947-23,904,870	AX-90054959
MYH7	rs397516268	chr14:23,881,947-23,904,870	AX-91993699
MYH7	---	chr14:23,881,947-23,904,870	AX-90054961
MYH7	---	chr14:23,881,947-23,904,870	AX-94379475
MYH7	---	chr14:23,881,947-23,904,870	AX-90026077
MYH7	---	chr14:23,881,947-23,904,870	AX-90057428
MYH7	---	chr14:23,881,947-23,904,870	AX-90035918
MYH7	rs2069542	chr14:23,881,947-23,904,870	AX-83279322
MYH7	---	chr14:23,881,947-23,904,870	AX-90054963
MYH7	rs267606910	chr14:23,881,947-23,904,870	AX-90054964
MYH7	rs397516265	chr14:23,881,947-23,904,870	AX-90056339
MYH7	rs397516264	chr14:23,881,947-23,904,870	AX-90054965
MYH7	rs397516263	chr14:23,881,947-23,904,870	AX-92025001
MYH7	---	chr14:23,881,947-23,904,870	AX-90026081
MYH7	---	chr14:23,881,947-23,904,870	AX-90054967
MYH7	---	chr14:23,881,947-23,904,870	AX-94362726
MYH7	---	chr14:23,881,947-23,904,870	AX-90054968
MYH7	---	chr14:23,881,947-23,904,870	AX-94362727
MYH7	rs121913645	chr14:23,881,947-23,904,870	AX-88753515
MYH7	---	chr14:23,881,947-23,904,870	AX-90054971
MYH7	---	chr14:23,881,947-23,904,870	AX-90054972
MYH7	---	chr14:23,881,947-23,904,870	AX-90026088
MYH7	---	chr14:23,881,947-23,904,870	AX-90026089
MYH7	---	chr14:23,881,947-23,904,870	AX-90026090
MYH7	rs397516260	chr14:23,881,947-23,904,870	AX-90026091
MYH7	---	chr14:23,881,947-23,904,870	AX-90052516
MYH7	rs397516258	chr14:23,881,947-23,904,870	AX-90054977
MYH7	rs2069541	chr14:23,881,947-23,904,870	AX-39732631
MYH7	---	chr14:23,881,947-23,904,870	AX-94379478
MYH7	---	chr14:23,881,947-23,904,870	AX-90054978
MYH7	---	chr14:23,881,947-23,904,870	AX-90054979
MYH7	---	chr14:23,881,947-23,904,870	AX-94379479
MYH7	---	chr14:23,881,947-23,904,870	AX-90054980
MYH7	---	chr14:23,881,947-23,904,870	AX-90026096
MYH7	---	chr14:23,881,947-23,904,870	AX-90026097
MYH7	rs369490861	chr14:23,881,947-23,904,870	AX-94362730
MYH7	---	chr14:23,881,947-23,904,870	AX-90035360
MYH7	rs201586880	chr14:23,881,947-23,904,870	AX-92002863
MYH7	rs7151241	chr14:23,881,947-23,904,870	AX-39732633
MYH7	rs73587634	chr14:23,881,947-23,904,870	AX-31312413
MYH7	rs28615777	chr14:23,881,947-23,904,870	AX-31312415
MYH7	rs114763452	chr14:23,881,947-23,904,870	AX-12725269
MYH7	rs2754165	chr14:23,881,947-23,904,870	AX-31312417
MYH7	---	chr14:23,881,947-23,904,870	AX-90054984
MYH7	---	chr14:23,881,947-23,904,870	AX-94379481
MYH7	---	chr14:23,881,947-23,904,870	AX-94362732
MYH7	---	chr14:23,881,947-23,904,870	AX-90026100
MYH7	rs45500700	chr14:23,881,947-23,904,870	AX-91988761

MYH7	---	chr14:23,881,947-23,904,870	AX-86711389
MYH7	rs397516212	chr14:23,881,947-23,904,870	AX-90026101
MYH7	---	chr14:23,881,947-23,904,870	AX-94362733
MYH7	rs397516209	chr14:23,881,947-23,904,870	AX-90026102
MYH7	---	chr14:23,881,947-23,904,870	AX-86561595
MYH7	---	chr14:23,881,947-23,904,870	AX-90065040
MYH7	---	chr14:23,881,947-23,904,870	AX-92024325
MYH7	rs3729806	chr14:23,881,947-23,904,870	AX-91982535
MYH7	rs146744056	chr14:23,881,947-23,904,870	AX-91985213
MYH7	rs149439730	chr14:23,881,947-23,904,870	AX-83533128
MYH7	rs397516183	chr14:23,881,947-23,904,870	AX-90026105
MYH7	rs36211408	chr14:23,881,947-23,904,870	AX-91978287
MYH7	rs2754166	chr14:23,881,947-23,904,870	AX-92006604
MYH7	rs140245862	chr14:23,881,947-23,904,870	AX-92023247
MYH7	rs148560996	chr14:23,881,947-23,904,870	AX-92022139
MYH7	---	chr14:23,881,947-23,904,870	AX-90029717
MYH7	rs3729995	chr14:23,881,947-23,904,870	AX-39732635
MYH7	rs2069540	chr14:23,881,947-23,904,870	AX-11365175
MYH7	---	chr14:23,881,947-23,904,870	AX-90054991
MYH7	---	chr14:23,881,947-23,904,870	AX-90054992
MYH7	rs373145667	chr14:23,881,947-23,904,870	AX-92020462
MYH7	---	chr14:23,881,947-23,904,870	AX-94362734
MYH7	rs376160714	chr14:23,881,947-23,904,870	AX-86644162
MYH7	---	chr14:23,881,947-23,904,870	AX-94379485
MYH7	rs186964570	chr14:23,881,947-23,904,870	AX-83255916
MYH7	---	chr14:23,881,947-23,904,870	AX-90054993
MYH7	---	chr14:23,881,947-23,904,870	AX-90081042
MYH7	---	chr14:23,881,947-23,904,870	AX-90054994
MYH7	rs3729993	chr14:23,881,947-23,904,870	AX-91997045
MYH7	rs2239578	chr14:23,881,947-23,904,870	AX-39732641
MYH7	rs45497293	chr14:23,881,947-23,904,870	AX-11510316
MYH7	rs2239577	chr14:23,881,947-23,904,870	AX-114182383
MYH7	rs2239577	chr14:23,881,947-23,904,870	AX-156266485
MYH7	rs17092326	chr14:23,881,947-23,904,870	AX-12490683
MYL2	rs199474815	chr12:111,348,624-111,358,404	AX-88795860
MYL2	rs199474813	chr12:111,348,624-111,358,404	AX-90069480
MYL2	rs12231049	chr12:111,348,624-111,358,404	AX-11197141
MYL2	rs143139258	chr12:111,348,624-111,358,404	AX-83216594
MYL2	rs199474811	chr12:111,348,624-111,358,404	AX-88795858
MYL2	rs121913658	chr12:111,348,624-111,358,404	AX-88795839
MYL2	rs11065769	chr12:111,348,624-111,358,404	AX-39288531
MYL2	rs104894369	chr12:111,348,624-111,358,404	AX-86894481
MYL2	rs199474809	chr12:111,348,624-111,358,404	AX-90069478
MYL2	rs933296	chr12:111,348,624-111,358,404	AX-39288539
MYL2	---	chr12:111,348,624-111,358,404	AX-83550653
MYL2	rs104894368	chr12:111,348,624-111,358,404	AX-88789537
MYL2	rs199474816	chr12:111,348,624-111,358,404	AX-88795861
MYL2	rs104894370	chr12:111,348,624-111,358,404	AX-88795781
MYL2	rs104894363	chr12:111,348,624-111,358,404	AX-83324468
MYL2	rs111525522	chr12:111,348,624-111,358,404	AX-16825437
MYL2	rs2233258	chr12:111,348,624-111,358,404	AX-39288565
MYL3	rs199474708	chr3:46,899,357-46,904,973	AX-90042039
MYL3	rs111617556	chr3:46,899,357-46,904,973	AX-90039927
MYL3	rs199474707	chr3:46,899,357-46,904,973	AX-86701846
MYL3	rs199474706	chr3:46,899,357-46,904,973	AX-88798925

MYL3	rs104893749	chr3:46,899,357-46,904,973	AX-86725846
MYL3	rs199474705	chr3:46,899,357-46,904,973	AX-88798924
MYL3	rs104893748	chr3:46,899,357-46,904,973	AX-88755648
MYL3	rs104893750	chr3:46,899,357-46,904,973	AX-88755649
MYL3	rs199474704	chr3:46,899,357-46,904,973	AX-90042036
MYL3	rs199474703	chr3:46,899,357-46,904,973	AX-88755878
MYL3	rs150634297	chr3:46,899,357-46,904,973	AX-90068613
MYL3	rs199474702	chr3:46,899,357-46,904,973	AX-88755877
MYL3	rs3792558	chr3:46,899,357-46,904,973	AX-41242637
MYLK2	rs117502839	chr20:30,407,178-30,422,500	AX-83341098
MYLK2	rs192056427	chr20:30,407,178-30,422,500	AX-83462248
MYLK2	rs121908108	chr20:30,407,178-30,422,500	AX-83421474
MYLK2	rs34396614	chr20:30,407,178-30,422,500	AX-83576264
MYLK2	rs4911532	chr20:30,407,178-30,422,500	AX-40526407
MYLK2	rs6119728	chr20:30,407,178-30,422,500	AX-32908913
MYLK2	rs17340492	chr20:30,407,178-30,422,500	AX-50272095
MYLK2	rs6089088	chr20:30,407,178-30,422,500	AX-11559009
MYOZ2	rs72673744	chr4:120,056,939-120,108,944	AX-34531665
MYOZ2	rs115956126	chr4:120,056,939-120,108,944	AX-14462122
MYOZ2	rs7655957	chr4:120,056,939-120,108,944	AX-14462162
MYOZ2	rs7668809	chr4:120,056,939-120,108,944	AX-12636181
MYOZ2	rs56034803	chr4:120,056,939-120,108,944	AX-34531759
MYOZ2	rs140126678	chr4:120,056,939-120,108,944	AX-83038636
MYPN	rs80149416	chr10:69,865,874-69,971,773	AX-16387925
MYPN	rs10823141	chr10:69,865,874-69,971,773	AX-12397494
MYPN	rs140148105	chr10:69,865,874-69,971,773	AX-83065473
MYPN	rs71578989	chr10:69,865,874-69,971,773	AX-90082250
MYPN	rs199476401	chr10:69,865,874-69,971,773	AX-90040308
MYPN	rs199476402	chr10:69,865,874-69,971,773	AX-90040309
MYPN	---	chr10:69,865,874-69,971,773	AX-90069196
MYPN	rs74143022	chr10:69,865,874-69,971,773	AX-29690333
MYPN	rs146336815	chr10:69,865,874-69,971,773	AX-83414062
MYPN	rs17456902	chr10:69,865,874-69,971,773	AX-11324962
MYPN	rs10997938	chr10:69,865,874-69,971,773	AX-11137131
MYPN	rs12217951	chr10:69,865,874-69,971,773	AX-16387971
MYPN	rs74759173	chr10:69,865,874-69,971,773	AX-16387980
MYPN	rs35168680	chr10:69,865,874-69,971,773	AX-29690377
MYPN	rs77368905	chr10:69,865,874-69,971,773	AX-29690381
MYPN	rs11598201	chr10:69,865,874-69,971,773	AX-11161393
MYPN	rs11598219	chr10:69,865,874-69,971,773	AX-11161395
MYPN	rs16925142	chr10:69,865,874-69,971,773	AX-11290236
MYPN	rs76451279	chr10:69,865,874-69,971,773	AX-16387995
MYPN	rs199476404	chr10:69,865,874-69,971,773	AX-90040311
MYPN	rs28429181	chr10:69,865,874-69,971,773	AX-29690391
MYPN	rs10997944	chr10:69,865,874-69,971,773	AX-38864737
MYPN	rs2673798	chr10:69,865,874-69,971,773	AX-16388000
MYPN	rs2635994	chr10:69,865,874-69,971,773	AX-38864747
MYPN	rs147596628	chr10:69,865,874-69,971,773	AX-83395665
MYPN	rs2817760	chr10:69,865,874-69,971,773	AX-11412242
MYPN	rs17535935	chr10:69,865,874-69,971,773	AX-11328060
MYPN	rs11596653	chr10:69,865,874-69,971,773	AX-38864761
MYPN	rs199476406	chr10:69,865,874-69,971,773	AX-90040312
MYPN	rs2673801	chr10:69,865,874-69,971,773	AX-11404417
MYPN	rs2817759	chr10:69,865,874-69,971,773	AX-29690419
MYPN	rs10997948	chr10:69,865,874-69,971,773	AX-12403658

MYPN	rs72795411	chr10:69,865,874-69,971,773	AX-29690421
MYPN	rs61857532	chr10:69,865,874-69,971,773	AX-29690431
MYPN	rs71475229	chr10:69,865,874-69,971,773	AX-29690445
MYPN	rs2634707	chr10:69,865,874-69,971,773	AX-29690463
MYPN	rs2087344	chr10:69,865,874-69,971,773	AX-12516938
MYPN	rs199476408	chr10:69,865,874-69,971,773	AX-90040313
MYPN	rs2634711	chr10:69,865,874-69,971,773	AX-16388049
MYPN	rs199476409	chr10:69,865,874-69,971,773	AX-90069200
MYPN	---	chr10:69,865,874-69,971,773	AX-16388051
MYPN	rs10997968	chr10:69,865,874-69,971,773	AX-38864815
MYPN	rs75449519	chr10:69,865,874-69,971,773	AX-29690515
MYPN	---	chr10:69,865,874-69,971,773	AX-11137134
MYPN	rs7916821	chr10:69,865,874-69,971,773	AX-11653589
MYPN	rs3814182	chr10:69,865,874-69,971,773	AX-16388068
MYPN	rs62620248	chr10:69,865,874-69,971,773	AX-29690545
MYPN	rs199476410	chr10:69,865,874-69,971,773	AX-90069201
MYPN	rs7097776	chr10:69,865,874-69,971,773	AX-12620565
MYPN	rs199476411	chr10:69,865,874-69,971,773	AX-90040316
MYPN	rs199476412	chr10:69,865,874-69,971,773	AX-86677240
MYPN	rs17458319	chr10:69,865,874-69,971,773	AX-38864839
MYPN	rs3998872	chr10:69,865,874-69,971,773	AX-29690577
MYPN	rs116403189	chr10:69,865,874-69,971,773	AX-29690585
MYPN	rs74769472	chr10:69,865,874-69,971,773	AX-16388092
MYPN	rs199476413	chr10:69,865,874-69,971,773	AX-90040317
MYPN	rs149887823	chr10:69,865,874-69,971,773	AX-83063544
MYPN	rs151282801	chr10:69,865,874-69,971,773	AX-83410279
MYPN	rs71584501	chr10:69,865,874-69,971,773	AX-88795483
MYPN	rs71534278	chr10:69,865,874-69,971,773	AX-83391674
MYPN	rs7079481	chr10:69,865,874-69,971,773	AX-11605365
MYPN	rs138313730	chr10:69,865,874-69,971,773	AX-82907228
MYPN	---	chr10:69,865,874-69,971,773	AX-83025392
MYPN	rs12358805	chr10:69,865,874-69,971,773	AX-16388129
MYPN	rs71534280	chr10:69,865,874-69,971,773	AX-83304968
MYPN	rs199476416	chr10:69,865,874-69,971,773	AX-90040318
MYPN	rs6480314	chr10:69,865,874-69,971,773	AX-11567069
MYPN	rs10998021	chr10:69,865,874-69,971,773	AX-16388152
MYPN	rs142877365	chr10:69,865,874-69,971,773	AX-83190637
MYPN	rs71541556	chr10:69,865,874-69,971,773	AX-29690683
NEBL	rs2296613	chr10:21,068,903-21,186,531	AX-15489990
NEBL	rs202184399	chr10:21,068,903-21,186,531	AX-83277406
NEBL	rs45543632	chr10:21,068,903-21,186,531	AX-60549343
NEBL	---	chr10:21,068,903-21,186,531	AX-90034306
NEBL	rs11012340	chr10:21,068,903-21,186,531	AX-11137867
NEBL	rs77698507	chr10:21,068,903-21,186,531	AX-15490515
NEBL	rs143584663	chr10:21,068,903-21,186,531	AX-83508650
NEBL	rs151012132	chr10:21,068,903-21,186,531	AX-86697981
NEBL	---	chr10:21,068,903-21,186,531	AX-86612589
NEBL	rs143930021	chr10:21,068,903-21,186,531	AX-83312129
NEBL	---	chr10:21,068,903-21,186,531	AX-94356606
NEBL	rs146218038	chr10:21,068,903-21,186,531	AX-83181274
NEBL	---	chr10:21,068,903-21,186,531	AX-15490992
NEBL	rs72785577	chr10:21,068,903-21,186,531	AX-29549331
NEBL	rs45463591	chr10:21,068,903-21,186,531	AX-12574303
NEBL	rs1409348	chr10:21,068,903-21,186,531	AX-11260843
NEBL	---	chr10:21,068,903-21,186,531	AX-90039930

NEBL	rs149647815	chr10:21,068,903-21,186,531	AX-86626415
NEBL	---	chr10:21,068,903-21,186,531	AX-86654677
NEBL	rs370026081	chr10:21,068,903-21,186,531	AX-86565178
NEBL	rs41277370	chr10:21,068,903-21,186,531	AX-29549387
NEBL	rs4025981	chr10:21,068,903-21,186,531	AX-11489344
NEBL	rs703088	chr10:21,068,903-21,186,531	AX-11602562
NEBL	rs41277372	chr10:21,068,903-21,186,531	AX-11491972
NEBL	rs703089	chr10:21,068,903-21,186,531	AX-38783277
NEBL	rs3791198	chr10:21,068,903-21,186,531	AX-11480892
NEBL	rs200799762	chr10:21,068,903-21,186,531	AX-83483306
NEBL	rs17797263	chr10:21,068,903-21,186,531	AX-11340815
NEBL	rs11012365	chr10:21,068,903-21,186,531	AX-11137870
NEBL	---	chr10:21,068,903-21,186,531	AX-94373357
NEBL	rs137973321	chr10:21,068,903-21,186,531	AX-83174711
NEBL	---	chr10:21,068,903-21,186,531	AX-94356608
NEBL	rs71541562	chr10:21,068,903-21,186,531	AX-82976012
NEBL	rs114662789	chr10:21,068,903-21,186,531	AX-15492585
NEBL	---	chr10:21,068,903-21,186,531	AX-90062123
NEBL	rs78714308	chr10:21,068,903-21,186,531	AX-29549505
NEBL	rs200249470	chr10:21,068,903-21,186,531	AX-86719579
NEBL	rs45594837	chr10:21,068,903-21,186,531	AX-11511345
NEBL	---	chr10:21,068,903-21,186,531	AX-86705405
NEBL	rs41277374	chr10:21,068,903-21,186,531	AX-83274197
NEBL	rs3791203	chr10:21,068,903-21,186,531	AX-15493072
NEBL	---	chr10:21,068,903-21,186,531	AX-83055986
NEBL	---	chr10:21,068,903-21,186,531	AX-86600755
NEXN	rs1166704	chr1:78,354,200-78,407,893	AX-13242334
NEXN	rs78977867	chr1:78,354,200-78,407,893	AX-37395083
NEXN	rs35117963	chr1:78,354,200-78,407,893	AX-40253949
NEXN	rs387907079	chr1:78,354,200-78,407,893	AX-90057304
NEXN	rs57444992	chr1:78,354,200-78,407,893	AX-32353307
NEXN	rs201447781	chr1:78,354,200-78,407,893	AX-83514612
NEXN	rs1166698	chr1:78,354,200-78,407,893	AX-13242360
NEXN	rs146245480	chr1:78,354,200-78,407,893	AX-82906723
NEXN	rs1780049	chr1:78,354,200-78,407,893	AX-40254003
NKX2-5	---	chr5:172,659,107-172,662,315	AX-90027806
NKX2-5	---	chr5:172,659,107-172,662,315	AX-90079794
NKX2-5	rs368366482	chr5:172,659,107-172,662,315	AX-86614380
NKX2-5	---	chr5:172,659,107-172,662,315	AX-90063634
NKX2-5	---	chr5:172,659,107-172,662,315	AX-90055228
NKX2-5	rs397515399	chr5:172,659,107-172,662,315	AX-90055969
NKX2-5	---	chr5:172,659,107-172,662,315	AX-90076446
NKX2-5	---	chr5:172,659,107-172,662,315	AX-90055989
NKX2-5	---	chr5:172,659,107-172,662,315	AX-90045022
NKX2-5	rs104893903	chr5:172,659,107-172,662,315	AX-88799199
NKX2-5	---	chr5:172,659,107-172,662,315	AX-90055230
NKX2-5	---	chr5:172,659,107-172,662,315	AX-90026346
NKX2-5	rs104893906	chr5:172,659,107-172,662,315	AX-88756155
NKX2-5	---	chr5:172,659,107-172,662,315	AX-90055232
NKX2-5	---	chr5:172,659,107-172,662,315	AX-90055233
NKX2-5	---	chr5:172,659,107-172,662,315	AX-90055234
NKX2-5	---	chr5:172,659,107-172,662,315	AX-90026350
NKX2-5	---	chr5:172,659,107-172,662,315	AX-84937850
NKX2-5	---	chr5:172,659,107-172,662,315	AX-90033841
NKX2-5	rs104893900	chr5:172,659,107-172,662,315	AX-88756152

NKX2-5	---	chr5:172,659,107-172,662,315	AX-90026351
NKX2-5	rs104893901	chr5:172,659,107-172,662,315	AX-88756153
NKX2-5	rs137852685	chr5:172,659,107-172,662,315	AX-90071019
NKX2-5	---	chr5:172,659,107-172,662,315	AX-90055237
NKX2-5	rs201582515	chr5:172,659,107-172,662,315	AX-90068708
NKX2-5	---	chr5:172,659,107-172,662,315	AX-90026353
NKX2-5	---	chr5:172,659,107-172,662,315	AX-90026354
NKX2-5	rs387906774	chr5:172,659,107-172,662,315	AX-90027657
NKX2-5	rs137852684	chr5:172,659,107-172,662,315	AX-83152307
NKX2-5	rs200039950	chr5:172,659,107-172,662,315	AX-83000946
NKX2-5	---	chr5:172,659,107-172,662,315	AX-90064511
NKX2-5	---	chr5:172,659,107-172,662,315	AX-90058035
NKX2-5	---	chr5:172,659,107-172,662,315	AX-90074177
NKX2-5	---	chr5:172,659,107-172,662,315	AX-90081263
NKX2-5	---	chr5:172,659,107-172,662,315	AX-90043951
NKX2-5	rs530270916	chr5:172,659,107-172,662,315	AX-90057291
NKX2-5	rs387906775	chr5:172,659,107-172,662,315	AX-90064025
NKX2-5	---	chr5:172,659,107-172,662,315	AX-90056671
NKX2-5	---	chr5:172,659,107-172,662,315	AX-90079688
NKX2-5	---	chr5:172,659,107-172,662,315	AX-90027397
NKX2-5	rs113818864	chr5:172,659,107-172,662,315	AX-83127340
NKX2-5	---	chr5:172,659,107-172,662,315	AX-90031530
NKX2-5	---	chr5:172,659,107-172,662,315	AX-90058774
NKX2-5	---	chr5:172,659,107-172,662,315	AX-83070044
NKX2-5	---	chr5:172,659,107-172,662,315	AX-90034772
NOTCH1	rs111627256	chr9:139,388,896-139,440,238	AX-83370234
NOTCH1	rs13301342	chr9:139,388,896-139,440,238	AX-42570265
NOTCH1	rs557319054	chr9:139,388,896-139,440,238	AX-90026396
NOTCH1	rs2229968	chr9:139,388,896-139,440,238	AX-36923409
NOTCH1	rs76371972	chr9:139,388,896-139,440,238	AX-69104722
NOTCH1	rs199860726	chr9:139,388,896-139,440,238	AX-82984307
NOTCH1	rs41309766	chr9:139,388,896-139,440,238	AX-88767936
NOTCH1	---	chr9:139,388,896-139,440,238	AX-90031176
NOTCH1	rs191645600	chr9:139,388,896-139,440,238	AX-83335053
NOTCH1	rs201215245	chr9:139,388,896-139,440,238	AX-83061255
NOTCH1	rs183156491	chr9:139,388,896-139,440,238	AX-83462637
NOTCH1	rs11145764	chr9:139,388,896-139,440,238	AX-36923411
NOTCH1	rs61751543	chr9:139,388,896-139,440,238	AX-83533615
NOTCH1	rs182330532	chr9:139,388,896-139,440,238	AX-83504650
NOTCH1	---	chr9:139,388,896-139,440,238	AX-90034091
NOTCH1	rs3812603	chr9:139,388,896-139,440,238	AX-42570299
NOTCH1	rs3124599	chr9:139,388,896-139,440,238	AX-42570305
NOTCH1	rs11145766	chr9:139,388,896-139,440,238	AX-42570311
NOTCH1	rs201620358	chr9:139,388,896-139,440,238	AX-83568398
NOTCH1	rs199506721	chr9:139,388,896-139,440,238	AX-83530699
NOTCH1	rs9411206	chr9:139,388,896-139,440,238	AX-36923459
NOTCH1	rs35136134	chr9:139,388,896-139,440,238	AX-83022691
NOTCH1	rs77596271	chr9:139,388,896-139,440,238	AX-36923475
NOTCH1	rs199719103	chr9:139,388,896-139,440,238	AX-83539539
NOTCH1	---	chr9:139,388,896-139,440,238	AX-83173353
NOTCH1	rs9411207	chr9:139,388,896-139,440,238	AX-36923483
NOTCH1	rs79782048	chr9:139,388,896-139,440,238	AX-83588486
NOTCH1	rs201077220	chr9:139,388,896-139,440,238	AX-83265431
NOTCH1	---	chr9:139,388,896-139,440,238	AX-90079160
NOTCH1	rs138504021	chr9:139,388,896-139,440,238	AX-83461579

NOTCH1	rs61755997	chr9:139,388,896-139,440,238	AX-86587434
NOTCH1	rs9411208	chr9:139,388,896-139,440,238	AX-36923517
NOTCH1	---	chr9:139,388,896-139,440,238	AX-83535214
NOTCH1	rs3124608	chr9:139,388,896-139,440,238	AX-36923567
NOTCH1	rs35863377	chr9:139,388,896-139,440,238	AX-36923571
NOTCH1	rs13290840	chr9:139,388,896-139,440,238	AX-36923589
NOTCH1	rs11145770	chr9:139,388,896-139,440,238	AX-42570357
NOTCH1	rs10870080	chr9:139,388,896-139,440,238	AX-36923599
NOTCH1	rs3013302	chr9:139,388,896-139,440,238	AX-42570365
NOTCH1	rs3013305	chr9:139,388,896-139,440,238	AX-36923653
NOTCH1	rs114832250	chr9:139,388,896-139,440,238	AX-55276551
NOTCH2	rs35586704	chr1:120,454,176-120,612,317	AX-83399206
NOTCH2	rs75831573	chr1:120,454,176-120,612,317	AX-83311672
NOTCH2	rs150516342	chr1:120,454,176-120,612,317	AX-83392205
NOTCH2	rs143236410	chr1:120,454,176-120,612,317	AX-83545615
NOTCH2	---	chr1:120,454,176-120,612,317	AX-94360429
NOTCH2	rs17258579	chr1:120,454,176-120,612,317	AX-11316585
NOTCH2	rs118188023	chr1:120,454,176-120,612,317	AX-86634716
NOTCH2	rs61752484	chr1:120,454,176-120,612,317	AX-16554678
NOTCH2	rs4659248	chr1:120,454,176-120,612,317	AX-11515825
NOTCH2	rs147223770	chr1:120,454,176-120,612,317	AX-83411972
NOTCH2	rs7543643	chr1:120,454,176-120,612,317	AX-12631533
NOTCH2	rs140311741	chr1:120,454,176-120,612,317	AX-83316341
NOTCH2	rs77801324	chr1:120,454,176-120,612,317	AX-16554792
NOTCH2	rs17024584	chr1:120,454,176-120,612,317	AX-39026899
NOTCH2	rs77542178	chr1:120,454,176-120,612,317	AX-16554820
NOTCH2	rs17258599	chr1:120,454,176-120,612,317	AX-11316586
NOTCH2	rs10923931	chr1:120,454,176-120,612,317	AX-11131906
NOTCH2	rs116062645	chr1:120,454,176-120,612,317	AX-16554843
NOTCH2	rs12132511	chr1:120,454,176-120,612,317	AX-29989143
OBSCN	rs117484136	chr1:228,395,831-228,566,575	AX-37442395
OBSCN	rs199979779	chr1:228,395,831-228,566,575	AX-83497732
OBSCN	---	chr1:228,395,831-228,566,575	AX-86605763
OBSCN	rs1771487	chr1:228,395,831-228,566,575	AX-83357471
OBSCN	rs2776853	chr1:228,395,831-228,566,575	AX-50090340
OBSCN	rs200897000	chr1:228,395,831-228,566,575	AX-83022697
OBSCN	rs180689095	chr1:228,395,831-228,566,575	AX-83597811
OBSCN	rs187569658	chr1:228,395,831-228,566,575	AX-83564049
OBSCN	rs199747438	chr1:228,395,831-228,566,575	AX-83512272
OBSCN	rs144514297	chr1:228,395,831-228,566,575	AX-83375589
OBSCN	---	chr1:228,395,831-228,566,575	AX-90045387
OBSCN	rs1757157	chr1:228,395,831-228,566,575	AX-16916970
OBSCN	rs201224400	chr1:228,395,831-228,566,575	AX-83325166
OBSCN	rs138166465	chr1:228,395,831-228,566,575	AX-83469055
OBSCN	---	chr1:228,395,831-228,566,575	AX-86572050
OBSCN	rs201854668	chr1:228,395,831-228,566,575	AX-82991021
OBSCN	rs6426495	chr1:228,395,831-228,566,575	AX-30661571
OBSCN	rs74142677	chr1:228,395,831-228,566,575	AX-16917117
OBSCN	rs185523702	chr1:228,395,831-228,566,575	AX-83560907
OBSCN	rs540662	chr1:228,395,831-228,566,575	AX-30661677
OBSCN	rs199646052	chr1:228,395,831-228,566,575	AX-83355164
OBSCN	---	chr1:228,395,831-228,566,575	AX-90028608
OBSCN	rs1188724	chr1:228,395,831-228,566,575	AX-30661743
OBSCN	rs200483572	chr1:228,395,831-228,566,575	AX-83284442
OBSCN	rs201790471	chr1:228,395,831-228,566,575	AX-83260665

OBSCN	rs200490850	chr1:228,395,831-228,566,575	AX-83101234
OBSCN	rs1188721	chr1:228,395,831-228,566,575	AX-39378935
OBSCN	rs1188722	chr1:228,395,831-228,566,575	AX-82927695
OBSCN	rs75280352	chr1:228,395,831-228,566,575	AX-83406319
OBSCN	rs201269256	chr1:228,395,831-228,566,575	AX-83556628
OBSCN	---	chr1:228,395,831-228,566,575	AX-86662388
OBSCN	---	chr1:228,395,831-228,566,575	AX-86624453
OBSCN	---	chr1:228,395,831-228,566,575	AX-86556897
OBSCN	rs200720682	chr1:228,395,831-228,566,575	AX-83415926
OBSCN	---	chr1:228,395,831-228,566,575	AX-86629217
OBSCN	rs3795783	chr1:228,395,831-228,566,575	AX-30661803
OBSCN	rs202052558	chr1:228,395,831-228,566,575	AX-83290250
OBSCN	rs12061320	chr1:228,395,831-228,566,575	AX-12427238
OBSCN	rs1188697	chr1:228,395,831-228,566,575	AX-83306671
OBSCN	rs150312546	chr1:228,395,831-228,566,575	AX-83052506
OBSCN	rs181194866	chr1:228,395,831-228,566,575	AX-83238214
OBSCN	rs3795785	chr1:228,395,831-228,566,575	AX-82959911
OBSCN	rs56168609	chr1:228,395,831-228,566,575	AX-30661829
OBSCN	rs201858717	chr1:228,395,831-228,566,575	AX-83125990
OBSCN	rs3795789	chr1:228,395,831-228,566,575	AX-83188604
OBSCN	rs4653546	chr1:228,395,831-228,566,575	AX-30661875
OBSCN	rs79902706	chr1:228,395,831-228,566,575	AX-30661881
OBSCN	rs199641194	chr1:228,395,831-228,566,575	AX-83488706
OBSCN	rs56218706	chr1:228,395,831-228,566,575	AX-30661907
OBSCN	rs147471011	chr1:228,395,831-228,566,575	AX-83526668
OBSCN	---	chr1:228,395,831-228,566,575	AX-83402548
OBSCN	rs564636	chr1:228,395,831-228,566,575	AX-39379025
OBSCN	---	chr1:228,395,831-228,566,575	AX-86730469
OBSCN	---	chr1:228,395,831-228,566,575	AX-86597266
OBSCN	rs56021350	chr1:228,395,831-228,566,575	AX-30661931
OBSCN	---	chr1:228,395,831-228,566,575	AX-86633119
OBSCN	rs61825286	chr1:228,395,831-228,566,575	AX-30661957
OBSCN	rs11584418	chr1:228,395,831-228,566,575	AX-39379053
OBSCN	rs41270183	chr1:228,395,831-228,566,575	AX-11491388
OBSCN	rs201781739	chr1:228,395,831-228,566,575	AX-83069701
OBSCN	rs201908193	chr1:228,395,831-228,566,575	AX-83322998
OBSCN	---	chr1:228,395,831-228,566,575	AX-86548208
OBSCN	rs435776	chr1:228,395,831-228,566,575	AX-83424283
OBSCN	rs55704206	chr1:228,395,831-228,566,575	AX-30662011
OBSCN	rs1150912	chr1:228,395,831-228,566,575	AX-83420821
OBSCN	rs116266256	chr1:228,395,831-228,566,575	AX-30662077
OBSCN	rs1188732	chr1:228,395,831-228,566,575	AX-83115605
OBSCN	rs61825301	chr1:228,395,831-228,566,575	AX-30662091
OBSCN	rs4653942	chr1:228,395,831-228,566,575	AX-83401047
OBSCN	---	chr1:228,395,831-228,566,575	AX-39379105
OBSCN	rs200399314	chr1:228,395,831-228,566,575	AX-83351806
OBSCN	rs3795800	chr1:228,395,831-228,566,575	AX-39379107
OBSCN	rs3795801	chr1:228,395,831-228,566,575	AX-83538127
OBSCN	rs371732381	chr1:228,395,831-228,566,575	AX-86547430
OBSCN	rs200354373	chr1:228,395,831-228,566,575	AX-83474162
OBSCN	rs142983293	chr1:228,395,831-228,566,575	AX-83551569
OBSCN	---	chr1:228,395,831-228,566,575	AX-86577014
OBSCN	rs373610	chr1:228,395,831-228,566,575	AX-39379121
OBSCN	rs41305122	chr1:228,395,831-228,566,575	AX-57094057
OBSCN	rs56359770	chr1:228,395,831-228,566,575	AX-83173967

OBSCN	---	chr1:228,395,831-228,566,575	AX-86616889
OBSCN	rs78577753	chr1:228,395,831-228,566,575	AX-30662169
OBSCN	rs287611	chr1:228,395,831-228,566,575	AX-39379139
OBSCN	rs287608	chr1:228,395,831-228,566,575	AX-30662193
OBSCN	rs56015866	chr1:228,395,831-228,566,575	AX-83580553
OBSCN	---	chr1:228,395,831-228,566,575	AX-86738582
OBSCN	rs113760892	chr1:228,395,831-228,566,575	AX-83123470
OBSCN	---	chr1:228,395,831-228,566,575	AX-86702581
OBSCN	rs199638902	chr1:228,395,831-228,566,575	AX-83263859
OBSCN	rs200706907	chr1:228,395,831-228,566,575	AX-83292312
OBSCN	rs3795809	chr1:228,395,831-228,566,575	AX-83072278
OBSCN	---	chr1:228,395,831-228,566,575	AX-90083169
OBSCN	rs191098985	chr1:228,395,831-228,566,575	AX-82929967
OBSCN	---	chr1:228,395,831-228,566,575	AX-86615996
OBSCN	rs56065114	chr1:228,395,831-228,566,575	AX-30662279
OBSCN	rs200362121	chr1:228,395,831-228,566,575	AX-82970327
OBSCN	rs1188710	chr1:228,395,831-228,566,575	AX-83079097
OBSCN	---	chr1:228,395,831-228,566,575	AX-86714262
OBSCN	rs78584475	chr1:228,395,831-228,566,575	AX-30662311
OBSCN	---	chr1:228,395,831-228,566,575	AX-83174268
OBSCN	rs140996408	chr1:228,395,831-228,566,575	AX-83337418
OBSCN	rs3795812	chr1:228,395,831-228,566,575	AX-39379229
OBSCN	rs883748	chr1:228,395,831-228,566,575	AX-30662381
OBSCN	---	chr1:228,395,831-228,566,575	AX-86570417
OBSCN	rs75458225	chr1:228,395,831-228,566,575	AX-30662421
OBSCN	---	chr1:228,395,831-228,566,575	AX-86623717
OBSCN	rs12035900	chr1:228,395,831-228,566,575	AX-39379271
OBSCN	rs145544878	chr1:228,395,831-228,566,575	AX-83320677
OBSCN	rs376526801	chr1:228,395,831-228,566,575	AX-86700852
OBSCN	rs35155240	chr1:228,395,831-228,566,575	AX-30662441
OBSCN	rs11585049	chr1:228,395,831-228,566,575	AX-83025254
OBSCN	rs35937215	chr1:228,395,831-228,566,575	AX-30662457
OBSCN	rs9727719	chr1:228,395,831-228,566,575	AX-30662459
OBSCN	rs55756479	chr1:228,395,831-228,566,575	AX-30662469
OBSCN	rs200436705	chr1:228,395,831-228,566,575	AX-83393133
OBSCN	---	chr1:228,395,831-228,566,575	AX-86597977
OBSCN	rs145597580	chr1:228,395,831-228,566,575	AX-82905391
OBSCN	rs202236799	chr1:228,395,831-228,566,575	AX-82998279
OBSCN	rs35186354	chr1:228,395,831-228,566,575	AX-83481881
OBSCN	rs201092597	chr1:228,395,831-228,566,575	AX-82996254
OBSCN	rs41305136	chr1:228,395,831-228,566,575	AX-83504494
OBSCN	rs560303483	chr1:228,395,831-228,566,575	AX-90033028
OBSCN	---	chr1:228,395,831-228,566,575	AX-86717665
OBSCN	rs72762066	chr1:228,395,831-228,566,575	AX-30662511
OBSCN	rs500049	chr1:228,395,831-228,566,575	AX-83570182
OBSCN	rs202143056	chr1:228,395,831-228,566,575	AX-83226923
OBSCN	rs199507775	chr1:228,395,831-228,566,575	AX-83570767
OBSCN	rs200271514	chr1:228,395,831-228,566,575	AX-86587928
OBSCN	rs377449573	chr1:228,395,831-228,566,575	AX-86677436
OBSCN	---	chr1:228,395,831-228,566,575	AX-90045430
OBSCN	rs144017124	chr1:228,395,831-228,566,575	AX-83455018
OBSCN	rs199866528	chr1:228,395,831-228,566,575	AX-83483102
OBSCN	rs200898403	chr1:228,395,831-228,566,575	AX-83443973
OBSCN	rs56174824	chr1:228,395,831-228,566,575	AX-83530358
OBSCN	rs182714476	chr1:228,395,831-228,566,575	AX-83317773

OBSCN	rs61730829	chr1:228,395,831-228,566,575	AX-30662561
PDLIM3	rs1801942	chr4:186,421,815-186,456,712	AX-11344751
PDLIM3	rs4635850	chr4:186,421,815-186,456,712	AX-14615153
PDLIM3	rs72715912	chr4:186,421,815-186,456,712	AX-34733565
PDLIM3	rs62347360	chr4:186,421,815-186,456,712	AX-83049245
PDLIM3	rs2306706	chr4:186,421,815-186,456,712	AX-11384069
PDLIM3	rs76900032	chr4:186,421,815-186,456,712	AX-14615172
PDLIM3	rs10446841	chr4:186,421,815-186,456,712	AX-11107138
PDLIM3	rs11944325	chr4:186,421,815-186,456,712	AX-41435889
PDLIM3	rs4605697	chr4:186,421,815-186,456,712	AX-34733603
PDLIM3	---	chr4:186,421,815-186,456,712	AX-34733619
PDLIM3	rs200354645	chr4:186,421,815-186,456,712	AX-83064570
PDLIM3	rs148570356	chr4:186,421,815-186,456,712	AX-83441411
PDLIM3	rs199476399	chr4:186,421,815-186,456,712	AX-86693420
PDLIM3	rs142951316	chr4:186,421,815-186,456,712	AX-83266358
PDLIM3	rs7664940	chr4:186,421,815-186,456,712	AX-14615229
PDLIM3	rs72715925	chr4:186,421,815-186,456,712	AX-34733671
PKP2	---	chr12:32,943,680-33,049,780	AX-94368624
PKP2	rs370599966	chr12:32,943,680-33,049,780	AX-91987228
PKP2	---	chr12:32,943,680-33,049,780	AX-90079668
PKP2	---	chr12:32,943,680-33,049,780	AX-90054753
PKP2	---	chr12:32,943,680-33,049,780	AX-90071491
PKP2	---	chr12:32,943,680-33,049,780	AX-90062120
PKP2	rs149968852	chr12:32,943,680-33,049,780	AX-83166542
PKP2	---	chr12:32,943,680-33,049,780	AX-90059422
PKP2	---	chr12:32,943,680-33,049,780	AX-90060352
PKP2	---	chr12:32,943,680-33,049,780	AX-94379401
PKP2	rs142362933	chr12:32,943,680-33,049,780	AX-92015445
PKP2	rs151264959	chr12:32,943,680-33,049,780	AX-82927945
PKP2	---	chr12:32,943,680-33,049,780	AX-90034559
PKP2	---	chr12:32,943,680-33,049,780	AX-94379402
PKP2	rs200947767	chr12:32,943,680-33,049,780	AX-91977809
PKP2	rs139734328	chr12:32,943,680-33,049,780	AX-82901855
PKP2	---	chr12:32,943,680-33,049,780	AX-94380924
PKP2	---	chr12:32,943,680-33,049,780	AX-90078101
PKP2	---	chr12:32,943,680-33,049,780	AX-90054755
PKP2	---	chr12:32,943,680-33,049,780	AX-90042607
PKP2	---	chr12:32,943,680-33,049,780	AX-90054756
PKP2	---	chr12:32,943,680-33,049,780	AX-90025872
PKP2	---	chr12:32,943,680-33,049,780	AX-94379404
PKP2	---	chr12:32,943,680-33,049,780	AX-94362655
PKP2	rs201487421	chr12:32,943,680-33,049,780	AX-94349465
PKP2	rs35826324	chr12:32,943,680-33,049,780	AX-16941424
PKP2	rs11615932	chr12:32,943,680-33,049,780	AX-11162678
PKP2	rs74072938	chr12:32,943,680-33,049,780	AX-16941437
PKP2	---	chr12:32,943,680-33,049,780	AX-94379406
PKP2	rs139098675	chr12:32,943,680-33,049,780	AX-92019704
PKP2	rs547215531	chr12:32,943,680-33,049,780	AX-94362657
PKP2	rs121434421	chr12:32,943,680-33,049,780	AX-88795816
PKP2	rs397517021	chr12:32,943,680-33,049,780	AX-91993938
PKP2	---	chr12:32,943,680-33,049,780	AX-90054758
PKP2	---	chr12:32,943,680-33,049,780	AX-94379408
PKP2	rs193922674	chr12:32,943,680-33,049,780	AX-86566512
PKP2	rs11052259	chr12:32,943,680-33,049,780	AX-12405408
PKP2	rs73090732	chr12:32,943,680-33,049,780	AX-16941468

PKP2	rs77624714	chr12:32,943,680-33,049,780	AX-30706725
PKP2	rs4931044	chr12:32,943,680-33,049,780	AX-16941475
PKP2	---	chr12:32,943,680-33,049,780	AX-94379409
PKP2	---	chr12:32,943,680-33,049,780	AX-90030316
PKP2	rs397517017	chr12:32,943,680-33,049,780	AX-90054759
PKP2	---	chr12:32,943,680-33,049,780	AX-90054760
PKP2	---	chr12:32,943,680-33,049,780	AX-90048312
PKP2	---	chr12:32,943,680-33,049,780	AX-94380925
PKP2	rs199583774	chr12:32,943,680-33,049,780	AX-91997508
PKP2	---	chr12:32,943,680-33,049,780	AX-90071493
PKP2	rs144601090	chr12:32,943,680-33,049,780	AX-86676334
PKP2	---	chr12:32,943,680-33,049,780	AX-90054761
PKP2	---	chr12:32,943,680-33,049,780	AX-90031793
PKP2	rs193922673	chr12:32,943,680-33,049,780	AX-88795857
PKP2	---	chr12:32,943,680-33,049,780	AX-94379410
PKP2	---	chr12:32,943,680-33,049,780	AX-90047537
PKP2	---	chr12:32,943,680-33,049,780	AX-94362661
PKP2	---	chr12:32,943,680-33,049,780	AX-90054762
PKP2	rs73090746	chr12:32,943,680-33,049,780	AX-30706787
PKP2	---	chr12:32,943,680-33,049,780	AX-90054763
PKP2	---	chr12:32,943,680-33,049,780	AX-86724815
PKP2	---	chr12:32,943,680-33,049,780	AX-90071494
PKP2	---	chr12:32,943,680-33,049,780	AX-94380700
PKP2	rs397517012	chr12:32,943,680-33,049,780	AX-90054764
PKP2	---	chr12:32,943,680-33,049,780	AX-90054765
PKP2	---	chr12:32,943,680-33,049,780	AX-90027487
PKP2	---	chr12:32,943,680-33,049,780	AX-90054766
PKP2	---	chr12:32,943,680-33,049,780	AX-90025882
PKP2	---	chr12:32,943,680-33,049,780	AX-90054768
PKP2	---	chr12:32,943,680-33,049,780	AX-94356287
PKP2	---	chr12:32,943,680-33,049,780	AX-90080383
PKP2	---	chr12:32,943,680-33,049,780	AX-90073725
PKP2	rs146102241	chr12:32,943,680-33,049,780	AX-83175800
PKP2	rs397517008	chr12:32,943,680-33,049,780	AX-90073382
PKP2	---	chr12:32,943,680-33,049,780	AX-90072269
PKP2	---	chr12:32,943,680-33,049,780	AX-90080115
PKP2	---	chr12:32,943,680-33,049,780	AX-94380701
PKP2	rs397517005	chr12:32,943,680-33,049,780	AX-90050099
PKP2	---	chr12:32,943,680-33,049,780	AX-94363953
PKP2	---	chr12:32,943,680-33,049,780	AX-90034109
PKP2	rs2389109	chr12:32,943,680-33,049,780	AX-39402863
PKP2	rs7301365	chr12:32,943,680-33,049,780	AX-11619795
PKP2	rs11609744	chr12:32,943,680-33,049,780	AX-39402895
PKP2	rs35292063	chr12:32,943,680-33,049,780	AX-16941569
PKP2	rs397517003	chr12:32,943,680-33,049,780	AX-90034780
PKP2	---	chr12:32,943,680-33,049,780	AX-90048907
PKP2	---	chr12:32,943,680-33,049,780	AX-90032721
PKP2	---	chr12:32,943,680-33,049,780	AX-90071495
PKP2	rs143782040	chr12:32,943,680-33,049,780	AX-16941597
PKP2	rs368740836	chr12:32,943,680-33,049,780	AX-91992050
PKP2	---	chr12:32,943,680-33,049,780	AX-90045283
PKP2	---	chr12:32,943,680-33,049,780	AX-90032373
PKP2	rs193922672	chr12:32,943,680-33,049,780	AX-88795856
PKP2	---	chr12:32,943,680-33,049,780	AX-90050075
PKP2	rs147240502	chr12:32,943,680-33,049,780	AX-83018137

PKP2	rs146882581	chr12:32,943,680-33,049,780	AX-83054752
PKP2	---	chr12:32,943,680-33,049,780	AX-90062771
PKP2	rs75650004	chr12:32,943,680-33,049,780	AX-16941601
PKP2	---	chr12:32,943,680-33,049,780	AX-92006800
PKP2	rs373399921	chr12:32,943,680-33,049,780	AX-94373039
PKP2	rs139159464	chr12:32,943,680-33,049,780	AX-83128733
PKP2	rs79032918	chr12:32,943,680-33,049,780	AX-16941620
PKP2	rs7304398	chr12:32,943,680-33,049,780	AX-11619997
PKP2	---	chr12:32,943,680-33,049,780	AX-90033062
PKP2	---	chr12:32,943,680-33,049,780	AX-94373040
PKP2	---	chr12:32,943,680-33,049,780	AX-90072747
PKP2	rs3748278	chr12:32,943,680-33,049,780	AX-92005743
PKP2	---	chr12:32,943,680-33,049,780	AX-94356291
PKP2	rs397516990	chr12:32,943,680-33,049,780	AX-90025884
PKP2	---	chr12:32,943,680-33,049,780	AX-90054770
PKP2	rs372827156	chr12:32,943,680-33,049,780	AX-86728805
PKP2	---	chr12:32,943,680-33,049,780	AX-90079381
PKP2	rs192041220	chr12:32,943,680-33,049,780	AX-94349468
PKP2	---	chr12:32,943,680-33,049,780	AX-94363954
PKP2	rs397516989	chr12:32,943,680-33,049,780	AX-90044197
PKP2	---	chr12:32,943,680-33,049,780	AX-90071866
PKP2	---	chr12:32,943,680-33,049,780	AX-94363956
PKP2	---	chr12:32,943,680-33,049,780	AX-90045294
PKP2	---	chr12:32,943,680-33,049,780	AX-94356292
PKP2	---	chr12:32,943,680-33,049,780	AX-90029942
PKP2	rs76774152	chr12:32,943,680-33,049,780	AX-16941665
PKP2	rs79688317	chr12:32,943,680-33,049,780	AX-16941669
PKP2	rs75422105	chr12:32,943,680-33,049,780	AX-16941675
PKP2	rs78966607	chr12:32,943,680-33,049,780	AX-16941688
PKP2	rs1454933	chr12:32,943,680-33,049,780	AX-11265183
PKP2	---	chr12:32,943,680-33,049,780	AX-90065813
PKP2	---	chr12:32,943,680-33,049,780	AX-90054771
PKP2	---	chr12:32,943,680-33,049,780	AX-94373043
PKP2	---	chr12:32,943,680-33,049,780	AX-94373044
PKP2	rs397516986	chr12:32,943,680-33,049,780	AX-90054772
PKP2	rs56869013	chr12:32,943,680-33,049,780	AX-92038028
PKP2	rs142742483	chr12:32,943,680-33,049,780	AX-92013075
PKP2	---	chr12:32,943,680-33,049,780	AX-83337500
PKP2	rs1046116	chr12:32,943,680-33,049,780	AX-11107882
PKP2	rs73090770	chr12:32,943,680-33,049,780	AX-30707059
PKP2	---	chr12:32,943,680-33,049,780	AX-94376954
PKP2	rs139851304	chr12:32,943,680-33,049,780	AX-83048506
PKP2	---	chr12:32,943,680-33,049,780	AX-94363957
PKP2	---	chr12:32,943,680-33,049,780	AX-90065399
PKP2	---	chr12:32,943,680-33,049,780	AX-90073352
PKP2	rs142636176	chr12:32,943,680-33,049,780	AX-91984612
PKP2	rs61729382	chr12:32,943,680-33,049,780	AX-91985130
PKP2	rs61729381	chr12:32,943,680-33,049,780	AX-92014858
PKP2	---	chr12:32,943,680-33,049,780	AX-94373045
PKP2	---	chr12:32,943,680-33,049,780	AX-90075826
PKP2	---	chr12:32,943,680-33,049,780	AX-94363958
PKP2	rs201944276	chr12:32,943,680-33,049,780	AX-83311607
PKP2	rs3748279	chr12:32,943,680-33,049,780	AX-12559803
PKP2	---	chr12:32,943,680-33,049,780	AX-94373046
PKP2	rs62001016	chr12:32,943,680-33,049,780	AX-37583959

PKP2	---	chr12:32,943,680-33,049,780	AX-90061623
PKP2	---	chr12:32,943,680-33,049,780	AX-94363959
PKP2	---	chr12:32,943,680-33,049,780	AX-94356297
PKP2	---	chr12:32,943,680-33,049,780	AX-90035930
PKP2	rs201580443	chr12:32,943,680-33,049,780	AX-94366224
PKP2	rs62001015	chr12:32,943,680-33,049,780	AX-83523606
PKP2	---	chr12:32,943,680-33,049,780	AX-86606553
PKP2	---	chr12:32,943,680-33,049,780	AX-90054773
PKP2	---	chr12:32,943,680-33,049,780	AX-90043416
PKP2	---	chr12:32,943,680-33,049,780	AX-90054774
PKP2	---	chr12:32,943,680-33,049,780	AX-94356298
PKP2	---	chr12:32,943,680-33,049,780	AX-90073062
PKP2	---	chr12:32,943,680-33,049,780	AX-94362662
PKP2	---	chr12:32,943,680-33,049,780	AX-94364178
PKP2	---	chr12:32,943,680-33,049,780	AX-90077485
PKP2	---	chr12:32,943,680-33,049,780	AX-94362663
PKP2	rs397517027	chr12:32,943,680-33,049,780	AX-86714092
PKP2	rs199643407	chr12:32,943,680-33,049,780	AX-90082264
PKP2	rs2389115	chr12:32,943,680-33,049,780	AX-91976080
PKP2	---	chr12:32,943,680-33,049,780	AX-94362664
PKP2	---	chr12:32,943,680-33,049,780	AX-94362665
PKP2	---	chr12:32,943,680-33,049,780	AX-90025890
PKP2	---	chr12:32,943,680-33,049,780	AX-94362666
PKP2	---	chr12:32,943,680-33,049,780	AX-90025891
PKP2	rs139215336	chr12:32,943,680-33,049,780	AX-82994891
PKP2	rs149542398	chr12:32,943,680-33,049,780	AX-83294881
PKP2	---	chr12:32,943,680-33,049,780	AX-90054777
PKP2	---	chr12:32,943,680-33,049,780	AX-94362667
PKP2	---	chr12:32,943,680-33,049,780	AX-90054778
PKP2	---	chr12:32,943,680-33,049,780	AX-90076001
PKP2	rs121434420	chr12:32,943,680-33,049,780	AX-88795815
PKP2	---	chr12:32,943,680-33,049,780	AX-90029868
PKP2	rs11052283	chr12:32,943,680-33,049,780	AX-39403077
PKP2	rs79323236	chr12:32,943,680-33,049,780	AX-30707177
PKP2	rs7302851	chr12:32,943,680-33,049,780	AX-39403091
PKP2	rs4420362	chr12:32,943,680-33,049,780	AX-39403123
PKP2	rs7959358	chr12:32,943,680-33,049,780	AX-39403129
PKP2	rs74072972	chr12:32,943,680-33,049,780	AX-16941758
PKP2	rs56969496	chr12:32,943,680-33,049,780	AX-30707229
PKP2	---	chr12:32,943,680-33,049,780	AX-90058392
PKP2	rs75909145	chr12:32,943,680-33,049,780	AX-83480287
PKP2	rs397517014	chr12:32,943,680-33,049,780	AX-91981242
PKP2	---	chr12:32,943,680-33,049,780	AX-94351847
PKP2	---	chr12:32,943,680-33,049,780	AX-90079586
PKP2	---	chr12:32,943,680-33,049,780	AX-94364179
PKP2	---	chr12:32,943,680-33,049,780	AX-90046883
PKP2	rs199601548	chr12:32,943,680-33,049,780	AX-83251253
PKP2	---	chr12:32,943,680-33,049,780	AX-86723204
PKP2	rs146708884	chr12:32,943,680-33,049,780	AX-82905670
PKP2	---	chr12:32,943,680-33,049,780	AX-94379418
PKP2	rs201210997	chr12:32,943,680-33,049,780	AX-86567210
PKP2	---	chr12:32,943,680-33,049,780	AX-94380929
PKP2	---	chr12:32,943,680-33,049,780	AX-90079601
PKP2	---	chr12:32,943,680-33,049,780	AX-94364181
PKP2	rs143004808	chr12:32,943,680-33,049,780	AX-83409678

PKP2	---	chr12:32,943,680-33,049,780	AX-94362669
PKP2	---	chr12:32,943,680-33,049,780	AX-94362670
PLN	rs3752581	chr6:118,869,442-118,881,587	AX-156276038
PLN	rs3752581	chr6:118,869,442-118,881,587	AX-156293052
PLN	rs12211576	chr6:118,869,442-118,881,587	AX-35523691
PLN	rs34030625	chr6:118,869,442-118,881,587	AX-151169828
PLN	rs34030625	chr6:118,869,442-118,881,587	AX-156284247
PLN	rs9489436	chr6:118,869,442-118,881,587	AX-156276039
PLN	rs73766547	chr6:118,869,442-118,881,587	AX-156276040
PLN	rs73766547	chr6:118,869,442-118,881,587	AX-156293054
PLN	rs9489437	chr6:118,869,442-118,881,587	AX-156276041
PLN	rs9489437	chr6:118,869,442-118,881,587	AX-156293055
PLN	rs13192336	chr6:118,869,442-118,881,587	AX-156276042
PLN	rs13192574	chr6:118,869,442-118,881,587	AX-148058677
PLN	rs13192574	chr6:118,869,442-118,881,587	AX-156276043
PLN	rs12153938	chr6:118,869,442-118,881,587	AX-119373847
PLN	rs12153938	chr6:118,869,442-118,881,587	AX-148154169
PLN	rs12153955	chr6:118,869,442-118,881,587	AX-41830225
PLN	rs9481825	chr6:118,869,442-118,881,587	AX-15209795
PLN	rs9481826	chr6:118,869,442-118,881,587	AX-122963132
PLN	rs2356497	chr6:118,869,442-118,881,587	AX-105165603
PLN	rs9489438	chr6:118,869,442-118,881,587	AX-156276044
PLN	rs9489438	chr6:118,869,442-118,881,587	AX-156293056
PLN	rs9489439	chr6:118,869,442-118,881,587	AX-15209800
PLN	rs11753886	chr6:118,869,442-118,881,587	AX-112924501
PLN	rs11753886	chr6:118,869,442-118,881,587	AX-112998577
PLN	rs111033559	chr6:118,869,442-118,881,587	AX-90071066
PLN	---	chr6:118,869,442-118,881,587	AX-90033888
PLN	---	chr6:118,869,442-118,881,587	AX-90074970
PLN	rs111033560	chr6:118,869,442-118,881,587	AX-86704343
PLN	---	chr6:118,869,442-118,881,587	AX-15209804
PRKAG2	rs17714947	chr7:151,253,201-151,574,316	AX-42144857
PRKAG2	rs113234987	chr7:151,253,201-151,574,316	AX-15612357
PRKAG2	rs6464147	chr7:151,253,201-151,574,316	AX-42144877
PRKAG2	rs267606979	chr7:151,253,201-151,574,316	AX-90055261
PRKAG2	rs28763998	chr7:151,253,201-151,574,316	AX-42144879
PRKAG2	rs201542789	chr7:151,253,201-151,574,316	AX-83055148
PRKAG2	---	chr7:151,253,201-151,574,316	AX-88757021
PRKAG2	rs121908990	chr7:151,253,201-151,574,316	AX-90042275
PRKAG2	rs267606977	chr7:151,253,201-151,574,316	AX-90031280
PRKAG2	---	chr7:151,253,201-151,574,316	AX-90034042
PRKAG2	rs267606976	chr7:151,253,201-151,574,316	AX-90027486
PRKAG2	rs17642575	chr7:151,253,201-151,574,316	AX-12502368
PRKAG2	rs28938173	chr7:151,253,201-151,574,316	AX-88756944
PRKAG2	rs121908988	chr7:151,253,201-151,574,316	AX-90042273
PRKAG2	rs2302530	chr7:151,253,201-151,574,316	AX-11383641
PRKAG2	rs74378494	chr7:151,253,201-151,574,316	AX-15612400
PRKAG2	---	chr7:151,253,201-151,574,316	AX-90074819
PRKAG2	rs121908987	chr7:151,253,201-151,574,316	AX-90042272
PRKAG2	rs6953900	chr7:151,253,201-151,574,316	AX-15612430
PRKAG2	rs2538038	chr7:151,253,201-151,574,316	AX-11397474
PRKAG2	rs2538045	chr7:151,253,201-151,574,316	AX-11397476
PRKAG2	rs6464156	chr7:151,253,201-151,574,316	AX-42145019
PRKAG2	rs1362237	chr7:151,253,201-151,574,316	AX-36094381
PRKAG2	rs870441	chr7:151,253,201-151,574,316	AX-11670854

PRKAG2	rs1419935	chr7:151,253,201-151,574,316	AX-11261750
PRKAG2	rs4725417	chr7:151,253,201-151,574,316	AX-36094417
PRKAG2	rs4726070	chr7:151,253,201-151,574,316	AX-11521193
PRKAG2	rs200736454	chr7:151,253,201-151,574,316	AX-83050478
PRKAG2	rs150248487	chr7:151,253,201-151,574,316	AX-90067477
PRKAG2	rs79780320	chr7:151,253,201-151,574,316	AX-36094447
PRKAG2	rs4726080	chr7:151,253,201-151,574,316	AX-36094455
PRKAG2	rs2254644	chr7:151,253,201-151,574,316	AX-11379154
PRKAG2	rs954482	chr7:151,253,201-151,574,316	AX-11693619
PRKAG2	rs73158145	chr7:151,253,201-151,574,316	AX-36094521
PRKAG2	rs2727540	chr7:151,253,201-151,574,316	AX-36094549
PRKAG2	rs12535248	chr7:151,253,201-151,574,316	AX-36094587
PRKAG2	rs2727553	chr7:151,253,201-151,574,316	AX-42145133
PRKAG2	rs10258842	chr7:151,253,201-151,574,316	AX-36094655
PRKAG2	rs62478181	chr7:151,253,201-151,574,316	AX-36094677
PRKAG2	rs2727551	chr7:151,253,201-151,574,316	AX-42145167
PRKAG2	rs112810819	chr7:151,253,201-151,574,316	AX-36094701
PRKAG2	rs10269789	chr7:151,253,201-151,574,316	AX-36094711
PRKAG2	rs6947064	chr7:151,253,201-151,574,316	AX-11596366
PRKAG2	rs6967507	chr7:151,253,201-151,574,316	AX-42145193
PRKAG2	rs2374270	chr7:151,253,201-151,574,316	AX-42145219
PRKAG2	rs2727563	chr7:151,253,201-151,574,316	AX-42145223
PRKAG2	rs2727562	chr7:151,253,201-151,574,316	AX-36094799
PRKAG2	rs7805747	chr7:151,253,201-151,574,316	AX-11646811
PRKAG2	rs28533208	chr7:151,253,201-151,574,316	AX-36094851
PRKAG2	rs115185349	chr7:151,253,201-151,574,316	AX-38397637
PRKAG2	rs10224002	chr7:151,253,201-151,574,316	AX-12385674
PRKAG2	rs1860743	chr7:151,253,201-151,574,316	AX-42145259
PRKAG2	rs62478183	chr7:151,253,201-151,574,316	AX-36094943
PRKAG2	rs4726086	chr7:151,253,201-151,574,316	AX-36094959
PRKAG2	rs2536069	chr7:151,253,201-151,574,316	AX-42145267
PRKAG2	rs1108722	chr7:151,253,201-151,574,316	AX-15612711
PRKAG2	rs34078741	chr7:151,253,201-151,574,316	AX-36095021
PRKAG2	rs1001116	chr7:151,253,201-151,574,316	AX-42145317
PRKAG2	rs11771445	chr7:151,253,201-151,574,316	AX-36095075
PRKAG2	rs11773668	chr7:151,253,201-151,574,316	AX-15612725
PRKAG2	rs62478188	chr7:151,253,201-151,574,316	AX-15612730
PRKAG2	rs7801616	chr7:151,253,201-151,574,316	AX-42145351
PRKAG2	rs35798852	chr7:151,253,201-151,574,316	AX-36095105
PRKAG2	rs2109782	chr7:151,253,201-151,574,316	AX-42145353
PRKAG2	rs868624	chr7:151,253,201-151,574,316	AX-11670689
PRKAG2	rs7800069	chr7:151,253,201-151,574,316	AX-42145369
PRKAG2	rs3934595	chr7:151,253,201-151,574,316	AX-36095173
PRKAG2	rs6965834	chr7:151,253,201-151,574,316	AX-42145379
PRKAG2	rs4726091	chr7:151,253,201-151,574,316	AX-36095205
PRKAG2	rs4725431	chr7:151,253,201-151,574,316	AX-42145381
PRKAG2	rs4418266	chr7:151,253,201-151,574,316	AX-11504871
PRKAG2	---	chr7:151,253,201-151,574,316	AX-42145397
PRKAG2	rs9632641	chr7:151,253,201-151,574,316	AX-42145401
PRKAG2	rs6464170	chr7:151,253,201-151,574,316	AX-42145407
PRKAG2	rs4726093	chr7:151,253,201-151,574,316	AX-36095237
PRKAG2	rs12375159	chr7:151,253,201-151,574,316	AX-36095243
PRKAG2	rs78291918	chr7:151,253,201-151,574,316	AX-36095245
PRKAG2	rs12703157	chr7:151,253,201-151,574,316	AX-36095257
PRKAG2	rs62478217	chr7:151,253,201-151,574,316	AX-36095259

PRKAG2	rs6953882	chr7:151,253,201-151,574,316	AX-36095265
PRKAG2	rs6979356	chr7:151,253,201-151,574,316	AX-36095283
PRKAG2	rs12671980	chr7:151,253,201-151,574,316	AX-36095285
PRKAG2	rs4425665	chr7:151,253,201-151,574,316	AX-15612771
PRKAG2	rs7796138	chr7:151,253,201-151,574,316	AX-42145417
PRKAG2	rs11770585	chr7:151,253,201-151,574,316	AX-36095325
PRKAG2	rs34735358	chr7:151,253,201-151,574,316	AX-36095327
PRKAG2	rs79918575	chr7:151,253,201-151,574,316	AX-36095345
PRKAG2	rs7789699	chr7:151,253,201-151,574,316	AX-42145419
PRKAG2	rs56037571	chr7:151,253,201-151,574,316	AX-36095359
PRKAG2	rs11768953	chr7:151,253,201-151,574,316	AX-36095377
PRKAG2	rs58943536	chr7:151,253,201-151,574,316	AX-36095379
PRKAG2	rs4726097	chr7:151,253,201-151,574,316	AX-42145427
PRKAG2	rs56131024	chr7:151,253,201-151,574,316	AX-36095403
PRKAG2	rs34802637	chr7:151,253,201-151,574,316	AX-42145437
PRKAG2	rs58825093	chr7:151,253,201-151,574,316	AX-36095419
PRKAG2	rs10257529	chr7:151,253,201-151,574,316	AX-15612789
PRKAG2	rs12539356	chr7:151,253,201-151,574,316	AX-42145455
PRKAG2	rs62478246	chr7:151,253,201-151,574,316	AX-36095455
PRKAG2	rs4632971	chr7:151,253,201-151,574,316	AX-42145465
PRKAG2	rs10952318	chr7:151,253,201-151,574,316	AX-42145469
PRKAG2	rs56129741	chr7:151,253,201-151,574,316	AX-15612812
PRKAG2	rs6954429	chr7:151,253,201-151,574,316	AX-42145471
PRKAG2	rs34244587	chr7:151,253,201-151,574,316	AX-36095491
PRKAG2	rs34388354	chr7:151,253,201-151,574,316	AX-36095503
PRKAG2	rs1881629	chr7:151,253,201-151,574,316	AX-42145489
PRKAG2	rs13309576	chr7:151,253,201-151,574,316	AX-36095523
PRKAG2	rs13309585	chr7:151,253,201-151,574,316	AX-42145493
PRKAG2	rs75064047	chr7:151,253,201-151,574,316	AX-38397717
PRKAG2	rs75808206	chr7:151,253,201-151,574,316	AX-36095565
PRKAG2	rs11760502	chr7:151,253,201-151,574,316	AX-42145529
PRKAG2	rs56138260	chr7:151,253,201-151,574,316	AX-36095629
PRKAG2	rs1881625	chr7:151,253,201-151,574,316	AX-36095673
PRKAG2	rs13238117	chr7:151,253,201-151,574,316	AX-42145541
PRKAG2	rs74782537	chr7:151,253,201-151,574,316	AX-36095677
PRKAG2	rs56998446	chr7:151,253,201-151,574,316	AX-36095701
PRKAG2	rs79369446	chr7:151,253,201-151,574,316	AX-36095713
PRKAG2	rs57486649	chr7:151,253,201-151,574,316	AX-36095767
PRKAG2	rs62480473	chr7:151,253,201-151,574,316	AX-36095769
PRKAG2	rs73160048	chr7:151,253,201-151,574,316	AX-36095797
PRKAG2	rs7795096	chr7:151,253,201-151,574,316	AX-42145583
PRKAG2	rs7776530	chr7:151,253,201-151,574,316	AX-42145587
PRKAG2	---	chr7:151,253,201-151,574,316	AX-36095889
PRKAG2	rs13245627	chr7:151,253,201-151,574,316	AX-36095905
PRKAG2	rs7789674	chr7:151,253,201-151,574,316	AX-42145615
PRKAG2	rs34867733	chr7:151,253,201-151,574,316	AX-36095961
PRKAG2	rs9640300	chr7:151,253,201-151,574,316	AX-42145623
PRKAG2	rs56118280	chr7:151,253,201-151,574,316	AX-36096007
PRKAG2	rs12669153	chr7:151,253,201-151,574,316	AX-11222050
PRKAG2	---	chr7:151,253,201-151,574,316	AX-42145637
PRKAG2	rs78881925	chr7:151,253,201-151,574,316	AX-36096073
PRKAG2	rs77902041	chr7:151,253,201-151,574,316	AX-36096081
PRKAG2	rs116605521	chr7:151,253,201-151,574,316	AX-38397741
PRKAG2	rs66628686	chr7:151,253,201-151,574,316	AX-36096083
PSEN1	rs1800844	chr14:73,603,143-73,690,399	AX-12821669

PSEN1	rs214279	chr14:73,603,143-73,690,399	AX-156280258
PSEN1	rs214279	chr14:73,603,143-73,690,399	AX-156295707
PSEN1	rs214278	chr14:73,603,143-73,690,399	AX-148217977
PSEN1	rs214278	chr14:73,603,143-73,690,399	AX-96781361
PSEN1	rs190932247	chr14:73,603,143-73,690,399	AX-92438212
PSEN1	rs190932247	chr14:73,603,143-73,690,399	AX-92649235
PSEN1	rs214277	chr14:73,603,143-73,690,399	AX-151367651
PSEN1	rs214277	chr14:73,603,143-73,690,399	AX-156271330
PSEN1	rs35581508	chr14:73,603,143-73,690,399	AX-106898197
PSEN1	rs35581508	chr14:73,603,143-73,690,399	AX-156271331
PSEN1	rs7145968	chr14:73,603,143-73,690,399	AX-11630400
PSEN1	rs3025774	chr14:73,603,143-73,690,399	AX-39808003
PSEN1	rs35891140	chr14:73,603,143-73,690,399	AX-156266502
PSEN1	rs60320273	chr14:73,603,143-73,690,399	AX-148566787
PSEN1	rs60320273	chr14:73,603,143-73,690,399	AX-156266503
PSEN1	rs57815955	chr14:73,603,143-73,690,399	AX-156266504
PSEN1	rs57815955	chr14:73,603,143-73,690,399	AX-156286985
PSEN1	---	chr14:73,603,143-73,690,399	AX-39808013
PSEN1	rs149562759	chr14:73,603,143-73,690,399	AX-86673936
PSEN1	rs34623110	chr14:73,603,143-73,690,399	AX-39808017
PSEN1	rs214274	chr14:73,603,143-73,690,399	AX-151246401
PSEN1	rs214274	chr14:73,603,143-73,690,399	AX-156269305
PSEN1	rs8018725	chr14:73,603,143-73,690,399	AX-11660312
PSEN1	rs10151436	chr14:73,603,143-73,690,399	AX-112924578
PSEN1	rs10151436	chr14:73,603,143-73,690,399	AX-113481262
PSEN1	rs214273	chr14:73,603,143-73,690,399	AX-11370055
PSEN1	rs115123722	chr14:73,603,143-73,690,399	AX-92465313
PSEN1	rs115123722	chr14:73,603,143-73,690,399	AX-92676336
PSEN1	rs8008686	chr14:73,603,143-73,690,399	AX-148472432
PSEN1	rs8008686	chr14:73,603,143-73,690,399	AX-88743101
PSEN1	rs75266743	chr14:73,603,143-73,690,399	AX-12821680
PSEN1	rs10135273	chr14:73,603,143-73,690,399	AX-148784733
PSEN1	rs10135273	chr14:73,603,143-73,690,399	AX-156271332
PSEN1	rs61986878	chr14:73,603,143-73,690,399	AX-92583804
PSEN1	rs61986878	chr14:73,603,143-73,690,399	AX-92794827
PSEN1	rs116226928	chr14:73,603,143-73,690,399	AX-31449041
PSEN1	rs10136911	chr14:73,603,143-73,690,399	AX-105134778
PSEN1	rs10136911	chr14:73,603,143-73,690,399	AX-156271333
PSEN1	rs10147873	chr14:73,603,143-73,690,399	AX-115156262
PSEN1	rs10147873	chr14:73,603,143-73,690,399	AX-156271334
PSEN1	rs68084031	chr14:73,603,143-73,690,399	AX-12821684
PSEN1	rs67647403	chr14:73,603,143-73,690,399	AX-12821686
PSEN1	rs12883075	chr14:73,603,143-73,690,399	AX-31449055
PSEN1	rs10143704	chr14:73,603,143-73,690,399	AX-115156667
PSEN1	rs10143704	chr14:73,603,143-73,690,399	AX-156289852
PSEN1	rs77119530	chr14:73,603,143-73,690,399	AX-123101577
PSEN1	rs12588591	chr14:73,603,143-73,690,399	AX-92511346
PSEN1	rs12588591	chr14:73,603,143-73,690,399	AX-92722369
PSEN1	rs12878362	chr14:73,603,143-73,690,399	AX-148430459
PSEN1	rs12878362	chr14:73,603,143-73,690,399	AX-156266505
PSEN1	rs8011335	chr14:73,603,143-73,690,399	AX-151292003
PSEN1	rs8011335	chr14:73,603,143-73,690,399	AX-156266506
PSEN1	rs61986883	chr14:73,603,143-73,690,399	AX-51108531
PSEN1	rs57050244	chr14:73,603,143-73,690,399	AX-12821698
PSEN1	---	chr14:73,603,143-73,690,399	AX-92486697

PSEN1	---	chr14:73,603,143-73,690,399	AX-92697720
PSEN1	rs362350	chr14:73,603,143-73,690,399	AX-11475790
PSEN1	rs139058678	chr14:73,603,143-73,690,399	AX-151415461
PSEN1	rs139058678	chr14:73,603,143-73,690,399	AX-156298546
PSEN1	rs72734455	chr14:73,603,143-73,690,399	AX-106919467
PSEN1	rs214271	chr14:73,603,143-73,690,399	AX-92522220
PSEN1	rs214271	chr14:73,603,143-73,690,399	AX-92733243
PSEN1	rs117170264	chr14:73,603,143-73,690,399	AX-12821700
PSEN1	rs199797974	chr14:73,603,143-73,690,399	AX-151395482
PSEN1	rs199797974	chr14:73,603,143-73,690,399	AX-156286279
PSEN1	rs10564849	chr14:73,603,143-73,690,399	AX-121933278
PSEN1	rs3025780	chr14:73,603,143-73,690,399	AX-112995311
PSEN1	rs61986885	chr14:73,603,143-73,690,399	AX-92552820
PSEN1	rs2283427	chr14:73,603,143-73,690,399	AX-115156623
PSEN1	rs2283427	chr14:73,603,143-73,690,399	AX-156266507
PSEN1	rs73305038	chr14:73,603,143-73,690,399	AX-156280259
PSEN1	rs73305038	chr14:73,603,143-73,690,399	AX-156295708
PSEN1	rs9743635	chr14:73,603,143-73,690,399	AX-151299533
PSEN1	rs9743635	chr14:73,603,143-73,690,399	AX-156280260
PSEN1	rs140189461	chr14:73,603,143-73,690,399	AX-94369263
PSEN1	rs63749824	chr14:73,603,143-73,690,399	AX-88796133
PSEN1	rs63749967	chr14:73,603,143-73,690,399	AX-88753095
PSEN1	---	chr14:73,603,143-73,690,399	AX-90043987
PSEN1	rs63750307	chr14:73,603,143-73,690,399	AX-94380496
PSEN1	rs63750599	chr14:73,603,143-73,690,399	AX-88753117
PSEN1	rs63750815	chr14:73,603,143-73,690,399	AX-88753123
PSEN1	rs63751141	chr14:73,603,143-73,690,399	AX-88753143
PSEN1	rs146855665	chr14:73,603,143-73,690,399	AX-94369273
PSEN1	rs63750831	chr14:73,603,143-73,690,399	AX-88753124
PSEN1	rs63750601	chr14:73,603,143-73,690,399	AX-88796163
PSEN1	---	chr14:73,603,143-73,690,399	AX-90060745
PSEN1	---	chr14:73,603,143-73,690,399	AX-94360747
PSEN1	---	chr14:73,603,143-73,690,399	AX-90026709
PSEN1	rs63750325	chr14:73,603,143-73,690,399	AX-90040794
PSEN1	rs63750321	chr14:73,603,143-73,690,399	AX-88753106
PSEN1	---	chr14:73,603,143-73,690,399	AX-90055595
PSEN1	rs63751399	chr14:73,603,143-73,690,399	AX-90055832
PSEN1	rs63751475	chr14:73,603,143-73,690,399	AX-88817241
PSEN1	rs362355	chr14:73,603,143-73,690,399	AX-39808057
PSEN1	rs362340	chr14:73,603,143-73,690,399	AX-12821706
PSEN1	rs73305043	chr14:73,603,143-73,690,399	AX-156266508
PSEN1	rs73305043	chr14:73,603,143-73,690,399	AX-156286986
PSEN1	---	chr14:73,603,143-73,690,399	AX-90055596
PSEN1	rs63750450	chr14:73,603,143-73,690,399	AX-88753109
PSEN1	---	chr14:73,603,143-73,690,399	AX-90055597
PSEN1	---	chr14:73,603,143-73,690,399	AX-90065758
PSEN1	rs63749805	chr14:73,603,143-73,690,399	AX-90062151
PSEN1	rs63750800	chr14:73,603,143-73,690,399	AX-90040819
PSEN1	---	chr14:73,603,143-73,690,399	AX-90026714
PSEN1	---	chr14:73,603,143-73,690,399	AX-90064018
PSEN1	rs63750378	chr14:73,603,143-73,690,399	AX-90069682
PSEN1	---	chr14:73,603,143-73,690,399	AX-90027871
PSEN1	rs63750353	chr14:73,603,143-73,690,399	AX-88796152
PSEN1	rs63751278	chr14:73,603,143-73,690,399	AX-88753149
PSEN1	rs41345849	chr14:73,603,143-73,690,399	AX-90040770

PSEN1	rs63751037	chr14:73,603,143-73,690,399	AX-90040834
PSEN1	rs63751106	chr14:73,603,143-73,690,399	AX-90037338
PSEN1	---	chr14:73,603,143-73,690,399	AX-90034632
PSEN1	rs63750522	chr14:73,603,143-73,690,399	AX-90040801
PSEN1	---	chr14:73,603,143-73,690,399	AX-90026716
PSEN1	rs63750322	chr14:73,603,143-73,690,399	AX-90069678
PSEN1	rs63750004	chr14:73,603,143-73,690,399	AX-90055602
PSEN1	rs63751071	chr14:73,603,143-73,690,399	AX-88796183
PSEN1	---	chr14:73,603,143-73,690,399	AX-90026718
PSEN1	---	chr14:73,603,143-73,690,399	AX-90064764
PSEN1	---	chr14:73,603,143-73,690,399	AX-90055604
PSEN1	---	chr14:73,603,143-73,690,399	AX-90064962
PSEN1	rs63750907	chr14:73,603,143-73,690,399	AX-88753129
PSEN1	rs142093869	chr14:73,603,143-73,690,399	AX-94369268
PSEN1	rs63750588	chr14:73,603,143-73,690,399	AX-88796161
PSEN1	rs63751292	chr14:73,603,143-73,690,399	AX-88796195
PSEN1	---	chr14:73,603,143-73,690,399	AX-90043838
PSEN1	rs214269	chr14:73,603,143-73,690,399	AX-92478882
PSEN1	rs214269	chr14:73,603,143-73,690,399	AX-92689905
PSEN1	rs362341	chr14:73,603,143-73,690,399	AX-12821709
PSEN1	rs11379162	chr14:73,603,143-73,690,399	AX-120527225
PSEN1	rs362361	chr14:73,603,143-73,690,399	AX-12558765
PSEN1	rs34940341	chr14:73,603,143-73,690,399	AX-121876348
PSEN1	rs34940341	chr14:73,603,143-73,690,399	AX-92530382
PSEN1	rs2333030	chr14:73,603,143-73,690,399	AX-39808077
PSEN1	rs10141217	chr14:73,603,143-73,690,399	AX-113020981
PSEN1	rs10141217	chr14:73,603,143-73,690,399	AX-113912260
PSEN1	rs214267	chr14:73,603,143-73,690,399	AX-39808079
PSEN1	rs12882830	chr14:73,603,143-73,690,399	AX-12821714
PSEN1	rs12587333	chr14:73,603,143-73,690,399	AX-113020982
PSEN1	rs12587333	chr14:73,603,143-73,690,399	AX-92519056
PSEN1	rs4899458	chr14:73,603,143-73,690,399	AX-31449101
PSEN1	rs61986889	chr14:73,603,143-73,690,399	AX-92521857
PSEN1	rs61986889	chr14:73,603,143-73,690,399	AX-92732880
PSEN1	rs214263	chr14:73,603,143-73,690,399	AX-151251845
PSEN1	rs214263	chr14:73,603,143-73,690,399	AX-156269306
PSEN1	rs8011748	chr14:73,603,143-73,690,399	AX-92579394
PSEN1	rs8011748	chr14:73,603,143-73,690,399	AX-92790417
PSEN1	rs12184969	chr14:73,603,143-73,690,399	AX-11241475
PSEN1	rs12184969	chr14:73,603,143-73,690,399	AX-156271335
PSEN1	rs63749885	chr14:73,603,143-73,690,399	AX-90069662
PSEN1	rs63750590	chr14:73,603,143-73,690,399	AX-90069695
PSEN1	rs63751010	chr14:73,603,143-73,690,399	AX-88796178
PSEN1	rs63751484	chr14:73,603,143-73,690,399	AX-88796199
PSEN1	rs63751458	chr14:73,603,143-73,690,399	AX-90082365
PSEN1	---	chr14:73,603,143-73,690,399	AX-90055605
PSEN1	---	chr14:73,603,143-73,690,399	AX-90060609
PSEN1	---	chr14:73,603,143-73,690,399	AX-90080645
PSEN1	rs63750418	chr14:73,603,143-73,690,399	AX-88796153
PSEN1	rs63751210	chr14:73,603,143-73,690,399	AX-88753146
PSEN1	rs63750577	chr14:73,603,143-73,690,399	AX-88796160
PSEN1	rs63750963	chr14:73,603,143-73,690,399	AX-88796175
PSEN1	rs63750299	chr14:73,603,143-73,690,399	AX-88753103
PSEN1	---	chr14:73,603,143-73,690,399	AX-90033399
PSEN1	rs63751025	chr14:73,603,143-73,690,399	AX-88796181

PSEN1	rs63750771	chr14:73,603,143-73,690,399	AX-90040815
PSEN1	rs63751068	chr14:73,603,143-73,690,399	AX-90069722
PSEN1	rs61986892	chr14:73,603,143-73,690,399	AX-31449117
PSEN1	rs7152131	chr14:73,603,143-73,690,399	AX-39808083
PSEN1	rs17125457	chr14:73,603,143-73,690,399	AX-11308329
PSEN1	---	chr14:73,603,143-73,690,399	AX-90036186
PSEN1	rs63750311	chr14:73,603,143-73,690,399	AX-88796150
PSEN1	rs112451138	chr14:73,603,143-73,690,399	AX-12821721
PSEN1	rs63750569	chr14:73,603,143-73,690,399	AX-88753114
PSEN1	---	chr14:73,603,143-73,690,399	AX-90063386
PSEN1	---	chr14:73,603,143-73,690,399	AX-90026723
PSEN1	rs63749880	chr14:73,603,143-73,690,399	AX-88796136
PSEN1	---	chr14:73,603,143-73,690,399	AX-90055609
PSEN1	---	chr14:73,603,143-73,690,399	AX-90055610
PSEN1	---	chr14:73,603,143-73,690,399	AX-90055611
PSEN1	---	chr14:73,603,143-73,690,399	AX-90060908
PSEN1	rs63751309	chr14:73,603,143-73,690,399	AX-88796196
PSEN1	rs63751003	chr14:73,603,143-73,690,399	AX-88753132
PSEN1	rs267606983	chr14:73,603,143-73,690,399	AX-90055612
PSEN1	rs63750444	chr14:73,603,143-73,690,399	AX-90069684
PSEN1	rs140064975	chr14:73,603,143-73,690,399	AX-94352511
PSEN1	rs63749987	chr14:73,603,143-73,690,399	AX-88753097
PSEN1	rs63750761	chr14:73,603,143-73,690,399	AX-88753120
PSEN1	rs63750009	chr14:73,603,143-73,690,399	AX-88753098
PSEN1	rs63751072	chr14:73,603,143-73,690,399	AX-88753139
PSEN1	---	chr14:73,603,143-73,690,399	AX-90026728
PSEN1	rs63750487	chr14:73,603,143-73,690,399	AX-90040800
PSEN1	rs63749961	chr14:73,603,143-73,690,399	AX-88796139
PSEN1	rs63749970	chr14:73,603,143-73,690,399	AX-88753096
PSEN1	rs63749836	chr14:73,603,143-73,690,399	AX-88753090
PSEN1	rs63750799	chr14:73,603,143-73,690,399	AX-90069704
PSEN1	---	chr14:73,603,143-73,690,399	AX-90026729
PSEN1	---	chr14:73,603,143-73,690,399	AX-90062779
PSEN1	rs63751024	chr14:73,603,143-73,690,399	AX-88753135
PSEN1	---	chr14:73,603,143-73,690,399	AX-90033264
PSEN1	rs63751130	chr14:73,603,143-73,690,399	AX-88796186
PSEN1	rs63749835	chr14:73,603,143-73,690,399	AX-88753089
PSEN1	---	chr14:73,603,143-73,690,399	AX-90026730
PSEN1	rs63750858	chr14:73,603,143-73,690,399	AX-90036675
PSEN1	---	chr14:73,603,143-73,690,399	AX-90026731
PSEN1	---	chr14:73,603,143-73,690,399	AX-90050298
PSEN1	rs63750888	chr14:73,603,143-73,690,399	AX-88753127
PSEN1	rs63750526	chr14:73,603,143-73,690,399	AX-88796156
PSEN1	rs63750634	chr14:73,603,143-73,690,399	AX-88796164
PSEN1	rs63751163	chr14:73,603,143-73,690,399	AX-88753145
PSEN1	---	chr14:73,603,143-73,690,399	AX-90026733
PSEN1	rs63751320	chr14:73,603,143-73,690,399	AX-88796197
PSEN1	rs112560505	chr14:73,603,143-73,690,399	AX-148795365
PSEN1	rs112560505	chr14:73,603,143-73,690,399	AX-156269307
PSEN1	rs17408630	chr14:73,603,143-73,690,399	AX-115156484
PSEN1	rs17408630	chr14:73,603,143-73,690,399	AX-156286987
PSEN1	rs142355580	chr14:73,603,143-73,690,399	AX-156285483
PSEN1	rs214262	chr14:73,603,143-73,690,399	AX-92416399
PSEN1	rs214262	chr14:73,603,143-73,690,399	AX-92627422
PSEN1	rs12587473	chr14:73,603,143-73,690,399	AX-148730168

PSEN1	rs12587473	chr14:73,603,143-73,690,399	AX-156266509
PSEN1	rs214261	chr14:73,603,143-73,690,399	AX-12821723
PSEN1	rs214260	chr14:73,603,143-73,690,399	AX-39808093
PSEN1	rs362342	chr14:73,603,143-73,690,399	AX-39808095
PSEN1	rs150191750	chr14:73,603,143-73,690,399	AX-156284626
PSEN1	rs8018739	chr14:73,603,143-73,690,399	AX-123030501
PSEN1	rs115702167	chr14:73,603,143-73,690,399	AX-148738516
PSEN1	rs115702167	chr14:73,603,143-73,690,399	AX-156278497
PSEN1	rs17125555	chr14:73,603,143-73,690,399	AX-31449137
PSEN1	rs63751420	chr14:73,603,143-73,690,399	AX-88796198
PSEN1	rs63750964	chr14:73,603,143-73,690,399	AX-88796176
PSEN1	---	chr14:73,603,143-73,690,399	AX-90062829
PSEN1	---	chr14:73,603,143-73,690,399	AX-90063445
PSEN1	rs63750543	chr14:73,603,143-73,690,399	AX-88796157
PSEN1	rs63751102	chr14:73,603,143-73,690,399	AX-88796185
PSEN1	rs63750301	chr14:73,603,143-73,690,399	AX-88796149
PSEN1	rs121917807	chr14:73,603,143-73,690,399	AX-88753519
PSEN1	rs63751229	chr14:73,603,143-73,690,399	AX-88753147
PSEN1	rs63750779	chr14:73,603,143-73,690,399	AX-88796167
PSEN1	rs63750900	chr14:73,603,143-73,690,399	AX-88796173
PSEN1	rs63750886	chr14:73,603,143-73,690,399	AX-88796171
PSEN1	rs63750772	chr14:73,603,143-73,690,399	AX-88796166
PSEN1	rs63750284	chr14:73,603,143-73,690,399	AX-88753101
PSEN1	---	chr14:73,603,143-73,690,399	AX-90055619
PSEN1	---	chr14:73,603,143-73,690,399	AX-90055834
PSEN1	rs63750524	chr14:73,603,143-73,690,399	AX-88796155
PSEN1	---	chr14:73,603,143-73,690,399	AX-90026735
PSEN1	rs114378630	chr14:73,603,143-73,690,399	AX-62213841
PSEN1	rs63749937	chr14:73,603,143-73,690,399	AX-88796138
PSEN1	---	chr14:73,603,143-73,690,399	AX-90029271
PSEN1	rs63750050	chr14:73,603,143-73,690,399	AX-88796144
PSEN1	rs63750324	chr14:73,603,143-73,690,399	AX-90040793
PSEN1	rs63750863	chr14:73,603,143-73,690,399	AX-88796170
PSEN1	rs63751139	chr14:73,603,143-73,690,399	AX-88753142
PSEN1	---	chr14:73,603,143-73,690,399	AX-90026737
PSEN1	rs165932	chr14:73,603,143-73,690,399	AX-39808101
PSEN1	rs165933	chr14:73,603,143-73,690,399	AX-12471965
PSEN1	rs362367	chr14:73,603,143-73,690,399	AX-12821726
PSEN1	rs140164309	chr14:73,603,143-73,690,399	AX-156269308
PSEN1	rs140164309	chr14:73,603,143-73,690,399	AX-98060526
PSEN1	rs56008848	chr14:73,603,143-73,690,399	AX-113238926
PSEN1	rs56008848	chr14:73,603,143-73,690,399	AX-156286988
PSEN1	rs142868766	chr14:73,603,143-73,690,399	AX-88790803
PSEN1	rs142868766	chr14:73,603,143-73,690,399	AX-92710437
PSEN1	rs13379148	chr14:73,603,143-73,690,399	AX-115156479
PSEN1	rs13379148	chr14:73,603,143-73,690,399	AX-156286989
PSEN1	rs12587379	chr14:73,603,143-73,690,399	AX-31449157
PSEN1	rs362372	chr14:73,603,143-73,690,399	AX-156280261
PSEN1	rs362372	chr14:73,603,143-73,690,399	AX-156295709
PSEN1	rs12894420	chr14:73,603,143-73,690,399	AX-11577798
PSEN1	rs12894420	chr14:73,603,143-73,690,399	AX-119793877
PSEN1	rs59341951	chr14:73,603,143-73,690,399	AX-148156158
PSEN1	rs59341951	chr14:73,603,143-73,690,399	AX-97912420
PSEN1	rs165934	chr14:73,603,143-73,690,399	AX-11280485
PSEN1	---	chr14:73,603,143-73,690,399	AX-90060341

PSEN1	rs63750298	chr14:73,603,143-73,690,399	AX-88753102
PSEN1	rs147638016	chr14:73,603,143-73,690,399	AX-94369274
PSEN1	rs17125721	chr14:73,603,143-73,690,399	AX-11308351
PSEN1	rs362374	chr14:73,603,143-73,690,399	AX-39808115
PSEN1	rs73305064	chr14:73,603,143-73,690,399	AX-12821730
PSEN1	rs61986901	chr14:73,603,143-73,690,399	AX-12821731
PSEN1	rs28607501	chr14:73,603,143-73,690,399	AX-92495505
PSEN1	rs28607501	chr14:73,603,143-73,690,399	AX-92706528
PSEN1	rs2280791	chr14:73,603,143-73,690,399	AX-31449167
PSEN1	rs28532025	chr14:73,603,143-73,690,399	AX-148789816
PSEN1	rs28532025	chr14:73,603,143-73,690,399	AX-156266510
PSEN1	rs12896793	chr14:73,603,143-73,690,399	AX-156286990
PSEN1	rs177415	chr14:73,603,143-73,690,399	AX-39808123
PSEN1	rs362377	chr14:73,603,143-73,690,399	AX-92545757
PSEN1	rs362377	chr14:73,603,143-73,690,399	AX-92756780
PSEN1	rs121917809	chr14:73,603,143-73,690,399	AX-86616242
PSEN1	rs138871096	chr14:73,603,143-73,690,399	AX-94352509
PSEN1	rs63750762	chr14:73,603,143-73,690,399	AX-90053477
PSEN1	rs149299753	chr14:73,603,143-73,690,399	AX-94352526
PSEN1	rs63751164	chr14:73,603,143-73,690,399	AX-84769307
PSEN1	rs143683769	chr14:73,603,143-73,690,399	AX-94352519
PSEN1	rs63751174	chr14:73,603,143-73,690,399	AX-83494347
PSEN1	rs63750941	chr14:73,603,143-73,690,399	AX-84690260
PSEN1	rs141822345	chr14:73,603,143-73,690,399	AX-94352517
PSEN1	---	chr14:73,603,143-73,690,399	AX-90035352
PSEN1	rs28660183	chr14:73,603,143-73,690,399	AX-92415532
PSEN1	rs28660183	chr14:73,603,143-73,690,399	AX-92626555
PSEN1	rs362343	chr14:73,603,143-73,690,399	AX-12821740
PSEN1	rs10146467	chr14:73,603,143-73,690,399	AX-88735361
PSEN1	rs10146467	chr14:73,603,143-73,690,399	AX-92584248
PSEN1	rs10146743	chr14:73,603,143-73,690,399	AX-92542443
PSEN1	rs10146743	chr14:73,603,143-73,690,399	AX-92753466
PSEN1	rs10140990	chr14:73,603,143-73,690,399	AX-12821746
PSEN1	rs177413	chr14:73,603,143-73,690,399	AX-92494005
PSEN1	rs177413	chr14:73,603,143-73,690,399	AX-92705028
PSEN1	rs2272585	chr14:73,603,143-73,690,399	AX-12821748
PSEN1	rs63751051	chr14:73,603,143-73,690,399	AX-88796182
PSEN1	---	chr14:73,603,143-73,690,399	AX-90055623
PSEN1	rs63750687	chr14:73,603,143-73,690,399	AX-84755534
PSEN1	rs63750646	chr14:73,603,143-73,690,399	AX-84786922
PSEN1	rs63749860	chr14:73,603,143-73,690,399	AX-84695010
PSEN1	rs63750883	chr14:73,603,143-73,690,399	AX-84729339
PSEN1	rs63751066	chr14:73,603,143-73,690,399	AX-84668611
PSEN1	rs63751416	chr14:73,603,143-73,690,399	AX-84766193
PSEN1	rs63750929	chr14:73,603,143-73,690,399	AX-84783734
PSEN1	rs146648448	chr14:73,603,143-73,690,399	AX-94380498
PSEN1	rs63751254	chr14:73,603,143-73,690,399	AX-84678589
PSEN1	rs63750227	chr14:73,603,143-73,690,399	AX-84778436
PSEN1	rs661	chr14:73,603,143-73,690,399	AX-84788645
PSEN1	---	chr14:73,603,143-73,690,399	AX-90029001
PSEN1	rs7147079	chr14:73,603,143-73,690,399	AX-92477405
PSEN1	rs7147079	chr14:73,603,143-73,690,399	AX-92688428
PSEN1	rs63751316	chr14:73,603,143-73,690,399	AX-84687900
PSEN1	rs63750802	chr14:73,603,143-73,690,399	AX-84787856
PSEN1	---	chr14:73,603,143-73,690,399	AX-90055624

PSEN1	---	chr14:73,603,143-73,690,399	AX-90034533
PSEN1	rs63751223	chr14:73,603,143-73,690,399	AX-84768237
PSEN1	---	chr14:73,603,143-73,690,399	AX-90055626
PSEN1	rs63750001	chr14:73,603,143-73,690,399	AX-84777429
PSEN1	rs63749925	chr14:73,603,143-73,690,399	AX-84749733
PSEN1	rs121917808	chr14:73,603,143-73,690,399	AX-88753008
PSEN1	rs63750249	chr14:73,603,143-73,690,399	AX-84771644
PSEN1	---	chr14:73,603,143-73,690,399	AX-90026742
PSEN1	---	chr14:73,603,143-73,690,399	AX-90079627
PSEN1	rs63750470	chr14:73,603,143-73,690,399	AX-94363748
PSEN1	rs140169796	chr14:73,603,143-73,690,399	AX-94369262
PSEN1	rs362385	chr14:73,603,143-73,690,399	AX-11475791
PSEN1	rs7523	chr14:73,603,143-73,690,399	AX-11627422
PSEN1	rs165935	chr14:73,603,143-73,690,399	AX-39808145
PSEN1	rs362393	chr14:73,603,143-73,690,399	AX-92431910
PSEN1	rs362393	chr14:73,603,143-73,690,399	AX-92642933
PSEN1	rs362344	chr14:73,603,143-73,690,399	AX-12821756
PSEN2	rs6660082	chr1:227,058,273-227,083,804	AX-156270755
PSEN2	rs6660082	chr1:227,058,273-227,083,804	AX-156289482
PSEN2	rs1295645	chr1:227,058,273-227,083,804	AX-39373591
PSEN2	rs1296171	chr1:227,058,273-227,083,804	AX-30651861
PSEN2	rs1295646	chr1:227,058,273-227,083,804	AX-30651865
PSEN2	rs1297990	chr1:227,058,273-227,083,804	AX-39373599
PSEN2	rs7523790	chr1:227,058,273-227,083,804	AX-156270756
PSEN2	rs7523790	chr1:227,058,273-227,083,804	AX-156289483
PSEN2	rs1295647	chr1:227,058,273-227,083,804	AX-148018378
PSEN2	rs1295647	chr1:227,058,273-227,083,804	AX-92416116
PSEN2	rs1295648	chr1:227,058,273-227,083,804	AX-148438298
PSEN2	rs4653469	chr1:227,058,273-227,083,804	AX-39373603
PSEN2	rs11588886	chr1:227,058,273-227,083,804	AX-30651879
PSEN2	rs7522645	chr1:227,058,273-227,083,804	AX-30651881
PSEN2	rs1295621	chr1:227,058,273-227,083,804	AX-30651883
PSEN2	rs6661000	chr1:227,058,273-227,083,804	AX-39373605
PSEN2	rs2793461	chr1:227,058,273-227,083,804	AX-11410735
PSEN2	rs6675452	chr1:227,058,273-227,083,804	AX-30651893
PSEN2	rs6678839	chr1:227,058,273-227,083,804	AX-30651897
PSEN2	rs6678940	chr1:227,058,273-227,083,804	AX-39373607
PSEN2	rs6671059	chr1:227,058,273-227,083,804	AX-16912096
PSEN2	rs12760155	chr1:227,058,273-227,083,804	AX-156270757
PSEN2	rs2073488	chr1:227,058,273-227,083,804	AX-156270758
PSEN2	rs7961	chr1:227,058,273-227,083,804	AX-39373611
PSEN2	rs2793462	chr1:227,058,273-227,083,804	AX-156270759
PSEN2	rs2793462	chr1:227,058,273-227,083,804	AX-156289486
PSEN2	rs2073489	chr1:227,058,273-227,083,804	AX-16912099
PSEN2	rs78557397	chr1:227,058,273-227,083,804	AX-30651915
PSEN2	rs76766807	chr1:227,058,273-227,083,804	AX-30651917
PSEN2	rs139498276	chr1:227,058,273-227,083,804	AX-151428473
PSEN2	rs139498276	chr1:227,058,273-227,083,804	AX-156286408
PSEN2	rs7525819	chr1:227,058,273-227,083,804	AX-39373617
PSEN2	rs732479	chr1:227,058,273-227,083,804	AX-39373619
PSEN2	rs17391089	chr1:227,058,273-227,083,804	AX-39373623
PSEN2	rs6677801	chr1:227,058,273-227,083,804	AX-156279962
PSEN2	rs6677801	chr1:227,058,273-227,083,804	AX-156295511
PSEN2	rs6677908	chr1:227,058,273-227,083,804	AX-148519587
PSEN2	rs6677908	chr1:227,058,273-227,083,804	AX-156279963

PSEN2	rs111567390	chr1:227,058,273-227,083,804	AX-30651951
PSEN2	rs79540408	chr1:227,058,273-227,083,804	AX-16912120
PSEN2	rs1295638	chr1:227,058,273-227,083,804	AX-115156491
PSEN2	rs1295638	chr1:227,058,273-227,083,804	AX-156289487
PSEN2	---	chr1:227,058,273-227,083,804	AX-83184160
PSEN2	rs143061887	chr1:227,058,273-227,083,804	AX-94370125
PSEN2	rs11405	chr1:227,058,273-227,083,804	AX-12412739
PSEN2	rs149354305	chr1:227,058,273-227,083,804	AX-94370151
PSEN2	rs6759	chr1:227,058,273-227,083,804	AX-39373635
PSEN2	rs12071324	chr1:227,058,273-227,083,804	AX-30651959
PSEN2	rs1295640	chr1:227,058,273-227,083,804	AX-39373639
PSEN2	rs1295641	chr1:227,058,273-227,083,804	AX-30651967
PSEN2	rs1295642	chr1:227,058,273-227,083,804	AX-30651975
PSEN2	rs1295643	chr1:227,058,273-227,083,804	AX-30651979
PSEN2	rs1295644	chr1:227,058,273-227,083,804	AX-30651981
PSEN2	rs58973334	chr1:227,058,273-227,083,804	AX-83424521
PSEN2	rs63750048	chr1:227,058,273-227,083,804	AX-90041501
PSEN2	rs1046240	chr1:227,058,273-227,083,804	AX-39373641
PSEN2	rs12057618	chr1:227,058,273-227,083,804	AX-156270760
PSEN2	rs12057618	chr1:227,058,273-227,083,804	AX-156289488
PSEN2	rs35519961	chr1:227,058,273-227,083,804	AX-30651991
PSEN2	rs12747261	chr1:227,058,273-227,083,804	AX-30651993
PSEN2	rs12401969	chr1:227,058,273-227,083,804	AX-156270761
PSEN2	rs1782530	chr1:227,058,273-227,083,804	AX-30652001
PSEN2	rs58855513	chr1:227,058,273-227,083,804	AX-30652003
PSEN2	rs76732250	chr1:227,058,273-227,083,804	AX-156270762
PSEN2	rs76732250	chr1:227,058,273-227,083,804	AX-156289490
PSEN2	rs63750197	chr1:227,058,273-227,083,804	AX-83483186
PSEN2	rs202178897	chr1:227,058,273-227,083,804	AX-86552974
PSEN2	rs63750215	chr1:227,058,273-227,083,804	AX-88797837
PSEN2	rs63750812	chr1:227,058,273-227,083,804	AX-94370063
PSEN2	rs139573101	chr1:227,058,273-227,083,804	AX-94370109
PSEN2	---	chr1:227,058,273-227,083,804	AX-30652011
PSEN2	rs1794038	chr1:227,058,273-227,083,804	AX-148006059
PSEN2	rs1794038	chr1:227,058,273-227,083,804	AX-153818386
PSEN2	rs199899855	chr1:227,058,273-227,083,804	AX-156286456
PSEN2	rs2236912	chr1:227,058,273-227,083,804	AX-119837848
PSEN2	rs2236912	chr1:227,058,273-227,083,804	AX-156289491
PSEN2	rs12069540	chr1:227,058,273-227,083,804	AX-30652019
PSEN2	rs2236913	chr1:227,058,273-227,083,804	AX-39373645
PSEN2	rs74502648	chr1:227,058,273-227,083,804	AX-30652027
PSEN2	rs2246221	chr1:227,058,273-227,083,804	AX-39373653
PSEN2	---	chr1:227,058,273-227,083,804	AX-90026427
PSEN2	rs1800680	chr1:227,058,273-227,083,804	AX-113897998
PSEN2	rs1800680	chr1:227,058,273-227,083,804	AX-113971836
PSEN2	rs1800681	chr1:227,058,273-227,083,804	AX-39373657
PSEN2	rs63750880	chr1:227,058,273-227,083,804	AX-88754793
PSEN2	rs61730652	chr1:227,058,273-227,083,804	AX-16912152
PSEN2	rs63749884	chr1:227,058,273-227,083,804	AX-88797836
PSEN2	rs140562931	chr1:227,058,273-227,083,804	AX-94370112
PSEN2	---	chr1:227,058,273-227,083,804	AX-92812211
PSEN2	rs7539119	chr1:227,058,273-227,083,804	AX-30652035
PSEN2	---	chr1:227,058,273-227,083,804	AX-39373661
PSEN2	rs150780392	chr1:227,058,273-227,083,804	AX-94370164
PSEN2	rs11804915	chr1:227,058,273-227,083,804	AX-156270763

PSEN2	rs11804915	chr1:227,058,273-227,083,804	AX-156289492
PSEN2	rs6680042	chr1:227,058,273-227,083,804	AX-30652051
PSEN2	rs41309643	chr1:227,058,273-227,083,804	AX-50090037
PSEN2	rs1800678	chr1:227,058,273-227,083,804	AX-16912159
PSEN2	rs2236914	chr1:227,058,273-227,083,804	AX-39373665
PSEN2	---	chr1:227,058,273-227,083,804	AX-39373667
PSEN2	rs6426553	chr1:227,058,273-227,083,804	AX-30652057
PSEN2	rs565698726	chr1:227,058,273-227,083,804	AX-94353366
PSEN2	rs2236915	chr1:227,058,273-227,083,804	AX-39373673
PSEN2	rs7537037	chr1:227,058,273-227,083,804	AX-156270764
PSEN2	rs7537037	chr1:227,058,273-227,083,804	AX-156289493
PSEN2	rs63750207	chr1:227,058,273-227,083,804	AX-94370061
PSEN2	---	chr1:227,058,273-227,083,804	AX-90029405
PSEN2	rs12130732	chr1:227,058,273-227,083,804	AX-156270765
PSEN2	rs12130732	chr1:227,058,273-227,083,804	AX-156289494
PSEN2	rs75612539	chr1:227,058,273-227,083,804	AX-30652067
PSEN2	rs12123818	chr1:227,058,273-227,083,804	AX-30652071
PSEN2	rs743601	chr1:227,058,273-227,083,804	AX-30652073
PSEN2	rs743602	chr1:227,058,273-227,083,804	AX-30652079
PSEN2	rs3820649	chr1:227,058,273-227,083,804	AX-156270766
PSEN2	rs3820649	chr1:227,058,273-227,083,804	AX-156289495
PSEN2	rs2855559	chr1:227,058,273-227,083,804	AX-156270767
PSEN2	rs10693643	chr1:227,058,273-227,083,804	AX-151363373
PSEN2	rs10693643	chr1:227,058,273-227,083,804	AX-156284386
PSEN2	rs2105822	chr1:227,058,273-227,083,804	AX-30652093
PSEN2	rs2855560	chr1:227,058,273-227,083,804	AX-30652095
PSEN2	rs3213436	chr1:227,058,273-227,083,804	AX-39373679
PSEN2	rs10753428	chr1:227,058,273-227,083,804	AX-112922257
PSEN2	rs10753428	chr1:227,058,273-227,083,804	AX-113961642
PSEN2	rs145671982	chr1:227,058,273-227,083,804	AX-94370138
PSEN2	rs2855562	chr1:227,058,273-227,083,804	AX-30652107
PSEN2	rs3829979	chr1:227,058,273-227,083,804	AX-30652111
PSEN2	rs2855563	chr1:227,058,273-227,083,804	AX-16912173
PSEN2	rs6426554	chr1:227,058,273-227,083,804	AX-39373681
PSEN2	rs7555739	chr1:227,058,273-227,083,804	AX-156279964
PSEN2	rs7555739	chr1:227,058,273-227,083,804	AX-156295512
PSEN2	rs7512986	chr1:227,058,273-227,083,804	AX-156279965
PSEN2	rs7512986	chr1:227,058,273-227,083,804	AX-156295513
PSEN2	rs4653470	chr1:227,058,273-227,083,804	AX-39373687
PSEN2	rs63750666	chr1:227,058,273-227,083,804	AX-86718019
PSEN2	rs142772982	chr1:227,058,273-227,083,804	AX-94353372
PSEN2	rs201922151	chr1:227,058,273-227,083,804	AX-86634258
PSEN2	rs63750110	chr1:227,058,273-227,083,804	AX-86600929
RAF1	rs1051208	chr3:12,625,100-12,705,700	AX-12392606
RAF1	---	chr3:12,625,100-12,705,700	AX-91984589
RAF1	rs3729931	chr3:12,625,100-12,705,700	AX-11476415
RAF1	rs397516815	chr3:12,625,100-12,705,700	AX-92013606
RAF1	rs80338798	chr3:12,625,100-12,705,700	AX-91994419
RAF1	---	chr3:12,625,100-12,705,700	AX-91976560
RAF1	rs2290159	chr3:12,625,100-12,705,700	AX-14107485
RAF1	---	chr3:12,625,100-12,705,700	AX-92022232
RAF1	---	chr3:12,625,100-12,705,700	AX-91978705
RAF1	rs2290161	chr3:12,625,100-12,705,700	AX-12524527
RAF1	rs904464	chr3:12,625,100-12,705,700	AX-14107740
RAF1	rs397516829	chr3:12,625,100-12,705,700	AX-92012335

RAF1	---	chr3:12,625,100-12,705,700	AX-92000871
RAF1	---	chr3:12,625,100-12,705,700	AX-91986307
RAF1	rs80338796	chr3:12,625,100-12,705,700	AX-91979703
RAF1	---	chr3:12,625,100-12,705,700	AX-91988305
RAF1	---	chr3:12,625,100-12,705,700	AX-94357126
RAF1	---	chr3:12,625,100-12,705,700	AX-94373877
RAF1	---	chr3:12,625,100-12,705,700	AX-94378269
RAF1	rs9817675	chr3:12,625,100-12,705,700	AX-12668391
RBM20	rs7091655	chr10:112,404,155-112,599,227	AX-14778949
RBM20	rs7915361	chr10:112,404,155-112,599,227	AX-14778958
RBM20	rs4488129	chr10:112,404,155-112,599,227	AX-29424783
RBM20	rs72834018	chr10:112,404,155-112,599,227	AX-29424803
RBM20	rs11591881	chr10:112,404,155-112,599,227	AX-29424913
RBM20	rs4918560	chr10:112,404,155-112,599,227	AX-29424963
RBM20	rs112860361	chr10:112,404,155-112,599,227	AX-29424967
RBM20	rs75379819	chr10:112,404,155-112,599,227	AX-29425003
RBM20	rs957307	chr10:112,404,155-112,599,227	AX-38717999
RBM20	rs10885025	chr10:112,404,155-112,599,227	AX-12399700
RBM20	rs4342957	chr10:112,404,155-112,599,227	AX-11501892
RBM20	rs10749049	chr10:112,404,155-112,599,227	AX-11118570
RBM20	rs12764660	chr10:112,404,155-112,599,227	AX-11226301
RBM20	rs11195294	chr10:112,404,155-112,599,227	AX-38718083
RBM20	rs118030849	chr10:112,404,155-112,599,227	AX-29425117
RBM20	rs10885030	chr10:112,404,155-112,599,227	AX-29425119
RBM20	rs71481106	chr10:112,404,155-112,599,227	AX-14781397
RBM20	rs1113798	chr10:112,404,155-112,599,227	AX-29425155
RBM20	rs1885790	chr10:112,404,155-112,599,227	AX-14781840
RBM20	rs117234122	chr10:112,404,155-112,599,227	AX-29425201
RBM20	rs34376649	chr10:112,404,155-112,599,227	AX-29425203
RBM20	rs72823883	chr10:112,404,155-112,599,227	AX-29425231
RBM20	rs7069694	chr10:112,404,155-112,599,227	AX-11604699
RBM20	rs2181407	chr10:112,404,155-112,599,227	AX-11373061
RBM20	rs536357058	chr10:112,404,155-112,599,227	AX-90034716
RBM20	rs61735268	chr10:112,404,155-112,599,227	AX-14782494
RBM20	rs201148126	chr10:112,404,155-112,599,227	AX-83594630
RBM20	rs61862866	chr10:112,404,155-112,599,227	AX-29425287
RBM20	rs12254016	chr10:112,404,155-112,599,227	AX-14782520
RBM20	rs61735272	chr10:112,404,155-112,599,227	AX-83310107
RBM20	rs6585012	chr10:112,404,155-112,599,227	AX-11573698
RBM20	rs55873340	chr10:112,404,155-112,599,227	AX-29425297
RBM20	rs189569984	chr10:112,404,155-112,599,227	AX-83176076
RBM20	rs11818323	chr10:112,404,155-112,599,227	AX-38718221
RBM20	rs1570427	chr10:112,404,155-112,599,227	AX-11276640
RBM20	rs12357780	chr10:112,404,155-112,599,227	AX-38718233
RBM20	rs55909737	chr10:112,404,155-112,599,227	AX-29425341
RBM20	rs76048624	chr10:112,404,155-112,599,227	AX-14782838
RBM20	rs183007628	chr10:112,404,155-112,599,227	AX-90067624
RBM20	rs74853168	chr10:112,404,155-112,599,227	AX-14782848
RBM20	rs117325635	chr10:112,404,155-112,599,227	AX-14782869
RBM20	rs12354559	chr10:112,404,155-112,599,227	AX-11201955
RBM20	rs12781566	chr10:112,404,155-112,599,227	AX-38718267
RBM20	rs11195329	chr10:112,404,155-112,599,227	AX-11150012
RBM20	rs11195330	chr10:112,404,155-112,599,227	AX-11150013
RBM20	rs59523266	chr10:112,404,155-112,599,227	AX-29425369
RBM20	rs75497793	chr10:112,404,155-112,599,227	AX-29425373

RBM20	rs2031130	chr10:112,404,155-112,599,227	AX-11361290
RBM20	rs2146546	chr10:112,404,155-112,599,227	AX-29425377
RBM20	rs6585015	chr10:112,404,155-112,599,227	AX-11573699
RBM20	rs34038942	chr10:112,404,155-112,599,227	AX-29425409
RBM20	rs10885051	chr10:112,404,155-112,599,227	AX-29425415
RBM20	---	chr10:112,404,155-112,599,227	AX-90026445
RBM20	rs267607001	chr10:112,404,155-112,599,227	AX-90055331
RBM20	---	chr10:112,404,155-112,599,227	AX-90059778
RBM20	---	chr10:112,404,155-112,599,227	AX-90055332
RBM20	rs267607004	chr10:112,404,155-112,599,227	AX-90055333
RBM20	rs267607005	chr10:112,404,155-112,599,227	AX-90026449
RBM20	rs267607003	chr10:112,404,155-112,599,227	AX-90026450
RBM20	rs74339620	chr10:112,404,155-112,599,227	AX-14783130
RBM20	rs375798246	chr10:112,404,155-112,599,227	AX-86647071
RBM20	rs61862917	chr10:112,404,155-112,599,227	AX-29425423
RBM20	rs61862919	chr10:112,404,155-112,599,227	AX-29425431
RBM20	rs10749053	chr10:112,404,155-112,599,227	AX-38718307
RBM20	rs201370621	chr10:112,404,155-112,599,227	AX-86645675
RBM20	---	chr10:112,404,155-112,599,227	AX-90061254
RBM20	rs75858380	chr10:112,404,155-112,599,227	AX-14783283
RBM20	---	chr10:112,404,155-112,599,227	AX-90033769
RBM20	rs116908219	chr10:112,404,155-112,599,227	AX-83398500
RBM20	rs12354578	chr10:112,404,155-112,599,227	AX-11201956
RBM20	rs61862943	chr10:112,404,155-112,599,227	AX-14783391
RBM20	rs74158134	chr10:112,404,155-112,599,227	AX-14783444
RBM20	rs17831429	chr10:112,404,155-112,599,227	AX-38718331
RBM20	rs10885054	chr10:112,404,155-112,599,227	AX-38718333
RBM20	rs4525160	chr10:112,404,155-112,599,227	AX-29425519
RBM20	rs17831542	chr10:112,404,155-112,599,227	AX-38718339
RBM20	rs11195346	chr10:112,404,155-112,599,227	AX-29425523
RBM20	rs17764171	chr10:112,404,155-112,599,227	AX-29425533
RBM20	---	chr10:112,404,155-112,599,227	AX-90027815
RBM20	rs942077	chr10:112,404,155-112,599,227	AX-38718343
RBM20	rs77555440	chr10:112,404,155-112,599,227	AX-29425537
RBM20	rs79603535	chr10:112,404,155-112,599,227	AX-29425539
RYR2	rs1406413	chr1:237,205,702-237,997,288	AX-11260561
RYR2	rs74146692	chr1:237,205,702-237,997,288	AX-16948625
RYR2	rs78437107	chr1:237,205,702-237,997,288	AX-16948628
RYR2	rs4659488	chr1:237,205,702-237,997,288	AX-39410433
RYR2	rs10925301	chr1:237,205,702-237,997,288	AX-11132003
RYR2	rs10754590	chr1:237,205,702-237,997,288	AX-30722127
RYR2	rs72764017	chr1:237,205,702-237,997,288	AX-16948638
RYR2	rs74467938	chr1:237,205,702-237,997,288	AX-16948641
RYR2	rs12129023	chr1:237,205,702-237,997,288	AX-16948647
RYR2	rs9428372	chr1:237,205,702-237,997,288	AX-16948666
RYR2	rs114831995	chr1:237,205,702-237,997,288	AX-16948667
RYR2	rs12037993	chr1:237,205,702-237,997,288	AX-11187572
RYR2	rs12022498	chr1:237,205,702-237,997,288	AX-30722191
RYR2	rs75606339	chr1:237,205,702-237,997,288	AX-30722193
RYR2	rs76090957	chr1:237,205,702-237,997,288	AX-16948681
RYR2	rs78117705	chr1:237,205,702-237,997,288	AX-16948695
RYR2	rs59173404	chr1:237,205,702-237,997,288	AX-30722211
RYR2	rs9428374	chr1:237,205,702-237,997,288	AX-11687027
RYR2	rs10802587	chr1:237,205,702-237,997,288	AX-11122972
RYR2	rs12118388	chr1:237,205,702-237,997,288	AX-30722221

RYR2	rs72764032	chr1:237,205,702-237,997,288	AX-30722233
RYR2	rs12733526	chr1:237,205,702-237,997,288	AX-30722243
RYR2	rs74892437	chr1:237,205,702-237,997,288	AX-37446009
RYR2	rs6428991	chr1:237,205,702-237,997,288	AX-30722257
RYR2	rs7529190	chr1:237,205,702-237,997,288	AX-39410545
RYR2	rs7551672	chr1:237,205,702-237,997,288	AX-11629226
RYR2	rs7554607	chr1:237,205,702-237,997,288	AX-11629395
RYR2	rs12736282	chr1:237,205,702-237,997,288	AX-30722309
RYR2	rs2039689	chr1:237,205,702-237,997,288	AX-30722311
RYR2	rs2490346	chr1:237,205,702-237,997,288	AX-30722313
RYR2	rs1415714	chr1:237,205,702-237,997,288	AX-11261409
RYR2	rs75079782	chr1:237,205,702-237,997,288	AX-16948756
RYR2	rs2485598	chr1:237,205,702-237,997,288	AX-11394474
RYR2	rs11583937	chr1:237,205,702-237,997,288	AX-11160306
RYR2	rs9287218	chr1:237,205,702-237,997,288	AX-16948760
RYR2	rs12130378	chr1:237,205,702-237,997,288	AX-16948761
RYR2	rs77980124	chr1:237,205,702-237,997,288	AX-30722363
RYR2	rs114604390	chr1:237,205,702-237,997,288	AX-16948773
RYR2	rs2485567	chr1:237,205,702-237,997,288	AX-11394468
RYR2	rs2485569	chr1:237,205,702-237,997,288	AX-30722377
RYR2	rs6693720	chr1:237,205,702-237,997,288	AX-39410603
RYR2	rs2485572	chr1:237,205,702-237,997,288	AX-30722393
RYR2	rs115060574	chr1:237,205,702-237,997,288	AX-37446033
RYR2	rs2490380	chr1:237,205,702-237,997,288	AX-30722399
RYR2	rs12139096	chr1:237,205,702-237,997,288	AX-30722419
RYR2	rs12142661	chr1:237,205,702-237,997,288	AX-11192756
RYR2	rs6670388	chr1:237,205,702-237,997,288	AX-30722451
RYR2	rs1361115	chr1:237,205,702-237,997,288	AX-16948837
RYR2	rs1415716	chr1:237,205,702-237,997,288	AX-11261410
RYR2	rs75317377	chr1:237,205,702-237,997,288	AX-30722527
RYR2	rs57868848	chr1:237,205,702-237,997,288	AX-16948850
RYR2	rs114411755	chr1:237,205,702-237,997,288	AX-30722561
RYR2	rs2490356	chr1:237,205,702-237,997,288	AX-16948862
RYR2	rs17671324	chr1:237,205,702-237,997,288	AX-11334324
RYR2	rs77928911	chr1:237,205,702-237,997,288	AX-30722589
RYR2	rs6680857	chr1:237,205,702-237,997,288	AX-16948894
RYR2	rs61832394	chr1:237,205,702-237,997,288	AX-30722591
RYR2	rs78538843	chr1:237,205,702-237,997,288	AX-16948929
RYR2	rs78578141	chr1:237,205,702-237,997,288	AX-16948930
RYR2	rs12724105	chr1:237,205,702-237,997,288	AX-30722613
RYR2	rs683787	chr1:237,205,702-237,997,288	AX-30722631
RYR2	rs587020	chr1:237,205,702-237,997,288	AX-30722647
RYR2	rs10158020	chr1:237,205,702-237,997,288	AX-30722667
RYR2	rs115909403	chr1:237,205,702-237,997,288	AX-16948949
RYR2	rs78500791	chr1:237,205,702-237,997,288	AX-30722687
RYR2	rs2152884	chr1:237,205,702-237,997,288	AX-11370830
RYR2	rs115659895	chr1:237,205,702-237,997,288	AX-94348732
RYR2	rs17626494	chr1:237,205,702-237,997,288	AX-11331939
RYR2	rs12353981	chr1:237,205,702-237,997,288	AX-11201936
RYR2	rs61832416	chr1:237,205,702-237,997,288	AX-30722727
RYR2	rs12407646	chr1:237,205,702-237,997,288	AX-16948979
RYR2	rs72764081	chr1:237,205,702-237,997,288	AX-30722753
RYR2	rs11587851	chr1:237,205,702-237,997,288	AX-12414075
RYR2	rs652792	chr1:237,205,702-237,997,288	AX-11569788
RYR2	rs6663668	chr1:237,205,702-237,997,288	AX-11576953

RYR2	rs581693	chr1:237,205,702-237,997,288	AX-30722779
RYR2	rs75804850	chr1:237,205,702-237,997,288	AX-16949017
RYR2	rs114721952	chr1:237,205,702-237,997,288	AX-30722791
RYR2	rs268779	chr1:237,205,702-237,997,288	AX-11405086
RYR2	rs10925349	chr1:237,205,702-237,997,288	AX-11132006
RYR2	rs585717	chr1:237,205,702-237,997,288	AX-30722807
RYR2	rs11810170	chr1:237,205,702-237,997,288	AX-12420083
RYR2	rs75678262	chr1:237,205,702-237,997,288	AX-30722819
RYR2	rs16835048	chr1:237,205,702-237,997,288	AX-12473158
RYR2	---	chr1:237,205,702-237,997,288	AX-11576960
RYR2	rs10925354	chr1:237,205,702-237,997,288	AX-16949057
RYR2	rs72764090	chr1:237,205,702-237,997,288	AX-30722829
RYR2	rs17627448	chr1:237,205,702-237,997,288	AX-39410851
RYR2	rs10495392	chr1:237,205,702-237,997,288	AX-39410853
RYR2	rs10495394	chr1:237,205,702-237,997,288	AX-11111147
RYR2	rs4659491	chr1:237,205,702-237,997,288	AX-39410873
RYR2	rs7519473	chr1:237,205,702-237,997,288	AX-11627182
RYR2	rs61832457	chr1:237,205,702-237,997,288	AX-30722861
RYR2	rs76592404	chr1:237,205,702-237,997,288	AX-63949296
RYR2	rs7537963	chr1:237,205,702-237,997,288	AX-12631370
RYR2	rs2860125	chr1:237,205,702-237,997,288	AX-30722899
RYR2	rs6686679	chr1:237,205,702-237,997,288	AX-30722903
RYR2	rs10802597	chr1:237,205,702-237,997,288	AX-11122973
RYR2	rs12024815	chr1:237,205,702-237,997,288	AX-50104262
RYR2	rs116207040	chr1:237,205,702-237,997,288	AX-37446109
RYR2	rs10754597	chr1:237,205,702-237,997,288	AX-39410927
RYR2	rs6682878	chr1:237,205,702-237,997,288	AX-30722961
RYR2	rs12164577	chr1:237,205,702-237,997,288	AX-30722977
RYR2	rs12741336	chr1:237,205,702-237,997,288	AX-39410951
RYR2	rs2543036	chr1:237,205,702-237,997,288	AX-16949159
RYR2	rs12755424	chr1:237,205,702-237,997,288	AX-11225945
RYR2	rs72765924	chr1:237,205,702-237,997,288	AX-16949168
RYR2	rs35588486	chr1:237,205,702-237,997,288	AX-39410969
RYR2	rs74659606	chr1:237,205,702-237,997,288	AX-16949177
RYR2	rs75894029	chr1:237,205,702-237,997,288	AX-16949178
RYR2	rs116553681	chr1:237,205,702-237,997,288	AX-16949181
RYR2	rs974893	chr1:237,205,702-237,997,288	AX-12667329
RYR2	rs7535911	chr1:237,205,702-237,997,288	AX-11628216
RYR2	rs73127292	chr1:237,205,702-237,997,288	AX-16949200
RYR2	rs16835142	chr1:237,205,702-237,997,288	AX-39410973
RYR2	---	chr1:237,205,702-237,997,288	AX-94371235
RYR2	rs6429005	chr1:237,205,702-237,997,288	AX-11563406
RYR2	rs10925374	chr1:237,205,702-237,997,288	AX-11132008
RYR2	rs80065345	chr1:237,205,702-237,997,288	AX-16949235
RYR2	rs77704048	chr1:237,205,702-237,997,288	AX-16949239
RYR2	rs12026757	chr1:237,205,702-237,997,288	AX-30723117
RYR2	rs4475735	chr1:237,205,702-237,997,288	AX-11507162
RYR2	rs17681893	chr1:237,205,702-237,997,288	AX-57110260
RYR2	rs77936709	chr1:237,205,702-237,997,288	AX-37446159
RYR2	rs79510842	chr1:237,205,702-237,997,288	AX-16949285
RYR2	---	chr1:237,205,702-237,997,288	AX-94373953
RYR2	rs397516539	chr1:237,205,702-237,997,288	AX-91986469
RYR2	---	chr1:237,205,702-237,997,288	AX-94371236
RYR2	rs201211033	chr1:237,205,702-237,997,288	AX-86548883
RYR2	---	chr1:237,205,702-237,997,288	AX-94354487

RYR2	rs72765933	chr1:237,205,702-237,997,288	AX-30723191
RYR2	rs10925394	chr1:237,205,702-237,997,288	AX-11132011
RYR2	rs17682073	chr1:237,205,702-237,997,288	AX-11334873
RYR2	rs4659786	chr1:237,205,702-237,997,288	AX-11515874
RYR2	---	chr1:237,205,702-237,997,288	AX-94373954
RYR2	---	chr1:237,205,702-237,997,288	AX-94371238
RYR2	---	chr1:237,205,702-237,997,288	AX-94371239
RYR2	rs79447782	chr1:237,205,702-237,997,288	AX-30723207
RYR2	rs12078819	chr1:237,205,702-237,997,288	AX-11189498
RYR2	rs74147258	chr1:237,205,702-237,997,288	AX-16949328
RYR2	rs4659787	chr1:237,205,702-237,997,288	AX-11515875
RYR2	rs56369530	chr1:237,205,702-237,997,288	AX-30723221
RYR2	rs115179216	chr1:237,205,702-237,997,288	AX-30723227
RYR2	rs114119896	chr1:237,205,702-237,997,288	AX-37446173
RYR2	rs12569314	chr1:237,205,702-237,997,288	AX-16949344
RYR2	rs35968847	chr1:237,205,702-237,997,288	AX-11472907
RYR2	rs4659792	chr1:237,205,702-237,997,288	AX-16949372
RYR2	rs13376665	chr1:237,205,702-237,997,288	AX-11251495
RYR2	rs12691536	chr1:237,205,702-237,997,288	AX-12445672
RYR2	rs72765955	chr1:237,205,702-237,997,288	AX-30723299
RYR2	rs6673187	chr1:237,205,702-237,997,288	AX-16949384
RYR2	---	chr1:237,205,702-237,997,288	AX-94354490
RYR2	---	chr1:237,205,702-237,997,288	AX-94354491
RYR2	rs4659794	chr1:237,205,702-237,997,288	AX-12576860
RYR2	rs7532079	chr1:237,205,702-237,997,288	AX-57110375
RYR2	rs12122723	chr1:237,205,702-237,997,288	AX-16949405
RYR2	rs7552945	chr1:237,205,702-237,997,288	AX-12631819
RYR2	rs60811417	chr1:237,205,702-237,997,288	AX-16949418
RYR2	rs10925414	chr1:237,205,702-237,997,288	AX-11132013
RYR2	rs17633019	chr1:237,205,702-237,997,288	AX-11332264
RYR2	rs11803630	chr1:237,205,702-237,997,288	AX-11176607
RYR2	rs12139603	chr1:237,205,702-237,997,288	AX-11192564
RYR2	---	chr1:237,205,702-237,997,288	AX-94354492
RYR2	---	chr1:237,205,702-237,997,288	AX-11189786
RYR2	---	chr1:237,205,702-237,997,288	AX-94357205
RYR2	---	chr1:237,205,702-237,997,288	AX-94354493
RYR2	---	chr1:237,205,702-237,997,288	AX-92791863
RYR2	---	chr1:237,205,702-237,997,288	AX-94373956
RYR2	---	chr1:237,205,702-237,997,288	AX-94371244
RYR2	---	chr1:237,205,702-237,997,288	AX-94373957
RYR2	rs190140598	chr1:237,205,702-237,997,288	AX-90068425
RYR2	---	chr1:237,205,702-237,997,288	AX-94354495
RYR2	rs73106477	chr1:237,205,702-237,997,288	AX-16949454
RYR2	rs76313299	chr1:237,205,702-237,997,288	AX-16949458
RYR2	rs121918602	chr1:237,205,702-237,997,288	AX-88754919
RYR2	rs3765097	chr1:237,205,702-237,997,288	AX-12560432
RYR2	rs376612295	chr1:237,205,702-237,997,288	AX-86658630
RYR2	rs78281932	chr1:237,205,702-237,997,288	AX-16949477
RYR2	rs1478912	chr1:237,205,702-237,997,288	AX-39411251
RYR2	rs2618679	chr1:237,205,702-237,997,288	AX-16949500
RYR2	rs73108485	chr1:237,205,702-237,997,288	AX-16949505
RYR2	rs35662567	chr1:237,205,702-237,997,288	AX-16949511
RYR2	---	chr1:237,205,702-237,997,288	AX-94354496
RYR2	rs10925430	chr1:237,205,702-237,997,288	AX-16949534
RYR2	rs16835294	chr1:237,205,702-237,997,288	AX-11282247

RYR2	rs12734456	chr1:237,205,702-237,997,288	AX-11225237
RYR2	rs6429021	chr1:237,205,702-237,997,288	AX-11563408
RYR2	rs75504890	chr1:237,205,702-237,997,288	AX-16949561
RYR2	rs8179365	chr1:237,205,702-237,997,288	AX-11667538
RYR2	rs2618683	chr1:237,205,702-237,997,288	AX-11401672
RYR2	rs80127105	chr1:237,205,702-237,997,288	AX-16949567
RYR2	rs111305036	chr1:237,205,702-237,997,288	AX-16949568
RYR2	rs12142897	chr1:237,205,702-237,997,288	AX-11192768
RYR2	rs1982645	chr1:237,205,702-237,997,288	AX-30723503
RYR2	rs6673954	chr1:237,205,702-237,997,288	AX-11577576
RYR2	rs2927936	chr1:237,205,702-237,997,288	AX-16949574
RYR2	rs72549414	chr1:237,205,702-237,997,288	AX-30723529
RYR2	---	chr1:237,205,702-237,997,288	AX-94371247
RYR2	rs202040519	chr1:237,205,702-237,997,288	AX-83245266
RYR2	rs2255277	chr1:237,205,702-237,997,288	AX-16949590
RYR2	rs116098815	chr1:237,205,702-237,997,288	AX-16949594
RYR2	---	chr1:237,205,702-237,997,288	AX-94371248
RYR2	rs2805425	chr1:237,205,702-237,997,288	AX-11411414
RYR2	rs61833803	chr1:237,205,702-237,997,288	AX-16949628
RYR2	rs79700489	chr1:237,205,702-237,997,288	AX-16949637
RYR2	rs2618687	chr1:237,205,702-237,997,288	AX-11401673
RYR2	rs75411491	chr1:237,205,702-237,997,288	AX-16949650
RYR2	rs10925439	chr1:237,205,702-237,997,288	AX-39411379
RYR2	rs2805432	chr1:237,205,702-237,997,288	AX-30723663
RYR2	rs116523878	chr1:237,205,702-237,997,288	AX-16949667
RYR2	rs10925446	chr1:237,205,702-237,997,288	AX-16949668
RYR2	rs2805446	chr1:237,205,702-237,997,288	AX-11411417
RYR2	rs12145423	chr1:237,205,702-237,997,288	AX-16949687
RYR2	rs2618674	chr1:237,205,702-237,997,288	AX-11401671
RYR2	rs115264366	chr1:237,205,702-237,997,288	AX-16949688
RYR2	rs2779378	chr1:237,205,702-237,997,288	AX-30723797
RYR2	---	chr1:237,205,702-237,997,288	AX-16949700
RYR2	rs2253273	chr1:237,205,702-237,997,288	AX-11379018
RYR2	rs149514924	chr1:237,205,702-237,997,288	AX-83500522
RYR2	---	chr1:237,205,702-237,997,288	AX-94362576
RYR2	rs113050511	chr1:237,205,702-237,997,288	AX-16949721
RYR2	rs115108564	chr1:237,205,702-237,997,288	AX-16949724
RYR2	rs2061840	chr1:237,205,702-237,997,288	AX-11364355
RYR2	rs71642898	chr1:237,205,702-237,997,288	AX-30723865
RYR2	rs2805473	chr1:237,205,702-237,997,288	AX-16949737
RYR2	rs2805389	chr1:237,205,702-237,997,288	AX-11411411
RYR2	rs3766865	chr1:237,205,702-237,997,288	AX-11479113
RYR2	rs201312753	chr1:237,205,702-237,997,288	AX-83338573
RYR2	rs200525962	chr1:237,205,702-237,997,288	AX-83443903
RYR2	rs116125917	chr1:237,205,702-237,997,288	AX-16949753
RYR2	rs12740534	chr1:237,205,702-237,997,288	AX-11225424
RYR2	rs2805399	chr1:237,205,702-237,997,288	AX-30723935
RYR2	rs12131976	chr1:237,205,702-237,997,288	AX-30723965
RYR2	rs10754613	chr1:237,205,702-237,997,288	AX-30723985
RYR2	rs1842085	chr1:237,205,702-237,997,288	AX-12507379
RYR2	rs79060382	chr1:237,205,702-237,997,288	AX-50645106
RYR2	rs2805411	chr1:237,205,702-237,997,288	AX-16949822
RYR2	rs56229512	chr1:237,205,702-237,997,288	AX-30724071
RYR2	rs2805413	chr1:237,205,702-237,997,288	AX-30724087
RYR2	rs115978575	chr1:237,205,702-237,997,288	AX-30724091

RYR2	rs1074189	chr1:237,205,702-237,997,288	AX-16949844
RYR2	rs791543	chr1:237,205,702-237,997,288	AX-11653508
RYR2	rs2779348	chr1:237,205,702-237,997,288	AX-11410068
RYR2	rs13374055	chr1:237,205,702-237,997,288	AX-16949861
RYR2	rs13376031	chr1:237,205,702-237,997,288	AX-11251466
RYR2	rs1717783	chr1:237,205,702-237,997,288	AX-11312552
RYR2	rs10925473	chr1:237,205,702-237,997,288	AX-11132020
RYR2	rs116450377	chr1:237,205,702-237,997,288	AX-16949868
RYR2	rs79811945	chr1:237,205,702-237,997,288	AX-16949870
RYR2	rs12121663	chr1:237,205,702-237,997,288	AX-11191299
RYR2	rs116123906	chr1:237,205,702-237,997,288	AX-37446279
RYR2	---	chr1:237,205,702-237,997,288	AX-90036812
RYR2	---	chr1:237,205,702-237,997,288	AX-94371249
RYR2	rs3820216	chr1:237,205,702-237,997,288	AX-39411623
RYR2	---	chr1:237,205,702-237,997,288	AX-94371250
RYR2	rs41315858	chr1:237,205,702-237,997,288	AX-11494611
RYR2	rs3766871	chr1:237,205,702-237,997,288	AX-11479114
RYR2	rs596502	chr1:237,205,702-237,997,288	AX-11552990
RYR2	rs111695681	chr1:237,205,702-237,997,288	AX-37446283
RYR2	rs72751219	chr1:237,205,702-237,997,288	AX-16949891
RYR2	rs115362592	chr1:237,205,702-237,997,288	AX-30724179
RYR2	rs571026	chr1:237,205,702-237,997,288	AX-11545731
RYR2	rs7531957	chr1:237,205,702-237,997,288	AX-11627961
RYR2	rs615869	chr1:237,205,702-237,997,288	AX-12597326
RYR2	rs186906598	chr1:237,205,702-237,997,288	AX-82945126
RYR2	---	chr1:237,205,702-237,997,288	AX-94354501
RYR2	---	chr1:237,205,702-237,997,288	AX-94379327
RYR2	rs1759119	chr1:237,205,702-237,997,288	AX-16949909
RYR2	rs116234420	chr1:237,205,702-237,997,288	AX-16949912
RYR2	rs12563366	chr1:237,205,702-237,997,288	AX-11215013
RYR2	---	chr1:237,205,702-237,997,288	AX-94362578
RYR2	---	chr1:237,205,702-237,997,288	AX-94371252
RYR2	---	chr1:237,205,702-237,997,288	AX-94354503
RYR2	rs7526807	chr1:237,205,702-237,997,288	AX-11627635
RYR2	rs121918597	chr1:237,205,702-237,997,288	AX-94353341
RYR2	---	chr1:237,205,702-237,997,288	AX-94379329
RYR2	rs80315247	chr1:237,205,702-237,997,288	AX-16949924
RYR2	---	chr1:237,205,702-237,997,288	AX-90028550
RYR2	rs707189	chr1:237,205,702-237,997,288	AX-39411679
RYR2	---	chr1:237,205,702-237,997,288	AX-94379330
RYR2	---	chr1:237,205,702-237,997,288	AX-94354505
RYR2	---	chr1:237,205,702-237,997,288	AX-94379331
RYR2	---	chr1:237,205,702-237,997,288	AX-94379332
RYR2	---	chr1:237,205,702-237,997,288	AX-94379333
RYR2	rs121918603	chr1:237,205,702-237,997,288	AX-94370097
RYR2	---	chr1:237,205,702-237,997,288	AX-94362584
RYR2	---	chr1:237,205,702-237,997,288	AX-86727416
RYR2	---	chr1:237,205,702-237,997,288	AX-90058302
RYR2	---	chr1:237,205,702-237,997,288	AX-94354506
RYR2	---	chr1:237,205,702-237,997,288	AX-94371257
RYR2	---	chr1:237,205,702-237,997,288	AX-94380104
RYR2	---	chr1:237,205,702-237,997,288	AX-94371258
RYR2	---	chr1:237,205,702-237,997,288	AX-94354509
RYR2	rs1342835	chr1:237,205,702-237,997,288	AX-12458446
RYR2	rs11583033	chr1:237,205,702-237,997,288	AX-39411709

RYR2	rs80107454	chr1:237,205,702-237,997,288	AX-16949944
RYR2	rs72549416	chr1:237,205,702-237,997,288	AX-30724263
RYR2	rs121918598	chr1:237,205,702-237,997,288	AX-94353342
RYR2	---	chr1:237,205,702-237,997,288	AX-94379335
RYR2	rs374191985	chr1:237,205,702-237,997,288	AX-86672405
RYR2	---	chr1:237,205,702-237,997,288	AX-86561291
RYR2	rs80252757	chr1:237,205,702-237,997,288	AX-30724267
RYR2	rs684923	chr1:237,205,702-237,997,288	AX-16949957
RYR2	rs1402803	chr1:237,205,702-237,997,288	AX-11260267
RYR2	rs559344	chr1:237,205,702-237,997,288	AX-11545028
RYR2	rs677730	chr1:237,205,702-237,997,288	AX-11584642
RYR2	rs10925490	chr1:237,205,702-237,997,288	AX-11132023
RYR2	rs56090696	chr1:237,205,702-237,997,288	AX-30724313
RYR2	rs2012813	chr1:237,205,702-237,997,288	AX-16950000
RYR2	---	chr1:237,205,702-237,997,288	AX-86550584
RYR2	rs34967813	chr1:237,205,702-237,997,288	AX-11456067
RYR2	rs12121446	chr1:237,205,702-237,997,288	AX-30724345
RYR2	rs950625	chr1:237,205,702-237,997,288	AX-11690594
RYR2	rs111448231	chr1:237,205,702-237,997,288	AX-16950025
RYR2	rs12032174	chr1:237,205,702-237,997,288	AX-39411769
RYR2	rs2461315	chr1:237,205,702-237,997,288	AX-16950065
RYR2	rs12754190	chr1:237,205,702-237,997,288	AX-30724425
RYR2	rs2797436	chr1:237,205,702-237,997,288	AX-39411779
RYR2	rs12125625	chr1:237,205,702-237,997,288	AX-11191581
RYR2	rs2797441	chr1:237,205,702-237,997,288	AX-39411805
RYR2	rs2090876	chr1:237,205,702-237,997,288	AX-12517012
RYR2	rs2819774	chr1:237,205,702-237,997,288	AX-11412362
RYR2	---	chr1:237,205,702-237,997,288	AX-94356249
RYR2	rs2685301	chr1:237,205,702-237,997,288	AX-16950139
RYR2	rs72751297	chr1:237,205,702-237,997,288	AX-30724517
RYR2	rs3905206	chr1:237,205,702-237,997,288	AX-16950166
RYR2	rs2819770	chr1:237,205,702-237,997,288	AX-11412361
RYR2	---	chr1:237,205,702-237,997,288	AX-94373000
RYR2	---	chr1:237,205,702-237,997,288	AX-94354510
RYR2	---	chr1:237,205,702-237,997,288	AX-94356251
RYR2	rs3817436	chr1:237,205,702-237,997,288	AX-16950200
RYR2	rs2253083	chr1:237,205,702-237,997,288	AX-16950215
RYR2	rs74412575	chr1:237,205,702-237,997,288	AX-37446347
RYR2	rs2250049	chr1:237,205,702-237,997,288	AX-30724607
RYR2	rs2249287	chr1:237,205,702-237,997,288	AX-16950228
RYR2	rs57827447	chr1:237,205,702-237,997,288	AX-16950234
RYR2	---	chr1:237,205,702-237,997,288	AX-94356252
RYR2	---	chr1:237,205,702-237,997,288	AX-94356253
RYR2	---	chr1:237,205,702-237,997,288	AX-94354511
RYR2	---	chr1:237,205,702-237,997,288	AX-94371263
RYR2	---	chr1:237,205,702-237,997,288	AX-94371264
RYR2	---	chr1:237,205,702-237,997,288	AX-94371265
RYR2	rs790889	chr1:237,205,702-237,997,288	AX-11653134
RYR2	---	chr1:237,205,702-237,997,288	AX-86622029
RYR2	---	chr1:237,205,702-237,997,288	AX-94371266
RYR2	---	chr1:237,205,702-237,997,288	AX-94371267
RYR2	---	chr1:237,205,702-237,997,288	AX-94354518
RYR2	---	chr1:237,205,702-237,997,288	AX-94354519
RYR2	---	chr1:237,205,702-237,997,288	AX-94356254
RYR2	---	chr1:237,205,702-237,997,288	AX-94371270

RYR2	---	chr1:237,205,702-237,997,288	AX-94371271
RYR2	---	chr1:237,205,702-237,997,288	AX-94356255
RYR2	---	chr1:237,205,702-237,997,288	AX-94354522
RYR2	---	chr1:237,205,702-237,997,288	AX-94371234
RYR2	---	chr1:237,205,702-237,997,288	AX-94371273
RYR2	---	chr1:237,205,702-237,997,288	AX-94354524
RYR2	---	chr1:237,205,702-237,997,288	AX-94373006
RYR2	---	chr1:237,205,702-237,997,288	AX-94356257
RYR2	---	chr1:237,205,702-237,997,288	AX-94373008
RYR2	---	chr1:237,205,702-237,997,288	AX-94354526
RYR2	---	chr1:237,205,702-237,997,288	AX-94373009
RYR2	---	chr1:237,205,702-237,997,288	AX-94371277
RYR2	---	chr1:237,205,702-237,997,288	AX-94371278
RYR2	---	chr1:237,205,702-237,997,288	AX-94371279
RYR2	---	chr1:237,205,702-237,997,288	AX-94371280
RYR2	---	chr1:237,205,702-237,997,288	AX-94356261
RYR2	---	chr1:237,205,702-237,997,288	AX-94356262
RYR2	---	chr1:237,205,702-237,997,288	AX-94356263
RYR2	rs121918605	chr1:237,205,702-237,997,288	AX-94370099
RYR2	---	chr1:237,205,702-237,997,288	AX-94356264
RYR2	rs200092869	chr1:237,205,702-237,997,288	AX-83111656
RYR2	rs2256242	chr1:237,205,702-237,997,288	AX-11379306
RYR2	rs121918600	chr1:237,205,702-237,997,288	AX-94370094
RYR2	---	chr1:237,205,702-237,997,288	AX-94356265
RYR2	---	chr1:237,205,702-237,997,288	AX-94356266
RYR2	rs397516510	chr1:237,205,702-237,997,288	AX-91999003
RYR2	rs114289907	chr1:237,205,702-237,997,288	AX-16950274
RYR2	rs189345192	chr1:237,205,702-237,997,288	AX-86636490
RYR2	---	chr1:237,205,702-237,997,288	AX-94356267
RYR2	rs117645754	chr1:237,205,702-237,997,288	AX-16950278
RYR2	rs147990209	chr1:237,205,702-237,997,288	AX-94350899
RYR2	---	chr1:237,205,702-237,997,288	AX-94373018
RYR2	---	chr1:237,205,702-237,997,288	AX-94354532
RYR2	rs790899	chr1:237,205,702-237,997,288	AX-12644252
RYR2	rs11806850	chr1:237,205,702-237,997,288	AX-11176733
RYR2	rs72753216	chr1:237,205,702-237,997,288	AX-30724689
RYR2	---	chr1:237,205,702-237,997,288	AX-94371283
RYR2	rs3999765	chr1:237,205,702-237,997,288	AX-12565422
RYR2	rs12043362	chr1:237,205,702-237,997,288	AX-11187881
RYR2	---	chr1:237,205,702-237,997,288	AX-94353348
RYR2	---	chr1:237,205,702-237,997,288	AX-94380689
RYR2	---	chr1:237,205,702-237,997,288	AX-92619362
RYR2	---	chr1:237,205,702-237,997,288	AX-94379337
RYR2	---	chr1:237,205,702-237,997,288	AX-94379338
RYR2	---	chr1:237,205,702-237,997,288	AX-94371286
RYR2	rs2794820	chr1:237,205,702-237,997,288	AX-16950301
RYR2	rs72753233	chr1:237,205,702-237,997,288	AX-30724757
RYR2	---	chr1:237,205,702-237,997,288	AX-90071994
RYR2	---	chr1:237,205,702-237,997,288	AX-94362589
RYR2	---	chr1:237,205,702-237,997,288	AX-94354537
RYR2	rs41267519	chr1:237,205,702-237,997,288	AX-11491191
RYR2	---	chr1:237,205,702-237,997,288	AX-94379340
RYR2	---	chr1:237,205,702-237,997,288	AX-90026428
RYR2	---	chr1:237,205,702-237,997,288	AX-94371288
RYR2	rs75206601	chr1:237,205,702-237,997,288	AX-16950321

RYR2	rs790886	chr1:237,205,702-237,997,288	AX-30724785
RYR2	rs7550039	chr1:237,205,702-237,997,288	AX-11629119
RYR2	rs80278432	chr1:237,205,702-237,997,288	AX-30724793
RYR2	---	chr1:237,205,702-237,997,288	AX-94379341
RYR2	---	chr1:237,205,702-237,997,288	AX-94354539
RYR2	---	chr1:237,205,702-237,997,288	AX-94362592
RYR2	---	chr1:237,205,702-237,997,288	AX-94379343
RYR2	---	chr1:237,205,702-237,997,288	AX-94371290
RYR2	rs121918606	chr1:237,205,702-237,997,288	AX-94370100
RYR2	rs12022376	chr1:237,205,702-237,997,288	AX-39412007
RYR2	rs2794841	chr1:237,205,702-237,997,288	AX-39412015
RYR2	rs2819742	chr1:237,205,702-237,997,288	AX-16950352
RYR2	rs790894	chr1:237,205,702-237,997,288	AX-16950354
RYR2	---	chr1:237,205,702-237,997,288	AX-94362595
RYR2	rs1891247	chr1:237,205,702-237,997,288	AX-16950358
RYR2	---	chr1:237,205,702-237,997,288	AX-94362596
RYR2	---	chr1:237,205,702-237,997,288	AX-94354541
RYR2	---	chr1:237,205,702-237,997,288	AX-94362597
RYR2	---	chr1:237,205,702-237,997,288	AX-94379348
RYR2	---	chr1:237,205,702-237,997,288	AX-92445953
RYR2	---	chr1:237,205,702-237,997,288	AX-94379349
RYR2	rs12594	chr1:237,205,702-237,997,288	AX-11216922
RYR2	rs11581226	chr1:237,205,702-237,997,288	AX-11160133
SCN4B	rs140348243	chr11:118,004,092-118,023,630	AX-86672199
SCN4B	rs116888881	chr11:118,004,092-118,023,630	AX-16496104
SCN4B	rs45539032	chr11:118,004,092-118,023,630	AX-29878797
SCN4B	rs955917	chr11:118,004,092-118,023,630	AX-38969193
SCN4B	rs1793137	chr11:118,004,092-118,023,630	AX-38969195
SCN4B	rs628299	chr11:118,004,092-118,023,630	AX-38969207
SCN4B	rs658624	chr11:118,004,092-118,023,630	AX-38969209
SCN4B	rs34913450	chr11:118,004,092-118,023,630	AX-29878821
SCN4B	rs586053	chr11:118,004,092-118,023,630	AX-38969213
SCN4B	rs12788624	chr11:118,004,092-118,023,630	AX-29878831
SCN4B	rs377558816	chr11:118,004,092-118,023,630	AX-86603324
SCN5A	rs41315485	chr3:38,589,553-38,691,164	AX-34309377
SCN5A	rs200849470	chr3:38,589,553-38,691,164	AX-151131242
SCN5A	rs4073797	chr3:38,589,553-38,691,164	AX-84741238
SCN5A	rs41310757	chr3:38,589,553-38,691,164	AX-11494328
SCN5A	rs7429945	chr3:38,589,553-38,691,164	AX-11624566
SCN5A	rs199473640	chr3:38,589,553-38,691,164	AX-90029406
SCN5A	---	chr3:38,589,553-38,691,164	AX-90078367
SCN5A	rs45489199	chr3:38,589,553-38,691,164	AX-83249005
SCN5A	---	chr3:38,589,553-38,691,164	AX-83312442
SCN5A	rs199473336	chr3:38,589,553-38,691,164	AX-90039721
SCN5A	rs199473335	chr3:38,589,553-38,691,164	AX-86588823
SCN5A	---	chr3:38,589,553-38,691,164	AX-90030522
SCN5A	rs199473331	chr3:38,589,553-38,691,164	AX-86698000
SCN5A	---	chr3:38,589,553-38,691,164	AX-86690052
SCN5A	rs13324293	chr3:38,589,553-38,691,164	AX-41228981
SCN5A	rs199473329	chr3:38,589,553-38,691,164	AX-90063462
SCN5A	---	chr3:38,589,553-38,691,164	AX-90062404
SCN5A	rs137854603	chr3:38,589,553-38,691,164	AX-86666431
SCN5A	---	chr3:38,589,553-38,691,164	AX-86723704
SCN5A	---	chr3:38,589,553-38,691,164	AX-90066196
SCN5A	rs150264233	chr3:38,589,553-38,691,164	AX-83535814

SCN5A	---	chr3:38,589,553-38,691,164	AX-90035645
SCN5A	rs45465995	chr3:38,589,553-38,691,164	AX-83392212
SCN5A	---	chr3:38,589,553-38,691,164	AX-90076754
SCN5A	---	chr3:38,589,553-38,691,164	AX-90056036
SCN5A	---	chr3:38,589,553-38,691,164	AX-90058888
SCN5A	---	chr3:38,589,553-38,691,164	AX-90056266
SCN5A	---	chr3:38,589,553-38,691,164	AX-90079363
SCN5A	---	chr3:38,589,553-38,691,164	AX-90029371
SCN5A	---	chr3:38,589,553-38,691,164	AX-90059186
SCN5A	rs45563942	chr3:38,589,553-38,691,164	AX-83420305
SCN5A	rs199473320	chr3:38,589,553-38,691,164	AX-86603768
SCN5A	rs137854610	chr3:38,589,553-38,691,164	AX-86639919
SCN5A	---	chr3:38,589,553-38,691,164	AX-90028400
SCN5A	---	chr3:38,589,553-38,691,164	AX-90075306
SCN5A	rs1805126	chr3:38,589,553-38,691,164	AX-14300688
SCN5A	rs137854619	chr3:38,589,553-38,691,164	AX-86650285
SCN5A	---	chr3:38,589,553-38,691,164	AX-90032656
SCN5A	rs397514449	chr3:38,589,553-38,691,164	AX-90077942
SCN5A	rs137854614	chr3:38,589,553-38,691,164	AX-90070904
SCN5A	rs137854615	chr3:38,589,553-38,691,164	AX-90070905
SCN5A	---	chr3:38,589,553-38,691,164	AX-90035714
SCN5A	---	chr3:38,589,553-38,691,164	AX-90075198
SCN5A	---	chr3:38,589,553-38,691,164	AX-90032612
SCN5A	---	chr3:38,589,553-38,691,164	AX-90080910
SCN5A	---	chr3:38,589,553-38,691,164	AX-90078767
SCN5A	rs137854601	chr3:38,589,553-38,691,164	AX-90042014
SCN5A	rs199473634	chr3:38,589,553-38,691,164	AX-86563483
SCN5A	---	chr3:38,589,553-38,691,164	AX-86653836
SCN5A	---	chr3:38,589,553-38,691,164	AX-90034708
SCN5A	---	chr3:38,589,553-38,691,164	AX-90029857
SCN5A	---	chr3:38,589,553-38,691,164	AX-90029670
SCN5A	---	chr3:38,589,553-38,691,164	AX-90027724
SCN5A	---	chr3:38,589,553-38,691,164	AX-90032888
SCN5A	---	chr3:38,589,553-38,691,164	AX-90029471
SCN5A	---	chr3:38,589,553-38,691,164	AX-90045660
SCN5A	---	chr3:38,589,553-38,691,164	AX-90037318
SCN5A	---	chr3:38,589,553-38,691,164	AX-90065998
SCN5A	---	chr3:38,589,553-38,691,164	AX-90036092
SCN5A	---	chr3:38,589,553-38,691,164	AX-90030585
SCN5A	---	chr3:38,589,553-38,691,164	AX-90062984
SCN5A	rs199473303	chr3:38,589,553-38,691,164	AX-90068607
SCN5A	---	chr3:38,589,553-38,691,164	AX-90033734
SCN5A	---	chr3:38,589,553-38,691,164	AX-90034740
SCN5A	---	chr3:38,589,553-38,691,164	AX-90029764
SCN5A	---	chr3:38,589,553-38,691,164	AX-90027246
SCN5A	---	chr3:38,589,553-38,691,164	AX-90056849
SCN5A	---	chr3:38,589,553-38,691,164	AX-90035512
SCN5A	rs137854604	chr3:38,589,553-38,691,164	AX-90070901
SCN5A	---	chr3:38,589,553-38,691,164	AX-90028267
SCN5A	---	chr3:38,589,553-38,691,164	AX-90064189
SCN5A	---	chr3:38,589,553-38,691,164	AX-90076864
SCN5A	---	chr3:38,589,553-38,691,164	AX-90031049
SCN5A	---	chr3:38,589,553-38,691,164	AX-90036908
SCN5A	---	chr3:38,589,553-38,691,164	AX-90034359
SCN5A	---	chr3:38,589,553-38,691,164	AX-90026302

SCN5A	---	chr3:38,589,553-38,691,164	AX-90046419
SCN5A	---	chr3:38,589,553-38,691,164	AX-90060936
SCN5A	---	chr3:38,589,553-38,691,164	AX-90056860
SCN5A	---	chr3:38,589,553-38,691,164	AX-90027912
SCN5A	---	chr3:38,589,553-38,691,164	AX-90033979
SCN5A	---	chr3:38,589,553-38,691,164	AX-90061022
SCN5A	---	chr3:38,589,553-38,691,164	AX-90030946
SCN5A	---	chr3:38,589,553-38,691,164	AX-90062048
SCN5A	---	chr3:38,589,553-38,691,164	AX-90062775
SCN5A	---	chr3:38,589,553-38,691,164	AX-90030607
SCN5A	rs28937316	chr3:38,589,553-38,691,164	AX-90066801
SCN5A	---	chr3:38,589,553-38,691,164	AX-90031850
SCN5A	---	chr3:38,589,553-38,691,164	AX-90037230
SCN5A	---	chr3:38,589,553-38,691,164	AX-86718640
SCN5A	---	chr3:38,589,553-38,691,164	AX-90027405
SCN5A	---	chr3:38,589,553-38,691,164	AX-90032451
SCN5A	---	chr3:38,589,553-38,691,164	AX-90026304
SCN5A	---	chr3:38,589,553-38,691,164	AX-90027108
SCN5A	---	chr3:38,589,553-38,691,164	AX-90064968
SCN5A	---	chr3:38,589,553-38,691,164	AX-90026971
SCN5A	---	chr3:38,589,553-38,691,164	AX-90070903
SCN5A	---	chr3:38,589,553-38,691,164	AX-90055857
SCN5A	---	chr3:38,589,553-38,691,164	AX-86719703
SCN5A	rs41315495	chr3:38,589,553-38,691,164	AX-41228983
SCN5A	---	chr3:38,589,553-38,691,164	AX-90026305
SCN5A	---	chr3:38,589,553-38,691,164	AX-90057228
SCN5A	---	chr3:38,589,553-38,691,164	AX-90035741
SCN5A	rs12053903	chr3:38,589,553-38,691,164	AX-41228985
SCN5A	rs7619648	chr3:38,589,553-38,691,164	AX-34309401
SCN5A	rs7373779	chr3:38,589,553-38,691,164	AX-34309403
SCN5A	rs56990533	chr3:38,589,553-38,691,164	AX-96927492
SCN5A	rs7638624	chr3:38,589,553-38,691,164	AX-96957425
SCN5A	rs7638909	chr3:38,589,553-38,691,164	AX-96077108
SCN5A	---	chr3:38,589,553-38,691,164	AX-90072409
SCN5A	---	chr3:38,589,553-38,691,164	AX-86702219
SCN5A	---	chr3:38,589,553-38,691,164	AX-86673218
SCN5A	rs199473278	chr3:38,589,553-38,691,164	AX-86577111
SCN5A	---	chr3:38,589,553-38,691,164	AX-90026973
SCN5A	---	chr3:38,589,553-38,691,164	AX-90026974
SCN5A	---	chr3:38,589,553-38,691,164	AX-90061659
SCN5A	---	chr3:38,589,553-38,691,164	AX-90033970
SCN5A	---	chr3:38,589,553-38,691,164	AX-90026306
SCN5A	---	chr3:38,589,553-38,691,164	AX-90036817
SCN5A	rs45514691	chr3:38,589,553-38,691,164	AX-11510503
SCN5A	---	chr3:38,589,553-38,691,164	AX-90059225
SCN5A	---	chr3:38,589,553-38,691,164	AX-90050954
SCN5A	---	chr3:38,589,553-38,691,164	AX-90037048
SCN5A	---	chr3:38,589,553-38,691,164	AX-90060536
SCN5A	---	chr3:38,589,553-38,691,164	AX-90033650
SCN5A	---	chr3:38,589,553-38,691,164	AX-90049684
SCN5A	---	chr3:38,589,553-38,691,164	AX-90031068
SCN5A	---	chr3:38,589,553-38,691,164	AX-90056455
SCN5A	---	chr3:38,589,553-38,691,164	AX-90029193
SCN5A	rs199473618	chr3:38,589,553-38,691,164	AX-83229868
SCN5A	rs199473269	chr3:38,589,553-38,691,164	AX-86688685

SCN5A	---	chr3:38,589,553-38,691,164	AX-90032553
SCN5A	---	chr3:38,589,553-38,691,164	AX-90028792
SCN5A	rs41315501	chr3:38,589,553-38,691,164	AX-156274118
SCN5A	rs41315501	chr3:38,589,553-38,691,164	AX-156291765
SCN5A	---	chr3:38,589,553-38,691,164	AX-90059487
SCN5A	---	chr3:38,589,553-38,691,164	AX-90031945
SCN5A	---	chr3:38,589,553-38,691,164	AX-90063204
SCN5A	---	chr3:38,589,553-38,691,164	AX-90071966
SCN5A	---	chr3:38,589,553-38,691,164	AX-90030106
SCN5A	---	chr3:38,589,553-38,691,164	AX-90065357
SCN5A	---	chr3:38,589,553-38,691,164	AX-90063295
SCN5A	---	chr3:38,589,553-38,691,164	AX-90051475
SCN5A	rs199473260	chr3:38,589,553-38,691,164	AX-90042033
SCN5A	---	chr3:38,589,553-38,691,164	AX-90037432
SCN5A	---	chr3:38,589,553-38,691,164	AX-90030936
SCN5A	---	chr3:38,589,553-38,691,164	AX-90042947
SCN5A	---	chr3:38,589,553-38,691,164	AX-90065054
SCN5A	---	chr3:38,589,553-38,691,164	AX-90029393
SCN5A	rs7645178	chr3:38,589,553-38,691,164	AX-41229007
SCN5A	rs41315507	chr3:38,589,553-38,691,164	AX-156274119
SCN5A	rs41315507	chr3:38,589,553-38,691,164	AX-156291766
SCN5A	---	chr3:38,589,553-38,691,164	AX-90064269
SCN5A	---	chr3:38,589,553-38,691,164	AX-90079357
SCN5A	---	chr3:38,589,553-38,691,164	AX-90026308
SCN5A	---	chr3:38,589,553-38,691,164	AX-90057111
SCN5A	---	chr3:38,589,553-38,691,164	AX-90064876
SCN5A	---	chr3:38,589,553-38,691,164	AX-90076484
SCN5A	---	chr3:38,589,553-38,691,164	AX-90065634
SCN5A	---	chr3:38,589,553-38,691,164	AX-90080328
SCN5A	---	chr3:38,589,553-38,691,164	AX-90029347
SCN5A	---	chr3:38,589,553-38,691,164	AX-90036466
SCN5A	---	chr3:38,589,553-38,691,164	AX-90027646
SCN5A	---	chr3:38,589,553-38,691,164	AX-90058382
SCN5A	---	chr3:38,589,553-38,691,164	AX-90060260
SCN5A	---	chr3:38,589,553-38,691,164	AX-90058895
SCN5A	---	chr3:38,589,553-38,691,164	AX-90036693
SCN5A	---	chr3:38,589,553-38,691,164	AX-90027498
SCN5A	---	chr3:38,589,553-38,691,164	AX-90060020
SCN5A	rs45505695	chr3:38,589,553-38,691,164	AX-11510409
SCN5A	rs41312391	chr3:38,589,553-38,691,164	AX-41229011
SCN5A	rs41312393	chr3:38,589,553-38,691,164	AX-156265641
SCN5A	rs41312393	chr3:38,589,553-38,691,164	AX-156265675
SCN5A	---	chr3:38,589,553-38,691,164	AX-90027177
SCN5A	---	chr3:38,589,553-38,691,164	AX-90033139
SCN5A	---	chr3:38,589,553-38,691,164	AX-90057783
SCN5A	---	chr3:38,589,553-38,691,164	AX-90058583
SCN5A	---	chr3:38,589,553-38,691,164	AX-90062706
SCN5A	---	chr3:38,589,553-38,691,164	AX-90064122
SCN5A	rs137854620	chr3:38,589,553-38,691,164	AX-88755774
SCN5A	---	chr3:38,589,553-38,691,164	AX-90056048
SCN5A	rs6804918	chr3:38,589,553-38,691,164	AX-34309425
SCN5A	rs6793245	chr3:38,589,553-38,691,164	AX-11585785
SCN5A	rs11720166	chr3:38,589,553-38,691,164	AX-34309429
SCN5A	rs369328824	chr3:38,589,553-38,691,164	AX-156286149
SCN5A	rs112108692	chr3:38,589,553-38,691,164	AX-156278784

SCN5A	rs112108692	chr3:38,589,553-38,691,164	AX-94348720
SCN5A	rs9845438	chr3:38,589,553-38,691,164	AX-34309431
SCN5A	rs13059004	chr3:38,589,553-38,691,164	AX-41229015
SCN5A	rs6799868	chr3:38,589,553-38,691,164	AX-156291767
SCN5A	---	chr3:38,589,553-38,691,164	AX-90055195
SCN5A	---	chr3:38,589,553-38,691,164	AX-90060972
SCN5A	rs137854612	chr3:38,589,553-38,691,164	AX-90070761
SCN5A	---	chr3:38,589,553-38,691,164	AX-90031038
SCN5A	---	chr3:38,589,553-38,691,164	AX-90059143
SCN5A	---	chr3:38,589,553-38,691,164	AX-90061843
SCN5A	rs397514446	chr3:38,589,553-38,691,164	AX-90049119
SCN5A	---	chr3:38,589,553-38,691,164	AX-90055197
SCN5A	---	chr3:38,589,553-38,691,164	AX-90055198
SCN5A	---	chr3:38,589,553-38,691,164	AX-90061350
SCN5A	---	chr3:38,589,553-38,691,164	AX-90037072
SCN5A	---	chr3:38,589,553-38,691,164	AX-90036899
SCN5A	---	chr3:38,589,553-38,691,164	AX-90065981
SCN5A	---	chr3:38,589,553-38,691,164	AX-90061649
SCN5A	---	chr3:38,589,553-38,691,164	AX-90063255
SCN5A	---	chr3:38,589,553-38,691,164	AX-90027332
SCN5A	---	chr3:38,589,553-38,691,164	AX-90045806
SCN5A	---	chr3:38,589,553-38,691,164	AX-90062377
SCN5A	---	chr3:38,589,553-38,691,164	AX-90060446
SCN5A	---	chr3:38,589,553-38,691,164	AX-90027278
SCN5A	---	chr3:38,589,553-38,691,164	AX-90063136
SCN5A	---	chr3:38,589,553-38,691,164	AX-90057829
SCN5A	---	chr3:38,589,553-38,691,164	AX-90056452
SCN5A	---	chr3:38,589,553-38,691,164	AX-90061234
SCN5A	---	chr3:38,589,553-38,691,164	AX-90057771
SCN5A	---	chr3:38,589,553-38,691,164	AX-86547396
SCN5A	---	chr3:38,589,553-38,691,164	AX-90060314
SCN5A	---	chr3:38,589,553-38,691,164	AX-90059268
SCN5A	---	chr3:38,589,553-38,691,164	AX-90060851
SCN5A	---	chr3:38,589,553-38,691,164	AX-90056905
SCN5A	---	chr3:38,589,553-38,691,164	AX-90035671
SCN5A	---	chr3:38,589,553-38,691,164	AX-90056476
SCN5A	---	chr3:38,589,553-38,691,164	AX-90061612
SCN5A	---	chr3:38,589,553-38,691,164	AX-90066300
SCN5A	rs28937317	chr3:38,589,553-38,691,164	AX-90055860
SCN5A	---	chr3:38,589,553-38,691,164	AX-90035247
SCN5A	rs73825594	chr3:38,589,553-38,691,164	AX-156274121
SCN5A	rs3935472	chr3:38,589,553-38,691,164	AX-11488256
SCN5A	rs62241188	chr3:38,589,553-38,691,164	AX-14300695
SCN5A	rs57574696	chr3:38,589,553-38,691,164	AX-96946330
SCN5A	rs57574696	chr3:38,589,553-38,691,164	AX-96956888
SCN5A	---	chr3:38,589,553-38,691,164	AX-96780764
SCN5A	rs9824500	chr3:38,589,553-38,691,164	AX-156267770
SCN5A	rs9824500	chr3:38,589,553-38,691,164	AX-156287759
SCN5A	---	chr3:38,589,553-38,691,164	AX-90062451
SCN5A	rs397514447	chr3:38,589,553-38,691,164	AX-90057127
SCN5A	rs199473220	chr3:38,589,553-38,691,164	AX-86705487
SCN5A	---	chr3:38,589,553-38,691,164	AX-90074928
SCN5A	---	chr3:38,589,553-38,691,164	AX-90035946
SCN5A	rs41313031	chr3:38,589,553-38,691,164	AX-83390367
SCN5A	rs199473603	chr3:38,589,553-38,691,164	AX-83256635

SCN5A	---	chr3:38,589,553-38,691,164	AX-90073580
SCN5A	rs28937319	chr3:38,589,553-38,691,164	AX-90070762
SCN5A	---	chr3:38,589,553-38,691,164	AX-90080495
SCN5A	---	chr3:38,589,553-38,691,164	AX-90063759
SCN5A	rs41311127	chr3:38,589,553-38,691,164	AX-83561442
SCN5A	---	chr3:38,589,553-38,691,164	AX-90035265
SCN5A	rs111471466	chr3:38,589,553-38,691,164	AX-96904629
SCN5A	rs201499061	chr3:38,589,553-38,691,164	AX-96924931
SCN5A	rs6792856	chr3:38,589,553-38,691,164	AX-96829452
SCN5A	rs7374138	chr3:38,589,553-38,691,164	AX-11623417
SCN5A	rs73070938	chr3:38,589,553-38,691,164	AX-34309451
SCN5A	rs7427447	chr3:38,589,553-38,691,164	AX-41229025
SCN5A	rs11129796	chr3:38,589,553-38,691,164	AX-96981996
SCN5A	rs11129796	chr3:38,589,553-38,691,164	AX-96988477
SCN5A	rs62241189	chr3:38,589,553-38,691,164	AX-96741507
SCN5A	rs62241189	chr3:38,589,553-38,691,164	AX-97392301
SCN5A	rs9832586	chr3:38,589,553-38,691,164	AX-41229029
SCN5A	rs9812912	chr3:38,589,553-38,691,164	AX-11703515
SCN5A	rs7374605	chr3:38,589,553-38,691,164	AX-41229031
SCN5A	rs7372251	chr3:38,589,553-38,691,164	AX-96845939
SCN5A	rs7372251	chr3:38,589,553-38,691,164	AX-96848028
SCN5A	rs73070948	chr3:38,589,553-38,691,164	AX-34309459
SCN5A	---	chr3:38,589,553-38,691,164	AX-90035770
SCN5A	rs199473341	chr3:38,589,553-38,691,164	AX-86557322
SCN5A	---	chr3:38,589,553-38,691,164	AX-90061360
SCN5A	rs137854618	chr3:38,589,553-38,691,164	AX-90070907
SCN5A	---	chr3:38,589,553-38,691,164	AX-90064782
SCN5A	---	chr3:38,589,553-38,691,164	AX-90057419
SCN5A	rs137854616	chr3:38,589,553-38,691,164	AX-86575539
SCN5A	---	chr3:38,589,553-38,691,164	AX-90060566
SCN5A	rs45589741	chr3:38,589,553-38,691,164	AX-90066802
SCN5A	---	chr3:38,589,553-38,691,164	AX-90059006
SCN5A	rs199473599	chr3:38,589,553-38,691,164	AX-86699238
SCN5A	---	chr3:38,589,553-38,691,164	AX-90028320
SCN5A	---	chr3:38,589,553-38,691,164	AX-90064006
SCN5A	---	chr3:38,589,553-38,691,164	AX-90056496
SCN5A	---	chr3:38,589,553-38,691,164	AX-86639161
SCN5A	---	chr3:38,589,553-38,691,164	AX-90035577
SCN5A	---	chr3:38,589,553-38,691,164	AX-90035118
SCN5A	---	chr3:38,589,553-38,691,164	AX-90031650
SCN5A	---	chr3:38,589,553-38,691,164	AX-90047404
SCN5A	rs9859771	chr3:38,589,553-38,691,164	AX-94349489
SCN5A	rs9859771	chr3:38,589,553-38,691,164	AX-96975166
SCN5A	rs9822819	chr3:38,589,553-38,691,164	AX-41229035
SCN5A	rs34991003	chr3:38,589,553-38,691,164	AX-96773584
SCN5A	rs115691534	chr3:38,589,553-38,691,164	AX-96811412
SCN5A	rs115691534	chr3:38,589,553-38,691,164	AX-96817772
SCN5A	rs9861242	chr3:38,589,553-38,691,164	AX-41229037
SCN5A	rs34041219	chr3:38,589,553-38,691,164	AX-96977820
SCN5A	rs9833086	chr3:38,589,553-38,691,164	AX-11704894
SCN5A	rs60437567	chr3:38,589,553-38,691,164	AX-96993707
SCN5A	rs60437567	chr3:38,589,553-38,691,164	AX-97486709
SCN5A	rs56961194	chr3:38,589,553-38,691,164	AX-96874708
SCN5A	rs56961194	chr3:38,589,553-38,691,164	AX-97486691
SCN5A	rs4130467	chr3:38,589,553-38,691,164	AX-41229039

SCN5A	rs4131778	chr3:38,589,553-38,691,164	AX-41229041
SCN5A	rs6422141	chr3:38,589,553-38,691,164	AX-96977339
SCN5A	rs6422141	chr3:38,589,553-38,691,164	AX-96992496
SCN5A	rs7620661	chr3:38,589,553-38,691,164	AX-12634370
SCN5A	rs7620661	chr3:38,589,553-38,691,164	AX-156274122
SCN5A	rs7620883	chr3:38,589,553-38,691,164	AX-96737401
SCN5A	rs7620883	chr3:38,589,553-38,691,164	AX-97333443
SCN5A	rs7623725	chr3:38,589,553-38,691,164	AX-151326337
SCN5A	rs7623725	chr3:38,589,553-38,691,164	AX-156278785
SCN5A	rs6772948	chr3:38,589,553-38,691,164	AX-34309475
SCN5A	rs58454174	chr3:38,589,553-38,691,164	AX-96775692
SCN5A	rs58454174	chr3:38,589,553-38,691,164	AX-96782198
SCN5A	rs56856626	chr3:38,589,553-38,691,164	AX-151326362
SCN5A	rs56856626	chr3:38,589,553-38,691,164	AX-156278786
SCN5A	---	chr3:38,589,553-38,691,164	AX-90042649
SCN5A	---	chr3:38,589,553-38,691,164	AX-90061153
SCN5A	---	chr3:38,589,553-38,691,164	AX-90073093
SCN5A	---	chr3:38,589,553-38,691,164	AX-90055200
SCN5A	---	chr3:38,589,553-38,691,164	AX-90035234
SCN5A	---	chr3:38,589,553-38,691,164	AX-90061026
SCN5A	---	chr3:38,589,553-38,691,164	AX-90031903
SCN5A	rs41261344	chr3:38,589,553-38,691,164	AX-34309479
SCN5A	---	chr3:38,589,553-38,691,164	AX-90026316
SCN5A	---	chr3:38,589,553-38,691,164	AX-90026317
SCN5A	---	chr3:38,589,553-38,691,164	AX-86695124
SCN5A	rs41310765	chr3:38,589,553-38,691,164	AX-86660151
SCN5A	---	chr3:38,589,553-38,691,164	AX-90058574
SCN5A	---	chr3:38,589,553-38,691,164	AX-90037189
SCN5A	rs6599213	chr3:38,589,553-38,691,164	AX-34309481
SCN5A	rs78095439	chr3:38,589,553-38,691,164	AX-148595839
SCN5A	rs78095439	chr3:38,589,553-38,691,164	AX-156281842
SCN5A	---	chr3:38,589,553-38,691,164	AX-90033017
SCN5A	---	chr3:38,589,553-38,691,164	AX-90060766
SCN5A	---	chr3:38,589,553-38,691,164	AX-90027359
SCN5A	---	chr3:38,589,553-38,691,164	AX-34309489
SCN5A	rs112357181	chr3:38,589,553-38,691,164	AX-96795830
SCN5A	rs7373819	chr3:38,589,553-38,691,164	AX-41229047
SCN5A	rs7428882	chr3:38,589,553-38,691,164	AX-96807906
SCN5A	rs7428882	chr3:38,589,553-38,691,164	AX-96818645
SCN5A	rs113845796	chr3:38,589,553-38,691,164	AX-96798647
SCN5A	rs113845796	chr3:38,589,553-38,691,164	AX-96826756
SCN5A	rs4073103	chr3:38,589,553-38,691,164	AX-96987900
SCN5A	rs4073103	chr3:38,589,553-38,691,164	AX-97405863
SCN5A	---	chr3:38,589,553-38,691,164	AX-90055203
SCN5A	---	chr3:38,589,553-38,691,164	AX-90026319
SCN5A	---	chr3:38,589,553-38,691,164	AX-90036575
SCN5A	---	chr3:38,589,553-38,691,164	AX-90036219
SCN5A	rs7626962	chr3:38,589,553-38,691,164	AX-41229053
SCN5A	rs199473192	chr3:38,589,553-38,691,164	AX-82959958
SCN5A	---	chr3:38,589,553-38,691,164	AX-90027519
SCN5A	rs41312411	chr3:38,589,553-38,691,164	AX-156291769
SCN5A	rs41313661	chr3:38,589,553-38,691,164	AX-156274124
SCN5A	rs41313661	chr3:38,589,553-38,691,164	AX-156291770
SCN5A	rs4072710	chr3:38,589,553-38,691,164	AX-34309501
SCN5A	rs7430407	chr3:38,589,553-38,691,164	AX-41229057

SCN5A	---	chr3:38,589,553-38,691,164	AX-90064479
SCN5A	---	chr3:38,589,553-38,691,164	AX-90062545
SCN5A	rs137854617	chr3:38,589,553-38,691,164	AX-90070906
SCN5A	rs199473592	chr3:38,589,553-38,691,164	AX-90039723
SCN5A	---	chr3:38,589,553-38,691,164	AX-90028954
SCN5A	---	chr3:38,589,553-38,691,164	AX-90078324
SCN5A	rs199473183	chr3:38,589,553-38,691,164	AX-86713610
SCN5A	---	chr3:38,589,553-38,691,164	AX-90074800
SCN5A	rs199473182	chr3:38,589,553-38,691,164	AX-86710863
SCN5A	---	chr3:38,589,553-38,691,164	AX-90036660
SCN5A	---	chr3:38,589,553-38,691,164	AX-90043037
SCN5A	rs41311135	chr3:38,589,553-38,691,164	AX-90066803
SCN5A	---	chr3:38,589,553-38,691,164	AX-90032386
SCN5A	rs199473180	chr3:38,589,553-38,691,164	AX-90066445
SCN5A	---	chr3:38,589,553-38,691,164	AX-90029657
SCN5A	---	chr3:38,589,553-38,691,164	AX-90028394
SCN5A	---	chr3:38,589,553-38,691,164	AX-90051211
SCN5A	rs9870213	chr3:38,589,553-38,691,164	AX-96766458
SCN5A	rs9870213	chr3:38,589,553-38,691,164	AX-97420099
SCN5A	rs6790619	chr3:38,589,553-38,691,164	AX-41229059
SCN5A	rs6772989	chr3:38,589,553-38,691,164	AX-156274125
SCN5A	rs6772989	chr3:38,589,553-38,691,164	AX-156291771
SCN5A	rs6773055	chr3:38,589,553-38,691,164	AX-34309509
SCN5A	rs6773076	chr3:38,589,553-38,691,164	AX-41229061
SCN5A	rs3922844	chr3:38,589,553-38,691,164	AX-41229063
SCN5A	rs3922843	chr3:38,589,553-38,691,164	AX-11487736
SCN5A	rs34519218	chr3:38,589,553-38,691,164	AX-156267771
SCN5A	rs34519218	chr3:38,589,553-38,691,164	AX-156287760
SCN5A	rs71323670	chr3:38,589,553-38,691,164	AX-34309513
SCN5A	rs7374004	chr3:38,589,553-38,691,164	AX-41229065
SCN5A	rs7355944	chr3:38,589,553-38,691,164	AX-96908452
SCN5A	rs7355944	chr3:38,589,553-38,691,164	AX-97263204
SCN5A	rs7432766	chr3:38,589,553-38,691,164	AX-41229067
SCN5A	---	chr3:38,589,553-38,691,164	AX-90033714
SCN5A	---	chr3:38,589,553-38,691,164	AX-90057082
SCN5A	---	chr3:38,589,553-38,691,164	AX-90035858
SCN5A	---	chr3:38,589,553-38,691,164	AX-90027117
SCN5A	---	chr3:38,589,553-38,691,164	AX-90058858
SCN5A	---	chr3:38,589,553-38,691,164	AX-90061822
SCN5A	---	chr3:38,589,553-38,691,164	AX-90032414
SCN5A	---	chr3:38,589,553-38,691,164	AX-90029025
SCN5A	---	chr3:38,589,553-38,691,164	AX-90028656
SCN5A	---	chr3:38,589,553-38,691,164	AX-90050541
SCN5A	---	chr3:38,589,553-38,691,164	AX-90080667
SCN5A	---	chr3:38,589,553-38,691,164	AX-90026320
SCN5A	---	chr3:38,589,553-38,691,164	AX-90029743
SCN5A	---	chr3:38,589,553-38,691,164	AX-90078954
SCN5A	---	chr3:38,589,553-38,691,164	AX-90060601
SCN5A	---	chr3:38,589,553-38,691,164	AX-90076667
SCN5A	---	chr3:38,589,553-38,691,164	AX-90034665
SCN5A	---	chr3:38,589,553-38,691,164	AX-90076513
SCN5A	---	chr3:38,589,553-38,691,164	AX-90029647
SCN5A	---	chr3:38,589,553-38,691,164	AX-90073690
SCN5A	---	chr3:38,589,553-38,691,164	AX-90063078
SCN5A	---	chr3:38,589,553-38,691,164	AX-90052147

SCN5A	---	chr3:38,589,553-38,691,164	AX-90064022
SCN5A	---	chr3:38,589,553-38,691,164	AX-90064588
SCN5A	---	chr3:38,589,553-38,691,164	AX-90057358
SCN5A	rs45475899	chr3:38,589,553-38,691,164	AX-12574355
SCN5A	---	chr3:38,589,553-38,691,164	AX-90030980
SCN5A	---	chr3:38,589,553-38,691,164	AX-90055206
SCN5A	---	chr3:38,589,553-38,691,164	AX-90059835
SCN5A	---	chr3:38,589,553-38,691,164	AX-90036810
SCN5A	---	chr3:38,589,553-38,691,164	AX-90034185
SCN5A	rs7645173	chr3:38,589,553-38,691,164	AX-11635881
SCN5A	rs63694161	chr3:38,589,553-38,691,164	AX-96915934
SCN5A	rs63694161	chr3:38,589,553-38,691,164	AX-96918117
SCN5A	rs7645358	chr3:38,589,553-38,691,164	AX-11635894
SCN5A	rs9311190	chr3:38,589,553-38,691,164	AX-41229071
SCN5A	rs11711097	chr3:38,589,553-38,691,164	AX-11169598
SCN5A	rs7432532	chr3:38,589,553-38,691,164	AX-41229075
SCN5A	---	chr3:38,589,553-38,691,164	AX-90066012
SCN5A	---	chr3:38,589,553-38,691,164	AX-90060079
SCN5A	---	chr3:38,589,553-38,691,164	AX-90058874
SCN5A	---	chr3:38,589,553-38,691,164	AX-90027804
SCN5A	---	chr3:38,589,553-38,691,164	AX-90033655
SCN5A	---	chr3:38,589,553-38,691,164	AX-90033093
SCN5A	---	chr3:38,589,553-38,691,164	AX-90030434
SCN5A	---	chr3:38,589,553-38,691,164	AX-90034495
SCN5A	---	chr3:38,589,553-38,691,164	AX-90036797
SCN5A	---	chr3:38,589,553-38,691,164	AX-90050623
SCN5A	---	chr3:38,589,553-38,691,164	AX-90034352
SCN5A	rs7426433	chr3:38,589,553-38,691,164	AX-156274126
SCN5A	rs7426433	chr3:38,589,553-38,691,164	AX-156291772
SCN5A	rs7433889	chr3:38,589,553-38,691,164	AX-11624716
SCN5A	rs6599214	chr3:38,589,553-38,691,164	AX-14300720
SCN5A	rs6599215	chr3:38,589,553-38,691,164	AX-34309531
SCN5A	rs6599216	chr3:38,589,553-38,691,164	AX-34309533
SCN5A	rs6599217	chr3:38,589,553-38,691,164	AX-34309535
SCN5A	rs6599218	chr3:38,589,553-38,691,164	AX-147808710
SCN5A	rs6599218	chr3:38,589,553-38,691,164	AX-147902688
SCN5A	rs62245110	chr3:38,589,553-38,691,164	AX-34309537
SCN5A	rs7613045	chr3:38,589,553-38,691,164	AX-14300722
SCN5A	rs63200660	chr3:38,589,553-38,691,164	AX-96911042
SCN5A	rs7373157	chr3:38,589,553-38,691,164	AX-41229081
SCN5A	rs73070977	chr3:38,589,553-38,691,164	AX-34309541
SCN5A	rs73070981	chr3:38,589,553-38,691,164	AX-14300724
SCN5A	rs61558743	chr3:38,589,553-38,691,164	AX-14300725
SCN5A	rs7374040	chr3:38,589,553-38,691,164	AX-11623416
SCN5A	rs61669000	chr3:38,589,553-38,691,164	AX-96874451
SCN5A	rs61669000	chr3:38,589,553-38,691,164	AX-96889398
SCN5A	rs10154914	chr3:38,589,553-38,691,164	AX-14300726
SCN5A	rs141384382	chr3:38,589,553-38,691,164	AX-151374321
SCN5A	rs141384382	chr3:38,589,553-38,691,164	AX-156284989
SCN5A	rs7374289	chr3:38,589,553-38,691,164	AX-11623423
SCN5A	rs11708996	chr3:38,589,553-38,691,164	AX-11169410
SCN5A	rs7374540	chr3:38,589,553-38,691,164	AX-14300730
SCN5A	rs7373862	chr3:38,589,553-38,691,164	AX-156291774
SCN5A	rs9311192	chr3:38,589,553-38,691,164	AX-148427025
SCN5A	rs9311192	chr3:38,589,553-38,691,164	AX-88736033

SCN5A	rs9311193	chr3:38,589,553-38,691,164	AX-96676204
SCN5A	rs9311193	chr3:38,589,553-38,691,164	AX-96744173
SCN5A	rs9311194	chr3:38,589,553-38,691,164	AX-156281843
SCN5A	rs9311194	chr3:38,589,553-38,691,164	AX-156296669
SCN5A	rs7374826	chr3:38,589,553-38,691,164	AX-34309551
SCN5A	rs11721012	chr3:38,589,553-38,691,164	AX-34309555
SCN5A	rs3924120	chr3:38,589,553-38,691,164	AX-34309557
SCN5A	rs62242766	chr3:38,589,553-38,691,164	AX-34309561
SCN5A	rs62242767	chr3:38,589,553-38,691,164	AX-156274130
SCN5A	rs13062931	chr3:38,589,553-38,691,164	AX-156274131
SCN5A	rs13062931	chr3:38,589,553-38,691,164	AX-156291775
SCN5A	rs6599219	chr3:38,589,553-38,691,164	AX-34309565
SCN5A	rs3935183	chr3:38,589,553-38,691,164	AX-34309567
SCN5A	rs3935184	chr3:38,589,553-38,691,164	AX-14300738
SCN5A	rs10212202	chr3:38,589,553-38,691,164	AX-156291776
SCN5A	rs59559755	chr3:38,589,553-38,691,164	AX-34309569
SCN5A	---	chr3:38,589,553-38,691,164	AX-90028769
SCN5A	---	chr3:38,589,553-38,691,164	AX-90031732
SCN5A	rs199473582	chr3:38,589,553-38,691,164	AX-90039724
SCN5A	---	chr3:38,589,553-38,691,164	AX-86672701
SCN5A	---	chr3:38,589,553-38,691,164	AX-90031593
SCN5A	---	chr3:38,589,553-38,691,164	AX-90027240
SCN5A	---	chr3:38,589,553-38,691,164	AX-90075746
SCN5A	---	chr3:38,589,553-38,691,164	AX-90061733
SCN5A	rs199473149	chr3:38,589,553-38,691,164	AX-86549491
SCN5A	---	chr3:38,589,553-38,691,164	AX-90064100
SCN5A	rs199473147	chr3:38,589,553-38,691,164	AX-90039725
SCN5A	---	chr3:38,589,553-38,691,164	AX-90071892
SCN5A	---	chr3:38,589,553-38,691,164	AX-90026322
SCN5A	rs45553235	chr3:38,589,553-38,691,164	AX-11510915
SCN5A	rs199473146	chr3:38,589,553-38,691,164	AX-83474798
SCN5A	rs199473145	chr3:38,589,553-38,691,164	AX-86693505
SCN5A	---	chr3:38,589,553-38,691,164	AX-90036162
SCN5A	---	chr3:38,589,553-38,691,164	AX-90063721
SCN5A	---	chr3:38,589,553-38,691,164	AX-90063014
SCN5A	---	chr3:38,589,553-38,691,164	AX-90030388
SCN5A	---	chr3:38,589,553-38,691,164	AX-90072898
SCN5A	---	chr3:38,589,553-38,691,164	AX-90056173
SCN5A	---	chr3:38,589,553-38,691,164	AX-90072733
SCN5A	rs199473139	chr3:38,589,553-38,691,164	AX-86619203
SCN5A	---	chr3:38,589,553-38,691,164	AX-90056770
SCN5A	---	chr3:38,589,553-38,691,164	AX-90027374
SCN5A	---	chr3:38,589,553-38,691,164	AX-90077450
SCN5A	---	chr3:38,589,553-38,691,164	AX-90039726
SCN5A	---	chr3:38,589,553-38,691,164	AX-90043103
SCN5A	---	chr3:38,589,553-38,691,164	AX-90034273
SCN5A	---	chr3:38,589,553-38,691,164	AX-90030268
SCN5A	---	chr3:38,589,553-38,691,164	AX-90061866
SCN5A	---	chr3:38,589,553-38,691,164	AX-90032100
SCN5A	---	chr3:38,589,553-38,691,164	AX-90034948
SCN5A	rs9833775	chr3:38,589,553-38,691,164	AX-11704949
SCN5A	rs9871385	chr3:38,589,553-38,691,164	AX-41229099
SCN5A	rs62242768	chr3:38,589,553-38,691,164	AX-34309577
SCN5A	rs73829008	chr3:38,589,553-38,691,164	AX-14300742
SCN5A	rs7427874	chr3:38,589,553-38,691,164	AX-96838830

SCN5A	rs7427874	chr3:38,589,553-38,691,164	AX-97426891
SCN5A	rs6599221	chr3:38,589,553-38,691,164	AX-11574596
SCN5A	rs11714074	chr3:38,589,553-38,691,164	AX-41229103
SCN5A	rs41313685	chr3:38,589,553-38,691,164	AX-156274133
SCN5A	rs41313685	chr3:38,589,553-38,691,164	AX-156291777
SCN5A	rs62242769	chr3:38,589,553-38,691,164	AX-34309581
SCN5A	---	chr3:38,589,553-38,691,164	AX-90064362
SCN5A	---	chr3:38,589,553-38,691,164	AX-90060845
SCN5A	rs199473577	chr3:38,589,553-38,691,164	AX-83573454
SCN5A	rs199473133	chr3:38,589,553-38,691,164	AX-86588562
SCN5A	rs45488304	chr3:38,589,553-38,691,164	AX-41229107
SCN5A	rs397517953	chr3:38,589,553-38,691,164	AX-90051497
SCN5A	---	chr3:38,589,553-38,691,164	AX-90031013
SCN5A	rs199473128	chr3:38,589,553-38,691,164	AX-86558966
SCN5A	---	chr3:38,589,553-38,691,164	AX-90051258
SCN5A	---	chr3:38,589,553-38,691,164	AX-90055208
SCN5A	---	chr3:38,589,553-38,691,164	AX-90058506
SCN5A	rs184442491	chr3:38,589,553-38,691,164	AX-86627620
SCN5A	---	chr3:38,589,553-38,691,164	AX-90037348
SCN5A	---	chr3:38,589,553-38,691,164	AX-90034298
SCN5A	rs1805124	chr3:38,589,553-38,691,164	AX-50383159
SCN5A	---	chr3:38,589,553-38,691,164	AX-90056033
SCN5A	---	chr3:38,589,553-38,691,164	AX-90056361
SCN5A	---	chr3:38,589,553-38,691,164	AX-90064418
SCN5A	---	chr3:38,589,553-38,691,164	AX-90026324
SCN5A	---	chr3:38,589,553-38,691,164	AX-86567742
SCN5A	---	chr3:38,589,553-38,691,164	AX-90031225
SCN5A	rs45627438	chr3:38,589,553-38,691,164	AX-86578366
SCN5A	rs41313691	chr3:38,589,553-38,691,164	AX-41229111
SCN5A	---	chr3:38,589,553-38,691,164	AX-90034663
SCN5A	---	chr3:38,589,553-38,691,164	AX-90074353
SCN5A	rs137854606	chr3:38,589,553-38,691,164	AX-90070902
SCN5A	---	chr3:38,589,553-38,691,164	AX-90080162
SCN5A	---	chr3:38,589,553-38,691,164	AX-90055862
SCN5A	rs34535972	chr3:38,589,553-38,691,164	AX-34309589
SCN5A	rs13084981	chr3:38,589,553-38,691,164	AX-41229113
SCN5A	---	chr3:38,589,553-38,691,164	AX-90064068
SCN5A	---	chr3:38,589,553-38,691,164	AX-90077961
SCN5A	---	chr3:38,589,553-38,691,164	AX-90026325
SCN5A	---	chr3:38,589,553-38,691,164	AX-90063479
SCN5A	---	chr3:38,589,553-38,691,164	AX-90031776
SCN5A	rs199473572	chr3:38,589,553-38,691,164	AX-86655392
SCN5A	rs7428779	chr3:38,589,553-38,691,164	AX-41229115
SCN5A	rs55899887	chr3:38,589,553-38,691,164	AX-34309591
SCN5A	rs11711602	chr3:38,589,553-38,691,164	AX-41229117
SCN5A	---	chr3:38,589,553-38,691,164	AX-90064950
SCN5A	rs199473339	chr3:38,589,553-38,691,164	AX-86683660
SCN5A	---	chr3:38,589,553-38,691,164	AX-90058498
SCN5A	---	chr3:38,589,553-38,691,164	AX-90060189
SCN5A	---	chr3:38,589,553-38,691,164	AX-86673515
SCN5A	rs199473111	chr3:38,589,553-38,691,164	AX-83154288
SCN5A	---	chr3:38,589,553-38,691,164	AX-90055211
SCN5A	---	chr3:38,589,553-38,691,164	AX-90035632
SCN5A	---	chr3:38,589,553-38,691,164	AX-86585594
SCN5A	rs72549410	chr3:38,589,553-38,691,164	AX-90070824

SCN5A	---	chr3:38,589,553-38,691,164	AX-90028294
SCN5A	---	chr3:38,589,553-38,691,164	AX-90031692
SCN5A	---	chr3:38,589,553-38,691,164	AX-90060274
SCN5A	---	chr3:38,589,553-38,691,164	AX-90063502
SCN5A	---	chr3:38,589,553-38,691,164	AX-90057244
SCN5A	---	chr3:38,589,553-38,691,164	AX-90032379
SCN5A	---	chr3:38,589,553-38,691,164	AX-90063636
SCN5A	---	chr3:38,589,553-38,691,164	AX-90027711
SCN5A	---	chr3:38,589,553-38,691,164	AX-90030233
SCN5A	---	chr3:38,589,553-38,691,164	AX-90033229
SCN5A	---	chr3:38,589,553-38,691,164	AX-90034222
SCN5A	---	chr3:38,589,553-38,691,164	AX-90031873
SCN5A	rs41312433	chr3:38,589,553-38,691,164	AX-34309597
SCN5A	rs6770569	chr3:38,589,553-38,691,164	AX-41229119
SCN5A	rs6599222	chr3:38,589,553-38,691,164	AX-96775727
SCN5A	rs6599222	chr3:38,589,553-38,691,164	AX-97437347
SCN5A	rs199473101	chr3:38,589,553-38,691,164	AX-90066036
SCN5A	---	chr3:38,589,553-38,691,164	AX-90059479
SCN5A	---	chr3:38,589,553-38,691,164	AX-90066275
SCN5A	---	chr3:38,589,553-38,691,164	AX-90062087
SCN5A	---	chr3:38,589,553-38,691,164	AX-90065551
SCN5A	---	chr3:38,589,553-38,691,164	AX-90029659
SCN5A	---	chr3:38,589,553-38,691,164	AX-90035009
SCN5A	---	chr3:38,589,553-38,691,164	AX-90061568
SCN5A	---	chr3:38,589,553-38,691,164	AX-90026327
SCN5A	---	chr3:38,589,553-38,691,164	AX-90031393
SCN5A	---	chr3:38,589,553-38,691,164	AX-90060084
SCN5A	---	chr3:38,589,553-38,691,164	AX-90027230
SCN5A	rs12491987	chr3:38,589,553-38,691,164	AX-41229125
SCN5A	rs187531872	chr3:38,589,553-38,691,164	AX-86727574
SCN5A	---	chr3:38,589,553-38,691,164	AX-90030746
SCN5A	---	chr3:38,589,553-38,691,164	AX-86620981
SCN5A	---	chr3:38,589,553-38,691,164	AX-90064998
SCN5A	---	chr3:38,589,553-38,691,164	AX-90065633
SCN5A	rs13315133	chr3:38,589,553-38,691,164	AX-41229127
SCN5A	rs9873213	chr3:38,589,553-38,691,164	AX-96910517
SCN5A	rs9873213	chr3:38,589,553-38,691,164	AX-97419956
SCN5A	rs13322122	chr3:38,589,553-38,691,164	AX-34309607
SCN5A	rs9853984	chr3:38,589,553-38,691,164	AX-41229129
SCN5A	rs6599223	chr3:38,589,553-38,691,164	AX-41229131
SCN5A	rs76022277	chr3:38,589,553-38,691,164	AX-34309617
SCN5A	rs10718761	chr3:38,589,553-38,691,164	AX-96780369
SCN5A	rs10718761	chr3:38,589,553-38,691,164	AX-97327860
SCN5A	rs182050752	chr3:38,589,553-38,691,164	AX-86691519
SCN5A	---	chr3:38,589,553-38,691,164	AX-90060000
SCN5A	---	chr3:38,589,553-38,691,164	AX-90030554
SCN5A	---	chr3:38,589,553-38,691,164	AX-90027076
SCN5A	rs137854608	chr3:38,589,553-38,691,164	AX-86594659
SCN5A	---	chr3:38,589,553-38,691,164	AX-90063638
SCN5A	rs199473085	chr3:38,589,553-38,691,164	AX-86656868
SCN5A	rs199473084	chr3:38,589,553-38,691,164	AX-86660196
SCN5A	---	chr3:38,589,553-38,691,164	AX-90034105
SCN5A	---	chr3:38,589,553-38,691,164	AX-90031661
SCN5A	---	chr3:38,589,553-38,691,164	AX-90031256
SCN5A	---	chr3:38,589,553-38,691,164	AX-90034397

SCN5A	---	chr3:38,589,553-38,691,164	AX-90062385
SCN5A	---	chr3:38,589,553-38,691,164	AX-90036687
SCN5A	---	chr3:38,589,553-38,691,164	AX-90026328
SCN5A	---	chr3:38,589,553-38,691,164	AX-90062939
SCN5A	---	chr3:38,589,553-38,691,164	AX-90036338
SCN5A	---	chr3:38,589,553-38,691,164	AX-90065160
SCN5A	rs9831389	chr3:38,589,553-38,691,164	AX-41229133
SCN5A	---	chr3:38,589,553-38,691,164	AX-41229135
SCN5A	rs75318896	chr3:38,589,553-38,691,164	AX-34309623
SCN5A	rs6599225	chr3:38,589,553-38,691,164	AX-50383164
SCN5A	rs6599226	chr3:38,589,553-38,691,164	AX-156274134
SCN5A	rs6599226	chr3:38,589,553-38,691,164	AX-156291778
SCN5A	rs6599227	chr3:38,589,553-38,691,164	AX-41229143
SCN5A	rs7430191	chr3:38,589,553-38,691,164	AX-11223772
SCN5A	rs7430191	chr3:38,589,553-38,691,164	AX-97295475
SCN5A	rs9874830	chr3:38,589,553-38,691,164	AX-151146932
SCN5A	rs9874830	chr3:38,589,553-38,691,164	AX-156278787
SCN5A	rs6790718	chr3:38,589,553-38,691,164	AX-41229145
SCN5A	rs6793943	chr3:38,589,553-38,691,164	AX-41229147
SCN5A	rs6791081	chr3:38,589,553-38,691,164	AX-41229149
SCN5A	rs6776383	chr3:38,589,553-38,691,164	AX-156274135
SCN5A	rs6776383	chr3:38,589,553-38,691,164	AX-156291779
SCN5A	rs6786119	chr3:38,589,553-38,691,164	AX-41229151
SCN5A	rs9851962	chr3:38,589,553-38,691,164	AX-41229153
SCN5A	rs41314302	chr3:38,589,553-38,691,164	AX-34309631
SCN5A	---	chr3:38,589,553-38,691,164	AX-90036229
SCN5A	---	chr3:38,589,553-38,691,164	AX-86733651
SCN5A	---	chr3:38,589,553-38,691,164	AX-90063922
SCN5A	rs199473071	chr3:38,589,553-38,691,164	AX-90059370
SCN5A	rs199473072	chr3:38,589,553-38,691,164	AX-86681147
SCN5A	---	chr3:38,589,553-38,691,164	AX-90027029
SCN5A	---	chr3:38,589,553-38,691,164	AX-90064071
SCN5A	rs45620037	chr3:38,589,553-38,691,164	AX-83280554
SCN5A	---	chr3:38,589,553-38,691,164	AX-90077051
SCN5A	---	chr3:38,589,553-38,691,164	AX-90058517
SCN5A	rs41276525	chr3:38,589,553-38,691,164	AX-83379999
SCN5A	---	chr3:38,589,553-38,691,164	AX-90034214
SCN5A	rs370438420	chr3:38,589,553-38,691,164	AX-86552551
SCN5A	rs9856587	chr3:38,589,553-38,691,164	AX-96740225
SCN5A	rs9856587	chr3:38,589,553-38,691,164	AX-96746794
SCN5A	rs6797133	chr3:38,589,553-38,691,164	AX-41229157
SCN5A	rs41314304	chr3:38,589,553-38,691,164	AX-34309637
SCN5A	rs41314314	chr3:38,589,553-38,691,164	AX-34309643
SCN5A	rs41312945	chr3:38,589,553-38,691,164	AX-34309645
SCN5A	rs73054549	chr3:38,589,553-38,691,164	AX-34309647
SCN5A	rs77613778	chr3:38,589,553-38,691,164	AX-34309649
SCN5A	rs7430361	chr3:38,589,553-38,691,164	AX-34309651
SCN5A	rs7431920	chr3:38,589,553-38,691,164	AX-34309653
SCN5A	rs7433206	chr3:38,589,553-38,691,164	AX-156291780
SCN5A	rs7433206	chr3:38,589,553-38,691,164	AX-96861710
SCN5A	rs11710077	chr3:38,589,553-38,691,164	AX-41229165
SCN5A	rs11926428	chr3:38,589,553-38,691,164	AX-41229167
SCN5A	rs73054554	chr3:38,589,553-38,691,164	AX-156291781
SCN5A	rs76337192	chr3:38,589,553-38,691,164	AX-14300746
SCN5A	rs34767956	chr3:38,589,553-38,691,164	AX-34309659

SCN5A	rs35349722	chr3:38,589,553-38,691,164	AX-34309661
SCN5A	rs199917358	chr3:38,589,553-38,691,164	AX-97471835
SCN5A	rs62242801	chr3:38,589,553-38,691,164	AX-34309663
SCN5A	rs62242802	chr3:38,589,553-38,691,164	AX-34309665
SCN5A	rs73825705	chr3:38,589,553-38,691,164	AX-14300747
SCN5A	rs7637849	chr3:38,589,553-38,691,164	AX-41229169
SCN5A	rs7433085	chr3:38,589,553-38,691,164	AX-34309669
SCN5A	rs55824920	chr3:38,589,553-38,691,164	AX-34309671
SCN5A	rs80101046	chr3:38,589,553-38,691,164	AX-34309673
SCN5A	rs12498069	chr3:38,589,553-38,691,164	AX-41229173
SCN5A	rs9832895	chr3:38,589,553-38,691,164	AX-41229175
SCN5A	rs397514252	chr3:38,589,553-38,691,164	AX-90079705
SCN5A	---	chr3:38,589,553-38,691,164	AX-90061323
SCN5A	rs199473559	chr3:38,589,553-38,691,164	AX-90055214
SCN5A	---	chr3:38,589,553-38,691,164	AX-90055215
SCN5A	---	chr3:38,589,553-38,691,164	AX-86574062
SCN5A	---	chr3:38,589,553-38,691,164	AX-86607294
SCN5A	---	chr3:38,589,553-38,691,164	AX-90032053
SCN5A	---	chr3:38,589,553-38,691,164	AX-90056236
SCN5A	rs192113333	chr3:38,589,553-38,691,164	AX-82902709
SCN5A	---	chr3:38,589,553-38,691,164	AX-90055216
SCN5A	---	chr3:38,589,553-38,691,164	AX-90059595
SCN5A	---	chr3:38,589,553-38,691,164	AX-90028501
SCN5A	---	chr3:38,589,553-38,691,164	AX-90063410
SCN5A	---	chr3:38,589,553-38,691,164	AX-90081484
SCN5A	---	chr3:38,589,553-38,691,164	AX-90046493
SCN5A	rs6599228	chr3:38,589,553-38,691,164	AX-41229177
SCN5A	rs6781731	chr3:38,589,553-38,691,164	AX-50383172
SCN5A	---	chr3:38,589,553-38,691,164	AX-86568177
SCN5A	---	chr3:38,589,553-38,691,164	AX-90057345
SCN5A	---	chr3:38,589,553-38,691,164	AX-90026332
SCN5A	---	chr3:38,589,553-38,691,164	AX-90055218
SCN5A	---	chr3:38,589,553-38,691,164	AX-86655656
SCN5A	---	chr3:38,589,553-38,691,164	AX-90076157
SCN5A	rs199473060	chr3:38,589,553-38,691,164	AX-86707946
SCN5A	---	chr3:38,589,553-38,691,164	AX-90062057
SCN5A	---	chr3:38,589,553-38,691,164	AX-90062992
SCN5A	rs368678204	chr3:38,589,553-38,691,164	AX-86623271
SCN5A	rs7431305	chr3:38,589,553-38,691,164	AX-96769724
SCN5A	rs7431305	chr3:38,589,553-38,691,164	AX-96789031
SCN5A	rs3934936	chr3:38,589,553-38,691,164	AX-96775768
SCN5A	rs3934936	chr3:38,589,553-38,691,164	AX-96782295
SCN5A	rs3934937	chr3:38,589,553-38,691,164	AX-156274137
SCN5A	rs72862756	chr3:38,589,553-38,691,164	AX-34309701
SCN5A	rs9876660	chr3:38,589,553-38,691,164	AX-41229185
SCN5A	rs78615060	chr3:38,589,553-38,691,164	AX-34309703
SCN5A	rs7624535	chr3:38,589,553-38,691,164	AX-41229189
SCN5A	rs75398812	chr3:38,589,553-38,691,164	AX-38048533
SCN5A	rs79352669	chr3:38,589,553-38,691,164	AX-38048535
SCN5A	rs78532995	chr3:38,589,553-38,691,164	AX-96765034
SCN5A	rs75882699	chr3:38,589,553-38,691,164	AX-96861005
SCN5A	rs7633974	chr3:38,589,553-38,691,164	AX-41229193
SCN5A	rs7627552	chr3:38,589,553-38,691,164	AX-96772249
SCN5A	rs7627552	chr3:38,589,553-38,691,164	AX-96787281
SCN5A	rs62242807	chr3:38,589,553-38,691,164	AX-156295091

SCN5A	rs62242807	chr3:38,589,553-38,691,164	AX-96842413
SCN5A	rs76736254	chr3:38,589,553-38,691,164	AX-156274138
SCN5A	rs76736254	chr3:38,589,553-38,691,164	AX-97301473
SCN5A	rs76040087	chr3:38,589,553-38,691,164	AX-34309707
SCN5A	rs13314361	chr3:38,589,553-38,691,164	AX-41229197
SCN5A	rs7432590	chr3:38,589,553-38,691,164	AX-96813509
SCN5A	rs7432590	chr3:38,589,553-38,691,164	AX-96815589
SCN5A	rs34712780	chr3:38,589,553-38,691,164	AX-96773427
SCN5A	rs6795580	chr3:38,589,553-38,691,164	AX-41229201
SCN5A	rs144207380	chr3:38,589,553-38,691,164	AX-156298098
SCN5A	rs7647031	chr3:38,589,553-38,691,164	AX-96916265
SCN5A	rs7647031	chr3:38,589,553-38,691,164	AX-96918472
SCN5A	rs41258324	chr3:38,589,553-38,691,164	AX-156274139
SCN5A	rs9818148	chr3:38,589,553-38,691,164	AX-34309723
SCN5A	rs7374891	chr3:38,589,553-38,691,164	AX-156291784
SCN5A	rs7374891	chr3:38,589,553-38,691,164	AX-96867177
SCN5A	rs112041752	chr3:38,589,553-38,691,164	AX-148495059
SCN5A	rs112041752	chr3:38,589,553-38,691,164	AX-156278788
SCN5A	rs7375123	chr3:38,589,553-38,691,164	AX-34309735
SCN5A	rs9848555	chr3:38,589,553-38,691,164	AX-34309737
SCN5A	rs77604382	chr3:38,589,553-38,691,164	AX-34309741
SCN5A	rs7373076	chr3:38,589,553-38,691,164	AX-34309743
SCN5A	rs7431820	chr3:38,589,553-38,691,164	AX-34309745
SCN5A	rs73056438	chr3:38,589,553-38,691,164	AX-34309749
SCN5A	rs45567533	chr3:38,589,553-38,691,164	AX-34309751
SCN5A	rs41312963	chr3:38,589,553-38,691,164	AX-34309753
SCN5A	---	chr3:38,589,553-38,691,164	AX-90072955
SCN5A	rs199473059	chr3:38,589,553-38,691,164	AX-86571862
SCN5A	---	chr3:38,589,553-38,691,164	AX-90057484
SCN5A	---	chr3:38,589,553-38,691,164	AX-90057456
SCN5A	---	chr3:38,589,553-38,691,164	AX-90056202
SCN5A	---	chr3:38,589,553-38,691,164	AX-90035174
SCN5A	---	chr3:38,589,553-38,691,164	AX-90028538
SCN5A	---	chr3:38,589,553-38,691,164	AX-90063745
SCN5A	---	chr3:38,589,553-38,691,164	AX-90056743
SCN5A	rs199473054	chr3:38,589,553-38,691,164	AX-86585308
SCN5A	---	chr3:38,589,553-38,691,164	AX-90060273
SCN5A	---	chr3:38,589,553-38,691,164	AX-90059103
SCN5A	rs7627488	chr3:38,589,553-38,691,164	AX-34309755
SCN5A	rs7636280	chr3:38,589,553-38,691,164	AX-96952601
SCN5A	rs7636280	chr3:38,589,553-38,691,164	AX-97386194
SCN5A	rs6599229	chr3:38,589,553-38,691,164	AX-11574597
SCN5A	rs7374392	chr3:38,589,553-38,691,164	AX-34309761
SCN5A	rs7432959	chr3:38,589,553-38,691,164	AX-41229205
SCN5A	rs56053699	chr3:38,589,553-38,691,164	AX-34309767
SCN5A	rs7433003	chr3:38,589,553-38,691,164	AX-34309769
SCN5A	rs35199243	chr3:38,589,553-38,691,164	AX-34309773
SCN5A	---	chr3:38,589,553-38,691,164	AX-90030465
SCN5A	---	chr3:38,589,553-38,691,164	AX-90058917
SCN5A	---	chr3:38,589,553-38,691,164	AX-90057552
SCN5A	---	chr3:38,589,553-38,691,164	AX-90051344
SCN5A	---	chr3:38,589,553-38,691,164	AX-90055219
SCN5A	---	chr3:38,589,553-38,691,164	AX-90030008
SCN5A	---	chr3:38,589,553-38,691,164	AX-90061479
SCN5A	---	chr3:38,589,553-38,691,164	AX-86627932

SCN5A	---	chr3:38,589,553-38,691,164	AX-90043503
SCN5A	rs6791924	chr3:38,589,553-38,691,164	AX-41229207
SCN5A	rs6599230	chr3:38,589,553-38,691,164	AX-34309781
SCN5A	rs562675882	chr3:38,589,553-38,691,164	AX-90059626
SCN5A	rs41311087	chr3:38,589,553-38,691,164	AX-90037920
SCN5A	rs199473044	chr3:38,589,553-38,691,164	AX-90060236
SCN5A	---	chr3:38,589,553-38,691,164	AX-90063416
SCN5A	---	chr3:38,589,553-38,691,164	AX-90026337
SCN5A	---	chr3:38,589,553-38,691,164	AX-90055223
SCN5A	rs11719543	chr3:38,589,553-38,691,164	AX-156274140
SCN5A	rs11719543	chr3:38,589,553-38,691,164	AX-156291785
SCN5A	rs11720524	chr3:38,589,553-38,691,164	AX-41229209
SCN5A	rs13060541	chr3:38,589,553-38,691,164	AX-41229213
SCN5A	rs11710223	chr3:38,589,553-38,691,164	AX-156274141
SCN5A	rs11710223	chr3:38,589,553-38,691,164	AX-156291786
SCN5A	rs7651854	chr3:38,589,553-38,691,164	AX-96867189
SCN5A	rs7651854	chr3:38,589,553-38,691,164	AX-97260867
SCN5A	rs6768135	chr3:38,589,553-38,691,164	AX-147911127
SCN5A	rs35430286	chr3:38,589,553-38,691,164	AX-34309795
SCN5A	rs7427106	chr3:38,589,553-38,691,164	AX-11624462
SCN5A	rs7427119	chr3:38,589,553-38,691,164	AX-156274143
SCN5A	rs7427119	chr3:38,589,553-38,691,164	AX-156291788
SCN5A	rs34636473	chr3:38,589,553-38,691,164	AX-34309801
SCN5A	rs111784596	chr3:38,589,553-38,691,164	AX-34309805
SCN5A	rs13073578	chr3:38,589,553-38,691,164	AX-41229221
SCN5A	rs41314851	chr3:38,589,553-38,691,164	AX-11494558
SCN5A	rs7373102	chr3:38,589,553-38,691,164	AX-41229225
SCN5A	rs35029027	chr3:38,589,553-38,691,164	AX-34309811
SCN5A	rs9311195	chr3:38,589,553-38,691,164	AX-11679996
SCN5A	rs73056454	chr3:38,589,553-38,691,164	AX-34309813
SCN5A	rs6763048	chr3:38,589,553-38,691,164	AX-41229229
SCN5A	rs9875443	chr3:38,589,553-38,691,164	AX-156281844
SCN5A	rs9875443	chr3:38,589,553-38,691,164	AX-96934123
SCN5A	rs41314861	chr3:38,589,553-38,691,164	AX-34309817
SCN5A	rs9880327	chr3:38,589,553-38,691,164	AX-34309819
SCN5A	rs13097780	chr3:38,589,553-38,691,164	AX-34309821
SCN5A	rs112401327	chr3:38,589,553-38,691,164	AX-34309823
SCN5A	rs80172327	chr3:38,589,553-38,691,164	AX-34309831
SCN5A	rs6768664	chr3:38,589,553-38,691,164	AX-11584029
SCN5A	rs6599231	chr3:38,589,553-38,691,164	AX-34309837
SCN5A	rs7427574	chr3:38,589,553-38,691,164	AX-34309839
SCN5A	rs57458156	chr3:38,589,553-38,691,164	AX-156274144
SCN5A	rs57458156	chr3:38,589,553-38,691,164	AX-156291789
SCN5A	---	chr3:38,589,553-38,691,164	AX-34309847
SCN5A	rs544071590	chr3:38,589,553-38,691,164	AX-151443905
SCN5A	rs544071590	chr3:38,589,553-38,691,164	AX-156298434
SCN5A	rs112058833	chr3:38,589,553-38,691,164	AX-34309851
SCN5A	rs7372712	chr3:38,589,553-38,691,164	AX-11623382
SCN5A	rs11711288	chr3:38,589,553-38,691,164	AX-34309857
SCN5A	rs55812368	chr3:38,589,553-38,691,164	AX-34309859
SCN5A	rs9856387	chr3:38,589,553-38,691,164	AX-34309861
SCN5A	rs34682488	chr3:38,589,553-38,691,164	AX-14300760
SCN5A	rs9841023	chr3:38,589,553-38,691,164	AX-34309865
SCN5A	rs55742514	chr3:38,589,553-38,691,164	AX-34309867
SCN5A	rs9841329	chr3:38,589,553-38,691,164	AX-34309869

SCN5A	rs79160746	chr3:38,589,553-38,691,164	AX-34309883
SCN5A	rs9809363	chr3:38,589,553-38,691,164	AX-41229239
SCN5A	rs9814083	chr3:38,589,553-38,691,164	AX-34309891
SCN5A	rs72862787	chr3:38,589,553-38,691,164	AX-34309893
SCN5A	rs72862788	chr3:38,589,553-38,691,164	AX-34309895
SCN5A	rs41310234	chr3:38,589,553-38,691,164	AX-34309897
SGCD	rs73297590	chr5:155,135,063-156,194,798	AX-14941377
SGCD	rs56829274	chr5:155,135,063-156,194,798	AX-14941378
SGCD	rs12655253	chr5:155,135,063-156,194,798	AX-14941379
SGCD	rs7733760	chr5:155,135,063-156,194,798	AX-12638590
SGCD	rs13160569	chr5:155,135,063-156,194,798	AX-123105452
SGCD	rs28601600	chr5:155,135,063-156,194,798	AX-156275442
SGCD	rs28601600	chr5:155,135,063-156,194,798	AX-156292652
SGCD	rs200057738	chr5:155,135,063-156,194,798	AX-153324007
SGCD	rs200057738	chr5:155,135,063-156,194,798	AX-156284310
SGCD	rs138321378	chr5:155,135,063-156,194,798	AX-120224135
SGCD	rs138321378	chr5:155,135,063-156,194,798	AX-156285546
SGCD	rs150903903	chr5:155,135,063-156,194,798	AX-148201780
SGCD	rs150903903	chr5:155,135,063-156,194,798	AX-156297659
SGCD	rs145586715	chr5:155,135,063-156,194,798	AX-148536585
SGCD	rs145586715	chr5:155,135,063-156,194,798	AX-156284047
SGCD	rs148875892	chr5:155,135,063-156,194,798	AX-148376300
SGCD	rs28971928	chr5:155,135,063-156,194,798	AX-14941384
SGCD	rs34330815	chr5:155,135,063-156,194,798	AX-120887749
SGCD	rs34330815	chr5:155,135,063-156,194,798	AX-156275443
SGCD	rs73810312	chr5:155,135,063-156,194,798	AX-14941386
SGCD	rs79266455	chr5:155,135,063-156,194,798	AX-35168059
SGCD	rs4077765	chr5:155,135,063-156,194,798	AX-156296921
SGCD	rs56324461	chr5:155,135,063-156,194,798	AX-14941393
SGCD	rs4434365	chr5:155,135,063-156,194,798	AX-11505506
SGCD	rs62382293	chr5:155,135,063-156,194,798	AX-35168071
SGCD	rs12187106	chr5:155,135,063-156,194,798	AX-14941398
SGCD	rs148917570	chr5:155,135,063-156,194,798	AX-156286378
SGCD	rs7379262	chr5:155,135,063-156,194,798	AX-14941400
SGCD	rs141033817	chr5:155,135,063-156,194,798	AX-120172639
SGCD	rs7446885	chr5:155,135,063-156,194,798	AX-156275444
SGCD	rs7446885	chr5:155,135,063-156,194,798	AX-156292653
SGCD	rs62382294	chr5:155,135,063-156,194,798	AX-35168079
SGCD	rs6556604	chr5:155,135,063-156,194,798	AX-14941405
SGCD	rs74764373	chr5:155,135,063-156,194,798	AX-14941406
SGCD	rs113068515	chr5:155,135,063-156,194,798	AX-38253903
SGCD	rs57306484	chr5:155,135,063-156,194,798	AX-14941407
SGCD	rs79905952	chr5:155,135,063-156,194,798	AX-14941410
SGCD	rs73297601	chr5:155,135,063-156,194,798	AX-35168081
SGCD	---	chr5:155,135,063-156,194,798	AX-153331457
SGCD	rs10063232	chr5:155,135,063-156,194,798	AX-156275445
SGCD	rs10063232	chr5:155,135,063-156,194,798	AX-96557002
SGCD	rs7717301	chr5:155,135,063-156,194,798	AX-11640796
SGCD	rs79363839	chr5:155,135,063-156,194,798	AX-14941413
SGCD	rs6865768	chr5:155,135,063-156,194,798	AX-156275446
SGCD	rs6865768	chr5:155,135,063-156,194,798	AX-156292654
SGCD	rs6865887	chr5:155,135,063-156,194,798	AX-41643607
SGCD	rs73810314	chr5:155,135,063-156,194,798	AX-14941416
SGCD	rs35102821	chr5:155,135,063-156,194,798	AX-123077771
SGCD	rs35102821	chr5:155,135,063-156,194,798	AX-123077772

SGCD	rs56102724	chr5:155,135,063-156,194,798	AX-14941420
SGCD	rs76035004	chr5:155,135,063-156,194,798	AX-14941421
SGCD	rs4608908	chr5:155,135,063-156,194,798	AX-14941422
SGCD	rs11134451	chr5:155,135,063-156,194,798	AX-14941425
SGCD	rs11134451	chr5:155,135,063-156,194,798	AX-156275447
SGCD	rs4518375	chr5:155,135,063-156,194,798	AX-156292655
SGCD	rs12173162	chr5:155,135,063-156,194,798	AX-14941427
SGCD	rs77549720	chr5:155,135,063-156,194,798	AX-14941428
SGCD	rs12173249	chr5:155,135,063-156,194,798	AX-11194116
SGCD	rs73810315	chr5:155,135,063-156,194,798	AX-14941430
SGCD	rs11134474	chr5:155,135,063-156,194,798	AX-41643611
SGCD	rs11134484	chr5:155,135,063-156,194,798	AX-41643613
SGCD	rs4357020	chr5:155,135,063-156,194,798	AX-41643615
SGCD	rs113490650	chr5:155,135,063-156,194,798	AX-14941431
SGCD	rs113490650	chr5:155,135,063-156,194,798	AX-153352653
SGCD	rs7446674	chr5:155,135,063-156,194,798	AX-41643617
SGCD	rs62382310	chr5:155,135,063-156,194,798	AX-14941434
SGCD	rs13158911	chr5:155,135,063-156,194,798	AX-14941436
SGCD	rs35497401	chr5:155,135,063-156,194,798	AX-153254732
SGCD	rs35497401	chr5:155,135,063-156,194,798	AX-156297977
SGCD	rs4616876	chr5:155,135,063-156,194,798	AX-11513783
SGCD	rs74460347	chr5:155,135,063-156,194,798	AX-35168099
SGCD	rs62382311	chr5:155,135,063-156,194,798	AX-14941439
SGCD	rs62382312	chr5:155,135,063-156,194,798	AX-35168103
SGCD	rs77073288	chr5:155,135,063-156,194,798	AX-14941441
SGCD	rs78161813	chr5:155,135,063-156,194,798	AX-14941442
SGCD	rs11948011	chr5:155,135,063-156,194,798	AX-12423848
SGCD	rs7714002	chr5:155,135,063-156,194,798	AX-14941446
SGCD	rs11741070	chr5:155,135,063-156,194,798	AX-41643621
SGCD	rs115113993	chr5:155,135,063-156,194,798	AX-14941447
SGCD	rs75213788	chr5:155,135,063-156,194,798	AX-14941448
SGCD	rs12514871	chr5:155,135,063-156,194,798	AX-35168109
SGCD	rs74365071	chr5:155,135,063-156,194,798	AX-35168111
SGCD	rs12187392	chr5:155,135,063-156,194,798	AX-156275449
SGCD	rs12187392	chr5:155,135,063-156,194,798	AX-156292656
SGCD	rs76517429	chr5:155,135,063-156,194,798	AX-14941454
SGCD	rs12186863	chr5:155,135,063-156,194,798	AX-14941455
SGCD	rs62382313	chr5:155,135,063-156,194,798	AX-156275450
SGCD	rs6876341	chr5:155,135,063-156,194,798	AX-35168117
SGCD	rs9942327	chr5:155,135,063-156,194,798	AX-35168119
SGCD	rs34507862	chr5:155,135,063-156,194,798	AX-148257851
SGCD	rs34507862	chr5:155,135,063-156,194,798	AX-156275451
SGCD	rs34760156	chr5:155,135,063-156,194,798	AX-35168123
SGCD	rs76064151	chr5:155,135,063-156,194,798	AX-14941458
SGCD	rs10462910	chr5:155,135,063-156,194,798	AX-14941459
SGCD	rs10462911	chr5:155,135,063-156,194,798	AX-12389432
SGCD	rs114183248	chr5:155,135,063-156,194,798	AX-14941464
SGCD	rs4526089	chr5:155,135,063-156,194,798	AX-156292657
SGCD	rs17524681	chr5:155,135,063-156,194,798	AX-156268145
SGCD	rs17524681	chr5:155,135,063-156,194,798	AX-156288011
SGCD	rs78189004	chr5:155,135,063-156,194,798	AX-14941472
SGCD	rs6895293	chr5:155,135,063-156,194,798	AX-156268146
SGCD	rs6875928	chr5:155,135,063-156,194,798	AX-35168139
SGCD	rs114271976	chr5:155,135,063-156,194,798	AX-156288013
SGCD	rs13153098	chr5:155,135,063-156,194,798	AX-35168141

SGCD	rs77651304	chr5:155,135,063-156,194,798	AX-14941475
SGCD	rs78338115	chr5:155,135,063-156,194,798	AX-14941478
SGCD	rs35751638	chr5:155,135,063-156,194,798	AX-35168145
SGCD	rs4273588	chr5:155,135,063-156,194,798	AX-148389029
SGCD	rs4273588	chr5:155,135,063-156,194,798	AX-156268148
SGCD	rs12521468	chr5:155,135,063-156,194,798	AX-35168149
SGCD	rs115349939	chr5:155,135,063-156,194,798	AX-148719776
SGCD	rs115349939	chr5:155,135,063-156,194,798	AX-156282262
SGCD	rs62382317	chr5:155,135,063-156,194,798	AX-153342175
SGCD	rs62382317	chr5:155,135,063-156,194,798	AX-156284048
SGCD	rs17052864	chr5:155,135,063-156,194,798	AX-14941482
SGCD	rs115963734	chr5:155,135,063-156,194,798	AX-148660649
SGCD	rs115963734	chr5:155,135,063-156,194,798	AX-156268149
SGCD	rs114131615	chr5:155,135,063-156,194,798	AX-156268150
SGCD	rs114131615	chr5:155,135,063-156,194,798	AX-156288014
SGCD	rs62382318	chr5:155,135,063-156,194,798	AX-14941489
SGCD	rs7447243	chr5:155,135,063-156,194,798	AX-156268151
SGCD	rs7447243	chr5:155,135,063-156,194,798	AX-156288015
SGCD	rs79365858	chr5:155,135,063-156,194,798	AX-14941492
SGCD	rs11744001	chr5:155,135,063-156,194,798	AX-14941494
SGCD	rs116017842	chr5:155,135,063-156,194,798	AX-122780368
SGCD	rs116017842	chr5:155,135,063-156,194,798	AX-148810565
SGCD	rs2127371	chr5:155,135,063-156,194,798	AX-156268152
SGCD	rs140901312	chr5:155,135,063-156,194,798	AX-88783562
SGCD	rs140901312	chr5:155,135,063-156,194,798	AX-96555555
SGCD	rs17052865	chr5:155,135,063-156,194,798	AX-14941497
SGCD	rs17052868	chr5:155,135,063-156,194,798	AX-156282263
SGCD	rs17052868	chr5:155,135,063-156,194,798	AX-156296922
SGCD	rs6866497	chr5:155,135,063-156,194,798	AX-41643635
SGCD	rs10476284	chr5:155,135,063-156,194,798	AX-14941499
SGCD	rs57521482	chr5:155,135,063-156,194,798	AX-14941504
SGCD	rs7379436	chr5:155,135,063-156,194,798	AX-156288017
SGCD	rs80088893	chr5:155,135,063-156,194,798	AX-148606065
SGCD	rs80088893	chr5:155,135,063-156,194,798	AX-156268154
SGCD	rs2046038	chr5:155,135,063-156,194,798	AX-35168169
SGCD	rs62382322	chr5:155,135,063-156,194,798	AX-156268155
SGCD	rs62382322	chr5:155,135,063-156,194,798	AX-156288018
SGCD	rs1479555	chr5:155,135,063-156,194,798	AX-35168173
SGCD	---	chr5:155,135,063-156,194,798	AX-156285894
SGCD	rs1479554	chr5:155,135,063-156,194,798	AX-156268156
SGCD	rs1479554	chr5:155,135,063-156,194,798	AX-156288019
SGCD	rs1351280	chr5:155,135,063-156,194,798	AX-14941517
SGCD	rs17052874	chr5:155,135,063-156,194,798	AX-156296923
SGCD	rs7379212	chr5:155,135,063-156,194,798	AX-14941518
SGCD	rs11135083	chr5:155,135,063-156,194,798	AX-41643647
SGCD	rs11135084	chr5:155,135,063-156,194,798	AX-41643649
SGCD	rs6885265	chr5:155,135,063-156,194,798	AX-14941521
SGCD	rs7447880	chr5:155,135,063-156,194,798	AX-14941524
SGCD	rs77571180	chr5:155,135,063-156,194,798	AX-156275453
SGCD	rs77571180	chr5:155,135,063-156,194,798	AX-156292658
SGCD	rs76605141	chr5:155,135,063-156,194,798	AX-14941526
SGCD	rs7444825	chr5:155,135,063-156,194,798	AX-156275454
SGCD	rs7444825	chr5:155,135,063-156,194,798	AX-156292659
SGCD	rs1382878	chr5:155,135,063-156,194,798	AX-14941529
SGCD	rs62382323	chr5:155,135,063-156,194,798	AX-14941530

SGCD	rs62382324	chr5:155,135,063-156,194,798	AX-35168189
SGCD	rs13154433	chr5:155,135,063-156,194,798	AX-148191653
SGCD	rs13154433	chr5:155,135,063-156,194,798	AX-96864023
SGCD	rs10462919	chr5:155,135,063-156,194,798	AX-148367430
SGCD	rs10462919	chr5:155,135,063-156,194,798	AX-148719718
SGCD	rs7378633	chr5:155,135,063-156,194,798	AX-148253289
SGCD	rs62382325	chr5:155,135,063-156,194,798	AX-114035808
SGCD	rs62382325	chr5:155,135,063-156,194,798	AX-148503734
SGCD	rs13188692	chr5:155,135,063-156,194,798	AX-151172261
SGCD	rs13188692	chr5:155,135,063-156,194,798	AX-156284049
SGCD	rs10462920	chr5:155,135,063-156,194,798	AX-12389433
SGCD	rs13170805	chr5:155,135,063-156,194,798	AX-11242717
SGCD	rs77204876	chr5:155,135,063-156,194,798	AX-14941534
SGCD	rs115613946	chr5:155,135,063-156,194,798	AX-35168191
SGCD	rs7379008	chr5:155,135,063-156,194,798	AX-14941536
SGCD	rs6556478	chr5:155,135,063-156,194,798	AX-156268157
SGCD	rs6556478	chr5:155,135,063-156,194,798	AX-156288020
SGCD	rs72812192	chr5:155,135,063-156,194,798	AX-14941539
SGCD	rs7712917	chr5:155,135,063-156,194,798	AX-14941541
SGCD	---	chr5:155,135,063-156,194,798	AX-14941542
SGCD	rs77656837	chr5:155,135,063-156,194,798	AX-14941544
SGCD	rs17052889	chr5:155,135,063-156,194,798	AX-41643655
SGCD	rs72812194	chr5:155,135,063-156,194,798	AX-14941545
SGCD	rs7445962	chr5:155,135,063-156,194,798	AX-156282265
SGCD	rs7705775	chr5:155,135,063-156,194,798	AX-50448216
SGCD	rs10515729	chr5:155,135,063-156,194,798	AX-41643657
SGCD	rs10515730	chr5:155,135,063-156,194,798	AX-11114993
SGCD	---	chr5:155,135,063-156,194,798	AX-156286115
SGCD	rs17052899	chr5:155,135,063-156,194,798	AX-12487852
SGCD	rs111308348	chr5:155,135,063-156,194,798	AX-148563439
SGCD	rs111308348	chr5:155,135,063-156,194,798	AX-156284050
SGCD	rs145171516	chr5:155,135,063-156,194,798	AX-148810725
SGCD	rs145171516	chr5:155,135,063-156,194,798	AX-156284051
SGCD	rs35125194	chr5:155,135,063-156,194,798	AX-148670712
SGCD	rs35125194	chr5:155,135,063-156,194,798	AX-156275455
SGCD	rs35483609	chr5:155,135,063-156,194,798	AX-148259797
SGCD	rs11743011	chr5:155,135,063-156,194,798	AX-148573375
SGCD	rs11743011	chr5:155,135,063-156,194,798	AX-156268158
SGCD	rs77675863	chr5:155,135,063-156,194,798	AX-14941550
SGCD	rs1545773	chr5:155,135,063-156,194,798	AX-12468886
SGCD	rs980914	chr5:155,135,063-156,194,798	AX-35168203
SGCD	rs2569028	chr5:155,135,063-156,194,798	AX-14941553
SGCD	rs2569029	chr5:155,135,063-156,194,798	AX-156268159
SGCD	rs2569029	chr5:155,135,063-156,194,798	AX-156288021
SGCD	rs75698624	chr5:155,135,063-156,194,798	AX-14941555
SGCD	rs78268325	chr5:155,135,063-156,194,798	AX-14941558
SGCD	rs76516504	chr5:155,135,063-156,194,798	AX-14941559
SGCD	rs2217640	chr5:155,135,063-156,194,798	AX-14941560
SGCD	rs11738666	chr5:155,135,063-156,194,798	AX-14941562
SGCD	rs61332716	chr5:155,135,063-156,194,798	AX-14941564
SGCD	rs6420106	chr5:155,135,063-156,194,798	AX-14941565
SGCD	rs114609185	chr5:155,135,063-156,194,798	AX-156275457
SGCD	rs114609185	chr5:155,135,063-156,194,798	AX-156292660
SGCD	rs2569030	chr5:155,135,063-156,194,798	AX-156275458
SGCD	rs2569030	chr5:155,135,063-156,194,798	AX-156292661

SGCD	rs111229407	chr5:155,135,063-156,194,798	AX-14941568
SGCD	rs114402302	chr5:155,135,063-156,194,798	AX-156275459
SGCD	rs114402302	chr5:155,135,063-156,194,798	AX-156292662
SGCD	rs75873627	chr5:155,135,063-156,194,798	AX-14941570
SGCD	rs2619725	chr5:155,135,063-156,194,798	AX-14941572
SGCD	rs2569031	chr5:155,135,063-156,194,798	AX-12532144
SGCD	rs62382330	chr5:155,135,063-156,194,798	AX-14941573
SGCD	rs2619724	chr5:155,135,063-156,194,798	AX-50448219
SGCD	rs2569032	chr5:155,135,063-156,194,798	AX-11398918
SGCD	rs2619723	chr5:155,135,063-156,194,798	AX-14941580
SGCD	rs1432814	chr5:155,135,063-156,194,798	AX-156275460
SGCD	rs1432814	chr5:155,135,063-156,194,798	AX-156292663
SGCD	rs77676362	chr5:155,135,063-156,194,798	AX-14941584
SGCD	rs76433976	chr5:155,135,063-156,194,798	AX-14941585
SGCD	rs76857637	chr5:155,135,063-156,194,798	AX-14941588
SGCD	rs2619722	chr5:155,135,063-156,194,798	AX-41643675
SGCD	rs6876095	chr5:155,135,063-156,194,798	AX-148424400
SGCD	rs6876095	chr5:155,135,063-156,194,798	AX-156275461
SGCD	rs10434699	chr5:155,135,063-156,194,798	AX-153332287
SGCD	rs10434699	chr5:155,135,063-156,194,798	AX-156275462
SGCD	rs10434700	chr5:155,135,063-156,194,798	AX-156282266
SGCD	rs2619721	chr5:155,135,063-156,194,798	AX-50448220
SGCD	rs116136949	chr5:155,135,063-156,194,798	AX-14941592
SGCD	rs11291385	chr5:155,135,063-156,194,798	AX-11154766
SGCD	rs114944823	chr5:155,135,063-156,194,798	AX-14941593
SGCD	rs79108524	chr5:155,135,063-156,194,798	AX-14941594
SGCD	rs2619720	chr5:155,135,063-156,194,798	AX-14941595
SGCD	rs78128930	chr5:155,135,063-156,194,798	AX-14941598
SGCD	rs114221206	chr5:155,135,063-156,194,798	AX-14941600
SGCD	rs2619718	chr5:155,135,063-156,194,798	AX-14941602
SGCD	rs3846688	chr5:155,135,063-156,194,798	AX-156275463
SGCD	rs3846688	chr5:155,135,063-156,194,798	AX-156292664
SGCD	rs78154039	chr5:155,135,063-156,194,798	AX-14941606
SGCD	rs11745289	chr5:155,135,063-156,194,798	AX-14941607
SGCD	rs74330127	chr5:155,135,063-156,194,798	AX-14941609
SGCD	rs6556568	chr5:155,135,063-156,194,798	AX-14941610
SGCD	rs2569033	chr5:155,135,063-156,194,798	AX-122583289
SGCD	rs2569033	chr5:155,135,063-156,194,798	AX-156292665
SGCD	rs78767053	chr5:155,135,063-156,194,798	AX-14941612
SGCD	rs202034555	chr5:155,135,063-156,194,798	AX-149013376
SGCD	rs202034555	chr5:155,135,063-156,194,798	AX-153329929
SGCD	rs200342209	chr5:155,135,063-156,194,798	AX-121282644
SGCD	rs201199660	chr5:155,135,063-156,194,798	AX-153271209
SGCD	rs4362928	chr5:155,135,063-156,194,798	AX-12570786
SGCD	rs9313906	chr5:155,135,063-156,194,798	AX-41643687
SGCD	rs9313907	chr5:155,135,063-156,194,798	AX-14941615
SGCD	rs1432816	chr5:155,135,063-156,194,798	AX-14941620
SGCD	rs1432817	chr5:155,135,063-156,194,798	AX-86335265
SGCD	rs6875111	chr5:155,135,063-156,194,798	AX-11591438
SGCD	rs10064619	chr5:155,135,063-156,194,798	AX-156275464
SGCD	rs34817459	chr5:155,135,063-156,194,798	AX-156275465
SGCD	rs34817459	chr5:155,135,063-156,194,798	AX-156292666
SGCD	rs2619717	chr5:155,135,063-156,194,798	AX-14941626
SGCD	rs11135225	chr5:155,135,063-156,194,798	AX-14941635
SGCD	rs1432818	chr5:155,135,063-156,194,798	AX-156282267

SGCD	rs1432818	chr5:155,135,063-156,194,798	AX-156296926
SGCD	rs142415348	chr5:155,135,063-156,194,798	AX-153329391
SGCD	rs115051635	chr5:155,135,063-156,194,798	AX-14941639
SGCD	rs117423930	chr5:155,135,063-156,194,798	AX-148667884
SGCD	rs117423930	chr5:155,135,063-156,194,798	AX-156282268
SGCD	rs62382351	chr5:155,135,063-156,194,798	AX-35168243
SGCD	rs116324036	chr5:155,135,063-156,194,798	AX-14941642
SGCD	rs2569034	chr5:155,135,063-156,194,798	AX-14941644
SGCD	rs11135229	chr5:155,135,063-156,194,798	AX-14941647
SGCD	rs199634853	chr5:155,135,063-156,194,798	AX-153335450
SGCD	---	chr5:155,135,063-156,194,798	AX-119489217
SGCD	rs2619715	chr5:155,135,063-156,194,798	AX-14941649
SGCD	rs74753416	chr5:155,135,063-156,194,798	AX-14941650
SGCD	rs114405775	chr5:155,135,063-156,194,798	AX-153341311
SGCD	rs114405775	chr5:155,135,063-156,194,798	AX-156275466
SGCD	rs11743670	chr5:155,135,063-156,194,798	AX-156275467
SGCD	rs11743670	chr5:155,135,063-156,194,798	AX-156292667
SGCD	rs11743693	chr5:155,135,063-156,194,798	AX-148672338
SGCD	rs11743693	chr5:155,135,063-156,194,798	AX-156275468
SGCD	rs12521447	chr5:155,135,063-156,194,798	AX-156275469
SGCD	rs12521447	chr5:155,135,063-156,194,798	AX-156292668
SGCD	rs10073856	chr5:155,135,063-156,194,798	AX-14941651
SGCD	rs6873333	chr5:155,135,063-156,194,798	AX-41643701
SGCD	rs201015575	chr5:155,135,063-156,194,798	AX-156298203
SGCD	rs77519690	chr5:155,135,063-156,194,798	AX-14941653
SGCD	rs72814211	chr5:155,135,063-156,194,798	AX-156275470
SGCD	rs72814211	chr5:155,135,063-156,194,798	AX-156292669
SGCD	rs2569023	chr5:155,135,063-156,194,798	AX-148258036
SGCD	rs2569023	chr5:155,135,063-156,194,798	AX-156275471
SGCD	rs62382353	chr5:155,135,063-156,194,798	AX-88321001
SGCD	rs62382353	chr5:155,135,063-156,194,798	AX-88327517
SGCD	rs79931516	chr5:155,135,063-156,194,798	AX-14941655
SGCD	rs2619714	chr5:155,135,063-156,194,798	AX-14941656
SGCD	rs2619713	chr5:155,135,063-156,194,798	AX-156275472
SGCD	rs2619713	chr5:155,135,063-156,194,798	AX-156292670
SGCD	rs2619712	chr5:155,135,063-156,194,798	AX-35168263
SGCD	---	chr5:155,135,063-156,194,798	AX-153348396
SGCD	---	chr5:155,135,063-156,194,798	AX-156298569
SGCD	rs2619710	chr5:155,135,063-156,194,798	AX-105155698
SGCD	rs2619710	chr5:155,135,063-156,194,798	AX-156275474
SGCD	rs115776377	chr5:155,135,063-156,194,798	AX-38253917
SGCD	rs6860927	chr5:155,135,063-156,194,798	AX-156275475
SGCD	rs6860927	chr5:155,135,063-156,194,798	AX-97249200
SGCD	rs62382356	chr5:155,135,063-156,194,798	AX-35168279
SGCD	rs114477925	chr5:155,135,063-156,194,798	AX-14941661
SGCD	rs11742133	chr5:155,135,063-156,194,798	AX-50448229
SGCD	rs1897554	chr5:155,135,063-156,194,798	AX-14941662
SGCD	rs77447671	chr5:155,135,063-156,194,798	AX-14941664
SGCD	rs2569024	chr5:155,135,063-156,194,798	AX-148373020
SGCD	rs2569024	chr5:155,135,063-156,194,798	AX-156282269
SGCD	rs200038727	chr5:155,135,063-156,194,798	AX-153343038
SGCD	rs200038727	chr5:155,135,063-156,194,798	AX-156298539
SGCD	rs10076006	chr5:155,135,063-156,194,798	AX-14941668
SGCD	rs2569025	chr5:155,135,063-156,194,798	AX-14941669
SGCD	rs1019928	chr5:155,135,063-156,194,798	AX-14941671

SGCD	rs41472848	chr5:155,135,063-156,194,798	AX-14941676
SGCD	rs62382359	chr5:155,135,063-156,194,798	AX-14941677
SGCD	rs2619728	chr5:155,135,063-156,194,798	AX-11401741
SGCD	rs116591184	chr5:155,135,063-156,194,798	AX-14941680
SGCD	rs2248389	chr5:155,135,063-156,194,798	AX-11378527
SGCD	rs7706170	chr5:155,135,063-156,194,798	AX-35168297
SGCD	rs76415033	chr5:155,135,063-156,194,798	AX-14941682
SGCD	rs7736695	chr5:155,135,063-156,194,798	AX-14941686
SGCD	rs1432812	chr5:155,135,063-156,194,798	AX-14941687
SGCD	rs6895133	chr5:155,135,063-156,194,798	AX-11592720
SGCD	rs2619727	chr5:155,135,063-156,194,798	AX-14941690
SGCD	rs3846690	chr5:155,135,063-156,194,798	AX-14941692
SGCD	rs12518602	chr5:155,135,063-156,194,798	AX-14941693
SGCD	rs5872439	chr5:155,135,063-156,194,798	AX-121820464
SGCD	---	chr5:155,135,063-156,194,798	AX-148664468
SGCD	---	chr5:155,135,063-156,194,798	AX-156284052
SGCD	rs75017687	chr5:155,135,063-156,194,798	AX-35168307
SGCD	rs59458149	chr5:155,135,063-156,194,798	AX-35168309
SGCD	rs200244865	chr5:155,135,063-156,194,798	AX-153258528
SGCD	rs200244865	chr5:155,135,063-156,194,798	AX-156286431
SGCD	rs71285050	chr5:155,135,063-156,194,798	AX-14941697
SGCD	rs71285050	chr5:155,135,063-156,194,798	AX-156285193
SGCD	rs2061908	chr5:155,135,063-156,194,798	AX-14941698
SGCD	rs28424124	chr5:155,135,063-156,194,798	AX-35168311
SGCD	rs28592780	chr5:155,135,063-156,194,798	AX-156275476
SGCD	rs28592780	chr5:155,135,063-156,194,798	AX-156292672
SGCD	rs7447535	chr5:155,135,063-156,194,798	AX-14941701
SGCD	rs6556773	chr5:155,135,063-156,194,798	AX-41643711
SGCD	rs2569027	chr5:155,135,063-156,194,798	AX-41643715
SGCD	rs62383761	chr5:155,135,063-156,194,798	AX-14941707
SGCD	rs2569026	chr5:155,135,063-156,194,798	AX-156282270
SGCD	rs2569026	chr5:155,135,063-156,194,798	AX-156296927
SGCD	rs2619719	chr5:155,135,063-156,194,798	AX-50448232
SGCD	rs2619716	chr5:155,135,063-156,194,798	AX-14941713
SGCD	rs77453483	chr5:155,135,063-156,194,798	AX-14941714
SGCD	rs11738333	chr5:155,135,063-156,194,798	AX-11171692
SGCD	rs974604	chr5:155,135,063-156,194,798	AX-107830826
SGCD	rs974604	chr5:155,135,063-156,194,798	AX-156275477
SGCD	rs6556779	chr5:155,135,063-156,194,798	AX-14941718
SGCD	rs11738514	chr5:155,135,063-156,194,798	AX-14941719
SGCD	rs61562015	chr5:155,135,063-156,194,798	AX-51103596
SGCD	rs13357771	chr5:155,135,063-156,194,798	AX-11251129
SGCD	rs2871580	chr5:155,135,063-156,194,798	AX-14941723
SGCD	rs79646394	chr5:155,135,063-156,194,798	AX-14941725
SGCD	rs112389596	chr5:155,135,063-156,194,798	AX-14941726
SGCD	rs73299488	chr5:155,135,063-156,194,798	AX-156275478
SGCD	rs73299488	chr5:155,135,063-156,194,798	AX-156292673
SGCD	rs75445692	chr5:155,135,063-156,194,798	AX-156275479
SGCD	rs75445692	chr5:155,135,063-156,194,798	AX-156292674
SGCD	rs58739548	chr5:155,135,063-156,194,798	AX-156275480
SGCD	rs58739548	chr5:155,135,063-156,194,798	AX-156292675
SGCD	rs72814239	chr5:155,135,063-156,194,798	AX-148389301
SGCD	rs72814239	chr5:155,135,063-156,194,798	AX-156275481
SGCD	rs1594658	chr5:155,135,063-156,194,798	AX-14941734
SGCD	rs11135369	chr5:155,135,063-156,194,798	AX-12408209

SGCD	rs76005690	chr5:155,135,063-156,194,798	AX-14941740
SGCD	rs114955282	chr5:155,135,063-156,194,798	AX-148654490
SGCD	rs114955282	chr5:155,135,063-156,194,798	AX-92656440
SGCD	rs11745732	chr5:155,135,063-156,194,798	AX-123014279
SGCD	rs11745732	chr5:155,135,063-156,194,798	AX-151269802
SGCD	rs7378774	chr5:155,135,063-156,194,798	AX-11623499
SGCD	rs13186927	chr5:155,135,063-156,194,798	AX-114298487
SGCD	rs13186927	chr5:155,135,063-156,194,798	AX-156282271
SGCD	rs76817418	chr5:155,135,063-156,194,798	AX-38253923
SGCD	rs80255077	chr5:155,135,063-156,194,798	AX-14941749
SGCD	rs73810324	chr5:155,135,063-156,194,798	AX-14941750
SGCD	rs114019818	chr5:155,135,063-156,194,798	AX-14941751
SGCD	rs244964	chr5:155,135,063-156,194,798	AX-11392722
SGCD	rs34557688	chr5:155,135,063-156,194,798	AX-107844361
SGCD	rs4444953	chr5:155,135,063-156,194,798	AX-156275482
SGCD	rs4444953	chr5:155,135,063-156,194,798	AX-156292676
SGCD	rs7726529	chr5:155,135,063-156,194,798	AX-11641420
SGCD	rs244963	chr5:155,135,063-156,194,798	AX-14941758
SGCD	rs73299493	chr5:155,135,063-156,194,798	AX-14941761
SGCD	rs165952	chr5:155,135,063-156,194,798	AX-112918852
SGCD	rs165952	chr5:155,135,063-156,194,798	AX-113475548
SGCD	rs244962	chr5:155,135,063-156,194,798	AX-14941762
SGCD	rs13436254	chr5:155,135,063-156,194,798	AX-41643731
SGCD	rs73299497	chr5:155,135,063-156,194,798	AX-14941763
SGCD	rs10056344	chr5:155,135,063-156,194,798	AX-148735220
SGCD	rs10056344	chr5:155,135,063-156,194,798	AX-156275483
SGCD	rs10065329	chr5:155,135,063-156,194,798	AX-148734828
SGCD	rs10065329	chr5:155,135,063-156,194,798	AX-156275484
SGCD	rs78995376	chr5:155,135,063-156,194,798	AX-35168345
SGCD	rs10069258	chr5:155,135,063-156,194,798	AX-12380333
SGCD	rs244961	chr5:155,135,063-156,194,798	AX-11392719
SGCD	rs10058373	chr5:155,135,063-156,194,798	AX-14941766
SGCD	rs244994	chr5:155,135,063-156,194,798	AX-35168349
SGCD	rs1368326	chr5:155,135,063-156,194,798	AX-156275485
SGCD	rs112514185	chr5:155,135,063-156,194,798	AX-38253927
SGCD	rs244992	chr5:155,135,063-156,194,798	AX-50448237
SGCD	rs17617120	chr5:155,135,063-156,194,798	AX-14941771
SGCD	rs165953	chr5:155,135,063-156,194,798	AX-14941774
SGCD	rs78541968	chr5:155,135,063-156,194,798	AX-14941775
SGCD	rs200008053	chr5:155,135,063-156,194,798	AX-156285204
SGCD	rs75894122	chr5:155,135,063-156,194,798	AX-14941778
SGCD	rs1432723	chr5:155,135,063-156,194,798	AX-12463191
SGCD	rs67625007	chr5:155,135,063-156,194,798	AX-14941782
SGCD	rs6866337	chr5:155,135,063-156,194,798	AX-96749277
SGCD	rs4526088	chr5:155,135,063-156,194,798	AX-156282272
SGCD	rs4526088	chr5:155,135,063-156,194,798	AX-156296928
SGCD	rs6874597	chr5:155,135,063-156,194,798	AX-41643739
SGCD	rs244991	chr5:155,135,063-156,194,798	AX-14941787
SGCD	rs244990	chr5:155,135,063-156,194,798	AX-156275487
SGCD	rs1432721	chr5:155,135,063-156,194,798	AX-11263058
SGCD	rs244989	chr5:155,135,063-156,194,798	AX-14941792
SGCD	rs244988	chr5:155,135,063-156,194,798	AX-14941793
SGCD	rs5016818	chr5:155,135,063-156,194,798	AX-11541377
SGCD	rs74545702	chr5:155,135,063-156,194,798	AX-14941796
SGCD	rs4704822	chr5:155,135,063-156,194,798	AX-156275488

SGCD	rs4704822	chr5:155,135,063-156,194,798	AX-156292679
SGCD	rs34028424	chr5:155,135,063-156,194,798	AX-153245929
SGCD	rs34028424	chr5:155,135,063-156,194,798	AX-156284182
SGCD	rs4502799	chr5:155,135,063-156,194,798	AX-156275489
SGCD	rs4502799	chr5:155,135,063-156,194,798	AX-156292680
SGCD	rs244986	chr5:155,135,063-156,194,798	AX-156275490
SGCD	rs244985	chr5:155,135,063-156,194,798	AX-148070582
SGCD	rs244985	chr5:155,135,063-156,194,798	AX-156275491
SGCD	rs10475521	chr5:155,135,063-156,194,798	AX-153356931
SGCD	rs10475521	chr5:155,135,063-156,194,798	AX-156275492
SGCD	rs244984	chr5:155,135,063-156,194,798	AX-156275493
SGCD	rs244983	chr5:155,135,063-156,194,798	AX-112918853
SGCD	rs244983	chr5:155,135,063-156,194,798	AX-113475549
SGCD	rs78472029	chr5:155,135,063-156,194,798	AX-35168387
SGCD	rs244982	chr5:155,135,063-156,194,798	AX-11392726
SGCD	rs2116739	chr5:155,135,063-156,194,798	AX-12517761
SGCD	rs75309704	chr5:155,135,063-156,194,798	AX-14941804
SGCD	rs78326014	chr5:155,135,063-156,194,798	AX-14941805
SGCD	rs244980	chr5:155,135,063-156,194,798	AX-35168393
SGCD	rs679660	chr5:155,135,063-156,194,798	AX-148307011
SGCD	rs679660	chr5:155,135,063-156,194,798	AX-156268160
SGCD	rs679656	chr5:155,135,063-156,194,798	AX-121346847
SGCD	rs679656	chr5:155,135,063-156,194,798	AX-156282273
SGCD	rs244979	chr5:155,135,063-156,194,798	AX-14941806
SGCD	rs11739086	chr5:155,135,063-156,194,798	AX-11171755
SGCD	rs112157476	chr5:155,135,063-156,194,798	AX-156282274
SGCD	rs112157476	chr5:155,135,063-156,194,798	AX-156296929
SGCD	rs17533269	chr5:155,135,063-156,194,798	AX-14941813
SGCD	rs79576243	chr5:155,135,063-156,194,798	AX-14941814
SGCD	rs244978	chr5:155,135,063-156,194,798	AX-14941816
SGCD	rs79109423	chr5:155,135,063-156,194,798	AX-14941817
SGCD	rs76989382	chr5:155,135,063-156,194,798	AX-35168401
SGCD	rs7733552	chr5:155,135,063-156,194,798	AX-14941819
SGCD	rs244977	chr5:155,135,063-156,194,798	AX-14941820
SGCD	rs17617422	chr5:155,135,063-156,194,798	AX-11331475
SGCD	rs73301325	chr5:155,135,063-156,194,798	AX-35168409
SGCD	rs73301328	chr5:155,135,063-156,194,798	AX-14941823
SGCD	rs9313417	chr5:155,135,063-156,194,798	AX-41643767
SGCD	rs244976	chr5:155,135,063-156,194,798	AX-14941827
SGCD	rs74295103	chr5:155,135,063-156,194,798	AX-35168415
SGCD	rs183626	chr5:155,135,063-156,194,798	AX-11346300
SGCD	rs73810342	chr5:155,135,063-156,194,798	AX-14941839
SGCD	rs17533521	chr5:155,135,063-156,194,798	AX-14941840
SGCD	rs111337309	chr5:155,135,063-156,194,798	AX-120142057
SGCD	rs111337309	chr5:155,135,063-156,194,798	AX-156284053
SGCD	rs6897920	chr5:155,135,063-156,194,798	AX-156268161
SGCD	rs6897920	chr5:155,135,063-156,194,798	AX-156288022
SGCD	rs73810343	chr5:155,135,063-156,194,798	AX-14941843
SGCD	rs13358188	chr5:155,135,063-156,194,798	AX-11251142
SGCD	rs1157296	chr5:155,135,063-156,194,798	AX-156282275
SGCD	rs1157296	chr5:155,135,063-156,194,798	AX-156296930
SGCD	rs244972	chr5:155,135,063-156,194,798	AX-11392723
SGCD	rs73810344	chr5:155,135,063-156,194,798	AX-14941845
SGCD	rs17052958	chr5:155,135,063-156,194,798	AX-14941846
SGCD	rs114338836	chr5:155,135,063-156,194,798	AX-14941847

SGCD	rs1864956	chr5:155,135,063-156,194,798	AX-11347992
SGCD	rs72805670	chr5:155,135,063-156,194,798	AX-14941850
SGCD	rs185281529	chr5:155,135,063-156,194,798	AX-156268162
SGCD	rs185281529	chr5:155,135,063-156,194,798	AX-156288023
SGCD	rs144994629	chr5:155,135,063-156,194,798	AX-121242826
SGCD	rs144994629	chr5:155,135,063-156,194,798	AX-156285991
SGCD	rs142789681	chr5:155,135,063-156,194,798	AX-148736991
SGCD	rs142789681	chr5:155,135,063-156,194,798	AX-156284054
SGCD	rs57169797	chr5:155,135,063-156,194,798	AX-148798752
SGCD	rs34361546	chr5:155,135,063-156,194,798	AX-14941853
SGCD	rs7724912	chr5:155,135,063-156,194,798	AX-12638285
SGCD	rs17052965	chr5:155,135,063-156,194,798	AX-12487857
SGCD	rs4400109	chr5:155,135,063-156,194,798	AX-11504113
SGCD	rs61533738	chr5:155,135,063-156,194,798	AX-14941856
SGCD	rs244966	chr5:155,135,063-156,194,798	AX-14941858
SGCD	rs1432734	chr5:155,135,063-156,194,798	AX-11263059
SGCD	rs1432813	chr5:155,135,063-156,194,798	AX-12463196
SGCD	rs10515731	chr5:155,135,063-156,194,798	AX-12392985
SGCD	rs17052977	chr5:155,135,063-156,194,798	AX-41643781
SGCD	rs56356229	chr5:155,135,063-156,194,798	AX-121138418
SGCD	rs56356229	chr5:155,135,063-156,194,798	AX-156284055
SGCD	rs111327524	chr5:155,135,063-156,194,798	AX-148724082
SGCD	rs111327524	chr5:155,135,063-156,194,798	AX-148873132
SGCD	rs3097823	chr5:155,135,063-156,194,798	AX-105153806
SGCD	rs3097823	chr5:155,135,063-156,194,798	AX-148177649
SGCD	rs55711130	chr5:155,135,063-156,194,798	AX-156275494
SGCD	rs55711130	chr5:155,135,063-156,194,798	AX-156292682
SGCD	rs3097822	chr5:155,135,063-156,194,798	AX-35168433
SGCD	rs3097813	chr5:155,135,063-156,194,798	AX-11433409
SGCD	rs3097821	chr5:155,135,063-156,194,798	AX-156268163
SGCD	rs3097821	chr5:155,135,063-156,194,798	AX-156288024
SGCD	rs34689423	chr5:155,135,063-156,194,798	AX-35168435
SGCD	rs244955	chr5:155,135,063-156,194,798	AX-12529541
SGCD	rs1073677	chr5:155,135,063-156,194,798	AX-156268164
SGCD	rs1073677	chr5:155,135,063-156,194,798	AX-156288025
SGCD	rs112260325	chr5:155,135,063-156,194,798	AX-94368155
SGCD	rs1594655	chr5:155,135,063-156,194,798	AX-35168443
SGCD	rs13169785	chr5:155,135,063-156,194,798	AX-148658284
SGCD	rs13169785	chr5:155,135,063-156,194,798	AX-156268165
SGCD	rs1432732	chr5:155,135,063-156,194,798	AX-156268166
SGCD	rs1432732	chr5:155,135,063-156,194,798	AX-156288026
SGCD	rs62380664	chr5:155,135,063-156,194,798	AX-14941868
SGCD	rs244954	chr5:155,135,063-156,194,798	AX-14941869
SGCD	rs4547913	chr5:155,135,063-156,194,798	AX-14941870
SGCD	rs1368332	chr5:155,135,063-156,194,798	AX-11257027
SGCD	rs11948388	chr5:155,135,063-156,194,798	AX-156268167
SGCD	rs11948388	chr5:155,135,063-156,194,798	AX-156288027
SGCD	rs11960137	chr5:155,135,063-156,194,798	AX-41643793
SGCD	rs200393489	chr5:155,135,063-156,194,798	AX-122985259
SGCD	rs200393489	chr5:155,135,063-156,194,798	AX-156285336
SGCD	rs201548921	chr5:155,135,063-156,194,798	AX-105034234
SGCD	rs201548921	chr5:155,135,063-156,194,798	AX-122703432
SGCD	rs6555912	chr5:155,135,063-156,194,798	AX-14941878
SGCD	rs573807246	chr5:155,135,063-156,194,798	AX-153382430
SGCD	rs573807246	chr5:155,135,063-156,194,798	AX-156285477

SGCD	rs201044606	chr5:155,135,063-156,194,798	AX-156284956
SGCD	rs7728169	chr5:155,135,063-156,194,798	AX-156282276
SGCD	rs7728169	chr5:155,135,063-156,194,798	AX-156296931
SGCD	rs430625	chr5:155,135,063-156,194,798	AX-156275495
SGCD	rs430625	chr5:155,135,063-156,194,798	AX-156292683
SGCD	rs244953	chr5:155,135,063-156,194,798	AX-41643813
SGCD	rs4704858	chr5:155,135,063-156,194,798	AX-35168465
SGCD	rs17052994	chr5:155,135,063-156,194,798	AX-14941886
SGCD	rs2163746	chr5:155,135,063-156,194,798	AX-11371687
SGCD	rs2008129	chr5:155,135,063-156,194,798	AX-35168467
SGCD	rs4435847	chr5:155,135,063-156,194,798	AX-35168469
SGCD	rs181741	chr5:155,135,063-156,194,798	AX-14941888
SGCD	rs1368331	chr5:155,135,063-156,194,798	AX-14941890
SGCD	rs62380666	chr5:155,135,063-156,194,798	AX-35168475
SGCD	rs113476377	chr5:155,135,063-156,194,798	AX-94351405
SGCD	rs78365975	chr5:155,135,063-156,194,798	AX-156286352
SGCD	rs80177286	chr5:155,135,063-156,194,798	AX-14941894
SGCD	rs17053001	chr5:155,135,063-156,194,798	AX-11301472
SGCD	rs17053002	chr5:155,135,063-156,194,798	AX-14941896
SGCD	rs165951	chr5:155,135,063-156,194,798	AX-14941898
SGCD	rs244960	chr5:155,135,063-156,194,798	AX-41643823
SGCD	rs244959	chr5:155,135,063-156,194,798	AX-12529542
SGCD	rs244958	chr5:155,135,063-156,194,798	AX-11392715
SGCD	rs244957	chr5:155,135,063-156,194,798	AX-156296932
SGCD	rs244957	chr5:155,135,063-156,194,798	AX-84652510
SGCD	rs4444954	chr5:155,135,063-156,194,798	AX-148483005
SGCD	rs4444954	chr5:155,135,063-156,194,798	AX-156275496
SGCD	rs244956	chr5:155,135,063-156,194,798	AX-120496405
SGCD	rs244956	chr5:155,135,063-156,194,798	AX-148061978
SGCD	rs80080561	chr5:155,135,063-156,194,798	AX-35168487
SGCD	rs78131374	chr5:155,135,063-156,194,798	AX-156275497
SGCD	rs147666530	chr5:155,135,063-156,194,798	AX-122841182
SGCD	rs147666530	chr5:155,135,063-156,194,798	AX-156298435
SGCD	rs75287098	chr5:155,135,063-156,194,798	AX-14941919
SGCD	rs10475581	chr5:155,135,063-156,194,798	AX-14941922
SGCD	rs13178470	chr5:155,135,063-156,194,798	AX-11243075
SGCD	rs17053010	chr5:155,135,063-156,194,798	AX-35168501
SGCD	rs17053011	chr5:155,135,063-156,194,798	AX-41643835
SGCD	rs10515732	chr5:155,135,063-156,194,798	AX-14941924
SGCD	rs74444839	chr5:155,135,063-156,194,798	AX-35168509
SGCD	rs6556055	chr5:155,135,063-156,194,798	AX-14941926
SGCD	rs76123169	chr5:155,135,063-156,194,798	AX-156268168
SGCD	rs11743754	chr5:155,135,063-156,194,798	AX-35168517
SGCD	rs9313627	chr5:155,135,063-156,194,798	AX-156268169
SGCD	rs9313627	chr5:155,135,063-156,194,798	AX-156288029
SGCD	rs77841783	chr5:155,135,063-156,194,798	AX-14941930
SGCD	rs141461413	chr5:155,135,063-156,194,798	AX-122688346
SGCD	rs112739409	chr5:155,135,063-156,194,798	AX-153330554
SGCD	rs112739409	chr5:155,135,063-156,194,798	AX-156268170
SGCD	rs75217931	chr5:155,135,063-156,194,798	AX-148435831
SGCD	rs75217931	chr5:155,135,063-156,194,798	AX-156268171
SGCD	---	chr5:155,135,063-156,194,798	AX-14941931
SGCD	rs10475595	chr5:155,135,063-156,194,798	AX-156268172
SGCD	rs10475595	chr5:155,135,063-156,194,798	AX-86898949
SGCD	rs4704874	chr5:155,135,063-156,194,798	AX-113570336

SGCD	rs4704874	chr5:155,135,063-156,194,798	AX-122371675
SGCD	rs200260395	chr5:155,135,063-156,194,798	AX-105063689
SGCD	rs4704876	chr5:155,135,063-156,194,798	AX-124389988
SGCD	rs4704876	chr5:155,135,063-156,194,798	AX-124394252
SGCD	rs74322565	chr5:155,135,063-156,194,798	AX-156268173
SGCD	rs74322565	chr5:155,135,063-156,194,798	AX-156288030
SGCD	rs4704878	chr5:155,135,063-156,194,798	AX-11519453
SGCD	rs17053028	chr5:155,135,063-156,194,798	AX-41643851
SGCD	rs73810354	chr5:155,135,063-156,194,798	AX-156288031
SGCD	rs17053030	chr5:155,135,063-156,194,798	AX-14941937
SGCD	rs10053345	chr5:155,135,063-156,194,798	AX-41643853
SGCD	rs17053032	chr5:155,135,063-156,194,798	AX-14941938
SGCD	rs17053035	chr5:155,135,063-156,194,798	AX-11301474
SGCD	rs34147443	chr5:155,135,063-156,194,798	AX-35168525
SGCD	rs4704880	chr5:155,135,063-156,194,798	AX-12578510
SGCD	rs143284601	chr5:155,135,063-156,194,798	AX-120373450
SGCD	rs78830942	chr5:155,135,063-156,194,798	AX-14941940
SGCD	rs17053040	chr5:155,135,063-156,194,798	AX-11301475
SGCD	rs56259673	chr5:155,135,063-156,194,798	AX-14941943
SGCD	rs137941682	chr5:155,135,063-156,194,798	AX-153324416
SGCD	rs137941682	chr5:155,135,063-156,194,798	AX-156298630
SGCD	rs1432730	chr5:155,135,063-156,194,798	AX-156292684
SGCD	rs6865706	chr5:155,135,063-156,194,798	AX-156275499
SGCD	rs6865706	chr5:155,135,063-156,194,798	AX-156292685
SGCD	rs17053050	chr5:155,135,063-156,194,798	AX-14941944
SGCD	rs6894182	chr5:155,135,063-156,194,798	AX-156292686
SGCD	rs6894361	chr5:155,135,063-156,194,798	AX-14941946
SGCD	rs6870438	chr5:155,135,063-156,194,798	AX-12612613
SGCD	rs6894695	chr5:155,135,063-156,194,798	AX-12613457
SGCD	rs73810356	chr5:155,135,063-156,194,798	AX-14941949
SGCD	rs73810357	chr5:155,135,063-156,194,798	AX-14941951
SGCD	rs73301362	chr5:155,135,063-156,194,798	AX-35168533
SGCD	rs78267523	chr5:155,135,063-156,194,798	AX-35168535
SGCD	rs6556104	chr5:155,135,063-156,194,798	AX-11571727
SGCD	---	chr5:155,135,063-156,194,798	AX-11641740
SGCD	rs201352882	chr5:155,135,063-156,194,798	AX-156285552
SGCD	rs1368327	chr5:155,135,063-156,194,798	AX-14941957
SGCD	rs1368328	chr5:155,135,063-156,194,798	AX-12460034
SGCD	rs1368329	chr5:155,135,063-156,194,798	AX-11257026
SGCD	rs1368330	chr5:155,135,063-156,194,798	AX-156292687
SGCD	rs7711809	chr5:155,135,063-156,194,798	AX-156275502
SGCD	rs7711809	chr5:155,135,063-156,194,798	AX-156292688
SGCD	rs62380670	chr5:155,135,063-156,194,798	AX-156275503
SGCD	rs7716740	chr5:155,135,063-156,194,798	AX-148300048
SGCD	rs7716740	chr5:155,135,063-156,194,798	AX-156275504
SGCD	rs11948724	chr5:155,135,063-156,194,798	AX-148074361
SGCD	rs11948724	chr5:155,135,063-156,194,798	AX-156275505
SGCD	rs11950822	chr5:155,135,063-156,194,798	AX-156292690
SGCD	rs7727854	chr5:155,135,063-156,194,798	AX-156282277
SGCD	rs4704884	chr5:155,135,063-156,194,798	AX-35168557
SGCD	rs78679054	chr5:155,135,063-156,194,798	AX-14941968
SGCD	rs6556145	chr5:155,135,063-156,194,798	AX-14941970
SGCD	rs111786881	chr5:155,135,063-156,194,798	AX-14941972
SGCD	rs10062650	chr5:155,135,063-156,194,798	AX-11090347
SGCD	rs58299963	chr5:155,135,063-156,194,798	AX-14941976

SGCD	rs4704888	chr5:155,135,063-156,194,798	AX-41643867
SGCD	rs28459094	chr5:155,135,063-156,194,798	AX-156275507
SGCD	rs28459094	chr5:155,135,063-156,194,798	AX-156292691
SGCD	rs28584510	chr5:155,135,063-156,194,798	AX-35168563
SGCD	rs6886366	chr5:155,135,063-156,194,798	AX-156275508
SGCD	rs10057754	chr5:155,135,063-156,194,798	AX-12379954
SGCD	rs200474037	chr5:155,135,063-156,194,798	AX-156298608
SGCD	rs34089031	chr5:155,135,063-156,194,798	AX-156298051
SGCD	rs200930271	chr5:155,135,063-156,194,798	AX-153244179
SGCD	rs200930271	chr5:155,135,063-156,194,798	AX-156297993
SGCD	rs4704889	chr5:155,135,063-156,194,798	AX-14941981
SGCD	rs4704890	chr5:155,135,063-156,194,798	AX-41643871
SGCD	rs891912	chr5:155,135,063-156,194,798	AX-35168567
SGCD	rs891911	chr5:155,135,063-156,194,798	AX-156275509
SGCD	rs891911	chr5:155,135,063-156,194,798	AX-156292693
SGCD	rs737307	chr5:155,135,063-156,194,798	AX-156292694
SGCD	rs737306	chr5:155,135,063-156,194,798	AX-156275511
SGCD	rs737306	chr5:155,135,063-156,194,798	AX-156292695
SGCD	rs4704748	chr5:155,135,063-156,194,798	AX-35168573
SGCD	rs4704749	chr5:155,135,063-156,194,798	AX-14941985
SGCD	rs57370050	chr5:155,135,063-156,194,798	AX-14941986
SGCD	rs113449211	chr5:155,135,063-156,194,798	AX-14941991
SGCD	rs13187962	chr5:155,135,063-156,194,798	AX-11243529
SGCD	rs56708228	chr5:155,135,063-156,194,798	AX-107834479
SGCD	rs56708228	chr5:155,135,063-156,194,798	AX-156292696
SGCD	rs72805699	chr5:155,135,063-156,194,798	AX-35168581
SGCD	rs1991792	chr5:155,135,063-156,194,798	AX-41643877
SGCD	rs17053068	chr5:155,135,063-156,194,798	AX-14941996
SGCD	rs2033455	chr5:155,135,063-156,194,798	AX-41643879
SGCD	rs10515733	chr5:155,135,063-156,194,798	AX-14942001
SGCD	rs1432733	chr5:155,135,063-156,194,798	AX-14942005
SGCD	rs11959572	chr5:155,135,063-156,194,798	AX-41643881
SGCD	rs6556227	chr5:155,135,063-156,194,798	AX-41643883
SGCD	rs6556228	chr5:155,135,063-156,194,798	AX-41643885
SGCD	rs7735601	chr5:155,135,063-156,194,798	AX-41643887
SGCD	rs1816399	chr5:155,135,063-156,194,798	AX-156275512
SGCD	rs73301391	chr5:155,135,063-156,194,798	AX-35168593
SGCD	rs13174879	chr5:155,135,063-156,194,798	AX-14942015
SGCD	rs17053082	chr5:155,135,063-156,194,798	AX-11301481
SGCD	rs6888324	chr5:155,135,063-156,194,798	AX-14942019
SGCD	rs1036185	chr5:155,135,063-156,194,798	AX-11104920
SGCD	rs10061798	chr5:155,135,063-156,194,798	AX-156275513
SGCD	rs10061798	chr5:155,135,063-156,194,798	AX-156292698
SGCD	rs10066451	chr5:155,135,063-156,194,798	AX-147795643
SGCD	rs10066451	chr5:155,135,063-156,194,798	AX-147889525
SGCD	rs61424805	chr5:155,135,063-156,194,798	AX-156275514
SGCD	rs61424805	chr5:155,135,063-156,194,798	AX-156292699
SGCD	rs1432726	chr5:155,135,063-156,194,798	AX-14942027
SGCD	rs146618637	chr5:155,135,063-156,194,798	AX-121247768
SGCD	rs146618637	chr5:155,135,063-156,194,798	AX-38253961
SGCD	rs4704899	chr5:155,135,063-156,194,798	AX-14942029
SGCD	rs75588262	chr5:155,135,063-156,194,798	AX-14942030
SGCD	rs11743064	chr5:155,135,063-156,194,798	AX-14942034
SGCD	rs11746342	chr5:155,135,063-156,194,798	AX-14942035
SGCD	rs73301393	chr5:155,135,063-156,194,798	AX-14942037

SGCD	rs11135000	chr5:155,135,063-156,194,798	AX-14942039
SGCD	rs2310879	chr5:155,135,063-156,194,798	AX-11384275
SGCD	rs7736718	chr5:155,135,063-156,194,798	AX-41643905
SGCD	rs1368333	chr5:155,135,063-156,194,798	AX-41643907
SGCD	rs56167566	chr5:155,135,063-156,194,798	AX-14942042
SGCD	rs13179859	chr5:155,135,063-156,194,798	AX-14942043
SGCD	rs200311798	chr5:155,135,063-156,194,798	AX-121876463
SGCD	rs35087560	chr5:155,135,063-156,194,798	AX-148080668
SGCD	rs35087560	chr5:155,135,063-156,194,798	AX-156275515
SGCD	rs112222682	chr5:155,135,063-156,194,798	AX-115158621
SGCD	rs112222682	chr5:155,135,063-156,194,798	AX-156275516
SGCD	rs34532809	chr5:155,135,063-156,194,798	AX-11448556
SGCD	rs73810381	chr5:155,135,063-156,194,798	AX-14942051
SGCD	rs144085692	chr5:155,135,063-156,194,798	AX-148722366
SGCD	rs144085692	chr5:155,135,063-156,194,798	AX-156284056
SGCD	rs4512121	chr5:155,135,063-156,194,798	AX-148159430
SGCD	rs4551052	chr5:155,135,063-156,194,798	AX-148096460
SGCD	rs958444	chr5:155,135,063-156,194,798	AX-41643911
SGCD	rs112900707	chr5:155,135,063-156,194,798	AX-156278660
SGCD	rs73810382	chr5:155,135,063-156,194,798	AX-14942053
SGCD	rs2033456	chr5:155,135,063-156,194,798	AX-11361549
SGCD	rs4704906	chr5:155,135,063-156,194,798	AX-41643913
SGCD	rs71582126	chr5:155,135,063-156,194,798	AX-149961976
SGCD	rs71582126	chr5:155,135,063-156,194,798	AX-92766389
SGCD	rs66754916	chr5:155,135,063-156,194,798	AX-148363792
SGCD	rs66754916	chr5:155,135,063-156,194,798	AX-156284057
SGCD	rs142148901	chr5:155,135,063-156,194,798	AX-153390249
SGCD	rs1123776	chr5:155,135,063-156,194,798	AX-35168629
SGCD	rs17053091	chr5:155,135,063-156,194,798	AX-12487865
SGCD	rs140489188	chr5:155,135,063-156,194,798	AX-153280625
SGCD	rs140489188	chr5:155,135,063-156,194,798	AX-156298484
SGCD	rs201998467	chr5:155,135,063-156,194,798	AX-153281055
SGCD	rs13163399	chr5:155,135,063-156,194,798	AX-14942060
SGCD	rs58134933	chr5:155,135,063-156,194,798	AX-14942061
SGCD	rs7726727	chr5:155,135,063-156,194,798	AX-41643917
SGCD	rs35176390	chr5:155,135,063-156,194,798	AX-14942063
SGCD	rs17053098	chr5:155,135,063-156,194,798	AX-41643921
SGCD	rs4704908	chr5:155,135,063-156,194,798	AX-156275518
SGCD	rs4704763	chr5:155,135,063-156,194,798	AX-153260440
SGCD	rs4704763	chr5:155,135,063-156,194,798	AX-156284058
SGCD	rs17053102	chr5:155,135,063-156,194,798	AX-12487866
SGCD	rs56267022	chr5:155,135,063-156,194,798	AX-14942070
SGCD	rs1019921	chr5:155,135,063-156,194,798	AX-14942071
SGCD	rs729518	chr5:155,135,063-156,194,798	AX-14942072
SGCD	rs56184653	chr5:155,135,063-156,194,798	AX-14942073
SGCD	rs35922649	chr5:155,135,063-156,194,798	AX-35168641
SGCD	rs17053110	chr5:155,135,063-156,194,798	AX-11301485
SGCD	rs10866706	chr5:155,135,063-156,194,798	AX-14942077
SGCD	rs4704766	chr5:155,135,063-156,194,798	AX-156292701
SGCD	rs56255014	chr5:155,135,063-156,194,798	AX-156282280
SGCD	rs56255014	chr5:155,135,063-156,194,798	AX-156296936
SGCD	rs13171665	chr5:155,135,063-156,194,798	AX-156296937
SGCD	rs79375969	chr5:155,135,063-156,194,798	AX-14942081
SGCD	rs61217176	chr5:155,135,063-156,194,798	AX-35168653
SGCD	rs57123572	chr5:155,135,063-156,194,798	AX-14942085

SGCD	rs58060178	chr5:155,135,063-156,194,798	AX-35168659
SGCD	rs75072411	chr5:155,135,063-156,194,798	AX-35168661
SGCD	rs12519159	chr5:155,135,063-156,194,798	AX-35168663
SGCD	rs6873515	chr5:155,135,063-156,194,798	AX-122148884
SGCD	rs6873515	chr5:155,135,063-156,194,798	AX-156268175
SGCD	rs6866134	chr5:155,135,063-156,194,798	AX-14942087
SGCD	rs1432724	chr5:155,135,063-156,194,798	AX-14942089
SGCD	rs1432725	chr5:155,135,063-156,194,798	AX-14942090
SGCD	rs13176599	chr5:155,135,063-156,194,798	AX-14942092
SGCD	rs58735899	chr5:155,135,063-156,194,798	AX-14942093
SGCD	rs34424222	chr5:155,135,063-156,194,798	AX-14942094
SGCD	rs12517364	chr5:155,135,063-156,194,798	AX-14942096
SGCD	rs1864943	chr5:155,135,063-156,194,798	AX-11347988
SGCD	rs6886073	chr5:155,135,063-156,194,798	AX-14942099
SGCD	rs10059037	chr5:155,135,063-156,194,798	AX-149208323
SGCD	rs137898073	chr5:155,135,063-156,194,798	AX-153348428
SGCD	rs137898073	chr5:155,135,063-156,194,798	AX-156284824
SGCD	rs35060941	chr5:155,135,063-156,194,798	AX-153287046
SGCD	rs35060941	chr5:155,135,063-156,194,798	AX-156268176
SGCD	rs201390010	chr5:155,135,063-156,194,798	AX-156285309
SGCD	rs10064121	chr5:155,135,063-156,194,798	AX-98016436
SGCD	rs57807656	chr5:155,135,063-156,194,798	AX-119470679
SGCD	rs13175377	chr5:155,135,063-156,194,798	AX-156296938
SGCD	rs10059344	chr5:155,135,063-156,194,798	AX-14942101
SGCD	rs10061155	chr5:155,135,063-156,194,798	AX-12380068
SGCD	rs35430862	chr5:155,135,063-156,194,798	AX-14942103
SGCD	rs10040967	chr5:155,135,063-156,194,798	AX-11089017
SGCD	rs10063307	chr5:155,135,063-156,194,798	AX-156268177
SGCD	rs9313767	chr5:155,135,063-156,194,798	AX-14942110
SGCD	rs9313768	chr5:155,135,063-156,194,798	AX-12656402
SGCD	rs9313769	chr5:155,135,063-156,194,798	AX-156268178
SGCD	rs9313769	chr5:155,135,063-156,194,798	AX-156288033
SGCD	rs10068623	chr5:155,135,063-156,194,798	AX-14942116
SGCD	---	chr5:155,135,063-156,194,798	AX-153335648
SGCD	rs10041446	chr5:155,135,063-156,194,798	AX-41643945
SGCD	rs6873111	chr5:155,135,063-156,194,798	AX-156275521
SGCD	rs6873111	chr5:155,135,063-156,194,798	AX-156292702
SGCD	rs200807025	chr5:155,135,063-156,194,798	AX-153357656
SGCD	rs200807025	chr5:155,135,063-156,194,798	AX-156298286
SGCD	rs10042990	chr5:155,135,063-156,194,798	AX-41643947
SGCD	rs12520821	chr5:155,135,063-156,194,798	AX-11212048
SGCD	rs6892854	chr5:155,135,063-156,194,798	AX-41643949
SGCD	rs6860532	chr5:155,135,063-156,194,798	AX-14942129
SGCD	rs28639474	chr5:155,135,063-156,194,798	AX-35168683
SGCD	rs10065181	chr5:155,135,063-156,194,798	AX-156275523
SGCD	rs10065181	chr5:155,135,063-156,194,798	AX-156292704
SGCD	rs1465687	chr5:155,135,063-156,194,798	AX-14942132
SGCD	rs4565195	chr5:155,135,063-156,194,798	AX-92607130
SGCD	rs4565195	chr5:155,135,063-156,194,798	AX-92665039
SGCD	rs17053124	chr5:155,135,063-156,194,798	AX-35168689
SGCD	rs4634337	chr5:155,135,063-156,194,798	AX-14942141
SGCD	rs17053126	chr5:155,135,063-156,194,798	AX-41643953
SGCD	rs4704918	chr5:155,135,063-156,194,798	AX-14942145
SGCD	rs73810389	chr5:155,135,063-156,194,798	AX-156268179
SGCD	rs73810389	chr5:155,135,063-156,194,798	AX-156288034

SGCD	rs73810390	chr5:155,135,063-156,194,798	AX-156268180
SGCD	rs73810390	chr5:155,135,063-156,194,798	AX-156288035
SGCD	rs73810391	chr5:155,135,063-156,194,798	AX-156268181
SGCD	rs73810391	chr5:155,135,063-156,194,798	AX-156288036
SGCD	rs73810392	chr5:155,135,063-156,194,798	AX-14942151
SGCD	rs7717273	chr5:155,135,063-156,194,798	AX-14942152
SGCD	rs7736205	chr5:155,135,063-156,194,798	AX-12638692
SGCD	rs6887369	chr5:155,135,063-156,194,798	AX-153323726
SGCD	rs6887369	chr5:155,135,063-156,194,798	AX-88323200
SGCD	rs17053134	chr5:155,135,063-156,194,798	AX-11301489
SGCD	rs10454973	chr5:155,135,063-156,194,798	AX-41643955
SGCD	rs75993682	chr5:155,135,063-156,194,798	AX-14942162
SGCD	rs73810394	chr5:155,135,063-156,194,798	AX-156282283
SGCD	rs73810394	chr5:155,135,063-156,194,798	AX-156296939
SGCD	rs79175999	chr5:155,135,063-156,194,798	AX-148561104
SGCD	rs79175999	chr5:155,135,063-156,194,798	AX-156282284
SGCD	rs200508172	chr5:155,135,063-156,194,798	AX-153333539
SGCD	rs200508172	chr5:155,135,063-156,194,798	AX-156286305
SGCD	rs11750885	chr5:155,135,063-156,194,798	AX-11172702
SGCD	rs12521203	chr5:155,135,063-156,194,798	AX-156268182
SGCD	rs12521203	chr5:155,135,063-156,194,798	AX-156288037
SGCD	rs4704921	chr5:155,135,063-156,194,798	AX-41643959
SGCD	rs73810395	chr5:155,135,063-156,194,798	AX-156268183
SGCD	rs73810395	chr5:155,135,063-156,194,798	AX-156288038
SGCD	rs73810396	chr5:155,135,063-156,194,798	AX-35168709
SGCD	rs73810397	chr5:155,135,063-156,194,798	AX-14942170
SGCD	rs10454961	chr5:155,135,063-156,194,798	AX-14942172
SGCD	rs112455027	chr5:155,135,063-156,194,798	AX-156284059
SGCD	rs57194836	chr5:155,135,063-156,194,798	AX-148588674
SGCD	rs57194836	chr5:155,135,063-156,194,798	AX-156268184
SGCD	rs17536262	chr5:155,135,063-156,194,798	AX-41643963
SGCD	rs4704923	chr5:155,135,063-156,194,798	AX-41643965
SGCD	rs76128112	chr5:155,135,063-156,194,798	AX-35168719
SGCD	rs73810399	chr5:155,135,063-156,194,798	AX-14942179
SGCD	rs35598606	chr5:155,135,063-156,194,798	AX-156286313
SGCD	rs553748113	chr5:155,135,063-156,194,798	AX-121171048
SGCD	rs553748113	chr5:155,135,063-156,194,798	AX-156285270
SGCD	rs2053041	chr5:155,135,063-156,194,798	AX-14942185
SGCD	rs17053140	chr5:155,135,063-156,194,798	AX-156275524
SGCD	rs17053140	chr5:155,135,063-156,194,798	AX-156292705
SGCD	rs256635	chr5:155,135,063-156,194,798	AX-12532084
SGCD	rs73302947	chr5:155,135,063-156,194,798	AX-121625398
SGCD	rs73302947	chr5:155,135,063-156,194,798	AX-156268185
SGCD	rs73812911	chr5:155,135,063-156,194,798	AX-156282285
SGCD	rs73812911	chr5:155,135,063-156,194,798	AX-156296940
SGCD	rs17536506	chr5:155,135,063-156,194,798	AX-35168729
SGCD	rs2163745	chr5:155,135,063-156,194,798	AX-35168731
SGCD	rs5872451	chr5:155,135,063-156,194,798	AX-153234175
SGCD	rs5872451	chr5:155,135,063-156,194,798	AX-156298109
SGCD	rs2116737	chr5:155,135,063-156,194,798	AX-11368325
SGCD	rs2436421	chr5:155,135,063-156,194,798	AX-11392021
SGCD	rs2436420	chr5:155,135,063-156,194,798	AX-14942195
SGCD	rs5872452	chr5:155,135,063-156,194,798	AX-107799226
SGCD	rs5872452	chr5:155,135,063-156,194,798	AX-156297698
SGCD	rs1432727	chr5:155,135,063-156,194,798	AX-14942199

SGCD	rs256634	chr5:155,135,063-156,194,798	AX-14942200
SGCD	rs256633	chr5:155,135,063-156,194,798	AX-35168735
SGCD	rs10037831	chr5:155,135,063-156,194,798	AX-35168737
SGCD	rs256632	chr5:155,135,063-156,194,798	AX-148035227
SGCD	rs256632	chr5:155,135,063-156,194,798	AX-96622694
SGCD	rs17536707	chr5:155,135,063-156,194,798	AX-11328097
SGCD	rs70981995	chr5:155,135,063-156,194,798	AX-153375030
SGCD	rs70981995	chr5:155,135,063-156,194,798	AX-156297955
SGCD	rs73812913	chr5:155,135,063-156,194,798	AX-14942206
SGCD	rs3097811	chr5:155,135,063-156,194,798	AX-14942208
SGCD	rs3097811	chr5:155,135,063-156,194,798	AX-156292706
SGCD	rs7721978	chr5:155,135,063-156,194,798	AX-14942209
SGCD	rs7707208	chr5:155,135,063-156,194,798	AX-14942210
SGCD	rs256631	chr5:155,135,063-156,194,798	AX-11398771
SGCD	rs256630	chr5:155,135,063-156,194,798	AX-35168739
SGCD	rs7734472	chr5:155,135,063-156,194,798	AX-35168741
SGCD	rs256629	chr5:155,135,063-156,194,798	AX-14942213
SGCD	rs256628	chr5:155,135,063-156,194,798	AX-35168743
SGCD	rs10053395	chr5:155,135,063-156,194,798	AX-11089766
SGCD	rs4704934	chr5:155,135,063-156,194,798	AX-41643973
SGCD	rs256627	chr5:155,135,063-156,194,798	AX-14942215
SGCD	rs4704935	chr5:155,135,063-156,194,798	AX-14942217
SGCD	rs77495425	chr5:155,135,063-156,194,798	AX-14942219
SGCD	rs256626	chr5:155,135,063-156,194,798	AX-156275525
SGCD	rs3097812	chr5:155,135,063-156,194,798	AX-156275526
SGCD	rs3097812	chr5:155,135,063-156,194,798	AX-156292708
SGCD	rs78882754	chr5:155,135,063-156,194,798	AX-14942222
SGCD	rs256625	chr5:155,135,063-156,194,798	AX-14942223
SGCD	rs1347116	chr5:155,135,063-156,194,798	AX-156275527
SGCD	rs1347116	chr5:155,135,063-156,194,798	AX-156292709
SGCD	---	chr5:155,135,063-156,194,798	AX-124391297
SGCD	---	chr5:155,135,063-156,194,798	AX-124392138
SGCD	rs6862821	chr5:155,135,063-156,194,798	AX-12612345
SGCD	rs77818256	chr5:155,135,063-156,194,798	AX-156275528
SGCD	rs77818256	chr5:155,135,063-156,194,798	AX-156292710
SGCD	rs3097855	chr5:155,135,063-156,194,798	AX-11433416
SGCD	rs73812915	chr5:155,135,063-156,194,798	AX-14942226
SGCD	rs7724181	chr5:155,135,063-156,194,798	AX-156275529
SGCD	rs7724181	chr5:155,135,063-156,194,798	AX-156292711
SGCD	rs7701517	chr5:155,135,063-156,194,798	AX-41643981
SGCD	rs1432728	chr5:155,135,063-156,194,798	AX-156282286
SGCD	rs77079151	chr5:155,135,063-156,194,798	AX-14942227
SGCD	rs1002844	chr5:155,135,063-156,194,798	AX-156275530
SGCD	rs1002844	chr5:155,135,063-156,194,798	AX-156292712
SGCD	rs77825574	chr5:155,135,063-156,194,798	AX-156268186
SGCD	rs77825574	chr5:155,135,063-156,194,798	AX-156288039
SGCD	rs17537018	chr5:155,135,063-156,194,798	AX-14942230
SGCD	rs74742493	chr5:155,135,063-156,194,798	AX-35168763
SGCD	rs113793267	chr5:155,135,063-156,194,798	AX-156284060
SGCD	rs10065929	chr5:155,135,063-156,194,798	AX-148200314
SGCD	rs10065929	chr5:155,135,063-156,194,798	AX-92605374
SGCD	rs112468444	chr5:155,135,063-156,194,798	AX-90039556
SGCD	rs111632521	chr5:155,135,063-156,194,798	AX-14942235
SGCD	rs10475659	chr5:155,135,063-156,194,798	AX-156268187
SGCD	rs12188985	chr5:155,135,063-156,194,798	AX-147826306

SGCD	rs12188985	chr5:155,135,063-156,194,798	AX-147920302
SGCD	rs61191718	chr5:155,135,063-156,194,798	AX-148259179
SGCD	rs61191718	chr5:155,135,063-156,194,798	AX-156268188
SGCD	rs116560833	chr5:155,135,063-156,194,798	AX-153174190
SGCD	rs116560833	chr5:155,135,063-156,194,798	AX-156288040
SGCD	rs2116738	chr5:155,135,063-156,194,798	AX-12517760
SGCD	rs1002843	chr5:155,135,063-156,194,798	AX-156268189
SGCD	rs1002843	chr5:155,135,063-156,194,798	AX-156288041
SGCD	rs9687494	chr5:155,135,063-156,194,798	AX-14942244
SGCD	rs58209951	chr5:155,135,063-156,194,798	AX-14942245
SGCD	rs1347118	chr5:155,135,063-156,194,798	AX-11254943
SGCD	rs142239333	chr5:155,135,063-156,194,798	AX-94351406
SGCD	rs75174473	chr5:155,135,063-156,194,798	AX-14942246
SGCD	rs992563	chr5:155,135,063-156,194,798	AX-156268190
SGCD	rs992563	chr5:155,135,063-156,194,798	AX-156288042
SGCD	rs79681499	chr5:155,135,063-156,194,798	AX-156283253
SGCD	rs79681499	chr5:155,135,063-156,194,798	AX-156297611
SGCD	rs75842661	chr5:155,135,063-156,194,798	AX-156282287
SGCD	rs75842661	chr5:155,135,063-156,194,798	AX-156296942
SGCD	rs79707962	chr5:155,135,063-156,194,798	AX-14942248
SGCD	rs78054006	chr5:155,135,063-156,194,798	AX-35168779
SGCD	rs58327079	chr5:155,135,063-156,194,798	AX-14942249
SGCD	rs73812917	chr5:155,135,063-156,194,798	AX-35168781
SGCD	rs58982831	chr5:155,135,063-156,194,798	AX-35168789
SGCD	rs140235000	chr5:155,135,063-156,194,798	AX-107801617
SGCD	rs78866105	chr5:155,135,063-156,194,798	AX-14942258
SGCD	rs4601027	chr5:155,135,063-156,194,798	AX-14942261
SGCD	rs17537158	chr5:155,135,063-156,194,798	AX-14942263
SGCD	rs7736045	chr5:155,135,063-156,194,798	AX-14942266
SGCD	rs73812919	chr5:155,135,063-156,194,798	AX-156268191
SGCD	rs4704943	chr5:155,135,063-156,194,798	AX-11519462
SGCD	rs111618151	chr5:155,135,063-156,194,798	AX-153303921
SGCD	rs111618151	chr5:155,135,063-156,194,798	AX-156284365
SGCD	rs77005236	chr5:155,135,063-156,194,798	AX-14942269
SGCD	rs73812920	chr5:155,135,063-156,194,798	AX-14942271
SGCD	rs6556352	chr5:155,135,063-156,194,798	AX-11571748
SGCD	rs6897824	chr5:155,135,063-156,194,798	AX-156282288
SGCD	rs6897824	chr5:155,135,063-156,194,798	AX-156296943
SGCD	rs77732553	chr5:155,135,063-156,194,798	AX-14942272
SGCD	rs73812922	chr5:155,135,063-156,194,798	AX-35168801
SGCD	rs73812923	chr5:155,135,063-156,194,798	AX-35168803
SGCD	rs74871824	chr5:155,135,063-156,194,798	AX-14942274
SGCD	rs4418084	chr5:155,135,063-156,194,798	AX-14942277
SGCD	rs56347212	chr5:155,135,063-156,194,798	AX-14942278
SGCD	rs79834561	chr5:155,135,063-156,194,798	AX-14942279
SGCD	rs6556357	chr5:155,135,063-156,194,798	AX-156275531
SGCD	rs6556357	chr5:155,135,063-156,194,798	AX-156292713
SGCD	rs77691983	chr5:155,135,063-156,194,798	AX-14942280
SGCD	rs12658158	chr5:155,135,063-156,194,798	AX-156288044
SGCD	rs12658158	chr5:155,135,063-156,194,798	AX-75176545
SGCD	rs113792361	chr5:155,135,063-156,194,798	AX-156268192
SGCD	rs113792361	chr5:155,135,063-156,194,798	AX-156288045
SGCD	rs72643425	chr5:155,135,063-156,194,798	AX-148427644
SGCD	rs11949606	chr5:155,135,063-156,194,798	AX-121737800
SGCD	rs11949606	chr5:155,135,063-156,194,798	AX-148204370

SGCD	rs112211079	chr5:155,135,063-156,194,798	AX-148466823
SGCD	rs112211079	chr5:155,135,063-156,194,798	AX-96844849
SGCD	rs111762202	chr5:155,135,063-156,194,798	AX-96568231
SGCD	rs149801030	chr5:155,135,063-156,194,798	AX-119774592
SGCD	rs149801030	chr5:155,135,063-156,194,798	AX-95862812
SGCD	rs4434366	chr5:155,135,063-156,194,798	AX-151137166
SGCD	rs4434366	chr5:155,135,063-156,194,798	AX-153337087
SGCD	rs11742181	chr5:155,135,063-156,194,798	AX-35168817
SGCD	rs111875875	chr5:155,135,063-156,194,798	AX-156288046
SGCD	rs80115108	chr5:155,135,063-156,194,798	AX-14942285
SGCD	rs998999	chr5:155,135,063-156,194,798	AX-156282289
SGCD	rs998999	chr5:155,135,063-156,194,798	AX-156296944
SGCD	rs998997	chr5:155,135,063-156,194,798	AX-50448280
SGCD	rs4273589	chr5:155,135,063-156,194,798	AX-156296945
SGCD	---	chr5:155,135,063-156,194,798	AX-14942288
SGCD	rs6897778	chr5:155,135,063-156,194,798	AX-107723642
SGCD	rs6897778	chr5:155,135,063-156,194,798	AX-156268195
SGCD	rs6874329	chr5:155,135,063-156,194,798	AX-156268196
SGCD	rs6874329	chr5:155,135,063-156,194,798	AX-156288047
SGCD	rs74699963	chr5:155,135,063-156,194,798	AX-156296946
SGCD	rs77332121	chr5:155,135,063-156,194,798	AX-156282292
SGCD	rs77332121	chr5:155,135,063-156,194,798	AX-156296947
SGCD	rs112068414	chr5:155,135,063-156,194,798	AX-148581181
SGCD	rs201283942	chr5:155,135,063-156,194,798	AX-153377574
SGCD	rs201283942	chr5:155,135,063-156,194,798	AX-156298267
SGCD	rs13356726	chr5:155,135,063-156,194,798	AX-156282293
SGCD	rs13356726	chr5:155,135,063-156,194,798	AX-156296948
SGCD	rs6890777	chr5:155,135,063-156,194,798	AX-148388488
SGCD	rs78761615	chr5:155,135,063-156,194,798	AX-14942294
SGCD	rs4478303	chr5:155,135,063-156,194,798	AX-88821090
SGCD	rs4478303	chr5:155,135,063-156,194,798	AX-88821336
SGCD	rs114926289	chr5:155,135,063-156,194,798	AX-156275532
SGCD	rs114926289	chr5:155,135,063-156,194,798	AX-156292714
SGCD	rs80281125	chr5:155,135,063-156,194,798	AX-50448281
SGCD	rs74921733	chr5:155,135,063-156,194,798	AX-14942298
SGCD	rs75991905	chr5:155,135,063-156,194,798	AX-156275533
SGCD	rs75991905	chr5:155,135,063-156,194,798	AX-156292715
SGCD	rs59005460	chr5:155,135,063-156,194,798	AX-153298366
SGCD	rs59005460	chr5:155,135,063-156,194,798	AX-156284598
SGCD	rs66652710	chr5:155,135,063-156,194,798	AX-156275534
SGCD	rs66652710	chr5:155,135,063-156,194,798	AX-156292716
SGCD	rs75332980	chr5:155,135,063-156,194,798	AX-156275535
SGCD	rs75332980	chr5:155,135,063-156,194,798	AX-156292717
SGCD	rs76668083	chr5:155,135,063-156,194,798	AX-50448282
SGCD	rs112774930	chr5:155,135,063-156,194,798	AX-153324140
SGCD	rs112774930	chr5:155,135,063-156,194,798	AX-156286120
SGCD	rs74809394	chr5:155,135,063-156,194,798	AX-156268197
SGCD	rs74809394	chr5:155,135,063-156,194,798	AX-156288048
SGCD	rs77498485	chr5:155,135,063-156,194,798	AX-156268198
SGCD	rs77498485	chr5:155,135,063-156,194,798	AX-156288049
SGCD	rs114572602	chr5:155,135,063-156,194,798	AX-156268199
SGCD	rs114572602	chr5:155,135,063-156,194,798	AX-156288050
SGCD	rs75776057	chr5:155,135,063-156,194,798	AX-156288051
SGCD	rs10070956	chr5:155,135,063-156,194,798	AX-11090878
SGCD	rs10069855	chr5:155,135,063-156,194,798	AX-156268201

SGCD	rs10069855	chr5:155,135,063-156,194,798	AX-156288052
SGCD	rs140165378	chr5:155,135,063-156,194,798	AX-156284061
SGCD	rs146691095	chr5:155,135,063-156,194,798	AX-148645853
SGCD	rs146691095	chr5:155,135,063-156,194,798	AX-92736143
SGCD	rs140303067	chr5:155,135,063-156,194,798	AX-156284062
SGCD	rs140303067	chr5:155,135,063-156,194,798	AX-88326101
SGCD	rs145210901	chr5:155,135,063-156,194,798	AX-148621924
SGCD	rs145210901	chr5:155,135,063-156,194,798	AX-96811242
SGCD	rs60166257	chr5:155,135,063-156,194,798	AX-156284063
SGCD	rs187138699	chr5:155,135,063-156,194,798	AX-114042561
SGCD	rs187138699	chr5:155,135,063-156,194,798	AX-121746832
SGCD	rs112059885	chr5:155,135,063-156,194,798	AX-156284064
SGCD	rs112059885	chr5:155,135,063-156,194,798	AX-96823234
SGCD	rs10476268	chr5:155,135,063-156,194,798	AX-149468466
SGCD	rs79970141	chr5:155,135,063-156,194,798	AX-148120680
SGCD	rs79970141	chr5:155,135,063-156,194,798	AX-156284065
SGCD	---	chr5:155,135,063-156,194,798	AX-156282295
SGCD	---	chr5:155,135,063-156,194,798	AX-94365404
SGCD	rs146119960	chr5:155,135,063-156,194,798	AX-119355262
SGCD	rs146119960	chr5:155,135,063-156,194,798	AX-153310863
SGCD	rs139257230	chr5:155,135,063-156,194,798	AX-11455855
SGCD	rs139257230	chr5:155,135,063-156,194,798	AX-119419366
SGCD	rs186408008	chr5:155,135,063-156,194,798	AX-114051243
SGCD	rs186408008	chr5:155,135,063-156,194,798	AX-156284066
SGCD	rs58659977	chr5:155,135,063-156,194,798	AX-153322647
SGCD	rs146853940	chr5:155,135,063-156,194,798	AX-119289711
SGCD	rs146853940	chr5:155,135,063-156,194,798	AX-92705994
SGCD	rs140270015	chr5:155,135,063-156,194,798	AX-144909465
SGCD	rs140270015	chr5:155,135,063-156,194,798	AX-148559325
SGCD	rs4704964	chr5:155,135,063-156,194,798	AX-153326725
SGCD	rs4704964	chr5:155,135,063-156,194,798	AX-156284067
SGCD	rs4704965	chr5:155,135,063-156,194,798	AX-121414485
SGCD	rs4704965	chr5:155,135,063-156,194,798	AX-153322417
SGCD	rs150098057	chr5:155,135,063-156,194,798	AX-153333225
SGCD	rs150098057	chr5:155,135,063-156,194,798	AX-156284068
SGCD	rs138634965	chr5:155,135,063-156,194,798	AX-121410828
SGCD	rs138634965	chr5:155,135,063-156,194,798	AX-156284069
SGCD	rs141464147	chr5:155,135,063-156,194,798	AX-148623299
SGCD	rs141464147	chr5:155,135,063-156,194,798	AX-96909537
SGCD	rs146975160	chr5:155,135,063-156,194,798	AX-148513739
SGCD	rs146975160	chr5:155,135,063-156,194,798	AX-148552220
SGCD	rs188019253	chr5:155,135,063-156,194,798	AX-119739691
SGCD	rs188019253	chr5:155,135,063-156,194,798	AX-151402722
SGCD	rs144731242	chr5:155,135,063-156,194,798	AX-148588129
SGCD	rs144731242	chr5:155,135,063-156,194,798	AX-156284070
SGCD	rs10476272	chr5:155,135,063-156,194,798	AX-148205233
SGCD	rs201713321	chr5:155,135,063-156,194,798	AX-153345547
SGCD	rs201713321	chr5:155,135,063-156,194,798	AX-156286346
SGCD	rs58999231	chr5:155,135,063-156,194,798	AX-153256556
SGCD	rs58999231	chr5:155,135,063-156,194,798	AX-153293386
SGCD	rs10476273	chr5:155,135,063-156,194,798	AX-119535993
SGCD	rs150622703	chr5:155,135,063-156,194,798	AX-105036709
SGCD	rs150622703	chr5:155,135,063-156,194,798	AX-156284071
SGCD	rs10476274	chr5:155,135,063-156,194,798	AX-105112423
SGCD	rs10476274	chr5:155,135,063-156,194,798	AX-156275536

SGCD	rs186521532	chr5:155,135,063-156,194,798	AX-148596262
SGCD	rs140166559	chr5:155,135,063-156,194,798	AX-148597803
SGCD	rs140166559	chr5:155,135,063-156,194,798	AX-96666029
SGCD	rs73813227	chr5:155,135,063-156,194,798	AX-148557727
SGCD	rs73813227	chr5:155,135,063-156,194,798	AX-156275537
SGCD	rs12187915	chr5:155,135,063-156,194,798	AX-11194557
SGCD	rs10476276	chr5:155,135,063-156,194,798	AX-41644037
SGCD	rs75721459	chr5:155,135,063-156,194,798	AX-14942314
SGCD	rs77171253	chr5:155,135,063-156,194,798	AX-14942315
SGCD	rs77546817	chr5:155,135,063-156,194,798	AX-14942316
SGCD	rs4704970	chr5:155,135,063-156,194,798	AX-11519465
SGCD	rs78132702	chr5:155,135,063-156,194,798	AX-14942317
SGCD	rs13157744	chr5:155,135,063-156,194,798	AX-58381248
SGCD	rs73813228	chr5:155,135,063-156,194,798	AX-156275538
SGCD	rs73813228	chr5:155,135,063-156,194,798	AX-156292718
SGCD	rs74938710	chr5:155,135,063-156,194,798	AX-153329396
SGCD	rs74938710	chr5:155,135,063-156,194,798	AX-156275539
SGCD	rs80138336	chr5:155,135,063-156,194,798	AX-156275540
SGCD	rs113472609	chr5:155,135,063-156,194,798	AX-148493126
SGCD	rs67349834	chr5:155,135,063-156,194,798	AX-156275541
SGCD	rs67349834	chr5:155,135,063-156,194,798	AX-156292719
SGCD	rs75500192	chr5:155,135,063-156,194,798	AX-35168891
SGCD	---	chr5:155,135,063-156,194,798	AX-38254005
SGCD	rs11135048	chr5:155,135,063-156,194,798	AX-41644047
SGCD	rs75213665	chr5:155,135,063-156,194,798	AX-156275542
SGCD	rs10062823	chr5:155,135,063-156,194,798	AX-156275543
SGCD	rs10062823	chr5:155,135,063-156,194,798	AX-156292721
SGCD	rs76838101	chr5:155,135,063-156,194,798	AX-14942332
SGCD	rs4582259	chr5:155,135,063-156,194,798	AX-41644051
SGCD	rs11953463	chr5:155,135,063-156,194,798	AX-41644053
SGCD	rs73813229	chr5:155,135,063-156,194,798	AX-14942333
SGCD	rs78890533	chr5:155,135,063-156,194,798	AX-14942334
SGCD	rs78906896	chr5:155,135,063-156,194,798	AX-14942336
SGCD	rs111420216	chr5:155,135,063-156,194,798	AX-148602363
SGCD	rs111420216	chr5:155,135,063-156,194,798	AX-156284073
SGCD	rs187051084	chr5:155,135,063-156,194,798	AX-153274528
SGCD	rs187051084	chr5:155,135,063-156,194,798	AX-156284074
SGCD	rs199600451	chr5:155,135,063-156,194,798	AX-142765565
SGCD	rs191383548	chr5:155,135,063-156,194,798	AX-156284075
SGCD	---	chr5:155,135,063-156,194,798	AX-153235193
SGCD	---	chr5:155,135,063-156,194,798	AX-156285320
SGCD	rs76525873	chr5:155,135,063-156,194,798	AX-156275544
SGCD	rs76525873	chr5:155,135,063-156,194,798	AX-156292722
SGCD	rs77909569	chr5:155,135,063-156,194,798	AX-148476592
SGCD	rs77909569	chr5:155,135,063-156,194,798	AX-156282296
SGCD	rs10067216	chr5:155,135,063-156,194,798	AX-156282297
SGCD	rs10067216	chr5:155,135,063-156,194,798	AX-156296949
SGCD	rs67257169	chr5:155,135,063-156,194,798	AX-14942339
SGCD	rs79631630	chr5:155,135,063-156,194,798	AX-148592772
SGCD	rs79631630	chr5:155,135,063-156,194,798	AX-156275545
SGCD	rs76666190	chr5:155,135,063-156,194,798	AX-14942340
SGCD	rs111571767	chr5:155,135,063-156,194,798	AX-153329420
SGCD	rs111571767	chr5:155,135,063-156,194,798	AX-156284076
SGCD	rs112718008	chr5:155,135,063-156,194,798	AX-14942344
SGCD	rs73298688	chr5:155,135,063-156,194,798	AX-14942348

SGCD	rs7714933	chr5:155,135,063-156,194,798	AX-11640666
SGCD	rs4466143	chr5:155,135,063-156,194,798	AX-156275546
SGCD	rs4466143	chr5:155,135,063-156,194,798	AX-156292723
SGCD	rs7716954	chr5:155,135,063-156,194,798	AX-11233746
SGCD	rs7716954	chr5:155,135,063-156,194,798	AX-148425112
SGCD	rs7702268	chr5:155,135,063-156,194,798	AX-148424445
SGCD	rs7702268	chr5:155,135,063-156,194,798	AX-156275547
SGCD	rs10040960	chr5:155,135,063-156,194,798	AX-148915317
SGCD	rs10040960	chr5:155,135,063-156,194,798	AX-156275548
SGCD	rs256776	chr5:155,135,063-156,194,798	AX-156275549
SGCD	rs256776	chr5:155,135,063-156,194,798	AX-156292724
SGCD	rs256775	chr5:155,135,063-156,194,798	AX-11398843
SGCD	rs34156495	chr5:155,135,063-156,194,798	AX-156284526
SGCD	rs34420220	chr5:155,135,063-156,194,798	AX-119780452
SGCD	rs79223307	chr5:155,135,063-156,194,798	AX-35168921
SGCD	rs57304745	chr5:155,135,063-156,194,798	AX-14942361
SGCD	rs12233931	chr5:155,135,063-156,194,798	AX-156275550
SGCD	rs12233931	chr5:155,135,063-156,194,798	AX-156292725
SGCD	rs32387	chr5:155,135,063-156,194,798	AX-156279372
SGCD	rs32387	chr5:155,135,063-156,194,798	AX-156295101
SGCD	rs32386	chr5:155,135,063-156,194,798	AX-41644061
SGCD	rs32385	chr5:155,135,063-156,194,798	AX-41644063
SGCD	rs76490600	chr5:155,135,063-156,194,798	AX-14942365
SGCD	rs78643088	chr5:155,135,063-156,194,798	AX-35168925
SGCD	rs39947	chr5:155,135,063-156,194,798	AX-41644067
SGCD	rs7701883	chr5:155,135,063-156,194,798	AX-148159511
SGCD	rs7701883	chr5:155,135,063-156,194,798	AX-156279373
SGCD	rs7707018	chr5:155,135,063-156,194,798	AX-11640155
SGCD	rs7712924	chr5:155,135,063-156,194,798	AX-14942372
SGCD	rs7713089	chr5:155,135,063-156,194,798	AX-41644069
SGCD	rs7702370	chr5:155,135,063-156,194,798	AX-14942374
SGCD	rs752058	chr5:155,135,063-156,194,798	AX-156296950
SGCD	rs891909	chr5:155,135,063-156,194,798	AX-12653934
SGCD	rs891908	chr5:155,135,063-156,194,798	AX-12653933
SGCD	rs12152972	chr5:155,135,063-156,194,798	AX-11193529
SGCD	rs1432707	chr5:155,135,063-156,194,798	AX-11263056
SGCD	rs32384	chr5:155,135,063-156,194,798	AX-14942380
SGCD	rs74864866	chr5:155,135,063-156,194,798	AX-14942381
SGCD	rs1813076	chr5:155,135,063-156,194,798	AX-96990543
SGCD	rs79869048	chr5:155,135,063-156,194,798	AX-14942383
SGCD	rs78803444	chr5:155,135,063-156,194,798	AX-38254017
SGCD	rs185370	chr5:155,135,063-156,194,798	AX-12507573
SGCD	rs148753107	chr5:155,135,063-156,194,798	AX-153323597
SGCD	rs60736426	chr5:155,135,063-156,194,798	AX-151288439
SGCD	rs60736426	chr5:155,135,063-156,194,798	AX-156284672
SGCD	rs199591425	chr5:155,135,063-156,194,798	AX-121303978
SGCD	rs199591425	chr5:155,135,063-156,194,798	AX-156284934
SGCD	rs113491664	chr5:155,135,063-156,194,798	AX-148399830
SGCD	rs113491664	chr5:155,135,063-156,194,798	AX-156284077
SGCD	rs32383	chr5:155,135,063-156,194,798	AX-156275551
SGCD	rs32383	chr5:155,135,063-156,194,798	AX-92682750
SGCD	rs1368321	chr5:155,135,063-156,194,798	AX-147821706
SGCD	rs1368321	chr5:155,135,063-156,194,798	AX-147915678
SGCD	rs3097865	chr5:155,135,063-156,194,798	AX-41644093
SGCD	rs1368320	chr5:155,135,063-156,194,798	AX-11257025

SGCD	rs17626481	chr5:155,135,063-156,194,798	AX-14942397
SGCD	rs2436328	chr5:155,135,063-156,194,798	AX-156284078
SGCD	rs55756765	chr5:155,135,063-156,194,798	AX-35168939
SGCD	rs1549887	chr5:155,135,063-156,194,798	AX-14942398
SGCD	rs3097863	chr5:155,135,063-156,194,798	AX-156292728
SGCD	rs1030659	chr5:155,135,063-156,194,798	AX-156292729
SGCD	rs1030659	chr5:155,135,063-156,194,798	AX-84662705
SGCD	rs1030658	chr5:155,135,063-156,194,798	AX-156282299
SGCD	rs1030658	chr5:155,135,063-156,194,798	AX-156296951
SGCD	rs7737257	chr5:155,135,063-156,194,798	AX-41644103
SGCD	rs10075096	chr5:155,135,063-156,194,798	AX-14942404
SGCD	rs10042085	chr5:155,135,063-156,194,798	AX-14942405
SGCD	rs6874980	chr5:155,135,063-156,194,798	AX-41644109
SGCD	rs6890997	chr5:155,135,063-156,194,798	AX-50448296
SGCD	rs6891351	chr5:155,135,063-156,194,798	AX-14942409
SGCD	rs79446952	chr5:155,135,063-156,194,798	AX-35168949
SGCD	rs145021986	chr5:155,135,063-156,194,798	AX-107766046
SGCD	rs145021986	chr5:155,135,063-156,194,798	AX-156285603
SGCD	rs144080122	chr5:155,135,063-156,194,798	AX-153306802
SGCD	rs144080122	chr5:155,135,063-156,194,798	AX-156284358
SGCD	rs1368319	chr5:155,135,063-156,194,798	AX-14942413
SGCD	rs2871724	chr5:155,135,063-156,194,798	AX-14942414
SGCD	rs76412476	chr5:155,135,063-156,194,798	AX-14942418
SGCD	rs32381	chr5:155,135,063-156,194,798	AX-41644115
SGCD	rs32380	chr5:155,135,063-156,194,798	AX-14942420
SGCD	rs41084	chr5:155,135,063-156,194,798	AX-41644119
SGCD	rs1368317	chr5:155,135,063-156,194,798	AX-41644121
SGCD	rs17538098	chr5:155,135,063-156,194,798	AX-50902267
SGCD	rs3097808	chr5:155,135,063-156,194,798	AX-14942426
SGCD	rs919728	chr5:155,135,063-156,194,798	AX-14942429
SGCD	rs32379	chr5:155,135,063-156,194,798	AX-14942436
SGCD	rs72807765	chr5:155,135,063-156,194,798	AX-14942438
SGCD	rs1368316	chr5:155,135,063-156,194,798	AX-14942442
SGCD	rs3097862	chr5:155,135,063-156,194,798	AX-156275555
SGCD	rs3097862	chr5:155,135,063-156,194,798	AX-156292730
SGCD	rs10476293	chr5:155,135,063-156,194,798	AX-14942444
SGCD	rs60577002	chr5:155,135,063-156,194,798	AX-14942446
SGCD	rs17053240	chr5:155,135,063-156,194,798	AX-14942447
SGCD	rs32336	chr5:155,135,063-156,194,798	AX-12550084
SGCD	rs32337	chr5:155,135,063-156,194,798	AX-14942450
SGCD	rs3097860	chr5:155,135,063-156,194,798	AX-14942454
SGCD	rs32338	chr5:155,135,063-156,194,798	AX-148096752
SGCD	rs32338	chr5:155,135,063-156,194,798	AX-156282300
SGCD	---	chr5:155,135,063-156,194,798	AX-148605808
SGCD	---	chr5:155,135,063-156,194,798	AX-156275556
SGCD	rs111665014	chr5:155,135,063-156,194,798	AX-148840467
SGCD	rs111665014	chr5:155,135,063-156,194,798	AX-156275557
SGCD	rs3097859	chr5:155,135,063-156,194,798	AX-148120153
SGCD	rs3097859	chr5:155,135,063-156,194,798	AX-156275558
SGCD	rs12109563	chr5:155,135,063-156,194,798	AX-156275559
SGCD	rs12109563	chr5:155,135,063-156,194,798	AX-96815524
SGCD	rs10043109	chr5:155,135,063-156,194,798	AX-14942460
SGCD	rs11748176	chr5:155,135,063-156,194,798	AX-11172458
SGCD	---	chr5:155,135,063-156,194,798	AX-11417872
SGCD	rs17053242	chr5:155,135,063-156,194,798	AX-41644125

SGCD	rs2436423	chr5:155,135,063-156,194,798	AX-41644127
SGCD	rs28678150	chr5:155,135,063-156,194,798	AX-11421996
SGCD	rs116341883	chr5:155,135,063-156,194,798	AX-14942461
SGCD	rs256774	chr5:155,135,063-156,194,798	AX-153340859
SGCD	rs256774	chr5:155,135,063-156,194,798	AX-156284079
SGCD	rs2436330	chr5:155,135,063-156,194,798	AX-148340800
SGCD	rs2436330	chr5:155,135,063-156,194,798	AX-88748592
SGCD	rs4704977	chr5:155,135,063-156,194,798	AX-14942464
SGCD	rs10037288	chr5:155,135,063-156,194,798	AX-156275560
SGCD	rs10037288	chr5:155,135,063-156,194,798	AX-156292731
SGCD	rs74715625	chr5:155,135,063-156,194,798	AX-156278661
SGCD	rs80034015	chr5:155,135,063-156,194,798	AX-14942466
SGCD	rs112787417	chr5:155,135,063-156,194,798	AX-14942468
SGCD	rs72807768	chr5:155,135,063-156,194,798	AX-35168999
SGCD	---	chr5:155,135,063-156,194,798	AX-153368105
SGCD	---	chr5:155,135,063-156,194,798	AX-96700010
SGCD	rs3097854	chr5:155,135,063-156,194,798	AX-148121041
SGCD	rs3097854	chr5:155,135,063-156,194,798	AX-96820597
SGCD	rs200918175	chr5:155,135,063-156,194,798	AX-153373544
SGCD	rs200918175	chr5:155,135,063-156,194,798	AX-156286455
SGCD	rs35364834	chr5:155,135,063-156,194,798	AX-153371277
SGCD	rs35364834	chr5:155,135,063-156,194,798	AX-156298496
SGCD	rs17053243	chr5:155,135,063-156,194,798	AX-41644131
SGCD	rs28589948	chr5:155,135,063-156,194,798	AX-35169001
SGCD	rs10068958	chr5:155,135,063-156,194,798	AX-14942475
SGCD	rs78073979	chr5:155,135,063-156,194,798	AX-35169005
SGCD	---	chr5:155,135,063-156,194,798	AX-41644135
SGCD	rs114154914	chr5:155,135,063-156,194,798	AX-14942477
SGCD	rs28622493	chr5:155,135,063-156,194,798	AX-156275561
SGCD	rs28622493	chr5:155,135,063-156,194,798	AX-92458494
SGCD	rs1594651	chr5:155,135,063-156,194,798	AX-14942479
SGCD	rs71577189	chr5:155,135,063-156,194,798	AX-153230771
SGCD	rs71577189	chr5:155,135,063-156,194,798	AX-156298386
SGCD	rs256771	chr5:155,135,063-156,194,798	AX-11398841
SGCD	rs73811412	chr5:155,135,063-156,194,798	AX-35169013
SGCD	rs256770	chr5:155,135,063-156,194,798	AX-156282301
SGCD	rs256769	chr5:155,135,063-156,194,798	AX-12532113
SGCD	rs56206905	chr5:155,135,063-156,194,798	AX-14942482
SGCD	rs34571631	chr5:155,135,063-156,194,798	AX-153345625
SGCD	rs78223757	chr5:155,135,063-156,194,798	AX-14942491
SGCD	rs10053788	chr5:155,135,063-156,194,798	AX-14942493
SGCD	rs6880666	chr5:155,135,063-156,194,798	AX-156275562
SGCD	rs6880666	chr5:155,135,063-156,194,798	AX-156292732
SGCD	rs3097806	chr5:155,135,063-156,194,798	AX-156292733
SGCD	rs58339191	chr5:155,135,063-156,194,798	AX-122531720
SGCD	rs11955720	chr5:155,135,063-156,194,798	AX-156292734
SGCD	rs1594649	chr5:155,135,063-156,194,798	AX-156275565
SGCD	rs17053245	chr5:155,135,063-156,194,798	AX-12487878
SGCD	rs6888796	chr5:155,135,063-156,194,798	AX-153378570
SGCD	rs6888796	chr5:155,135,063-156,194,798	AX-156284080
SGCD	rs6888987	chr5:155,135,063-156,194,798	AX-148839312
SGCD	rs10078170	chr5:155,135,063-156,194,798	AX-14942503
SGCD	rs73811416	chr5:155,135,063-156,194,798	AX-156275566
SGCD	rs73811416	chr5:155,135,063-156,194,798	AX-156292735
SGCD	rs73811417	chr5:155,135,063-156,194,798	AX-14942508

SGCD	rs77413324	chr5:155,135,063-156,194,798	AX-14942511
SGCD	rs1966638	chr5:155,135,063-156,194,798	AX-14942512
SGCD	rs1966637	chr5:155,135,063-156,194,798	AX-11356496
SGCD	rs73300637	chr5:155,135,063-156,194,798	AX-14942513
SGCD	rs17053247	chr5:155,135,063-156,194,798	AX-41644147
SGCD	rs56342808	chr5:155,135,063-156,194,798	AX-14942515
SGCD	rs73811418	chr5:155,135,063-156,194,798	AX-35169033
SGCD	rs113685185	chr5:155,135,063-156,194,798	AX-14942516
SGCD	rs73811419	chr5:155,135,063-156,194,798	AX-14942517
SGCD	rs76387127	chr5:155,135,063-156,194,798	AX-35169035
SGCD	rs74883185	chr5:155,135,063-156,194,798	AX-35169037
SGCD	rs10055867	chr5:155,135,063-156,194,798	AX-123060398
SGCD	rs10055867	chr5:155,135,063-156,194,798	AX-156282302
SGCD	rs75964912	chr5:155,135,063-156,194,798	AX-148668023
SGCD	rs75964912	chr5:155,135,063-156,194,798	AX-156282303
SGCD	rs17053249	chr5:155,135,063-156,194,798	AX-14942521
SGCD	rs67331625	chr5:155,135,063-156,194,798	AX-14942525
SGCD	rs59728202	chr5:155,135,063-156,194,798	AX-35169045
SGCD	rs7724249	chr5:155,135,063-156,194,798	AX-14942526
SGCD	rs17053251	chr5:155,135,063-156,194,798	AX-12487879
SGCD	rs115327209	chr5:155,135,063-156,194,798	AX-35169049
SGCD	rs17053253	chr5:155,135,063-156,194,798	AX-41644151
SGCD	rs140971432	chr5:155,135,063-156,194,798	AX-153344914
SGCD	rs140971432	chr5:155,135,063-156,194,798	AX-156285268
SGCD	rs73811509	chr5:155,135,063-156,194,798	AX-35169055
SGCD	rs1835927	chr5:155,135,063-156,194,798	AX-156275567
SGCD	rs1835927	chr5:155,135,063-156,194,798	AX-156292736
SGCD	rs72807771	chr5:155,135,063-156,194,798	AX-35169057
SGCD	rs74757950	chr5:155,135,063-156,194,798	AX-35169059
SGCD	rs73811512	chr5:155,135,063-156,194,798	AX-14942530
SGCD	rs56872047	chr5:155,135,063-156,194,798	AX-14942531
SGCD	rs17053254	chr5:155,135,063-156,194,798	AX-41644155
SGCD	rs76492040	chr5:155,135,063-156,194,798	AX-35169065
SGCD	rs10055361	chr5:155,135,063-156,194,798	AX-41644157
SGCD	rs1816398	chr5:155,135,063-156,194,798	AX-11345396
SGCD	rs1821245	chr5:155,135,063-156,194,798	AX-14942533
SGCD	rs1821244	chr5:155,135,063-156,194,798	AX-14942534
SGCD	rs2311448	chr5:155,135,063-156,194,798	AX-41644159
SGCD	rs2311449	chr5:155,135,063-156,194,798	AX-12525645
SGCD	rs2311450	chr5:155,135,063-156,194,798	AX-35169073
SGCD	rs1821243	chr5:155,135,063-156,194,798	AX-12506885
SGCD	rs1821242	chr5:155,135,063-156,194,798	AX-12506884
SGCD	rs10054296	chr5:155,135,063-156,194,798	AX-35169083
SGCD	rs7715033	chr5:155,135,063-156,194,798	AX-12637929
SGCD	rs2311451	chr5:155,135,063-156,194,798	AX-156275568
SGCD	rs2311451	chr5:155,135,063-156,194,798	AX-156292738
SGCD	rs17053262	chr5:155,135,063-156,194,798	AX-14942539
SGCD	rs28532765	chr5:155,135,063-156,194,798	AX-153355868
SGCD	rs28532765	chr5:155,135,063-156,194,798	AX-156275569
SGCD	rs56356206	chr5:155,135,063-156,194,798	AX-14942542
SGCD	rs1972775	chr5:155,135,063-156,194,798	AX-14942543
SGCD	rs11952838	chr5:155,135,063-156,194,798	AX-41644169
SGCD	rs1972774	chr5:155,135,063-156,194,798	AX-156282304
SGCD	rs1972773	chr5:155,135,063-156,194,798	AX-11356812
SGCD	rs11948975	chr5:155,135,063-156,194,798	AX-41644173

SGCD	rs1972772	chr5:155,135,063-156,194,798	AX-41644175
SGCD	rs10063678	chr5:155,135,063-156,194,798	AX-156282305
SGCD	rs10063678	chr5:155,135,063-156,194,798	AX-156296954
SGCD	rs11739922	chr5:155,135,063-156,194,798	AX-14942548
SGCD	rs17053271	chr5:155,135,063-156,194,798	AX-156275570
SGCD	rs17053271	chr5:155,135,063-156,194,798	AX-156292739
SGCD	rs10059221	chr5:155,135,063-156,194,798	AX-156275571
SGCD	rs10059221	chr5:155,135,063-156,194,798	AX-156292740
SGCD	rs4397116	chr5:155,135,063-156,194,798	AX-156275572
SGCD	rs6886990	chr5:155,135,063-156,194,798	AX-14942557
SGCD	rs1835925	chr5:155,135,063-156,194,798	AX-14942562
SGCD	rs1835924	chr5:155,135,063-156,194,798	AX-35169095
SGCD	rs73811522	chr5:155,135,063-156,194,798	AX-14942565
SGCD	rs78920533	chr5:155,135,063-156,194,798	AX-35169099
SGCD	rs56282009	chr5:155,135,063-156,194,798	AX-11708346
SGCD	rs7703516	chr5:155,135,063-156,194,798	AX-149563648
SGCD	rs58716367	chr5:155,135,063-156,194,798	AX-156268202
SGCD	rs7704124	chr5:155,135,063-156,194,798	AX-12637540
SGCD	rs2311452	chr5:155,135,063-156,194,798	AX-156268203
SGCD	rs2311452	chr5:155,135,063-156,194,798	AX-156288054
SGCD	rs150535799	chr5:155,135,063-156,194,798	AX-153360245
SGCD	rs150535799	chr5:155,135,063-156,194,798	AX-156285956
SGCD	rs11741638	chr5:155,135,063-156,194,798	AX-156282306
SGCD	rs11741638	chr5:155,135,063-156,194,798	AX-156296955
SGCD	rs7714789	chr5:155,135,063-156,194,798	AX-12637921
SGCD	rs10866716	chr5:155,135,063-156,194,798	AX-14942582
SGCD	rs4704979	chr5:155,135,063-156,194,798	AX-14942586
SGCD	rs4704980	chr5:155,135,063-156,194,798	AX-156288055
SGCD	rs4704981	chr5:155,135,063-156,194,798	AX-41644201
SGCD	rs7733123	chr5:155,135,063-156,194,798	AX-156288056
SGCD	rs4704983	chr5:155,135,063-156,194,798	AX-119407701
SGCD	rs4704983	chr5:155,135,063-156,194,798	AX-153272440
SGCD	rs79905900	chr5:155,135,063-156,194,798	AX-14942588
SGCD	rs73811523	chr5:155,135,063-156,194,798	AX-14942590
SGCD	rs4704984	chr5:155,135,063-156,194,798	AX-41644207
SGCD	rs114121569	chr5:155,135,063-156,194,798	AX-14942595
SGCD	rs115263165	chr5:155,135,063-156,194,798	AX-14942596
SGCD	rs7709917	chr5:155,135,063-156,194,798	AX-11640322
SGCD	rs73811524	chr5:155,135,063-156,194,798	AX-14942598
SGCD	rs78679963	chr5:155,135,063-156,194,798	AX-14942601
SGCD	rs7726257	chr5:155,135,063-156,194,798	AX-14942602
SGCD	rs6556456	chr5:155,135,063-156,194,798	AX-14942605
SGCD	rs7731274	chr5:155,135,063-156,194,798	AX-41644211
SGCD	rs7731803	chr5:155,135,063-156,194,798	AX-148219024
SGCD	rs7732153	chr5:155,135,063-156,194,798	AX-14942607
SGCD	rs11954107	chr5:155,135,063-156,194,798	AX-41644215
SGCD	rs5872454	chr5:155,135,063-156,194,798	AX-12591353
SGCD	rs5872454	chr5:155,135,063-156,194,798	AX-156275574
SGCD	rs10064955	chr5:155,135,063-156,194,798	AX-156268206
SGCD	rs10064955	chr5:155,135,063-156,194,798	AX-156288057
SGCD	rs10075720	chr5:155,135,063-156,194,798	AX-11091152
SGCD	rs78651833	chr5:155,135,063-156,194,798	AX-14942610
SGCD	rs62380717	chr5:155,135,063-156,194,798	AX-156288058
SGCD	rs73811527	chr5:155,135,063-156,194,798	AX-156268208
SGCD	rs73811527	chr5:155,135,063-156,194,798	AX-156288059

SGCD	rs72809694	chr5:155,135,063-156,194,798	AX-35169123
SGCD	rs7736255	chr5:155,135,063-156,194,798	AX-14942611
SGCD	rs57341712	chr5:155,135,063-156,194,798	AX-153369299
SGCD	rs57341712	chr5:155,135,063-156,194,798	AX-156298224
SGCD	rs73811529	chr5:155,135,063-156,194,798	AX-14942617
SGCD	rs1835922	chr5:155,135,063-156,194,798	AX-156282307
SGCD	rs1835922	chr5:155,135,063-156,194,798	AX-156296957
SGCD	rs111857080	chr5:155,135,063-156,194,798	AX-122409187
SGCD	rs10223195	chr5:155,135,063-156,194,798	AX-14942621
SGCD	---	chr5:155,135,063-156,194,798	AX-105066450
SGCD	rs200439050	chr5:155,135,063-156,194,798	AX-153286716
SGCD	rs200439050	chr5:155,135,063-156,194,798	AX-156285099
SGCD	rs6885373	chr5:155,135,063-156,194,798	AX-11592065
SGCD	rs4526092	chr5:155,135,063-156,194,798	AX-14942623
SGCD	rs2311453	chr5:155,135,063-156,194,798	AX-14942625
SGCD	rs74938463	chr5:155,135,063-156,194,798	AX-156284081
SGCD	rs4704985	chr5:155,135,063-156,194,798	AX-156275575
SGCD	rs4704985	chr5:155,135,063-156,194,798	AX-156292743
SGCD	rs4704986	chr5:155,135,063-156,194,798	AX-41644221
SGCD	rs4704782	chr5:155,135,063-156,194,798	AX-156268210
SGCD	rs4704782	chr5:155,135,063-156,194,798	AX-156288061
SGCD	rs55866075	chr5:155,135,063-156,194,798	AX-14942634
SGCD	rs59869767	chr5:155,135,063-156,194,798	AX-156288062
SGCD	rs57052744	chr5:155,135,063-156,194,798	AX-156288063
SGCD	rs61351228	chr5:155,135,063-156,194,798	AX-35169147
SGCD	rs4704987	chr5:155,135,063-156,194,798	AX-35169149
SGCD	rs28524790	chr5:155,135,063-156,194,798	AX-156268212
SGCD	rs28524790	chr5:155,135,063-156,194,798	AX-156288064
SGCD	rs28407242	chr5:155,135,063-156,194,798	AX-14942638
SGCD	rs6872617	chr5:155,135,063-156,194,798	AX-11591275
SGCD	rs6897151	chr5:155,135,063-156,194,798	AX-14942641
SGCD	rs6860981	chr5:155,135,063-156,194,798	AX-14942643
SGCD	rs6861175	chr5:155,135,063-156,194,798	AX-156282308
SGCD	rs6861175	chr5:155,135,063-156,194,798	AX-95864188
SGCD	rs6890078	chr5:155,135,063-156,194,798	AX-148139623
SGCD	rs6890078	chr5:155,135,063-156,194,798	AX-92732009
SGCD	rs6861703	chr5:155,135,063-156,194,798	AX-35169157
SGCD	rs6890597	chr5:155,135,063-156,194,798	AX-156268213
SGCD	rs6890597	chr5:155,135,063-156,194,798	AX-156288065
SGCD	rs68120551	chr5:155,135,063-156,194,798	AX-148154910
SGCD	rs68120551	chr5:155,135,063-156,194,798	AX-156268214
SGCD	rs10476319	chr5:155,135,063-156,194,798	AX-148049670
SGCD	rs10476319	chr5:155,135,063-156,194,798	AX-156268215
SGCD	rs10475679	chr5:155,135,063-156,194,798	AX-14942649
SGCD	rs4342313	chr5:155,135,063-156,194,798	AX-156268218
SGCD	rs4540164	chr5:155,135,063-156,194,798	AX-156268219
SGCD	rs4626322	chr5:155,135,063-156,194,798	AX-156288070
SGCD	rs35159937	chr5:155,135,063-156,194,798	AX-107834025
SGCD	rs35159937	chr5:155,135,063-156,194,798	AX-156297924
SGCD	rs2871769	chr5:155,135,063-156,194,798	AX-35169171
SGCD	rs60446159	chr5:155,135,063-156,194,798	AX-156268221
SGCD	rs60446159	chr5:155,135,063-156,194,798	AX-156288071
SGCD	rs59484684	chr5:155,135,063-156,194,798	AX-14942654
SGCD	rs57906039	chr5:155,135,063-156,194,798	AX-14942655
SGCD	rs200410807	chr5:155,135,063-156,194,798	AX-153242678

SGCD	rs149090365	chr5:155,135,063-156,194,798	AX-121718257
SGCD	rs1835921	chr5:155,135,063-156,194,798	AX-14942656
SGCD	rs76473087	chr5:155,135,063-156,194,798	AX-14942657
SGCD	rs1835920	chr5:155,135,063-156,194,798	AX-148114871
SGCD	rs1835920	chr5:155,135,063-156,194,798	AX-98009692
SGCD	rs4704784	chr5:155,135,063-156,194,798	AX-156296958
SGCD	rs73811534	chr5:155,135,063-156,194,798	AX-14942661
SGCD	rs76220427	chr5:155,135,063-156,194,798	AX-156292744
SGCD	rs76220427	chr5:155,135,063-156,194,798	AX-50680457
SGCD	rs10073691	chr5:155,135,063-156,194,798	AX-12380492
SGCD	rs7729219	chr5:155,135,063-156,194,798	AX-14942664
SGCD	rs149641799	chr5:155,135,063-156,194,798	AX-118500561
SGCD	rs13159707	chr5:155,135,063-156,194,798	AX-150853961
SGCD	rs13186876	chr5:155,135,063-156,194,798	AX-121862095
SGCD	rs13182225	chr5:155,135,063-156,194,798	AX-153129033
SGCD	rs13182225	chr5:155,135,063-156,194,798	AX-156284082
SGCD	rs7711145	chr5:155,135,063-156,194,798	AX-119759348
SGCD	rs7711145	chr5:155,135,063-156,194,798	AX-96674537
SGCD	rs62380736	chr5:155,135,063-156,194,798	AX-14942666
SGCD	rs73811535	chr5:155,135,063-156,194,798	AX-156268222
SGCD	rs73811535	chr5:155,135,063-156,194,798	AX-156288072
SGCD	rs1835919	chr5:155,135,063-156,194,798	AX-14942667
SGCD	rs1835918	chr5:155,135,063-156,194,798	AX-14942668
SGCD	rs1835917	chr5:155,135,063-156,194,798	AX-35169183
SGCD	rs60536864	chr5:155,135,063-156,194,798	AX-14942670
SGCD	rs6860628	chr5:155,135,063-156,194,798	AX-156268223
SGCD	rs6860628	chr5:155,135,063-156,194,798	AX-156288073
SGCD	rs1835916	chr5:155,135,063-156,194,798	AX-14942674
SGCD	rs991611	chr5:155,135,063-156,194,798	AX-41644239
SGCD	rs12189076	chr5:155,135,063-156,194,798	AX-41644241
SGCD	rs4704988	chr5:155,135,063-156,194,798	AX-41644243
SGCD	rs13165703	chr5:155,135,063-156,194,798	AX-156268224
SGCD	rs13165703	chr5:155,135,063-156,194,798	AX-156288074
SGCD	rs60249910	chr5:155,135,063-156,194,798	AX-14942678
SGCD	rs6883190	chr5:155,135,063-156,194,798	AX-14942679
SGCD	rs6876263	chr5:155,135,063-156,194,798	AX-14942682
SGCD	rs76534858	chr5:155,135,063-156,194,798	AX-153229932
SGCD	rs76534858	chr5:155,135,063-156,194,798	AX-156298581
SGCD	rs11741549	chr5:155,135,063-156,194,798	AX-11171965
SGCD	rs11742893	chr5:155,135,063-156,194,798	AX-148037402
SGCD	rs11742893	chr5:155,135,063-156,194,798	AX-156275576
SGCD	rs13152929	chr5:155,135,063-156,194,798	AX-148431842
SGCD	rs13152929	chr5:155,135,063-156,194,798	AX-156275577
SGCD	rs73811537	chr5:155,135,063-156,194,798	AX-35169197
SGCD	rs35426260	chr5:155,135,063-156,194,798	AX-14942690
SGCD	rs17053320	chr5:155,135,063-156,194,798	AX-14942691
SGCD	rs62380739	chr5:155,135,063-156,194,798	AX-14942692
SGCD	rs4704989	chr5:155,135,063-156,194,798	AX-14942694
SGCD	rs4704991	chr5:155,135,063-156,194,798	AX-156282310
SGCD	rs4704991	chr5:155,135,063-156,194,798	AX-156296959
SGCD	rs4704785	chr5:155,135,063-156,194,798	AX-35169209
SGCD	rs10037014	chr5:155,135,063-156,194,798	AX-14942695
SGCD	rs73811538	chr5:155,135,063-156,194,798	AX-14942696
SGCD	rs12054896	chr5:155,135,063-156,194,798	AX-35169215
SGCD	rs114145307	chr5:155,135,063-156,194,798	AX-14942698

SGCD	rs12054851	chr5:155,135,063-156,194,798	AX-11188546
SGCD	rs12054855	chr5:155,135,063-156,194,798	AX-14942701
SGCD	rs1835915	chr5:155,135,063-156,194,798	AX-14942703
SGCD	rs62380741	chr5:155,135,063-156,194,798	AX-14942707
SGCD	rs57509490	chr5:155,135,063-156,194,798	AX-35169221
SGCD	rs13178125	chr5:155,135,063-156,194,798	AX-41644255
SGCD	rs11950500	chr5:155,135,063-156,194,798	AX-148039467
SGCD	rs11950500	chr5:155,135,063-156,194,798	AX-156275578
SGCD	rs116659662	chr5:155,135,063-156,194,798	AX-38254139
SGCD	rs4704786	chr5:155,135,063-156,194,798	AX-14942711
SGCD	rs4704992	chr5:155,135,063-156,194,798	AX-156275579
SGCD	rs4704992	chr5:155,135,063-156,194,798	AX-156292745
SGCD	rs4704787	chr5:155,135,063-156,194,798	AX-35169235
SGCD	rs62380743	chr5:155,135,063-156,194,798	AX-14942713
SGCD	rs75363043	chr5:155,135,063-156,194,798	AX-148803970
SGCD	rs75363043	chr5:155,135,063-156,194,798	AX-156275580
SGCD	rs12188348	chr5:155,135,063-156,194,798	AX-156275581
SGCD	rs12188348	chr5:155,135,063-156,194,798	AX-156292746
SGCD	rs1968434	chr5:155,135,063-156,194,798	AX-41644257
SGCD	rs62380744	chr5:155,135,063-156,194,798	AX-35169245
SGCD	rs1968433	chr5:155,135,063-156,194,798	AX-41644259
SGCD	rs62380745	chr5:155,135,063-156,194,798	AX-14942715
SGCD	rs11747910	chr5:155,135,063-156,194,798	AX-14942718
SGCD	rs4704993	chr5:155,135,063-156,194,798	AX-35169249
SGCD	rs4704994	chr5:155,135,063-156,194,798	AX-11519466
SGCD	rs17053328	chr5:155,135,063-156,194,798	AX-12487883
SGCD	rs17053330	chr5:155,135,063-156,194,798	AX-35169251
SGCD	rs11748101	chr5:155,135,063-156,194,798	AX-14942721
SGCD	rs1366227	chr5:155,135,063-156,194,798	AX-14942724
SGCD	rs10515735	chr5:155,135,063-156,194,798	AX-41644261
SGCD	rs249887	chr5:155,135,063-156,194,798	AX-41644263
SGCD	rs11951117	chr5:155,135,063-156,194,798	AX-12423956
SGCD	rs17053343	chr5:155,135,063-156,194,798	AX-14942728
SGCD	rs17053346	chr5:155,135,063-156,194,798	AX-14942730
SGCD	rs30283	chr5:155,135,063-156,194,798	AX-156275583
SGCD	rs30283	chr5:155,135,063-156,194,798	AX-156292748
SGCD	rs56660863	chr5:155,135,063-156,194,798	AX-14942733
SGCD	rs17053347	chr5:155,135,063-156,194,798	AX-156275584
SGCD	rs17053347	chr5:155,135,063-156,194,798	AX-156292749
SGCD	rs57229190	chr5:155,135,063-156,194,798	AX-35169265
SGCD	rs249886	chr5:155,135,063-156,194,798	AX-156268225
SGCD	rs249886	chr5:155,135,063-156,194,798	AX-156288075
SGCD	rs249885	chr5:155,135,063-156,194,798	AX-14942741
SGCD	rs62380749	chr5:155,135,063-156,194,798	AX-14942743
SGCD	rs249884	chr5:155,135,063-156,194,798	AX-14942744
SGCD	rs73811546	chr5:155,135,063-156,194,798	AX-156282311
SGCD	rs73811546	chr5:155,135,063-156,194,798	AX-156296960
SGCD	rs249883	chr5:155,135,063-156,194,798	AX-156275585
SGCD	rs77357980	chr5:155,135,063-156,194,798	AX-148862389
SGCD	rs77357980	chr5:155,135,063-156,194,798	AX-156275586
SGCD	rs72811731	chr5:155,135,063-156,194,798	AX-35169271
SGCD	rs249881	chr5:155,135,063-156,194,798	AX-12530691
SGCD	rs249880	chr5:155,135,063-156,194,798	AX-122892240
SGCD	rs249880	chr5:155,135,063-156,194,798	AX-156268226
SGCD	rs249878	chr5:155,135,063-156,194,798	AX-96092285

SGCD	rs77156821	chr5:155,135,063-156,194,798	AX-35169275
SGCD	rs17053349	chr5:155,135,063-156,194,798	AX-14942763
SGCD	rs17053350	chr5:155,135,063-156,194,798	AX-14942764
SGCD	rs30276	chr5:155,135,063-156,194,798	AX-41644285
SGCD	rs39584	chr5:155,135,063-156,194,798	AX-14942772
SGCD	rs114603152	chr5:155,135,063-156,194,798	AX-66999955
SGCD	rs405179	chr5:155,135,063-156,194,798	AX-11489633
SGCD	rs447817	chr5:155,135,063-156,194,798	AX-14942779
SGCD	rs249872	chr5:155,135,063-156,194,798	AX-128218793
SGCD	rs249872	chr5:155,135,063-156,194,798	AX-156268228
SGCD	rs11949402	chr5:155,135,063-156,194,798	AX-148189520
SGCD	rs11949402	chr5:155,135,063-156,194,798	AX-156268229
SGCD	rs249873	chr5:155,135,063-156,194,798	AX-153230559
SGCD	rs249873	chr5:155,135,063-156,194,798	AX-156268230
SGCD	rs249874	chr5:155,135,063-156,194,798	AX-148363020
SGCD	rs249874	chr5:155,135,063-156,194,798	AX-156282312
SGCD	rs7730492	chr5:155,135,063-156,194,798	AX-119367156
SGCD	rs7730492	chr5:155,135,063-156,194,798	AX-156284083
SGCD	rs7730494	chr5:155,135,063-156,194,798	AX-119652480
SGCD	rs7730494	chr5:155,135,063-156,194,798	AX-121550022
SGCD	rs670814	chr5:155,135,063-156,194,798	AX-148397848
SGCD	rs670814	chr5:155,135,063-156,194,798	AX-156268231
SGCD	rs184548079	chr5:155,135,063-156,194,798	AX-148824028
SGCD	rs184548079	chr5:155,135,063-156,194,798	AX-156284084
SGCD	rs74883231	chr5:155,135,063-156,194,798	AX-113210907
SGCD	rs74883231	chr5:155,135,063-156,194,798	AX-130112879
SGCD	rs28750556	chr5:155,135,063-156,194,798	AX-148173370
SGCD	rs28750556	chr5:155,135,063-156,194,798	AX-149107882
SGCD	rs59232655	chr5:155,135,063-156,194,798	AX-148883356
SGCD	rs59232655	chr5:155,135,063-156,194,798	AX-156284085
SGCD	rs249875	chr5:155,135,063-156,194,798	AX-153227221
SGCD	rs249875	chr5:155,135,063-156,194,798	AX-156268232
SGCD	rs30273	chr5:155,135,063-156,194,798	AX-14942792
SGCD	rs30274	chr5:155,135,063-156,194,798	AX-14942797
SGCD	rs75940341	chr5:155,135,063-156,194,798	AX-14942800
SGCD	rs30275	chr5:155,135,063-156,194,798	AX-14942805
SGCD	rs249877	chr5:155,135,063-156,194,798	AX-14942806
SGCD	rs249894	chr5:155,135,063-156,194,798	AX-147998027
SGCD	rs249894	chr5:155,135,063-156,194,798	AX-153811297
SGCD	rs249892	chr5:155,135,063-156,194,798	AX-41644293
SGCD	rs249891	chr5:155,135,063-156,194,798	AX-14942807
SGCD	rs2312058	chr5:155,135,063-156,194,798	AX-148180451
SGCD	rs2312058	chr5:155,135,063-156,194,798	AX-148370058
SGCD	rs249890	chr5:155,135,063-156,194,798	AX-156275587
SGCD	rs249890	chr5:155,135,063-156,194,798	AX-92858174
SGCD	rs249889	chr5:155,135,063-156,194,798	AX-120238670
SGCD	rs249889	chr5:155,135,063-156,194,798	AX-156275588
SGCD	rs189692026	chr5:155,135,063-156,194,798	AX-113772039
SGCD	rs189692026	chr5:155,135,063-156,194,798	AX-156292751
SGCD	rs249888	chr5:155,135,063-156,194,798	AX-11395142
SGCD	rs57499459	chr5:155,135,063-156,194,798	AX-14942813
SGCD	rs70982001	chr5:155,135,063-156,194,798	AX-153284949
SGCD	rs70982001	chr5:155,135,063-156,194,798	AX-156284281
SGCD	rs17053358	chr5:155,135,063-156,194,798	AX-12487885
SGCD	rs62382677	chr5:155,135,063-156,194,798	AX-14942815

SGCD	rs17053360	chr5:155,135,063-156,194,798	AX-14942817
SGCD	rs30282	chr5:155,135,063-156,194,798	AX-11432267
SGCD	rs187495	chr5:155,135,063-156,194,798	AX-12508521
SGCD	rs30281	chr5:155,135,063-156,194,798	AX-14942820
SGCD	rs30280	chr5:155,135,063-156,194,798	AX-14942821
SGCD	rs4704996	chr5:155,135,063-156,194,798	AX-14942822
SGCD	rs4704997	chr5:155,135,063-156,194,798	AX-14942823
SGCD	rs62382678	chr5:155,135,063-156,194,798	AX-14942824
SGCD	rs62382679	chr5:155,135,063-156,194,798	AX-14942825
SGCD	rs62382681	chr5:155,135,063-156,194,798	AX-148237686
SGCD	rs62382681	chr5:155,135,063-156,194,798	AX-156268233
SGCD	rs13354310	chr5:155,135,063-156,194,798	AX-14942826
SGCD	rs17053370	chr5:155,135,063-156,194,798	AX-12487886
SGCD	rs114395809	chr5:155,135,063-156,194,798	AX-14942827
SGCD	rs2033208	chr5:155,135,063-156,194,798	AX-14942831
SGCD	rs139241635	chr5:155,135,063-156,194,798	AX-113235502
SGCD	rs139241635	chr5:155,135,063-156,194,798	AX-156284086
SGCD	rs1896632	chr5:155,135,063-156,194,798	AX-35169343
SGCD	rs1896631	chr5:155,135,063-156,194,798	AX-41644301
SGCD	rs112337099	chr5:155,135,063-156,194,798	AX-119334082
SGCD	rs2033207	chr5:155,135,063-156,194,798	AX-12514574
SGCD	rs10075212	chr5:155,135,063-156,194,798	AX-14942835
SGCD	rs4235721	chr5:155,135,063-156,194,798	AX-14942837
SGCD	rs4704998	chr5:155,135,063-156,194,798	AX-41644303
SGCD	rs1593883	chr5:155,135,063-156,194,798	AX-148217061
SGCD	rs1593883	chr5:155,135,063-156,194,798	AX-156275589
SGCD	rs1428135	chr5:155,135,063-156,194,798	AX-156275590
SGCD	rs1428135	chr5:155,135,063-156,194,798	AX-156292752
SGCD	rs6556512	chr5:155,135,063-156,194,798	AX-14942839
SGCD	rs4560532	chr5:155,135,063-156,194,798	AX-156275591
SGCD	rs4560532	chr5:155,135,063-156,194,798	AX-156292753
SGCD	rs6556514	chr5:155,135,063-156,194,798	AX-14942844
SGCD	rs10058777	chr5:155,135,063-156,194,798	AX-14942845
SGCD	rs10593621	chr5:155,135,063-156,194,798	AX-156285763
SGCD	rs7725829	chr5:155,135,063-156,194,798	AX-14942847
SGCD	rs1428139	chr5:155,135,063-156,194,798	AX-14942848
SGCD	rs10062793	chr5:155,135,063-156,194,798	AX-156268234
SGCD	rs10062793	chr5:155,135,063-156,194,798	AX-156288077
SGCD	rs1121854	chr5:155,135,063-156,194,798	AX-156268235
SGCD	rs1121854	chr5:155,135,063-156,194,798	AX-156288078
SGCD	rs60559509	chr5:155,135,063-156,194,798	AX-156296961
SGCD	rs4134197	chr5:155,135,063-156,194,798	AX-14942853
SGCD	rs61215787	chr5:155,135,063-156,194,798	AX-14942855
SGCD	rs12653936	chr5:155,135,063-156,194,798	AX-14942857
SGCD	rs34501775	chr5:155,135,063-156,194,798	AX-41644315
SGCD	rs1346486	chr5:155,135,063-156,194,798	AX-14942861
SGCD	rs73811554	chr5:155,135,063-156,194,798	AX-14942864
SGCD	rs10038926	chr5:155,135,063-156,194,798	AX-156292755
SGCD	rs2312059	chr5:155,135,063-156,194,798	AX-11384321
SGCD	rs56874129	chr5:155,135,063-156,194,798	AX-156292756
SGCD	rs1593885	chr5:155,135,063-156,194,798	AX-156275595
SGCD	rs1593885	chr5:155,135,063-156,194,798	AX-156292757
SGCD	rs1593884	chr5:155,135,063-156,194,798	AX-156275596
SGCD	rs1593884	chr5:155,135,063-156,194,798	AX-156292758
SGCD	rs1834945	chr5:155,135,063-156,194,798	AX-156275597

SGCD	rs1834945	chr5:155,135,063-156,194,798	AX-156292759
SGCD	rs35052017	chr5:155,135,063-156,194,798	AX-35169375
SGCD	rs4461618	chr5:155,135,063-156,194,798	AX-156275598
SGCD	rs4461618	chr5:155,135,063-156,194,798	AX-156292760
SGCD	rs972324	chr5:155,135,063-156,194,798	AX-156268236
SGCD	rs972324	chr5:155,135,063-156,194,798	AX-156288079
SGCD	rs1366228	chr5:155,135,063-156,194,798	AX-14942872
SGCD	rs6866397	chr5:155,135,063-156,194,798	AX-147840386
SGCD	rs6866397	chr5:155,135,063-156,194,798	AX-147934522
SGCD	rs4555777	chr5:155,135,063-156,194,798	AX-11510966
SGCD	rs17053376	chr5:155,135,063-156,194,798	AX-156282313
SGCD	rs115251155	chr5:155,135,063-156,194,798	AX-14942877
SGCD	rs17628942	chr5:155,135,063-156,194,798	AX-14942878
SGCD	rs17053378	chr5:155,135,063-156,194,798	AX-41644337
SGCD	rs12513679	chr5:155,135,063-156,194,798	AX-156292761
SGCD	rs13170433	chr5:155,135,063-156,194,798	AX-41644343
SGCD	rs10060287	chr5:155,135,063-156,194,798	AX-156275600
SGCD	rs10515734	chr5:155,135,063-156,194,798	AX-14942882
SGCD	rs143810910	chr5:155,135,063-156,194,798	AX-153340623
SGCD	rs143810910	chr5:155,135,063-156,194,798	AX-156286437
SGCD	rs12716365	chr5:155,135,063-156,194,798	AX-156275601
SGCD	rs12716365	chr5:155,135,063-156,194,798	AX-156292763
SGCD	rs12517231	chr5:155,135,063-156,194,798	AX-148453253
SGCD	rs12517231	chr5:155,135,063-156,194,798	AX-156275602
SGCD	rs116357544	chr5:155,135,063-156,194,798	AX-148588868
SGCD	rs116357544	chr5:155,135,063-156,194,798	AX-156269266
SGCD	rs12187758	chr5:155,135,063-156,194,798	AX-122375624
SGCD	rs113997386	chr5:155,135,063-156,194,798	AX-120470350
SGCD	rs113997386	chr5:155,135,063-156,194,798	AX-148389626
SGCD	rs73302651	chr5:155,135,063-156,194,798	AX-156275603
SGCD	rs11953745	chr5:155,135,063-156,194,798	AX-156275604
SGCD	rs11953745	chr5:155,135,063-156,194,798	AX-156292764
SGCD	rs12716369	chr5:155,135,063-156,194,798	AX-156275605
SGCD	rs12716370	chr5:155,135,063-156,194,798	AX-148430462
SGCD	rs12716370	chr5:155,135,063-156,194,798	AX-156275606
SGCD	rs34676117	chr5:155,135,063-156,194,798	AX-107832603
SGCD	rs34676117	chr5:155,135,063-156,194,798	AX-156298348
SGCD	rs7341071	chr5:155,135,063-156,194,798	AX-14942887
SGCD	rs73302655	chr5:155,135,063-156,194,798	AX-14942893
SGCD	rs12657973	chr5:155,135,063-156,194,798	AX-156268237
SGCD	rs12657973	chr5:155,135,063-156,194,798	AX-156288080
SGCD	---	chr5:155,135,063-156,194,798	AX-35169397
SGCD	rs12655476	chr5:155,135,063-156,194,798	AX-14942894
SGCD	rs75300904	chr5:155,135,063-156,194,798	AX-38254185
SGCD	rs78835680	chr5:155,135,063-156,194,798	AX-35169399
SGCD	rs62383776	chr5:155,135,063-156,194,798	AX-14942896
SGCD	rs76887603	chr5:155,135,063-156,194,798	AX-14942897
SGCD	rs143174874	chr5:155,135,063-156,194,798	AX-156284385
SGCD	rs13153827	chr5:155,135,063-156,194,798	AX-156282314
SGCD	rs13153827	chr5:155,135,063-156,194,798	AX-156296963
SGCD	rs17553409	chr5:155,135,063-156,194,798	AX-14942901
SGCD	rs10066281	chr5:155,135,063-156,194,798	AX-41644353
SGCD	rs6865821	chr5:155,135,063-156,194,798	AX-35169409
SGCD	---	chr5:155,135,063-156,194,798	AX-35169411
SGCD	rs10068407	chr5:155,135,063-156,194,798	AX-41644355

SGCD	rs10059484	chr5:155,135,063-156,194,798	AX-14942909
SGCD	rs56282784	chr5:155,135,063-156,194,798	AX-14942910
SGCD	rs10065545	chr5:155,135,063-156,194,798	AX-11090521
SGCD	rs1504932	chr5:155,135,063-156,194,798	AX-14942912
SGCD	rs10061360	chr5:155,135,063-156,194,798	AX-41644357
SGCD	rs11956108	chr5:155,135,063-156,194,798	AX-12424089
SGCD	rs73811576	chr5:155,135,063-156,194,798	AX-14942914
SGCD	rs988686	chr5:155,135,063-156,194,798	AX-41644361
SGCD	rs10074679	chr5:155,135,063-156,194,798	AX-14942919
SGCD	rs57752082	chr5:155,135,063-156,194,798	AX-14942923
SGCD	rs73811577	chr5:155,135,063-156,194,798	AX-14942926
SGCD	rs73811578	chr5:155,135,063-156,194,798	AX-14942928
SGCD	rs11370605	chr5:155,135,063-156,194,798	AX-156275607
SGCD	rs201189503	chr5:155,135,063-156,194,798	AX-120624769
SGCD	rs60202362	chr5:155,135,063-156,194,798	AX-156282315
SGCD	rs60202362	chr5:155,135,063-156,194,798	AX-156296964
SGCD	rs61271334	chr5:155,135,063-156,194,798	AX-156282316
SGCD	rs61271334	chr5:155,135,063-156,194,798	AX-156296965
SGCD	rs17553613	chr5:155,135,063-156,194,798	AX-35169419
SGCD	rs57539502	chr5:155,135,063-156,194,798	AX-14942934
SGCD	rs10064971	chr5:155,135,063-156,194,798	AX-11090471
SGCD	rs62383781	chr5:155,135,063-156,194,798	AX-148548294
SGCD	rs62383781	chr5:155,135,063-156,194,798	AX-156284087
SGCD	rs182900273	chr5:155,135,063-156,194,798	AX-105103062
SGCD	rs182900273	chr5:155,135,063-156,194,798	AX-105103063
SGCD	rs6879742	chr5:155,135,063-156,194,798	AX-149047780
SGCD	rs6879742	chr5:155,135,063-156,194,798	AX-96742525
SGCD	rs6864200	chr5:155,135,063-156,194,798	AX-120889774
SGCD	rs6864200	chr5:155,135,063-156,194,798	AX-156292765
SGCD	rs6864242	chr5:155,135,063-156,194,798	AX-148490498
SGCD	rs6864242	chr5:155,135,063-156,194,798	AX-151755869
SGCD	rs187508610	chr5:155,135,063-156,194,798	AX-120088582
SGCD	rs187508610	chr5:155,135,063-156,194,798	AX-148456651
SGCD	rs147140950	chr5:155,135,063-156,194,798	AX-151375356
SGCD	rs147140950	chr5:155,135,063-156,194,798	AX-156284088
SGCD	rs12523569	chr5:155,135,063-156,194,798	AX-151300728
SGCD	rs12523569	chr5:155,135,063-156,194,798	AX-156284089
SGCD	rs76535153	chr5:155,135,063-156,194,798	AX-148712230
SGCD	rs76535153	chr5:155,135,063-156,194,798	AX-156284090
SGCD	rs10071376	chr5:155,135,063-156,194,798	AX-41644369
SGCD	rs12657569	chr5:155,135,063-156,194,798	AX-14942937
SGCD	rs10050429	chr5:155,135,063-156,194,798	AX-41644371
SGCD	rs10052410	chr5:155,135,063-156,194,798	AX-11089704
SGCD	rs12658724	chr5:155,135,063-156,194,798	AX-14942940
SGCD	rs17053396	chr5:155,135,063-156,194,798	AX-11301510
SGCD	rs71672922	chr5:155,135,063-156,194,798	AX-151262722
SGCD	rs71672922	chr5:155,135,063-156,194,798	AX-156286072
SGCD	rs17053400	chr5:155,135,063-156,194,798	AX-41644375
SGCD	rs10077764	chr5:155,135,063-156,194,798	AX-12380624
SGCD	rs6887144	chr5:155,135,063-156,194,798	AX-156268239
SGCD	rs6887144	chr5:155,135,063-156,194,798	AX-156288082
SGCD	rs72811747	chr5:155,135,063-156,194,798	AX-14942948
SGCD	rs2174420	chr5:155,135,063-156,194,798	AX-156288083
SGCD	rs2135033	chr5:155,135,063-156,194,798	AX-41644379
SGCD	rs2312060	chr5:155,135,063-156,194,798	AX-41644381

SGCD	rs10054153	chr5:155,135,063-156,194,798	AX-156268241
SGCD	rs10054153	chr5:155,135,063-156,194,798	AX-156288084
SGCD	rs10054192	chr5:155,135,063-156,194,798	AX-156268242
SGCD	rs10054192	chr5:155,135,063-156,194,798	AX-156288085
SGCD	rs9885248	chr5:155,135,063-156,194,798	AX-35169427
SGCD	rs2089189	chr5:155,135,063-156,194,798	AX-156282317
SGCD	rs2089189	chr5:155,135,063-156,194,798	AX-156296966
SGCD	rs2312061	chr5:155,135,063-156,194,798	AX-12525657
SGCD	rs34232746	chr5:155,135,063-156,194,798	AX-156285370
SGCD	rs78679437	chr5:155,135,063-156,194,798	AX-156268243
SGCD	rs78679437	chr5:155,135,063-156,194,798	AX-156288086
SGCD	rs1604960	chr5:155,135,063-156,194,798	AX-11278462
SGCD	rs55805499	chr5:155,135,063-156,194,798	AX-14942959
SGCD	rs397515714	chr5:155,135,063-156,194,798	AX-156285101
SGCD	rs182881432	chr5:155,135,063-156,194,798	AX-105100966
SGCD	rs182881432	chr5:155,135,063-156,194,798	AX-96656038
SGCD	rs192170911	chr5:155,135,063-156,194,798	AX-120302901
SGCD	rs192170911	chr5:155,135,063-156,194,798	AX-120722574
SGCD	rs182712075	chr5:155,135,063-156,194,798	AX-119748218
SGCD	rs182712075	chr5:155,135,063-156,194,798	AX-156284091
SGCD	rs34887087	chr5:155,135,063-156,194,798	AX-11454674
SGCD	rs6878121	chr5:155,135,063-156,194,798	AX-156268245
SGCD	rs6878121	chr5:155,135,063-156,194,798	AX-156288087
SGCD	rs13358565	chr5:155,135,063-156,194,798	AX-156288088
SGCD	rs28826428	chr5:155,135,063-156,194,798	AX-148184800
SGCD	rs28826428	chr5:155,135,063-156,194,798	AX-156268247
SGCD	rs79876175	chr5:155,135,063-156,194,798	AX-14942969
SGCD	rs36049454	chr5:155,135,063-156,194,798	AX-120891353
SGCD	rs36049454	chr5:155,135,063-156,194,798	AX-156282318
SGCD	rs1604961	chr5:155,135,063-156,194,798	AX-156282319
SGCD	rs1604961	chr5:155,135,063-156,194,798	AX-156296967
SGCD	rs73811589	chr5:155,135,063-156,194,798	AX-156282320
SGCD	rs62380676	chr5:155,135,063-156,194,798	AX-156275608
SGCD	rs62380676	chr5:155,135,063-156,194,798	AX-156292766
SGCD	rs1580927	chr5:155,135,063-156,194,798	AX-14942974
SGCD	---	chr5:155,135,063-156,194,798	AX-156278662
SGCD	---	chr5:155,135,063-156,194,798	AX-156294866
SGCD	rs10063909	chr5:155,135,063-156,194,798	AX-35169447
SGCD	rs6556541	chr5:155,135,063-156,194,798	AX-156288090
SGCD	rs2221439	chr5:155,135,063-156,194,798	AX-151190625
SGCD	rs2221439	chr5:155,135,063-156,194,798	AX-156268250
SGCD	rs2202432	chr5:155,135,063-156,194,798	AX-151271331
SGCD	rs2202432	chr5:155,135,063-156,194,798	AX-156284092
SGCD	rs2102285	chr5:155,135,063-156,194,798	AX-156268251
SGCD	rs114902158	chr5:155,135,063-156,194,798	AX-148305386
SGCD	rs114902158	chr5:155,135,063-156,194,798	AX-148719319
SGCD	rs72813859	chr5:155,135,063-156,194,798	AX-156288091
SGCD	rs141393998	chr5:155,135,063-156,194,798	AX-151384324
SGCD	rs141393998	chr5:155,135,063-156,194,798	AX-156284301
SGCD	rs202032391	chr5:155,135,063-156,194,798	AX-148184451
SGCD	rs202032391	chr5:155,135,063-156,194,798	AX-151434519
SGCD	rs2135034	chr5:155,135,063-156,194,798	AX-148179921
SGCD	rs2135034	chr5:155,135,063-156,194,798	AX-156268253
SGCD	rs2135035	chr5:155,135,063-156,194,798	AX-148189280
SGCD	rs2135035	chr5:155,135,063-156,194,798	AX-156268254

SGCD	rs7704662	chr5:155,135,063-156,194,798	AX-41644395
SGCD	rs201510325	chr5:155,135,063-156,194,798	AX-119630896
SGCD	rs201510325	chr5:155,135,063-156,194,798	AX-84630771
SGCD	rs143136453	chr5:155,135,063-156,194,798	AX-121079258
SGCD	rs143136453	chr5:155,135,063-156,194,798	AX-148317683
SGCD	rs76362912	chr5:155,135,063-156,194,798	AX-156282321
SGCD	rs76362912	chr5:155,135,063-156,194,798	AX-156296969
SGCD	rs2135036	chr5:155,135,063-156,194,798	AX-14942988
SGCD	rs2135037	chr5:155,135,063-156,194,798	AX-14942990
SGCD	rs1504940	chr5:155,135,063-156,194,798	AX-14942991
SGCD	rs116356591	chr5:155,135,063-156,194,798	AX-14942992
SGCD	rs1504942	chr5:155,135,063-156,194,798	AX-14942994
SGCD	rs76245178	chr5:155,135,063-156,194,798	AX-14942996
SGCD	---	chr5:155,135,063-156,194,798	AX-156285873
SGCD	rs6556545	chr5:155,135,063-156,194,798	AX-156268256
SGCD	rs7737496	chr5:155,135,063-156,194,798	AX-11642104
SGCD	rs11744424	chr5:155,135,063-156,194,798	AX-41644403
SGCD	rs10044520	chr5:155,135,063-156,194,798	AX-41644405
SGCD	rs6898100	chr5:155,135,063-156,194,798	AX-156288093
SGCD	rs1604962	chr5:155,135,063-156,194,798	AX-156268258
SGCD	rs1604962	chr5:155,135,063-156,194,798	AX-156288094
SGCD	rs7710388	chr5:155,135,063-156,194,798	AX-156268259
SGCD	rs7710388	chr5:155,135,063-156,194,798	AX-156288095
SGCD	rs1857768	chr5:155,135,063-156,194,798	AX-14943003
SGCD	rs4705000	chr5:155,135,063-156,194,798	AX-14943005
SGCD	rs10463088	chr5:155,135,063-156,194,798	AX-41644415
SGCD	rs74973159	chr5:155,135,063-156,194,798	AX-14943009
SGCD	rs4704790	chr5:155,135,063-156,194,798	AX-148195917
SGCD	rs4704790	chr5:155,135,063-156,194,798	AX-156275609
SGCD	rs6885958	chr5:155,135,063-156,194,798	AX-148101100
SGCD	rs6885958	chr5:155,135,063-156,194,798	AX-156275610
SGCD	rs6886148	chr5:155,135,063-156,194,798	AX-148118954
SGCD	rs112872065	chr5:155,135,063-156,194,798	AX-148725217
SGCD	rs4705001	chr5:155,135,063-156,194,798	AX-14943011
SGCD	rs113789074	chr5:155,135,063-156,194,798	AX-14943012
SGCD	rs74953545	chr5:155,135,063-156,194,798	AX-156275611
SGCD	rs74953545	chr5:155,135,063-156,194,798	AX-156292767
SGCD	rs6875001	chr5:155,135,063-156,194,798	AX-41644419
SGCD	rs6875169	chr5:155,135,063-156,194,798	AX-11591443
SGCD	rs10045825	chr5:155,135,063-156,194,798	AX-156275612
SGCD	rs7736412	chr5:155,135,063-156,194,798	AX-14943020
SGCD	rs7737383	chr5:155,135,063-156,194,798	AX-119554053
SGCD	rs7737383	chr5:155,135,063-156,194,798	AX-156275613
SGCD	rs7730533	chr5:155,135,063-156,194,798	AX-120399535
SGCD	rs10038955	chr5:155,135,063-156,194,798	AX-41644427
SGCD	rs74418250	chr5:155,135,063-156,194,798	AX-156268261
SGCD	rs74418250	chr5:155,135,063-156,194,798	AX-156288096
SGCD	rs75817468	chr5:155,135,063-156,194,798	AX-14943023
SGCD	rs115677644	chr5:155,135,063-156,194,798	AX-14943024
SGCD	rs4705002	chr5:155,135,063-156,194,798	AX-11519468
SGCD	rs4235722	chr5:155,135,063-156,194,798	AX-14943025
SGCD	rs77956244	chr5:155,135,063-156,194,798	AX-14943027
SGCD	rs7737367	chr5:155,135,063-156,194,798	AX-41644429
SGCD	rs34400642	chr5:155,135,063-156,194,798	AX-120142706
SGCD	rs11738065	chr5:155,135,063-156,194,798	AX-14943028

SGCD	rs4705003	chr5:155,135,063-156,194,798	AX-14943031
SGCD	rs7729633	chr5:155,135,063-156,194,798	AX-11641603
SGCD	rs77723579	chr5:155,135,063-156,194,798	AX-14943034
SGCD	rs10057826	chr5:155,135,063-156,194,798	AX-156275614
SGCD	rs10057826	chr5:155,135,063-156,194,798	AX-156292769
SGCD	rs10058119	chr5:155,135,063-156,194,798	AX-156275615
SGCD	rs10058119	chr5:155,135,063-156,194,798	AX-156292770
SGCD	rs76169209	chr5:155,135,063-156,194,798	AX-14943038
SGCD	rs7724969	chr5:155,135,063-156,194,798	AX-41644435
SGCD	rs13170573	chr5:155,135,063-156,194,798	AX-156275616
SGCD	rs13170573	chr5:155,135,063-156,194,798	AX-156292771
SGCD	rs17554270	chr5:155,135,063-156,194,798	AX-14943045
SGCD	rs7713117	chr5:155,135,063-156,194,798	AX-41644443
SGCD	rs73304431	chr5:155,135,063-156,194,798	AX-35169489
SGCD	rs10064593	chr5:155,135,063-156,194,798	AX-156292772
SGCD	rs11744154	chr5:155,135,063-156,194,798	AX-14943047
SGCD	rs11740347	chr5:155,135,063-156,194,798	AX-14943049
SGCD	rs10447212	chr5:155,135,063-156,194,798	AX-156275618
SGCD	rs10447212	chr5:155,135,063-156,194,798	AX-92620672
SGCD	rs142576328	chr5:155,135,063-156,194,798	AX-148656630
SGCD	rs142576328	chr5:155,135,063-156,194,798	AX-156284093
SGCD	rs60426853	chr5:155,135,063-156,194,798	AX-156284094
SGCD	rs4704791	chr5:155,135,063-156,194,798	AX-14943054
SGCD	rs4704792	chr5:155,135,063-156,194,798	AX-14943057
SGCD	rs75194700	chr5:155,135,063-156,194,798	AX-14943058
SGCD	rs4705004	chr5:155,135,063-156,194,798	AX-11519469
SGCD	rs149207751	chr5:155,135,063-156,194,798	AX-120369387
SGCD	rs149207751	chr5:155,135,063-156,194,798	AX-156298104
SGCD	rs4705005	chr5:155,135,063-156,194,798	AX-14943061
SGCD	rs78000700	chr5:155,135,063-156,194,798	AX-14943062
SGCD	rs4705006	chr5:155,135,063-156,194,798	AX-14943063
SGCD	rs1354565	chr5:155,135,063-156,194,798	AX-12459350
SGCD	rs1394605	chr5:155,135,063-156,194,798	AX-156292773
SGCD	rs1394606	chr5:155,135,063-156,194,798	AX-50848678
SGCD	rs1948863	chr5:155,135,063-156,194,798	AX-156275620
SGCD	rs79672495	chr5:155,135,063-156,194,798	AX-14943067
SGCD	rs79179968	chr5:155,135,063-156,194,798	AX-14943072
SGCD	rs6893125	chr5:155,135,063-156,194,798	AX-14943077
SGCD	rs4705007	chr5:155,135,063-156,194,798	AX-14943080
SGCD	rs4705008	chr5:155,135,063-156,194,798	AX-14943081
SGCD	rs78571373	chr5:155,135,063-156,194,798	AX-14943083
SGCD	rs10462927	chr5:155,135,063-156,194,798	AX-35169493
SGCD	rs72813885	chr5:155,135,063-156,194,798	AX-14943085
SGCD	rs13160261	chr5:155,135,063-156,194,798	AX-35169495
SGCD	rs1354566	chr5:155,135,063-156,194,798	AX-156275621
SGCD	rs1354566	chr5:155,135,063-156,194,798	AX-156292775
SGCD	rs10061194	chr5:155,135,063-156,194,798	AX-156282323
SGCD	rs10061194	chr5:155,135,063-156,194,798	AX-156296971
SGCD	rs73815031	chr5:155,135,063-156,194,798	AX-14943090
SGCD	rs10039835	chr5:155,135,063-156,194,798	AX-11088940
SGCD	rs10042069	chr5:155,135,063-156,194,798	AX-12379471
SGCD	rs4704793	chr5:155,135,063-156,194,798	AX-14943093
SGCD	rs74940388	chr5:155,135,063-156,194,798	AX-14943094
SGCD	rs60947826	chr5:155,135,063-156,194,798	AX-35169499
SGCD	rs10074370	chr5:155,135,063-156,194,798	AX-147828730

SGCD	rs149320789	chr5:155,135,063-156,194,798	AX-151363102
SGCD	rs10065774	chr5:155,135,063-156,194,798	AX-14943100
SGCD	rs6888978	chr5:155,135,063-156,194,798	AX-41644461
SGCD	rs147489698	chr5:155,135,063-156,194,798	AX-121393271
SGCD	rs147489698	chr5:155,135,063-156,194,798	AX-156284479
SGCD	rs2135038	chr5:155,135,063-156,194,798	AX-14943103
SGCD	rs4394110	chr5:155,135,063-156,194,798	AX-41644463
SGCD	rs80146179	chr5:155,135,063-156,194,798	AX-35169501
SGCD	rs2135039	chr5:155,135,063-156,194,798	AX-14943104
SGCD	rs201754678	chr5:155,135,063-156,194,798	AX-151155398
SGCD	rs6894973	chr5:155,135,063-156,194,798	AX-14943108
SGCD	rs78308722	chr5:155,135,063-156,194,798	AX-14943111
SGCD	rs1857770	chr5:155,135,063-156,194,798	AX-11347216
SGCD	rs17053449	chr5:155,135,063-156,194,798	AX-156282324
SGCD	rs17053449	chr5:155,135,063-156,194,798	AX-156296972
SGCD	rs7725923	chr5:155,135,063-156,194,798	AX-14943115
SGCD	rs2871924	chr5:155,135,063-156,194,798	AX-41644469
SGCD	---	chr5:155,135,063-156,194,798	AX-156282325
SGCD	---	chr5:155,135,063-156,194,798	AX-156296973
SGCD	rs7714142	chr5:155,135,063-156,194,798	AX-14943116
SGCD	rs6556552	chr5:155,135,063-156,194,798	AX-14943117
SGCD	rs8180473	chr5:155,135,063-156,194,798	AX-35169511
SGCD	---	chr5:155,135,063-156,194,798	AX-156284709
SGCD	rs1801193	chr5:155,135,063-156,194,798	AX-14943120
SGCD	rs200476861	chr5:155,135,063-156,194,798	AX-83418775
SGCD	rs4434364	chr5:155,135,063-156,194,798	AX-35169515
SGCD	rs9313890	chr5:155,135,063-156,194,798	AX-41644471
SGCD	rs566287615	chr5:155,135,063-156,194,798	AX-151470445
SGCD	rs566287615	chr5:155,135,063-156,194,798	AX-156286168
SGCD	rs6892761	chr5:155,135,063-156,194,798	AX-35169517
SGCD	rs2055608	chr5:155,135,063-156,194,798	AX-12515647
SGCD	rs11307716	chr5:155,135,063-156,194,798	AX-14943123
SGCD	rs11750080	chr5:155,135,063-156,194,798	AX-14943124
SGCD	rs11748820	chr5:155,135,063-156,194,798	AX-151174490
SGCD	rs11748820	chr5:155,135,063-156,194,798	AX-156275623
SGCD	rs4579244	chr5:155,135,063-156,194,798	AX-148775590
SGCD	rs4579244	chr5:155,135,063-156,194,798	AX-88745718
SGCD	rs4345305	chr5:155,135,063-156,194,798	AX-14943125
SGCD	rs1394607	chr5:155,135,063-156,194,798	AX-156275624
SGCD	rs78115090	chr5:155,135,063-156,194,798	AX-14943128
SGCD	rs13172727	chr5:155,135,063-156,194,798	AX-156292777
SGCD	rs6556553	chr5:155,135,063-156,194,798	AX-156268262
SGCD	rs6556553	chr5:155,135,063-156,194,798	AX-156288097
SGCD	rs12652756	chr5:155,135,063-156,194,798	AX-156268263
SGCD	rs12652756	chr5:155,135,063-156,194,798	AX-156288098
SGCD	rs12652777	chr5:155,135,063-156,194,798	AX-96771730
SGCD	rs12652777	chr5:155,135,063-156,194,798	AX-96786770
SGCD	rs1504944	chr5:155,135,063-156,194,798	AX-151174190
SGCD	rs1504944	chr5:155,135,063-156,194,798	AX-156268264
SGCD	rs10056289	chr5:155,135,063-156,194,798	AX-14943134
SGCD	rs2135041	chr5:155,135,063-156,194,798	AX-148166057
SGCD	rs2135041	chr5:155,135,063-156,194,798	AX-156282326
SGCD	rs2174421	chr5:155,135,063-156,194,798	AX-35169535
SGCD	rs77410345	chr5:155,135,063-156,194,798	AX-14943136
SGCD	rs76574299	chr5:155,135,063-156,194,798	AX-156282327

SGCD	rs76574299	chr5:155,135,063-156,194,798	AX-156296974
SGCD	rs12153015	chr5:155,135,063-156,194,798	AX-14943137
SGCD	rs4404663	chr5:155,135,063-156,194,798	AX-156282328
SGCD	rs35935568	chr5:155,135,063-156,194,798	AX-14943138
SGCD	rs76012524	chr5:155,135,063-156,194,798	AX-14943140
SGCD	rs6872904	chr5:155,135,063-156,194,798	AX-14943141
SGCD	rs78463773	chr5:155,135,063-156,194,798	AX-14943143
SGCD	rs10037772	chr5:155,135,063-156,194,798	AX-14943146
SGCD	rs556167517	chr5:155,135,063-156,194,798	AX-151422034
SGCD	rs556167517	chr5:155,135,063-156,194,798	AX-156298122
SGCD	rs17053458	chr5:155,135,063-156,194,798	AX-41644483
SGCD	rs7447475	chr5:155,135,063-156,194,798	AX-41644485
SGCD	rs1504946	chr5:155,135,063-156,194,798	AX-14943150
SGCD	rs10059872	chr5:155,135,063-156,194,798	AX-14943152
SGCD	rs17555038	chr5:155,135,063-156,194,798	AX-14943154
SGCD	rs17053462	chr5:155,135,063-156,194,798	AX-14943155
SGCD	rs17053464	chr5:155,135,063-156,194,798	AX-14943156
SGCD	rs17053465	chr5:155,135,063-156,194,798	AX-11301518
SGCD	rs58533381	chr5:155,135,063-156,194,798	AX-120959677
SGCD	rs12514604	chr5:155,135,063-156,194,798	AX-14943157
SGCD	rs10044891	chr5:155,135,063-156,194,798	AX-41644487
SGCD	rs2036556	chr5:155,135,063-156,194,798	AX-14943159
SGCD	rs4704794	chr5:155,135,063-156,194,798	AX-14943160
SGCD	rs4094450	chr5:155,135,063-156,194,798	AX-41644489
SGCD	rs4235723	chr5:155,135,063-156,194,798	AX-11497356
SGCD	rs1394608	chr5:155,135,063-156,194,798	AX-14943164
SGCD	rs4288104	chr5:155,135,063-156,194,798	AX-35169551
SGCD	rs1394609	chr5:155,135,063-156,194,798	AX-35169553
SGCD	rs73815040	chr5:155,135,063-156,194,798	AX-14943166
SGCD	rs73815041	chr5:155,135,063-156,194,798	AX-14943167
SGCD	rs76499093	chr5:155,135,063-156,194,798	AX-14943169
SGCD	rs13182616	chr5:155,135,063-156,194,798	AX-14943172
SGCD	rs11743060	chr5:155,135,063-156,194,798	AX-156282329
SGCD	---	chr5:155,135,063-156,194,798	AX-12418262
SGCD	rs4704795	chr5:155,135,063-156,194,798	AX-14943173
SGCD	rs11135163	chr5:155,135,063-156,194,798	AX-41644493
SGCD	rs58520099	chr5:155,135,063-156,194,798	AX-14943177
SGCD	rs1394602	chr5:155,135,063-156,194,798	AX-14943178
SGCD	rs116076554	chr5:155,135,063-156,194,798	AX-35169561
SGCD	rs77552770	chr5:155,135,063-156,194,798	AX-14943179
SGCD	rs6860183	chr5:155,135,063-156,194,798	AX-156275626
SGCD	rs6860183	chr5:155,135,063-156,194,798	AX-156292778
SGCD	rs80261187	chr5:155,135,063-156,194,798	AX-156275627
SGCD	rs80261187	chr5:155,135,063-156,194,798	AX-156292779
SGCD	rs59973825	chr5:155,135,063-156,194,798	AX-14943182
SGCD	rs17053470	chr5:155,135,063-156,194,798	AX-14943183
SGCD	rs7706091	chr5:155,135,063-156,194,798	AX-11640096
SGCD	rs7714201	chr5:155,135,063-156,194,798	AX-11640625
SGCD	rs2014026	chr5:155,135,063-156,194,798	AX-35169567
SGCD	rs148532213	chr5:155,135,063-156,194,798	AX-148706530
SGCD	rs148532213	chr5:155,135,063-156,194,798	AX-156284095
SGCD	rs1813059	chr5:155,135,063-156,194,798	AX-148427468
SGCD	rs1813059	chr5:155,135,063-156,194,798	AX-148779510
SGCD	rs74435952	chr5:155,135,063-156,194,798	AX-14943185
SGCD	rs10043401	chr5:155,135,063-156,194,798	AX-147876396

SGCD	rs10043401	chr5:155,135,063-156,194,798	AX-147970522
SGCD	rs72798928	chr5:155,135,063-156,194,798	AX-35169569
SGCD	rs76024207	chr5:155,135,063-156,194,798	AX-35169571
SGCD	rs4068396	chr5:155,135,063-156,194,798	AX-14943188
SGCD	rs147014804	chr5:155,135,063-156,194,798	AX-121317687
SGCD	rs147014804	chr5:155,135,063-156,194,798	AX-156284899
SGCD	rs9313897	chr5:155,135,063-156,194,798	AX-14943189
SGCD	rs73815049	chr5:155,135,063-156,194,798	AX-35169573
SGCD	rs10036928	chr5:155,135,063-156,194,798	AX-156268266
SGCD	rs10036928	chr5:155,135,063-156,194,798	AX-156288100
SGCD	rs17636636	chr5:155,135,063-156,194,798	AX-11332445
SGCD	rs80087683	chr5:155,135,063-156,194,798	AX-14943191
SGCD	rs6889255	chr5:155,135,063-156,194,798	AX-148446811
SGCD	rs6889255	chr5:155,135,063-156,194,798	AX-156268267
SGCD	rs6873000	chr5:155,135,063-156,194,798	AX-11591303
SGCD	rs77327847	chr5:155,135,063-156,194,798	AX-14943192
SGCD	rs17053477	chr5:155,135,063-156,194,798	AX-147804987
SGCD	rs17053477	chr5:155,135,063-156,194,798	AX-147898948
SGCD	rs17555767	chr5:155,135,063-156,194,798	AX-14943194
SGCD	rs147504894	chr5:155,135,063-156,194,798	AX-122584376
SGCD	rs35804717	chr5:155,135,063-156,194,798	AX-156282330
SGCD	rs35804717	chr5:155,135,063-156,194,798	AX-156296977
SGCD	rs112151431	chr5:155,135,063-156,194,798	AX-156282331
SGCD	rs112151431	chr5:155,135,063-156,194,798	AX-156296978
SGCD	rs71935610	chr5:155,135,063-156,194,798	AX-151169750
SGCD	rs71935610	chr5:155,135,063-156,194,798	AX-156286229
SGCD	rs10038418	chr5:155,135,063-156,194,798	AX-156292780
SGCD	rs12523127	chr5:155,135,063-156,194,798	AX-41644509
SGCD	rs114294828	chr5:155,135,063-156,194,798	AX-156275629
SGCD	rs7700863	chr5:155,135,063-156,194,798	AX-156275630
SGCD	rs76524654	chr5:155,135,063-156,194,798	AX-14943198
SGCD	rs11741970	chr5:155,135,063-156,194,798	AX-11172003
SGCD	rs138548425	chr5:155,135,063-156,194,798	AX-121816101
SGCD	rs17053484	chr5:155,135,063-156,194,798	AX-14943199
SGCD	rs12514194	chr5:155,135,063-156,194,798	AX-41644517
SGCD	rs61603347	chr5:155,135,063-156,194,798	AX-148157880
SGCD	rs61603347	chr5:155,135,063-156,194,798	AX-156284096
SGCD	rs72798934	chr5:155,135,063-156,194,798	AX-148409332
SGCD	rs72798934	chr5:155,135,063-156,194,798	AX-156275631
SGCD	rs77946635	chr5:155,135,063-156,194,798	AX-14943204
SGCD	rs1504926	chr5:155,135,063-156,194,798	AX-12466786
SGCD	rs6866394	chr5:155,135,063-156,194,798	AX-12612461
SGCD	rs7722282	chr5:155,135,063-156,194,798	AX-14943206
SGCD	rs7722398	chr5:155,135,063-156,194,798	AX-14943207
SGCD	rs75894030	chr5:155,135,063-156,194,798	AX-14943209
SGCD	rs73812207	chr5:155,135,063-156,194,798	AX-14943210
SGCD	rs17637007	chr5:155,135,063-156,194,798	AX-11332460
SGCD	rs17053491	chr5:155,135,063-156,194,798	AX-41644523
SGCD	rs79448192	chr5:155,135,063-156,194,798	AX-14943215
SGCD	rs7732608	chr5:155,135,063-156,194,798	AX-147793551
SGCD	rs7732608	chr5:155,135,063-156,194,798	AX-147887435
SGCD	rs7721793	chr5:155,135,063-156,194,798	AX-12638158
SGCD	rs73812209	chr5:155,135,063-156,194,798	AX-14943219
SGCD	rs17053495	chr5:155,135,063-156,194,798	AX-35169599
SGCD	rs17053497	chr5:155,135,063-156,194,798	AX-41644527

SGCD	rs9313900	chr5:155,135,063-156,194,798	AX-41644529
SGCD	rs13184996	chr5:155,135,063-156,194,798	AX-14943223
SGCD	rs72798948	chr5:155,135,063-156,194,798	AX-14943224
SGCD	rs7725840	chr5:155,135,063-156,194,798	AX-41644531
SGCD	rs113196974	chr5:155,135,063-156,194,798	AX-14943226
SGCD	rs115674853	chr5:155,135,063-156,194,798	AX-156275632
SGCD	rs115674853	chr5:155,135,063-156,194,798	AX-156292783
SGCD	rs13355607	chr5:155,135,063-156,194,798	AX-156275633
SGCD	rs13355607	chr5:155,135,063-156,194,798	AX-92528617
SGCD	rs1967228	chr5:155,135,063-156,194,798	AX-35169605
SGCD	rs144249075	chr5:155,135,063-156,194,798	AX-122490735
SGCD	rs144249075	chr5:155,135,063-156,194,798	AX-156284838
SGCD	rs75722607	chr5:155,135,063-156,194,798	AX-35169607
SGCD	rs970476	chr5:155,135,063-156,194,798	AX-11700926
SGCD	rs75440809	chr5:155,135,063-156,194,798	AX-148860636
SGCD	rs75440809	chr5:155,135,063-156,194,798	AX-156275634
SGCD	rs970475	chr5:155,135,063-156,194,798	AX-35169611
SGCD	rs72798952	chr5:155,135,063-156,194,798	AX-14943227
SGCD	rs78159313	chr5:155,135,063-156,194,798	AX-156275635
SGCD	rs78159313	chr5:155,135,063-156,194,798	AX-156292784
SGCD	rs60618824	chr5:155,135,063-156,194,798	AX-35169613
SGCD	rs1021903	chr5:155,135,063-156,194,798	AX-156275636
SGCD	rs1021903	chr5:155,135,063-156,194,798	AX-156292785
SGCD	rs1875963	chr5:155,135,063-156,194,798	AX-14943233
SGCD	rs76717701	chr5:155,135,063-156,194,798	AX-14943234
SGCD	rs1504927	chr5:155,135,063-156,194,798	AX-14943235
SGCD	rs2055609	chr5:155,135,063-156,194,798	AX-12515648
SGCD	rs34201599	chr5:155,135,063-156,194,798	AX-35169617
SGCD	rs10062636	chr5:155,135,063-156,194,798	AX-41644535
SGCD	rs3969758	chr5:155,135,063-156,194,798	AX-41644537
SGCD	rs2312062	chr5:155,135,063-156,194,798	AX-35169625
SGCD	rs2312063	chr5:155,135,063-156,194,798	AX-14943238
SGCD	rs58394898	chr5:155,135,063-156,194,798	AX-14943239
SGCD	rs80013625	chr5:155,135,063-156,194,798	AX-156275637
SGCD	rs80013625	chr5:155,135,063-156,194,798	AX-156292786
SGCD	rs77234766	chr5:155,135,063-156,194,798	AX-35169631
SGCD	rs2312064	chr5:155,135,063-156,194,798	AX-14943243
SGCD	rs1472756	chr5:155,135,063-156,194,798	AX-14943244
SGCD	rs12521900	chr5:155,135,063-156,194,798	AX-41644539
SGCD	rs12521918	chr5:155,135,063-156,194,798	AX-14943245
SGCD	rs75672801	chr5:155,135,063-156,194,798	AX-14943246
SGCD	rs11289380	chr5:155,135,063-156,194,798	AX-151256440
SGCD	rs7737163	chr5:155,135,063-156,194,798	AX-35169633
SGCD	rs7737609	chr5:155,135,063-156,194,798	AX-14943252
SGCD	rs7715464	chr5:155,135,063-156,194,798	AX-14943253
SGCD	rs10475702	chr5:155,135,063-156,194,798	AX-41644545
SGCD	rs6880774	chr5:155,135,063-156,194,798	AX-12612972
SGCD	rs17053509	chr5:155,135,063-156,194,798	AX-156275638
SGCD	rs17053509	chr5:155,135,063-156,194,798	AX-156292787
SGCD	rs2135026	chr5:155,135,063-156,194,798	AX-148301480
SGCD	rs2135026	chr5:155,135,063-156,194,798	AX-156275639
SGCD	rs2135027	chr5:155,135,063-156,194,798	AX-148446975
SGCD	rs2135027	chr5:155,135,063-156,194,798	AX-156275640
SGCD	rs59512108	chr5:155,135,063-156,194,798	AX-148746216
SGCD	rs59512108	chr5:155,135,063-156,194,798	AX-156275641

SGCD	rs931798	chr5:155,135,063-156,194,798	AX-14943255
SGCD	rs140617	chr5:155,135,063-156,194,798	AX-11260541
SGCD	rs140616	chr5:155,135,063-156,194,798	AX-147938499
SGCD	rs140615	chr5:155,135,063-156,194,798	AX-14943256
SGCD	rs111699907	chr5:155,135,063-156,194,798	AX-119533275
SGCD	rs1394603	chr5:155,135,063-156,194,798	AX-41644555
SGCD	rs35129841	chr5:155,135,063-156,194,798	AX-41644557
SGCD	rs6556570	chr5:155,135,063-156,194,798	AX-14943261
SGCD	rs10476369	chr5:155,135,063-156,194,798	AX-41644561
SGCD	rs1983099	chr5:155,135,063-156,194,798	AX-11357560
SGCD	rs11960521	chr5:155,135,063-156,194,798	AX-41644563
SGCD	rs7723660	chr5:155,135,063-156,194,798	AX-14943263
SGCD	rs62380724	chr5:155,135,063-156,194,798	AX-14943264
SGCD	rs924872	chr5:155,135,063-156,194,798	AX-14943265
SGCD	rs4438860	chr5:155,135,063-156,194,798	AX-41644567
SGCD	rs905789	chr5:155,135,063-156,194,798	AX-156275642
SGCD	rs905789	chr5:155,135,063-156,194,798	AX-156292788
SGCD	rs6556572	chr5:155,135,063-156,194,798	AX-35169643
SGCD	rs73812224	chr5:155,135,063-156,194,798	AX-14943266
SGCD	rs6556573	chr5:155,135,063-156,194,798	AX-14943267
SGCD	rs6556574	chr5:155,135,063-156,194,798	AX-35169645
SGCD	rs1553534	chr5:155,135,063-156,194,798	AX-11274825
SGCD	rs9313905	chr5:155,135,063-156,194,798	AX-41644571
SGCD	rs7708513	chr5:155,135,063-156,194,798	AX-14943271
SGCD	rs1553535	chr5:155,135,063-156,194,798	AX-35169649
SGCD	rs79005184	chr5:155,135,063-156,194,798	AX-14943272
SGCD	rs62380725	chr5:155,135,063-156,194,798	AX-14943273
SGCD	rs10476372	chr5:155,135,063-156,194,798	AX-41644573
SGCD	rs77214147	chr5:155,135,063-156,194,798	AX-14943275
SGCD	rs55669320	chr5:155,135,063-156,194,798	AX-50448389
SGCD	rs4704798	chr5:155,135,063-156,194,798	AX-14943276
SGCD	rs1059020	chr5:155,135,063-156,194,798	AX-35169651
SGCD	rs77038602	chr5:155,135,063-156,194,798	AX-148884304
SGCD	rs10476373	chr5:155,135,063-156,194,798	AX-147984250
SGCD	rs10476373	chr5:155,135,063-156,194,798	AX-153799259
SGCD	rs10476374	chr5:155,135,063-156,194,798	AX-156275643
SGCD	rs79019903	chr5:155,135,063-156,194,798	AX-156275644
SGCD	rs79019903	chr5:155,135,063-156,194,798	AX-156292789
SGCD	rs77871929	chr5:155,135,063-156,194,798	AX-156275645
SGCD	rs77871929	chr5:155,135,063-156,194,798	AX-156292790
SGCD	rs6556578	chr5:155,135,063-156,194,798	AX-156275646
SGCD	rs6556578	chr5:155,135,063-156,194,798	AX-156292791
SGCD	rs73293248	chr5:155,135,063-156,194,798	AX-14943278
SGCD	rs6884659	chr5:155,135,063-156,194,798	AX-148316911
SGCD	rs6884659	chr5:155,135,063-156,194,798	AX-156275647
SGCD	rs10780121	chr5:155,135,063-156,194,798	AX-156275648
SGCD	rs62380727	chr5:155,135,063-156,194,798	AX-35169663
SGCD	rs11743065	chr5:155,135,063-156,194,798	AX-14943279
SGCD	rs6862189	chr5:155,135,063-156,194,798	AX-14943280
SGCD	rs10036164	chr5:155,135,063-156,194,798	AX-156275650
SGCD	rs10036164	chr5:155,135,063-156,194,798	AX-156292794
SGCD	rs1472755	chr5:155,135,063-156,194,798	AX-156275651
SGCD	rs1472755	chr5:155,135,063-156,194,798	AX-156292795
SGCD	rs114027505	chr5:155,135,063-156,194,798	AX-148768610
SGCD	rs114027505	chr5:155,135,063-156,194,798	AX-156268268

SGCD	rs4704799	chr5:155,135,063-156,194,798	AX-148031149
SGCD	rs4704799	chr5:155,135,063-156,194,798	AX-156268269
SGCD	rs17053531	chr5:155,135,063-156,194,798	AX-41644581
SGCD	rs62380728	chr5:155,135,063-156,194,798	AX-35169679
SGCD	rs62380729	chr5:155,135,063-156,194,798	AX-35169681
SGCD	rs62380730	chr5:155,135,063-156,194,798	AX-35169683
SGCD	rs10475703	chr5:155,135,063-156,194,798	AX-14943284
SGCD	rs11960866	chr5:155,135,063-156,194,798	AX-41644583
SGCD	rs75708568	chr5:155,135,063-156,194,798	AX-14943286
SGCD	rs62380731	chr5:155,135,063-156,194,798	AX-14943288
SGCD	rs4704800	chr5:155,135,063-156,194,798	AX-156288101
SGCD	rs73812228	chr5:155,135,063-156,194,798	AX-14943291
SGCD	rs6869537	chr5:155,135,063-156,194,798	AX-156282332
SGCD	rs6869537	chr5:155,135,063-156,194,798	AX-156296979
SGCD	rs28405992	chr5:155,135,063-156,194,798	AX-41644591
SGCD	rs4705012	chr5:155,135,063-156,194,798	AX-12578514
SGCD	rs4704801	chr5:155,135,063-156,194,798	AX-41644595
SGCD	rs62380732	chr5:155,135,063-156,194,798	AX-14943295
SGCD	rs55755417	chr5:155,135,063-156,194,798	AX-14943296
SGCD	rs11743557	chr5:155,135,063-156,194,798	AX-11172119
SGCD	rs4454042	chr5:155,135,063-156,194,798	AX-12572532
SGCD	rs62380750	chr5:155,135,063-156,194,798	AX-14943299
SGCD	rs10054859	chr5:155,135,063-156,194,798	AX-11089880
SGCD	rs62380751	chr5:155,135,063-156,194,798	AX-156292797
SGCD	rs9313909	chr5:155,135,063-156,194,798	AX-11680218
SGCD	rs2135028	chr5:155,135,063-156,194,798	AX-12518368
SGCD	rs73293268	chr5:155,135,063-156,194,798	AX-14943303
SGCD	rs6865531	chr5:155,135,063-156,194,798	AX-14943304
SGCD	rs62380752	chr5:155,135,063-156,194,798	AX-14943305
SGCD	rs12716390	chr5:155,135,063-156,194,798	AX-12446247
SGCD	rs62380753	chr5:155,135,063-156,194,798	AX-14943307
SGCD	rs9313910	chr5:155,135,063-156,194,798	AX-14943308
SGCD	rs10043022	chr5:155,135,063-156,194,798	AX-35169697
SGCD	rs13185401	chr5:155,135,063-156,194,798	AX-156275654
SGCD	rs62380754	chr5:155,135,063-156,194,798	AX-35169701
SGCD	rs56709418	chr5:155,135,063-156,194,798	AX-14943311
SGCD	rs62380756	chr5:155,135,063-156,194,798	AX-14943314
SGCD	rs12054876	chr5:155,135,063-156,194,798	AX-14943318
SGCD	rs2017982	chr5:155,135,063-156,194,798	AX-148373102
SGCD	rs2017982	chr5:155,135,063-156,194,798	AX-156282333
SGCD	rs10062440	chr5:155,135,063-156,194,798	AX-35169709
SGCD	rs4705013	chr5:155,135,063-156,194,798	AX-11519470
SGCD	rs6881353	chr5:155,135,063-156,194,798	AX-35169711
SGCD	rs10515736	chr5:155,135,063-156,194,798	AX-35169715
SGCD	rs79374679	chr5:155,135,063-156,194,798	AX-14943323
SGCD	rs11296545	chr5:155,135,063-156,194,798	AX-11154850
SGCD	rs7731517	chr5:155,135,063-156,194,798	AX-14943325
SGCD	rs7731883	chr5:155,135,063-156,194,798	AX-11641737
SGCD	rs113841326	chr5:155,135,063-156,194,798	AX-105145308
SGCD	rs113841326	chr5:155,135,063-156,194,798	AX-92499059
SGCD	rs1504928	chr5:155,135,063-156,194,798	AX-35169719
SGCD	rs61422788	chr5:155,135,063-156,194,798	AX-14943328
SGCD	rs6886901	chr5:155,135,063-156,194,798	AX-156275656
SGCD	rs6886901	chr5:155,135,063-156,194,798	AX-156292798
SGCD	rs76446055	chr5:155,135,063-156,194,798	AX-156275657

SGCD	rs76446055	chr5:155,135,063-156,194,798	AX-156292799
SGCD	rs6879264	chr5:155,135,063-156,194,798	AX-156275658
SGCD	rs6879264	chr5:155,135,063-156,194,798	AX-156292800
SGCD	rs6879407	chr5:155,135,063-156,194,798	AX-12612930
SGCD	rs9313916	chr5:155,135,063-156,194,798	AX-156268271
SGCD	rs9313916	chr5:155,135,063-156,194,798	AX-156288102
SGCD	rs1827687	chr5:155,135,063-156,194,798	AX-148290680
SGCD	rs1827687	chr5:155,135,063-156,194,798	AX-156268272
SGCD	rs2055610	chr5:155,135,063-156,194,798	AX-41644613
SGCD	rs139072272	chr5:155,135,063-156,194,798	AX-113496746
SGCD	---	chr5:155,135,063-156,194,798	AX-151272390
SGCD	---	chr5:155,135,063-156,194,798	AX-151185966
SGCD	rs2036557	chr5:155,135,063-156,194,798	AX-50448398
SGCD	rs7710094	chr5:155,135,063-156,194,798	AX-86902545
SGCD	rs56371636	chr5:155,135,063-156,194,798	AX-14943336
SGCD	rs6556587	chr5:155,135,063-156,194,798	AX-156282334
SGCD	rs6556587	chr5:155,135,063-156,194,798	AX-156296980
SGCD	rs35414747	chr5:155,135,063-156,194,798	AX-156282335
SGCD	rs35414747	chr5:155,135,063-156,194,798	AX-156296981
SGCD	rs1910781	chr5:155,135,063-156,194,798	AX-148280325
SGCD	rs1910781	chr5:155,135,063-156,194,798	AX-148354556
SGCD	rs7727771	chr5:155,135,063-156,194,798	AX-147980675
SGCD	rs7727771	chr5:155,135,063-156,194,798	AX-153796096
SGCD	rs6872627	chr5:155,135,063-156,194,798	AX-35169737
SGCD	rs6896669	chr5:155,135,063-156,194,798	AX-14943341
SGCD	rs10042626	chr5:155,135,063-156,194,798	AX-148010404
SGCD	rs7729019	chr5:155,135,063-156,194,798	AX-41644621
SGCD	rs57792332	chr5:155,135,063-156,194,798	AX-14943343
SGCD	rs57620638	chr5:155,135,063-156,194,798	AX-14943344
SGCD	rs56347407	chr5:155,135,063-156,194,798	AX-14943346
SGCD	rs75451933	chr5:155,135,063-156,194,798	AX-14943347
SGCD	rs6894759	chr5:155,135,063-156,194,798	AX-14943348
SGCD	rs73812240	chr5:155,135,063-156,194,798	AX-35169745
SGCD	rs6556594	chr5:155,135,063-156,194,798	AX-14943350
SGCD	rs7718903	chr5:155,135,063-156,194,798	AX-14943352
SGCD	rs56132359	chr5:155,135,063-156,194,798	AX-156282336
SGCD	rs56132359	chr5:155,135,063-156,194,798	AX-156296982
SGCD	rs2174417	chr5:155,135,063-156,194,798	AX-14943355
SGCD	rs12163943	chr5:155,135,063-156,194,798	AX-35169751
SGCD	rs7729818	chr5:155,135,063-156,194,798	AX-41644627
SGCD	rs10476390	chr5:155,135,063-156,194,798	AX-14943358
SGCD	rs68077272	chr5:155,135,063-156,194,798	AX-151169942
SGCD	rs68077272	chr5:155,135,063-156,194,798	AX-156285730
SGCD	rs185598356	chr5:155,135,063-156,194,798	AX-151323255
SGCD	rs185598356	chr5:155,135,063-156,194,798	AX-156284097
SGCD	rs181901232	chr5:155,135,063-156,194,798	AX-151344771
SGCD	rs181901232	chr5:155,135,063-156,194,798	AX-156284098
SGCD	rs200906259	chr5:155,135,063-156,194,798	AX-156284994
SGCD	rs186925058	chr5:155,135,063-156,194,798	AX-151316880
SGCD	rs186925058	chr5:155,135,063-156,194,798	AX-156284099
SGCD	rs13357986	chr5:155,135,063-156,194,798	AX-151272485
SGCD	rs13357986	chr5:155,135,063-156,194,798	AX-156284100
SGCD	rs12163970	chr5:155,135,063-156,194,798	AX-119976927
SGCD	rs11135199	chr5:155,135,063-156,194,798	AX-156284101
SGCD	rs7448162	chr5:155,135,063-156,194,798	AX-120253339

SGCD	rs7448162	chr5:155,135,063-156,194,798	AX-151115759
SGCD	---	chr5:155,135,063-156,194,798	AX-156288103
SGCD	rs4502803	chr5:155,135,063-156,194,798	AX-14943359
SGCD	rs11135200	chr5:155,135,063-156,194,798	AX-156268274
SGCD	rs11135200	chr5:155,135,063-156,194,798	AX-156288104
SGCD	rs11135202	chr5:155,135,063-156,194,798	AX-14943360
SGCD	rs1504929	chr5:155,135,063-156,194,798	AX-12466787
SGCD	rs1504930	chr5:155,135,063-156,194,798	AX-120356631
SGCD	rs1504930	chr5:155,135,063-156,194,798	AX-156288105
SGCD	rs4705014	chr5:155,135,063-156,194,798	AX-14943364
SGCD	rs6556603	chr5:155,135,063-156,194,798	AX-156268275
SGCD	rs6556603	chr5:155,135,063-156,194,798	AX-156288106
SGCD	rs1604957	chr5:155,135,063-156,194,798	AX-156288107
SGCD	rs79374913	chr5:155,135,063-156,194,798	AX-14943367
SGCD	rs10063250	chr5:155,135,063-156,194,798	AX-11090381
SGCD	rs10038124	chr5:155,135,063-156,194,798	AX-148274620
SGCD	rs10038124	chr5:155,135,063-156,194,798	AX-156268277
SGCD	rs57757758	chr5:155,135,063-156,194,798	AX-151215741
SGCD	rs57757758	chr5:155,135,063-156,194,798	AX-156268278
SGCD	rs112828647	chr5:155,135,063-156,194,798	AX-14943369
SGCD	rs1604958	chr5:155,135,063-156,194,798	AX-156268279
SGCD	rs1604958	chr5:155,135,063-156,194,798	AX-156288108
SGCD	rs78674996	chr5:155,135,063-156,194,798	AX-14943370
SGCD	rs2135029	chr5:155,135,063-156,194,798	AX-11369551
SGCD	rs6556609	chr5:155,135,063-156,194,798	AX-14943372
SGCD	rs6556610	chr5:155,135,063-156,194,798	AX-156282337
SGCD	rs6556612	chr5:155,135,063-156,194,798	AX-156268281
SGCD	rs78269284	chr5:155,135,063-156,194,798	AX-14943375
SGCD	rs1587857	chr5:155,135,063-156,194,798	AX-156288111
SGCD	rs867009	chr5:155,135,063-156,194,798	AX-35169779
SGCD	rs116064669	chr5:155,135,063-156,194,798	AX-148762330
SGCD	rs116064669	chr5:155,135,063-156,194,798	AX-156268283
SGCD	rs6556615	chr5:155,135,063-156,194,798	AX-11571769
SGCD	rs62382689	chr5:155,135,063-156,194,798	AX-14943383
SGCD	---	chr5:155,135,063-156,194,798	AX-38254299
SGCD	rs905788	chr5:155,135,063-156,194,798	AX-147865460
SGCD	rs905788	chr5:155,135,063-156,194,798	AX-147961257
SGCD	rs144172041	chr5:155,135,063-156,194,798	AX-14943387
SGCD	rs144172041	chr5:155,135,063-156,194,798	AX-156297841
SGCD	rs6881542	chr5:155,135,063-156,194,798	AX-14943389
SGCD	rs2055611	chr5:155,135,063-156,194,798	AX-14943390
SGCD	rs1504931	chr5:155,135,063-156,194,798	AX-11269874
SGCD	rs2055612	chr5:155,135,063-156,194,798	AX-14943391
SGCD	rs74986115	chr5:155,135,063-156,194,798	AX-14943392
SGCD	rs17053557	chr5:155,135,063-156,194,798	AX-12487900
SGCD	rs6880816	chr5:155,135,063-156,194,798	AX-12612974
SGCD	rs13189810	chr5:155,135,063-156,194,798	AX-35169791
SGCD	rs7737328	chr5:155,135,063-156,194,798	AX-12638723
SGCD	rs6556623	chr5:155,135,063-156,194,798	AX-14943398
SGCD	rs6556624	chr5:155,135,063-156,194,798	AX-14943400
SGCD	rs6556625	chr5:155,135,063-156,194,798	AX-11571771
SGCD	rs4705015	chr5:155,135,063-156,194,798	AX-35169795
SGCD	rs4705017	chr5:155,135,063-156,194,798	AX-14943403
SGCD	rs2312182	chr5:155,135,063-156,194,798	AX-12525661
SGCD	rs75105227	chr5:155,135,063-156,194,798	AX-38254305

SGCD	rs10054228	chr5:155,135,063-156,194,798	AX-92722698
SGCD	rs1504933	chr5:155,135,063-156,194,798	AX-12466788
SGCD	rs75493479	chr5:155,135,063-156,194,798	AX-14943417
SGCD	rs1504934	chr5:155,135,063-156,194,798	AX-41644657
SGCD	rs1504935	chr5:155,135,063-156,194,798	AX-12466789
SGCD	rs10078675	chr5:155,135,063-156,194,798	AX-35169815
SGCD	rs62382691	chr5:155,135,063-156,194,798	AX-14943433
SGCD	rs6863243	chr5:155,135,063-156,194,798	AX-14943434
SGCD	rs6883722	chr5:155,135,063-156,194,798	AX-11591958
SGCD	rs12652669	chr5:155,135,063-156,194,798	AX-11220942
SGCD	rs62382692	chr5:155,135,063-156,194,798	AX-14943437
SGCD	rs12659033	chr5:155,135,063-156,194,798	AX-35169817
SGCD	rs10052627	chr5:155,135,063-156,194,798	AX-148833852
SGCD	rs10052627	chr5:155,135,063-156,194,798	AX-156275660
SGCD	rs7718439	chr5:155,135,063-156,194,798	AX-148492936
SGCD	rs17053564	chr5:155,135,063-156,194,798	AX-156275661
SGCD	rs17053564	chr5:155,135,063-156,194,798	AX-156292803
SGCD	rs74843411	chr5:155,135,063-156,194,798	AX-113251381
SGCD	rs74843411	chr5:155,135,063-156,194,798	AX-156275662
SGCD	rs57683078	chr5:155,135,063-156,194,798	AX-14943443
SGCD	rs17052633	chr5:155,135,063-156,194,798	AX-11301450
SGCD	rs62382694	chr5:155,135,063-156,194,798	AX-14943446
SGCD	rs140625177	chr5:155,135,063-156,194,798	AX-14943447
SGCD	rs140625177	chr5:155,135,063-156,194,798	AX-156284476
SGCD	rs62382695	chr5:155,135,063-156,194,798	AX-14943453
SGCD	rs10476415	chr5:155,135,063-156,194,798	AX-41644673
SGCD	rs11135226	chr5:155,135,063-156,194,798	AX-11146232
SGCD	rs13162852	chr5:155,135,063-156,194,798	AX-35169829
SGCD	rs77020940	chr5:155,135,063-156,194,798	AX-14943466
SGCD	rs117639292	chr5:155,135,063-156,194,798	AX-14943467
SGCD	rs983467	chr5:155,135,063-156,194,798	AX-11705004
SGCD	rs77238449	chr5:155,135,063-156,194,798	AX-14943471
SGCD	rs149406899	chr5:155,135,063-156,194,798	AX-122389193
SGCD	rs149406899	chr5:155,135,063-156,194,798	AX-156297714
SGCD	rs7722422	chr5:155,135,063-156,194,798	AX-148762253
SGCD	rs7722422	chr5:155,135,063-156,194,798	AX-156275663
SGCD	rs7717944	chr5:155,135,063-156,194,798	AX-122901097
SGCD	rs7717944	chr5:155,135,063-156,194,798	AX-148055056
SGCD	---	chr5:155,135,063-156,194,798	AX-148014241
SGCD	---	chr5:155,135,063-156,194,798	AX-153825523
SGCD	rs113636560	chr5:155,135,063-156,194,798	AX-156275664
SGCD	rs17053572	chr5:155,135,063-156,194,798	AX-14943487
SGCD	rs7719992	chr5:155,135,063-156,194,798	AX-151145850
SGCD	rs112190759	chr5:155,135,063-156,194,798	AX-148734655
SGCD	rs112190759	chr5:155,135,063-156,194,798	AX-156275666
SGCD	rs9313941	chr5:155,135,063-156,194,798	AX-11680219
SGCD	rs7720922	chr5:155,135,063-156,194,798	AX-156275667
SGCD	rs7720922	chr5:155,135,063-156,194,798	AX-156292804
SGCD	rs7721082	chr5:155,135,063-156,194,798	AX-41644683
SGCD	rs55820495	chr5:155,135,063-156,194,798	AX-147982496
SGCD	rs55820495	chr5:155,135,063-156,194,798	AX-153797688
SGCD	rs1504936	chr5:155,135,063-156,194,798	AX-41644687
SGCD	rs2312183	chr5:155,135,063-156,194,798	AX-14943503
SGCD	rs2135030	chr5:155,135,063-156,194,798	AX-14943504
SGCD	rs55872084	chr5:155,135,063-156,194,798	AX-14943505

SGCD	rs73295175	chr5:155,135,063-156,194,798	AX-14943506
SGCD	rs4704803	chr5:155,135,063-156,194,798	AX-41644691
SGCD	rs1354563	chr5:155,135,063-156,194,798	AX-156275668
SGCD	rs1354563	chr5:155,135,063-156,194,798	AX-156292805
SGCD	rs1354564	chr5:155,135,063-156,194,798	AX-41644695
SGCD	rs111703649	chr5:155,135,063-156,194,798	AX-148850809
SGCD	rs111703649	chr5:155,135,063-156,194,798	AX-156284102
SGCD	rs2135031	chr5:155,135,063-156,194,798	AX-151131603
SGCD	rs2135031	chr5:155,135,063-156,194,798	AX-156284103
SGCD	rs13359664	chr5:155,135,063-156,194,798	AX-151421355
SGCD	rs13359664	chr5:155,135,063-156,194,798	AX-156284104
SGCD	rs4704804	chr5:155,135,063-156,194,798	AX-12578506
SGCD	rs980149	chr5:155,135,063-156,194,798	AX-11702882
SGCD	rs980148	chr5:155,135,063-156,194,798	AX-14943522
SGCD	rs17638584	chr5:155,135,063-156,194,798	AX-11332542
SGCD	rs115180142	chr5:155,135,063-156,194,798	AX-14943527
SGCD	rs11304197	chr5:155,135,063-156,194,798	AX-107817695
SGCD	rs11304197	chr5:155,135,063-156,194,798	AX-156275669
SGCD	rs2055613	chr5:155,135,063-156,194,798	AX-11363729
SGCD	rs2312184	chr5:155,135,063-156,194,798	AX-148165173
SGCD	rs2312184	chr5:155,135,063-156,194,798	AX-156275670
SGCD	rs61068059	chr5:155,135,063-156,194,798	AX-156284105
SGCD	rs61068059	chr5:155,135,063-156,194,798	AX-86896730
SGCD	rs2174419	chr5:155,135,063-156,194,798	AX-156292806
SGCD	rs4705018	chr5:155,135,063-156,194,798	AX-11519471
SGCD	rs4307078	chr5:155,135,063-156,194,798	AX-11500512
SGCD	rs11949028	chr5:155,135,063-156,194,798	AX-151230678
SGCD	rs10866739	chr5:155,135,063-156,194,798	AX-35169889
SGCD	rs34285044	chr5:155,135,063-156,194,798	AX-151290200
SGCD	rs34285044	chr5:155,135,063-156,194,798	AX-156286007
SGCD	rs256846	chr5:155,135,063-156,194,798	AX-14943540
SGCD	rs256845	chr5:155,135,063-156,194,798	AX-156275672
SGCD	rs7720984	chr5:155,135,063-156,194,798	AX-11641068
SGCD	rs256844	chr5:155,135,063-156,194,798	AX-14943549
SGCD	rs256843	chr5:155,135,063-156,194,798	AX-14943550
SGCD	rs1368300	chr5:155,135,063-156,194,798	AX-14943552
SGCD	rs256842	chr5:155,135,063-156,194,798	AX-11398876
SGCD	rs256841	chr5:155,135,063-156,194,798	AX-11398874
SGCD	rs6886828	chr5:155,135,063-156,194,798	AX-11592156
SGCD	rs256840	chr5:155,135,063-156,194,798	AX-14943555
SGCD	---	chr5:155,135,063-156,194,798	AX-151251691
SGCD	---	chr5:155,135,063-156,194,798	AX-156275673
SGCD	rs11738716	chr5:155,135,063-156,194,798	AX-14943560
SGCD	rs116566991	chr5:155,135,063-156,194,798	AX-14943561
SGCD	rs34567151	chr5:155,135,063-156,194,798	AX-120577939
SGCD	rs34567151	chr5:155,135,063-156,194,798	AX-156284212
SGCD	rs256821	chr5:155,135,063-156,194,798	AX-12532129
SGCD	rs256820	chr5:155,135,063-156,194,798	AX-156282339
SGCD	rs256820	chr5:155,135,063-156,194,798	AX-156296983
SGCD	rs6880345	chr5:155,135,063-156,194,798	AX-156282340
SGCD	rs6880345	chr5:155,135,063-156,194,798	AX-156296984
SGCD	rs256819	chr5:155,135,063-156,194,798	AX-156275674
SGCD	rs256819	chr5:155,135,063-156,194,798	AX-156292808
SGCD	rs73297105	chr5:155,135,063-156,194,798	AX-14943578
SGCD	rs4588577	chr5:155,135,063-156,194,798	AX-156292809

SGCD	rs4613690	chr5:155,135,063-156,194,798	AX-156275676
SGCD	rs4613690	chr5:155,135,063-156,194,798	AX-156292810
SGCD	rs1835912	chr5:155,135,063-156,194,798	AX-156275678
SGCD	rs1835912	chr5:155,135,063-156,194,798	AX-156292812
SGCD	rs9313957	chr5:155,135,063-156,194,798	AX-41644723
SGCD	rs256822	chr5:155,135,063-156,194,798	AX-147799063
SGCD	rs256822	chr5:155,135,063-156,194,798	AX-147892957
SGCD	rs256823	chr5:155,135,063-156,194,798	AX-14943582
SGCD	rs78854340	chr5:155,135,063-156,194,798	AX-35169911
SGCD	rs256824	chr5:155,135,063-156,194,798	AX-14943587
SGCD	rs256825	chr5:155,135,063-156,194,798	AX-14943592
SGCD	---	chr5:155,135,063-156,194,798	AX-86664935
SGCD	rs74846539	chr5:155,135,063-156,194,798	AX-83465691
SGCD	rs45559835	chr5:155,135,063-156,194,798	AX-11510984
SGCD	rs256826	chr5:155,135,063-156,194,798	AX-12532131
SGCD	rs934417	chr5:155,135,063-156,194,798	AX-12657259
SGCD	rs13360574	chr5:155,135,063-156,194,798	AX-11251212
SGCD	rs78118273	chr5:155,135,063-156,194,798	AX-35169917
SGCD	rs256839	chr5:155,135,063-156,194,798	AX-14943603
SGCD	rs183745	chr5:155,135,063-156,194,798	AX-11346362
SGCD	rs1368299	chr5:155,135,063-156,194,798	AX-12460032
SGCD	rs256838	chr5:155,135,063-156,194,798	AX-156275679
SGCD	rs256838	chr5:155,135,063-156,194,798	AX-156292813
SGCD	rs891907	chr5:155,135,063-156,194,798	AX-14943612
SGCD	rs891906	chr5:155,135,063-156,194,798	AX-12653932
SGCD	rs6883496	chr5:155,135,063-156,194,798	AX-14943614
SGCD	rs256837	chr5:155,135,063-156,194,798	AX-113251383
SGCD	rs256837	chr5:155,135,063-156,194,798	AX-156292814
SGCD	rs17639279	chr5:155,135,063-156,194,798	AX-156275680
SGCD	rs17639279	chr5:155,135,063-156,194,798	AX-156292815
SGCD	rs73812681	chr5:155,135,063-156,194,798	AX-14943615
SGCD	rs256836	chr5:155,135,063-156,194,798	AX-11398870
SGCD	rs73297112	chr5:155,135,063-156,194,798	AX-14943618
SGCD	rs77542985	chr5:155,135,063-156,194,798	AX-156292816
SGCD	rs256835	chr5:155,135,063-156,194,798	AX-14943621
SGCD	rs111375704	chr5:155,135,063-156,194,798	AX-151217032
SGCD	rs256834	chr5:155,135,063-156,194,798	AX-35169935
SGCD	rs256833	chr5:155,135,063-156,194,798	AX-151323233
SGCD	rs256833	chr5:155,135,063-156,194,798	AX-156275682
SGCD	rs256832	chr5:155,135,063-156,194,798	AX-147993448
SGCD	rs256832	chr5:155,135,063-156,194,798	AX-153807283
SGCD	rs256831	chr5:155,135,063-156,194,798	AX-148568986
SGCD	rs256831	chr5:155,135,063-156,194,798	AX-156268284
SGCD	rs56235470	chr5:155,135,063-156,194,798	AX-148369863
SGCD	rs56235470	chr5:155,135,063-156,194,798	AX-156284106
SGCD	rs256830	chr5:155,135,063-156,194,798	AX-156268285
SGCD	rs256830	chr5:155,135,063-156,194,798	AX-156288112
SGCD	rs256829	chr5:155,135,063-156,194,798	AX-113734795
SGCD	rs112504711	chr5:155,135,063-156,194,798	AX-156285736
SGCD	rs256828	chr5:155,135,063-156,194,798	AX-153810205
SGCD	rs256828	chr5:155,135,063-156,194,798	AX-92467994
SGCD	rs256827	chr5:155,135,063-156,194,798	AX-156282341
SGCD	rs256827	chr5:155,135,063-156,194,798	AX-156296986
SGCD	rs113601248	chr5:155,135,063-156,194,798	AX-121773452
SGCD	rs113601248	chr5:155,135,063-156,194,798	AX-148551837

SGCD	rs115321168	chr5:155,135,063-156,194,798	AX-35169951
SGCD	rs139085097	chr5:155,135,063-156,194,798	AX-148823277
SGCD	rs139085097	chr5:155,135,063-156,194,798	AX-156284107
SGCD	rs4639205	chr5:155,135,063-156,194,798	AX-156282343
SGCD	rs4639205	chr5:155,135,063-156,194,798	AX-156296987
SGCD	rs115672382	chr5:155,135,063-156,194,798	AX-156282344
SGCD	rs7700916	chr5:155,135,063-156,194,798	AX-148384501
SGCD	rs7700916	chr5:155,135,063-156,194,798	AX-156282345
SGCD	rs7715748	chr5:155,135,063-156,194,798	AX-123016163
SGCD	rs7715748	chr5:155,135,063-156,194,798	AX-156282346
SGCD	rs7701076	chr5:155,135,063-156,194,798	AX-156282347
SGCD	---	chr5:155,135,063-156,194,798	AX-151421704
SGCD	---	chr5:155,135,063-156,194,798	AX-156284108
SGCD	rs4447971	chr5:155,135,063-156,194,798	AX-148299096
SGCD	rs4447971	chr5:155,135,063-156,194,798	AX-156282348
SGCD	rs2656894	chr5:155,135,063-156,194,798	AX-14943626
SGCD	rs75352978	chr5:155,135,063-156,194,798	AX-14943628
SGCD	rs6889462	chr5:155,135,063-156,194,798	AX-35169957
SGCD	rs78667023	chr5:155,135,063-156,194,798	AX-14943631
SGCD	rs79835445	chr5:155,135,063-156,194,798	AX-14943632
SGCD	rs6869314	chr5:155,135,063-156,194,798	AX-41644749
SGCD	rs72801133	chr5:155,135,063-156,194,798	AX-14943633
SGCD	rs80019372	chr5:155,135,063-156,194,798	AX-14943634
SGCD	rs1432681	chr5:155,135,063-156,194,798	AX-156275684
SGCD	rs1432681	chr5:155,135,063-156,194,798	AX-156292818
SGCD	rs57640108	chr5:155,135,063-156,194,798	AX-148799293
SGCD	rs57640108	chr5:155,135,063-156,194,798	AX-156275685
SGCD	rs2656895	chr5:155,135,063-156,194,798	AX-148069527
SGCD	rs2656895	chr5:155,135,063-156,194,798	AX-156275686
SGCD	rs7715938	chr5:155,135,063-156,194,798	AX-156275687
SGCD	rs7715938	chr5:155,135,063-156,194,798	AX-156292819
SGCD	rs73297120	chr5:155,135,063-156,194,798	AX-156275688
SGCD	rs73297120	chr5:155,135,063-156,194,798	AX-156292820
SGCD	rs4705019	chr5:155,135,063-156,194,798	AX-14943640
SGCD	rs6897602	chr5:155,135,063-156,194,798	AX-12613560
SGCD	rs2082388	chr5:155,135,063-156,194,798	AX-14943642
SGCD	rs17053596	chr5:155,135,063-156,194,798	AX-11301527
SGCD	rs77627069	chr5:155,135,063-156,194,798	AX-14943646
SGCD	rs10463128	chr5:155,135,063-156,194,798	AX-14943647
SGCD	rs6893816	chr5:155,135,063-156,194,798	AX-120669944
SGCD	rs6893816	chr5:155,135,063-156,194,798	AX-156275690
SGCD	rs144320058	chr5:155,135,063-156,194,798	AX-148855843
SGCD	rs144320058	chr5:155,135,063-156,194,798	AX-96733820
SGCD	rs115307128	chr5:155,135,063-156,194,798	AX-14943649
SGCD	rs12517498	chr5:155,135,063-156,194,798	AX-14943650
SGCD	rs7715923	chr5:155,135,063-156,194,798	AX-14943653
SGCD	rs60128166	chr5:155,135,063-156,194,798	AX-120580236
SGCD	rs60128166	chr5:155,135,063-156,194,798	AX-122466600
SGCD	rs11951311	chr5:155,135,063-156,194,798	AX-120074000
SGCD	rs11951311	chr5:155,135,063-156,194,798	AX-148713421
SGCD	rs56183897	chr5:155,135,063-156,194,798	AX-14943654
SGCD	rs17558929	chr5:155,135,063-156,194,798	AX-41644753
SGCD	rs194145	chr5:155,135,063-156,194,798	AX-35169973
SGCD	rs6897183	chr5:155,135,063-156,194,798	AX-156275691
SGCD	rs6897183	chr5:155,135,063-156,194,798	AX-156292822

SGCD	rs145342273	chr5:155,135,063-156,194,798	AX-122424428
SGCD	rs145342273	chr5:155,135,063-156,194,798	AX-156298384
SGCD	rs75152502	chr5:155,135,063-156,194,798	AX-156282349
SGCD	rs75152502	chr5:155,135,063-156,194,798	AX-156296989
SGCD	rs113985602	chr5:155,135,063-156,194,798	AX-147993020
SGCD	rs113985602	chr5:155,135,063-156,194,798	AX-92477667
SGCD	rs253604	chr5:155,135,063-156,194,798	AX-14943658
SGCD	rs12517691	chr5:155,135,063-156,194,798	AX-14943659
SGCD	rs72801137	chr5:155,135,063-156,194,798	AX-14943661
SGCD	rs73297132	chr5:155,135,063-156,194,798	AX-14943662
SGCD	rs2431771	chr5:155,135,063-156,194,798	AX-14943663
SGCD	rs1432682	chr5:155,135,063-156,194,798	AX-35169977
SGCD	rs112947957	chr5:155,135,063-156,194,798	AX-107754603
SGCD	rs112947957	chr5:155,135,063-156,194,798	AX-156298423
SGCD	rs200501640	chr5:155,135,063-156,194,798	AX-156286317
SGCD	rs2431770	chr5:155,135,063-156,194,798	AX-14943665
SGCD	rs77385690	chr5:155,135,063-156,194,798	AX-14943669
SGCD	rs73297136	chr5:155,135,063-156,194,798	AX-14943671
SGCD	rs6897279	chr5:155,135,063-156,194,798	AX-14943672
SGCD	rs10515740	chr5:155,135,063-156,194,798	AX-14943674
SGCD	rs73297140	chr5:155,135,063-156,194,798	AX-14943675
SGCD	rs9313970	chr5:155,135,063-156,194,798	AX-41644759
SGCD	rs17639646	chr5:155,135,063-156,194,798	AX-156275692
SGCD	rs17639646	chr5:155,135,063-156,194,798	AX-156292823
SGCD	rs11955038	chr5:155,135,063-156,194,798	AX-41644763
SGCD	rs147260561	chr5:155,135,063-156,194,798	AX-151416534
SGCD	rs147260561	chr5:155,135,063-156,194,798	AX-156298488
SGCD	rs11746739	chr5:155,135,063-156,194,798	AX-41644765
SGCD	rs253603	chr5:155,135,063-156,194,798	AX-14943681
SGCD	rs73297148	chr5:155,135,063-156,194,798	AX-14943682
SGCD	rs34263435	chr5:155,135,063-156,194,798	AX-120998769
SGCD	rs194144	chr5:155,135,063-156,194,798	AX-14943684
SGCD	rs11743093	chr5:155,135,063-156,194,798	AX-156275693
SGCD	rs11743093	chr5:155,135,063-156,194,798	AX-156292824
SGCD	rs12521822	chr5:155,135,063-156,194,798	AX-35169993
SGCD	rs253602	chr5:155,135,063-156,194,798	AX-12531518
SGCD	rs113494196	chr5:155,135,063-156,194,798	AX-151412317
SGCD	rs113494196	chr5:155,135,063-156,194,798	AX-156275694
SGCD	rs253601	chr5:155,135,063-156,194,798	AX-148620405
SGCD	rs253601	chr5:155,135,063-156,194,798	AX-156275695
SGCD	rs253600	chr5:155,135,063-156,194,798	AX-147999423
SGCD	rs253600	chr5:155,135,063-156,194,798	AX-153812552
SGCD	rs6877566	chr5:155,135,063-156,194,798	AX-35169995
SGCD	rs6885454	chr5:155,135,063-156,194,798	AX-11592071
SGCD	rs79127894	chr5:155,135,063-156,194,798	AX-35169997
SGCD	rs253599	chr5:155,135,063-156,194,798	AX-156292825
SGCD	rs1432683	chr5:155,135,063-156,194,798	AX-14943692
SGCD	rs17053604	chr5:155,135,063-156,194,798	AX-41644767
SGCD	rs2216656	chr5:155,135,063-156,194,798	AX-12521393
SGCD	rs17639859	chr5:155,135,063-156,194,798	AX-14943695
SGCD	rs253598	chr5:155,135,063-156,194,798	AX-41644771
SGCD	rs77362812	chr5:155,135,063-156,194,798	AX-14943696
SGCD	rs4337839	chr5:155,135,063-156,194,798	AX-11501712
SGCD	rs6861920	chr5:155,135,063-156,194,798	AX-41644777
SGCD	rs11750620	chr5:155,135,063-156,194,798	AX-12418487

SGCD	rs35783917	chr5:155,135,063-156,194,798	AX-120374372
SGCD	rs253596	chr5:155,135,063-156,194,798	AX-14943700
SGCD	rs253595	chr5:155,135,063-156,194,798	AX-41644779
SGCD	rs253594	chr5:155,135,063-156,194,798	AX-11397401
SGCD	rs253593	chr5:155,135,063-156,194,798	AX-14943702
SGCD	rs17053613	chr5:155,135,063-156,194,798	AX-12487907
SGCD	rs10071452	chr5:155,135,063-156,194,798	AX-14943706
SGCD	rs10076239	chr5:155,135,063-156,194,798	AX-156275697
SGCD	rs10076239	chr5:155,135,063-156,194,798	AX-156292826
SGCD	rs73297178	chr5:155,135,063-156,194,798	AX-156275698
SGCD	rs73297178	chr5:155,135,063-156,194,798	AX-156292827
SGCD	---	chr5:155,135,063-156,194,798	AX-14943710
SGCD	rs79963846	chr5:155,135,063-156,194,798	AX-114216059
SGCD	rs79963846	chr5:155,135,063-156,194,798	AX-156275699
SGCD	rs6880966	chr5:155,135,063-156,194,798	AX-156275700
SGCD	rs6880966	chr5:155,135,063-156,194,798	AX-156292828
SGCD	rs11748068	chr5:155,135,063-156,194,798	AX-14943711
SGCD	rs13358990	chr5:155,135,063-156,194,798	AX-41644785
SGCD	rs6890183	chr5:155,135,063-156,194,798	AX-41644787
SGCD	rs34260400	chr5:155,135,063-156,194,798	AX-156292829
SGCD	rs73297179	chr5:155,135,063-156,194,798	AX-14943718
SGCD	rs11749788	chr5:155,135,063-156,194,798	AX-148419817
SGCD	rs11749788	chr5:155,135,063-156,194,798	AX-92510862
SGCD	rs11738926	chr5:155,135,063-156,194,798	AX-148015920
SGCD	rs11738926	chr5:155,135,063-156,194,798	AX-148669455
SGCD	rs7717533	chr5:155,135,063-156,194,798	AX-11640809
SGCD	rs73297180	chr5:155,135,063-156,194,798	AX-14943722
SGCD	rs6876726	chr5:155,135,063-156,194,798	AX-14943724
SGCD	rs11948829	chr5:155,135,063-156,194,798	AX-156275701
SGCD	rs11948829	chr5:155,135,063-156,194,798	AX-156292830
SGCD	rs79527093	chr5:155,135,063-156,194,798	AX-14943726
SGCD	rs73297184	chr5:155,135,063-156,194,798	AX-14943727
SGCD	rs1368301	chr5:155,135,063-156,194,798	AX-14943728
SGCD	rs6874767	chr5:155,135,063-156,194,798	AX-11591424
SGCD	rs75476567	chr5:155,135,063-156,194,798	AX-35170031
SGCD	rs7727643	chr5:155,135,063-156,194,798	AX-12638365
SGCD	rs60716901	chr5:155,135,063-156,194,798	AX-14943730
SGCD	rs58558564	chr5:155,135,063-156,194,798	AX-14943731
SGCD	rs253592	chr5:155,135,063-156,194,798	AX-14943733
SGCD	rs1978450	chr5:155,135,063-156,194,798	AX-113734799
SGCD	rs1978450	chr5:155,135,063-156,194,798	AX-156282350
SGCD	rs28432313	chr5:155,135,063-156,194,798	AX-41644795
SGCD	rs146445826	chr5:155,135,063-156,194,798	AX-151412833
SGCD	rs146445826	chr5:155,135,063-156,194,798	AX-156298114
SGCD	rs144623770	chr5:155,135,063-156,194,798	AX-156284839
SGCD	rs78909890	chr5:155,135,063-156,194,798	AX-14943738
SGCD	rs73297187	chr5:155,135,063-156,194,798	AX-156275702
SGCD	rs73297187	chr5:155,135,063-156,194,798	AX-156292831
SGCD	rs9686475	chr5:155,135,063-156,194,798	AX-156275703
SGCD	rs73297188	chr5:155,135,063-156,194,798	AX-14943741
SGCD	rs2312187	chr5:155,135,063-156,194,798	AX-14943743
SGCD	rs2312188	chr5:155,135,063-156,194,798	AX-14943744
SGCD	rs77074720	chr5:155,135,063-156,194,798	AX-35170039
SGCD	rs2431769	chr5:155,135,063-156,194,798	AX-35170041
SGCD	rs201119142	chr5:155,135,063-156,194,798	AX-151294464

SGCD	rs437395	chr5:155,135,063-156,194,798	AX-14943751
SGCD	rs4705020	chr5:155,135,063-156,194,798	AX-41644799
SGCD	rs447983	chr5:155,135,063-156,194,798	AX-14943752
SGCD	rs60598425	chr5:155,135,063-156,194,798	AX-14943753
SGCD	rs447938	chr5:155,135,063-156,194,798	AX-11507309
SGCD	rs365187	chr5:155,135,063-156,194,798	AX-35170047
SGCD	rs412201	chr5:155,135,063-156,194,798	AX-35170049
SGCD	rs450151	chr5:155,135,063-156,194,798	AX-35170051
SGCD	rs11955923	chr5:155,135,063-156,194,798	AX-14943757
SGCD	rs11951246	chr5:155,135,063-156,194,798	AX-14943758
SGCD	rs11951275	chr5:155,135,063-156,194,798	AX-156275704
SGCD	rs11951275	chr5:155,135,063-156,194,798	AX-156292833
SGCD	rs6880543	chr5:155,135,063-156,194,798	AX-14943764
SGCD	rs146253791	chr5:155,135,063-156,194,798	AX-151392703
SGCD	rs146253791	chr5:155,135,063-156,194,798	AX-156298471
SGCD	rs73297195	chr5:155,135,063-156,194,798	AX-14943765
SGCD	rs1036180	chr5:155,135,063-156,194,798	AX-35170059
SGCD	rs73297198	chr5:155,135,063-156,194,798	AX-14943766
SGCD	rs74412895	chr5:155,135,063-156,194,798	AX-35170061
SGCD	rs10071360	chr5:155,135,063-156,194,798	AX-156275705
SGCD	rs10071360	chr5:155,135,063-156,194,798	AX-156292834
SGCD	rs7448097	chr5:155,135,063-156,194,798	AX-35170065
SGCD	rs3846689	chr5:155,135,063-156,194,798	AX-156292835
SGCD	rs80275589	chr5:155,135,063-156,194,798	AX-14943768
SGCD	rs3857410	chr5:155,135,063-156,194,798	AX-12563743
SGCD	rs7720258	chr5:155,135,063-156,194,798	AX-148762318
SGCD	rs7720258	chr5:155,135,063-156,194,798	AX-156275707
SGCD	rs7715587	chr5:155,135,063-156,194,798	AX-156282351
SGCD	rs7715587	chr5:155,135,063-156,194,798	AX-156296990
SGCD	rs4080412	chr5:155,135,063-156,194,798	AX-151126956
SGCD	rs79782815	chr5:155,135,063-156,194,798	AX-14943771
SGCD	rs11354048	chr5:155,135,063-156,194,798	AX-151271169
SGCD	rs73297202	chr5:155,135,063-156,194,798	AX-14943772
SGCD	rs6880030	chr5:155,135,063-156,194,798	AX-35170073
SGCD	rs6880155	chr5:155,135,063-156,194,798	AX-148428605
SGCD	rs111811162	chr5:155,135,063-156,194,798	AX-86896881
SGCD	rs11135273	chr5:155,135,063-156,194,798	AX-14943774
SGCD	rs11135274	chr5:155,135,063-156,194,798	AX-156275708
SGCD	rs11740312	chr5:155,135,063-156,194,798	AX-35170075
SGCD	rs61318634	chr5:155,135,063-156,194,798	AX-14943775
SGCD	rs74746676	chr5:155,135,063-156,194,798	AX-35170077
SGCD	rs111862138	chr5:155,135,063-156,194,798	AX-148418473
SGCD	rs111862138	chr5:155,135,063-156,194,798	AX-148787811
SGCD	rs112146477	chr5:155,135,063-156,194,798	AX-148287214
SGCD	rs112146477	chr5:155,135,063-156,194,798	AX-156284109
SGCD	rs171690	chr5:155,135,063-156,194,798	AX-14943779
SGCD	rs10079506	chr5:155,135,063-156,194,798	AX-14943780
SGCD	rs10055481	chr5:155,135,063-156,194,798	AX-35170087
SGCD	rs78311701	chr5:155,135,063-156,194,798	AX-148740252
SGCD	rs78311701	chr5:155,135,063-156,194,798	AX-156275709
SGCD	rs199772807	chr5:155,135,063-156,194,798	AX-105159667
SGCD	rs253605	chr5:155,135,063-156,194,798	AX-14943781
SGCD	rs55634634	chr5:155,135,063-156,194,798	AX-14943782
SGCD	rs7718591	chr5:155,135,063-156,194,798	AX-156275710
SGCD	rs75776487	chr5:155,135,063-156,194,798	AX-14943784

SGCD	rs7730140	chr5:155,135,063-156,194,798	AX-41644803
SGCD	rs73299105	chr5:155,135,063-156,194,798	AX-14943785
SGCD	rs74869065	chr5:155,135,063-156,194,798	AX-14943786
SGCD	rs57484573	chr5:155,135,063-156,194,798	AX-122206137
SGCD	rs57484573	chr5:155,135,063-156,194,798	AX-156298450
SGCD	rs11742816	chr5:155,135,063-156,194,798	AX-14943790
SGCD	rs17559950	chr5:155,135,063-156,194,798	AX-14943792
SGCD	rs253606	chr5:155,135,063-156,194,798	AX-86898080
SGCD	rs73812688	chr5:155,135,063-156,194,798	AX-35170095
SGCD	rs73299107	chr5:155,135,063-156,194,798	AX-35170097
SGCD	rs10069144	chr5:155,135,063-156,194,798	AX-12380330
SGCD	rs17640310	chr5:155,135,063-156,194,798	AX-12502326
SGCD	rs7718881	chr5:155,135,063-156,194,798	AX-41644807
SGCD	rs60989660	chr5:155,135,063-156,194,798	AX-14943799
SGCD	rs73299111	chr5:155,135,063-156,194,798	AX-14943802
SGCD	rs10052818	chr5:155,135,063-156,194,798	AX-14943804
SGCD	rs74466783	chr5:155,135,063-156,194,798	AX-14943805
SGCD	rs73299113	chr5:155,135,063-156,194,798	AX-14943806
SGCD	rs4382149	chr5:155,135,063-156,194,798	AX-14943807
SGCD	rs17640346	chr5:155,135,063-156,194,798	AX-156275712
SGCD	rs17640346	chr5:155,135,063-156,194,798	AX-156292837
SGCD	rs253607	chr5:155,135,063-156,194,798	AX-35170105
SGCD	rs17640369	chr5:155,135,063-156,194,798	AX-14943808
SGCD	rs6556717	chr5:155,135,063-156,194,798	AX-12602551
SGCD	rs934418	chr5:155,135,063-156,194,798	AX-14943812
SGCD	rs77667734	chr5:155,135,063-156,194,798	AX-148803726
SGCD	rs77667734	chr5:155,135,063-156,194,798	AX-156278663
SGCD	rs112881066	chr5:155,135,063-156,194,798	AX-148789121
SGCD	rs112881066	chr5:155,135,063-156,194,798	AX-156284110
SGCD	rs113980265	chr5:155,135,063-156,194,798	AX-96791935
SGCD	rs10214042	chr5:155,135,063-156,194,798	AX-121254438
SGCD	rs10214042	chr5:155,135,063-156,194,798	AX-148123835
SGCD	rs55975207	chr5:155,135,063-156,194,798	AX-122474798
SGCD	rs55975207	chr5:155,135,063-156,194,798	AX-96630193
SGCD	rs56390385	chr5:155,135,063-156,194,798	AX-148142989
SGCD	rs56390385	chr5:155,135,063-156,194,798	AX-156284111
SGCD	rs147919561	chr5:155,135,063-156,194,798	AX-148788855
SGCD	rs147919561	chr5:155,135,063-156,194,798	AX-156284112
SGCD	rs10040642	chr5:155,135,063-156,194,798	AX-96687710
SGCD	rs147791143	chr5:155,135,063-156,194,798	AX-148723324
SGCD	rs147791143	chr5:155,135,063-156,194,798	AX-156284113
SGCD	rs9687336	chr5:155,135,063-156,194,798	AX-151402247
SGCD	rs9687336	chr5:155,135,063-156,194,798	AX-156284114
SGCD	rs111596506	chr5:155,135,063-156,194,798	AX-148588618
SGCD	rs111596506	chr5:155,135,063-156,194,798	AX-156284115
SGCD	rs139288409	chr5:155,135,063-156,194,798	AX-120511009
SGCD	rs139288409	chr5:155,135,063-156,194,798	AX-148599889
SGCD	rs147232585	chr5:155,135,063-156,194,798	AX-148789014
SGCD	rs147232585	chr5:155,135,063-156,194,798	AX-156284116
SGCD	rs6883510	chr5:155,135,063-156,194,798	AX-148159420
SGCD	rs6883510	chr5:155,135,063-156,194,798	AX-156275713
SGCD	rs11135286	chr5:155,135,063-156,194,798	AX-105115286
SGCD	rs11135286	chr5:155,135,063-156,194,798	AX-156275714
SGCD	rs11135287	chr5:155,135,063-156,194,798	AX-151174257
SGCD	rs11135287	chr5:155,135,063-156,194,798	AX-151219012

SGCD	---	chr5:155,135,063-156,194,798	AX-148070038
SGCD	---	chr5:155,135,063-156,194,798	AX-156275715
SGCD	rs80206401	chr5:155,135,063-156,194,798	AX-148548965
SGCD	rs80206401	chr5:155,135,063-156,194,798	AX-156284117
SGCD	rs6894554	chr5:155,135,063-156,194,798	AX-148438707
SGCD	rs6894554	chr5:155,135,063-156,194,798	AX-156275716
SGCD	rs7706946	chr5:155,135,063-156,194,798	AX-156275717
SGCD	rs12520121	chr5:155,135,063-156,194,798	AX-35170115
SGCD	rs6859694	chr5:155,135,063-156,194,798	AX-148191879
SGCD	rs6859694	chr5:155,135,063-156,194,798	AX-156275718
SGCD	rs6887546	chr5:155,135,063-156,194,798	AX-156275719
SGCD	rs6887546	chr5:155,135,063-156,194,798	AX-156292838
SGCD	rs6860238	chr5:155,135,063-156,194,798	AX-156275720
SGCD	rs6860238	chr5:155,135,063-156,194,798	AX-156292839
SGCD	rs10077734	chr5:155,135,063-156,194,798	AX-14943817
SGCD	rs146842955	chr5:155,135,063-156,194,798	AX-156285315
SGCD	---	chr5:155,135,063-156,194,798	AX-14943821
SGCD	rs75354763	chr5:155,135,063-156,194,798	AX-156275722
SGCD	rs75354763	chr5:155,135,063-156,194,798	AX-156292840
SGCD	rs17053637	chr5:155,135,063-156,194,798	AX-156282353
SGCD	---	chr5:155,135,063-156,194,798	AX-122561120
SGCD	rs2436327	chr5:155,135,063-156,194,798	AX-41644811
SGCD	rs2431401	chr5:155,135,063-156,194,798	AX-156282354
SGCD	rs2431401	chr5:155,135,063-156,194,798	AX-156296992
SGCD	rs6864938	chr5:155,135,063-156,194,798	AX-14943832
SGCD	rs7710178	chr5:155,135,063-156,194,798	AX-12637757
SGCD	rs10077411	chr5:155,135,063-156,194,798	AX-11091256
SGCD	rs113379066	chr5:155,135,063-156,194,798	AX-113734801
SGCD	rs113379066	chr5:155,135,063-156,194,798	AX-156292841
SGCD	rs72801154	chr5:155,135,063-156,194,798	AX-35170131
SGCD	rs2277956	chr5:155,135,063-156,194,798	AX-41644827
SGCD	rs280475	chr5:155,135,063-156,194,798	AX-14943840
SGCD	---	chr5:155,135,063-156,194,798	AX-86560220
SGCD	rs181875	chr5:155,135,063-156,194,798	AX-14943842
SGCD	rs72801155	chr5:155,135,063-156,194,798	AX-35170135
SGCD	rs3797574	chr5:155,135,063-156,194,798	AX-156292842
SGCD	rs157292	chr5:155,135,063-156,194,798	AX-12470271
SGCD	rs157292	chr5:155,135,063-156,194,798	AX-156292843
SGCD	rs157293	chr5:155,135,063-156,194,798	AX-14943845
SGCD	rs3797577	chr5:155,135,063-156,194,798	AX-14943848
SGCD	rs73299128	chr5:155,135,063-156,194,798	AX-14943850
SGCD	rs201111387	chr5:155,135,063-156,194,798	AX-14943852
SGCD	rs201111387	chr5:155,135,063-156,194,798	AX-151161354
SGCD	rs157290	chr5:155,135,063-156,194,798	AX-156275724
SGCD	rs157290	chr5:155,135,063-156,194,798	AX-156292844
SGCD	rs157291	chr5:155,135,063-156,194,798	AX-35170143
SGCD	rs60105121	chr5:155,135,063-156,194,798	AX-14943853
SGCD	rs10515738	chr5:155,135,063-156,194,798	AX-41644837
SGCD	rs73299136	chr5:155,135,063-156,194,798	AX-14943857
SGCD	rs73299137	chr5:155,135,063-156,194,798	AX-156275725
SGCD	rs73299137	chr5:155,135,063-156,194,798	AX-156292845
SGCD	rs11739522	chr5:155,135,063-156,194,798	AX-14943858
SGCD	rs7714176	chr5:155,135,063-156,194,798	AX-14943861
SGCD	rs73299139	chr5:155,135,063-156,194,798	AX-14943862
SGCD	rs73299140	chr5:155,135,063-156,194,798	AX-14943863

SGCD	rs888842	chr5:155,135,063-156,194,798	AX-14943864
SGCD	rs7720093	chr5:155,135,063-156,194,798	AX-41644847
SGCD	rs7715397	chr5:155,135,063-156,194,798	AX-41644849
SGCD	rs6872066	chr5:155,135,063-156,194,798	AX-35170159
SGCD	rs6556729	chr5:155,135,063-156,194,798	AX-35170161
SGCD	rs76814622	chr5:155,135,063-156,194,798	AX-35170163
SGCD	rs142300885	chr5:155,135,063-156,194,798	AX-156286411
SGCD	rs280468	chr5:155,135,063-156,194,798	AX-35170165
SGCD	rs280469	chr5:155,135,063-156,194,798	AX-156282355
SGCD	rs280470	chr5:155,135,063-156,194,798	AX-14943868
SGCD	rs76906458	chr5:155,135,063-156,194,798	AX-35170167
SGCD	rs35305422	chr5:155,135,063-156,194,798	AX-41644855
SGCD	rs280471	chr5:155,135,063-156,194,798	AX-148711153
SGCD	rs280471	chr5:155,135,063-156,194,798	AX-156275726
SGCD	rs182203	chr5:155,135,063-156,194,798	AX-14943874
SGCD	rs280472	chr5:155,135,063-156,194,798	AX-156275728
SGCD	rs203997	chr5:155,135,063-156,194,798	AX-156275729
SGCD	rs10068837	chr5:155,135,063-156,194,798	AX-113078911
SGCD	rs10068837	chr5:155,135,063-156,194,798	AX-148466084
SGCD	rs73299146	chr5:155,135,063-156,194,798	AX-148636106
SGCD	rs73299146	chr5:155,135,063-156,194,798	AX-156275730
SGCD	rs563016540	chr5:155,135,063-156,194,798	AX-151352909
SGCD	rs563016540	chr5:155,135,063-156,194,798	AX-156298075
SGCD	rs11957615	chr5:155,135,063-156,194,798	AX-156275731
SGCD	rs11957615	chr5:155,135,063-156,194,798	AX-156292849
SGCD	rs280473	chr5:155,135,063-156,194,798	AX-41644861
SGCD	rs35321875	chr5:155,135,063-156,194,798	AX-156292850
SGCD	rs77081211	chr5:155,135,063-156,194,798	AX-156285191
SGCD	rs73299150	chr5:155,135,063-156,194,798	AX-156275733
SGCD	rs73299150	chr5:155,135,063-156,194,798	AX-156292851
SGCD	rs73299153	chr5:155,135,063-156,194,798	AX-14943879
SGCD	rs280474	chr5:155,135,063-156,194,798	AX-156292852
SGCD	rs73299154	chr5:155,135,063-156,194,798	AX-14943880
SGCD	rs73299156	chr5:155,135,063-156,194,798	AX-148653635
SGCD	rs1833609	chr5:155,135,063-156,194,798	AX-156268286
SGCD	rs1833609	chr5:155,135,063-156,194,798	AX-156288113
SGCD	rs6556736	chr5:155,135,063-156,194,798	AX-156282356
SGCD	rs6556736	chr5:155,135,063-156,194,798	AX-156296994
SGCD	rs6890122	chr5:155,135,063-156,194,798	AX-151357561
SGCD	rs6890122	chr5:155,135,063-156,194,798	AX-156282357
SGCD	rs6895333	chr5:155,135,063-156,194,798	AX-156296995
SGCD	rs11953051	chr5:155,135,063-156,194,798	AX-156296996
SGCD	rs76657545	chr5:155,135,063-156,194,798	AX-14943882
SGCD	rs9314007	chr5:155,135,063-156,194,798	AX-153719393
SGCD	rs9314007	chr5:155,135,063-156,194,798	AX-153739380
SGCD	---	chr5:155,135,063-156,194,798	AX-156282360
SGCD	rs366643	chr5:155,135,063-156,194,798	AX-148209264
SGCD	rs73299159	chr5:155,135,063-156,194,798	AX-156282362
SGCD	rs73299159	chr5:155,135,063-156,194,798	AX-156296998
SGCD	rs145050755	chr5:155,135,063-156,194,798	AX-156286322
SGCD	rs149206504	chr5:155,135,063-156,194,798	AX-122780654
SGCD	rs149206504	chr5:155,135,063-156,194,798	AX-156284118
SGCD	rs9885279	chr5:155,135,063-156,194,798	AX-105173362
SGCD	rs9885279	chr5:155,135,063-156,194,798	AX-96633999
SGCD	rs9885282	chr5:155,135,063-156,194,798	AX-156282363

SGCD	rs9885311	chr5:155,135,063-156,194,798	AX-95864977
SGCD	rs9885311	chr5:155,135,063-156,194,798	AX-96648753
SGCD	rs143543864	chr5:155,135,063-156,194,798	AX-148783480
SGCD	rs143543864	chr5:155,135,063-156,194,798	AX-156284119
SGCD	rs150496590	chr5:155,135,063-156,194,798	AX-120620889
SGCD	rs150496590	chr5:155,135,063-156,194,798	AX-156284120
SGCD	rs189904433	chr5:155,135,063-156,194,798	AX-119990862
SGCD	rs189904433	chr5:155,135,063-156,194,798	AX-148083014
SGCD	rs192112737	chr5:155,135,063-156,194,798	AX-148784208
SGCD	rs192112737	chr5:155,135,063-156,194,798	AX-96870230
SGCD	rs149255623	chr5:155,135,063-156,194,798	AX-119414695
SGCD	rs149255623	chr5:155,135,063-156,194,798	AX-151404715
SGCD	rs148208831	chr5:155,135,063-156,194,798	AX-120660876
SGCD	rs148208831	chr5:155,135,063-156,194,798	AX-120921945
SGCD	rs144089755	chr5:155,135,063-156,194,798	AX-123001580
SGCD	rs144089755	chr5:155,135,063-156,194,798	AX-149461608
SGCD	rs146004678	chr5:155,135,063-156,194,798	AX-151410424
SGCD	rs146004678	chr5:155,135,063-156,194,798	AX-156284121
SGCD	rs11956458	chr5:155,135,063-156,194,798	AX-113578874
SGCD	rs11956458	chr5:155,135,063-156,194,798	AX-148764402
SGCD	rs11955254	chr5:155,135,063-156,194,798	AX-148783222
SGCD	rs11955254	chr5:155,135,063-156,194,798	AX-156284122
SGCD	---	chr5:155,135,063-156,194,798	AX-151319793
SGCD	---	chr5:155,135,063-156,194,798	AX-156284808
SGCD	rs141873448	chr5:155,135,063-156,194,798	AX-113590779
SGCD	rs141873448	chr5:155,135,063-156,194,798	AX-148713815
SGCD	rs7717319	chr5:155,135,063-156,194,798	AX-151293733
SGCD	rs7717319	chr5:155,135,063-156,194,798	AX-156284123
SGCD	rs113954092	chr5:155,135,063-156,194,798	AX-148204395
SGCD	rs113954092	chr5:155,135,063-156,194,798	AX-156284124
SGCD	rs188563161	chr5:155,135,063-156,194,798	AX-88317894
SGCD	rs149394887	chr5:155,135,063-156,194,798	AX-151187573
SGCD	rs149394887	chr5:155,135,063-156,194,798	AX-156284125
SGCD	rs143073862	chr5:155,135,063-156,194,798	AX-113113245
SGCD	rs143073862	chr5:155,135,063-156,194,798	AX-156284126
SGCD	rs11957592	chr5:155,135,063-156,194,798	AX-148638677
SGCD	rs11953741	chr5:155,135,063-156,194,798	AX-148799434
SGCD	rs11953741	chr5:155,135,063-156,194,798	AX-156284127
SGCD	rs138095347	chr5:155,135,063-156,194,798	AX-148664909
SGCD	rs138095347	chr5:155,135,063-156,194,798	AX-156284128
SGCD	---	chr5:155,135,063-156,194,798	AX-151395167
SGCD	---	chr5:155,135,063-156,194,798	AX-156284129
SGCD	rs189047611	chr5:155,135,063-156,194,798	AX-148131209
SGCD	rs189047611	chr5:155,135,063-156,194,798	AX-148678366
SGCD	rs144286229	chr5:155,135,063-156,194,798	AX-148529666
SGCD	rs144286229	chr5:155,135,063-156,194,798	AX-156284130
SGCD	rs280453	chr5:155,135,063-156,194,798	AX-92496998
SGCD	rs60774908	chr5:155,135,063-156,194,798	AX-151367476
SGCD	rs60774908	chr5:155,135,063-156,194,798	AX-156284131
SGCD	rs56959778	chr5:155,135,063-156,194,798	AX-148677935
SGCD	rs56959778	chr5:155,135,063-156,194,798	AX-156284132
SGCD	rs147755620	chr5:155,135,063-156,194,798	AX-148852712
SGCD	rs147755620	chr5:155,135,063-156,194,798	AX-156284133
SGCD	rs112696830	chr5:155,135,063-156,194,798	AX-148139338
SGCD	rs112696830	chr5:155,135,063-156,194,798	AX-149273688

SGCD	rs280455	chr5:155,135,063-156,194,798	AX-148085112
SGCD	rs280455	chr5:155,135,063-156,194,798	AX-156282364
SGCD	rs113568042	chr5:155,135,063-156,194,798	AX-113547835
SGCD	rs113568042	chr5:155,135,063-156,194,798	AX-148105050
SGCD	rs12519349	chr5:155,135,063-156,194,798	AX-121863665
SGCD	rs12519349	chr5:155,135,063-156,194,798	AX-150507622
SGCD	rs12521380	chr5:155,135,063-156,194,798	AX-151189162
SGCD	rs12521380	chr5:155,135,063-156,194,798	AX-156284134
SGCD	rs60841274	chr5:155,135,063-156,194,798	AX-148682120
SGCD	rs60841274	chr5:155,135,063-156,194,798	AX-156284135
SGCD	rs57210770	chr5:155,135,063-156,194,798	AX-148654699
SGCD	rs57210770	chr5:155,135,063-156,194,798	AX-156284136
SGCD	rs59036281	chr5:155,135,063-156,194,798	AX-148036666
SGCD	rs59036281	chr5:155,135,063-156,194,798	AX-148671922
SGCD	rs73299171	chr5:155,135,063-156,194,798	AX-14943888
SGCD	rs73299173	chr5:155,135,063-156,194,798	AX-14943889
SGCD	rs74577265	chr5:155,135,063-156,194,798	AX-35170207
SGCD	rs73299175	chr5:155,135,063-156,194,798	AX-156282366
SGCD	rs73299175	chr5:155,135,063-156,194,798	AX-156296999
SGCD	rs280456	chr5:155,135,063-156,194,798	AX-14943890
SGCD	rs73815171	chr5:155,135,063-156,194,798	AX-14943891
SGCD	rs400303	chr5:155,135,063-156,194,798	AX-14943892
SGCD	rs280457	chr5:155,135,063-156,194,798	AX-14943895
SGCD	rs280458	chr5:155,135,063-156,194,798	AX-14943896
SGCD	rs280459	chr5:155,135,063-156,194,798	AX-14943897
SGCD	rs57911436	chr5:155,135,063-156,194,798	AX-14943899
SGCD	rs6877118	chr5:155,135,063-156,194,798	AX-41644871
SGCD	rs73299180	chr5:155,135,063-156,194,798	AX-14943903
SGCD	---	chr5:155,135,063-156,194,798	AX-41644873
SGCD	rs73299183	chr5:155,135,063-156,194,798	AX-35170219
SGCD	rs78706094	chr5:155,135,063-156,194,798	AX-14943906
SGCD	rs77177982	chr5:155,135,063-156,194,798	AX-156275736
SGCD	rs77177982	chr5:155,135,063-156,194,798	AX-156292853
SGCD	rs10611348	chr5:155,135,063-156,194,798	AX-151296659
SGCD	rs10611348	chr5:155,135,063-156,194,798	AX-156298390
SGCD	rs280461	chr5:155,135,063-156,194,798	AX-41644875
SGCD	rs73299187	chr5:155,135,063-156,194,798	AX-14943910
SGCD	rs280462	chr5:155,135,063-156,194,798	AX-11411368
SGCD	rs73299189	chr5:155,135,063-156,194,798	AX-14943911
SGCD	rs73299190	chr5:155,135,063-156,194,798	AX-14943912
SGCD	rs78787033	chr5:155,135,063-156,194,798	AX-156275737
SGCD	rs78787033	chr5:155,135,063-156,194,798	AX-156292854
SGCD	rs80072297	chr5:155,135,063-156,194,798	AX-35170225
SGCD	rs73299193	chr5:155,135,063-156,194,798	AX-14943913
SGCD	rs73299195	chr5:155,135,063-156,194,798	AX-14943914
SGCD	rs280463	chr5:155,135,063-156,194,798	AX-14943916
SGCD	rs76893442	chr5:155,135,063-156,194,798	AX-14943917
SGCD	rs144351878	chr5:155,135,063-156,194,798	AX-151410476
SGCD	rs144351878	chr5:155,135,063-156,194,798	AX-156297763
SGCD	rs73299198	chr5:155,135,063-156,194,798	AX-14943918
SGCD	rs114645617	chr5:155,135,063-156,194,798	AX-14943919
SGCD	rs280464	chr5:155,135,063-156,194,798	AX-148072026
SGCD	rs11949585	chr5:155,135,063-156,194,798	AX-14943921
SGCD	rs280465	chr5:155,135,063-156,194,798	AX-35170229
SGCD	rs62380868	chr5:155,135,063-156,194,798	AX-35170231

SGCD	rs280466	chr5:155,135,063-156,194,798	AX-14943923
SGCD	rs73299201	chr5:155,135,063-156,194,798	AX-14943925
SGCD	rs17053678	chr5:155,135,063-156,194,798	AX-41644887
SGCD	rs57944803	chr5:155,135,063-156,194,798	AX-14943927
SGCD	rs73299202	chr5:155,135,063-156,194,798	AX-35170237
SGCD	rs280467	chr5:155,135,063-156,194,798	AX-156275738
SGCD	rs59205688	chr5:155,135,063-156,194,798	AX-14943929
SGCD	rs73301108	chr5:155,135,063-156,194,798	AX-14943930
SGCD	rs111415262	chr5:155,135,063-156,194,798	AX-148789664
SGCD	rs111415262	chr5:155,135,063-156,194,798	AX-156284137
SGCD	rs6860519	chr5:155,135,063-156,194,798	AX-14943938
SGCD	rs1363743	chr5:155,135,063-156,194,798	AX-41644891
SGCD	rs11948320	chr5:155,135,063-156,194,798	AX-12423861
SGCD	rs73301123	chr5:155,135,063-156,194,798	AX-14943943
SGCD	rs17561110	chr5:155,135,063-156,194,798	AX-113251390
SGCD	rs17561110	chr5:155,135,063-156,194,798	AX-156292856
SGCD	rs17561145	chr5:155,135,063-156,194,798	AX-14943945
SGCD	rs11743012	chr5:155,135,063-156,194,798	AX-41644897
SGCD	rs11740282	chr5:155,135,063-156,194,798	AX-151332444
SGCD	rs11740282	chr5:155,135,063-156,194,798	AX-156284138
SGCD	rs1363742	chr5:155,135,063-156,194,798	AX-14943955
SGCD	---	chr5:155,135,063-156,194,798	AX-156297612
SGCD	rs79258400	chr5:155,135,063-156,194,798	AX-14943957
SGCD	rs7723780	chr5:155,135,063-156,194,798	AX-14943958
SGCD	rs56785833	chr5:155,135,063-156,194,798	AX-156298451
SGCD	rs56785833	chr5:155,135,063-156,194,798	AX-38254451
SGCD	rs1422220	chr5:155,135,063-156,194,798	AX-156282367
SGCD	rs1422220	chr5:155,135,063-156,194,798	AX-156297000
SGCD	rs76711652	chr5:155,135,063-156,194,798	AX-35170253
SGCD	rs11743091	chr5:155,135,063-156,194,798	AX-14943960
SGCD	rs151318600	chr5:155,135,063-156,194,798	AX-151424786
SGCD	rs151318600	chr5:155,135,063-156,194,798	AX-156297854
SGCD	rs6886792	chr5:155,135,063-156,194,798	AX-14943968
SGCD	rs6871079	chr5:155,135,063-156,194,798	AX-14943969
SGCD	rs4331879	chr5:155,135,063-156,194,798	AX-148124001
SGCD	rs4331879	chr5:155,135,063-156,194,798	AX-156275739
SGCD	rs4355508	chr5:155,135,063-156,194,798	AX-105087015
SGCD	rs4355508	chr5:155,135,063-156,194,798	AX-148225318
SGCD	rs4295372	chr5:155,135,063-156,194,798	AX-114066099
SGCD	rs4295372	chr5:155,135,063-156,194,798	AX-84629682
SGCD	rs11743435	chr5:155,135,063-156,194,798	AX-122367896
SGCD	rs11743435	chr5:155,135,063-156,194,798	AX-156275740
SGCD	rs10068125	chr5:155,135,063-156,194,798	AX-35170259
SGCD	rs281047	chr5:155,135,063-156,194,798	AX-11411749
SGCD	rs80220415	chr5:155,135,063-156,194,798	AX-35170261
SGCD	rs6872094	chr5:155,135,063-156,194,798	AX-12612674
SGCD	rs42685	chr5:155,135,063-156,194,798	AX-14943977
SGCD	rs73301125	chr5:155,135,063-156,194,798	AX-14943979
SGCD	rs32063	chr5:155,135,063-156,194,798	AX-14943981
SGCD	rs6877490	chr5:155,135,063-156,194,798	AX-11591578
SGCD	rs71822411	chr5:155,135,063-156,194,798	AX-151439558
SGCD	rs71822411	chr5:155,135,063-156,194,798	AX-156298241
SGCD	rs32062	chr5:155,135,063-156,194,798	AX-14943983
SGCD	rs41074	chr5:155,135,063-156,194,798	AX-113251392
SGCD	rs157320	chr5:155,135,063-156,194,798	AX-156275742

SGCD	rs40486	chr5:155,135,063-156,194,798	AX-14943984
SGCD	rs59802535	chr5:155,135,063-156,194,798	AX-14943985
SGCD	rs32061	chr5:155,135,063-156,194,798	AX-14943986
SGCD	rs32060	chr5:155,135,063-156,194,798	AX-11436952
SGCD	rs185246	chr5:155,135,063-156,194,798	AX-14943991
SGCD	rs73301129	chr5:155,135,063-156,194,798	AX-14943993
SGCD	rs75599360	chr5:155,135,063-156,194,798	AX-14943994
SGCD	rs76867717	chr5:155,135,063-156,194,798	AX-35170271
SGCD	rs32059	chr5:155,135,063-156,194,798	AX-41644915
SGCD	rs32058	chr5:155,135,063-156,194,798	AX-14943995
SGCD	rs10476477	chr5:155,135,063-156,194,798	AX-119518449
SGCD	rs10476477	chr5:155,135,063-156,194,798	AX-148458543
SGCD	rs73301135	chr5:155,135,063-156,194,798	AX-14943997
SGCD	rs41073	chr5:155,135,063-156,194,798	AX-14943998
SGCD	rs32057	chr5:155,135,063-156,194,798	AX-14944000
SGCD	rs434847	chr5:155,135,063-156,194,798	AX-41644919
SGCD	rs434116	chr5:155,135,063-156,194,798	AX-41644921
SGCD	rs446261	chr5:155,135,063-156,194,798	AX-113251394
SGCD	rs394183	chr5:155,135,063-156,194,798	AX-14944005
SGCD	rs452986	chr5:155,135,063-156,194,798	AX-41644925
SGCD	rs74617632	chr5:155,135,063-156,194,798	AX-156275745
SGCD	rs74617632	chr5:155,135,063-156,194,798	AX-156292857
SGCD	rs17053716	chr5:155,135,063-156,194,798	AX-41644927
SGCD	rs32056	chr5:155,135,063-156,194,798	AX-14944009
SGCD	rs39925	chr5:155,135,063-156,194,798	AX-12565395
SGCD	rs141437738	chr5:155,135,063-156,194,798	AX-120746105
SGCD	rs141437738	chr5:155,135,063-156,194,798	AX-156285954
SGCD	rs32055	chr5:155,135,063-156,194,798	AX-35170279
SGCD	rs39924	chr5:155,135,063-156,194,798	AX-35170283
SGCD	rs32054	chr5:155,135,063-156,194,798	AX-14944015
SGCD	rs460462	chr5:155,135,063-156,194,798	AX-35170285
SGCD	rs462375	chr5:155,135,063-156,194,798	AX-148493640
SGCD	rs462375	chr5:155,135,063-156,194,798	AX-156275746
SGCD	rs7705897	chr5:155,135,063-156,194,798	AX-156275747
SGCD	rs7705897	chr5:155,135,063-156,194,798	AX-98051292
SGCD	rs7721216	chr5:155,135,063-156,194,798	AX-105049721
SGCD	rs7721216	chr5:155,135,063-156,194,798	AX-88316465
SGCD	rs7709957	chr5:155,135,063-156,194,798	AX-148092320
SGCD	rs7709957	chr5:155,135,063-156,194,798	AX-148705168
SGCD	rs9314041	chr5:155,135,063-156,194,798	AX-120016694
SGCD	rs9314041	chr5:155,135,063-156,194,798	AX-156275748
SGCD	rs10491464	chr5:155,135,063-156,194,798	AX-11110442
SGCD	rs138183547	chr5:155,135,063-156,194,798	AX-156286311
SGCD	rs10077414	chr5:155,135,063-156,194,798	AX-156268287
SGCD	rs10077414	chr5:155,135,063-156,194,798	AX-156288114
SGCD	---	chr5:155,135,063-156,194,798	AX-151423345
SGCD	---	chr5:155,135,063-156,194,798	AX-156268288
SGCD	rs415118	chr5:155,135,063-156,194,798	AX-156268289
SGCD	rs415118	chr5:155,135,063-156,194,798	AX-156288115
SGCD	rs449858	chr5:155,135,063-156,194,798	AX-156282368
SGCD	rs408310	chr5:155,135,063-156,194,798	AX-148482969
SGCD	rs408310	chr5:155,135,063-156,194,798	AX-156282369
SGCD	rs693752	chr5:155,135,063-156,194,798	AX-156282370
SGCD	rs693752	chr5:155,135,063-156,194,798	AX-156297002
SGCD	rs4704806	chr5:155,135,063-156,194,798	AX-122200372

SGCD	rs4704806	chr5:155,135,063-156,194,798	AX-148740382
SGCD	rs182211	chr5:155,135,063-156,194,798	AX-148757760
SGCD	rs182211	chr5:155,135,063-156,194,798	AX-156282371
SGCD	rs73301167	chr5:155,135,063-156,194,798	AX-148816037
SGCD	rs73301167	chr5:155,135,063-156,194,798	AX-156282372
SGCD	rs7731165	chr5:155,135,063-156,194,798	AX-35170299
SGCD	rs112277348	chr5:155,135,063-156,194,798	AX-148812347
SGCD	rs112277348	chr5:155,135,063-156,194,798	AX-156275749
SGCD	rs281037	chr5:155,135,063-156,194,798	AX-14944018
SGCD	rs139192914	chr5:155,135,063-156,194,798	AX-122395384
SGCD	rs281038	chr5:155,135,063-156,194,798	AX-35170301
SGCD	rs111884528	chr5:155,135,063-156,194,798	AX-14944019
SGCD	rs281039	chr5:155,135,063-156,194,798	AX-14944021
SGCD	rs73301172	chr5:155,135,063-156,194,798	AX-14944022
SGCD	rs281040	chr5:155,135,063-156,194,798	AX-14944023
SGCD	rs281041	chr5:155,135,063-156,194,798	AX-14944024
SGCD	rs281042	chr5:155,135,063-156,194,798	AX-148231331
SGCD	rs79759527	chr5:155,135,063-156,194,798	AX-14944026
SGCD	rs281044	chr5:155,135,063-156,194,798	AX-14944027
SGCD	rs73301180	chr5:155,135,063-156,194,798	AX-156275751
SGCD	rs73301180	chr5:155,135,063-156,194,798	AX-156292859
SGCD	rs77616909	chr5:155,135,063-156,194,798	AX-14944030
SGCD	rs281045	chr5:155,135,063-156,194,798	AX-14944031
SGCD	rs281046	chr5:155,135,063-156,194,798	AX-156292860
SGCD	rs188130	chr5:155,135,063-156,194,798	AX-14944033
SGCD	rs147682890	chr5:155,135,063-156,194,798	AX-148825385
SGCD	rs147682890	chr5:155,135,063-156,194,798	AX-156284139
SGCD	rs199499900	chr5:155,135,063-156,194,798	AX-151394911
SGCD	rs199499900	chr5:155,135,063-156,194,798	AX-156286248
SGCD	rs157319	chr5:155,135,063-156,194,798	AX-148395768
SGCD	rs157319	chr5:155,135,063-156,194,798	AX-156275753
SGCD	rs281049	chr5:155,135,063-156,194,798	AX-148235991
SGCD	rs281049	chr5:155,135,063-156,194,798	AX-156275754
SGCD	rs73301187	chr5:155,135,063-156,194,798	AX-148816565
SGCD	rs73301187	chr5:155,135,063-156,194,798	AX-156275755
SGCD	rs59915775	chr5:155,135,063-156,194,798	AX-35170325
SGCD	rs111309403	chr5:155,135,063-156,194,798	AX-14944043
SGCD	rs58622977	chr5:155,135,063-156,194,798	AX-35170335
SGCD	rs77271234	chr5:155,135,063-156,194,798	AX-14944048
SGCD	rs281053	chr5:155,135,063-156,194,798	AX-14944049
SGCD	rs11955617	chr5:155,135,063-156,194,798	AX-151423575
SGCD	rs11955617	chr5:155,135,063-156,194,798	AX-156275756
SGCD	rs73301193	chr5:155,135,063-156,194,798	AX-14944051
SGCD	rs73301195	chr5:155,135,063-156,194,798	AX-14944052
SGCD	rs32082	chr5:155,135,063-156,194,798	AX-114216071
SGCD	rs7706067	chr5:155,135,063-156,194,798	AX-14944054
SGCD	rs111719866	chr5:155,135,063-156,194,798	AX-14944056
SGCD	rs113687593	chr5:155,135,063-156,194,798	AX-14944059
SGCD	rs41075	chr5:155,135,063-156,194,798	AX-14944061
SGCD	rs10056555	chr5:155,135,063-156,194,798	AX-156275757
SGCD	rs10056555	chr5:155,135,063-156,194,798	AX-156292861
SGCD	rs32080	chr5:155,135,063-156,194,798	AX-147852105
SGCD	rs32080	chr5:155,135,063-156,194,798	AX-84651646
SGCD	rs116052603	chr5:155,135,063-156,194,798	AX-14944066
SGCD	rs11744039	chr5:155,135,063-156,194,798	AX-14944067

SGCD	rs61610409	chr5:155,135,063-156,194,798	AX-14944068
SGCD	rs78786340	chr5:155,135,063-156,194,798	AX-14944076
SGCD	rs32078	chr5:155,135,063-156,194,798	AX-35170353
SGCD	rs32077	chr5:155,135,063-156,194,798	AX-14944083
SGCD	rs33912988	chr5:155,135,063-156,194,798	AX-119557099
SGCD	rs33912988	chr5:155,135,063-156,194,798	AX-156284846
SGCD	rs10071427	chr5:155,135,063-156,194,798	AX-35170355
SGCD	rs77014517	chr5:155,135,063-156,194,798	AX-14944086
SGCD	rs34853524	chr5:155,135,063-156,194,798	AX-41644951
SGCD	rs73302804	chr5:155,135,063-156,194,798	AX-14944087
SGCD	rs32076	chr5:155,135,063-156,194,798	AX-14944088
SGCD	rs32075	chr5:155,135,063-156,194,798	AX-14944089
SGCD	rs73302808	chr5:155,135,063-156,194,798	AX-14944090
SGCD	rs32073	chr5:155,135,063-156,194,798	AX-156282374
SGCD	rs32073	chr5:155,135,063-156,194,798	AX-156297003
SGCD	rs80000754	chr5:155,135,063-156,194,798	AX-14944092
SGCD	rs57380802	chr5:155,135,063-156,194,798	AX-14944093
SGCD	rs157671	chr5:155,135,063-156,194,798	AX-41644957
SGCD	rs73302810	chr5:155,135,063-156,194,798	AX-14944097
SGCD	rs157670	chr5:155,135,063-156,194,798	AX-14944098
SGCD	rs32072	chr5:155,135,063-156,194,798	AX-14944099
SGCD	rs32071	chr5:155,135,063-156,194,798	AX-156275758
SGCD	rs32071	chr5:155,135,063-156,194,798	AX-156292862
SGCD	rs7723731	chr5:155,135,063-156,194,798	AX-12638243
SGCD	rs32070	chr5:155,135,063-156,194,798	AX-88899428
SGCD	rs32070	chr5:155,135,063-156,194,798	AX-88899429
SGCD	rs32069	chr5:155,135,063-156,194,798	AX-156275759
SGCD	rs39927	chr5:155,135,063-156,194,798	AX-14944100
SGCD	rs73302817	chr5:155,135,063-156,194,798	AX-156275760
SGCD	rs73302817	chr5:155,135,063-156,194,798	AX-156292864
SGCD	rs32068	chr5:155,135,063-156,194,798	AX-14944102
SGCD	rs157331	chr5:155,135,063-156,194,798	AX-41644965
SGCD	rs73302819	chr5:155,135,063-156,194,798	AX-14944104
SGCD	rs39926	chr5:155,135,063-156,194,798	AX-156275761
SGCD	rs39926	chr5:155,135,063-156,194,798	AX-156292865
SGCD	rs73302821	chr5:155,135,063-156,194,798	AX-14944105
SGCD	rs17053760	chr5:155,135,063-156,194,798	AX-11301540
SGCD	rs10036419	chr5:155,135,063-156,194,798	AX-35170375
SGCD	rs157332	chr5:155,135,063-156,194,798	AX-113251395
SGCD	rs157332	chr5:155,135,063-156,194,798	AX-156275762
SGCD	rs157333	chr5:155,135,063-156,194,798	AX-14944109
SGCD	rs157334	chr5:155,135,063-156,194,798	AX-14944110
SGCD	rs157335	chr5:155,135,063-156,194,798	AX-14944111
SGCD	rs6890150	chr5:155,135,063-156,194,798	AX-14944113
SGCD	rs281054	chr5:155,135,063-156,194,798	AX-122673920
SGCD	rs281054	chr5:155,135,063-156,194,798	AX-148751630
SGCD	rs281055	chr5:155,135,063-156,194,798	AX-14944118
SGCD	rs5872479	chr5:155,135,063-156,194,798	AX-151398249
SGCD	rs5872479	chr5:155,135,063-156,194,798	AX-156297812
SGCD	rs281056	chr5:155,135,063-156,194,798	AX-156282375
SGCD	rs281056	chr5:155,135,063-156,194,798	AX-156297004
SGCD	rs79211336	chr5:155,135,063-156,194,798	AX-156275763
SGCD	rs79211336	chr5:155,135,063-156,194,798	AX-156292866
SGCD	rs281057	chr5:155,135,063-156,194,798	AX-156292867
SGCD	rs11953043	chr5:155,135,063-156,194,798	AX-41644977

SGCD	rs281058	chr5:155,135,063-156,194,798	AX-14944127
SGCD	rs32095	chr5:155,135,063-156,194,798	AX-14944129
SGCD	rs32094	chr5:155,135,063-156,194,798	AX-14944130
SGCD	rs10085029	chr5:155,135,063-156,194,798	AX-35170391
SGCD	---	chr5:155,135,063-156,194,798	AX-156284140
SGCD	rs39929	chr5:155,135,063-156,194,798	AX-156275765
SGCD	rs39929	chr5:155,135,063-156,194,798	AX-92438608
SGCD	rs468494	chr5:155,135,063-156,194,798	AX-92796838
SGCD	rs467530	chr5:155,135,063-156,194,798	AX-151313028
SGCD	rs157664	chr5:155,135,063-156,194,798	AX-14944134
SGCD	rs73302831	chr5:155,135,063-156,194,798	AX-14944137
SGCD	rs32091	chr5:155,135,063-156,194,798	AX-14944139
SGCD	rs32090	chr5:155,135,063-156,194,798	AX-14944140
SGCD	rs32089	chr5:155,135,063-156,194,798	AX-156275768
SGCD	rs32089	chr5:155,135,063-156,194,798	AX-156292868
SGCD	rs61231457	chr5:155,135,063-156,194,798	AX-156275769
SGCD	rs61231457	chr5:155,135,063-156,194,798	AX-156292869
SGCD	rs73302837	chr5:155,135,063-156,194,798	AX-14944143
SGCD	rs32088	chr5:155,135,063-156,194,798	AX-14944144
SGCD	rs73302841	chr5:155,135,063-156,194,798	AX-35170403
SGCD	rs32087	chr5:155,135,063-156,194,798	AX-14944149
SGCD	rs32086	chr5:155,135,063-156,194,798	AX-14944150
SGCD	rs144978766	chr5:155,135,063-156,194,798	AX-120701150
SGCD	rs144978766	chr5:155,135,063-156,194,798	AX-14944151
SGCD	rs80153581	chr5:155,135,063-156,194,798	AX-14944153
SGCD	rs75107684	chr5:155,135,063-156,194,798	AX-14944154
SGCD	rs39928	chr5:155,135,063-156,194,798	AX-11489012
SGCD	rs32085	chr5:155,135,063-156,194,798	AX-14944156
SGCD	rs73302848	chr5:155,135,063-156,194,798	AX-14944159
SGCD	rs32084	chr5:155,135,063-156,194,798	AX-11436994
SGCD	rs32083	chr5:155,135,063-156,194,798	AX-12549834
SGCD	rs73302852	chr5:155,135,063-156,194,798	AX-14944163
SGCD	rs157336	chr5:155,135,063-156,194,798	AX-11276921
SGCD	rs157337	chr5:155,135,063-156,194,798	AX-14944167
SGCD	rs17053816	chr5:155,135,063-156,194,798	AX-120572950
SGCD	rs17053816	chr5:155,135,063-156,194,798	AX-156292870
SGCD	rs157338	chr5:155,135,063-156,194,798	AX-156275770
SGCD	rs157339	chr5:155,135,063-156,194,798	AX-14944168
SGCD	rs157340	chr5:155,135,063-156,194,798	AX-156275771
SGCD	rs281060	chr5:155,135,063-156,194,798	AX-14944171
SGCD	rs75252927	chr5:155,135,063-156,194,798	AX-14944173
SGCD	rs116174051	chr5:155,135,063-156,194,798	AX-14944174
SGCD	rs157341	chr5:155,135,063-156,194,798	AX-14944176
SGCD	rs157342	chr5:155,135,063-156,194,798	AX-41645001
SGCD	rs157343	chr5:155,135,063-156,194,798	AX-41645005
SGCD	rs17053824	chr5:155,135,063-156,194,798	AX-41645007
SGCD	rs157344	chr5:155,135,063-156,194,798	AX-156275772
SGCD	rs157345	chr5:155,135,063-156,194,798	AX-156275773
SGCD	rs157345	chr5:155,135,063-156,194,798	AX-156292873
SGCD	rs141243873	chr5:155,135,063-156,194,798	AX-151433200
SGCD	rs141243873	chr5:155,135,063-156,194,798	AX-156284936
SGCD	rs157346	chr5:155,135,063-156,194,798	AX-41645013
SGCD	rs10706643	chr5:155,135,063-156,194,798	AX-156282376
SGCD	rs157347	chr5:155,135,063-156,194,798	AX-41645017
SGCD	rs157348	chr5:155,135,063-156,194,798	AX-14944183

SGCD	rs76116452	chr5:155,135,063-156,194,798	AX-35170423
SGCD	rs157349	chr5:155,135,063-156,194,798	AX-14944185
SGCD	rs139685817	chr5:155,135,063-156,194,798	AX-151420497
SGCD	rs139685817	chr5:155,135,063-156,194,798	AX-156284779
SGCD	rs73302871	chr5:155,135,063-156,194,798	AX-14944188
SGCD	rs157350	chr5:155,135,063-156,194,798	AX-14944189
SGCD	rs153843	chr5:155,135,063-156,194,798	AX-14944190
SGCD	rs157351	chr5:155,135,063-156,194,798	AX-35170427
SGCD	rs467770	chr5:155,135,063-156,194,798	AX-113251396
SGCD	rs157352	chr5:155,135,063-156,194,798	AX-14944196
SGCD	rs157353	chr5:155,135,063-156,194,798	AX-114216074
SGCD	rs157354	chr5:155,135,063-156,194,798	AX-14944198
SGCD	rs10515741	chr5:155,135,063-156,194,798	AX-11114994
SGCD	rs282474	chr5:155,135,063-156,194,798	AX-11412866
SGCD	rs116302240	chr5:155,135,063-156,194,798	AX-35170437
SGCD	rs73302881	chr5:155,135,063-156,194,798	AX-14944199
SGCD	rs78302942	chr5:155,135,063-156,194,798	AX-14944201
SGCD	rs17561376	chr5:155,135,063-156,194,798	AX-12501187
SGCD	rs80065492	chr5:155,135,063-156,194,798	AX-14944204
SGCD	rs114680992	chr5:155,135,063-156,194,798	AX-14944205
SGCD	rs72803018	chr5:155,135,063-156,194,798	AX-156275775
SGCD	rs157676	chr5:155,135,063-156,194,798	AX-41645025
SGCD	rs72803019	chr5:155,135,063-156,194,798	AX-14944209
SGCD	rs157677	chr5:155,135,063-156,194,798	AX-14944210
SGCD	rs11749404	chr5:155,135,063-156,194,798	AX-14944211
SGCD	rs157678	chr5:155,135,063-156,194,798	AX-123095361
SGCD	rs75317960	chr5:155,135,063-156,194,798	AX-156275776
SGCD	rs75317960	chr5:155,135,063-156,194,798	AX-156292876
SGCD	rs116148515	chr5:155,135,063-156,194,798	AX-148612785
SGCD	rs116148515	chr5:155,135,063-156,194,798	AX-156275777
SGCD	rs11948514	chr5:155,135,063-156,194,798	AX-148464293
SGCD	rs11948514	chr5:155,135,063-156,194,798	AX-156275778
SGCD	rs149131739	chr5:155,135,063-156,194,798	AX-156285478
SGCD	rs157488	chr5:155,135,063-156,194,798	AX-156275779
SGCD	rs282475	chr5:155,135,063-156,194,798	AX-114328035
SGCD	rs282475	chr5:155,135,063-156,194,798	AX-123105193
SGCD	rs73302892	chr5:155,135,063-156,194,798	AX-14944214
SGCD	rs282465	chr5:155,135,063-156,194,798	AX-12538811
SGCD	rs157679	chr5:155,135,063-156,194,798	AX-12470382
SGCD	rs157680	chr5:155,135,063-156,194,798	AX-156282377
SGCD	rs157680	chr5:155,135,063-156,194,798	AX-156297005
SGCD	rs17053833	chr5:155,135,063-156,194,798	AX-12487926
SGCD	rs79857689	chr5:155,135,063-156,194,798	AX-38254501
SGCD	rs73302901	chr5:155,135,063-156,194,798	AX-156292877
SGCD	rs17053838	chr5:155,135,063-156,194,798	AX-41645037
SGCD	rs67453120	chr5:155,135,063-156,194,798	AX-14944222
SGCD	rs459055	chr5:155,135,063-156,194,798	AX-12575369
SGCD	rs17053847	chr5:155,135,063-156,194,798	AX-14944228
SGCD	rs2642660	chr5:155,135,063-156,194,798	AX-41645045
SGCD	rs67059134	chr5:155,135,063-156,194,798	AX-14944229
SGCD	rs73304909	chr5:155,135,063-156,194,798	AX-35170463
SGCD	rs282466	chr5:155,135,063-156,194,798	AX-11412842
SGCD	rs2652324	chr5:155,135,063-156,194,798	AX-14944230
SGCD	rs466781	chr5:155,135,063-156,194,798	AX-14944233
SGCD	rs463728	chr5:155,135,063-156,194,798	AX-35170469

SGCD	rs72803027	chr5:155,135,063-156,194,798	AX-156275781
SGCD	rs17053865	chr5:155,135,063-156,194,798	AX-14944234
SGCD	rs35575932	chr5:155,135,063-156,194,798	AX-14944235
SGCD	rs113473126	chr5:155,135,063-156,194,798	AX-156275782
SGCD	rs462058	chr5:155,135,063-156,194,798	AX-14944236
SGCD	rs456290	chr5:155,135,063-156,194,798	AX-12574816
SGCD	rs73304925	chr5:155,135,063-156,194,798	AX-14944237
SGCD	rs72803029	chr5:155,135,063-156,194,798	AX-151336233
SGCD	---	chr5:155,135,063-156,194,798	AX-14944238
SGCD	rs10515742	chr5:155,135,063-156,194,798	AX-14944239
SGCD	rs10515743	chr5:155,135,063-156,194,798	AX-14944241
SGCD	rs284443	chr5:155,135,063-156,194,798	AX-123059551
SGCD	rs284443	chr5:155,135,063-156,194,798	AX-123059552
SGCD	rs284444	chr5:155,135,063-156,194,798	AX-14944242
SGCD	rs112812694	chr5:155,135,063-156,194,798	AX-14944244
SGCD	rs283098	chr5:155,135,063-156,194,798	AX-14944245
SGCD	rs55667497	chr5:155,135,063-156,194,798	AX-148604404
SGCD	rs55667497	chr5:155,135,063-156,194,798	AX-156282378
SGCD	rs11949511	chr5:155,135,063-156,194,798	AX-156282379
SGCD	rs11949511	chr5:155,135,063-156,194,798	AX-156297006
SGCD	rs76691977	chr5:155,135,063-156,194,798	AX-156275784
SGCD	rs76691977	chr5:155,135,063-156,194,798	AX-156292880
SGCD	rs17053873	chr5:155,135,063-156,194,798	AX-12487931
SGCD	rs141400817	chr5:155,135,063-156,194,798	AX-151408710
SGCD	rs141400817	chr5:155,135,063-156,194,798	AX-156298309
SGCD	rs62382382	chr5:155,135,063-156,194,798	AX-14944256
SGCD	rs464992	chr5:155,135,063-156,194,798	AX-11515129
SGCD	rs61663045	chr5:155,135,063-156,194,798	AX-14944257
SGCD	rs284442	chr5:155,135,063-156,194,798	AX-11417102
SGCD	rs72803033	chr5:155,135,063-156,194,798	AX-14944259
SGCD	rs172240	chr5:155,135,063-156,194,798	AX-95859937
SGCD	rs10515744	chr5:155,135,063-156,194,798	AX-14944262
SGCD	rs284441	chr5:155,135,063-156,194,798	AX-14944263
SGCD	rs61495230	chr5:155,135,063-156,194,798	AX-35170495
SGCD	rs11955986	chr5:155,135,063-156,194,798	AX-14944267
SGCD	rs5872483	chr5:155,135,063-156,194,798	AX-12591354
SGCD	rs73304939	chr5:155,135,063-156,194,798	AX-14944269
SGCD	rs284440	chr5:155,135,063-156,194,798	AX-11394897
SGCD	rs10515745	chr5:155,135,063-156,194,798	AX-14944270
SGCD	rs284439	chr5:155,135,063-156,194,798	AX-14944273
SGCD	rs59605435	chr5:155,135,063-156,194,798	AX-156275785
SGCD	rs59605435	chr5:155,135,063-156,194,798	AX-156292881
SGCD	rs3969608	chr5:155,135,063-156,194,798	AX-156275786
SGCD	rs57805501	chr5:155,135,063-156,194,798	AX-151382040
SGCD	rs57805501	chr5:155,135,063-156,194,798	AX-156284786
SGCD	rs150518478	chr5:155,135,063-156,194,798	AX-121641265
SGCD	rs73304945	chr5:155,135,063-156,194,798	AX-14944276
SGCD	rs201553093	chr5:155,135,063-156,194,798	AX-121800053
SGCD	rs201553093	chr5:155,135,063-156,194,798	AX-156286407
SGCD	rs66744889	chr5:155,135,063-156,194,798	AX-14944279
SGCD	rs56297325	chr5:155,135,063-156,194,798	AX-14944283
SGCD	rs151071457	chr5:155,135,063-156,194,798	AX-156286370
SGCD	rs113570565	chr5:155,135,063-156,194,798	AX-151274314
SGCD	rs113570565	chr5:155,135,063-156,194,798	AX-156297789
SGCD	rs10050975	chr5:155,135,063-156,194,798	AX-122534326

SGCD	rs10050975	chr5:155,135,063-156,194,798	AX-151324842
SGCD	rs59428757	chr5:155,135,063-156,194,798	AX-148406279
SGCD	rs59428757	chr5:155,135,063-156,194,798	AX-156275787
SGCD	rs3857411	chr5:155,135,063-156,194,798	AX-14944296
SGCD	rs961272	chr5:155,135,063-156,194,798	AX-41645065
SGCD	rs3898659	chr5:155,135,063-156,194,798	AX-156275788
SGCD	rs3898659	chr5:155,135,063-156,194,798	AX-156292883
SGCD	rs73304951	chr5:155,135,063-156,194,798	AX-156275789
SGCD	rs73304951	chr5:155,135,063-156,194,798	AX-156292884
SGCD	rs11135380	chr5:155,135,063-156,194,798	AX-14944298
SGCD	rs11135381	chr5:155,135,063-156,194,798	AX-14944299
SGCD	rs11135382	chr5:155,135,063-156,194,798	AX-14944300
SGCD	rs11135383	chr5:155,135,063-156,194,798	AX-156275790
SGCD	rs11135383	chr5:155,135,063-156,194,798	AX-156292885
SGCD	rs57838784	chr5:155,135,063-156,194,798	AX-14944301
SGCD	rs57452961	chr5:155,135,063-156,194,798	AX-14944302
SGCD	rs57399099	chr5:155,135,063-156,194,798	AX-14944303
SGCD	rs71723387	chr5:155,135,063-156,194,798	AX-156285564
SGCD	rs12110214	chr5:155,135,063-156,194,798	AX-14944307
SGCD	rs3215118	chr5:155,135,063-156,194,798	AX-88767570
SGCD	rs3215118	chr5:155,135,063-156,194,798	AX-88810615
SGCD	---	chr5:155,135,063-156,194,798	AX-90030721
SGCD	rs145887064	chr5:155,135,063-156,194,798	AX-151315117
SGCD	rs145887064	chr5:155,135,063-156,194,798	AX-156284602
SGCD	rs1494542	chr5:155,135,063-156,194,798	AX-14944312
SGCD	rs3913020	chr5:155,135,063-156,194,798	AX-156275791
SGCD	rs3913020	chr5:155,135,063-156,194,798	AX-156292886
SGCD	rs72803039	chr5:155,135,063-156,194,798	AX-14944314
SGCD	rs72803040	chr5:155,135,063-156,194,798	AX-14944317
SGCD	rs6886827	chr5:155,135,063-156,194,798	AX-12613182
SGCD	rs3857412	chr5:155,135,063-156,194,798	AX-14944318
SGCD	rs72803041	chr5:155,135,063-156,194,798	AX-14944319
SGCD	rs284445	chr5:155,135,063-156,194,798	AX-14944320
SGCD	rs1389818	chr5:155,135,063-156,194,798	AX-11259029
SGCD	rs72803042	chr5:155,135,063-156,194,798	AX-14944322
SGCD	rs56293630	chr5:155,135,063-156,194,798	AX-14944323
SGCD	rs72803043	chr5:155,135,063-156,194,798	AX-14944324
SGCD	rs72803044	chr5:155,135,063-156,194,798	AX-14944325
SGCD	rs72803046	chr5:155,135,063-156,194,798	AX-14944326
SGCD	rs3842051	chr5:155,135,063-156,194,798	AX-11484201
SGCD	rs3842051	chr5:155,135,063-156,194,798	AX-156284590
SGCD	rs56182215	chr5:155,135,063-156,194,798	AX-14944328
SGCD	rs56389540	chr5:155,135,063-156,194,798	AX-35170537
SGCD	rs55653598	chr5:155,135,063-156,194,798	AX-14944331
SGCD	rs73304958	chr5:155,135,063-156,194,798	AX-14944333
SGCD	rs1845479	chr5:155,135,063-156,194,798	AX-11346701
SGCD	rs3913482	chr5:155,135,063-156,194,798	AX-11487282
SGCD	rs11953631	chr5:155,135,063-156,194,798	AX-14944335
SGCD	rs72803047	chr5:155,135,063-156,194,798	AX-14944337
SGCD	rs61633788	chr5:155,135,063-156,194,798	AX-14944338
SGCD	rs1827368	chr5:155,135,063-156,194,798	AX-12507016
SGCD	rs72803048	chr5:155,135,063-156,194,798	AX-14944340
SGCD	rs56233900	chr5:155,135,063-156,194,798	AX-14944342
SNTA1	---	chr20:31,995,763-32,031,698	AX-32916179
SNTA1	rs56157422	chr20:31,995,763-32,031,698	AX-83446209

SNTA1	rs657040	chr20:31,995,763-32,031,698	AX-40529419
TAZ	---	chrX:153,639,877-153,650,063	AX-90080648
TAZ	---	chrX:153,639,877-153,650,063	AX-90058641
TAZ	---	chrX:153,639,877-153,650,063	AX-90035485
TAZ	---	chrX:153,639,877-153,650,063	AX-37208805
TAZ	---	chrX:153,639,877-153,650,063	AX-90060737
TAZ	---	chrX:153,639,877-153,650,063	AX-90062427
TAZ	---	chrX:153,639,877-153,650,063	AX-90077480
TAZ	---	chrX:153,639,877-153,650,063	AX-90056031
TAZ	---	chrX:153,639,877-153,650,063	AX-90050486
TAZ	---	chrX:153,639,877-153,650,063	AX-90057035
TAZ	rs104894941	chrX:153,639,877-153,650,063	AX-88800921
TAZ	---	chrX:153,639,877-153,650,063	AX-90077214
TAZ	---	chrX:153,639,877-153,650,063	AX-90033191
TAZ	---	chrX:153,639,877-153,650,063	AX-90057793
TAZ	---	chrX:153,639,877-153,650,063	AX-90054566
TAZ	---	chrX:153,639,877-153,650,063	AX-90060595
TAZ	---	chrX:153,639,877-153,650,063	AX-90025682
TAZ	---	chrX:153,639,877-153,650,063	AX-90031622
TAZ	---	chrX:153,639,877-153,650,063	AX-90025683
TAZ	---	chrX:153,639,877-153,650,063	AX-90031746
TAZ	---	chrX:153,639,877-153,650,063	AX-90037431
TAZ	---	chrX:153,639,877-153,650,063	AX-90027739
TAZ	---	chrX:153,639,877-153,650,063	AX-90043914
TAZ	---	chrX:153,639,877-153,650,063	AX-90025684
TAZ	rs104894942	chrX:153,639,877-153,650,063	AX-88757877
TAZ	---	chrX:153,639,877-153,650,063	AX-90062816
TAZ	---	chrX:153,639,877-153,650,063	AX-90025685
TAZ	---	chrX:153,639,877-153,650,063	AX-90060006
TAZ	---	chrX:153,639,877-153,650,063	AX-90066262
TAZ	---	chrX:153,639,877-153,650,063	AX-90048971
TAZ	rs397515739	chrX:153,639,877-153,650,063	AX-90035660
TAZ	---	chrX:153,639,877-153,650,063	AX-90035538
TAZ	---	chrX:153,639,877-153,650,063	AX-90057452
TAZ	rs104894937	chrX:153,639,877-153,650,063	AX-90071408
TAZ	---	chrX:153,639,877-153,650,063	AX-90054572
TAZ	---	chrX:153,639,877-153,650,063	AX-90058513
TAZ	---	chrX:153,639,877-153,650,063	AX-90056579
TAZ	---	chrX:153,639,877-153,650,063	AX-90035440
TAZ	---	chrX:153,639,877-153,650,063	AX-90063903
TAZ	---	chrX:153,639,877-153,650,063	AX-90074563
TAZ	---	chrX:153,639,877-153,650,063	AX-90025688
TAZ	---	chrX:153,639,877-153,650,063	AX-90048634
TAZ	---	chrX:153,639,877-153,650,063	AX-90031378
TAZ	---	chrX:153,639,877-153,650,063	AX-90080527
TAZ	---	chrX:153,639,877-153,650,063	AX-90025689
TAZ	---	chrX:153,639,877-153,650,063	AX-90025690
TAZ	---	chrX:153,639,877-153,650,063	AX-90048436
TAZ	---	chrX:153,639,877-153,650,063	AX-90059147
TAZ	---	chrX:153,639,877-153,650,063	AX-90032769
TAZ	rs132630277	chrX:153,639,877-153,650,063	AX-88801113
TAZ	rs397515746	chrX:153,639,877-153,650,063	AX-90054576
TAZ	---	chrX:153,639,877-153,650,063	AX-90049557
TAZ	---	chrX:153,639,877-153,650,063	AX-90079420
TAZ	---	chrX:153,639,877-153,650,063	AX-90025692

TAZ	---	chrX:153,639,877-153,650,063	AX-90025693
TAZ	---	chrX:153,639,877-153,650,063	AX-90061897
TAZ	---	chrX:153,639,877-153,650,063	AX-90036553
TAZ	---	chrX:153,639,877-153,650,063	AX-90079674
TAZ	---	chrX:153,639,877-153,650,063	AX-90072296
TAZ	---	chrX:153,639,877-153,650,063	AX-90054581
TAZ	---	chrX:153,639,877-153,650,063	AX-90080591
TAZ	---	chrX:153,639,877-153,650,063	AX-90060565
TAZ	---	chrX:153,639,877-153,650,063	AX-90027062
TAZ	---	chrX:153,639,877-153,650,063	AX-90027865
TAZ	---	chrX:153,639,877-153,650,063	AX-90062039
TAZ	rs397515750	chrX:153,639,877-153,650,063	AX-90057350
TAZ	---	chrX:153,639,877-153,650,063	AX-90071764
TAZ	---	chrX:153,639,877-153,650,063	AX-90074064
TCAP	rs45495192	chr17:37,821,599-37,822,807	AX-83517234
TCAP	---	chr17:37,821,599-37,822,807	AX-90027554
TCAP	---	chr17:37,821,599-37,822,807	AX-90052504
TCAP	rs104894655	chr17:37,821,599-37,822,807	AX-90041219
TCAP	---	chr17:37,821,599-37,822,807	AX-90031033
TCAP	rs45578741	chr17:37,821,599-37,822,807	AX-83038041
TCAP	---	chr17:37,821,599-37,822,807	AX-90060581
TCAP	---	chr17:37,821,599-37,822,807	AX-90026759
TCAP	rs1053651	chr17:37,821,599-37,822,807	AX-40175283
TMEM43	rs2733584	chr3:14,166,440-14,185,180	AX-41124411
TMEM43	rs34099410	chr3:14,166,440-14,185,180	AX-83457763
TMEM43	rs145619906	chr3:14,166,440-14,185,180	AX-82967020
TMEM43	rs4685076	chr3:14,166,440-14,185,180	AX-41124499
TMEM43	rs2340917	chr3:14,166,440-14,185,180	AX-11385825
TMEM43	rs6766740	chr3:14,166,440-14,185,180	AX-41124531
TMEM43	rs113449357	chr3:14,166,440-14,185,180	AX-83556072
TMEM43	rs11943	chr3:14,166,440-14,185,180	AX-34081005
TMEM43	rs1043953	chr3:14,166,440-14,185,180	AX-41124725
TMPO	rs190115355	chr12:98,909,351-98,944,157	AX-156288775
TMPO	rs190115355	chr12:98,909,351-98,944,157	AX-84963193
TMPO	rs200177466	chr12:98,909,351-98,944,157	AX-83300229
TMPO	rs249825	chr12:98,909,351-98,944,157	AX-39536843
TMPO	rs11829793	chr12:98,909,351-98,944,157	AX-120002470
TMPO	rs11829793	chr12:98,909,351-98,944,157	AX-156266442
TMPO	rs11109509	chr12:98,909,351-98,944,157	AX-17087735
TMPO	rs11109510	chr12:98,909,351-98,944,157	AX-30949425
TMPO	rs73136485	chr12:98,909,351-98,944,157	AX-30949427
TMPO	rs113882661	chr12:98,909,351-98,944,157	AX-123075025
TMPO	rs113882661	chr12:98,909,351-98,944,157	AX-123075026
TMPO	rs111966040	chr12:98,909,351-98,944,157	AX-151268127
TMPO	rs111966040	chr12:98,909,351-98,944,157	AX-153464093
TMPO	rs4394881	chr12:98,909,351-98,944,157	AX-156266443
TMPO	rs4394881	chr12:98,909,351-98,944,157	AX-156286955
TMPO	rs1420446	chr12:98,909,351-98,944,157	AX-39536849
TMPO	rs35183227	chr12:98,909,351-98,944,157	AX-151358810
TMPO	rs113822789	chr12:98,909,351-98,944,157	AX-94348268
TMPO	rs73136486	chr12:98,909,351-98,944,157	AX-151306777
TMPO	rs73136486	chr12:98,909,351-98,944,157	AX-156280224
TMPO	rs111441521	chr12:98,909,351-98,944,157	AX-148521570
TMPO	rs7135186	chr12:98,909,351-98,944,157	AX-119722104
TMPO	rs7135937	chr12:98,909,351-98,944,157	AX-148041185

TMPO	rs7135937	chr12:98,909,351-98,944,157	AX-156271274
TMPO	rs142925974	chr12:98,909,351-98,944,157	AX-106803762
TMPO	rs142925974	chr12:98,909,351-98,944,157	AX-156285544
TMPO	rs7309364	chr12:98,909,351-98,944,157	AX-148068876
TMPO	rs7309364	chr12:98,909,351-98,944,157	AX-156271275
TMPO	rs77639919	chr12:98,909,351-98,944,157	AX-148659810
TMPO	rs77639919	chr12:98,909,351-98,944,157	AX-156266444
TMPO	rs12581633	chr12:98,909,351-98,944,157	AX-156266445
TMPO	rs12581633	chr12:98,909,351-98,944,157	AX-156286956
TMPO	rs11109512	chr12:98,909,351-98,944,157	AX-39536853
TMPO	rs11109513	chr12:98,909,351-98,944,157	AX-148522679
TMPO	rs7973270	chr12:98,909,351-98,944,157	AX-151116247
TMPO	rs7973270	chr12:98,909,351-98,944,157	AX-156266446
TMPO	rs11837732	chr12:98,909,351-98,944,157	AX-156266447
TMPO	rs11837732	chr12:98,909,351-98,944,157	AX-156286957
TMPO	rs11109515	chr12:98,909,351-98,944,157	AX-156266448
TMPO	rs78830853	chr12:98,909,351-98,944,157	AX-30949467
TMPO	rs73382588	chr12:98,909,351-98,944,157	AX-156266449
TMPO	rs7316636	chr12:98,909,351-98,944,157	AX-30949471
TMPO	rs12366275	chr12:98,909,351-98,944,157	AX-156266450
TMPO	rs12366275	chr12:98,909,351-98,944,157	AX-156286959
TMPO	rs11109516	chr12:98,909,351-98,944,157	AX-148522691
TMPO	rs11109516	chr12:98,909,351-98,944,157	AX-156280225
TMPO	rs199946287	chr12:98,909,351-98,944,157	AX-156285861
TMPO	rs10651296	chr12:98,909,351-98,944,157	AX-156284959
TMPO	rs7135956	chr12:98,909,351-98,944,157	AX-156280226
TMPO	rs7135956	chr12:98,909,351-98,944,157	AX-156295694
TMPO	rs12311323	chr12:98,909,351-98,944,157	AX-30949475
TMPO	rs7136406	chr12:98,909,351-98,944,157	AX-39536861
TMPO	rs35124796	chr12:98,909,351-98,944,157	AX-30949479
TMPO	rs11109517	chr12:98,909,351-98,944,157	AX-39536863
TMPO	rs111529998	chr12:98,909,351-98,944,157	AX-96968419
TMPO	rs10860339	chr12:98,909,351-98,944,157	AX-148007731
TMPO	rs10860339	chr12:98,909,351-98,944,157	AX-92722887
TMPO	rs7961024	chr12:98,909,351-98,944,157	AX-156280227
TMPO	rs7961024	chr12:98,909,351-98,944,157	AX-156295695
TMPO	rs35207802	chr12:98,909,351-98,944,157	AX-39536871
TMPO	rs36047490	chr12:98,909,351-98,944,157	AX-107770729
TMPO	rs36047490	chr12:98,909,351-98,944,157	AX-156280228
TMPO	rs7307828	chr12:98,909,351-98,944,157	AX-148441054
TMPO	rs7307828	chr12:98,909,351-98,944,157	AX-156266451
TMPO	rs112315612	chr12:98,909,351-98,944,157	AX-113732041
TMPO	rs61934004	chr12:98,909,351-98,944,157	AX-148042748
TMPO	rs61934004	chr12:98,909,351-98,944,157	AX-156266452
TMPO	rs3213901	chr12:98,909,351-98,944,157	AX-17087754
TMPO	rs3213900	chr12:98,909,351-98,944,157	AX-11437202
TMPO	rs146054750	chr12:98,909,351-98,944,157	AX-156298312
TMPO	rs73384409	chr12:98,909,351-98,944,157	AX-156266453
TMPO	rs138790561	chr12:98,909,351-98,944,157	AX-82961280
TMPO	rs139700737	chr12:98,909,351-98,944,157	AX-83370903
TMPO	rs35969221	chr12:98,909,351-98,944,157	AX-17087756
TMPO	rs141443652	chr12:98,909,351-98,944,157	AX-83282388
TMPO	rs17459334	chr12:98,909,351-98,944,157	AX-17087759
TMPO	---	chr12:98,909,351-98,944,157	AX-86584595
TMPO	rs17028450	chr12:98,909,351-98,944,157	AX-12486157

TMPO	rs11109520	chr12:98,909,351-98,944,157	AX-17087762
TMPO	rs17028456	chr12:98,909,351-98,944,157	AX-11299234
TMPO	rs11109521	chr12:98,909,351-98,944,157	AX-156266454
TMPO	rs11109521	chr12:98,909,351-98,944,157	AX-156286961
TMPO	rs11109522	chr12:98,909,351-98,944,157	AX-39536885
TMPO	rs34646198	chr12:98,909,351-98,944,157	AX-39536887
TMPO	rs12825374	chr12:98,909,351-98,944,157	AX-30949525
TMPO	rs140195963	chr12:98,909,351-98,944,157	AX-151448437
TMPO	rs140195963	chr12:98,909,351-98,944,157	AX-156285430
TMPO	rs11109523	chr12:98,909,351-98,944,157	AX-148524396
TMPO	rs11109523	chr12:98,909,351-98,944,157	AX-156266455
TMPO	rs36011276	chr12:98,909,351-98,944,157	AX-156286962
TMPO	rs4762494	chr12:98,909,351-98,944,157	AX-17087771
TMPO	rs4762495	chr12:98,909,351-98,944,157	AX-39536891
TMPO	rs4762496	chr12:98,909,351-98,944,157	AX-113551869
TMPO	rs4762496	chr12:98,909,351-98,944,157	AX-148522965
TMPO	rs58209561	chr12:98,909,351-98,944,157	AX-121432003
TMPO	rs149584406	chr12:98,909,351-98,944,157	AX-148264832
TMPO	rs149584406	chr12:98,909,351-98,944,157	AX-148752147
TMPO	rs12810190	chr12:98,909,351-98,944,157	AX-151295614
TMPO	rs12810190	chr12:98,909,351-98,944,157	AX-156269294
TMPO	rs10860341	chr12:98,909,351-98,944,157	AX-39536893
TMPO	rs7133258	chr12:98,909,351-98,944,157	AX-39536895
TMPO	rs200199961	chr12:98,909,351-98,944,157	AX-156285822
TMPO	rs201498123	chr12:98,909,351-98,944,157	AX-156286140
TMPO	rs2068060	chr12:98,909,351-98,944,157	AX-156266457
TMPO	rs2068060	chr12:98,909,351-98,944,157	AX-156286963
TMPO	rs11109524	chr12:98,909,351-98,944,157	AX-39536899
TMPO	rs60808759	chr12:98,909,351-98,944,157	AX-156266458
TMPO	rs34449077	chr12:98,909,351-98,944,157	AX-82939267
TMPO	rs4762208	chr12:98,909,351-98,944,157	AX-156266460
TMPO	rs10542097	chr12:98,909,351-98,944,157	AX-122370332
TMPO	rs10542097	chr12:98,909,351-98,944,157	AX-156297953
TMPO	rs114805943	chr12:98,909,351-98,944,157	AX-156266461
TMPO	rs10860342	chr12:98,909,351-98,944,157	AX-39536903
TMPO	rs28535939	chr12:98,909,351-98,944,157	AX-105094854
TMPO	rs28535939	chr12:98,909,351-98,944,157	AX-148037348
TMPO	rs147137090	chr12:98,909,351-98,944,157	AX-156269295
TMPO	rs112838469	chr12:98,909,351-98,944,157	AX-148446041
TMPO	rs112838469	chr12:98,909,351-98,944,157	AX-156266462
TMPO	---	chr12:98,909,351-98,944,157	AX-17087781
TNNC1	rs104893823	chr3:52,485,107-52,488,057	AX-90070849
TNNC1	rs267607124	chr3:52,485,107-52,488,057	AX-86578678
TNNC1	---	chr3:52,485,107-52,488,057	AX-90058541
TNNC1	rs267607126	chr3:52,485,107-52,488,057	AX-90026978
TNNC1	---	chr3:52,485,107-52,488,057	AX-90029297
TNNC1	---	chr3:52,485,107-52,488,057	AX-90033359
TNNC1	rs267607123	chr3:52,485,107-52,488,057	AX-90026979
TNNC1	rs72965257	chr3:52,485,107-52,488,057	AX-34346515
TNNC1	rs267607125	chr3:52,485,107-52,488,057	AX-90055865
TNNI3	rs104894725	chr19:55,663,136-55,669,100	AX-88754567
TNNI3	---	chr19:55,663,136-55,669,100	AX-90055149
TNNI3	---	chr19:55,663,136-55,669,100	AX-90055150
TNNI3	rs267607127	chr19:55,663,136-55,669,100	AX-90026266
TNNI3	---	chr19:55,663,136-55,669,100	AX-90031577

TNNI3	---	chr19:55,663,136-55,669,100	AX-90065723
TNNI3	---	chr19:55,663,136-55,669,100	AX-90036815
TNNI3	---	chr19:55,663,136-55,669,100	AX-90026267
TNNI3	---	chr19:55,663,136-55,669,100	AX-90055153
TNNI3	---	chr19:55,663,136-55,669,100	AX-90028768
TNNI3	rs104894729	chr19:55,663,136-55,669,100	AX-86894435
TNNI3	---	chr19:55,663,136-55,669,100	AX-90058440
TNNI3	rs397516357	chr19:55,663,136-55,669,100	AX-86894502
TNNI3	rs267607129	chr19:55,663,136-55,669,100	AX-90059262
TNNI3	rs397516356	chr19:55,663,136-55,669,100	AX-90060465
TNNI3	rs72301544	chr19:55,663,136-55,669,100	AX-122836764
TNNI3	rs72301544	chr19:55,663,136-55,669,100	AX-156297909
TNNI3	rs28591612	chr19:55,663,136-55,669,100	AX-32795767
TNNI3	rs78694716	chr19:55,663,136-55,669,100	AX-148483636
TNNI3	rs78694716	chr19:55,663,136-55,669,100	AX-156283236
TNNI3	rs117989514	chr19:55,663,136-55,669,100	AX-54836419
TNNI3	rs75062926	chr19:55,663,136-55,669,100	AX-156272763
TNNI3	rs75062926	chr19:55,663,136-55,669,100	AX-156290810
TNNI3	rs11671466	chr19:55,663,136-55,669,100	AX-92420765
TNNI3	rs11671466	chr19:55,663,136-55,669,100	AX-92631788
TNNI3	rs10414493	chr19:55,663,136-55,669,100	AX-32795771
TNNI3	rs10414696	chr19:55,663,136-55,669,100	AX-40475201
TNNI3	rs201521806	chr19:55,663,136-55,669,100	AX-151447803
TNNI3	rs201521806	chr19:55,663,136-55,669,100	AX-156286015
TNNI3	---	chr19:55,663,136-55,669,100	AX-90026269
TNNI3	rs397516355	chr19:55,663,136-55,669,100	AX-90036056
TNNI3	rs3729841	chr19:55,663,136-55,669,100	AX-50264634
TNNI3	---	chr19:55,663,136-55,669,100	AX-90073025
TNNI3	rs121917761	chr19:55,663,136-55,669,100	AX-88797681
TNNI3	---	chr19:55,663,136-55,669,100	AX-90026270
TNNI3	---	chr19:55,663,136-55,669,100	AX-90046430
TNNI3	---	chr19:55,663,136-55,669,100	AX-90026271
TNNI3	rs546367368	chr19:55,663,136-55,669,100	AX-90066022
TNNI3	---	chr19:55,663,136-55,669,100	AX-86626008
TNNI3	---	chr19:55,663,136-55,669,100	AX-90031238
TNNI3	rs368861241	chr19:55,663,136-55,669,100	AX-86700980
TNNI3	rs397516353	chr19:55,663,136-55,669,100	AX-86894503
TNNI3	rs397516349	chr19:55,663,136-55,669,100	AX-86668168
TNNI3	---	chr19:55,663,136-55,669,100	AX-90026273
TNNI3	---	chr19:55,663,136-55,669,100	AX-90041297
TNNI3	---	chr19:55,663,136-55,669,100	AX-90041369
TNNI3	---	chr19:55,663,136-55,669,100	AX-90055159
TNNI3	rs397516347	chr19:55,663,136-55,669,100	AX-86894456
TNNI3	---	chr19:55,663,136-55,669,100	AX-90029771
TNNI3	---	chr19:55,663,136-55,669,100	AX-90033897
TNNI3	---	chr19:55,663,136-55,669,100	AX-90026275
TNNI3	---	chr19:55,663,136-55,669,100	AX-90033952
TNNI3	rs140633085	chr19:55,663,136-55,669,100	AX-151441517
TNNI3	rs140633085	chr19:55,663,136-55,669,100	AX-156288798
TNNI3	rs62126324	chr19:55,663,136-55,669,100	AX-92411987
TNNI3	rs62126324	chr19:55,663,136-55,669,100	AX-96853954
TNNI3	rs62126325	chr19:55,663,136-55,669,100	AX-92507891
TNNI3	rs62126325	chr19:55,663,136-55,669,100	AX-92718914
TNNI3	---	chr19:55,663,136-55,669,100	AX-90028331
TNNI3	rs77615401	chr19:55,663,136-55,669,100	AX-32795783

TNNI3	---	chr19:55,663,136-55,669,100	AX-90026276
TNNI3	rs3729711	chr19:55,663,136-55,669,100	AX-40475209
TNNI3	rs3729710	chr19:55,663,136-55,669,100	AX-83480316
TNNI3	rs3729709	chr19:55,663,136-55,669,100	AX-40475213
TNNI3	rs73617692	chr19:55,663,136-55,669,100	AX-32795787
TNNI3	rs73617693	chr19:55,663,136-55,669,100	AX-32795789
TNNI3	rs28526132	chr19:55,663,136-55,669,100	AX-32795791
TNNI3	rs3729838	chr19:55,663,136-55,669,100	AX-40475221
TNNI3	---	chr19:55,663,136-55,669,100	AX-86693617
TNNI3	---	chr19:55,663,136-55,669,100	AX-90060270
TNNI3	rs3729836	chr19:55,663,136-55,669,100	AX-156265659
TNNI3	rs3729836	chr19:55,663,136-55,669,100	AX-92458744
TNNI3	rs113899745	chr19:55,663,136-55,669,100	AX-82885183
TNNI3	rs202087239	chr19:55,663,136-55,669,100	AX-92733012
TNNI3	rs11671293	chr19:55,663,136-55,669,100	AX-40475223
TNNI3	rs11667847	chr19:55,663,136-55,669,100	AX-32795797
TNNI3	rs3729708	chr19:55,663,136-55,669,100	AX-119441042
TNNI3	rs3729708	chr19:55,663,136-55,669,100	AX-97374470
TNNI3	rs397516359	chr19:55,663,136-55,669,100	AX-86691086
TNNI3	rs3729707	chr19:55,663,136-55,669,100	AX-156265660
TNNI3	rs3729707	chr19:55,663,136-55,669,100	AX-156265693
TNNT2	rs45576635	chr1:201,328,136-201,346,828	AX-30416733
TNNT2	---	chr1:201,328,136-201,346,828	AX-90065088
TNNT2	rs121964861	chr1:201,328,136-201,346,828	AX-88754924
TNNT2	rs397516483	chr1:201,328,136-201,346,828	AX-90063229
TNNT2	rs2275863	chr1:201,328,136-201,346,828	AX-39249945
TNNT2	rs45509695	chr1:201,328,136-201,346,828	AX-156270643
TNNT2	rs45509695	chr1:201,328,136-201,346,828	AX-156289409
TNNT2	rs7521796	chr1:201,328,136-201,346,828	AX-39249951
TNNT2	rs10920181	chr1:201,328,136-201,346,828	AX-39249953
TNNT2	rs12564577	chr1:201,328,136-201,346,828	AX-39249955
TNNT2	rs3730238	chr1:201,328,136-201,346,828	AX-39249961
TNNT2	---	chr1:201,328,136-201,346,828	AX-90032640
TNNT2	rs45466197	chr1:201,328,136-201,346,828	AX-83012305
TNNT2	rs2275861	chr1:201,328,136-201,346,828	AX-156279921
TNNT2	rs2275861	chr1:201,328,136-201,346,828	AX-156295480
TNNT2	rs3730240	chr1:201,328,136-201,346,828	AX-39249963
TNNT2	rs121964860	chr1:201,328,136-201,346,828	AX-88754923
TNNT2	rs11810834	chr1:201,328,136-201,346,828	AX-30416759
TNNT2	rs11807637	chr1:201,328,136-201,346,828	AX-39249965
TNNT2	rs11806184	chr1:201,328,136-201,346,828	AX-39249967
TNNT2	---	chr1:201,328,136-201,346,828	AX-90046193
TNNT2	rs1104859	chr1:201,328,136-201,346,828	AX-39249969
TNNT2	rs2365652	chr1:201,328,136-201,346,828	AX-39249973
TNNT2	rs1104858	chr1:201,328,136-201,346,828	AX-30416771
TNNT2	rs1892027	chr1:201,328,136-201,346,828	AX-39249975
TNNT2	---	chr1:201,328,136-201,346,828	AX-90054589
TNNT2	---	chr1:201,328,136-201,346,828	AX-90071828
TNNT2	rs397516471	chr1:201,328,136-201,346,828	AX-90033058
TNNT2	---	chr1:201,328,136-201,346,828	AX-90062823
TNNT2	---	chr1:201,328,136-201,346,828	AX-90064371
TNNT2	rs11808175	chr1:201,328,136-201,346,828	AX-156270644
TNNT2	rs11808175	chr1:201,328,136-201,346,828	AX-156289410
TNNT2	rs1892026	chr1:201,328,136-201,346,828	AX-39249989
TNNT2	rs1892025	chr1:201,328,136-201,346,828	AX-123024761

TNNT2	rs2001410	chr1:201,328,136-201,346,828	AX-30416777
TNNT2	rs201048783	chr1:201,328,136-201,346,828	AX-90068398
TNNT2	rs483352832	chr1:201,328,136-201,346,828	AX-90054591
TNNT2	---	chr1:201,328,136-201,346,828	AX-90025707
TNNT2	---	chr1:201,328,136-201,346,828	AX-86618914
TNNT2	---	chr1:201,328,136-201,346,828	AX-86894430
TNNT2	rs397516466	chr1:201,328,136-201,346,828	AX-90025708
TNNT2	---	chr1:201,328,136-201,346,828	AX-90062975
TNNT2	rs74315380	chr1:201,328,136-201,346,828	AX-88754804
TNNT2	rs3767546	chr1:201,328,136-201,346,828	AX-156270645
TNNT2	rs3767546	chr1:201,328,136-201,346,828	AX-156289411
TNNT2	rs10920182	chr1:201,328,136-201,346,828	AX-39249999
TNNT2	---	chr1:201,328,136-201,346,828	AX-90054595
TNNT2	---	chr1:201,328,136-201,346,828	AX-90054596
TNNT2	---	chr1:201,328,136-201,346,828	AX-90025712
TNNT2	rs3729547	chr1:201,328,136-201,346,828	AX-11476389
TNNT2	---	chr1:201,328,136-201,346,828	AX-90054598
TNNT2	---	chr1:201,328,136-201,346,828	AX-90078005
TNNT2	rs397516459	chr1:201,328,136-201,346,828	AX-90025714
TNNT2	---	chr1:201,328,136-201,346,828	AX-90048032
TNNT2	---	chr1:201,328,136-201,346,828	AX-90025715
TNNT2	---	chr1:201,328,136-201,346,828	AX-90054601
TNNT2	---	chr1:201,328,136-201,346,828	AX-90025717
TNNT2	rs397516456	chr1:201,328,136-201,346,828	AX-86894453
TNNT2	---	chr1:201,328,136-201,346,828	AX-90025718
TNNT2	---	chr1:201,328,136-201,346,828	AX-90054604
TNNT2	rs397516455	chr1:201,328,136-201,346,828	AX-90025720
TNNT2	---	chr1:201,328,136-201,346,828	AX-90025721
TNNT2	---	chr1:201,328,136-201,346,828	AX-86548045
TNNT2	---	chr1:201,328,136-201,346,828	AX-90034437
TNNT2	---	chr1:201,328,136-201,346,828	AX-90025722
TNNT2	rs397516451	chr1:201,328,136-201,346,828	AX-90028451
TNNT2	rs121964855	chr1:201,328,136-201,346,828	AX-86894480
TNNT2	rs144900708	chr1:201,328,136-201,346,828	AX-83092825
TNNT2	rs397516450	chr1:201,328,136-201,346,828	AX-90031203
TNNT2	---	chr1:201,328,136-201,346,828	AX-90033366
TNNT2	rs3729845	chr1:201,328,136-201,346,828	AX-11476405
TNNT2	rs3841823	chr1:201,328,136-201,346,828	AX-88811926
TNNT2	rs7544061	chr1:201,328,136-201,346,828	AX-156270646
TNNT2	rs7544061	chr1:201,328,136-201,346,828	AX-156289412
TNNT2	---	chr1:201,328,136-201,346,828	AX-90054608
TNNT2	rs45449197	chr1:201,328,136-201,346,828	AX-30416807
TNNT2	rs45585240	chr1:201,328,136-201,346,828	AX-30416809
TNNT2	rs10800775	chr1:201,328,136-201,346,828	AX-39250017
TNNT2	rs1892028	chr1:201,328,136-201,346,828	AX-39250019
TNNT2	---	chr1:201,328,136-201,346,828	AX-90060984
TNNT2	rs3729843	chr1:201,328,136-201,346,828	AX-39250025
TNNT2	rs3729842	chr1:201,328,136-201,346,828	AX-39250027
TNNT2	---	chr1:201,328,136-201,346,828	AX-90061720
TNNT2	rs3753999	chr1:201,328,136-201,346,828	AX-39250031
TNNT2	rs3753997	chr1:201,328,136-201,346,828	AX-39250033
TNNT2	rs79556064	chr1:201,328,136-201,346,828	AX-37431007
TNNT2	rs16848462	chr1:201,328,136-201,346,828	AX-39250039
TNNT2	rs10800776	chr1:201,328,136-201,346,828	AX-156265646
TNNT2	rs10920183	chr1:201,328,136-201,346,828	AX-39250045

TNNT2	rs10920184	chr1:201,328,136-201,346,828	AX-107849877
TNNT2	rs10920184	chr1:201,328,136-201,346,828	AX-156265682
TNNT2	---	chr1:201,328,136-201,346,828	AX-30416823
TNNT2	rs45584633	chr1:201,328,136-201,346,828	AX-156265643
TNNT2	rs45584633	chr1:201,328,136-201,346,828	AX-156265678
TNNT2	rs541173867	chr1:201,328,136-201,346,828	AX-151432957
TNNT2	rs541173867	chr1:201,328,136-201,346,828	AX-156298619
TNNT2	rs1419276	chr1:201,328,136-201,346,828	AX-39250049
TNNT2	rs12567686	chr1:201,328,136-201,346,828	AX-156266185
TNNT2	rs12567686	chr1:201,328,136-201,346,828	AX-156286772
TNNT2	rs149812965	chr1:201,328,136-201,346,828	AX-151358932
TNNT2	rs149812965	chr1:201,328,136-201,346,828	AX-156284259
TNNT2	rs4915240	chr1:201,328,136-201,346,828	AX-39250053
TNNT2	rs6427901	chr1:201,328,136-201,346,828	AX-39250055
TNNT2	rs115156877	chr1:201,328,136-201,346,828	AX-30416841
TNNT2	rs45533739	chr1:201,328,136-201,346,828	AX-83447214
TNNT2	rs868407	chr1:201,328,136-201,346,828	AX-123130871
TNNT2	rs868407	chr1:201,328,136-201,346,828	AX-123130872
TNNT2	rs6663175	chr1:201,328,136-201,346,828	AX-16791526
TNNT2	rs10920186	chr1:201,328,136-201,346,828	AX-30416851
TNNT2	rs12567382	chr1:201,328,136-201,346,828	AX-30416853
TNNT2	rs12568262	chr1:201,328,136-201,346,828	AX-30416857
TNNT2	rs1539693	chr1:201,328,136-201,346,828	AX-123024762
TNNT2	rs1539693	chr1:201,328,136-201,346,828	AX-123024763
TNNT2	rs3020557	chr1:201,328,136-201,346,828	AX-92686101
TNNT2	rs3020556	chr1:201,328,136-201,346,828	AX-156295481
TNNT2	rs56126027	chr1:201,328,136-201,346,828	AX-30416863
TNNT2	rs61820016	chr1:201,328,136-201,346,828	AX-30416865
TNNT2	rs56982832	chr1:201,328,136-201,346,828	AX-156286773
TNNT2	rs59982007	chr1:201,328,136-201,346,828	AX-30416869
TNNT2	rs2996494	chr1:201,328,136-201,346,828	AX-39250067
TNNT2	rs2996493	chr1:201,328,136-201,346,828	AX-156266187
TNNT2	rs2996493	chr1:201,328,136-201,346,828	AX-156286774
TNNT2	rs113236409	chr1:201,328,136-201,346,828	AX-156266188
TNNT2	rs113236409	chr1:201,328,136-201,346,828	AX-156286775
TNNT2	rs2996492	chr1:201,328,136-201,346,828	AX-156266189
TNNT2	rs2996492	chr1:201,328,136-201,346,828	AX-156286776
TNNT2	rs73076669	chr1:201,328,136-201,346,828	AX-30416877
TNNT2	rs12566896	chr1:201,328,136-201,346,828	AX-156266190
TNNT2	rs12566896	chr1:201,328,136-201,346,828	AX-156286777
TNNT2	rs60086234	chr1:201,328,136-201,346,828	AX-113652059
TNNT2	rs60086234	chr1:201,328,136-201,346,828	AX-123132893
TNNT2	rs73076673	chr1:201,328,136-201,346,828	AX-156266191
TNNT2	rs73076673	chr1:201,328,136-201,346,828	AX-156286778
TNNT2	rs74136606	chr1:201,328,136-201,346,828	AX-156266192
TNNT2	rs74136606	chr1:201,328,136-201,346,828	AX-156286779
TNNT2	rs75826050	chr1:201,328,136-201,346,828	AX-156266193
TNNT2	rs12568802	chr1:201,328,136-201,346,828	AX-39250069
TNNT2	rs12563097	chr1:201,328,136-201,346,828	AX-39250071
TNNT2	rs12563114	chr1:201,328,136-201,346,828	AX-39250075
TNNT2	rs12568177	chr1:201,328,136-201,346,828	AX-39250077
TNNT2	rs12564416	chr1:201,328,136-201,346,828	AX-156279923
TNNT2	rs12564416	chr1:201,328,136-201,346,828	AX-156295482
TNNT2	rs947486	chr1:201,328,136-201,346,828	AX-39250081
TNNT2	rs947485	chr1:201,328,136-201,346,828	AX-39250083

TNNT2	rs12564445	chr1:201,328,136-201,346,828	AX-39250085
TPM1	rs397516364	chr15:63,334,838-63,364,113	AX-90065672
TPM1	rs199476301	chr15:63,334,838-63,364,113	AX-90040932
TPM1	rs397516382	chr15:63,334,838-63,364,113	AX-90063927
TPM1	rs199476303	chr15:63,334,838-63,364,113	AX-90040933
TPM1	---	chr15:63,334,838-63,364,113	AX-90069788
TPM1	rs199476304	chr15:63,334,838-63,364,113	AX-90069820
TPM1	rs199476305	chr15:63,334,838-63,364,113	AX-90069821
TPM1	rs199476306	chr15:63,334,838-63,364,113	AX-90069822
TPM1	---	chr15:63,334,838-63,364,113	AX-90065914
TPM1	rs6494386	chr15:63,334,838-63,364,113	AX-31668665
TPM1	rs5813188	chr15:63,334,838-63,364,113	AX-121135762
TPM1	rs5813188	chr15:63,334,838-63,364,113	AX-156280293
TPM1	rs8042331	chr15:63,334,838-63,364,113	AX-156271457
TPM1	rs8026502	chr15:63,334,838-63,364,113	AX-31668671
TPM1	rs79854225	chr15:63,334,838-63,364,113	AX-12949668
TPM1	rs57645645	chr15:63,334,838-63,364,113	AX-31668673
TPM1	rs76273871	chr15:63,334,838-63,364,113	AX-31668675
TPM1	rs4075583	chr15:63,334,838-63,364,113	AX-12949669
TPM1	rs4075584	chr15:63,334,838-63,364,113	AX-156289933
TPM1	rs73431508	chr15:63,334,838-63,364,113	AX-156271459
TPM1	rs148517607	chr15:63,334,838-63,364,113	AX-88768005
TPM1	rs148517607	chr15:63,334,838-63,364,113	AX-88811050
TPM1	rs11856618	chr15:63,334,838-63,364,113	AX-31668679
TPM1	rs66793829	chr15:63,334,838-63,364,113	AX-12949671
TPM1	rs78880342	chr15:63,334,838-63,364,113	AX-31668681
TPM1	rs12441488	chr15:63,334,838-63,364,113	AX-31668685
TPM1	rs28609950	chr15:63,334,838-63,364,113	AX-31668687
TPM1	rs16946345	chr15:63,334,838-63,364,113	AX-39927149
TPM1	rs11071720	chr15:63,334,838-63,364,113	AX-12949672
TPM1	rs74685685	chr15:63,334,838-63,364,113	AX-112926699
TPM1	rs74685685	chr15:63,334,838-63,364,113	AX-113483340
TPM1	rs76113767	chr15:63,334,838-63,364,113	AX-12949674
TPM1	rs3803499	chr15:63,334,838-63,364,113	AX-107829457
TPM1	rs3803499	chr15:63,334,838-63,364,113	AX-113474644
TPM1	rs3840027	chr15:63,334,838-63,364,113	AX-88773414
TPM1	rs199942026	chr15:63,334,838-63,364,113	AX-113026869
TPM1	rs570374605	chr15:63,334,838-63,364,113	AX-90056107
TPM1	rs17752921	chr15:63,334,838-63,364,113	AX-11338484
TPM1	rs3840028	chr15:63,334,838-63,364,113	AX-107785641
TPM1	rs35363782	chr15:63,334,838-63,364,113	AX-107808988
TPM1	rs55719615	chr15:63,334,838-63,364,113	AX-112992132
TPM1	rs200383195	chr15:63,334,838-63,364,113	AX-151211951
TPM1	rs200383195	chr15:63,334,838-63,364,113	AX-156298026
TPM1	rs1135403	chr15:63,334,838-63,364,113	AX-39927153
TPM1	rs6494387	chr15:63,334,838-63,364,113	AX-39927157
TPM1	rs7170462	chr15:63,334,838-63,364,113	AX-39927159
TPM1	rs5813192	chr15:63,334,838-63,364,113	AX-156284498
TPM1	rs1574311	chr15:63,334,838-63,364,113	AX-12949679
TPM1	rs4468558	chr15:63,334,838-63,364,113	AX-12949680
TPM1	rs4238370	chr15:63,334,838-63,364,113	AX-12949683
TPM1	rs4238371	chr15:63,334,838-63,364,113	AX-12949684
TPM1	rs16946372	chr15:63,334,838-63,364,113	AX-39927163
TPM1	rs16953467	chr15:63,334,838-63,364,113	AX-39927165
TPM1	rs16953475	chr15:63,334,838-63,364,113	AX-12949685

TPM1	rs72743223	chr15:63,334,838-63,364,113	AX-31668713
TPM1	rs7166724	chr15:63,334,838-63,364,113	AX-39927167
TPM1	rs7166624	chr15:63,334,838-63,364,113	AX-114236690
TPM1	rs28645615	chr15:63,334,838-63,364,113	AX-148662507
TPM1	rs28645615	chr15:63,334,838-63,364,113	AX-92589086
TPM1	---	chr15:63,334,838-63,364,113	AX-119987801
TPM1	rs4775613	chr15:63,334,838-63,364,113	AX-11525364
TPM1	rs28485524	chr15:63,334,838-63,364,113	AX-12949689
TPM1	rs199476310	chr15:63,334,838-63,364,113	AX-90040938
TPM1	rs104894504	chr15:63,334,838-63,364,113	AX-90069789
TPM1	---	chr15:63,334,838-63,364,113	AX-90036600
TPM1	rs7173072	chr15:63,334,838-63,364,113	AX-39927171
TPM1	rs17753220	chr15:63,334,838-63,364,113	AX-11338500
TPM1	rs67399516	chr15:63,334,838-63,364,113	AX-107708515
TPM1	rs67399516	chr15:63,334,838-63,364,113	AX-156285153
TPM1	rs4450350	chr15:63,334,838-63,364,113	AX-11506148
TPM1	rs11071721	chr15:63,334,838-63,364,113	AX-12949691
TPM1	rs73438597	chr15:63,334,838-63,364,113	AX-12949692
TPM1	rs11071722	chr15:63,334,838-63,364,113	AX-12949693
TPM1	rs80349772	chr15:63,334,838-63,364,113	AX-12949695
TPM1	rs28531385	chr15:63,334,838-63,364,113	AX-31668721
TPM1	rs4238372	chr15:63,334,838-63,364,113	AX-11497611
TPM1	rs73440404	chr15:63,334,838-63,364,113	AX-12949698
TPM1	rs4775614	chr15:63,334,838-63,364,113	AX-12949699
TPM1	rs1071646	chr15:63,334,838-63,364,113	AX-12949700
TPM1	rs199476311	chr15:63,334,838-63,364,113	AX-90040939
TPM1	rs3803501	chr15:63,334,838-63,364,113	AX-12562063
TPM1	rs3803502	chr15:63,334,838-63,364,113	AX-156271460
TPM1	rs3803502	chr15:63,334,838-63,364,113	AX-156289935
TPM1	rs7162932	chr15:63,334,838-63,364,113	AX-39927177
TPM1	rs199476312	chr15:63,334,838-63,364,113	AX-86562641
TPM1	rs104894503	chr15:63,334,838-63,364,113	AX-90040900
TPM1	---	chr15:63,334,838-63,364,113	AX-90032725
TPM1	---	chr15:63,334,838-63,364,113	AX-90040899
TPM1	rs199476314	chr15:63,334,838-63,364,113	AX-90069826
TPM1	rs144700226	chr15:63,334,838-63,364,113	AX-83424373
TPM1	rs4774472	chr15:63,334,838-63,364,113	AX-39927181
TPM1	rs199476315	chr15:63,334,838-63,364,113	AX-86894434
TPM1	rs397516487	chr15:63,334,838-63,364,113	AX-90034112
TPM1	rs199476316	chr15:63,334,838-63,364,113	AX-90069827
TPM1	rs199476319	chr15:63,334,838-63,364,113	AX-90040942
TPM1	rs372214593	chr15:63,334,838-63,364,113	AX-94358682
TPM1	---	chr15:63,334,838-63,364,113	AX-11206528
TPM1	rs11071723	chr15:63,334,838-63,364,113	AX-12949706
TPM1	rs12148828	chr15:63,334,838-63,364,113	AX-96062610
TPM1	rs555534926	chr15:63,334,838-63,364,113	AX-90034686
TPM1	rs16946410	chr15:63,334,838-63,364,113	AX-39927185
TPM1	rs75449003	chr15:63,334,838-63,364,113	AX-12949709
TPM1	rs8042050	chr15:63,334,838-63,364,113	AX-39927187
TPM1	rs28660606	chr15:63,334,838-63,364,113	AX-113729304
TPM1	rs28660606	chr15:63,334,838-63,364,113	AX-156271461
TPM1	rs397516392	chr15:63,334,838-63,364,113	AX-90060612
TPM1	rs28730803	chr15:63,334,838-63,364,113	AX-31668739
TPM1	rs73440420	chr15:63,334,838-63,364,113	AX-12949714
TPM1	rs16946416	chr15:63,334,838-63,364,113	AX-12949717

TPM1	rs11636549	chr15:63,334,838-63,364,113	AX-156271462
TPM1	rs11636549	chr15:63,334,838-63,364,113	AX-156289936
TPM1	rs28583444	chr15:63,334,838-63,364,113	AX-156289937
TPM1	rs374459540	chr15:63,334,838-63,364,113	AX-94375433
TPM1	rs28512289	chr15:63,334,838-63,364,113	AX-156271464
TPM1	rs28512289	chr15:63,334,838-63,364,113	AX-156289938
TPM1	rs28735896	chr15:63,334,838-63,364,113	AX-156271465
TPM1	rs28735896	chr15:63,334,838-63,364,113	AX-156289939
TPM1	rs17826341	chr15:63,334,838-63,364,113	AX-156271466
TPM1	rs146808377	chr15:63,334,838-63,364,113	AX-156284603
TPM1	rs4619348	chr15:63,334,838-63,364,113	AX-12949722
TPM1	rs77372534	chr15:63,334,838-63,364,113	AX-156271467
TPM1	rs77372534	chr15:63,334,838-63,364,113	AX-156289941
TPM1	rs73440424	chr15:63,334,838-63,364,113	AX-12949725
TPM1	rs2729826	chr15:63,334,838-63,364,113	AX-12949726
TPM1	rs2729827	chr15:63,334,838-63,364,113	AX-39927197
TPM1	rs8040219	chr15:63,334,838-63,364,113	AX-12949727
TPM1	rs1972041	chr15:63,334,838-63,364,113	AX-11356775
TPM1	rs34359816	chr15:63,334,838-63,364,113	AX-12949728
TPM1	rs112894511	chr15:63,334,838-63,364,113	AX-148128674
TPM1	rs112894511	chr15:63,334,838-63,364,113	AX-88317995
TPM1	rs2173982	chr15:63,334,838-63,364,113	AX-123079078
TPM1	rs2173982	chr15:63,334,838-63,364,113	AX-123079079
TPM1	rs2729828	chr15:63,334,838-63,364,113	AX-156271468
TPM1	rs2729828	chr15:63,334,838-63,364,113	AX-92549739
TPM1	rs73440433	chr15:63,334,838-63,364,113	AX-12949733
TPM1	---	chr15:63,334,838-63,364,113	AX-123003239
TPM1	---	chr15:63,334,838-63,364,113	AX-123003240
TPM1	rs68083918	chr15:63,334,838-63,364,113	AX-105051176
TPM1	rs68083918	chr15:63,334,838-63,364,113	AX-113352535
TPM1	rs9920576	chr15:63,334,838-63,364,113	AX-39927201
TPM1	rs144045691	chr15:63,334,838-63,364,113	AX-83039629
TPM1	rs1050255	chr15:63,334,838-63,364,113	AX-12391663
TPM1	rs8519	chr15:63,334,838-63,364,113	AX-39927207
TPM1	rs707602	chr15:63,334,838-63,364,113	AX-12619776
TPM1	rs6738	chr15:63,334,838-63,364,113	AX-12607735
TPM1	rs7178040	chr15:63,334,838-63,364,113	AX-39927209
TTN	rs281864929	chr2:179,390,717-179,672,150	AX-90029929
TTN	rs281864930	chr2:179,390,717-179,672,150	AX-90047286
TTN	rs267607156	chr2:179,390,717-179,672,150	AX-90027182
TTN	---	chr2:179,390,717-179,672,150	AX-90033436
TTN	rs281864931	chr2:179,390,717-179,672,150	AX-90034831
TTN	rs16866373	chr2:179,390,717-179,672,150	AX-13767752
TTN	rs72629793	chr2:179,390,717-179,672,150	AX-83011396
TTN	rs16866378	chr2:179,390,717-179,672,150	AX-11285024
TTN	rs55842557	chr2:179,390,717-179,672,150	AX-83050073
TTN	---	chr2:179,390,717-179,672,150	AX-90073909
TTN	rs56324602	chr2:179,390,717-179,672,150	AX-83521932
TTN	rs56207956	chr2:179,390,717-179,672,150	AX-13767757
TTN	rs66961115	chr2:179,390,717-179,672,150	AX-83165791
TTN	rs16866380	chr2:179,390,717-179,672,150	AX-13767758
TTN	rs56324595	chr2:179,390,717-179,672,150	AX-33413111
TTN	rs3829749	chr2:179,390,717-179,672,150	AX-11483960
TTN	---	chr2:179,390,717-179,672,150	AX-90075580
TTN	rs3813250	chr2:179,390,717-179,672,150	AX-11482707

TTN	rs56308529	chr2:179,390,717-179,672,150	AX-33413113
TTN	rs568460311	chr2:179,390,717-179,672,150	AX-86687951
TTN	rs3829748	chr2:179,390,717-179,672,150	AX-11483959
TTN	rs201218828	chr2:179,390,717-179,672,150	AX-83320023
TTN	rs68080670	chr2:179,390,717-179,672,150	AX-83522724
TTN	---	chr2:179,390,717-179,672,150	AX-33413117
TTN	rs199642423	chr2:179,390,717-179,672,150	AX-82938542
TTN	rs3829747	chr2:179,390,717-179,672,150	AX-11483958
TTN	rs192001910	chr2:179,390,717-179,672,150	AX-83305269
TTN	rs148525155	chr2:179,390,717-179,672,150	AX-83119621
TTN	rs3731752	chr2:179,390,717-179,672,150	AX-13767761
TTN	rs56173891	chr2:179,390,717-179,672,150	AX-83328404
TTN	rs56347248	chr2:179,390,717-179,672,150	AX-83087555
TTN	rs2857265	chr2:179,390,717-179,672,150	AX-11419766
TTN	rs140319117	chr2:179,390,717-179,672,150	AX-83020626
TTN	rs144963736	chr2:179,390,717-179,672,150	AX-83068896
TTN	rs55669553	chr2:179,390,717-179,672,150	AX-83056688
TTN	rs55886356	chr2:179,390,717-179,672,150	AX-83077916
TTN	rs34924609	chr2:179,390,717-179,672,150	AX-11455314
TTN	rs56273463	chr2:179,390,717-179,672,150	AX-83425998
TTN	rs2278196	chr2:179,390,717-179,672,150	AX-40776699
TTN	rs200166942	chr2:179,390,717-179,672,150	AX-82892455
TTN	rs4894027	chr2:179,390,717-179,672,150	AX-33413131
TTN	rs55675869	chr2:179,390,717-179,672,150	AX-13767764
TTN	rs55742743	chr2:179,390,717-179,672,150	AX-33413133
TTN	rs72648278	chr2:179,390,717-179,672,150	AX-83226537
TTN	rs2303539	chr2:179,390,717-179,672,150	AX-33413139
TTN	---	chr2:179,390,717-179,672,150	AX-11534157
TTN	rs55977045	chr2:179,390,717-179,672,150	AX-33413141
TTN	rs149001703	chr2:179,390,717-179,672,150	AX-83140669
TTN	rs72648273	chr2:179,390,717-179,672,150	AX-83261632
TTN	rs72648272	chr2:179,390,717-179,672,150	AX-83380431
TTN	rs72648270	chr2:179,390,717-179,672,150	AX-33413143
TTN	rs2288325	chr2:179,390,717-179,672,150	AX-40776701
TTN	---	chr2:179,390,717-179,672,150	AX-90061211
TTN	---	chr2:179,390,717-179,672,150	AX-90064999
TTN	rs3820978	chr2:179,390,717-179,672,150	AX-13767768
TTN	rs3731751	chr2:179,390,717-179,672,150	AX-37942955
TTN	rs3731750	chr2:179,390,717-179,672,150	AX-13767769
TTN	---	chr2:179,390,717-179,672,150	AX-83103746
TTN	rs3731749	chr2:179,390,717-179,672,150	AX-11476504
TTN	rs62621236	chr2:179,390,717-179,672,150	AX-13767773
TTN	rs199781261	chr2:179,390,717-179,672,150	AX-83418558
TTN	rs72648263	chr2:179,390,717-179,672,150	AX-83362572
TTN	rs62621206	chr2:179,390,717-179,672,150	AX-82980308
TTN	rs191484894	chr2:179,390,717-179,672,150	AX-83282341
TTN	---	chr2:179,390,717-179,672,150	AX-90057376
TTN	rs72648257	chr2:179,390,717-179,672,150	AX-13767777
TTN	rs72648256	chr2:179,390,717-179,672,150	AX-83507166
TTN	rs2288327	chr2:179,390,717-179,672,150	AX-33413161
TTN	rs181104321	chr2:179,390,717-179,672,150	AX-83292044
TTN	rs67665715	chr2:179,390,717-179,672,150	AX-13767779
TTN	rs72648251	chr2:179,390,717-179,672,150	AX-83347552
TTN	rs35445420	chr2:179,390,717-179,672,150	AX-11464092
TTN	rs3731748	chr2:179,390,717-179,672,150	AX-11476503

TTN	rs180975448	chr2:179,390,717-179,672,150	AX-82938431
TTN	rs11694623	chr2:179,390,717-179,672,150	AX-11168496
TTN	rs16866391	chr2:179,390,717-179,672,150	AX-40776707
TTN	rs182422055	chr2:179,390,717-179,672,150	AX-83271583
TTN	rs747122	chr2:179,390,717-179,672,150	AX-13767780
TTN	rs890578	chr2:179,390,717-179,672,150	AX-13767781
TTN	rs148617456	chr2:179,390,717-179,672,150	AX-83554854
TTN	rs182549226	chr2:179,390,717-179,672,150	AX-83502525
TTN	rs72648244	chr2:179,390,717-179,672,150	AX-83409909
TTN	rs11897366	chr2:179,390,717-179,672,150	AX-11180961
TTN	rs11887722	chr2:179,390,717-179,672,150	AX-40776709
TTN	rs150430592	chr2:179,390,717-179,672,150	AX-83144145
TTN	---	chr2:179,390,717-179,672,150	AX-90042684
TTN	---	chr2:179,390,717-179,672,150	AX-90047294
TTN	rs72648238	chr2:179,390,717-179,672,150	AX-82885192
TTN	---	chr2:179,390,717-179,672,150	AX-94372114
TTN	rs200513274	chr2:179,390,717-179,672,150	AX-83001000
TTN	rs200899806	chr2:179,390,717-179,672,150	AX-83032576
TTN	rs146181116	chr2:179,390,717-179,672,150	AX-83334645
TTN	rs200305114	chr2:179,390,717-179,672,150	AX-96558268
TTN	rs3731747	chr2:179,390,717-179,672,150	AX-96548223
TTN	rs147015460	chr2:179,390,717-179,672,150	AX-92865283
TTN	rs147015460	chr2:179,390,717-179,672,150	AX-96549298
TTN	rs35504893	chr2:179,390,717-179,672,150	AX-33413171
TTN	rs16866397	chr2:179,390,717-179,672,150	AX-40776715
TTN	rs9808036	chr2:179,390,717-179,672,150	AX-11703182
TTN	rs9808377	chr2:179,390,717-179,672,150	AX-11703204
TTN	rs183013408	chr2:179,390,717-179,672,150	AX-83240499
TTN	rs72648229	chr2:179,390,717-179,672,150	AX-13767789
TTN	rs72648227	chr2:179,390,717-179,672,150	AX-82907153
TTN	rs12621078	chr2:179,390,717-179,672,150	AX-11218715
TTN	rs73036370	chr2:179,390,717-179,672,150	AX-13767790
TTN	rs201611599	chr2:179,390,717-179,672,150	AX-156285341
TTN	rs201611599	chr2:179,390,717-179,672,150	AX-96642598
TTN	---	chr2:179,390,717-179,672,150	AX-156286187
TTN	---	chr2:179,390,717-179,672,150	AX-96635479
TTN	rs28653157	chr2:179,390,717-179,672,150	AX-13767792
TTN	rs56310931	chr2:179,390,717-179,672,150	AX-13767793
TTN	rs190721759	chr2:179,390,717-179,672,150	AX-83216922
TTN	rs72648221	chr2:179,390,717-179,672,150	AX-83561385
TTN	rs73036373	chr2:179,390,717-179,672,150	AX-13767796
TTN	rs201348580	chr2:179,390,717-179,672,150	AX-83493298
TTN	rs2366751	chr2:179,390,717-179,672,150	AX-11387461
TTN	rs3829746	chr2:179,390,717-179,672,150	AX-13767798
TTN	rs185002960	chr2:179,390,717-179,672,150	AX-83070822
TTN	rs201422612	chr2:179,390,717-179,672,150	AX-83566606
TTN	rs11896637	chr2:179,390,717-179,672,150	AX-40776719
TTN	rs55634791	chr2:179,390,717-179,672,150	AX-83410833
TTN	rs201158906	chr2:179,390,717-179,672,150	AX-83020696
TTN	rs201958805	chr2:179,390,717-179,672,150	AX-83390752
TTN	rs56214710	chr2:179,390,717-179,672,150	AX-13767804
TTN	rs186273940	chr2:179,390,717-179,672,150	AX-82897371
TTN	rs3813247	chr2:179,390,717-179,672,150	AX-11482706
TTN	rs79926414	chr2:179,390,717-179,672,150	AX-13767805
TTN	---	chr2:179,390,717-179,672,150	AX-11476502

TTN	---	chr2:179,390,717-179,672,150	AX-33413183
TTN	rs3731745	chr2:179,390,717-179,672,150	AX-40776723
TTN	rs369019463	chr2:179,390,717-179,672,150	AX-91985246
TTN	rs72648206	chr2:179,390,717-179,672,150	AX-83129627
TTN	---	chr2:179,390,717-179,672,150	AX-13767809
TTN	rs12463674	chr2:179,390,717-179,672,150	AX-40776727
TTN	rs56018860	chr2:179,390,717-179,672,150	AX-13767810
TTN	rs3813246	chr2:179,390,717-179,672,150	AX-11482705
TTN	rs3813245	chr2:179,390,717-179,672,150	AX-13767811
TTN	rs3813243	chr2:179,390,717-179,672,150	AX-40776731
TTN	---	chr2:179,390,717-179,672,150	AX-86693270
TTN	rs142453163	chr2:179,390,717-179,672,150	AX-83241097
TTN	rs6732060	chr2:179,390,717-179,672,150	AX-11581492
TTN	rs201512527	chr2:179,390,717-179,672,150	AX-83271403
TTN	rs744426	chr2:179,390,717-179,672,150	AX-13767813
TTN	rs72646898	chr2:179,390,717-179,672,150	AX-83571570
TTN	rs55762754	chr2:179,390,717-179,672,150	AX-83320010
TTN	rs56201325	chr2:179,390,717-179,672,150	AX-83043549
TTN	rs56399205	chr2:179,390,717-179,672,150	AX-82930880
TTN	rs10164753	chr2:179,390,717-179,672,150	AX-11097002
TTN	rs55837610	chr2:179,390,717-179,672,150	AX-83064948
TTN	rs12464787	chr2:179,390,717-179,672,150	AX-11208034
TTN	---	chr2:179,390,717-179,672,150	AX-90049730
TTN	rs201043950	chr2:179,390,717-179,672,150	AX-83172795
TTN	rs72646885	chr2:179,390,717-179,672,150	AX-83414902
TTN	rs67041405	chr2:179,390,717-179,672,150	AX-33413207
TTN	rs72646881	chr2:179,390,717-179,672,150	AX-33413209
TTN	rs55980498	chr2:179,390,717-179,672,150	AX-82917783
TTN	rs199506676	chr2:179,390,717-179,672,150	AX-83576915
TTN	---	chr2:179,390,717-179,672,150	AX-83138570
TTN	---	chr2:179,390,717-179,672,150	AX-86598984
TTN	rs3769864	chr2:179,390,717-179,672,150	AX-113406023
TTN	rs10497517	chr2:179,390,717-179,672,150	AX-11111534
TTN	rs2288571	chr2:179,390,717-179,672,150	AX-40776741
TTN	rs2288570	chr2:179,390,717-179,672,150	AX-40776743
TTN	rs72646873	chr2:179,390,717-179,672,150	AX-83528863
TTN	rs2303838	chr2:179,390,717-179,672,150	AX-13767821
TTN	rs72646869	chr2:179,390,717-179,672,150	AX-50339928
TTN	rs183276016	chr2:179,390,717-179,672,150	AX-83507981
TTN	rs4894029	chr2:179,390,717-179,672,150	AX-11534158
TTN	rs72646866	chr2:179,390,717-179,672,150	AX-90041790
TTN	---	chr2:179,390,717-179,672,150	AX-86712695
TTN	rs16866400	chr2:179,390,717-179,672,150	AX-13767828
TTN	rs13021201	chr2:179,390,717-179,672,150	AX-11236327
TTN	rs72646861	chr2:179,390,717-179,672,150	AX-33413217
TTN	rs150661999	chr2:179,390,717-179,672,150	AX-83006042
TTN	rs181717727	chr2:179,390,717-179,672,150	AX-82949451
TTN	rs2042996	chr2:179,390,717-179,672,150	AX-13767831
TTN	rs1007025	chr2:179,390,717-179,672,150	AX-13767832
TTN	rs72646857	chr2:179,390,717-179,672,150	AX-13767833
TTN	rs72646855	chr2:179,390,717-179,672,150	AX-83425810
TTN	rs72646850	chr2:179,390,717-179,672,150	AX-33413225
TTN	rs200689750	chr2:179,390,717-179,672,150	AX-83250208
TTN	---	chr2:179,390,717-179,672,150	AX-86663161
TTN	rs1560221	chr2:179,390,717-179,672,150	AX-40776747

TTN	rs199895260	chr2:179,390,717-179,672,150	AX-82901253
TTN	rs2163009	chr2:179,390,717-179,672,150	AX-11371623
TTN	rs141973925	chr2:179,390,717-179,672,150	AX-83251414
TTN	rs72646845	chr2:179,390,717-179,672,150	AX-83133177
TTN	rs200212521	chr2:179,390,717-179,672,150	AX-83253796
TTN	rs201487340	chr2:179,390,717-179,672,150	AX-83205832
TTN	rs16866406	chr2:179,390,717-179,672,150	AX-13767839
TTN	rs72646841	chr2:179,390,717-179,672,150	AX-33413231
TTN	rs72646840	chr2:179,390,717-179,672,150	AX-83132796
TTN	rs72646838	chr2:179,390,717-179,672,150	AX-86713488
TTN	rs2288569	chr2:179,390,717-179,672,150	AX-83136669
TTN	rs62178962	chr2:179,390,717-179,672,150	AX-96640833
TTN	rs4894030	chr2:179,390,717-179,672,150	AX-13767841
TTN	rs28378468	chr2:179,390,717-179,672,150	AX-33413241
TTN	rs6712785	chr2:179,390,717-179,672,150	AX-148108649
TTN	rs6712785	chr2:179,390,717-179,672,150	AX-156273125
TTN	rs188185141	chr2:179,390,717-179,672,150	AX-83528634
TTN	rs35833641	chr2:179,390,717-179,672,150	AX-13767846
TTN	rs200001206	chr2:179,390,717-179,672,150	AX-83027647
TTN	rs377000174	chr2:179,390,717-179,672,150	AX-90061944
TTN	rs1001238	chr2:179,390,717-179,672,150	AX-11087300
TTN	rs13009158	chr2:179,390,717-179,672,150	AX-13767849
TTN	rs72646823	chr2:179,390,717-179,672,150	AX-83063011
TTN	rs76226740	chr2:179,390,717-179,672,150	AX-33413259
TTN	rs201412693	chr2:179,390,717-179,672,150	AX-83416855
TTN	rs72646817	chr2:179,390,717-179,672,150	AX-156286028
TTN	rs10497518	chr2:179,390,717-179,672,150	AX-11111535
TTN	rs2303834	chr2:179,390,717-179,672,150	AX-12525349
TTN	rs2303833	chr2:179,390,717-179,672,150	AX-33413263
TTN	rs80098281	chr2:179,390,717-179,672,150	AX-13767857
TTN	rs72646809	chr2:179,390,717-179,672,150	AX-13767858
TTN	rs2303832	chr2:179,390,717-179,672,150	AX-40776761
TTN	rs185913848	chr2:179,390,717-179,672,150	AX-83557702
TTN	---	chr2:179,390,717-179,672,150	AX-83095539
TTN	rs201213901	chr2:179,390,717-179,672,150	AX-83527583
TTN	rs200650668	chr2:179,390,717-179,672,150	AX-83086734
TTN	rs16866412	chr2:179,390,717-179,672,150	AX-11285029
TTN	rs191539637	chr2:179,390,717-179,672,150	AX-83594719
TTN	rs36043230	chr2:179,390,717-179,672,150	AX-51297476
TTN	rs202094100	chr2:179,390,717-179,672,150	AX-83133903
TTN	rs72677243	chr2:179,390,717-179,672,150	AX-83259147
TTN	rs16866416	chr2:179,390,717-179,672,150	AX-156265642
TTN	rs16866416	chr2:179,390,717-179,672,150	AX-156265677
TTN	rs202141158	chr2:179,390,717-179,672,150	AX-83028698
TTN	rs72677237	chr2:179,390,717-179,672,150	AX-83469909
TTN	rs3835071	chr2:179,390,717-179,672,150	AX-13767873
TTN	rs3835071	chr2:179,390,717-179,672,150	AX-96638658
TTN	rs72677235	chr2:179,390,717-179,672,150	AX-91990524
TTN	rs72677235	chr2:179,390,717-179,672,150	AX-92015651
TTN	rs146181477	chr2:179,390,717-179,672,150	AX-83427506
TTN	rs72677233	chr2:179,390,717-179,672,150	AX-82913695
TTN	rs72677232	chr2:179,390,717-179,672,150	AX-83221336
TTN	rs72677231	chr2:179,390,717-179,672,150	AX-82920594
TTN	rs201717871	chr2:179,390,717-179,672,150	AX-83418675
TTN	---	chr2:179,390,717-179,672,150	AX-90065945

TTN	rs72677225	chr2:179,390,717-179,672,150	AX-33413283
TTN	rs17354992	chr2:179,390,717-179,672,150	AX-11320928
TTN	rs12693162	chr2:179,390,717-179,672,150	AX-13767883
TTN	rs1017323	chr2:179,390,717-179,672,150	AX-11097525
TTN	rs12614435	chr2:179,390,717-179,672,150	AX-40776771
TTN	rs12988307	chr2:179,390,717-179,672,150	AX-92737707
TTN	rs12988307	chr2:179,390,717-179,672,150	AX-96640255
TTN	rs36113194	chr2:179,390,717-179,672,150	AX-156291063
TTN	rs16866418	chr2:179,390,717-179,672,150	AX-96558798
TTN	rs16866418	chr2:179,390,717-179,672,150	AX-96642367
TTN	rs16866420	chr2:179,390,717-179,672,150	AX-84615666
TTN	rs16866420	chr2:179,390,717-179,672,150	AX-84627283
TTN	rs192766485	chr2:179,390,717-179,672,150	AX-83241875
TTN	rs56034831	chr2:179,390,717-179,672,150	AX-33413305
TTN	rs6723526	chr2:179,390,717-179,672,150	AX-11580845
TTN	---	chr2:179,390,717-179,672,150	AX-12475297
TTN	rs7590037	chr2:179,390,717-179,672,150	AX-40776783
TTN	rs2288563	chr2:179,390,717-179,672,150	AX-11382040
TTN	---	chr2:179,390,717-179,672,150	AX-86699306
TTN	rs201257644	chr2:179,390,717-179,672,150	AX-83397539
TTN	rs201944202	chr2:179,390,717-179,672,150	AX-83508576
TTN	rs34659500	chr2:179,390,717-179,672,150	AX-12553290
TTN	rs185968622	chr2:179,390,717-179,672,150	AX-92572252
TTN	rs1469473	chr2:179,390,717-179,672,150	AX-40776797
TTN	rs2562845	chr2:179,390,717-179,672,150	AX-13767905
TTN	rs72650066	chr2:179,390,717-179,672,150	AX-33413335
TTN	rs148140756	chr2:179,390,717-179,672,150	AX-83374461
TTN	rs1429094	chr2:179,390,717-179,672,150	AX-33413339
TTN	rs72650064	chr2:179,390,717-179,672,150	AX-83364865
TTN	rs116792417	chr2:179,390,717-179,672,150	AX-91983601
TTN	rs116792417	chr2:179,390,717-179,672,150	AX-92008728
TTN	rs139167585	chr2:179,390,717-179,672,150	AX-96553384
TTN	rs139167585	chr2:179,390,717-179,672,150	AX-96636953
TTN	rs2163008	chr2:179,390,717-179,672,150	AX-96715761
TTN	rs2163008	chr2:179,390,717-179,672,150	AX-96725973
TTN	rs2562848	chr2:179,390,717-179,672,150	AX-11398636
TTN	rs10221760	chr2:179,390,717-179,672,150	AX-40776813
TTN	rs12998857	chr2:179,390,717-179,672,150	AX-96557230
TTN	rs12998857	chr2:179,390,717-179,672,150	AX-96640799
TTN	rs202014478	chr2:179,390,717-179,672,150	AX-83027103
TTN	rs377319699	chr2:179,390,717-179,672,150	AX-86563304
TTN	---	chr2:179,390,717-179,672,150	AX-86592714
TTN	rs577363824	chr2:179,390,717-179,672,150	AX-86590361
TTN	rs140640738	chr2:179,390,717-179,672,150	AX-83012719
TTN	rs2472751	chr2:179,390,717-179,672,150	AX-13767930
TTN	rs71023451	chr2:179,390,717-179,672,150	AX-96549783
TTN	rs71023451	chr2:179,390,717-179,672,150	AX-96633352
TTN	rs62178972	chr2:179,390,717-179,672,150	AX-96552325
TTN	rs62178972	chr2:179,390,717-179,672,150	AX-96635894
TTN	rs2742351	chr2:179,390,717-179,672,150	AX-13767935
TTN	rs2742349	chr2:179,390,717-179,672,150	AX-13767937
TTN	rs186084940	chr2:179,390,717-179,672,150	AX-88748799
TTN	rs186084940	chr2:179,390,717-179,672,150	AX-88791844
TTN	rs35112591	chr2:179,390,717-179,672,150	AX-13767939
TTN	---	chr2:179,390,717-179,672,150	AX-86676185

TTN	---	chr2:179,390,717-179,672,150	AX-82954024
TTN	rs36051007	chr2:179,390,717-179,672,150	AX-11474292
TTN	---	chr2:179,390,717-179,672,150	AX-86662140
TTN	rs115744476	chr2:179,390,717-179,672,150	AX-37942967
TTN	rs72650034	chr2:179,390,717-179,672,150	AX-82913023
TTN	rs2303828	chr2:179,390,717-179,672,150	AX-156273127
TTN	rs2303828	chr2:179,390,717-179,672,150	AX-156291064
TTN	rs116676813	chr2:179,390,717-179,672,150	AX-13767950
TTN	rs72650031	chr2:179,390,717-179,672,150	AX-83151113
TTN	---	chr2:179,390,717-179,672,150	AX-90081259
TTN	rs2627038	chr2:179,390,717-179,672,150	AX-13767954
TTN	rs2244492	chr2:179,390,717-179,672,150	AX-11378152
TTN	rs145387989	chr2:179,390,717-179,672,150	AX-86619950
TTN	rs372371333	chr2:179,390,717-179,672,150	AX-86580609
TTN	rs2255167	chr2:179,390,717-179,672,150	AX-156273128
TTN	rs2255167	chr2:179,390,717-179,672,150	AX-96633997
TTN	rs2042995	chr2:179,390,717-179,672,150	AX-11362540
TTN	rs73038323	chr2:179,390,717-179,672,150	AX-13767959
TTN	rs73038324	chr2:179,390,717-179,672,150	AX-13767960
TTN	rs16866439	chr2:179,390,717-179,672,150	AX-96549018
TTN	rs16866439	chr2:179,390,717-179,672,150	AX-96632587
TTN	rs6755456	chr2:179,390,717-179,672,150	AX-96638492
TTN	rs3045696	chr2:179,390,717-179,672,150	AX-151129218
TTN	rs113059495	chr2:179,390,717-179,672,150	AX-156278753
TTN	rs112088511	chr2:179,390,717-179,672,150	AX-151360692
TTN	rs112088511	chr2:179,390,717-179,672,150	AX-156278754
TTN	rs73038328	chr2:179,390,717-179,672,150	AX-13767963
TTN	rs141526230	chr2:179,390,717-179,672,150	AX-119621894
TTN	rs141526230	chr2:179,390,717-179,672,150	AX-96552865
TTN	rs16866440	chr2:179,390,717-179,672,150	AX-33413395
TTN	rs2251987	chr2:179,390,717-179,672,150	AX-40776843
TTN	rs72650011	chr2:179,390,717-179,672,150	AX-82891029
TTN	rs2366753	chr2:179,390,717-179,672,150	AX-40776845
TTN	rs16866442	chr2:179,390,717-179,672,150	AX-13767966
TTN	rs2742345	chr2:179,390,717-179,672,150	AX-11408568
TTN	rs72953397	chr2:179,390,717-179,672,150	AX-13767968
TTN	rs72650006	chr2:179,390,717-179,672,150	AX-33413417
TTN	rs2742343	chr2:179,390,717-179,672,150	AX-13767970
TTN	---	chr2:179,390,717-179,672,150	AX-86606851
TTN	rs371163094	chr2:179,390,717-179,672,150	AX-94355365
TTN	---	chr2:179,390,717-179,672,150	AX-90055163
TTN	rs4893852	chr2:179,390,717-179,672,150	AX-11534139
TTN	rs10203085	chr2:179,390,717-179,672,150	AX-33413423
TTN	rs77643383	chr2:179,390,717-179,672,150	AX-33413425
TTN	rs76465131	chr2:179,390,717-179,672,150	AX-33413429
TTN	rs2742332	chr2:179,390,717-179,672,150	AX-13767980
TTN	rs72648998	chr2:179,390,717-179,672,150	AX-13767982
TTN	rs12997957	chr2:179,390,717-179,672,150	AX-13767984
TTN	rs2742331	chr2:179,390,717-179,672,150	AX-156273129
TTN	rs2742331	chr2:179,390,717-179,672,150	AX-156291065
TTN	rs72648996	chr2:179,390,717-179,672,150	AX-90070678
TTN	rs201229221	chr2:179,390,717-179,672,150	AX-92024826
TTN	rs72648994	chr2:179,390,717-179,672,150	AX-83048346
TTN	rs199557654	chr2:179,390,717-179,672,150	AX-83194254
TTN	rs72648992	chr2:179,390,717-179,672,150	AX-33413437

TTN	rs13398235	chr2:179,390,717-179,672,150	AX-11252542
TTN	rs2562839	chr2:179,390,717-179,672,150	AX-13767986
TTN	rs72648990	chr2:179,390,717-179,672,150	AX-83035926
TTN	rs72648989	chr2:179,390,717-179,672,150	AX-83370588
TTN	rs12693164	chr2:179,390,717-179,672,150	AX-11223524
TTN	rs2562838	chr2:179,390,717-179,672,150	AX-13767987
TTN	rs2562836	chr2:179,390,717-179,672,150	AX-40776867
TTN	rs72648987	chr2:179,390,717-179,672,150	AX-33413439
TTN	rs12622914	chr2:179,390,717-179,672,150	AX-96642962
TTN	rs10183237	chr2:179,390,717-179,672,150	AX-40776871
TTN	rs200355367	chr2:179,390,717-179,672,150	AX-83011791
TTN	rs10183361	chr2:179,390,717-179,672,150	AX-156265668
TTN	rs10183361	chr2:179,390,717-179,672,150	AX-156265703
TTN	rs2562834	chr2:179,390,717-179,672,150	AX-11398635
TTN	rs2562833	chr2:179,390,717-179,672,150	AX-156281479
TTN	rs2562833	chr2:179,390,717-179,672,150	AX-96641311
TTN	rs2562832	chr2:179,390,717-179,672,150	AX-11398634
TTN	rs149855485	chr2:179,390,717-179,672,150	AX-83106673
TTN	rs72648986	chr2:179,390,717-179,672,150	AX-13767992
TTN	rs62178977	chr2:179,390,717-179,672,150	AX-13767993
TTN	rs13390491	chr2:179,390,717-179,672,150	AX-11252170
TTN	rs2627043	chr2:179,390,717-179,672,150	AX-11402136
TTN	rs6750145	chr2:179,390,717-179,672,150	AX-40776875
TTN	rs72648984	chr2:179,390,717-179,672,150	AX-83250659
TTN	rs201571580	chr2:179,390,717-179,672,150	AX-83251898
TTN	rs200103997	chr2:179,390,717-179,672,150	AX-83328482
TTN	rs72648981	chr2:179,390,717-179,672,150	AX-83248400
TTN	rs72648978	chr2:179,390,717-179,672,150	AX-33413441
TTN	rs62178978	chr2:179,390,717-179,672,150	AX-116090842
TTN	rs62178978	chr2:179,390,717-179,672,150	AX-156265694
TTN	rs16866465	chr2:179,390,717-179,672,150	AX-11285037
TTN	rs373060681	chr2:179,390,717-179,672,150	AX-86595432
TTN	rs149523263	chr2:179,390,717-179,672,150	AX-83119994
TTN	rs72648972	chr2:179,390,717-179,672,150	AX-82968895
TTN	rs17452588	chr2:179,390,717-179,672,150	AX-11324806
TTN	rs2562830	chr2:179,390,717-179,672,150	AX-13767998
TTN	---	chr2:179,390,717-179,672,150	AX-33413445
TTN	rs72648969	chr2:179,390,717-179,672,150	AX-83360693
TTN	rs201065037	chr2:179,390,717-179,672,150	AX-83230235
TTN	---	chr2:179,390,717-179,672,150	AX-94375769
TTN	---	chr2:179,390,717-179,672,150	AX-13768000
TTN	rs16866473	chr2:179,390,717-179,672,150	AX-13768001
TTN	rs2742327	chr2:179,390,717-179,672,150	AX-13768002
TTN	rs189951108	chr2:179,390,717-179,672,150	AX-83077632
TTN	rs72648964	chr2:179,390,717-179,672,150	AX-83260221
TTN	rs72648962	chr2:179,390,717-179,672,150	AX-13768003
TTN	rs72648960	chr2:179,390,717-179,672,150	AX-13768004
TTN	rs17355446	chr2:179,390,717-179,672,150	AX-11320948
TTN	---	chr2:179,390,717-179,672,150	AX-83128474
TTN	rs200866883	chr2:179,390,717-179,672,150	AX-83140464
TTN	rs17355460	chr2:179,390,717-179,672,150	AX-11320950
TTN	rs149470241	chr2:179,390,717-179,672,150	AX-83112026
TTN	rs2129110	chr2:179,390,717-179,672,150	AX-13768009
TTN	rs146627500	chr2:179,390,717-179,672,150	AX-83469437
TTN	---	chr2:179,390,717-179,672,150	AX-90051967

TTN	rs2627042	chr2:179,390,717-179,672,150	AX-107765093
TTN	rs2627042	chr2:179,390,717-179,672,150	AX-147949060
TTN	rs11888217	chr2:179,390,717-179,672,150	AX-11180475
TTN	rs72648954	chr2:179,390,717-179,672,150	AX-13768014
TTN	rs72648953	chr2:179,390,717-179,672,150	AX-82973641
TTN	rs184412722	chr2:179,390,717-179,672,150	AX-82919902
TTN	rs72648949	chr2:179,390,717-179,672,150	AX-82901938
TTN	rs200359082	chr2:179,390,717-179,672,150	AX-83345383
TTN	rs72648947	chr2:179,390,717-179,672,150	AX-83026558
TTN	rs17076	chr2:179,390,717-179,672,150	AX-13768017
TTN	rs146983095	chr2:179,390,717-179,672,150	AX-83002824
TTN	rs72648943	chr2:179,390,717-179,672,150	AX-83531619
TTN	rs72648942	chr2:179,390,717-179,672,150	AX-83161670
TTN	---	chr2:179,390,717-179,672,150	AX-90043694
TTN	rs72648940	chr2:179,390,717-179,672,150	AX-83489194
TTN	rs190636272	chr2:179,390,717-179,672,150	AX-83063597
TTN	rs72648937	chr2:179,390,717-179,672,150	AX-33413467
TTN	rs72648930	chr2:179,390,717-179,672,150	AX-82895334
TTN	rs72648927	chr2:179,390,717-179,672,150	AX-33413479
TTN	rs72648925	chr2:179,390,717-179,672,150	AX-83392571
TTN	rs72648923	chr2:179,390,717-179,672,150	AX-82932952
TTN	rs2742347	chr2:179,390,717-179,672,150	AX-11408569
TTN	rs2627041	chr2:179,390,717-179,672,150	AX-33413485
TTN	rs2627040	chr2:179,390,717-179,672,150	AX-40776905
TTN	rs73038347	chr2:179,390,717-179,672,150	AX-156279695
TTN	rs73038347	chr2:179,390,717-179,672,150	AX-156295311
TTN	rs147879266	chr2:179,390,717-179,672,150	AX-86674087
TTN	---	chr2:179,390,717-179,672,150	AX-86737890
TTN	rs200204761	chr2:179,390,717-179,672,150	AX-83118190
TTN	rs55857742	chr2:179,390,717-179,672,150	AX-83406858
TTN	rs1883085	chr2:179,390,717-179,672,150	AX-11349830
TTN	rs2562829	chr2:179,390,717-179,672,150	AX-11398633
TTN	rs267607158	chr2:179,390,717-179,672,150	AX-90029644
TTN	---	chr2:179,390,717-179,672,150	AX-37942971
TTN	rs201437752	chr2:179,390,717-179,672,150	AX-83529645
TTN	rs34618570	chr2:179,390,717-179,672,150	AX-83262280
TTN	rs34070843	chr2:179,390,717-179,672,150	AX-11440660
TTN	rs33971253	chr2:179,390,717-179,672,150	AX-83266682
TTN	---	chr2:179,390,717-179,672,150	AX-86711124
TTN	rs2627037	chr2:179,390,717-179,672,150	AX-11402135
TTN	rs59479297	chr2:179,390,717-179,672,150	AX-13768031
TTN	rs11893580	chr2:179,390,717-179,672,150	AX-156273130
TTN	rs11884172	chr2:179,390,717-179,672,150	AX-40776915
TTN	rs72648915	chr2:179,390,717-179,672,150	AX-13768045
TTN	rs145183384	chr2:179,390,717-179,672,150	AX-83269285
TTN	rs201273719	chr2:179,390,717-179,672,150	AX-86679726
TTN	rs72648913	chr2:179,390,717-179,672,150	AX-83277609
TTN	---	chr2:179,390,717-179,672,150	AX-86707547
TTN	---	chr2:179,390,717-179,672,150	AX-94359020
TTN	---	chr2:179,390,717-179,672,150	AX-94359021
TTN	rs62179016	chr2:179,390,717-179,672,150	AX-83588239
TTN	rs72648911	chr2:179,390,717-179,672,150	AX-13768046
TTN	rs66677602	chr2:179,390,717-179,672,150	AX-13768047
TTN	rs142973956	chr2:179,390,717-179,672,150	AX-82921066
TTN	rs72648910	chr2:179,390,717-179,672,150	AX-13768049

TTN	---	chr2:179,390,717-179,672,150	AX-90042886
TTN	rs61233923	chr2:179,390,717-179,672,150	AX-91991396
TTN	rs61233923	chr2:179,390,717-179,672,150	AX-92016524
TTN	---	chr2:179,390,717-179,672,150	AX-86599834
TTN	rs145581345	chr2:179,390,717-179,672,150	AX-83468569
TTN	rs72648909	chr2:179,390,717-179,672,150	AX-83283355
TTN	rs72648907	chr2:179,390,717-179,672,150	AX-33413515
TTN	---	chr2:179,390,717-179,672,150	AX-90079145
TTN	rs150615457	chr2:179,390,717-179,672,150	AX-82998715
TTN	---	chr2:179,390,717-179,672,150	AX-86582212
TTN	rs139486133	chr2:179,390,717-179,672,150	AX-82885211
TTN	rs75785339	chr2:179,390,717-179,672,150	AX-13768050
TTN	rs149748934	chr2:179,390,717-179,672,150	AX-83400839
TTN	rs140909116	chr2:179,390,717-179,672,150	AX-83379313
TTN	rs142304137	chr2:179,390,717-179,672,150	AX-83121849
TTN	rs72647901	chr2:179,390,717-179,672,150	AX-83560405
TTN	rs72647897	chr2:179,390,717-179,672,150	AX-83584191
TTN	rs922984	chr2:179,390,717-179,672,150	AX-86332321
TTN	rs922984	chr2:179,390,717-179,672,150	AX-86334397
TTN	---	chr2:179,390,717-179,672,150	AX-13768057
TTN	---	chr2:179,390,717-179,672,150	AX-86573121
TTN	rs148115514	chr2:179,390,717-179,672,150	AX-83125806
TTN	rs201047740	chr2:179,390,717-179,672,150	AX-83372864
TTN	rs200284932	chr2:179,390,717-179,672,150	AX-83442275
TTN	rs3754953	chr2:179,390,717-179,672,150	AX-156279701
TTN	rs3754953	chr2:179,390,717-179,672,150	AX-156295317
TTN	rs1484116	chr2:179,390,717-179,672,150	AX-156273131
TTN	rs1484116	chr2:179,390,717-179,672,150	AX-156291067
TTN	rs145167466	chr2:179,390,717-179,672,150	AX-96558905
TTN	rs200064770	chr2:179,390,717-179,672,150	AX-96550642
TTN	rs1484118	chr2:179,390,717-179,672,150	AX-11267887
TTN	rs7585334	chr2:179,390,717-179,672,150	AX-11631625
TTN	---	chr2:179,390,717-179,672,150	AX-86573284
TTN	---	chr2:179,390,717-179,672,150	AX-86689202
TTN	rs72955213	chr2:179,390,717-179,672,150	AX-33413537
TTN	rs371596417	chr2:179,390,717-179,672,150	AX-86709576
TTN	rs2291310	chr2:179,390,717-179,672,150	AX-11382381
TTN	---	chr2:179,390,717-179,672,150	AX-94359022
TTN	rs7600001	chr2:179,390,717-179,672,150	AX-12633561
TTN	rs73038360	chr2:179,390,717-179,672,150	AX-13768078
TTN	rs7604033	chr2:179,390,717-179,672,150	AX-13768079
TTN	rs35770984	chr2:179,390,717-179,672,150	AX-13768080
TTN	rs7572955	chr2:179,390,717-179,672,150	AX-40776945
TTN	rs4894042	chr2:179,390,717-179,672,150	AX-156273132
TTN	rs4894042	chr2:179,390,717-179,672,150	AX-156291068
TTN	rs7559166	chr2:179,390,717-179,672,150	AX-11629735
TTN	rs35168948	chr2:179,390,717-179,672,150	AX-156284673
TTN	rs115985443	chr2:179,390,717-179,672,150	AX-37942979
TTN	rs34819099	chr2:179,390,717-179,672,150	AX-11453503
TTN	rs2291311	chr2:179,390,717-179,672,150	AX-11382382
TTN	rs2291312	chr2:179,390,717-179,672,150	AX-13768089
TTN	rs149755500	chr2:179,390,717-179,672,150	AX-82989493
TTN	rs3816782	chr2:179,390,717-179,672,150	AX-96551684
TTN	rs3816782	chr2:179,390,717-179,672,150	AX-96635253
TTN	rs4894044	chr2:179,390,717-179,672,150	AX-40776955

TTN	rs4893853	chr2:179,390,717-179,672,150	AX-11534140
TTN	rs72647894	chr2:179,390,717-179,672,150	AX-83451603
TTN	rs2291313	chr2:179,390,717-179,672,150	AX-12524600
TTN	rs4471922	chr2:179,390,717-179,672,150	AX-13768092
TTN	---	chr2:179,390,717-179,672,150	AX-90078725
TTN	rs72647884	chr2:179,390,717-179,672,150	AX-90070676
TTN	rs375549179	chr2:179,390,717-179,672,150	AX-92009462
TTN	rs2306636	chr2:179,390,717-179,672,150	AX-11384059
TTN	rs33917087	chr2:179,390,717-179,672,150	AX-82967329
TTN	rs556908341	chr2:179,390,717-179,672,150	AX-94359023
TTN	rs4893854	chr2:179,390,717-179,672,150	AX-13768095
TTN	rs4894047	chr2:179,390,717-179,672,150	AX-156273133
TTN	rs4894047	chr2:179,390,717-179,672,150	AX-156291069
TTN	---	chr2:179,390,717-179,672,150	AX-90044204
TTN	rs1905521	chr2:179,390,717-179,672,150	AX-96557849
TTN	rs1905521	chr2:179,390,717-179,672,150	AX-96641418
TTN	---	chr2:179,390,717-179,672,150	AX-33413569
TTN	rs2291306	chr2:179,390,717-179,672,150	AX-12524599
TTN	rs146970027	chr2:179,390,717-179,672,150	AX-83271288
TTN	rs4894048	chr2:179,390,717-179,672,150	AX-11534159
TTN	---	chr2:179,390,717-179,672,150	AX-94375774
TTN	rs3816781	chr2:179,390,717-179,672,150	AX-40776967
TTN	rs4894049	chr2:179,390,717-179,672,150	AX-13768099
TTN	rs189149543	chr2:179,390,717-179,672,150	AX-83552914
TTN	rs146496197	chr2:179,390,717-179,672,150	AX-83251265
TTN	rs141213991	chr2:179,390,717-179,672,150	AX-82949183
TTN	rs12476289	chr2:179,390,717-179,672,150	AX-83045848
TTN	rs719201	chr2:179,390,717-179,672,150	AX-12624057
TTN	---	chr2:179,390,717-179,672,150	AX-83521160
TTN	rs56180285	chr2:179,390,717-179,672,150	AX-13768105
TTN	rs72647873	chr2:179,390,717-179,672,150	AX-33413577
TTN	rs16866531	chr2:179,390,717-179,672,150	AX-40776975
TTN	rs36021856	chr2:179,390,717-179,672,150	AX-83306114
TTN	rs2291301	chr2:179,390,717-179,672,150	AX-156265654
TTN	rs2291301	chr2:179,390,717-179,672,150	AX-156265689
TTN	rs1552280	chr2:179,390,717-179,672,150	AX-96716216
TTN	rs1552280	chr2:179,390,717-179,672,150	AX-96726428
TTN	rs2035496	chr2:179,390,717-179,672,150	AX-40776985
TTN	rs6709235	chr2:179,390,717-179,672,150	AX-33413583
TTN	rs150667217	chr2:179,390,717-179,672,150	AX-83229378
TTN	rs10497520	chr2:179,390,717-179,672,150	AX-11111536
TTN	rs12693168	chr2:179,390,717-179,672,150	AX-156273134
TTN	rs12693168	chr2:179,390,717-179,672,150	AX-156291070
TTN	rs2054708	chr2:179,390,717-179,672,150	AX-12515607
TTN	rs72647870	chr2:179,390,717-179,672,150	AX-83308005
TTN	rs2054707	chr2:179,390,717-179,672,150	AX-156273135
TTN	rs2054707	chr2:179,390,717-179,672,150	AX-156291071
TTN	rs2054706	chr2:179,390,717-179,672,150	AX-84615667
TTN	rs2054706	chr2:179,390,717-179,672,150	AX-84627284
TTN	rs55914517	chr2:179,390,717-179,672,150	AX-83064566
TTN	rs142951505	chr2:179,390,717-179,672,150	AX-83225654
TTN	---	chr2:179,390,717-179,672,150	AX-90055164
TTN	rs60305852	chr2:179,390,717-179,672,150	AX-156265669
TTN	rs60305852	chr2:179,390,717-179,672,150	AX-156265704
TTN	rs2306637	chr2:179,390,717-179,672,150	AX-156281482

TTN	rs2306637	chr2:179,390,717-179,672,150	AX-156296423
TTN	rs67143080	chr2:179,390,717-179,672,150	AX-121418904
TTN	rs67143080	chr2:179,390,717-179,672,150	AX-96637421
TTN	rs6729746	chr2:179,390,717-179,672,150	AX-33413593
TTN	rs3769863	chr2:179,390,717-179,672,150	AX-11479323
TTN	rs6733291	chr2:179,390,717-179,672,150	AX-40776997
TTN	rs3835920	chr2:179,390,717-179,672,150	AX-88767258
TTN	rs3835920	chr2:179,390,717-179,672,150	AX-88810303
TTN	rs35813871	chr2:179,390,717-179,672,150	AX-33413599
TTN	rs6715406	chr2:179,390,717-179,672,150	AX-11580276
TTN	rs267607157	chr2:179,390,717-179,672,150	AX-90055165
TTN	rs28933405	chr2:179,390,717-179,672,150	AX-88782118
TTN	rs6715901	chr2:179,390,717-179,672,150	AX-11580309
TTN	rs10497521	chr2:179,390,717-179,672,150	AX-40776999
TTN	rs7423101	chr2:179,390,717-179,672,150	AX-96631666
TTN	rs11897386	chr2:179,390,717-179,672,150	AX-96554866
TTN	rs11892354	chr2:179,390,717-179,672,150	AX-96551553
TTN	rs11892354	chr2:179,390,717-179,672,150	AX-96635122
TTN	rs10497523	chr2:179,390,717-179,672,150	AX-40777011
TTN	rs111598947	chr2:179,390,717-179,672,150	AX-121725293
TTN	rs10184892	chr2:179,390,717-179,672,150	AX-12384350
TTN	rs67587484	chr2:179,390,717-179,672,150	AX-13768123
TTN	rs72957312	chr2:179,390,717-179,672,150	AX-33413607
TTN	rs79491116	chr2:179,390,717-179,672,150	AX-13768126
TTN	rs72647851	chr2:179,390,717-179,672,150	AX-83456618
TTN	rs16866537	chr2:179,390,717-179,672,150	AX-40777015
TTN	---	chr2:179,390,717-179,672,150	AX-94359025
TTN	---	chr2:179,390,717-179,672,150	AX-94359026
TTN	rs146000949	chr2:179,390,717-179,672,150	AX-83022209
TTN	rs2291303	chr2:179,390,717-179,672,150	AX-50339964
TTN	rs56128843	chr2:179,390,717-179,672,150	AX-83051946
TTN	rs16866538	chr2:179,390,717-179,672,150	AX-11285041
TTN	rs3754950	chr2:179,390,717-179,672,150	AX-12560064
TTN	rs3754949	chr2:179,390,717-179,672,150	AX-40777019
TTN	rs73973151	chr2:179,390,717-179,672,150	AX-13768135
TTN	rs10497524	chr2:179,390,717-179,672,150	AX-40777023
TTN	rs146858785	chr2:179,390,717-179,672,150	AX-96549277
TTN	rs146858785	chr2:179,390,717-179,672,150	AX-96632846
TTN	rs66733621	chr2:179,390,717-179,672,150	AX-33413621
TTN	rs376737897	chr2:179,390,717-179,672,150	AX-92812888
TTN	---	chr2:179,390,717-179,672,150	AX-94375777
TTN	rs13420249	chr2:179,390,717-179,672,150	AX-40777027
TTN	rs3769862	chr2:179,390,717-179,672,150	AX-156281483
TTN	rs3769862	chr2:179,390,717-179,672,150	AX-156296424
TTN	rs11321728	chr2:179,390,717-179,672,150	AX-122745946
TTN	rs11321728	chr2:179,390,717-179,672,150	AX-156273136
TTN	rs35683768	chr2:179,390,717-179,672,150	AX-11468190
TTN	rs139517732	chr2:179,390,717-179,672,150	AX-86699884
TTN	rs3816849	chr2:179,390,717-179,672,150	AX-12562640
TTN	rs3769858	chr2:179,390,717-179,672,150	AX-147899821
TTN	rs1872202	chr2:179,390,717-179,672,150	AX-40777037
TTN	rs111272780	chr2:179,390,717-179,672,150	AX-148606868
TTN	rs111272780	chr2:179,390,717-179,672,150	AX-156278755
TTN	rs111692972	chr2:179,390,717-179,672,150	AX-148290885
TTN	rs111692972	chr2:179,390,717-179,672,150	AX-156278756

TTN	rs60137285	chr2:179,390,717-179,672,150	AX-13768148
TTN	rs4894050	chr2:179,390,717-179,672,150	AX-40777041
TTN	rs10497525	chr2:179,390,717-179,672,150	AX-156273137
TTN	rs10497525	chr2:179,390,717-179,672,150	AX-156291072
TTN	rs2279472	chr2:179,390,717-179,672,150	AX-40777049
TTN	rs72647838	chr2:179,390,717-179,672,150	AX-88773193
TTN	rs72647838	chr2:179,390,717-179,672,150	AX-88816238
VCL	rs67672457	chr10:75,754,951-75,879,914	AX-29713163
VCL	rs7914216	chr10:75,754,951-75,879,914	AX-88733589
VCL	rs147211033	chr10:75,754,951-75,879,914	AX-88745847
VCL	rs147211033	chr10:75,754,951-75,879,914	AX-88788892
VCL	---	chr10:75,754,951-75,879,914	AX-16394693
VCL	rs111264881	chr10:75,754,951-75,879,914	AX-29713167
VCL	rs3812624	chr10:75,754,951-75,879,914	AX-92805110
VCL	rs3812625	chr10:75,754,951-75,879,914	AX-92802538
VCL	rs3812625	chr10:75,754,951-75,879,914	AX-92805126
VCL	rs114200964	chr10:75,754,951-75,879,914	AX-29713169
VCL	rs7923778	chr10:75,754,951-75,879,914	AX-38877091
VCL	rs7079796	chr10:75,754,951-75,879,914	AX-16394697
VCL	rs116822498	chr10:75,754,951-75,879,914	AX-156278515
VCL	rs116822498	chr10:75,754,951-75,879,914	AX-156294773
VCL	rs35630135	chr10:75,754,951-75,879,914	AX-120393341
VCL	rs35630135	chr10:75,754,951-75,879,914	AX-156297707
VCL	rs114123848	chr10:75,754,951-75,879,914	AX-148896762
VCL	rs114123848	chr10:75,754,951-75,879,914	AX-156278516
VCL	rs12357316	chr10:75,754,951-75,879,914	AX-11202084
VCL	rs114993631	chr10:75,754,951-75,879,914	AX-16394701
VCL	rs12250729	chr10:75,754,951-75,879,914	AX-12431594
VCL	rs142835655	chr10:75,754,951-75,879,914	AX-148858527
VCL	rs142835655	chr10:75,754,951-75,879,914	AX-88326285
VCL	rs12245468	chr10:75,754,951-75,879,914	AX-123037750
VCL	rs12245468	chr10:75,754,951-75,879,914	AX-156278517
VCL	rs547779787	chr10:75,754,951-75,879,914	AX-151453946
VCL	rs547779787	chr10:75,754,951-75,879,914	AX-156285717
VCL	rs11815936	chr10:75,754,951-75,879,914	AX-120856526
VCL	rs67017494	chr10:75,754,951-75,879,914	AX-156294774
VCL	rs11000842	chr10:75,754,951-75,879,914	AX-148888642
VCL	rs11000842	chr10:75,754,951-75,879,914	AX-156278519
VCL	rs76231488	chr10:75,754,951-75,879,914	AX-113098546
VCL	rs76231488	chr10:75,754,951-75,879,914	AX-113616728
VCL	rs2395075	chr10:75,754,951-75,879,914	AX-16394707
VCL	rs6480714	chr10:75,754,951-75,879,914	AX-11567087
VCL	rs200588325	chr10:75,754,951-75,879,914	AX-156284261
VCL	rs202191446	chr10:75,754,951-75,879,914	AX-156284849
VCL	rs59096058	chr10:75,754,951-75,879,914	AX-151168270
VCL	rs184142844	chr10:75,754,951-75,879,914	AX-92714853
VCL	rs184142844	chr10:75,754,951-75,879,914	AX-92786587
VCL	rs11000845	chr10:75,754,951-75,879,914	AX-148896004
VCL	rs11000845	chr10:75,754,951-75,879,914	AX-156278520
VCL	rs116843906	chr10:75,754,951-75,879,914	AX-29713205
VCL	rs1403629	chr10:75,754,951-75,879,914	AX-11260334
VCL	rs7915720	chr10:75,754,951-75,879,914	AX-121344740
VCL	rs7915720	chr10:75,754,951-75,879,914	AX-123021478
VCL	rs57348795	chr10:75,754,951-75,879,914	AX-148820050
VCL	rs57348795	chr10:75,754,951-75,879,914	AX-156283619

VCL	rs12776171	chr10:75,754,951-75,879,914	AX-122259502
VCL	rs12776171	chr10:75,754,951-75,879,914	AX-148193719
VCL	rs11000847	chr10:75,754,951-75,879,914	AX-148110786
VCL	rs11000848	chr10:75,754,951-75,879,914	AX-29713215
VCL	rs145980369	chr10:75,754,951-75,879,914	AX-156283620
VCL	rs11000849	chr10:75,754,951-75,879,914	AX-148325833
VCL	rs11000849	chr10:75,754,951-75,879,914	AX-156279433
VCL	---	chr10:75,754,951-75,879,914	AX-86901307
VCL	rs143307823	chr10:75,754,951-75,879,914	AX-151227653
VCL	rs143307823	chr10:75,754,951-75,879,914	AX-156285205
VCL	rs141891105	chr10:75,754,951-75,879,914	AX-120365552
VCL	rs141891105	chr10:75,754,951-75,879,914	AX-156284929
VCL	rs11000850	chr10:75,754,951-75,879,914	AX-156278521
VCL	rs11000850	chr10:75,754,951-75,879,914	AX-156294775
VCL	rs202084961	chr10:75,754,951-75,879,914	AX-151386239
VCL	rs10762571	chr10:75,754,951-75,879,914	AX-121300198
VCL	rs10762571	chr10:75,754,951-75,879,914	AX-156278522
VCL	rs4746166	chr10:75,754,951-75,879,914	AX-123046264
VCL	rs4746166	chr10:75,754,951-75,879,914	AX-123046265
VCL	rs1965491	chr10:75,754,951-75,879,914	AX-121228575
VCL	rs1965491	chr10:75,754,951-75,879,914	AX-122995081
VCL	rs7074651	chr10:75,754,951-75,879,914	AX-148229363
VCL	rs7074651	chr10:75,754,951-75,879,914	AX-148346452
VCL	rs7090820	chr10:75,754,951-75,879,914	AX-151234253
VCL	rs7090820	chr10:75,754,951-75,879,914	AX-156283621
VCL	rs78398862	chr10:75,754,951-75,879,914	AX-156278523
VCL	rs78398862	chr10:75,754,951-75,879,914	AX-156294776
VCL	rs10458640	chr10:75,754,951-75,879,914	AX-11107690
VCL	---	chr10:75,754,951-75,879,914	AX-11107692
VCL	rs74146309	chr10:75,754,951-75,879,914	AX-16394722
VCL	rs11000851	chr10:75,754,951-75,879,914	AX-156294777
VCL	rs12570572	chr10:75,754,951-75,879,914	AX-29713221
VCL	rs10824054	chr10:75,754,951-75,879,914	AX-156278524
VCL	rs7075862	chr10:75,754,951-75,879,914	AX-156278525
VCL	rs7075862	chr10:75,754,951-75,879,914	AX-156294778
VCL	rs11000852	chr10:75,754,951-75,879,914	AX-38877105
VCL	rs11000853	chr10:75,754,951-75,879,914	AX-12403724
VCL	rs11000854	chr10:75,754,951-75,879,914	AX-11137256
VCL	rs10824055	chr10:75,754,951-75,879,914	AX-156278526
VCL	rs10824055	chr10:75,754,951-75,879,914	AX-96646574
VCL	rs11000855	chr10:75,754,951-75,879,914	AX-156278527
VCL	rs11000855	chr10:75,754,951-75,879,914	AX-156294779
VCL	rs7069584	chr10:75,754,951-75,879,914	AX-38877111
VCL	rs147299322	chr10:75,754,951-75,879,914	AX-151427644
VCL	rs147299322	chr10:75,754,951-75,879,914	AX-156283622
VCL	rs11000856	chr10:75,754,951-75,879,914	AX-121866380
VCL	rs11000856	chr10:75,754,951-75,879,914	AX-148510434
VCL	rs79708660	chr10:75,754,951-75,879,914	AX-105099211
VCL	rs79708660	chr10:75,754,951-75,879,914	AX-119612410
VCL	rs7914469	chr10:75,754,951-75,879,914	AX-123056462
VCL	rs7914469	chr10:75,754,951-75,879,914	AX-123056463
VCL	rs11000857	chr10:75,754,951-75,879,914	AX-114211625
VCL	rs11000857	chr10:75,754,951-75,879,914	AX-147864042
VCL	rs10824058	chr10:75,754,951-75,879,914	AX-148147467
VCL	rs10824058	chr10:75,754,951-75,879,914	AX-156278528

VCL	rs182237817	chr10:75,754,951-75,879,914	AX-148755192
VCL	rs182237817	chr10:75,754,951-75,879,914	AX-156283623
VCL	rs201254748	chr10:75,754,951-75,879,914	AX-122990357
VCL	rs201254748	chr10:75,754,951-75,879,914	AX-151142760
VCL	rs199882142	chr10:75,754,951-75,879,914	AX-148476971
VCL	rs113534623	chr10:75,754,951-75,879,914	AX-156269244
VCL	rs113534623	chr10:75,754,951-75,879,914	AX-156288761
VCL	rs12413620	chr10:75,754,951-75,879,914	AX-11204324
VCL	rs4745730	chr10:75,754,951-75,879,914	AX-12580028
VCL	rs7099171	chr10:75,754,951-75,879,914	AX-16394728
VCL	rs79424913	chr10:75,754,951-75,879,914	AX-16394732
VCL	rs7084014	chr10:75,754,951-75,879,914	AX-38877121
VCL	rs55947600	chr10:75,754,951-75,879,914	AX-148318063
VCL	rs55947600	chr10:75,754,951-75,879,914	AX-156279434
VCL	rs10762573	chr10:75,754,951-75,879,914	AX-38877123
VCL	rs12782972	chr10:75,754,951-75,879,914	AX-38877125
VCL	rs60297489	chr10:75,754,951-75,879,914	AX-16394736
VCL	rs10824060	chr10:75,754,951-75,879,914	AX-156278529
VCL	rs80134912	chr10:75,754,951-75,879,914	AX-16394737
VCL	rs11594818	chr10:75,754,951-75,879,914	AX-156278530
VCL	---	chr10:75,754,951-75,879,914	AX-91996101
VCL	rs149210908	chr10:75,754,951-75,879,914	AX-156283624
VCL	rs12240297	chr10:75,754,951-75,879,914	AX-156278531
VCL	rs12240297	chr10:75,754,951-75,879,914	AX-156294781
VCL	rs12255404	chr10:75,754,951-75,879,914	AX-86898130
VCL	rs12255404	chr10:75,754,951-75,879,914	AX-86901729
VCL	rs11000859	chr10:75,754,951-75,879,914	AX-152093919
VCL	rs61865576	chr10:75,754,951-75,879,914	AX-29713261
VCL	rs10762574	chr10:75,754,951-75,879,914	AX-123021479
VCL	rs10762574	chr10:75,754,951-75,879,914	AX-123021480
VCL	rs11000860	chr10:75,754,951-75,879,914	AX-115159686
VCL	rs11000860	chr10:75,754,951-75,879,914	AX-156278532
VCL	rs3829127	chr10:75,754,951-75,879,914	AX-156278533
VCL	rs3829127	chr10:75,754,951-75,879,914	AX-156294782
VCL	rs4414157	chr10:75,754,951-75,879,914	AX-16394742
VCL	rs12256764	chr10:75,754,951-75,879,914	AX-12431724
VCL	rs11596000	chr10:75,754,951-75,879,914	AX-16394744
VCL	rs12258654	chr10:75,754,951-75,879,914	AX-156278534
VCL	rs12258654	chr10:75,754,951-75,879,914	AX-156294783
VCL	rs146279725	chr10:75,754,951-75,879,914	AX-151404701
VCL	rs146279725	chr10:75,754,951-75,879,914	AX-156298263
VCL	rs7100021	chr10:75,754,951-75,879,914	AX-148139945
VCL	rs7100021	chr10:75,754,951-75,879,914	AX-88778377
VCL	rs10762575	chr10:75,754,951-75,879,914	AX-12395096
VCL	rs80068231	chr10:75,754,951-75,879,914	AX-16394750
VCL	rs200859155	chr10:75,754,951-75,879,914	AX-151183511
VCL	rs201293196	chr10:75,754,951-75,879,914	AX-122633382
VCL	rs7901068	chr10:75,754,951-75,879,914	AX-156278535
VCL	rs7901068	chr10:75,754,951-75,879,914	AX-156294784
VCL	rs10824063	chr10:75,754,951-75,879,914	AX-123039036
VCL	rs10824063	chr10:75,754,951-75,879,914	AX-148168008
VCL	rs56314304	chr10:75,754,951-75,879,914	AX-16394752
VCL	rs12266886	chr10:75,754,951-75,879,914	AX-38877153
VCL	rs11000861	chr10:75,754,951-75,879,914	AX-148312613
VCL	rs11000861	chr10:75,754,951-75,879,914	AX-156279435

VCL	rs11000862	chr10:75,754,951-75,879,914	AX-148826571
VCL	rs11000862	chr10:75,754,951-75,879,914	AX-156279436
VCL	rs4587673	chr10:75,754,951-75,879,914	AX-121062839
VCL	rs4587673	chr10:75,754,951-75,879,914	AX-156279437
VCL	rs74496529	chr10:75,754,951-75,879,914	AX-16394756
VCL	rs4268450	chr10:75,754,951-75,879,914	AX-16394758
VCL	rs11000864	chr10:75,754,951-75,879,914	AX-16394759
VCL	rs10824064	chr10:75,754,951-75,879,914	AX-148313454
VCL	rs10824064	chr10:75,754,951-75,879,914	AX-156278536
VCL	rs150376237	chr10:75,754,951-75,879,914	AX-105128765
VCL	rs150376237	chr10:75,754,951-75,879,914	AX-148845503
VCL	rs11000865	chr10:75,754,951-75,879,914	AX-12403725
VCL	rs11000866	chr10:75,754,951-75,879,914	AX-156278537
VCL	rs11000866	chr10:75,754,951-75,879,914	AX-156294785
VCL	rs56361489	chr10:75,754,951-75,879,914	AX-29713305
VCL	rs10824065	chr10:75,754,951-75,879,914	AX-115136012
VCL	rs10824065	chr10:75,754,951-75,879,914	AX-156278538
VCL	rs7917915	chr10:75,754,951-75,879,914	AX-148623768
VCL	rs7917915	chr10:75,754,951-75,879,914	AX-156278539
VCL	rs7921019	chr10:75,754,951-75,879,914	AX-151344894
VCL	rs7921019	chr10:75,754,951-75,879,914	AX-156278540
VCL	rs7098158	chr10:75,754,951-75,879,914	AX-16394764
VCL	rs74146311	chr10:75,754,951-75,879,914	AX-16394765
VCL	rs74146312	chr10:75,754,951-75,879,914	AX-29713309
VCL	rs60709638	chr10:75,754,951-75,879,914	AX-16394767
VCL	rs57273561	chr10:75,754,951-75,879,914	AX-16394768
VCL	rs59090270	chr10:75,754,951-75,879,914	AX-148621203
VCL	rs59090270	chr10:75,754,951-75,879,914	AX-156278541
VCL	rs9663522	chr10:75,754,951-75,879,914	AX-156278542
VCL	rs9663522	chr10:75,754,951-75,879,914	AX-156294786
VCL	rs111598996	chr10:75,754,951-75,879,914	AX-151347620
VCL	rs111598996	chr10:75,754,951-75,879,914	AX-156278486
VCL	rs60819773	chr10:75,754,951-75,879,914	AX-16394769
VCL	rs57384313	chr10:75,754,951-75,879,914	AX-16394770
VCL	rs143197525	chr10:75,754,951-75,879,914	AX-96652689
VCL	rs4357629	chr10:75,754,951-75,879,914	AX-120383327
VCL	rs7916635	chr10:75,754,951-75,879,914	AX-122990359
VCL	rs7916635	chr10:75,754,951-75,879,914	AX-122990360
VCL	rs61865578	chr10:75,754,951-75,879,914	AX-148331304
VCL	rs112985803	chr10:75,754,951-75,879,914	AX-151352707
VCL	rs112985803	chr10:75,754,951-75,879,914	AX-156283625
VCL	rs57828435	chr10:75,754,951-75,879,914	AX-16394772
VCL	rs201304453	chr10:75,754,951-75,879,914	AX-151344704
VCL	rs201304453	chr10:75,754,951-75,879,914	AX-156286347
VCL	rs146473758	chr10:75,754,951-75,879,914	AX-151342924
VCL	rs146473758	chr10:75,754,951-75,879,914	AX-156284244
VCL	rs12253805	chr10:75,754,951-75,879,914	AX-38877159
VCL	rs74146313	chr10:75,754,951-75,879,914	AX-16394775
VCL	rs200410168	chr10:75,754,951-75,879,914	AX-156284454
VCL	rs3012027	chr10:75,754,951-75,879,914	AX-148326969
VCL	rs3012027	chr10:75,754,951-75,879,914	AX-156278543
VCL	rs3012028	chr10:75,754,951-75,879,914	AX-148166739
VCL	rs3012028	chr10:75,754,951-75,879,914	AX-156278544
VCL	rs7893217	chr10:75,754,951-75,879,914	AX-88789478
VCL	rs7909764	chr10:75,754,951-75,879,914	AX-120260764

VCL	rs7909764	chr10:75,754,951-75,879,914	AX-88739502
VCL	rs59511859	chr10:75,754,951-75,879,914	AX-16394777
VCL	rs7893758	chr10:75,754,951-75,879,914	AX-16394778
VCL	rs7893659	chr10:75,754,951-75,879,914	AX-38877163
VCL	rs7894127	chr10:75,754,951-75,879,914	AX-38877165
VCL	rs7910622	chr10:75,754,951-75,879,914	AX-156278545
VCL	rs7910622	chr10:75,754,951-75,879,914	AX-156294787
VCL	rs7910748	chr10:75,754,951-75,879,914	AX-148635003
VCL	rs7910748	chr10:75,754,951-75,879,914	AX-156278546
VCL	rs56079825	chr10:75,754,951-75,879,914	AX-16394780
VCL	rs59698250	chr10:75,754,951-75,879,914	AX-16394781
VCL	rs35828059	chr10:75,754,951-75,879,914	AX-38877167
VCL	---	chr10:75,754,951-75,879,914	AX-90026460
VCL	rs56314318	chr10:75,754,951-75,879,914	AX-16394782
VCL	rs113195070	chr10:75,754,951-75,879,914	AX-37482875
VCL	rs2131960	chr10:75,754,951-75,879,914	AX-11369321
VCL	rs4745731	chr10:75,754,951-75,879,914	AX-156278547
VCL	rs4745731	chr10:75,754,951-75,879,914	AX-156294788
VCL	rs74146314	chr10:75,754,951-75,879,914	AX-156279438
VCL	rs74146314	chr10:75,754,951-75,879,914	AX-156295135
VCL	rs74146315	chr10:75,754,951-75,879,914	AX-156279439
VCL	rs74146315	chr10:75,754,951-75,879,914	AX-156295136
VCL	rs79479684	chr10:75,754,951-75,879,914	AX-151344818
VCL	rs79479684	chr10:75,754,951-75,879,914	AX-156278548
VCL	rs71524373	chr10:75,754,951-75,879,914	AX-148618519
VCL	rs71524384	chr10:75,754,951-75,879,914	AX-156283626
VCL	rs71524384	chr10:75,754,951-75,879,914	AX-92538506
VCL	rs111609277	chr10:75,754,951-75,879,914	AX-156278549
VCL	rs74146316	chr10:75,754,951-75,879,914	AX-16394786
VCL	rs74146317	chr10:75,754,951-75,879,914	AX-16394787
VCL	rs144075927	chr10:75,754,951-75,879,914	AX-156283627
VCL	rs144075927	chr10:75,754,951-75,879,914	AX-94367448
VCL	rs199568482	chr10:75,754,951-75,879,914	AX-156286247
VCL	rs76932597	chr10:75,754,951-75,879,914	AX-151346899
VCL	rs76932597	chr10:75,754,951-75,879,914	AX-156278550
VCL	rs112743588	chr10:75,754,951-75,879,914	AX-148618477
VCL	rs112743588	chr10:75,754,951-75,879,914	AX-156278551
VCL	rs61584917	chr10:75,754,951-75,879,914	AX-156278552
VCL	rs61584917	chr10:75,754,951-75,879,914	AX-16476905
VCL	rs58332888	chr10:75,754,951-75,879,914	AX-16394788
VCL	rs60563569	chr10:75,754,951-75,879,914	AX-156278553
VCL	rs60563569	chr10:75,754,951-75,879,914	AX-156294789
VCL	rs58026892	chr10:75,754,951-75,879,914	AX-156278554
VCL	rs58026892	chr10:75,754,951-75,879,914	AX-156294790
VCL	rs56413529	chr10:75,754,951-75,879,914	AX-16394793
VCL	rs4745733	chr10:75,754,951-75,879,914	AX-29713353
VCL	rs4745734	chr10:75,754,951-75,879,914	AX-156278555
VCL	rs4745734	chr10:75,754,951-75,879,914	AX-156294791
VCL	rs4745735	chr10:75,754,951-75,879,914	AX-156278556
VCL	rs4745735	chr10:75,754,951-75,879,914	AX-156294792
VCL	rs201182836	chr10:75,754,951-75,879,914	AX-151343752
VCL	rs201182836	chr10:75,754,951-75,879,914	AX-156284681
VCL	rs189064054	chr10:75,754,951-75,879,914	AX-156278557
VCL	rs189064054	chr10:75,754,951-75,879,914	AX-156294793
VCL	rs4745736	chr10:75,754,951-75,879,914	AX-16394794

VCL	rs4746169	chr10:75,754,951-75,879,914	AX-38877175
VCL	rs74146318	chr10:75,754,951-75,879,914	AX-156278558
VCL	rs74146318	chr10:75,754,951-75,879,914	AX-156294794
VCL	rs1874152	chr10:75,754,951-75,879,914	AX-11348904
VCL	rs2934773	chr10:75,754,951-75,879,914	AX-38877177
VCL	rs55724951	chr10:75,754,951-75,879,914	AX-16394795
VCL	rs112780265	chr10:75,754,951-75,879,914	AX-156298509
VCL	rs112780265	chr10:75,754,951-75,879,914	AX-16394797
VCL	rs1908337	chr10:75,754,951-75,879,914	AX-156278559
VCL	rs74146320	chr10:75,754,951-75,879,914	AX-16394799
VCL	rs144857138	chr10:75,754,951-75,879,914	AX-156284874
VCL	rs74146321	chr10:75,754,951-75,879,914	AX-16394800
VCL	rs55782045	chr10:75,754,951-75,879,914	AX-156278560
VCL	rs55782045	chr10:75,754,951-75,879,914	AX-156294795
VCL	rs1908338	chr10:75,754,951-75,879,914	AX-156278561
VCL	rs1908338	chr10:75,754,951-75,879,914	AX-156294796
VCL	rs35830669	chr10:75,754,951-75,879,914	AX-121116318
VCL	rs35830669	chr10:75,754,951-75,879,914	AX-156278562
VCL	rs74146323	chr10:75,754,951-75,879,914	AX-156278563
VCL	rs74146323	chr10:75,754,951-75,879,914	AX-156294797
VCL	rs145629058	chr10:75,754,951-75,879,914	AX-156297998
VCL	rs145629058	chr10:75,754,951-75,879,914	AX-16394804
VCL	rs80127811	chr10:75,754,951-75,879,914	AX-16394806
VCL	rs2131961	chr10:75,754,951-75,879,914	AX-38877183
VCL	rs11000868	chr10:75,754,951-75,879,914	AX-11137257
VCL	rs60612652	chr10:75,754,951-75,879,914	AX-16394807
VCL	rs2270548	chr10:75,754,951-75,879,914	AX-11380079
VCL	rs71579353	chr10:75,754,951-75,879,914	AX-83465722
VCL	rs5786133	chr10:75,754,951-75,879,914	AX-151144919
VCL	rs1908339	chr10:75,754,951-75,879,914	AX-38877187
VCL	rs61731180	chr10:75,754,951-75,879,914	AX-16394809
VCL	---	chr10:75,754,951-75,879,914	AX-88320415
VCL	rs7076458	chr10:75,754,951-75,879,914	AX-114075802
VCL	rs7076458	chr10:75,754,951-75,879,914	AX-156278564
VCL	rs4746170	chr10:75,754,951-75,879,914	AX-151181862
VCL	rs11599181	chr10:75,754,951-75,879,914	AX-148428651
VCL	rs4745737	chr10:75,754,951-75,879,914	AX-148632449
VCL	rs4745737	chr10:75,754,951-75,879,914	AX-156269246
VCL	rs12571772	chr10:75,754,951-75,879,914	AX-151240545
VCL	rs12571772	chr10:75,754,951-75,879,914	AX-156283628
VCL	rs186352082	chr10:75,754,951-75,879,914	AX-151300472
VCL	rs191660137	chr10:75,754,951-75,879,914	AX-149885061
VCL	rs191660137	chr10:75,754,951-75,879,914	AX-92758910
VCL	rs12772220	chr10:75,754,951-75,879,914	AX-148101608
VCL	rs12772220	chr10:75,754,951-75,879,914	AX-152037317
VCL	rs11596526	chr10:75,754,951-75,879,914	AX-50025143
VCL	rs11000869	chr10:75,754,951-75,879,914	AX-92802523
VCL	rs76974852	chr10:75,754,951-75,879,914	AX-29713371
VCL	rs7083479	chr10:75,754,951-75,879,914	AX-38877193
VCL	rs7908476	chr10:75,754,951-75,879,914	AX-16394817
VCL	---	chr10:75,754,951-75,879,914	AX-12600217
VCL	---	chr10:75,754,951-75,879,914	AX-94360564
VCL	rs7073889	chr10:75,754,951-75,879,914	AX-38877195
VCL	rs10652052	chr10:75,754,951-75,879,914	AX-151332521
VCL	rs10652052	chr10:75,754,951-75,879,914	AX-156298363

VCL	rs146278697	chr10:75,754,951-75,879,914	AX-82890782
VCL	rs374522164	chr10:75,754,951-75,879,914	AX-86739828
VCL	rs12258268	chr10:75,754,951-75,879,914	AX-122783342
VCL	rs12258268	chr10:75,754,951-75,879,914	AX-149505377
VCL	rs111748583	chr10:75,754,951-75,879,914	AX-16394823
VCL	rs138676040	chr10:75,754,951-75,879,914	AX-156285974
VCL	rs113750112	chr10:75,754,951-75,879,914	AX-148395674
VCL	rs113750112	chr10:75,754,951-75,879,914	AX-156283629
VCL	rs113584681	chr10:75,754,951-75,879,914	AX-105108221
VCL	rs113584681	chr10:75,754,951-75,879,914	AX-156283630
VCL	rs16931166	chr10:75,754,951-75,879,914	AX-38877199
VCL	rs143750279	chr10:75,754,951-75,879,914	AX-148652385
VCL	rs147134358	chr10:75,754,951-75,879,914	AX-106835873
VCL	rs147134358	chr10:75,754,951-75,879,914	AX-120676748
VCL	rs11000870	chr10:75,754,951-75,879,914	AX-148389880
VCL	rs11000870	chr10:75,754,951-75,879,914	AX-156278565
VCL	rs75684366	chr10:75,754,951-75,879,914	AX-16394826
VCL	rs74146324	chr10:75,754,951-75,879,914	AX-156278566
VCL	rs10824069	chr10:75,754,951-75,879,914	AX-16394827
VCL	rs2229507	chr10:75,754,951-75,879,914	AX-121213938
VCL	rs2229507	chr10:75,754,951-75,879,914	AX-156265699
VCL	rs2228373	chr10:75,754,951-75,879,914	AX-16394828
VCL	rs141033098	chr10:75,754,951-75,879,914	AX-83308422
VCL	rs4746172	chr10:75,754,951-75,879,914	AX-12580040
VCL	---	chr10:75,754,951-75,879,914	AX-156285609
VCL	rs12243178	chr10:75,754,951-75,879,914	AX-16394835
VCL	rs74146325	chr10:75,754,951-75,879,914	AX-16394836
VCL	rs60384973	chr10:75,754,951-75,879,914	AX-16394838
VCL	rs140966874	chr10:75,754,951-75,879,914	AX-151272182
VCL	rs140966874	chr10:75,754,951-75,879,914	AX-156298042
VCL	rs79993375	chr10:75,754,951-75,879,914	AX-29713399
VCL	rs74146326	chr10:75,754,951-75,879,914	AX-16394839
VCL	rs17743339	chr10:75,754,951-75,879,914	AX-38877207
VCL	rs71579374	chr10:75,754,951-75,879,914	AX-83492773
VCL	rs199610574	chr10:75,754,951-75,879,914	AX-151425244
VCL	rs199610574	chr10:75,754,951-75,879,914	AX-156284383
VCL	rs11000872	chr10:75,754,951-75,879,914	AX-92647212
VCL	rs11000872	chr10:75,754,951-75,879,914	AX-92670978
VCL	rs7912297	chr10:75,754,951-75,879,914	AX-123021481
VCL	rs7912297	chr10:75,754,951-75,879,914	AX-123021482
VCL	rs10709887	chr10:75,754,951-75,879,914	AX-122464424
VCL	rs2270552	chr10:75,754,951-75,879,914	AX-11380080
VCL	rs2131959	chr10:75,754,951-75,879,914	AX-38877215
VCL	rs11000874	chr10:75,754,951-75,879,914	AX-38877217
VCL	rs767809	chr10:75,754,951-75,879,914	AX-16394843
VCL	rs57050827	chr10:75,754,951-75,879,914	AX-16394844
VCL	rs2131957	chr10:75,754,951-75,879,914	AX-16394845
VCL	rs2279648	chr10:75,754,951-75,879,914	AX-16394846
VCL	rs1874151	chr10:75,754,951-75,879,914	AX-29713417
VCL	rs1874150	chr10:75,754,951-75,879,914	AX-16394848
VCL	rs3793921	chr10:75,754,951-75,879,914	AX-16394849
VCL	rs2270551	chr10:75,754,951-75,879,914	AX-16394852
VCL	rs10824070	chr10:75,754,951-75,879,914	AX-16394853
VCL	rs7909664	chr10:75,754,951-75,879,914	AX-38877219
VCL	rs12415418	chr10:75,754,951-75,879,914	AX-16394855

VCL	rs16931177	chr10:75,754,951-75,879,914	AX-38877223
VCL	rs2131956	chr10:75,754,951-75,879,914	AX-16394859
VCL	rs34885185	chr10:75,754,951-75,879,914	AX-38877227
VCL	rs10824071	chr10:75,754,951-75,879,914	AX-12397512
VCL	rs10824072	chr10:75,754,951-75,879,914	AX-156265666
VCL	rs10824072	chr10:75,754,951-75,879,914	AX-156265700
VCL	rs397517238	chr10:75,754,951-75,879,914	AX-91983660
VCL	rs10824073	chr10:75,754,951-75,879,914	AX-16394862
VCL	rs6480716	chr10:75,754,951-75,879,914	AX-16394863
VCL	rs7922894	chr10:75,754,951-75,879,914	AX-16394864
VCL	rs7100462	chr10:75,754,951-75,879,914	AX-156278567
VCL	rs34188235	chr10:75,754,951-75,879,914	AX-156284272
VCL	rs10824074	chr10:75,754,951-75,879,914	AX-119407738
VCL	rs10824074	chr10:75,754,951-75,879,914	AX-120523075
VCL	rs12572820	chr10:75,754,951-75,879,914	AX-151151193
VCL	rs11597732	chr10:75,754,951-75,879,914	AX-151143950
VCL	rs11597732	chr10:75,754,951-75,879,914	AX-156278569
VCL	rs373317423	chr10:75,754,951-75,879,914	AX-86648418
VCL	rs16408	chr10:75,754,951-75,879,914	AX-121237760
VCL	rs16408	chr10:75,754,951-75,879,914	AX-84963749
VCL	rs703258	chr10:75,754,951-75,879,914	AX-38877241
VCL	rs8255	chr10:75,754,951-75,879,914	AX-51242354
VCL	rs8255	chr10:75,754,951-75,879,914	AX-84931175
VCL	rs201189785	chr10:75,754,951-75,879,914	AX-156298659
VCL	rs201189785	chr10:75,754,951-75,879,914	AX-84925963
